# Antennal transcriptome analyses and olfactory protein identification in an important wood-boring moth pest, *Streltzoviella insularis* (Lepidoptera: Cossidae)

**DOI:** 10.1038/s41598-019-54455-w

**Published:** 2019-11-29

**Authors:** Yuchao Yang, Wenbo Li, Jing Tao, Shixiang Zong

**Affiliations:** Beijing Key Laboratory for Forest Pest Control, Beijing Forestry University, Beijing, 100083 China

**Keywords:** Chemical ecology, Next-generation sequencing, Transcriptomics

## Abstract

Olfaction plays key roles in insect survival and reproduction, such as feeding, courtship, mating, and oviposition. The olfactory-based control strategies have been developed an important means for pest management. *Streltzoviella insularis* is a destructive insect pest of many street tree species, and characterization of its olfactory proteins could provide targets for the disruption of their odour recognition processes and for urban forestry protection. In this study, we assembled the antennal transcriptome of *S. insularis* by next-generation sequencing and annotated the main olfactory multi-gene families, including 28 odorant-binding proteins (OBPs), 12 chemosensory proteins (CSPs), 56 odorant receptors (ORs), 11 ionotropic receptors (IRs), two sensory neuron membrane proteins (SNMPs), and 101 odorant-degrading enzymes (ODEs). Sequence and phylogenetic analyses confirmed the characteristics of these proteins. We further detected tissue- and sex-specific expression patterns of OBPs, CSPs and SNMPs by quantitative real time-PCR. Most OBPs were highly and differentially expressed in the antennae of both sexes. *SinsCSP10* was expressed more highly in male antennae than in other tissues. Two SNMPs were highly expressed in the antennae, with no significant difference in expression between the sexes. Our results lay a solid foundation for understanding the precise molecular mechanisms underlying *S. insularis* odour recognition.

## Introduction

Olfaction plays key roles in insect survival and reproduction, and the antennae are regarded as important olfactory organs in insects; they can sensitively detect chemical signals from the environment and produce behavioural reactions, such as feeding, courtship, mating, and oviposition^1–5^. However, these processes cannot occur without the involvement of olfactory proteins expressed in the antennae. In general, olfactory proteins are classified into several categories: odorant-binding proteins (OBPs), chemosensory proteins (CSPs), olfactory receptors (ORs), ionotropic receptors (IRs), sensory neuron membrane proteins (SNMPs) and odorant-degrading enzymes (ODEs)^6–8^.

OBPs and CSPs are highly abundant in the sensillar lymph of insect antennae. They bind to hydrophobic odorant molecules and transport them in a pH-dependent manner to ORs during the first step of olfactory recognition^8–10^. OBPs are small soluble proteins with a pattern of six cysteines that form two or three disulfide bridges, which are typically divided into two subfamilies in Lepidoptera species, pheromone-binding proteins (PBPs) and general odorant-binding proteins (GOBPs)^11^. Besides OBPs, CSPs are smaller and more conserved than OBPs; they are characterized by the presence of four cysteines with two disulfide bridges^7^. Previous studies have verified that CSPs are expressed both in olfactory tissues and non-olfactory tissues, and seemed to play roles in pheromone transport, moulting, development and leg regeneration^12–14^. In addition, SNMPs are olfactory-specific membrane proteins and are homologous to the human fatty-acid transport protein CD36 receptor family^15^. Lepidopterans generally have two SNMP subfamilies (SNMP1 and SNMP2). SNMP1 is expressed on the dendrite membrane of pheromone-sensitive neurons in *Heliothis virescens*, and is a crucial cofactor for the detection of the fatty acid derived pheromone 11-cis-vaccenyl acetate in *Drosophila*. It might be involved in sex pheromone recognition^16–18^. SNMP2 is also associated with pheromone-sensitive sensilla, but it is only expressed in supporting cells^18,19^. Moreover, insect olfactory reception involves two receptor types (ORs and IRs). Insect ORs are heteromultimers formed by two proteins, a conventional OR and an obligate olfactory co-receptor (Orco)^20^. The binding of odorant molecules by the OR/Orco complex triggers the transduction of chemical signals to electrical signals that are transmitted to the brain^21,22^. IRs are a novel family of chemosensory receptors that are related to ionotropic glutamate receptors (iGluRs), and act as ligand-based ion channels^23^. Insect IRs include two subfamilies: the “antennal IRs” and the species-specific “divergent IRs”^24^. Earlier studies have proved that IRs also are involved in odour detection. IRs are narrowly tuned for acids and amines during biological decomposition, and ORs are widely tuned for alcohols and esters^23,25^. After OR activation, olfactory signals must be degraded rapidly to prevent from prolonged olfactory neuronal excitation, and ODEs inactivate odorant molecules by enzymatic degradation in the sensillar lymph of insect antennae, such as carboxylesterase (CEX), aldehyde oxidases (AOX), alcohol dehydrogenase (AD), cytochrome P450 (CYP) and glutathione S-transferases (GST)^26–29^.

*Streltzoviella insularis* (Staudinger) (Lepidoptera: Cossidae) is an important wood-boring insect pest and occurs in many provinces and cities in China. It mainly attacks various street tree species, such as *Fraxinus americana*, *Ginkgo biloba*, *Sophora* spp. and *Ulmus* spp. Once a female *S. insularis* lays eggs in the cracks of the host tree trunk, the larvae of this moth hatch out and then bore into the phloem and xylem, which stresses or kills the infested host trees, seriously threatening urban forestry^30–32^. The difficult detection, high population and long lifecycle of *S. insularis* pose an immense challenge with respect to pest control. Traditional chemical insecticides do not work well against *S. insularis* and can lead to pesticide resistance and damage to human health and urban environments^30^. Based on the key roles of insect olfactory proteins in chemical communication, olfactory-based control strategies have been developed an important means for pest management^33–37^. For example, Jayanthi *et al*. described a “computational reverse chemical ecology” approach for the screening of attractants based on the binding ability of OBPs as an alternative to behavioural bioassays in *Bactrocera dorsalis*^38^. Applying a reverse chemical ecology approach, Choo *et al*. discovered CquiOR36 responds to acetaldehyde, a potent oviposition attractant for *Culex quinquefasciatus*^39^. However, olfactory proteins in *S. insularis* have not been identified. Therefore, we assembled the antennal transcriptome of male and female *S. insularis* and identified a series of putative olfactory proteins (OBPs, CSPs, ORs, IRs, SNMPs and ODEs). We further examined the expression patterns of OBPs, CSPs and SNMPs by quantitative real-time PCR (RT-qPCR) in various tissues of the two sexes. These results lay a solid foundation for understanding the molecular basis of odour recognition in *S. insularis* and other insects, and they provide a basis for the development of new pest control methods targeting the olfactory system in *S. insularis*.

## Results

### Transcriptome sequencing and de novo assembly

**Figure 1:**
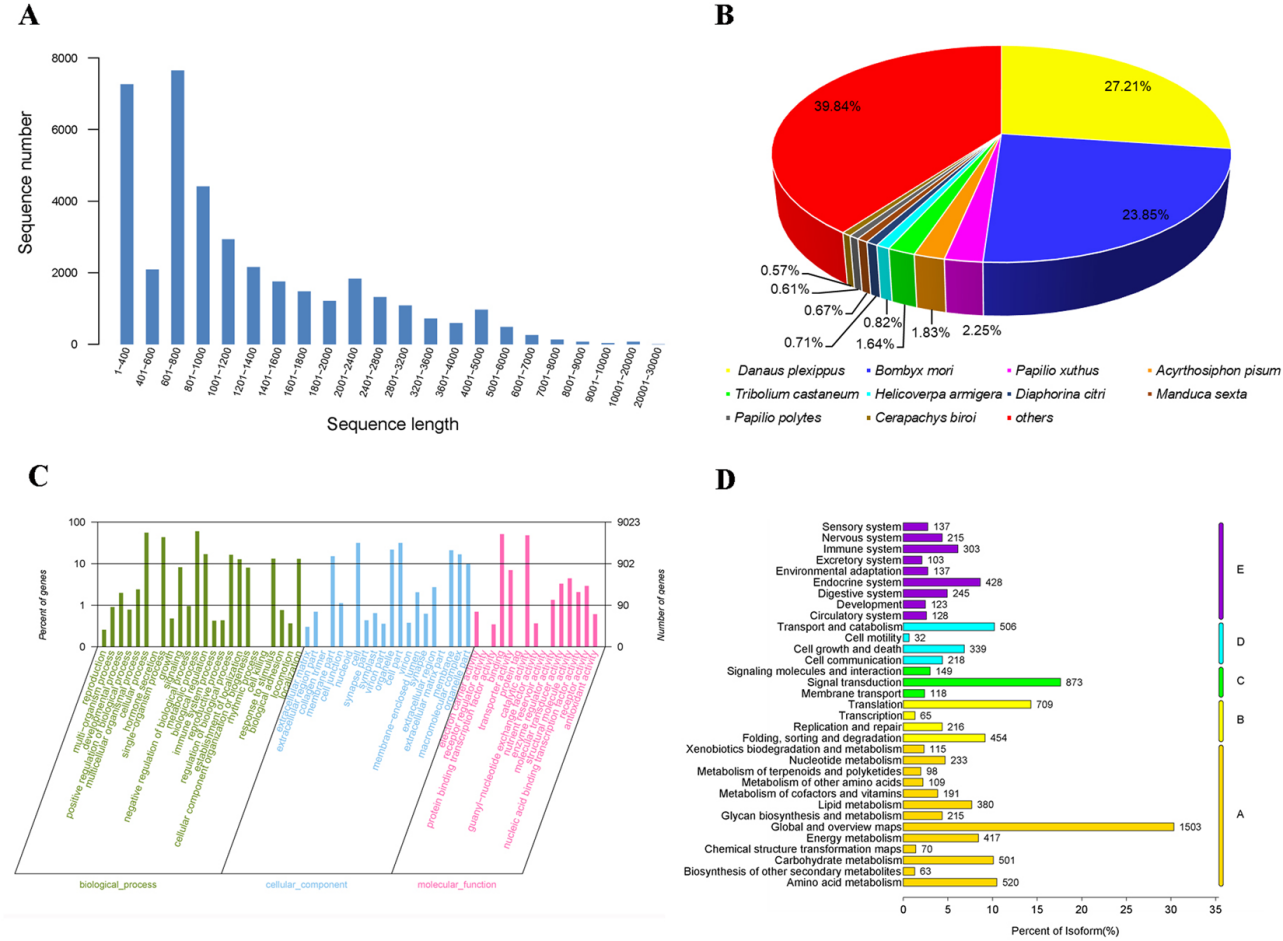
(**A**) Size distribution of *S. insularis* unigenes. (**B**) Species distribution from a homology search with the *S. insularis* unigenes against the NCBI Nr protein database. (**C**) GO classification of *S. insularis* unigenes. (**D**) KEGG classification of *S. insularis* unigenes. (A: processes, B: environmental information processing, C: genetic information processing, D: metabolism, E: organismal systems).

We performed transcriptome sequencing of male and female *S. insularis* antennae to identify olfactory multi-gene families, with three replicates per sex. We obtained approximately 52.6 million and 58.8 million raw reads from the antennal cDNA libraries of male and female *S. insularis*, respectively (see Supplementary Table S1). After filtering low-quality raw sequences, we generated approximately 49.9 million male and 55.8 million female clean reads, respectively (see Supplementary Table S2). Subsequently, both male and female clean reads were assembled together to produce 38,487 unigenes, with an N50 of 2050 bp, an average length of 1359 bp and a maximum length of 24,019 bp (Fig. 1A). The raw reads have been deposited in the National Center for Biotechnology Information (NCBI) – Sequence Read Archive (SRA) database with the accession number SRP166379.

### Functional annotation of *S. insularis* unigenes

Among the 38,487 unigenes, 17,984 (46.73%) matched loci in the NCBI non-redundant protein (Nr) database by a BLASTX homology search with a cut-off E-value of 10^−5^. The best matches were obtained for *Danaus plexippus* sequences (27.21%), followed by *Bombyx mori* (23.85%), *Papilio xuthus* (2.25%), *Acyrthosiphon pisum* (1.83%) and *Tribolium castaneum* sequences (1.64%), as shown in Fig. 1B.

Gene Ontology (GO) annotation was used to classify the unigenes into three functional groups (molecular function, cellular component, and biological process) according to the GO categories. Of 38,487 unigenes of *S. insularis*, 9023 (23.44%) were annotated. As shown in Fig. 1C, 10,973 unigenes were assigned to the molecular function category, and “binding” and “catalytic activity” were the most highly represented terms with this category. A total of 14,108 unigenes were assigned to GO terms in the cellular component category, and “cell part” and “cell” were the most abundant terms. Furthermore, 23,096 unigenes were assigned to GO terms in the biological process category, and the main terms were “metabolic process” and “cellular process”.

The KEGG Orthology (KO) system was used to classify the unigenes into five branches of Kyoto Encyclopedia of Genes and Genomes (KEGG) metabolic pathways, including processes, environmental information processing, genetic information processing, metabolism, and organismal systems. Most unigenes were assigned to the processes branch, and “global and overview maps” was the most highly represented term (Fig. 1D).

### Identification of putative OBPs

**Figure 2:**
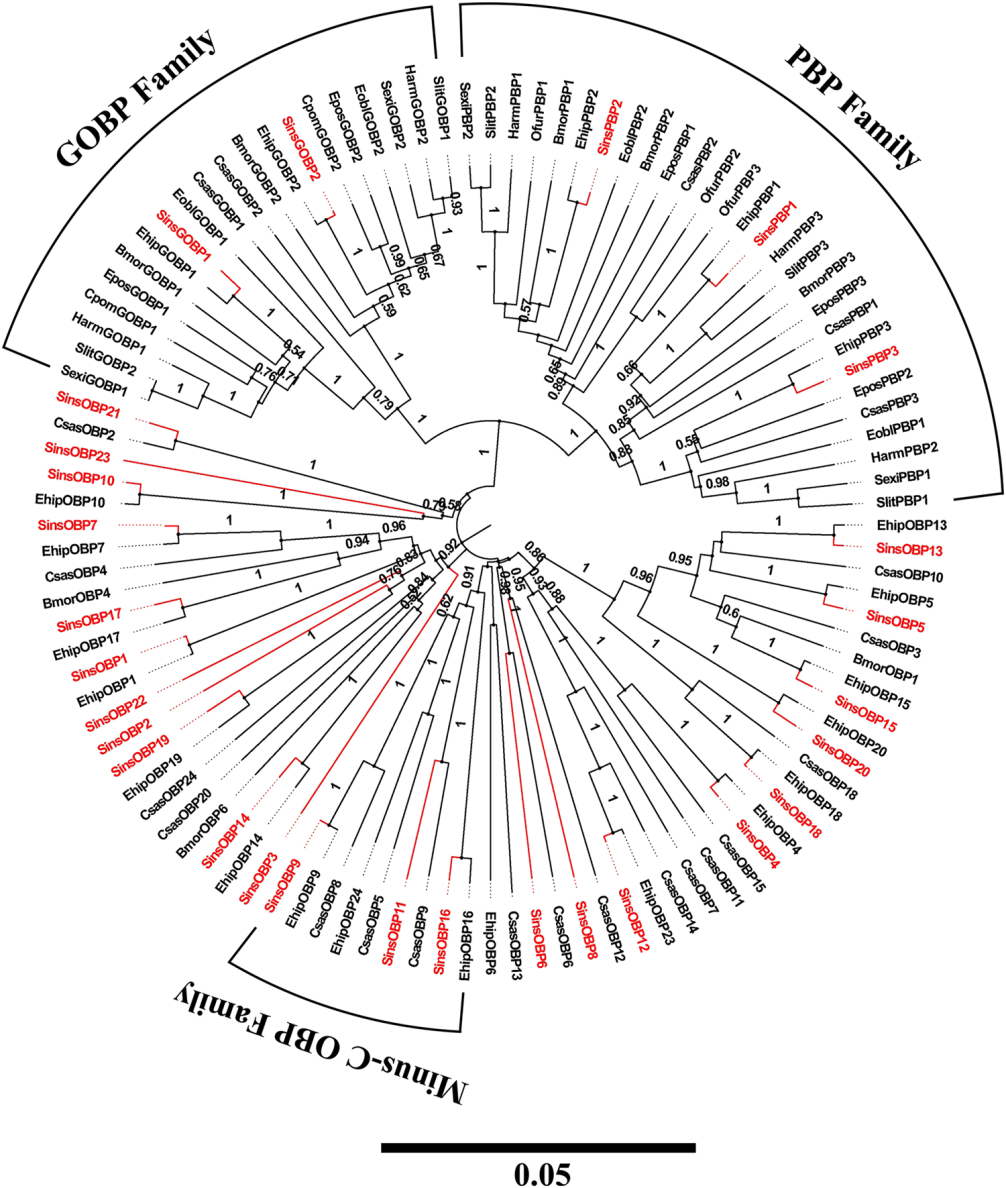
Neighbor-joining phylogenetic tree of putative OBPs from *S. insularis*, *E. hippophaecolus*, *B. mori*, *Carposina sasakii*, *Epiphyas postvittana*, *Spodoptera litura*, *Grapholita molesta*, *Helicoverpa armigera*, *Ostrinia furnacalis*, *Spodoptera exigua*, *Ectropis obliqua* and *Cydia pomonella*. The protein sequences of OBPs used to build phylogenetic trees are listed in Supplementary Table S10. The stability of the nodes was assessed by a bootstrap analysis with 1,000 replications, and only bootstrap values of ≥0.5 are shown at the corresponding nodes. The scale bar represents 0.05 substitutions per site. *S. insularis* sequences are shown in red.

We identified 28 putative OBPs in *S. insularis*, including two GOBPs and three PBPs. Among 28 OBPs, 17 OBPs (SinsOBP1, 3–4, 6, 9–12, 16–19, 21–23, PBP3 and GOBP2) were full-length genes with intact open reading frames (ORFs) of at least 400 bp and a signal peptide (see Supplementary Tables S3 and S4). The BLASTX results indicated that all OBPs of *S. insularis* shared relatively higher amino acid identities with other Lepidoptera OBPs in the NCBI Nr database (>50%). Three OBPs (SinsOBP9, 11 and 16) belonged to the minus–C OBPs based on the lack of the second and fifth cysteines, and the remaining 25 OBPs were identified as classical OBPs with the motif “C1-X_15–39_-C2-X_3_-C3-X_21–44_-C4-X_7–12_-C5-X_8_-C6” (where X represents any amino acid). A neighbor–joining phylogenetic tree was constructed using OBPs of Lepidoptera species (Fig. 2). Most OBPs of *S. insularis* had high homology with those of *Eogystia hippophaecolus*. SinsGOBP1–2 were clustered with the GOBP family, whereas SinsPBP1–3 formed part of a PBP family clade. Lepidopteran GOBPs and PBPs were highly conserved base on their different functions. Based on reads per kilobase of exon model per million mapped reads (FPKM) values, 13 OBPs (SinsOBP4–6, 9, 14–15, 18, 23, PBP1–3 and GOBP1–2) were highly abundant in male and female antennae of *S. insularis* (FPKM value > 1,000).

### Identification of putative CSPs

**Figure 3:**
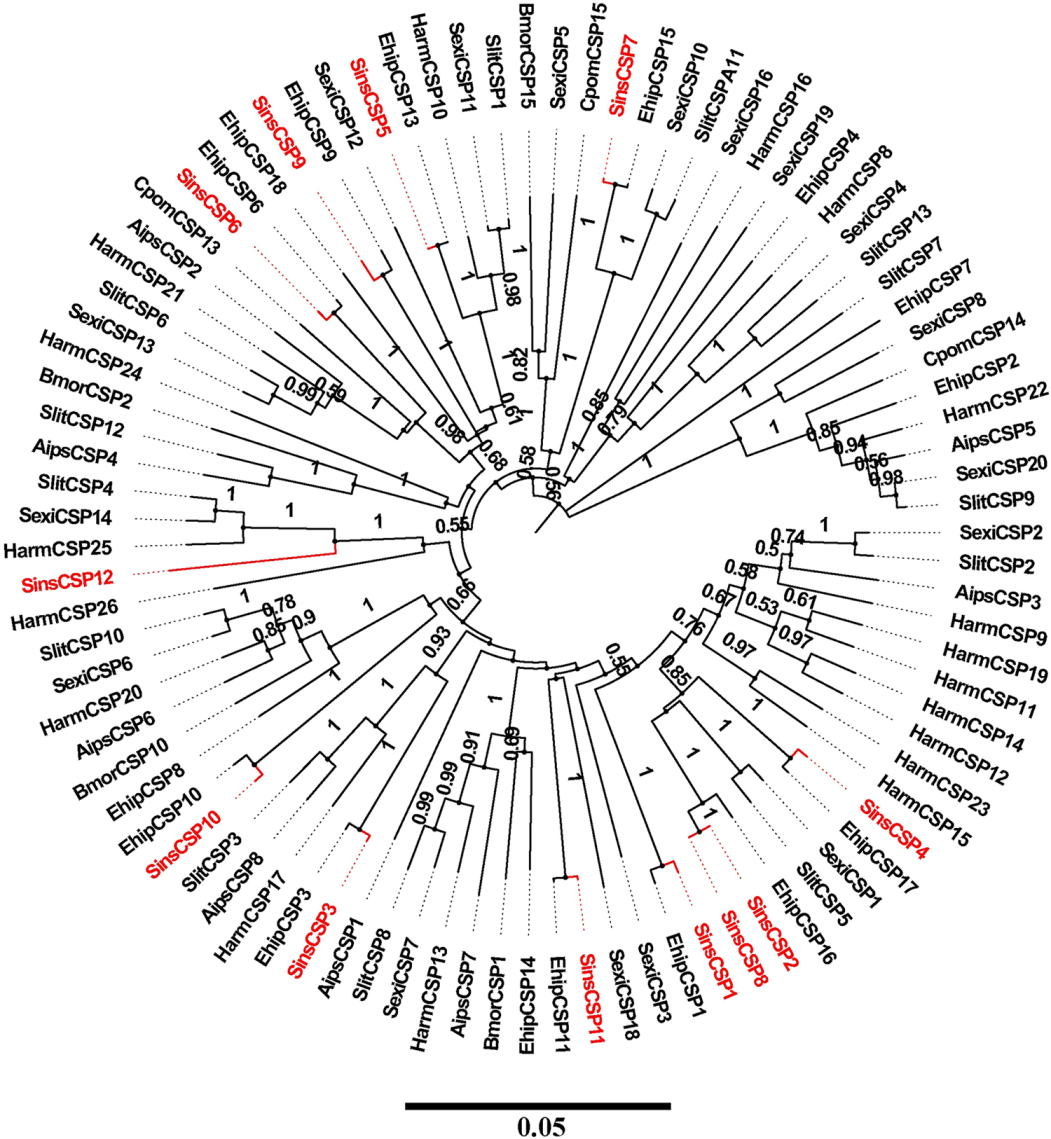
Neighbor-joining phylogenetic tree of putative CSPs from *S. insularis*, *E. hippophaecolus*, *B. mori*, *S. litura, H. armigera*, *S. exigua*, *C. pomonella* and *Agrotis ipsilon*. The protein sequences of CSPs used to build phylogenetic trees are listed in Supplementary Table S10. The stability of the nodes was assessed by a bootstrap analysis with 1,000 replications, and only bootstrap values of ≥0.5 are shown at the corresponding nodes. The scale bar represents 0.05 substitutions per site. *S. insularis* sequences are shown in red.

In the antennal transcriptome of *S. insularis*, we identified 12 putative CSPs with lengths ranging from 564 bp to 2794 bp, including ten CSPs (SinssCSP2–10 and 12) with intact ORFs and signal peptides (see Supplementary Tables S3 and S5). All putative CSPs had four cysteine residues and fit the motif “C1-X_6–8_-C2-X_16–21_-C3-X_2_-C4” (X represents any amino acid). The BLASTX results showed that 12 CSPs had relatively higher amino acid identities with Lepidoptera CSPs in the Nr database (>50%). Based on FPKM values, four CSPs (SinsCSP2, 6, 8 and 10) had relatively high expression levels in the antennal transcriptome of *S. insularis* (FPKM value > 1,000). According to the neighbor-joining phylogenetic tree of CSPs (Fig. 3), we observed that CSPs are distributed in various clades throughout the cladogram. SinsCSP1, SinsCSP3–7 and SinsCSP9–11 clustered together with EhipCSPs with high bootstrap support.

### Identification of putative ORs

**Figure 4:**
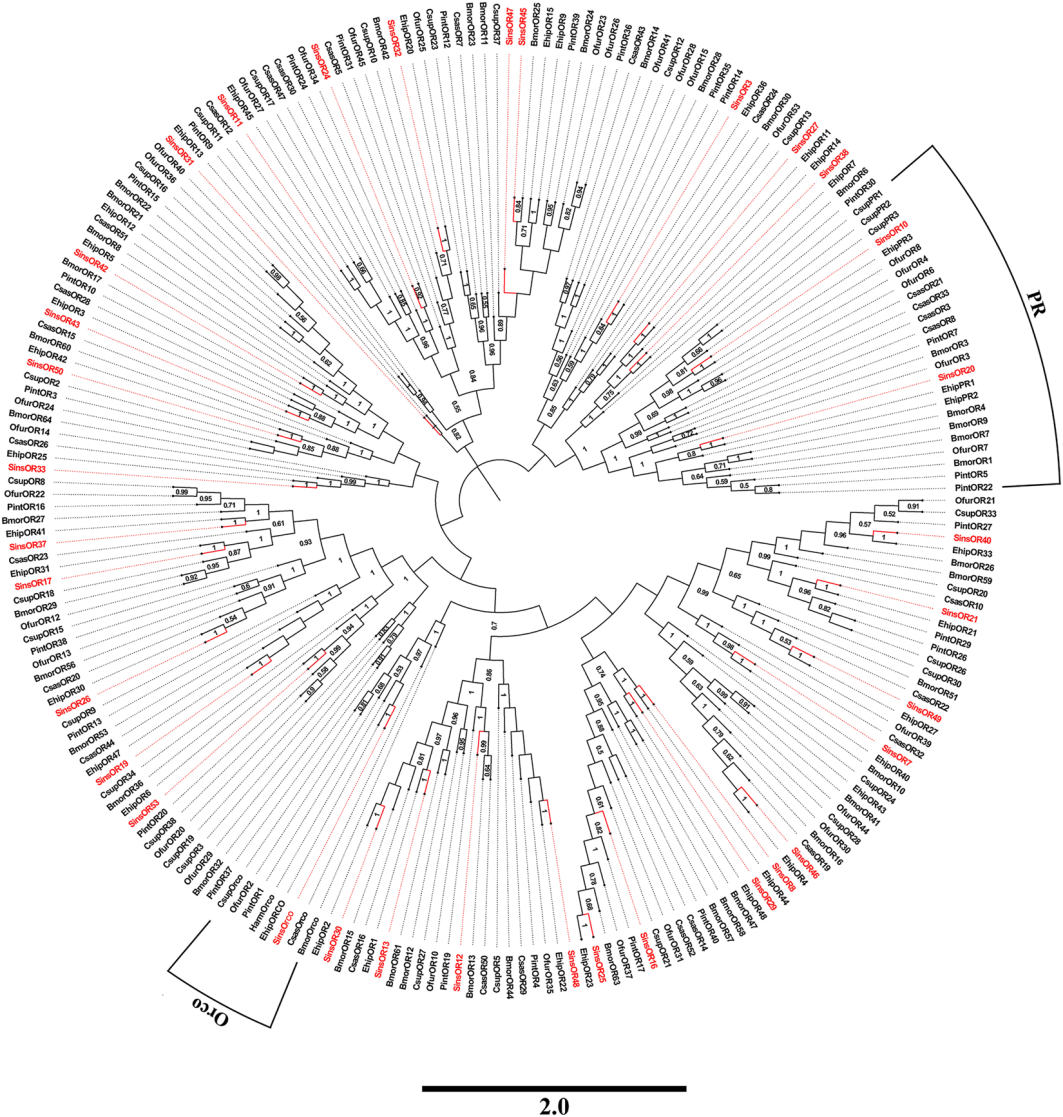
Neighbor-joining phylogenetic tree of putative ORs from *S. insularis*, *E. hippophaecolus*, *B. mori, C. sasakii*, *O. furnacalis*, *Chilo suppressalis* and *Plodia interpunctella*. The protein sequences of ORs used to build phylogenetic trees are listed in Supplementary Table S10. The stability of the nodes was assessed by bootstrap analysis with 1,000 replications, and only bootstrap values of ≥0.5 are shown at the corresponding nodes. The scale bar represents 2.0 substitutions per site. *S. insularis* sequences are shown in red.

We identified 56 putative ORs in the male and female transcriptome, 52 of which were likely full-length OR genes, encoding proteins of more than 300 amino acids with intact ORFs (see Supplementary Tables S3 and S6). The sequence identities of the best BLASTX matches in the Nr database ranged from 45% to 99%. In a FPKM analysis, SinsOrco displayed the highest expression levels in male and female antennae, with FPKM values of 472.06 and 437.42, respectively. However, the other 55 ORs showed the relatively low expression levels, with FPKM values of 0 to 260.09. The expression levels of ORs were relatively lower than those of OBPs and CSPs in *S. insularis* antennae. Of 56 ORs, 22 ORs sequences were shorter than 300 bp or had no common sites for computing distances; accordingly, we only used the 34 OR sequences of *S. insularis* to construct a phylogenetic tree. In the neighbor-joining tree of ORs (Fig. 4), two ORs (SinsOR10 and 20) were clustered into the pheromone receptor (PR) clade, and SinsOrco sequence showed high homology to the conserved insect odorant co-receptor clustered in the odorant co-receptor clade (Orco). The remaining ORs were divided to different Lepidoptera ORs ortholog clades.

### Identification of putative IRs

**Figure 5:**
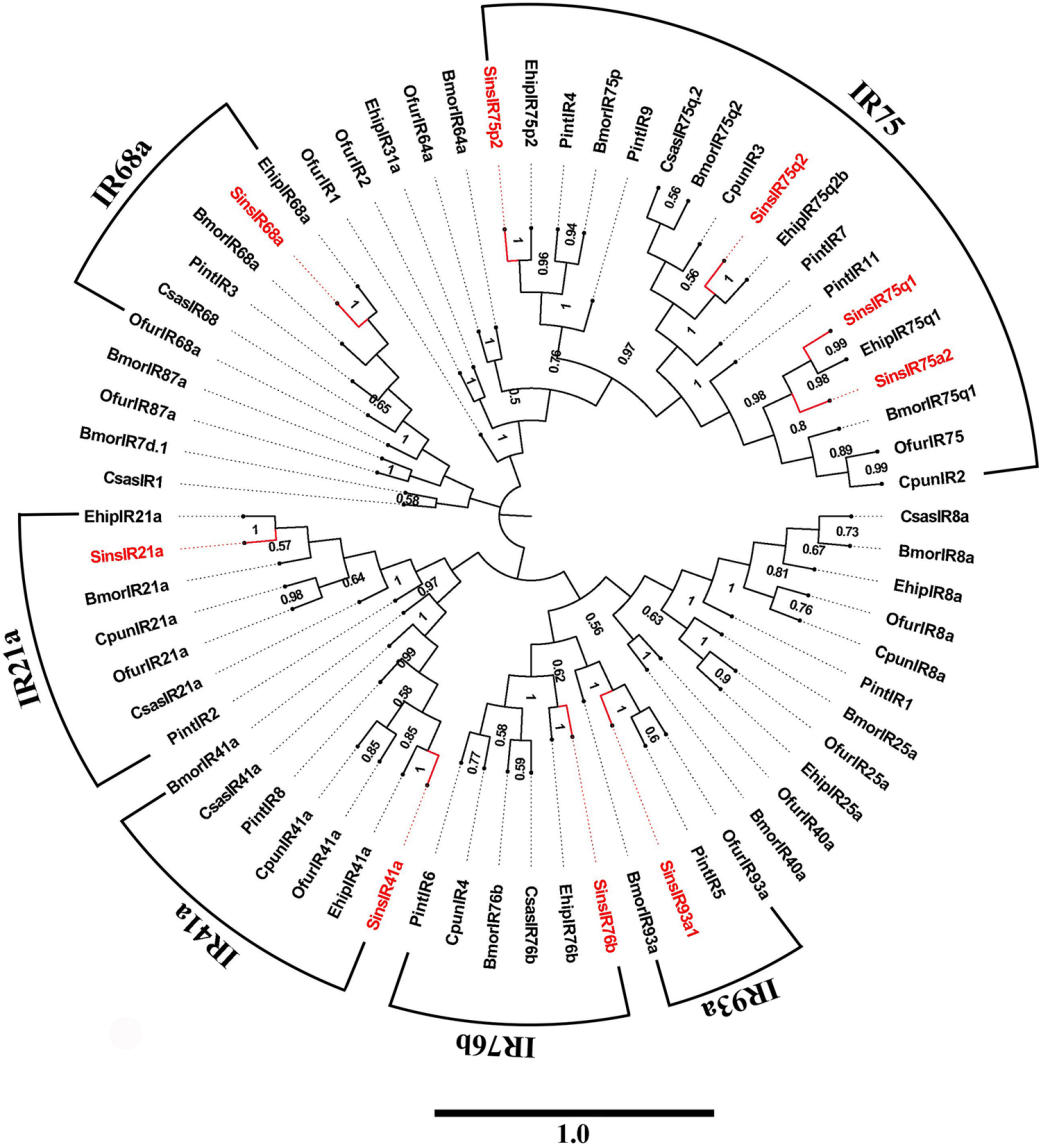
Neighbor-joining phylogenetic tree of putative IRs from *S. insularis*, *E. hippophaecolus*, *B. mori C. sasakii*, *O. furnacalis*, *C. suppressalis*, *P. interpunctella* and *Conogethes punctiferalis*. The protein sequences of IRs used to build phylogenetic trees are listed in Supplementary Table S10. The stability of the nodes was assessed by bootstrap analysis with 1,000 replications, and only bootstrap values of ≥0.5 are shown at the corresponding nodes. The scale bar represents 1.0 substitutions per site. *S. insularis* sequences are shown in red.

We identified 11 putative IRs in the antennal transcriptome of *S. insularis*, of which five sequences (SinIR93a1, 75q2, 75p2, 75a1 and 21a) were predicted to be full-length sequences with intact ORFs and signal peptides (see Supplementary Tables S3 and S7). The FPKM results showed that SinsIRs had relatively low expression levels (FPKM values ranged from 0.39 to 109.58). Among the 11 IRs, SinsIR93a2 and SinsIR75q1 sequences were shorter than 400 bp. We ued the remaining nine IRs sequences to construct a phylogenetic tree (Fig. 5). The IRs identified in the antennal transcriptome of *S. insularis* were assigned to the different clades of the conserved IRs with reliable bootstrap support, including IR21a, IR41a, IR68a, IR93a, IR76b and IR75. IR75 included four SinsIRs (SinsIR75p2, SinsIR75q2, SinsIR75q1 and SinsIR75a2).

### Identification of putative SNMPs

**Figure 6:**
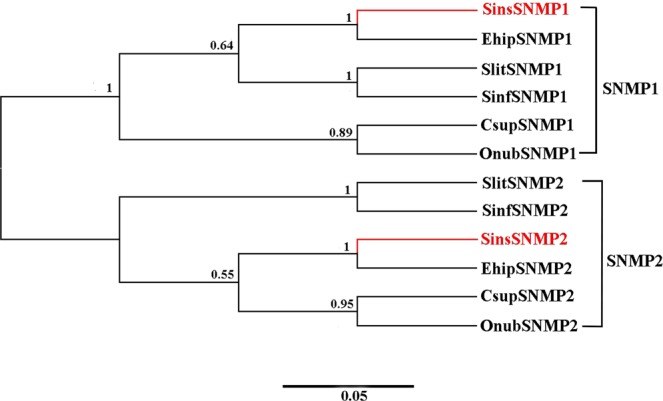
Neighbor-joining phylogenetic tree of putative SNMPs from *S. insularis*, *E. hippophaecolus*, *S. litura*, *C. suppressalis, Sesamia inferens* and *Ostrinia nubilalis*. The protein sequences of SNMPs used to build phylogenetic trees are listed in Supplementary Table S10. The stability of the nodes was assessed by a bootstrap analysis with 1,000 replications, and only bootstrap values of ≥0.5 are shown at the corresponding nodes. The scale bar represents 0.05 substitutions per site. *S. insularis* sequences are shown in red.

Two putative SNMPs with intact ORFs (1473 and 1566 bp, respectively) were identified in the combined male and female antennal transcriptome of *S. insularis* (see Supplementary Tables S3 and S8) and shared relatively high identities (over 95%) with SNMPs of *E. hippophaecolus*. The FPKM analysis showed that the expression levels of SinsSNMPs in male antennae were higher than those in female antennae. Based on a phylogenetic tree (Fig. 6), SinsSNMP1 and SinsSNMP2 clustered within the SNMP1 subfamily and SNMP2 subfamily, respectively.

### Identification of putative ODEs

We also identified 101 putative ODEs in the antennal transcriptome of *S. insularis*. ODEs were divided into five families, including 19 CEXs, ten AOXs, eight ADs, 47 CYPs and 17 GSTs (see Supplementary Tables S3 and S9). Among the 101 putative ODE genes, 12 had intact ORFs and signal peptides (SinsCEX3, 5, 7, 9–11, 16, 19, SinsAD4, SinsCYP18 and SinsGST8, 13). The BLASTX results indicated that 101 identified ODEs shared relatively higher amino acid identities ranging from 50% to 98% with Lepidoptera ODEs in the NCBI Nr protein database. An FPKM analysis showed that only three ODEs (SinsCEX3, SinsCYP20 and 36) were highly abundant in the antennal transcriptome of *S. insularis* (FPKM value > 1,000).

### Tissue-specific and sex-specific expression of putative OBPs, CSPs and SNMPs

**Figure 7:**
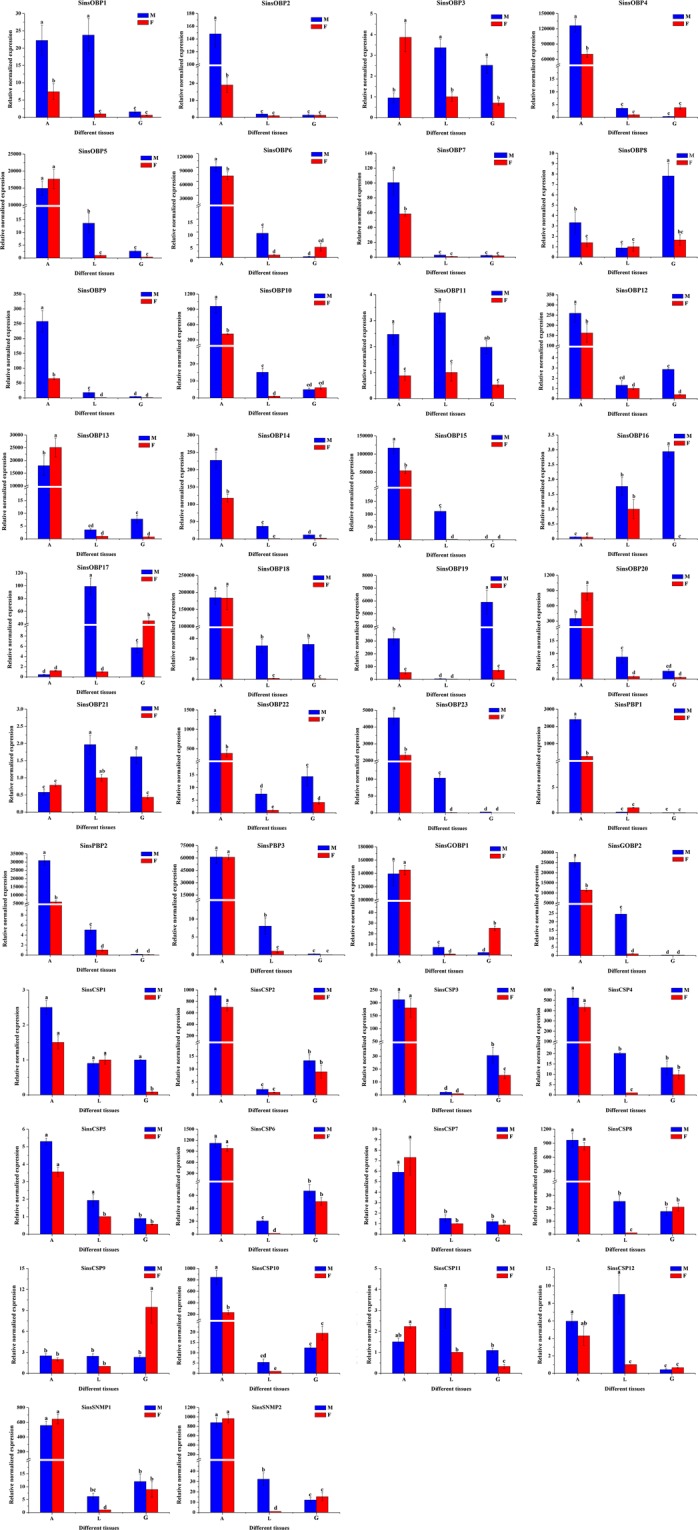
Expression profiles of the putative OBPs, CSPs and SNMPs in various *S. insularis* tissues. A: antennae; L: legs; G: genitalia (copulatory organ for male moths; ovipositor for female moths). β-actin was used as a reference gene for the normalization of target gene expression. Blue and red represents males and females, respectively. The standard errors are represented by the error bars, and different lowercase letters (a–d) above the bars denote significant differences (*p* < 0.05).

To better understand the functions of 28 putative OBPs, 12 CSPs and two SNMPs in the different tissues of male and female *S. insularis*, we used RT-qPCR to evaluate expression patterns. We found that 20 OBPs were specifically and highly expressed in both female and male antennae (Fig. 7), of which 14 OBPs (*SinsOBP2*, *4*, *6–7*, *9–10*, *12*, *14–15*, *22–23*, *PBP1–2* and *GOBP2*) were expressed at significantly higher levels in males than in females. Two OBPs (*SinsOBP13* and* 20*) were expressed at significantly higher levels in female antennae than in male antennae. The expression of four OBPs (*SinsOBP5*, *18*, *PBP3* and *GOBP1*) were not significantly different between male and female antennae. In addition, three OBPs (*SinsOBP8*, *16* and *19*) were more highly expressed in the male genitalia than in other tissues. *SinsOBP17* was highly expressed in the male legs. Finally, the remaining four OBPs (*SinsOBP1*, *3*, *11* and *21*) were expressed in various tissues of both sexes. In CSPs, six CSPs (*SinsCSP2–4*, *6*, *8*, *10*) were highly expressed in the antennae, and *SinsCSP10* expression in male antennae was highly significantly different from expression in female antennae. The other six CSPs (*SinsCSP1*, *5*,* 7*, *9*, *11–12*) were expressed in all tissues. In addition, the expression of *SinsSNMP1* and *SinsSNMP2* were highly expressed in the antennae of *S. insularis*, with no significant difference in expression level was found between the sexes.

## Discussion

The gene sets reported in this study substantially increase the pool of genes encoding olfactory protein genes in Cossidae. Prior to our study, members of the major olfactory gene families in Cossidae were identified only from the antennal transcriptome of *E. hippophaecolus* (excluding ODEs)^40^. What’s more, the previous studies in *S. insularis* had focused on biology and ecology. Recently, next-generation sequencing is widely used for the identification of olfactory proteins in insects owing to their important functional roles^40–45^. Our results will provide potential targets for the disruption of the olfactory system in *S. insularis* for pest control purposes.

We identified 38,487 unigenes with a mean length of1359 bp from the male and female *S. insularis* antennal transcriptome, indicating the high quality and great depth of sequencing at the transcriptome level. BLASTX homology search in the NCBI Nr protein database found that *S. insularis* unigenes shared the relatively high homology with sequences from other Lepidoptera species, further supporting the accuracy of our transcriptome data. Additionally, we identified large numbers of transcripts encoding putative olfactory proteins, including 28 OBPs, 12 CSPs, 56 ORs, 11 IRs, two SNMPs and 101 ODEs. These olfactory protein counts in *S. insularis* were similar to these in *E. hippophaecolus* (29 OBPs, 18 CSPs, 63 ORs, 12 IRs, and two SNMPs)^40^, *Carposina sasakii* (29 OBPs, 13 CSPs, 52 ORs, eight IRs, and one SNMPs)^45^, *Plutella xyllostella* (24 OBPs, 15 CSPs, 54 ORs, 16 IRs, and two SNMPs)^46^ and other Lepidoptera insects. By contrast, previous studies have detected fewer ODEs; Zhang *et al*. (2017) identified 18 putative CXEs and four AOXs genes from *Cnaphalocrocis medinalis* antennal transcriptome^27^, Liu *et al*. (2015) reported 19 putative CXEs and 16 GSTs in *Chilo suppressalis* antennae^47^, and Leitch *et al*. (2015) identified 97 candidate CYPs and 39 CEXs in the antennal transcriptome of *Calliphora stygia*^28^.

Hydrophobic odours are thought to interact with OBPs and CSPs prior to the ligand–receptor interaction. An OBP has a mass of approximately 15–17 kDa^48^. We detected fewer OBPs in the *S. insularis* antennal transcriptome than previously reported in *Plodia interpunctella*^49^, *Mythimna separate*^50^, and *Bombyx mori*^51^, but more than in *Sesamia inferens*^52^. In the phylogenetic tree of OBPs, we observed the differentiation of Lepidopteran OBPs into several branches over a long evolutionary timescale, in accordance with previous results^40–46^. GOBPs and PBPs subfamilies formed separate clusters, suggesting that they diverged from a common ancestral gene due to speciation and reproductive isolation^49^. Furthermore, we observed distinct tissue-biased of OBPs in insects, strongly indicative of biological functions^53^. In general, an antenna-enriched expression profile is correlated with a role in olfactory perception, whereas genes that are highly expressed in gustatory organs, such as the proboscis, leg and ovipositor, could be involved in taste detection^54–58^. Remarkably, 20 OBPs (*SinsOBP2*, *4–7*, *9–10*, *12–15*, 18, 20, *22–23*, Sins*PBP1–3* and Sins*GOBP1–2*) were highly expressed in the antennae and may have vital roles in the detection of odorants, such as sex pheromones and host plant volatiles. These results were consistent with the expression patterns observed in other insects belonging to Lepidoptera, such as *E. hippophaecolus*^40^, *Helicoverpa assulta*^59^, and *Agrotis ipsilon*^60^. *SinsOBP17* was highly expressed in the legs and may therefore be involved in the recognition of these contact substances on host plant surfaces^61^. In addition, we observed three OBPs (*SinsOBP8*, 16 and 19) that were highly expressed in male genitalia; these might contribute to the controlled release of semiochemicals into the environment^62^. In comparison, the molecular weights of CSPs (10–15 kDa) were lower than those of OBPs^7^. The number of CSPs reported in our antennal transcriptome was in accordance with the number detected for *A. ipsilon*^60^, but less than those in the *P. interpunctella*^49^ and *M. separate*^50^. SinsCSPs were distributed among various clades in the CSP phylogeny, indicating that these genes may have various functions in chemical communication^12–14^. In our RT-qPCR analysis, CSPs were expressed in both olfactory organ and non-olfactory organ, suggesting that they have broad functions in *S. insularis*, including roles in non-olfactory functions. We identified two SNMPs (SinsSNMP1 and SinsSNMP2) assigned to the SNMP1 subfamily and SNMP2 subfamily in the phylogenetic tree; the high divergence suggests that these SNMPs have different function *in vivo*. *SinsSNMP1* and *SinsSNMP2* were specifically expressed in antennae. Combined with previous findings, SNMPs may be indispensable for the identification of sex pheromones^16–19,63^.

Moth olfactory receptors belong to two families: ORs and IRs. ORs are seven transmembrane domain proteins with an inverted topology compared with that of vertebrates ORs and play a crucial role in chemosensory reception^20,64,65^. We identified 56 ORs were identified in the *S. insularis* antennal transcriptome, similar to the numbers identified in other moths (63 in *E. hippophaecolus* and 58 in *Cydia pomonella*)^40,66^. In a phylogenetic tree of ORs, SinsOR10 and SinsOR20, and other Lepidopteran PRs, were assigned to the same clade in the tree, particularly those encoding PR orthologues in *E. hippophaecolus*. SinsOR10 and SinsOR20 could be the PRs in *S. insularis*. Additionally, SinsOrco was clustered into the Orco clade, with Orco orthologues also in *E. hippophaecolus*. Accordingly, SinsOrco is most likely the Orco. In addition, IRs are a conserved family of synaptic ligand-gated ion channels, but the specificity of ligand recognition by IRs is still unclear^23,24,67^. We identified 11 IRs in *S. insularis*, similar to the numbers in other Lepidoptera species, such as *E. hippophaecolus*^40^, *Athetis dissimilis*^68^ and *P. interpunctella*^49^. A phylogenetic analysis suggested that SinsIR93a1, SinsIR76b, SinsIR41a, SinsIR21a, and SinsIR68a are the IR93a, IR76b, IR41a, IR21a, IR68a, respectively, and SinsIR75p2, SinsIR75q2, SinsIR75q1 and SinsIR75a2 are the IR75 genes of *S. insularis*. IR8a and IR25a are were predicted to be the co-receptors present in the IR group and are conserved in many insects^23,25^; however, we did not find them in the *S. insularis* antennal transcriptome, which may be explained by a lower expression level in *S. insularis*, this indicated a further study was needed to identify.

In insects, the rapid degradation of odorant molecules in antennae is important for the sensitivity of olfactory receptor neurons. Odorant degradation in antennae is mediated by various enzymes. We identified 19 CEXs, ten AOXs, eight ADs, 47 CYPs and 17 GSTs in *S. insularis*. Of these, many antennae-specific CXEs have been identified in various insects, uch as *Spodoptera exigua*, *S. littoralis*, *S. litura* and *D. melanogaster*, and are involved in olfactory signal (host plant volatiles and sex pheromones) termination^69–71^. AOXs are also found in insect antennae and are thought to degrade aldehyde odorant compounds in *B. mori* and *Mamestra brassicae*^72,73^. Recently, ADs and CYPs were cloned from insect antennae and may play roles in xenobiotic degradation, detoxification and biotransformation of endobiotic compounds^74,75^. GSTs are also related to odorant degradation. For example, BmGSTD4 is specifically expressed in male *B. mori* antennae, suggesting it is involved in sex pheromone degradation^76^. It is possible that many transcripts of putative enzymes are ODEs of *S. insularis*, but further biochemical analyses of their specific physiological roles are necessary.

## Conclusions

*S. insularis* is regarded as a destructive wood-boring pest affecting various street trees in China. However, the olfactory system of this moth has not been deciphered so far. In this study, we first assembled the antennal transcriptome of male and female *S. insularis* and annotated a set of olfactory genes, including 28 OBPs, 12 CSPs, 56 ORs, 11 IRs, two SNMPs, and 101 ODEs. Then, Sequence and phylogenetic analyses confirmed the characteristics of these proteins. Additionally, using RT-qPCR, we observed both tissue- and sex-specific expression of OBPs, CSPs and SNMPs, indicating that these loci play crucial roles in courtship, mating and oviposition behaviour. Further studies are needed to determine the function of putative PBPs and PRs of *S. insularis*, to reveal their specificity and binding to sex pheromones. These analyses could provide novel targets for the disruption of chemical communication in *S. insularis* for pest control purposes.

## Methods

### Ethics statement

*S. insularis* is not on the “List of Endangered and Protected Animals in China”. The Beijing Municipal Bureau of Landscape and Forestry issued a permit for the field collection.

### Insect and tissue collection

*S. insularis* individuals were collected from *Fraxinus americana* on Beijing Forestry University North Road, Haidian District, Beijing, China (40°0′N, 116°20′E), in May 2017. Damaged trunks were cut off and taken to the laboratory. Larvae inside the trunks were fed on the phloem and xylem of the host under natural environmental conditions. After their eclosion, the moths were sorted by sex according to the genitalia. 150 antennae, 60 legs, and 60 genitalia (copulatory organ for male moths; ovipositor for female moths) were excised from the two sexes, immediately placed in RNAlater (Ambion, Austin, TX, USA), and then stored at −80 °C.

### RNA extraction

Total RNA was extracted from 25 antennae of each sex using TRIzol reagent (Invitrogen, Carlsbad, CA, USA) following the manufacturer’s instructions, with three replicates per sex. RNA purity was evaluated using the NanoDrop 2000 (Thermo, Waltham, MA, USA), RNA concentration was measured using the QubitRNA Assay Kit with a Qubit2.0 Fluorometer (Life Technologies, Carlsbad, CA, USA), RNA integrity was detected using the Agilent Bioanalyzer 2100 system (Agilent Technologies, Santa Clara, CA, USA), and RNA degradation and contamination were monitored on a 1% agarose gel to ensure the quality of the RNA samples for subsequent transcriptome sequencing.

### cDNA library construction and Illumina sequencing

cDNA library construction and Illumina sequencing were performed at Shanghai Majorbio Bio-pharm Technology Co., Ltd. (Shanghai, China). According to the TruSeq RNA Sample Preparation Guide V2 (Illumina), mRNA was purified from total RNA using oligo (dT) magnetic beads and then fragmented by the addition of fragmentation buffer. Random hexamer primers were used to synthesize the first-strand cDNA, followed by the synthesis of the second-strand cDNA using dNTPs, RNase H and DNA polymerase I. Remaining overhangs were passivated via polymerase activity. After the end-repairing, Poly-A tailing and the ligation of adapters, cDNA fragments of 150–200 bp were purified using the AMPure XP system (Beckman Coulter, Beverly, MA, USA). Then, 3 μl USER Enzyme (NEB, Ipswich, MA, USA) was used with size-selected, adaptor-ligated cDNA at 37 °C for 15 min, followed by 5 min at 95 °C prior to PCR amplification. PCR products were purified using the AMPure XP system and library quality was assessed on the Agilent Bioanalyzer 2100 system. The cDNA libraries of *S. insularis* were sequenced on the Illumina Hiseq^TM^ 4,000 platform, and paired-end reads were generated.

### Sequence assembly and functional annotation

To obtain the clean reads, the raw reads were processed to remove low-quality reads and adapter sequences. Then, GC Content, Q20 and Q30 were used to assess the sequencing quality. Clean reads were assembled de novo with Trinity^77^ after combined the male and female *S. insularis* clean reads. The largest alternative splicing variants in the Trinity results were called unigenes. BLASTX searches were used to align unigenes and compare them with the NCBI Nr database, using a cut-off E-value of 10^−5^. Then, Nr BLASTX results were subjected to GO annotation using Blast2GO^78^. Pathway annotations for unigenes were determined using KO^79^. ORF of each unigenes were then predicted using ORF finder (http://www.ncbi.nlm.nih.gov/gorf/gorf.html). FPKM values calculated by RSEM (RNA-Seq by Expectation-Maximization) with default parameters represented gene expression levels in the male and female *S. insularis* antennae^80^.

### Identification of olfactory genes and phylogenetic analyses

Using TBLASTN, the sequences of OBP, CSP, OR, IR, SNMP and ODE from insecta species as queries to identify putative unigenes encoding putative OBPs, CSPs, ORs, IRs, SNMPs and ODEs in *S. insularis*. All putative OBPs, CSPs, ORs, IRs, SNMPs and ODEs were manually checked by the BLASTX program in NCBI online. The N-terminal signal peptides of putative OBPs, CSPs, ORs, IRs, SNMPs, and ODEs were found using SignalP4.0 (http://www.cbs.dtu.dk/services/SignalP/). All putative OBP, CSP, OR, IR, SNMP and ODE amino acid sequences of *S. insularis* and other insect species were aligned using ClustalW implemented in MEGA (version 5.0). Phylogenetic trees were generated by the neighbor-joining method as implemented in MEGA (version 5.0), with the p-distance model and pairwise deletion of gaps. Bootstrap support for tree branches was assessed by re-sampling amino acid positions 1000 times^81^. Phylogenetic trees were color-coded and arranged using FigTree (Version 1.4.2). The amino acid sequences of olfactory proteins used to build phylogenetic trees are listed in Supplementary Table S10.

### Expression patterns of putative OBPs by RT-qPCR

Expression patterns of putative OBPs in various tissues (antennae, legs and genitals) of the two sexes were analysed by RT-qPCR using the Bio-Rad CFX96 PCR System (Hercules, CA, USA). Total RNA was extracted from 25 antennae, ten legs and ten genitals from each sex following the method described above, and was transcribed into cDNA using the PrimeScriptRT Reagent Kit with gDNA Eraser (No. RR047A; TaKaRa, Shiga, Japan). Gene-specific primers were designed using Primer 3 Plus (http://www.bioinformatics.nl/cgi-bin/primer3plus/primer3plus.cgi) (see Supplementary Table S11). β-actin from *S. insularis* was used as a reference gene. The RT-qPCR mixtures were composed of 12.5 µl of TB Green™ Premix Ex Taq™ II (Tli RNaseH Plus) (No. RR820A; TaKaRa), 1 µl of forward primer (10 µM), 1 µl of reverse primer (10 µM), 2 µl of cDNA and 8.5 µl of sterilized H_2_O. RT-qPCR cycling parameters were as follows: 95 °C for 30 s, followed by 40 cycles of 95 °C for 5 s and 60 °C for 30 s, and 65 °C–95 °C in increments of 0.5 °C for 5 s to generate the melting curves. To check reproducibility, each reaction for each tissue was performed in three biological replicates and three technical replicates. Negative controls without the template were included in each experiment. The relative expression levels were calculated according to the comparative 2^−ΔΔC^_T_ method (the amplification efficiency for 42 genes was close to 100%)^82^, and the female leg sample was used as the calibrator, β-actin was used for calculating and normalizing the target gene expression and correcting for sample to sample variation. Data (means ± SE) from different samples were subjected to one-way nested analysis of variance, followed by Tukey’s honestly significance difference tests implemented in SPSS Statistics 22.0 (IBM, Chicago, IL, USA).

## Supplementary information

Supplementary Table S1

Supplementary Table S2

Supplementary Table S3

Supplementary Table S4

Supplementary Table S5

Supplementary Table S6

Supplementary Table S7

Supplementary Table S8

Supplementary Table S9

Supplementary Table S10

Supplementary Table S11
