# Supplementary material for: Antennal transcriptome analyses and olfactory protein identification in an important wood-boring moth pest, Streltzoviella insularis (Lepidoptera: Cossidae)
Source: Sci Rep. 2019 Nov 29;9:17951. doi: 10.1038/s41598-019-54455-w (PMC6884542; doi:10.1038/s41598-019-54455-w)
Supplement: Supplementary file 1 — Supplementary Table S1 [file 41598_2019_54455_MOESM1_ESM.docx]

**Supplementary Information for**

**Antennal transcriptome analyses and olfactory protein identification in an important wood-boring moth pest, *Streltzoviella insularis* (Lepidoptera: Cossidae)**

**Yuchao Yang^1^, Wenbo Li^1^, Jing Tao^1^*, Shixiang Zong^1^***

^1^Beijing Key Laboratory for Forest Pest Control, Beijing Forestry University, Beijing 100083, China

* Corresponding authors

**Email addresses:**

Yuchao Yang: yangyc68@126.com

Wenbo Li: leonardolee24@hotmail.com

Jing Tao: taojing1029@hotmail.com

Shixiang Zong: zongsx@126.com

**Table S1.** Summary of raw data for *S. insularis.*

| **Samples** | **Raw data** | | | | |
| --- | --- | --- | --- | --- | --- |
|  | **Read number** | **Base number** | **GC content** | **%≥Q20** | **%≥Q30** |
| ♂Antennae 1 | 48,529,372 | 7,327,935,172 | 43.36% | 96.99% | 92.98% |
| ♂Antennae 2 | 56,809,292 | 8,578,203,092 | 43.89% | 97.25% | 93.42% |
| ♂Antennae 3 | 52,497,784 | 7,927,165,384 | 43.67% | 96.89% | 92.75% |
| ♀Antennae 1 | 52,783,690 | 7,970,337,190 | 43.82% | 96.93% | 92.80% |
| ♀Antennae 2 | 65,259,648 | 9,854,206,848 | 43.43% | 97.14% | 93.20% |
| ♀Antennae 3 | 58,327,992 | 8,807,526,792 | 44.40% | 97.16% | 93.24% |

♂Antennae 1-3: Three biological replicate groups of male antennae.

♀Antennae 1-3: Three biological replicate groups of female antennae.
