# Supplementary material for: Antennal transcriptome analyses and olfactory protein identification in an important wood-boring moth pest, Streltzoviella insularis (Lepidoptera: Cossidae)
Source: Sci Rep. 2019 Nov 29;9:17951. doi: 10.1038/s41598-019-54455-w (PMC6884542; doi:10.1038/s41598-019-54455-w)
Supplement: Supplementary file 2 — Supplementary Table S2 [file 41598_2019_54455_MOESM2_ESM.docx]

**Supplementary Information for**

**Antennal transcriptome analyses and olfactory protein identification in an important wood-boring moth pest, *Streltzoviella insularis* (Lepidoptera: Cossidae)**

**Yuchao Yang^1^, Wenbo Li^1^, Jing Tao^1^*, Shixiang Zong^1^***

^1^Beijing Key Laboratory for Forest Pest Control, Beijing Forestry University, Beijing 100083, China

* Corresponding authors

**Email addresses:**

Yuchao Yang: yangyc68@126.com

Wenbo Li: leonardolee24@hotmail.com

Jing Tao: taojing1029@hotmail.com

Shixiang Zong: zongsx@126.com

**Table S2.** Summary of clean data for *S. insularis.*

| **Samples** | **Clean data** | | | | |
| --- | --- | --- | --- | --- | --- |
|  | **Read number** | **Base number** | **GC content** | **%≥Q20** | **%≥Q30** |
| ♂Antennae 1 | 46,012,288 | 6,821,566,178 | 43.18% | 98.26% | 94.99% |
| ♂Antennae 2 | 54,043,554 | 8,025,278,424 | 43.74% | 98.35% | 95.17% |
| ♂Antennae 3 | 49,726,740 | 7,366,455,790 | 43.50% | 98.20% | 94.83% |
| ♀Antennae 1 | 50,014,330 | 7,419,580,133 | 43.65% | 98.18% | 94.79% |
| ♀Antennae 2 | 62,077,706 | 9,209,394,060 | 43.27% | 98.27% | 95% |
| ♀Antennae 3 | 55,449,354 | 8,225,116,516 | 44.25% | 98.30% | 95.05% |

♂Antennae 1-3: Three biological replicate groups of male antennae.

♀Antennae 1-3: Three biological replicate groups of female antennae.
