# Supplementary material for: Antennal transcriptome analyses and olfactory protein identification in an important wood-boring moth pest, Streltzoviella insularis (Lepidoptera: Cossidae)
Source: Sci Rep. 2019 Nov 29;9:17951. doi: 10.1038/s41598-019-54455-w (PMC6884542; doi:10.1038/s41598-019-54455-w)
Supplement: Supplementary file 3 — Supplementary Table S3 [file 41598_2019_54455_MOESM3_ESM.docx]

**Supplementary Information for**

**Antennal transcriptome analyses and olfactory protein identification in an important wood-boring moth pest, *Streltzoviella insularis* (Lepidoptera: Cossidae)**

**Yuchao Yang^1^, Wenbo Li^1^, Jing Tao^1^*, Shixiang Zong^1^***

^1^Beijing Key Laboratory for Forest Pest Control, Beijing Forestry University, Beijing 100083, China

* Corresponding authors

**Email addresses:**

Yuchao Yang: yangyc68@126.com

Wenbo Li: leonardolee24@hotmail.com

Jing Tao: taojing1029@hotmail.com

Shixiang Zong: zongsx@126.com

**Table S3.** Nucleic acid sequences of all putative olfactory genes identified in the *S. insularis* transcriptome.

**OBPs:**

>SinsOBP1

TTTTTTTTTATTAAACGAATAACACTATTTTATTGGTTATATTTATTGCGAGTACAAGAGTTCAAATATCTTCTTCCCGAATACTTTTTCAATATACGTAGCCGAAATGTTGCTTCTGGTCCAAGAAGCAGTTGTAGATCAATTTGGCACGATCGCAGCCCTTCGGTCCGTCGCTCACGCTTTGCTCGTTTACTGATACACATGCTTTGACTGCTTCATTTGCCTTCTCCAATTCACCCTCCTCAGCATAATATTGGCTTCTTTCAGCTGCTTTAGCTTCGACGAATAAGCCTTTGTCATCCAAAAGCCCGGTCTTCTGGAAGACACACGCAAAGAAACATGCGTTAGATTCATCAGGACGCTTTTGTTGTTGTCTGAATTCCTTCAAATAATCCAATGTGATGCTATGCTCCTTGCCGCATTCAGTTAAAATAGGGACTAACTTGGCTTTAAGCTCCGTTTTCTTCTCTTCGGTTATTGCTTCAGTACTTTGCATTGTTGCCGCAAAGATAGCGACAAACATAGCAATGAAAATTTTTACGGCCATTTTTTTTAAATAATATGTTTTGCTAAATATCCTAATTGTACAGAGCCTTAATGATTTCTGAACACAAACCAGACATTCAACCACTATATATACTCCTTCAAATGTGTCGGATTAGTCATTTTAAATAAATTATTTTTTGTTGATGACTTCGCGCTTGAATCCG

>SinsOBP2

ATGCTACTAAGCGATATACATATTCATGTTAAAAAGAAAAGAAAAAAAAATCTAAGCAAGTCGTCTAATCAATTATAAGCGTAAAATGAATCGAAGGCCTTCATAGTGTCATTCAGGATATTGGGATCCGCCATAGATGCTTTGTTTAGTATAAAACCAACTGAAATTTTGTTACAAACATCACATTCATCTTAGCAACTTGATTGATTAGATCGATTATAAACAACATAATACGTGTAATCAAGATGTTCAAGTCAGTTTTAGTCTCATTTGCTTTATTTGTCTTAATTTTAGGAAACGCGAAGGCCATCAGTGATGAGTACAGGACCCAAATACAATCCAAAATCGTAGAAGTGGGCGCTGATTGTTTCAAAGAGCATCCGTTGGGCCTGGATGACATAGCCGCTTTCAAAATCAGAAAGTTCCCTGACGGAGAAAATGCCGGTTGTTTTACTTCTTGCATTTTCAAAAAAATTGGAATTTTCGATGAAACAGGCATCTTATCTCGTGAGACCGCTATAGAAAATGTCAAAGAAATCATTGAAGATGAAGAGGAGCTACAGAACGTACGCGATTTCTTCGCCGCTTGCTCTTCAGTAAATGACGAAGATGTGACTGACGGTGAATTAGGCTGTGACCGCGCCAAACTTGCATTCAATTGCTTTGTCGAGAATTCCGAAAAGTTTGGACTCAACCTTGACTTTTA

>SinsOBP3

AGTTATGATATATTTATATATTTAATTTTAAGATATGAATCTACATAATAAATCAACGTTGTCTAAACCGGGTAAAAGTCCTGTAATAATTCTTCCATGTAAATATTCTTTGCTGACACCAGTACACGCCAGTTGGAGTAGTGCCCTGAATACCCCGCCGATGATTTCTCGTAAATCTCGTTTTGTTCTAACCGGACTGGTAGAATTTACATTTTCGCCATCTAATATTGTAGGTCTTTCACTGGGACTTTCCATTTTTGTCTCCTCCATTATGCACGACAGCACCAGCTTGGCTCTATCACAACCCTTGACTCCGTCTGATACTTCAAGATCGTTGACATAAGAACAATCTAGAAACACTGAGACAACAGCATTAAATTCATCCATATCCGTAAATAACATTTTTGCAACCTCAGCCACTGCCTCTGGAGAAACCTTACCTGTAGAATCTATCATATTGGCTTTTTTTAATATACAAGCAAAGAAACAAGGCGCATCTTTTCCTTTGGGCATATTAAAATTTTGTAAAGCGCGGATATCTTCAAAAGATACATTGTGTTTGGCCAAACATCCTACACCAAAAGTCACAAAATGATGACTTAACTTTCTCTTATGATTGTCTGATAATGATGGATCACAATAAACACAGACAGAGAATTTCATTAGCAATATTGTAACGAATGTAATTTGAATATTCATTTTCTTCACGTGCGATCTTGTTTCGTACTAAAGAATTACTAGTCTGCCTTTGTATAAACAATTATACAAACTGATTTTTTGTATAGTCATTGAGATGACAGTGGAATTTAGACCATAACGAATTATAATTTTGGTGGTCGAAGTAATGAGATCTTAAAAAAATCGCGGCAAAGTATGAAAAGCTACTCGTATTATATCAATAAATGGGAATGATAATATACTACTAAGCGATATACATATGTACATATTTAGTAACTTAAATGTATAA

>SinsOBP4

TGTGTACAAAGTTTCATGATGATCAGTTGAGTAGTTTTCACGTGAAAGCATAACAAACAAATGGACATTCACATTTATAATATATATTATATTAATATATTTAATTTCACATTTATAAATTATATTATATTAATATATTTAATGATATTATATACTTGACCAATTTTTAGGGTTGACTGCTCAATAATTAGCTAGATTATTTTAATCTATTTAATTATATCTAGAATCATGAGTTTTGTGTTATACCATAGTTTGGGGACCATAGGGACTGAGCATTATCTAAAGTGTAATGTATGTTGTTTGTTTGTTTAATATAGCTTACCCTTAGTGTTTTGATGTCAACTAGCAGTATTTTTTGTTTTTCTATTAAATATCTATCAGTATTATATGGATTTCTGGAGGTGGAGACCTGGTGGCCGAGATACAGGTAACCCTAGTTTGATAACCAGTCTCTACAGATTTTTATTACAACTATTAAGTGGCTACAGCTATTATAACTAGTATTAAGTACTGTAGAGAAATTAATTATTATTAATTAAAAAAATGTTGTATCACAATCATAACAGTAGGAATGTAGACTATTCCAAGTGAAGCATAGTTATCACATCAGTGAATGCAATAGTCATAACTGAATCAGCAAGAAATCACAAACTGTGAAGTGACAGTTATACAAATTCGAAACAAAAATCTGTCACTTTTATTTTTGTTTTGTAAATCAACCTGTTATCCAAGTGTTTCAGAATGTTGAACTTTGTATGAACTTGATGAAAAAATTAATGGAATCAAACTCGTTCTCTCTGTTTGTCTGTCCAGTAAGCATCATAAGAAGCTGCTGGACAGATTTAAATGATACATTCTCAGTACACTTATGTATTATACTCTGTCTCTGTGAATAGACTCTGATGAACGCGCTTGCGTCATGCAGATGAAGTGATAGGCAGTTAGTTATTAATAAATAAAATTTTATTGCTAAATCATACTTTTGTAGGTTAACTACTTGTAAAATTGCACACAACAAGTACCTAATAAAATATTAATTTTATACTACACCTGCTCAGAAAGTAGGTTCTTACTTGCCCAAGAACGGGTCAGAAAATTCGTTTAATTGTCATACAGATATGCGGGCGGAAAAGTTATATGAAAATCCATTTCTCCCTGCTAGGGCGTGCCTTGCCCTTCTTGCCTGCTGGTTTTTTTTTTAAATTGTTGACAGTATTAAAAAAGAACAGTACACTAACTTTAGTCTTCCTAGCAAAAATATTACTAGTAATTTTAGTAACTACTAATTTTAATATAAACTTTAATTAGAGAAAAATTAGAGAAATTATGTGTTTTATTTTCCCTGTAAGTGTAGTATAAAGCACCATGAATCACTCGGTATAAAGCACCATGTATCACTCGGTATAAAGTGCGATTTTCAACGTGGCTGTATTAAGTTACACCCTCGTTGGAAATCGGTCATCGGTGAACAATGAATGTTTACATTATGTTCATTGAAACATATATTTTTATGTTCTCAATTCTGCTCTAGGATTGGTCTAATGGACTTCGAATTATACCTAGATTTCATATTAGCACAGTTGTTATTTTAAAAGATACATTTTACATTAGTATAAAACAGCGAGTTTATATAATATATAATATAAATGGTAATATTTAGTTAAACTGGACAGTGTCCAGTTCGTTCAAGATATGGTTCAGAAGATATCATTCCAATTGACTTATTTGCAGATAGATGATGTATACATACAAACAAAAATAAGCTCCAACGTCATGAGTTAGAAGTAGATTGTTTCACACGAGGAAGAATTCCGCCTTGTTAGCTTTCTGCCAGCAGACCTGCGTCAAGAATGCCGTGTCGCAGTCGTCGGCGCCTTTCTGCGTGCCGCACCCTCGGATAAACCCCTCGCTCTTCTTCATATGTTCAGGCAACATTGCGATCACCCCTTCTACTTCCACTTGCCCCTCGGAAAACATTCCCGCCGTCTCCATGACGCACTTGATGTAACATTTGAGCTTTGGGTCGGGCATCAAGTCCGCCCCGCCGTTCACCTGGTCCACCAGGCCCAGGTCGACTCCGGTCTCCTCGCCGCAGTTCTCTCGCAGCATGCGAGCCAACTCCGCCATCTCCTCGTCCATGGCGGCCGCGCCCGCCACCAGCGCGACTAGCACCATACACAGCGTCGAACTTTCCATCGCCATGTCTTCGTGTGTGGACGTTCGAAGCGATGATTGGATGCATGCAGCAGATGGCTCGTTATATAGGGCTCGCGATGTCACATGTCCGCCGATGATCGTTTTTGTTCAGTGGCAATTGGCACAGTCGATGCACAAATTACACCTTTCCGATCTCGTCTCTGTTATTA

>SinsOBP5

AGAAATGTCTCCTAAGAAAAATGGATTGTCAAAGAAGAAAAGAAAAGAGAGAATAGCTCCATTTTATACCATACCATACCATTTAATAGATATATTAATACCACTTAAAAAGGTATGTGTTTAAAAAAAATGTGTTGTAAAATGTGGAAAAATTATACACGTAGGTGATACTTTATGCACTGTGGTAAATGAAAAAGGCGGATCAATCTATGAAATATGTAATTTTAATACATCAAACTGTTATTTGCATCACAACCGTATATGAACAAGCATCTTAAAGAAATACCTTATATTTATATAACAAGCAAGGACGTGTGACTTTATAAAAAGACTTTATCAATATATCAATCATTGAATTCTCTTCGTTCAGCAGCACTAGTAATTTTCTTTGCATAATAATTTTTAAATAAAAAAAAGAGATGATTTCCTTGAATAATTCAGTCTTTGTTTATATTATTTTTTTTATAATTTTAAGCAGAACTTTTGTAATGTCGATGACCAGGCAACAAATGAAGAATTCTGGGAAACTATTAAAAAAGACGTGTATGCCGAAAAATGACGTCACAGAAGATCAAGTGGGCAGTATAGAGCAAGGCAAGTTCATCGAAAACAAGAATGTGATGTGTTACATAGCGTGTATTTATAGTATGGGACAAGTTGTAAAGAATAATAAAATAGTATTTGATGCAATGATAAAACAAGTTGATATGATGTTTCCGCCAGATATGAAGGAGCCTTTTAAGGAATCGATAGAAAAATGCAAAGGAGTTCCAAAGAAGTACAAAGACATCTGCGAAGCTTCTTATTGGACAGCTAAATGTCTCTACGATGCAGACCCAGCAAATTTCTTCTTCCCATGATCAATGCTATAATGATCAATGCAGTTAAAATGTGTTTATTAGTATAAATAACATTTAAAAAATATGCTATTAAAAAGAGGTCGCACATATTTGTTTTATTTTTAAATAAAAATCGTATATAAAAAATTGGAGTTCAAAAAAAAAA

>SinsOBP6

AGGTTGGTAACGCATTTGTGATTCTGGTATTACAAAAGTCTATGGATGCCGATAACTGTTTAACATCACGCGAATCGCTAGCTCATTTGCCAATTTCGACATAAAAAAAACATGATTAATTAGAGAATACAGACACAATATATCTATTATTATAAGTATTTAATTTGTTGCCTGTAACATTCATCTTTTCTAATCTCTCTACATGTAATATACAAACTAACTAAATGATATCCACTTTAAATTCTCATGTCGGATGCAGTGTCGGTTAAGTTAATGCATTTCATATCTTTTTATTTCCATTTCCATGAGACATTTCATGTAATTGTAGGATCTCTCGCATTTACAGAACTCCTTTCTATCGGTACAGGTTGCAGCCATTTCTTCAATATCGTCCATGGGACGACCACCTCTTTCTCCTGCGAACACGGATGCAGTCTTTGCAGGGTCAAACACACCTTTCTCCTCATCGAATACTCCAGATTTTTCCAAAATACACTTTATGTAACATTTTGGCGTCTTGTCCGTTTCGTCCAAAAAACTGCCACTTGCGTTTAATGACTCCAAGTACGACATTTCGATGTGGAACGTCTCGTTGCATTCATTCATAATTGCCATCATATCCACGTCATTTATTCTATTCTGTTCTTCCATTGACATTGCAGGTATAATTGTTGACATATTTATTTCCTCATCTTTATTCATCTTGACATCGTCCTTGCGTACATTTAGCGATTTTGCCAATGAACACCATAGTAATACTATGGTCAATAACAAAATAAATGACTTCGTCATTTTTATGACAATTTGGTTCATGTAATGTCCATGGAAGAACAGAATAGAATAAA

>SinsOBP7

GATACGATATCTCCTTAGTTCTGTAATCTGTGGTCAGCAGCTAGAGGGTGAACACCGTAGTGGCACACATCTCGGGTTTCTCCGTGCTTCGGAAGTTACGTAAAGCCATTATTTTTAGTTTTCATTGTCCTATATGGGTTGATACTGTTGTTGAACTATTCTTATCTCTTTATTGCCGCATTCAATTCAATTTATACTAAAGTTACAAAAACAGACATCATCATCATCGTGCATGAAAAATGACTTTAAAGAGTCAAATGTTTTTTAATTATTTTCATTTCGAAAACCAATTCACGAATAATTTAAAAGTACATACGATTTCATGAGTTTACAGCTCTGTCAAATGTGAATACATGGTCTGAACTTTAATTATTTTTTTTTGTAATAATCAGGCGCTAACAGATGAAGAAATTAAAGCGGAATTCATTAAACTAGTGATGAAATGCCACAAAGAATATCCAGTGGATATGTCCGAACTGTTGCAGCTTCAAAAATTCATTGTACCTAAGAAAACCGAGACAAAATGTCTATTGGCCTGTGCGTACAAGATTGATGGAATGCTAAACGACAAAGGCATGTATGATTTAGAACACGCATACAAAAGAGCTGAATTAACAAAAAATGGTGATGAAAAAAGATTAGTAAATGCAAAAAAATTAGCGGATATTTGTGTTAAAGTGAACGATGTGGAAGTAAGCGACGGTGAAAAGGGTTGTGAGAGGTCGGCGTTGATGTTCAAATGTCTCGTTCATAATGCTCCCAAGTTAGGATTCAAGTTGTGAAGAAAAAAAAATAAAAGCCATAGTTTGTGATGCTCCACA

>SinsOBP8

TTTGCCCCAAAGCTTCCATACGCCGTTCCATTGTAACTTTATCTAGTATGTGTTCCATTTTCATGTGCATACAATCATTGAAGACGGATGCGTATTCGCAGATTTCGAAAATGCCCTCGACGACATCGGTGCATTCCTCGACGGCGATCTTCAGTCTCGTCATTACTTCACTGTCGTCACTATACACGGCCCTGTTTGCTTCTATAAGATTTTCTTTGGAGATTTTATTGTTCTTTATTATGGAATTCCTTTTTAGAATGCAAGCGACGAAACATTTACCTTGCATCGTGCCCTGTGGTATTCTTTTGAGGAAATTTTCTACATCCACCATGGAGGCTTTGACTTGACGAGCGCATTCTGTTAAATTCCTCCTGAAAGGTTCCACTTCTTCGTTCATTTTGACGATCTTATCGAGATGCTCGCCTTTAGACAGAGGCTCGAAAGAGACGACGAGACAGATTTTCGAAGTCGACGTCAAAAATGTTATGAGTGTGACGAAATTCGTGAAAGTAAATTGCATTTTCGTAGCACGTTTTCTGGTCCCGCG

>SinsOBP9

CGTATATAAGCCGTCGCCTGTAAGACTTTATTCAGTCCTGTGTCATACGCATTCAACATGAGAACTTTGCTGGTATTCGCTGTCTGCTTCGTAGTCGCTCAGGCTTTGACCGATGAGCAAAAAGACAAATTGAAGAAACACCGGTCCGAGTGTCTCTCTGAGACGAAGGTCGACCAGCAGCTGGTCGATAAACTCAAGAGTGGAGACTTCAAGACCGAAAATGAACCTCTTAAAAAGTATTCCCTCTGCATGCTCATGAAGTCAGAACTGATGACCAAGGACGGCAAATTCAAGAAGGACGTTGCTCTAGCTAAAGTTCCTAATGAAGCCGACAAGCCCACAGTTGAGAAGCTAATCGACACCTGCTTGGCGAACAAAGGCAACAACCCACACCAAACCGCTTGGAACTACGTCAAATGTTATCATGAGAAGGATCCGAAACACGCCATCTTCTTGTAAATGTACAAACTGACCCCATCGGACGACAACAGTCAATTTCCAGAAATGTCGAGTTTGACCCCAGATCAGTCAACGGTCAATAACGTTATAGACTACATATATGTAACCTTTTTGAATCGTATATATGCAATATTCTAGTTTGCAGTAAGAGAAATAACGTGTCCCCAAATTTTGTATTCGGTGTCCCCAAATTTTATATCTGGTGTCCCCAAACACAAAATCTAAGCATGTATCATCCCTCTTACATCGTAATGTCAAGATTCAAACCATAATGGAACTCAAGTGGGAAATATTGTCTCAATAATCATGGGATTATAATGGGATTCTTATGGATTCAAAAGGGTTAAAACTGATTTGAACATCTAAATCAGTGCGTTTGCTCAATACAGACGAAATGTTCTCTCATAGTCAACGTACCATTTGCATTTTAATAACGATTCTATGGCATTTATAAAAACGATAAATTTAATATACAGTCTGTCATGGTAGGAAGAAAAGATTTTGAATATACTTATCTACTTTTTGTAGAATAAAATTACTGCCCAGGACGAATTTATTTTTTTTAATTTACGCCAATAAAAATCAATGCCTATAAAAAAAAAA

>SinsOBP10

ATTTCTTTGGCACGCTCTGTAGCGTTAAGGATAGGCGGATTTTAACATTCGAGGTGTTGCTAAATTCGACTTGATTGTGGGAGTGATATAAGTTAAAAAATGTTTTACCATTTTTGGCAGAATGTTATACCATTTTAGTGTTTTTCGTTAAGTATCTAAGCAGTCGGGTGTATATTTTTTAAATAAATAATATTATTACTTATGTTAATCAACAGATATTTGAAGATTATTAAAAACAAGTAGGTCAAAAAATAATTACTAATAGCATTAGCTAGACGGCCAAGGACTGTTCTTATAGTCGTCCTTTGCGGTTAGTAGTAATACAAATTCATACAAACTAGAAATATCGACCTGTTTTTGTTCTACTATGATATAGGTATGTAGCTTATCATCAATAGAATCAGATATTCTATATCAGAATATTTTATATTAAATTAATTTTTTAAACATTATGTATGTATATTGGTGCCATAACTTAAGTATTTTTATACATGTATAAGACGGCTTTTAAAATGTTTGCTTGTTTGTCTGAATTTTTGCGCACGTATCACATAAAAATGCCTGAACGAATTTTAACGGGGTTTTCACTGATGTATTCTGTCAAGGTCGGACTAACATCTAAGTTTTGCTTCAACGCAGTCGGTATTCGAGAAATAAAGTTATGTTAATTTGAAAATCAGCGATTGTCCATGTATCTAAACTCAAACGGATGCAAACGAAGCTGTGTGCACAGCTTGCTACTAATATAATAATCTGTGGGACACAGCACAGCCGGTAGTTAAATAAAAATATATCAACTTTTATCCAGACTTTATTCTGTTTTGCATTCATACTAACTATACTATATAATACAATAATAAATTTGGTAAAATACATAATTTATAATAACGATTTTTGCCCATATCAGTGGAAAGTTCTCAAATTGTACCATTCATTTTCCGTTCAGAAAAATTTAACATCAAGCAGCGTTCAAGTTTATTGGAAAATGAACACCCATTATCATTACCATCTGTTTCTAACTCCTGGAAGCAAGCTCGGATCTGATCCCGCAGAGCAACTCGTGATTGTTGTGATGCCAATGAAGACTGAACTCTGTTCCAGAGCTCGGCTTCCAGAGGAATTCCTCTTGAATCGATCACTTCTAGATTAGCGAAAACGCATTGGCTTAGACACTGATCAGAATCGTCCAGCAGTGGTTCAGTTCGTTTAACTCTAGTGTTATTTGCTGATTCATTTACAAATTCCGGACGTGTCACAGAGCTGGTCTGTCTTTTACCACCCCTTTCAGTATCATTACAACCATAATTATATTCTGTTGTATTTAAGACATGGATCTGTTTAGGTATACGCATATCTGTCGCATTTCTTTTCTCATGCCCATACTGATTCATGTCCTCTTTCACGCCACTGTCTATTTTTGGTGATTGTTCATCGTAGAATCTGTTCGAGCTTCTCACGTAGCGGGTTTGACGTTGATCTTCATTTCCTTTGACAATGTTCGTATTCGTGTCTGGATAAGAACAGGCGCGTAAAGCGAGAGCTATCTCATCTTCTAGAAGATGGATATTACCTTCGGTGGCAGAAGTAACCAATGAAGGCAAGAGCGCGAGAAATGCTACCACAGTTACCATTGTTATTAGATACACTGTTAATGTGCGGTAAATATAACCAGAATATAAACTGGTGATGCACAGGAAACTGGATCATTATTGTTGTCATATGATTCAGGCACCACTGCGCCATGTGTGATTATGTAAGGTACCATGTGGATTATAAATAACGAATACATAATGCAAACCGACATACATGGATGACAAATTAATGAGCAATAAAAGGTCGTCAGCACATTTTACGAAGGCTCCGGTGACATTAAAGCTGTTTCAAGGGTTTTTACTCGACTGCGCCAGAAGGAGGGTTA

>SinsOBP11

GGGCGAGAACACACATAGTTAAACTGTCAAAAGTGCGTGCACGCATAAAACCAGGTGCGCATTCGCTTGCGCGCGAGTGCTACTGCGAACGCTGCTACGGCTGCCGCTGATTGCTCTGTTGCTGCTCGTGCCGGTGTCGCTGCCACCGATACTCTACCCAACTGAGCCGACTGCACTGCAGAGCTGACACCATGCCGCCCTTGGTTACGAGCTTGGCGTTATTGGTCATCATAGTTAGTGTTCATCAGGCAACTTTAGGATGCAAAAATTGCATTATTTTAGGTAAAGAAGAAAAGGCCATGTTTCGTGTTCACTCTGACGCTTGCCAAGCTCAGTCTCAAGTTGATTCCAAATTGCTGGAGTCGTTGCTCAATGGTGAACTCATTGACGATCCAGGGTTGAGGAAACATGTGTACTGTGTGCTGCTAAAGTGTAAAATGATAGGGAAGGACGGGAAGCTGCAGAAGGCTGCAATCTTGGGGAAGATGGCTCCAAGAGTCGATGGAAGGAATGCTACGAAAGTATTAGAAAGCTGTTCAGAACAAAAAGGCGACTCACCTGAAGATGTAGCTTGGAATTTGTTCCGTTGCGGGTACGACAAGAAAGCGCTCCTTTTCGACTACATGCCATCTGGTGGCGGCGGGGATATTGACAACAATTCCAATTAATAGTTCGTAGTAACCACAAAAGATGCCACATGTATTTTTTGTGATTTACTCCATAGATGGCGTTCTTAGTATCCTAATATAAAATAGCAAAATTTAATCTTTGTGTTTCTTAAAATGTTATTTGATTTGTAAACTATTTAAGTATATAATATATTTGAATTGTTACCTTTTACATTAAATAATACAATTAACGTGAG

>SinsOBP12

ACAAATTTCTGCGAAAGTGAACATGTGCCACATAAAGTATTACATTTTTGTCGTATTAATTTTAGTTTCGGATTCTTTCGCTTTGAATTGTCGATCAGATGGTGGTCCGAAGGAAGAAGAATTGAAGGCTATATATATGAATTGCTTAAAAAGACAAGAGGGAAAAAATTCTAGCGACAATCGTGGATATTCAGGAGAGCTAGATTGGAATGAATCTCGAGGACAAAATCAAAGATCTCATTGTGATAAAAGAGATGATAGTAGGAGTAGAGACGAAAGGACGGGAAGTAGAGACGATAGAATGGAGAGTAGAGACGACAGAATGGGAAGAAGAAACGATAGAACACGGAATAGAGACGATAGATCAGGTGGCAGAGACGACAGAATAAGTAATAGAGACGACGGAATGACTGCAAGAGATGATAGATTTGGAAATCATGACAACAGATTTGGAAGAGAAGATCATTTGAATGGAAAAGATGATTTTGGATTAATGGAAGACGATTTTGGAAGTGATATACGCAGATACAACAACTACGGTGCCACTAACCAGCCCTCTAAAAGATTCAAACGTGAGCGACGTTTAGAAATGAATTCTGGACATCGAAGTCAATACAATCCGAATTTTTCTCCAAAGTCTTCAAGTTATGACGACACTTATGGTAACAACGAAAACAATTCAAGTGAGAGTAGTTCGAATAGCGATAGCAAAGCGTGCGCGCTGCATTGTTTCCTAGAAGAACTGAAAATGATGGGGGACAACGGCATGCCAGACAGATATTTGGTAACTCATGCGATCACAAAAGACGTAAAGAATGAGGATTTAAGAGATTTCTTACAAGAATCTATAGAAGAATGTTTTCAAATACTATACAATGAAAACACTGACGATAAATGTGCTTTCTCAAAGAATTTAATGATGTGTCTGTCGGAGAAGGGACGAGCTAATTGCGATGATTGGAAAGATGATATACGATTCTGATAAATGGATGAATTTTAATTTTCCAAGGAACAGTTATGCTGCTGAATATTTACTAATATATAAATAGCGAAGAAATGATGAAATTAAAAATATTATTTATTAATTTATATTAAATATATTTTCATTTGTTTATTTAACATATATTTGATTCTCAAACTAGTTAATAACGGTGCCTTGACGACCTCTTGGTGTGTCAGTGGGAGAGCAGCAGCCTCTATTGACACAAAGGGCGTGGGTTCAGATCCCACACGGGGCAAATATATAGGTAGTATGTACCTGGGTACCTGTACTATTGGTATTTCTCCAGTTCCAATCTAGTTTGAGATGAGATCGTTTAAAAAAAAACAAAACAAAAAACAAACTTGATACTCAGTGCCCTCTTCTCCAACGTTGTTATTCTAATTCTCATTATAAATATATTTAAATTAAATGTAATATAACTAATACAAATTGGAAATCTCGTAAATGGTCACAATTCGACCAGTTTGAAATTTTA

>SinsOBP13

GACAACAATTTAAAAATTCTGGGGGTATCATTCTCTTAAGTGTTTGTTCAATAATAATATGAATATGCAAAAAGTTCATTTGTTCTGTTTTTTTATTGTTTTGATAGATTTTTTGCTTTTCAACATCGGTTTTGCGGTGACAAGACAACAATTTAAAAATTCTGGGAAACTGCTGAAGAAATCGTGCATGCCGAAAAACGACGTTACCGAAGAACAAGTTGGGGAAATAGAACAAGGAAAATTTATAGAGGATCGTAATGTCATGTGTTACATCGCTTGTATCTACACTATGTCGCAAATTGTGAAAAATAACAAAATTAATTATGAAGCCGTGATGAAACAAGTGGATATGTTGTTTCCACCCGAACTCAAAGAGCCGGCAAAAGCCGCTGCGGCGAATTGCAAAGATGTGTCTAAGAAACACAAGGATTTATGTGAAGCCGCATATTGGACTGCGAAATGTATGTACGATTTTGATCCGAAAAGTTTCATATTTCCATAGGAAGAGTTCGCAATATATTAAAATTTCGGGATTCGGAGTTATATTATATTATAAAATTTTATTTTAGTGATTTGTTTTTTATTTTATTATTTATTGTAAATTGACTTACCCGATTCAGTTTATTGATATTATTGTTTGTCCTCTAGATAGTAATTGTACCACGAGTATAAAGAGTTCATTTTTGTCAATTACAATAAAAAAAAAACAATCATGCGGACCTGGTGCAAATTGGCAAAGACTTATATTATAAGTTTATAAGGCGAGTGTCCAATCTTAATATACCTAATTTATAGAACTTTCATTTTTAATGGACAATGCTTTATTTCGATAGGTAGATTTTTAATTAAAACTTTCGACGAATAAAACTTATTCTTGTTAAATAAACTAAAAGTTCAAAAGTCTCTAGTATCTCTGTATTATATTTAAACAAATTATTACCCATATACTAAATATAAATGTGAAAAAACCTTTCTTAGCGGTCTTTTCTTCAAGCTACAGCGACGATGAGTGAGTGAGTCGGGACATTTCTTTTACAAGTTGTTAATTAACTTTATATGTAAGTTAGTATCATTACAAAAGGCGGTGTACATACTAACACTGCTCTTAGTAATTAGGTATCGCGTTTATATGTAGATATATTATTAGATCCTCAAAGCGTATAATTATAGTTTCACCTTCATGAATGGTTTCCAAAATAAAACGAGCAACTATTATTGGAATATACTAGAATTATGTTTGAATTTTATTCATAACAAACAATGACATGCGAGAAAATAATTGTAGGTTTTTTAAACGTTCACAAATGTTACCAATTTTATGTGATTCAAAATGTGACATGATCGGCTTTACTTCAATAATTATTATTAGTATTAAATTAGGGGTCGGAGTCGATTAATTTCAGATATACCAACATCCATTTTTATATATGTAATATATATACAACGTTAGTATAGT

>SinsOBP14

GATTTTATTTGCTTCATTGTCATGTCATTGTAGAGTTTTTCCTGCTTCTTTGTATGCAGCCATCATCGCACGTAAAATGATAAGCGAGATATGTATCTCACAATAATTAAACAGCTTGTATATTTGTATATAAAAAGTCTGTTGGACTTTTATAAAACAGAGCAACGTCTAGGCCGGAAGCAATAGTCCGCTCTGCACCAAACATTATTTGTTTATGAAAATGGCCTCCTTTTATTGTGTATTCTTGTGTTACGGAATTGTGGCTCTCTATTTTATAGACGTTAACGCCGTGTCCCAAGAAGAGATAATCAAGATCGATGGCGCATTACTACCATTCATCACTGAATGTAGTGTTCAGAATGGCGTTAATATAGAAGATCTGACAGCTGCAAAGAAAAATGATAACTATGATAACTTAAACCCTTGTCTTATCGCTTGTGTGTTTAAAAAGACCGGAATGATGGACGATAAGGGACTGTTCAATTTAGATAAAGCGTTGGAAAAAACCAAGAAATTCCTGAAATCCGAAGAGGACATCGATAAAGCAGCCAAAGTAGCAAAATCTTGTGCCTCAGTAAATGATCAAGAAATAAGTGATGACGATAAGAGCTGCAAGAGAGCCAAATTATTACTCGATTGTTTTATCAAACATAAAGGACAGTTCCCCATATCTATATAAAGAGAAAATGAACATGAACGCATTCAAGACAATGAACTAACATATACATGATTAATTGTATACGGATGTATCTAACTTTTTTTCACTACTATGAAAGTACATTATATTCTATTTAAAAATAAATTATTTTGTTATTCAAAAAAAAAAAA

>SinsOBP15

TTTTTTTTTTTTTTATAATTTTTAATGGTTTAATTGTACATAAAAAAATTAAGTTACGCAAAGATAAAATTCTTTGGATCATTCTCATAAATGCACTTCGCTGTCCAATATGAAGATTCGCACAAGTCCTTGTATTTCTTTGAAACATCTTTACATTTCGTTATGGTAGCTTTTGCTGCCTCTTTTATATCTGCAGGATACATCATATCCACCTGTTTAAGAGATGCCTCATAATTCAACTTATTATTCTTCACAACTTGTAACATTTGATATATGCAAGCAATGTAACACATAACATTTCTGTCTTCGATAAATTTTCCTTTTTCTATATCGCCTATTAAATCTTCTGTGACCTGATTTTTTCCCATACATTGTTTTTTCAACATTTTTGATGAATTCTTTAACTGTTGTCTCGTCATCGATTCGGTGCAACTAAATACTATTATAATAATAAGCAAACAAATCTCTTTTCTCTCCATGATTGTGAAGAAAAAACTACACATTGAATGTTTCCTTGTAGCCGAACATGACGAGA

>SinsOBP16

TGAATTCATACACAATGTGACATAATATGTTATTATGGGCAGAACAACGTCTGCGAGCTAGAGCTAGTATAATAAAGATTTTAAACTTTATTAACCCTAGAAAGATAATCTACGTCTGTGAGACGTAGGCGGCGGCTAAATATTGCTATACATATTTTTTTACTTACGTCACCCAATGCATTGTATTTCTCAGCCAACTCTGTCGTTGGGTAGAGATGTCGTGGGACGGTTGTGCCGTAGCGATAAGTACCCCTAGCTTCTAGATATAGCTAGCCTGGAAAACCCTGTATGCGGCAATCACAAATAGATGTGTCATAAATGTGAAATTATTAGAAAGATATGCATAATAACTGATTAGCATTTAAAGTGTCAAAAAGTGCCAAATAACGACAATCAAAATCTATTAGCATAAAAGATTCCTTCAGAAGAAGAGATTATAATTTTTGTTGATTTTTAGAGGACTTTTAAGTTTTACATTTTAGTTTTAAACAAATAAATATATTAACAGTATTAGTGTTTTATTTTATTTTAAAAATAACTGAAGTAATATGTGAGATCATTTAAGTTATTATAAAATACTTCGATTTTCTAATATACACTAGTTATTAGGCATATGTCTCTAGAACGTAAATTATCTTTAACGCCGTTTGGAATATGATTATCTTTCTAGGGTTAACTGAAAATGTCAATAAGCCTGTGTTTTTTATAGATTTACTTATTCGATAATAAATATCGAATCATTAAAAATGTATTTCAGTTGTTAAAATGAAATTATCACAGGTGTCCTCTTAATGAAGCATTTAAATATCTTAAACGTTGTATCTGCCGGATCTTGGCCATTTTCTTTGTTGCACGATTCCATTGCCTTTTGAAGGGGAGCTATTTCAACAGTTTTTGGATAAAACTGTAAGACTTTTTCAATTTTCCAATGACCCTCGGCTGTAGTAAGATCAGATTTGGTAAAATCGCAATAGATAAACTTCTTAAACTGTTCTTCGTTGCTGATCTTCATGCTGTTTAGTTCAAGAACTATGCTTGGATCGGAACCAGTTTCACTGATACATTGCGTTATTATCGGTTGTAATCGAGAATTAACATCTGGTGCCAAACGTATAACTTGCTGAGCAGATTTTGTGTATGCAAGAGATAGAATAAAAGAAAACACGATTAAGTAATACATTATTATGTGAATTACAAAATTAAAATAAATATGTTGTACCGACAGCTCTCTTTAAGTGAATGGTTTGAACACTAGCGCGCTTATAATCTTTATAGCCTAGAAGTATTCTACATAGATAAGTATAACAATTACGTCAGATTAAGGATAACTAAATAAAACGCATTGAAAATCTCTAAACATCCTGACACTTATATAACGACGACAACTTCCTTTCGTAGAGGCTATTTTTAACCGACTTCCAAAAGGAGAGGTTCCCAGTTTGGCTGTATTTGTTTTTTTTATATATGAATGTTTGTTTTTTTTGTTTGAAATAGTGTCCTACCAAGGGGCTACCAAGTAATTTTGATCAAAATATGACCAAGGACTTCGAAGATATGCCGAGGAACTCCTCAATGAATGATGAACATTGAAGTCAGTTTTTTTTCTTAAAAAACTTTTAACCATAATTTAAAAAAAATATATATAGGTGTAAATTAATATCCCAAAAACACGAATAAAATAAAAATGAAATAAAAATCCTATAACAATTACTTACTACATATATATTAATATATTTTTTTTAGATTTAATAGAGATGTTTTTATCAAACCAGTTGCTCTATACTCAGACTAGGATACAACCTGTAGTGATTAAATCCCCTTCGACATTGACAACCTGTACTATAGGTCTAATCTACATAAGTCAAATAGAAAGGACGTTTTTTCTTCATTAAACTGCTTTACATTACATTATTATTGGAAATAATCTTAGTTACCTAAATGTCAGATTTGAATTTACTCATGTATAAACTATTTTCTTCTTTGCCCGAAAGAACTGCTTAAACAGTATCCTATGAAAGAAGAACAGGATAAACAGTATCCTATGTCCTGCGTGTCCACATATATTCCTATGCAAAATTTCATATCGCTATCTGTTACGTACCTACATCGTTTAAGAGCGAGTAATGTACAAACAAACGCGCGCACAATCACAATTATAATATATAATAAGTACCTATGTATAATCCAGTTAGGAATTTGGGATATCGCCATTCATTCGTGAAAAATACCTCTAATTTATAAAATTAAATAATTTTCACCATTATAAATGGACAATTCGTCGGCTACACTTTGGCAGCCATAGCCGAAATAGAATCCCAAAAAAATATTGTTTGTTCGGAACTTATATTCACGA

>SinsOBP17

TATATTTATGATTTTTTAGTTAAAATGTCAAATATTTTTCTATTTTTATTATGTTCATATTTGATCAGTGCGACATATGCAAGGTCGGACGAAGAGATAAAACAATGGTTCATAAGCGTAGGTATGGAATGCAATAAAGATTATCCGATTACACCGGATGACATGCAGACAATGATGCAAAAGTACCAAATTCCTAATAGCAAAAACGCTAAATGCTTGATGGCGTGTGTCTACAGGAAAGCCAATTGGTTGGACAGTGAAGGTATGTTCGATGTGGACAGTGCGAATTCAATGATGGAAAAAGAACACGCGGATGATCCCGCAAAGATGGAAAAGGCTAAAAAATTATTCAAAATTTGCAAGAAAGTTAACGACGAACCAGTAACTGACGGCAATGAAGGTTGCGATCGATCAGCTCACATGTTCCAATGTTTGGTTGAAAATGCTTTAAAGATGGGATTCAAAATACAATAAACATCTGTGATCCTGATCATGTAGATATCTAATGTTCCACATAGCATGGTAAACGATTTAAACTTAAATGATTTTAATAGTA

>SinsOBP18

TTTTTTTTTTTTGTTTAAACATTTATTAAGGGAAATAAAATATATCAGGATTATATTTGTAAATACATTTGGTTACGTGAAATGACGCTTCACAGATGTCCTTGTGTGAGTCCGCGACTTTTTTGCAAGCTGTGATAGCTTCTTTAGCTGGTTCTTTTATTTCTTCTGGTAACAGCAGATCAGCCTGCTTAATAGCTGCGTCGTAGTTCAATTTACCATTTTTTATCGTATTTGCCATTTTCATGATACACGCCATATAGCACATCACTTCTTTTTCTTCTATGAATACGCCATTTTGTATTGGATCGATTTTTTCATCTTCGACGTTATTTCTTGGTTGACACGACTTTCTCATCATCTTTCCAGTAGATTTTATTTGCTTCATTGTCATCGCATAACAAAAAGTTGTTAATATCAATAATTTAACAAGTAAAATTAGGATCATTTTCCTAATTGATGAAGAGATATGAGATATATCTCTGAAAA

>SinsOBP19

AAATAAATTATTTTGTTATTCAAATGCGTTTTTCTGTATCCCAAATTACCTACTTTTAATATTAAAAAGTACTTAAAATATACTACCTATATTATAGGATTGGATCTCTAAATTTGATCTGACGACGAAAATTATTCTGCAATTTATTTATATACTAATACATACATAGATGTCTATATTTTACACTAACGTATGTCAGTGTTGTAGTTTTTATGTAAAAAAATTACAGTGTATAATAGAAATATTGTGAACATTTCAAAGTTGAAATTACTATAGCATTGTCAATGAGTACCTATTTATATTACGATTCGTTCCGGAACCGAAACTAATTTTTACAAGTTAGCAAAATCCGTCGTTTGGGATCCATTTTATACGTAACAGCGTGTTTCCGTCACTAAATGAAGGCCAGAAAAATATAGAAATTGACGTGCACCATTGATGCAGTAAATGAGAATAGTTGACGTCGGCCTATTTAGTATAAAATCACTTGTAACATATATTATGTCACTAATACTGATTTATATTTCAAACGGACGTTCTAGAATTTTAACACGGTCATGTATAATAATTTTTGTTATGTTTGTTTCTTTTTAGTTATTTTTATAATAAAAGATGCCTTGGCAATAACTCTAATTCAAAAAACTAGGATCCAAGCAAAGTTTCTGGCAAGTGGAATTGAATGCATAAATGATTATCCTTTAACGGCGAATGATATTCAGATCTTGAAGAGTAAAGTGATGCCTGAAAATGAAAATGCTAATTGTTTTGTAAATTGCCTGTTCAAGAAAATTGGCATTATGGATGATATGGGTAAAATGACGCAGGCCGGAGCCAGGGAATTTGCTAAGCAAGTTTTTAAGGACGATGATGAAAATGTAAAGAAAACCGATGAATTATATGGACAATGTTCCTCCGTTAACGAGAAGAGTGTGAGTGATGGAGACAAAGGGTGTGATCGAGCAAGACTAGCGTTCATTTGTTTAGCTGAAAATGCACCTAAGTTTGGACTGGATGTTGATTTCTAAACAGTAGAAAACGCAAAATTTTGGAAAAAAAATAGGTTCCTCTTTGTAGATGTTACTTTGAAGAAGCTAGTGGATTCTTTGTTTGGGTTTGTTAAATAAATATTGAAGCGGGGAAAAAAA

>SinsOBP20

TCAATAATTATTATTAGTATTAAACTTTATATAAATAAAATAGAAATAAAATAATATATAATATATGTATAATGTCTGAAAATTGTACTATATATGTGTTTATAACACAGATATTCCCAAAATACTATAGTCAGTCGCTGCCTTTTAAAATGCGTATAGACTATAGATTTTACAAAATGTTACTTGTTACTTCCGTTAGTAAAATATGAGTTACTCTGAAGTCGTGCCTAAAGAAGTTTTACTCCAAAATTAGCGTTAACATTTTTCAAATTACTGTTTTTAGGATACAAAAAAGTAATCAAGGTTAGATGGCTCTTATTGTTAAAGGAAAGAAGAATATAACAGAAATGTTGTTGAGTTTTAATTATACATATAATGATATGTGTTAAAAACATAGTAATTACAGGTTAGGTCTGGCTGTAAACGTTGCCAATAACCATCTATTTTTGGTAGATAACTAGTATTTTGAGTTACATGATTTTGATATAAGTCTGACGACAAATAGTCTGTTTATTAGTACTGAGCAGGATATATTAGGATTTAAACGTTATTTGTCGCTAGTGGAGAATATTTTTTGTAACATAGCGAAGTAAATTAAAGTCAATTAATTAAAAGAAAATGCGCCTTTCGTTAGTTATTGTTATTTTGATGAACATTGTCTGTGCATATGGAGTAAGACACGTTTCTATTATTTAATATTGCAAAGGGCTGTATATGATGCTTAATTTGTCATAATTATACCGCCTTTAGTATACAAGTAAAACACTTGGTTTTTTTGTGTTAAGTACCTTAATAATTTTTGTATGCATTTTAATATTCTTTCCCCGATTAACATTTCGTTAAACGTAAAATAATAAAATATTTATACTTTTCATATGTTGCAATTTTGAAATTGTATATTTATATAAGCATTTTTTTATATATAGATGACTAAATCACAACTCAAAAAAACATTATCTGTTACGAAGAGACAGTGTATGCCTAAAAATGGAGTTACAGAAGGTAATTTAGACATATTCACCTTATAGTCTTTTCTCCTTTTCTTCTTCAGTTTTTTTTACACTTTAGTACAAGATACAATTTAATTTTCTGCTCTTAGTTTCTTTTTCTTAAGACTTAGTTTACAACCACTTAACTACGTGTACAAACGGCCTGTCCGAATTATTGTGGACTATTAAAGTTTACGCATACTTAATTTCTCTCTGTATGTTCTGTAAACGTTATGATGAACTTTTCCAACTTGTCAAAGCTTTTTTCCACATCTTGGAAAAAAGCTTTGAACTTATTACATAAGAAAAAACTTGATTGTACCAGTGCACTGATCATCACCGTAGTGAAGTTCATCCCCATTACCATGAATTTGGAAGTTTCCATACAAACTGTTTTATTGTGCATTTCTTGTGCGAACCTGCATTTATAGTGATCTTCCTTCGAAAGTTCTTCTTTTTGCGTTACGATATGGGTTTCTTCATGACGTGTGAAAAAAAGTCTTGTAACAAATATTTTACATGGGTCAAGATTTCATTGAGATATGTCAAATATTTTTTTTAGATCAAGTTGGACACATAAACGAAGGAATATTTGTAGAAAATCATAATGTTATGTGTTATATTGCTTGTATTTACAAGTTAACTCAAGCGGTGAAAAATAACAAACTAAATTTGGATTTATTGATAAAACAAATCGATATCCTCTATCCTACAGACATGAAGGAAGAAGTAAAAAAGTCAGTGTACGCTTGTATTCACGTTCAGGACAATTACGATGATATGTGCGAGGCAATCTTCTACACTACTAAATGTTTTTATGAATTTGATCCAACATATTTTATATTTGCATGAACGCAGCATAAGGAAAAATTATTTATTTTAAGAGTGAAATTTTATAAAAATTCTATGTCTCTAACCATTACAAAATGATTGTACATAATTCACTAATATTATATTTTCGGCAAGGTCCCTCGTTCTGTGCCTCCCAGTATATGTATAGATAACCTGTGTATAAAACCATATTATGTGCACCTCGATTTAGTTTTACTATTTTTCCATTAGCTGCCACAAACTGCATAGTTCAAAGTGAATATATATTAATATAAATTAAACTTATAATGTGATTTAATTTATGCTAACCAAGTTTATATTAATTAATCCAAGGAAATGTTTTATAGTCGTCTTGAAAAATATTAATATGTCAATTATAAGTAAAATAAGTTAAAGAATGCAAATATATGTCAAAATAAAAAAACTGGTTTAAGATTAAACTGGGTTTTAAACCGGTTTTCTGTTTTAACAAAAATGCATTTAAAATGCATACAAAAATTAATATTTAACACGATAAAACTGGGTTTTAATCCTTTTAAATATTAATTTTTGTATGCGTTTTAATATTTTTTCCCCGATTAAACTGGGTTAAAACCCAGTTTTATCTTAAACCAGTTTTTAAGAAAGTTATTTTATTTCCTTACTACAAGAACGTGTTTCATGTTTCAGTTTTTTCTTCATTCTTTCTTTTCTTTCTAAGATGACTTCTTCGAAAAAACTGCCGAAATATTTTGATATTATACTGAGTATACACTGTATTTAGGTAATAAAAAAAAAGAACAAACATGTTAGCCATTTTTTAAACCCGTTATTAGTTACGGGAATATAATAATATCGAAAAAAAAAACAGGTTTTCTATGTCCAGTGAATTTTATTGTTTAAAATGTATTAAAATGTGTACGTCCTTTGATTGTTGCCATCAATTCTAAGGTACCTAGTACTTTGCAAAATACTGGTTTATTATGGTAAAATTGAAAAATTAAAATTGAGTAGTTTCGGTTACGACCAAACAAACACTTGATGATGGTAGAAATGTCTCCTAAGAAAAATGT

>SinsOBP21

ATTCGCAAGTTACGTTCCATACTGCGATAGATTGCACACCTTTCGTCGCACGCTCTCCAATTCGTCGAAAAAAAAAGTTTTAAATTCGAAATTCAAAACAATAGTGATATAATTCCCTCCATTTTTAAAATTTTATTTGGTAATAATCGTGTATTTGTGTGAAAAATGGTTCCTAAAATTAGTGCACTTTTGTGTTATATTTGCGTATTCAGTATTTCGTTGAGTGATACAGCAATATCAGCTGAAGACGAGAAAAGATGCAAGAATCCTCCGACAGCTCCACAAAAGATCGAACGAGTCATCACTCTGTGTCAAGATGAAATAAAGCTGTCCATACTTAGAGAAGCTCTGGACGTAATAAAGGAGGAGCACACGATGCCGGCGCAGCGCATACGCAACAAGCGCGAAGTGCCGTTCACGCACGACGAGAAACGGATCGCCGGATGTCTACTGCAGTGTGTGTATAGAAAAGTGAAAGCAGTGGACGGTTTCGGGTTCCCTACGCTGGAAGGTCTGGTGGGCTTGTATTCGGACGGCGTCAACGAGCGTGGCTACTTCATGGCCGTGCTGGAGGCTTCCAGGGAATGCCTCATGAGGAACCACGACAAATTCTCCAGAACCACACCCATGGATAACGGTCGCAACTGTGATGTATCGTTCGACATATTCGAGTGCATATCTGACCGCATCGGCGAGTATTGCGGAACTTCGGGCCTTTAATAATTCGCAGCGAATTATTTTCGTTACATAAACATTACTGCTTTATACGACCACCTACGGAAGAAATTATTTTAAGAACATTCAATTGTATTGTGATATATGCAGCCAATAACTATTCAAGTAGACGTGTTGTTCAATTGATGGATGAAATTTTAATCACTAAAATCAAACGGAAATAAATAATAATTATTTGTGAATTTTACGTCGCTCGAATTTTTTAATTGGTAAGTAATTAATTTTTGATATAAGATATATGTATTTACCTATATATTAAATTGATAGTGACTAAAACTTCATCCTATAAAGATATATGTAACAGAAATTAGTAGTAATAGTAAAAGATATAGGAAATAAACAGAGTAGGTACTATTGTCATGTAATAATTATCAGAATCTAAACCTGATAAGCACAAGGATATACGATATACTAAAAAAAAAAAATACAAAAATTAACACAATTTAAGTAAAACGGTGTGTTTGATTCCATAGGCCGCGGTTTCCATCATGCTTTATATGAGTAAGTGAATAATAAAATTAAAACATAGTTAATTATAAAGAGGAAAATTTTCAAAATGCATATAAAAATTCATTTTGTACATTTAGAAATTTTCATTTATTTATTTTTATAAAATTTAATACGAATCCTTGCATTTTAGACCAATCTCTGAGATTATTTAAATAAATAATACCTACTACTCATTTCTCTTATTTACGTCAGTTATGTGTCTCTAGGAGCTAAACTACTGTTTTAATTAATACATGTAAATATGTATTTTCCATAATTGGGAGAGTTTATCTCTTTCAATCTGATATATGAGGAAATGGTTACTTAATTATATATTATATATGTATTTCTATATGTCAAATGCAATAGATTTTACCAATATACCTACTAGTTACCGGTTTATCATAAAACAATCACATAATCTTACGTAACTAATAATAAGCCTTGTACATATTGTGATAACTTTACTTTTTAATGCTCAAATAACATATTAGTTGTAAAATTAATTATGATCTGTTGGATAAGCAAGTAAAAACAAGACAATACATATTATGATTTTGCTTAATTCTTTGATTTGTACTTTAAAAAAAAACCGGTCAAATGCGACCCTCATAATGAACCCTTTTATTTGATATGTCACA

>SinsOBP22

TAATGGTTATAACAACCCACACTTTACTGCAGAATGAATATCAAATGCGTTGCTAAATTTCACATAAAATTATTGCAGCTATTTACATATTACATTCAACAATTGTTATAGAATATATGCTTAAATGAATAGAATTATATGGGAAATCCTTTTTTTATCATCTGATGGAATAATATTGCACATCAATCTGTAAAAAACCATCCATGACATACATGCAACTTTGCCTCCTTAGCTACAAAAAATAATAGAACCAATTGTTCTTTTACATAGAGTTTCTTCATGTCAACGCAAATTTTATGATAATTTTTTGAACTCCAACCAGTAATAAGTACTTATTACGCTCAATGTCATTCCCCAAATACATTGCTTCGTGAAAACTAATAACATAGATACTGAGTAGCTTATTATTTATTAATTGAGATTTCAACAATATAAATTCAGAGTTTCGCCAAATTTTGTCAAAACTCAACAACTACTCAGCTAAGCTACAACTTCTGATTTTAACTTTTAAGTTAAAGTGTTCACAATGTCTCAAGCAATAGTTTTGTTGTGTGGTACATTCTTGTTAGCTTCTATTCCTTTTACAAGTGCCGTCACCGATGAGCAAAAAGCAGAAATTCAAGCATACTTCGAGAAAATTGGGGCTGAATGTATCACAGATCATCAGATCTCGGAAGCCGATATTACTGAACTACGAGCCAGGAAAATTTCCTCTGGTGAAAATGTTCCGTGTTTCTTAGCCTGCATGATGAAAAAAATGGGCATTATGGAAGACGGCGGCAATTTAAGAAAAGAAACAGCACTAGAACTAATCAAAGAAGTTATCAAAGATGCCGACGAACTGAAGAAAATTGAAGAGTACCTGCATTCTTGCTCCCATGTTAATACCGAACCTATTGGAAGTGGTGAAGTGAGTTGTGAACGTGCCATGTTAGCGTACAAGTGCATGATTGACAACTCTTCTCAGTTCGGCATTCTGGTTTAAAATCATCAAATTTTCCTGAGAACAGTTTAAAATAAATGTTCTACGACCACAAGGCAGAACTCGGATAATTGAAGCGATAGATAGTTAAGTACATATTATATGCTTAAGTTAAATATGATTTATATTTATTGTACTATTTTAATGTAAGTTATAAAATAAATATGCATGTTCAATTAAAAAAA

>SinsOBP23

GCAACAAGACCACGCATCTCCGTTAATGTGTTAATTGCTAACTAAGATAACTACCGCTACTCGCCACTTTACTGTTGAGATCCAAATCCATAATATAAAACGGTGAGTTAAAATAGCGTTTATCGGCGCATAGGCAGCGTAAGCGTCATGTCTTACACGACGCTGCCAGTTTTTCTACTATTGATAACATTTGGTTATAGTGCTAAAGAAAAACCGGAATTCAGTGAGGAAATCAAAGAAATAATCCAACATGTTCACAACGAGTGTGTCGGTAAAACCGGGGTCGCCGAGGAGGATATAACGAATTGCGAAAACGGCATATTTAAGGAGGACGTGAAATTGAAGTGCTATATGTTTTGCCTGCTAGAGGAGGCGAGTCTCGTGGATGATGATGGCATTGTAGATTACGATATGATGGTTAGTTTAATTCCAGAACAGTATTACGATAGAGTAACTAAAATGATATATTCTTGCAAACATCTGGATACGGAGGATAAAGACAAATGTCAAAGAGCATTCGATGTCCACAAATGTTCGTATGAAAAAGATCCAAGCTTTTACTTTTTATTCTAAACCTTAAAACACAACTGATGAGCGAGAAAATAACACAATATATTAATAAAATATTCTAACAAGTAATATTGTTTTCAACCGACTTCAACAAGAAGAAGGTTCTCAATTCGACTGAATGTTTTTTTTTTTTTTTTTTTTTTTATTTGGCAATCAGTCGCCAACTTGGCCACGACTATTCTGCCAATGTCACGT

>SinsPBP1

TATTAGGTGGATGAAAGATTTTTTTTTTTTTAAAATTCTTCAAGTTAATTTTATTAAATTAAAAATCTTTCATCCACCTAATAAAAGAAATGCAAATATGATTAAAGTACCTATACCCTCTTACAAACTTCTATAAGAAAAAATTAAATTATCAATTACTAAATTTCTTATTAGTTATTTAATAGAATTAACATTTATTGGATAAATAAAGACATAAGACATATAATAATGTAAAGTCCGTTTTACTCGCCGGTCCGTAAGATAGATTAAAAGAGTCACTTCATAACTGATATAAGTATTTTTAGAATCTTGTATCCTATACTTATTAATAATCTCAAACTTCTGCTATCACTTCTCCTACCATCAGATCCATGCTCGGTGCCCAATCGAGCTTATGTATTTCTGCCTTGAAGCATTTAGCTATTCCCAACATCATGATACAGCCATCATCGTTCCGTGGAGTTGATTTCTCACATTGGTGTATAATGTCTATCAGTTGTTTTGCCATTGCATCATCAGCGCCATGCTTTTTGGCAAATTCGTGAGCATTCCCGTGATGAAGGCCTCCATCAGGATCCACCAAATCTAACTTAGTAGAGAGACATATGATGGCACAGCCTGTATAGCGATTAGATACTTCGTAATCTTCTTTCCAGAAGTTGTAGAAATCCGCATCTATGGAATCTGGTAATCCCATCTCCTTTTTACAATCTGCCAACGCTTTTCCGAAATTGACAGTCATCTCTTTCATTATCTCCTTCGACGATTCAACTCTCGAATCTATCGCCAAATACACAATCACCAGAACCACTATTTTAGTTTGCGTTAACATTTTCGCTGTATATTTTTGTAACGCTTCTTTGTATATTATGTCTGCTACTTCCGAGTCCATTGATAAATTTACACTTGGTTTTATATAGATTTGTTGTATAATTAAAAATGTTTTGATTACAGATTCGTTATTTAATTATTCATTGAGCTTTAATGACTGTGCGAAGTCATCGTTAACTCGATAATTATTAACTGTCAAAGGTGTATATAGAAAAATGGATTTTAAAATTGTGTGTACAGCAGAAATATTAAAGCTGATAATTTTCACGTCCACGTCACGTAATAGGGTAACATCAGAAATTAAATGCAAAAGGATAATTATTCTAGTAACGAATTT

>SinsPBP2

AAAAAGGAGAAGGTTCTCAATTCGGCAGAATTATTTTTGTGTGTAGGTATGTTATGCAATATCTTCGCTTATTGATACCATTACAATTTGAAAAACAAAATGTTTTTTTTTATAAAAAGAAACCATATAAAAATTTAAAATTAATACATAATTTTTGGAGTTGGTGCCAGTCAATATAATGTTTCGTATCTAAATAGACAAATCTCCTTGCATTGACTGTCACCGACTTCAAAAAATATTATTTCCAATTTTTGAGTTGAAGCGTACATATTATGAAGGCAATTTTTTGTAGTCGTGTTTAATTTTTCATAAAAAATATTTTATTAAAATTCTCCATTTAATAGTTTTAACTTGTGACATTGTCCGCATATATGTAGATGCAAATTCTCAAAAGAAAAACATTTTAAAAATCAATTAAACAAAATAAAAGTGTGTGGAAATCTATTTAAAATTCTTGTAATAATATTTGCAATATAAGAAAAGCATGTAATTGCTAGACACAATTTTCTTACATATTTAAATAATAAAAAAACAATTCTGCGAAAATGTGGATGTTTATTTTGTTAGCATATAAGAACAATTTCACGTGTAAACCCATTATTAAATCAACCTTCCCTTGAAAAGAAAAAAAATTACACAAGTAATATTTAAACTAGGTAGTATAAATATTTAAACTAGGTATATAACAAGGTAAATATACAACTGACGAATGTATGAGAAAATAACAGATTATATATAACTTAAATTATGTAGGTAATAAAATCACAAGTCCATGTATGCATAGCCCAGGTCGCAATTAAGTCTTCCGAATTCTTGTAACTTTGCCACAGTTACTAATGTTCAATGTGTACTAAACGTGGGTCACGTTTGATCCTTACGCGGTTGACATAACGTTTATTCGAACTAAGTGATTTGTTAACTCGTTTATCTTACGTACTGCCAAATTGGAGAATACTCTCCTGCGTCTGTTTCCCGATTGACATAACTTGGGTCTCTGAAGACTACAGTGAAGCATTCGTTTGAGCCAGTGCTCACGATCATTGGCCTCTTCTTCACTTACCAGGCCAGGATCTGTCTATAATTGATAAGAATGGAACTTAACAAACGGTAGAAATCTGCATATCCTATGAGGCTTTTAATACAGTTCTTCTGTTTTCTCTTCTTACTAATAAAATCAGAAATAAGATTCCCTTTATTACATTTCTAGTTACTAATACTCCTTAATTTAATTCAAGATTCGAAAATGAAAATAGTTACGATGGTAGGTCTACATTGCTGGTTACTGGTATTAATGTTTAATAACCAATAGGTCAAACATTTATCTATTGAGATGAATTCTTAAACACCTTATATTGTCTCACACAGCAATCACAAAAATTTCTTCAATCTTCGTTATGAGATTCAAAGAAGAAAAACTAATGTATTCTTGACAACTTCGTTTTATAAATATCTTCATCAATATAAAAGTAATAAAATAATCATTTTTAACATCAAACATTATTAAATATGGCTTTAGCTTTAAGTAAGACTTATATTTCAGTCATAATCTCTTCGATGATCACCTCCATGGGAGGTGCCCAATTTAGATCATGGATCTTGGAGCGGAAGCATTTAGAGATCTCCAGCACTCTTGAGCAGTCATCACCAATGCCTGCATGAGTGTTCTCACATTCATGGGTAATTGTCACTAATTGTTTGGCCATGGTGTCATCAGCTCCATGGCTCTTGACGAACTCATGCGCTTTCCCATGATGCATTTTCATCTCATCAGTGAGGAGGTCCAATTTGCTCGCTATGCACATGATGACGCAGCCCATGTCCTTGTTCAGCAGCTCGTAATCTTCACGCCAGTAATTGTAGAAATCCTTCATAATGTGATCACCAACGTTCAGCTCATTTTTGCATTCTTCCAACACTTTTCCAAAAGTGCTACTTAAGTTCTTCATAACATCTTGCGACGAATTCACTTTGTGCAAATCAAAAGCCAAAACTGCAAGACATATCACAAACGCCCGCCATTTTGTGACTGCCGCCATCTTGTCGACTGCACTAAATAATTGAATTTGTCCCGCCGTCTTTATATCTTTCATGAATCGTTTTTAATTACAGCTTTTGATTTTAATGTAGGATTGATAGGTTTTTAATTAATAG

>SinsPBP3

AAATACGTCAATTTTCAAAATTTTATGCAGTACCTTCTTAAAATTTAGATCACCAACCAAAGATCGTATGTTTTTCAATTTTCTATATACTCTCCGAAATATGTATATACTATATTTCTAGTGTCCTAGTGTACGCAGGAACTACATTGTTTAATTCCTTCGGCCTTGTTTCAATATCGGAACACTAGATGCGGCTTAGGATTTTAATGTTTAGGTAGTTAATATAACATTTAATCGAACTAAGCGATATGCGTATTGTCAAATTATGGTATACTCTTCCTGCGTCTGTATTTTTCGATTCATATAATTCGGATCTCTTCAAGACAAGAGTGAATAGGCATCTTTTGGGTCAGTGCGCACCATCTTCGGCCCCGTCGTCGGTTACCATCAGGTGAGACTGAGACCAAGCGATAAACATTATTCATTAGAAATTATTATGTATATTAGATTTTCACAATGAATTTTTTTACAATGAATGTCACTTTAAACTTCATCGAGAAGCACGTAGAAATGAATAGTTTATTATTTATTTTAGTTTTTTAGACGCCTCGTCATTTGCACATGATGTGTACAACACATTATGTATATTGAATAAATTTATCTGTAAATTCTTTATTTTTGAGTTTATATGTCATGGAAATAGTAATCTAAAATACTACCCAGTCATTGTATAAAGTGACATTTTACATAGTCAAGGGTTAATGAAATTAGATTTATGTAGATATCACCATATATATTATTATATATTATATACAAATAAAGTAGATGTTAAGACTTCTATAGATAAGTAAGGTAAAGAGTTTAATTCGGTTGCGATATTAGGCCTTGCGTCCACTCGAAGCGGATAGCTGCGAAAACGTACAAAATTGAATGCGTCCAAAAACTTGCGTAGGATCTACGTAATGAATCAAGCCACGTCCACTAGGCGCGCATTATTCATCCGTTTTCTGGTGTATACTTGCGAGACACGATCCGATCCAATTCAGTTCGGATTTTGACGTGCGATTGCTGAGTTCAAATTCAATGGATTGTAAAAAAATTTTCTTTTGTAAAAATACATTTTATTTTTAGTGGTAAACTTTTTTAAACCTTTTTATAAAACACCGAGTTTTATAGTCTAATATGAAATTTCATCCCCTATTTTTCCACCCTTAGGGGTGGATTTTTTCTTGAAATTTAAATGGGACCATCCTCTAGATATGGCCTATTCAATAAAAATAGAATCAATCAGCGAAGATATCGCGTAACGTACCCTAAAAAAATTTGTGGAATGGAGAACCTACTTCTTTTTAAAAAGAAATCAAAGGATCTAAGTTTTCTGAAATTCCTATTTAAACGTTTATTAATATTTCTATTTCATTTCAAATGCGAAAATTGTTCCGTCAATATCAACATTGACTCTTGTACATATCTTAATTAATACTGTTTTAAACTCAATTATAGACCGTCTAAGTCTCTACGTAGCTATTAATTATTGCTAACAATTAAATACAATTAACGAAGACAAGATAATCATTAATGTATACGCAGAAATTCTATATATATGGTAAATCACTGTAGATTTAAACACTTCTCTCTGGCAAATCCATAAAAATGGCAGGACAACTGCAATTCTTTGTCGCTCTAGTGCTGTTTGCTATAGGTGTAACTGAGATAGATAGTTCAGCAGAAACAATGAAGAATATATCTTCAGGGTTCATTAAAGTTCTGGACGAATGCAAACAAGAGCTGAACTTAGGAGAACACATTCTAAACGACTTCTACCACTTTTGGAAAGAGGAATACTCTCTTCTGAACCGTGAAACAGGATGTGCCATTATATGTATGAGTAAGAAGCTGGACCTCCTAGATCCTGATGGAAACTTACACCATGGAAACGCAAAGGAATTCGCCATGAAACACGGCGCTGAGGACGAAGTAGCTTCCAAGCTGATTACGCTAATCCACGAATGCGAAAAACAGCATACCGCTGTTGAAGACGAATGCTTGAGGAAGCTGGAAGTTGCCAAATGCTTCCGTTCTGGCATTCACCAACTAAACTGGGCACCGAATATGGATGTCATCGTTACTGAAGTACTTACTGAAATGTAACCTGGCATACATACCTTGATTTTTATATATAAACCTGTATGACCAATGCGACCTGTTTTAGCTCATCAGCATTTGTGATCTCAATCTAAAATAATTTTAATGCTATCATAGTACAGGCTCTGCCTAGTTT

>SinsGOBP1

GTTCTTCGTTACGACGTTGAAGACGACGTAGAAGTTCACATCAAGGCATCAGAGTGGCCTAACAAAAGGCGAGACTCTGCCTGTAGAACGCCTTCTCTCTAGATGGCGACTCCAGGAACAAAAACGATTGGATACACGGTCAGTTAGATACAATGTCAGACATTAGCACTATATAATAATGGCCAGTCATTCATTGTGGTCAATCAGTTATGCATATAACGCGCCAGGGAGCAGGTGGAACGATCCAGTCTCTGGACGTGTGTCAGGTCGAAAGAATGGAACGGAAAATGTTGAAGTTGATAGGAATATTATTATGCTCGCTGTTGGCGGGCTCGCTGGCAACCGTGGAAGTGATGAAGGATGTCACGCTCGGTTTCGGAGAAGCCTTGAAATATTGCAGAGAAGAAAGTCAATTGTCAGAGGAGCAGATGGAGGAGTTCTTCCACTTCTGGCGCGACGACTTCAAGTTTAATCTCCGGGAGATCGGCTGCGCCATCCGCTGCATGAGCCACCACTTCAACCTTCTCACCGACTCCCACCGCATGCATCACGACAACACCGACAAGTTCATCAAGTCCTTCCCCAATGGTGAGCTGTTGTCGCAAAAGATGGTGACACTGATCCACGAGTGTGAGAAGCAATTCGAGTCAGAGGAAGACCATTGTTGGCGGATACTTCGAATAGCTGAATGCTTCAAAACGGGCTGTAAGCAGCAGGGCATCGCCCCTACCATGGAGATGATCATGGCCGAGTTCATCATGGAGTCTGAAAGTCGATGAAAACGACCATTTTAAGACAGTTAGATGTTGACATATTATACCTGTGGCGCTTTTTATAGGTAAAATGATTTTTTAGTATAAGTTATCGCGAAGTCGTAACGAGTTCTGTAACGAACACTAAAGTATATTTAAACGTTATATTAATGATATATTTCGACACTGTAATGGATGTGGTTAATTGTTTTTTACAATGATTTATATTC

>SinsGOBP2

AGACATTAGAAAAACACCTTTTTCGGTTCTTTTTGGCTAAAACTTAAACGTTACTCTCTGATGCTTCTTTAAAAATAATTTCCTATATTATTTTATTATATCAAATATGTTTATATATATAGTAAGTATCTTATAGTATTTTCTTTTCCCCGCATATTCTTTATAGTTGTTATATGTACATATATTTTAACTTTTCATATATAGGCAATATTTTAACTTTTCAACTTTTATTTTATACACCCACCACAATTCATGTATGTATGTCATATAATAACTCAAAGGTTGGCCGAATGAAATCTAATAATGAGATAAGATTTCCTTTGTATACATAGATTTTTTATTATACTTTGTTTCTGTTTTTTTATTATTTATGTACAATAAATAACACACGCACAGAAAGAGTAAAAATAAATAAGTAAACATAATTTAATAAGTATGTCCTTGTTGATTTTCTTCTGATCAAACAAATTTATTGTCATACCAATATTAAGGTTACCATGCCAAACATCAAGTAATTATACAATATTAAATAATCATCAGATAAATTAGTGTTACTTATTTAATTATTGTACTGTTCCATGACTGCTTCTATCATAGTTACTTCTGGCGCTATTCCCTCTTTCTTTGCATCTACTTTGAAGCATGCTGCCACCTTCACCACCCGACTGCAGTCATCTTTTATATCATCATACTGCTTCTCGCAATTGTGGATCAATTCCACCATCTTCGCTGAGAGGATATCGCCTTTAGGGAAGCTTTTGATATAATCATCCATGTTCATGTGATGCATTCTAGTATCGTCTTTGAGCAGCGAGAATTTATTCGACATGCAAATAATAGCACATCCTAGCTCGCGGTGAACCACCTCGAAATCTTCGCTCCAGAAGTGCTTGAACTCCTCCATGATTTCTGAAGAGAGTCCTGACTCTTTCCGACATTCATCCAATGATTTCCCAAAATGTGCCGTAACATGGCTCATCACCTCAGCTGTTCCCATCACTTCATCAGTGATTGAAACAAAAATAACCAAGGAAAACAACAAACAGTAAACAGCTGCCATTTTGTCTTCAACTTAAGCAGAAACTATGCACGGTTGAGAAAATCACTTCATTATATCTAAGAATTTTTTTTTTTATTTACATGAATAACATGTTATTAT

**CSPs:**

>SinsCSP1

GTTAATATATAAGGATGGATACTGAAGCCATCATCATTCATTATTTTACAACTATAGCTGAAACATCTATAATCAGAATGAGGAGTCTTATCGTAATCTGCTTGCTTGCCTTGGTCGGCATTACTCTATCTAATCCAATAGAGACATACACAGATCGCTTTGACAACATAGATCTAGACGAAATTTTGGATAACAAGCGTTTGTTGATACCGTACATAAAGTGTATGTTGGACCAGGGAAAATGTTCACCGGACGGAAAGGAAATCAAAAAGAATGTAGTGGAAGCTCTTGAACATGATTGCCATAAATGTACTCCTACACAGAAGAAAGGCACAAAGAAAATAATCCGTCATCTGATCAATAAGGAGAATAACTACTGGATAGAGCTCAGCCATAAATATGATCCAGACCGTCACTTTGTAGGTAGATACGAAAATGAACTTACCGAACATTAATAATTTTTATCATGTGATATGTATAATATCTAAGTCAACAAAAAGCAATACAATAAATTACAAGTTACTACTAATAAGTTACAAGTTTGTAAAAATAAATGAATAGTTA

> SinsCSP2

CTGTTGTTGTGAGGAGTTGGGATTAATGGTTTATTTAATACTCGGACACTATACATATATATAAGAACGACAACCTCCTTCAGTTTACAATTCTCGGCCGCGGTCAGCTAAAAGGCACACGAATTAACTATTTATTCTATTTAAAAAGAAAATAAACTTAACAACAAAATGAAAACCTTCATTGTTGTCTGTTTGTTCGCTGTTGTTACTATCGCATCGGCTCGTCCTGATGACAAATATACTGACAAATATGATAACATCAGTCTTGATGAAATCCTCGCAAACCGTCGGCTACTGATTCCTTACATTAAGTGTATTCTAGAACAGGGCAAGTGCGCACCTGAAGGCAAGGAGCTCAAATCTCACATTCAAGAAGCTTTGGAAAACAATTGTGCTAAGTGTACCGAAACTCAACGTAACGGCACACGTCGCGTGATCGGTCACCTGATCAACAATGAGGAAGAATACTGGGGCCAACTTAAAGCCAAATATGACCCAACCAATAAGTACGCGACCAAGTATGAAGATGAACTCAGATCTGTCAAAGCTTAAAAAAATGCATCATTTAAACAAAAGACTGATCCAATATATTGGTGTATCATCAATAGATAATATAAAAATCATCTTTTTAAACATTTGATGTGAAAAACAAATTATTTCTTGTCCAATATATGTATTATGTAAAAAAAAAAAAA

> SinsCSP3

ACGCGCGTAAAAACGACTATTTAAAGATACTTTACAGATTAACGAAATTATGACTAAAGTTGAGAATAAATTTAGAAATTAGACTTTTGTTTAGTTACTATATAGATTTATTATAATAAATACGTCGGTATATTATATGTTTTTTAAATTTACAAGTTATTTGAAATGTTTATTAACATGTGCATATATTTTTCTTCTTGTGATTAAAAGATTAAATTCTTCAACTAATTATTTATTAATTGTGCAAAATTCTGCGATTGTCGATAACCGCCGTCTTAAGTTAACATTGAAAAAATAAAAAACTTGTTTTTAACGTTTTAATATTCCAAGAACATCGTCTCTCTCGTGTTTATGACGCAAATCAACGCAAACGTGTGAGATGCATGAGCAAGAATTACGGATAAAAAAATATTGCGTACCTACACTATTAATTTACTGGTGACATTTAAAACAAAAGAACAGTGTAGCGTTATTTATGTACATCGGCTGTTTATTGAACATAACAAGTACATAGTTTGTTTCACATTCCCCTATCGATCTTAATCAACCGACATTTTGTATTATAATTTATTTTTTAATCTTTAGCAATAAGAAATGCTTCATAAATTTCCTTGTACTCGTCGTCAGGATCATATTTGCTCTTCAACTCCTCCCAATATTTAGGTTCCTTATCTCTGATATGATTGACAACTAATCTGGCACCTTGCTTCTGTTTATCTGTACATTTTGAACACGAGGACTGTATAGCATCTTTTATGTGTGATTTCAGTTCTCTCCCTTCAGGCGTGCAAGACCCTTTATCCAGTATACATTTCATATAAGCATTGAACAAACGTTTGTTATCCAATACTTCTTGAACGTTTATATTGTCATATCTGTCCGTGTATTGTTCACCAATCGACAGACACACAAGGGTCAAACCTATTGTTAAAAATAACCAATTCATGTCGTACGTTTCCACCCTGTCCACCAGAATGCACTAAATGACACTGAGTATTTGTTGAAATGTGCCTTTTATATCTCAAATGCATCAATATCAATGATGATTTCCTTATTGAGGTAAACATCTTTGCTATTGTTATTGTAAAATTTTACGCAAGATACCAGTAGATATCCCATAGCATAATTAAGTCTCTTTGTTACAATTATAAGTGTCTAACGATTAATTTATTGACGCCTAACTTTACTAATAAACATATTTATTGTTCAACATATTCCATAGGTACATCTGCCAATTATTTATTTATTTATTCTTTAGCACAAAATAAATTATACATGGCGGAATTAATGCCATTCGGCATTCTCTACCAGTCAACCATTGGGTAAAATGAG

>SinsCSP4 ATGAAGGTCTTCATACTTTTTGAGATCGATGAATGATAACGGTGACGTCATGTCGTTTTGTGTAGGGGCTAATCGGTGGATATAACGAGTTTTGACGTTATTCTCAAATGTTCTATTTAGTATAAAAAGTCCGCGAGATCTGACCACTACATCACAAACAAACCTCGAGTCGTCCTGATAAGTATTTCCTTCTGTTATATCAAAAAGTATGAAGACCTTCATTGTCGTTTGTTTCTTCGCGCTGGTAGCTATTACCACTGCTCTCCCTGGCGCACGCTACACCGATAGATATGACAACATAGATCTCGACGAAATCCTTGGCAACCCTCGTCTGCTCAACCCTTATATACTATGCCTTCTTGAGCAGGGAAAGTGCACCGCTGAAGGCAAAGAACTCAAATCGCATATCCATGAAAGTCTCGAGAACTATTGTGAAAATTGTACTGAAGCACAGAGGAAAGGCGCGCGCCGTGTGATTGCTCATCTTATTAACAATGAACCTGAATCCTGGGAAAAACTCAACGCCAAATATGATCCTGAACATAAATACACCAAGAAGTACGAGGACGAACTCCGCACTATCACGCAATAAATGTTTGACTGAAATCTATAAAATAAACTATTATAGTTCATATCAAAATTTGGAGAAAACACTCATACGCTATTATATAGAACAGTGCCATTGTTAAGTTAGAAAACAAAGAGATTTAATTATATGATAACAAGGCTTGTGAATTTTATAAGACACATTATTATGTATATAAAGAAAGAAATTAAATAACATAAACCTAACTAAGATATGTTTTATTATAACTTAGCTTATTTTAGTATTAACCTTCCGTCAGCCTGGTGGAGTCTCAAATACCCAATACAGTTTTAAACAATTGTTTTCACATTAAATTAGTACGCTAACGATTTGAAATCTGAAATAGCTGGCATTAATCGATTGCGTTATTGATCTCCAACACTTTTACTGGACCGCCCAAACTCCACCAGGCTCACCACGTTGTAAAATTTCAAGTATTATTTTAACGCTATATTGGTTAGTTTTAAAGAAGAATGGCGAAATCTATGTATTTTAAGACCCAAGCTTAGAAGTCTA

> SinsCSP5

TCTATAAATCTGGAAAAAAATACTTAACAATAATTTGTTAACATCTTAATAGATGCCATAAAATTGACTACGAATTACAATTTAATTCGTTGGGAAATAAATAATAAGGTCCTTGCTACTTTAATTAACAAAGTATCACAATATTTTTAAGTTATTCTAAAGTCTGTAGTAAAACCATCTGATCTGATGTTCCAAAAGTTTAGAAAAAAAAACGATATTTTTTTAACTTAACTAAACTATAATTTATTAACTACATTTGTCGTTAATTCTAGTTAAATTAACTCGATTGGATGAATTTGTCGAAATCAGCGCGATGCTTGCCTGAAGGATCATTTTTCTGGACAAGCTCTTCCCATTGTTTCGGATGCCGTTGCTGAATGGCCTTTATTACCTTCTTTATGTTCACCTTTTGCTTTTCGGTACATTTACCACAAACGGTCTCCACAGCTTCTGGTAGTGTTTTTTTGAAATCGGAACCTTCAGGTGTACACCGCCCTTTGTCAAGGAAGCAATTGATGTAAGACTTGAGGAGCCTATCATTGGAGATCAATGTGTCCACATCGAAATTATCATATTTCGAGTGATATTTTTCAGCAGCATATGCAGCGACGCCCAAACAAGCCAAAACAATCAGGACCTTCATTCTGTAGACAAATCTTTTACTCTCTATAGCAGACTCAAAGAACTAGTGTGGCAGAAAGCGACAGTGCTTTTATATTGACGACTATAAACCCACTACCTTTATCGTCACATGGGCCAACCACTGCACCAGACAAACATTCGATTTTAAACCTTTTACTAAAATACATAGGAACTCCATAAAGTATACCAAATATAATTTTTGGACCCTTTGTGAATTATTAGTGAAATGTTGTTAGCCGTCTCTTATGGACTATAGTTGAAAACATATTTACATGGATT

> SinsCSP6

TTGAAGTCAGCGCCTTCTGGAGTACAGTATTGTTAGAGCTGAGAAAGAAGGAAGTTCTAAAAAATTCACTATGAAGACGATCATAGCTTTTTGCGCATTGGTAGTTGTGGTGGTCGCCTTCCCCGGGGACACCTACGATCCTCGGTACGACAATTTCAATGCGCAAGAATTAGCAGACAACCTTCGTCTGCTCAAGAGTTATGGTAAATGTTTCCTCGATCAGGGACCCTGCACTCCAGAAGGCGCTGACTTCAAAAAATCCATCCCCGAGGCCCTCAGAACCACTTGCGCTAAGTGCACTCCTAAACAGCGCGAATTGATCAGAATCGTTGTCCGCGCCTTCCAAACCAAGTTGCCTGATGTTTGGGAACAAATAACCAAGAAGGAAGATCCTAATGGAGAATACAAAGAATCTTTCGAGGCCTTCCTGAACCGCTCTGACTAAGAATATAAAGCCAAAGTTAAATAAACAATGCCATCTTGATCGTCGTATGCTTTATGACTAATTTCAATAAGTTTTAGTTTAATTTCGATGTATACATGGCTGCGAATAGTAAATACATCACATTAATTATAATTAGAACATAAGTTTAAAAAAACTGTGATGTTGATTTAAAAACACATTTTTATGAATATACCGTTTAATAAACAGAAATGATAAAATTAAAAAAAAAAAAA

> SinsCSP7 TTCCACTATAAAACAATTTATTAACTTAAAACTATTACAATAAATTAGGAATACAAATTATTTGCAAATAAATTATGATCAAATTCACCCGAAAGCCATGGATTTATAAAATTAAATGGCTTCTAGGCCAGTATAGGATTTTACCAACGTCTCCGGCGGCATATATTATAATAAAGTTGATGAGTTTAATTGCCAGTGACTAAGAAATTGTATAAGGCATCCCTATTGGAGCGATCTGGATCGTATTTGTCTAGCAGCTCGAGGAATCGCGGTTCGCTTTTTGCCCTGATGCCTAGCAACATTTTGCGGACTACGAACTTCTGTTTTGAAGAGCAACGACTGCATGCGGTTGTCAACGTCTCTGGTAATACGCGTTTAAATATCTTCCCATCCTTTGTACAGGGTCCTTTGTCCATGACGCACTTGTAATAGGCAAGAAGGATGCGTTCGTTTTGTATGATAGAGTCAGTATTGAAGTTGTCATAACGGTTATAATGCTGCTGTTGGCCGGAACAGGAGACCACCACCGTCAGCGCGCATAAACACAACAGCCAATTCCTCATTTTGT

> SinsCSP8

CTGTTGTTGTGAGGAGTTGGGATTAATGGTTTATTTAATACTCGGACACTATACATATATATAAGAACGACAACCTCCTTCAGTTTACAATTCTCGGCCGCGGTCAGCTAAAAGGCACACGAATTAACTATTTATTCTATTTAAAAAGAAAATAAACTTAACAACAAAATGAAAACCTTCATTGTTGTCTGTTTGTTCGCTGTTGTTACTATCGCATCGGCTCGTCCTGATGACAAATATACTGACAAATATGATAACATCAGTCTTGATGAAATCCTCGCAAACCGTCGGCTACTGATTCCTTACATTAAGTGTATTCTAGAACAGGGCAAGTGCGCACCTGAAGGCAAGGAGCTCAAATCTCACATTCAAGAAGCTTTGGAAAACAATTGTGCTAAGTGTACCGAAACTCAACGTAACGGCACACGTCGCGTGATCGGTCACCTGATCAACAATGAGGAAGAATACTGGGGCCAACTTAAAGCCAAATATGACCCAACCAATAAGTACGCGACCAAGTATGAAGATGAACTCAGATCTGTCAAAGCTTAAAAAAATGCATCATTTAAACAAAAGACTGATCCAATATATTGGTGTATCATCAATAGATAATATAAAAATCATCTTTTTAAACATTTGATGTGAAAAACAAATTATTTCTTGTCCAATATATGTATTATGTAAAAAAAAAAAAA

> SinsCSP9

TGGTTGTAGCGACGACGACTCGTAGCTTATTCAGTGATAACACTAGAAGTGTTGCAAGTGCGTAATTATATGTAAACATATTACATACAAAAATAGGGTACACTGAAAAAGCTACGATGAAGTCTGCAATAGCTTTGGCCGTGCTGGTGCTGGTTGTAGCGACGACGACTCGTGCCGATGAGGATTTTTACGACAGCAAGTACGACGAATATGATTTGGATGCCATTATTTCCAATAAGCGGTTGTTGGAGAACTACATAAATTGTTTCCTTGGAAAGGGCAAATGTACTCCCGATGGTGCTAAGTTTAAAAAGATCCTTCCTGAGGCCCTTGAATCTACGTGTGGCAGGTGTACACCGAAGCAAGGAATCTTGGTTCGCAAAGGCATTAGGGCAATACAGGAGCAATTGCCGGAATCGTGGGCACAATTGGTAAAGACGTACGACCCGGAGGGAAAGTATAGGAACTCATTTGAGAAGTTCCTTGCGAACACCGATTGAAATAGAATTTAAATCCAACATTCGCTAATTTTGCCAAATACATAATAAGTTAGCAAATACAATAGGGTATTTTATTCTTGTTTGTAAGGAATATTTAATTGATTTATTTTCATTTTATGAAATCGTTTAGTTGACGTAGACAAATAAAATTTCAATTTTCTATTTCCGCACGCAAAACATTAGCCACCATGTCTTCCTGTCTTGTGAAGTCCTGATAAACGTAAGTCCCGGCACACGACGCGACGCTTCGCTTTGTTTTGTATTGAATTTATACATTTTGTTGAAATAATAATGACTTACAATATTACAATTAACGATAGTTAATGCGCTATACAATAATGTAAGAATTTATTGTATTAAGTTGTTAGACGTTTAACGTTTACACAGTCAGTAACGTCACGGCTGAAACATTATGGCGGCTATTGTTTACTTTGATTCTGATATTAGTAAAAGATTAAAAAAAAATTTTTTTTTTTGCATATTTCCCGAGTATATAATTTAGTGTTTGTACGACTTTTGTAGCCGGTACAATCGATATCTGCTGTGTATAATAATAGTAATTTGTAAATAAATATGTGCGTAGTTTTTAGTACTTAGGTATTAAACTCACTATTTTCCAAA

> SinsCSP10

AGCTAAAATTTTAAAATTTCAAACGTAAACATTTTAAAAATTTGAAACTTAAAAATGTAAATAATTACAGTCGAATTTCGACCGTCTGTAGGATTAATTACGATCGAATTTCGATCGTTTGCTGGACTTCTAGTATAAATTAATAGGTGAACCAATAGAGAATTCTAGGAGCAGATATACTCTTCCGGGTGTCAAAGTTGGAGTAAAAAGATATCAGATGGAAAAAATATAAATAGGTATAACTCGTAAGTTATCAAATGATAACACATTTATCAAATTACACTGACAACTAATTTATTTATATTAAATATAAATTTTATGTAAGTACCGTTTATTTCATTGCCTAGTTATTAAGCATCCTGTAAAATATATTTATTATAAAGTCACCTGATTTTTTATTTTTTTCTTAAATAGACAATGTGGTAAATCATAATATGCACTTTATGCTTTATATCTAAATATATGTACATACAGAGTCTAACCTCCACCCGAAATTATATTATGAATTAAAAAAAAATTTAAAAACATATTACTACCAATATAAAGTGATTTAGTAGTATTTTAATACTTACTGATTTAGTAGTTTCTGCATAAGAAATTAATTTTTAAAGAATACATTTTTTTATTCTCTTACATCACTGTCACTCTCCCAACGTTTGAGGTTCTAAAACTTCAGTTTGAGGTTTTTTATCGCTTATTGCAAAAAATATTTTAGCTTAGCGAATAATCGAAGTGACAATTTACTTACATTTTACTACAACCTGACACATGGAAACATCTTAATTTTACATGTAAATAATCTAAGGCATACATATAACATGTGTGTCTGTGAATTTTTTATTCACCATTTATTAAAAGGTCTTGTAAGGATTTAGTTGAATTTCGCATACGAATAAAAGTTATTTGTAGAAACCAAAAAATTTTAATAGTGGAGAAATATGATACTAGTAGGTATATGATAAAAGTTTAAAAATAAAATAAGTATAAAGTTCTATGTAACCCACAACTTTATTAGCGACTTCCTTATTCCGTATCTAAATCTAACGTGGTCCTTGCATTTATTATGCATTTTAACTTTTATAATCCCTTTATTTATTCTTCTTTTTCATCTGATTCTTTATTATCATTAATACCCTTTTCAGCGTTATGTGTAGATTTGATCTCGGAAGACGTATTATTTTCTGCTGTTTTACTTTCTAAATATTTCCTTTTGTAACTTCCATCAGAATTGTATTTCTCTTCAAGTTCTTTCCAATCCTCTGTTCTATGGTCAATAATATAGTGCATCACCTGATCGGCACCTTCACGTTGTCTTGTATTACATTTTTGACAGTCGTTAGTTATCGCGTCAGGCAAAGTTCTTTTCAATTCTTTGCCGTCGGGAGTGCAAGGTCCTTTATCTAGCAAACAATTTATGTAACCAGTCAACAAACGATCACTAGCCAAGATTTCATCGAGATTCACGTTGTCATATTTGGTTGTATACGTTTGTGTTTCTTCAGCATTATAATGTTTCATTTGGTAAACAAGCAACAGATATATTAGCTGAGTTTTCATATTCATTTTCACCCACTTTTAATTGTCGTATCATAAATTAGTGGTTTGTTTCTCTTTAGTCAATAAAACTTACCTATAATTTCTTTGCGTTTAGATATAATGCAATGTTTTAACGAAATCATATCTTTTATATGGACGCAAATGTTACTT

> SinsCSP11

GTTAGAAGTTAATCTAGTGTTGATTTGTGAAACTATTGTATTCGCATTTTAAAATGAAACTCTTTCTGATCGCTGTAGCTCTTTCCATGTTCTGCATATTGGTTAATGCACAAACGTATACTGATAAATATGACACTATCAACCTGGACGAAGTGCTTTCCAACAAGAGACTGCTGGGCGCCTACATCAAATGTGTGCTGGAACAAGGACGTTGTACTCCCGAAGGGAAAGAATTGAAATCTCACATAACAGACGCTCTTCAGACAGGCTGTTCGAAATGCACGTCCAAGCAACGCCAGGGCATGAAACGAGTCATCAAACACCTCATTACTTACGAAGATGATTCATGGCAACTGCTGGTGGAGAAATACGACCCTCAAAGGATTTACTCCCATAAATACGAAAAGGAACTTAATTCTTTGTAGAGAAACCAGTGCGGAATTTTGTATAAGGGCACTTAATCATATCCACTGTAAGCTTTTCGTATAATAAGGCAGTTTCTATGAAAAACAGAAAAACATCCTAGGTATCACTGTCGAAATTGGAATTTTGTAAATTTAGGAATTTGGTGAATTTTGTTATATATTTAGATACTTAGATAGCAAGAGTGCCACGTAAGTAAAATAAGTAGACTTTCTGAAATTTCGATTTTTCTAGTACCTACGAGATTTCCAATTTGTCTCGTGTGGAAATCGAACCCACGACCTCTGTGTCTACCATGAGTTTATTCGCTAAAGTAATGACTTTACCCGCTAGCGACTCCGTAAAGTATTTGTATGAGAAATTG

> SinsCSP12

AAAGCGTCTCGACTCGCCGCACGCGCAACTATGTATATGCAACCATATAACTGCGCGGTTTGTCTCGCTCGAGCCATATCAACACGCAAACTGGAATCGCGCGCACGAATGCCTTTGATACCAACAGCATAATTGCGCTGGAGTTCACAATCAAGGTGCAGTCAAGATATGTGAATGCGATTTTTATAAAAAGAACAGGGTTTTCTGTAAAGTGAACATTATCAATATGCGTGCTGTGTTTTTCTTGTGTATTAGTGCGTGTGCCATTGTTGTTGGCCAGGACATAAATACTATGCAAAATATGCCTACATACGATGCCAGATATGATTATTTAGATGTAGATGCTATACTGGACAGTAAGAGATTAGTTACTAACTATGTGAATTGCCTAATCAATGAGAAACCGTGCACTCCCGAGGGGAAAGCGCTTAAACGATTACTCCCGGAGGCTTTGAGAACAAAATGCGTCCGGTGCTCAGAGCGACAAAAGAGGACCGCTGTCAAAGTAATCAGACGCCTAAAGAATGAATATCCAGATGAATGGGCCAAGGTTGCTTCCCGGTGGGACCCCACCGGAGATTTCTCCAGATACTTTGAAGAATATACGGGCAGAGATCAAACTAATACCATATTTAATACCGGTAATGAGGTTCCAGCGTCGTCTCCACCGCCGCCACTGCCGTTGAACGTGCCACTTAGGACTACCGCTCCTGTCGCTCCAGCTCAACCGACGAGTCCACAAGTTCCAATAGCAAGGTCTACATCAGCACCACCTATTATACTTAATAGGTTCGGTGACGACGGCGTGTTGATAGTTAGCAGCTCATCTCCATCGAACACAGCGTCTACAGTGACGCCACGATCATCTAACACTATTAGATCTGTCATCCAAACTAGGGCGACACCAGCTACGTGGGCCGGCGCAGCATCAGACATCATACCGACACAGGTTCCAGTACGTCCAGCGAACGAAATTCCTCCTTCATACTCTACAGCCATGACCATCATTGACGAGATTGGACACAAAATCATTCGAACCACTGAACTTGTCTCTGATATACTTAGGAATACGGTGCGAGCTGTTGTAGGTTGATCACACGCATCGTATAAAATCAAAGTTGAATCAAAGTTGCTCAGTTTTATCGCAAATATCCAACCTAAGTTTTTTTGAGGAAAATATGAGATTTATTACCTATATTTAATTATAATTTAGTGGCAACAATACCACCGATGTTTTGGATATTATATTGGAAAAAAATGCTCATTTTACGATAATATTGGAGGAATTGAATAATTAATTATTCTAAAAGTTTAAAAAGAAACTGACATCCGCCGTTCATGAGATTACTATAATGTTTAGGATACGTATACCTACATTTGGAATTGAAATTAATAGTTTTGTACTTAATTTTAATTTATATACATATTTACTAAACATTATCTCGAAATCAACGCTTTATATAATTTATTTTTATTTTATAAAATATCTAATGCAATATAATAATTAGTATCGATCAGGAATAACGAGATTAATAAGTATATATAGTAATTCTTGGTAAGATGTCGCGACAAGTTCAGTTTAGTAGGTATCCTTATCAAGCCCCTTAGTGTAGCTCCCAATGTGTATACGGAAACCATTTAGGGGACGGTGACAAGCTGGATTGCATCTGAGCTGCCGCTCCTTAAATCGGAGGACCTGTGCATACTTTTGATTTATTTATTATACTTTATTGCATGTTCAAAAGGTGCATTTAATGCTATGTTATATTTTCTACCGGCTAACTACAGAGTGTTTGAGCAAAAAGCAGAGTGGGCGTGCAATTCACGTATTTATAATAAGAAAAAAAAGGAAAATATAAATATATACTTATCATTTATAGTAGGATTAAATACTTTAAAGGTGATAGTTAATGTTGATTTTGTATGTTAGTGGTACTTTGAAAACTTTATGTAATGCTAACAATAAGTGGGCGGTGAGCGCCATAAAGTTGAGAAACTCTGTCTTAAAGTTTCATCTAAGGAGCTCTTCAAGGACAAGAATCTCTTTCGTTATGTGTCGTTAAGCCAGTAAAACTCTTATTATCCATATGAATTTCTTATATTGGGTGGAAAAAACGAACGGGACATGGAAGGAAATTGACAGTCTAGGTTGGGATGTCAGTTAGAGTCGACATGCACGAATTGTTAGATTTCATGGTCTGTTCGTTTTTTCCACCCTGTATAATTATGTGGAAGCCTATGACAGACGGATGGACTCGCAAACCAAAATTATATATGTTCTATAGTGGACTATGGGGCCCTAATAATTGTAATTTGTAATATAATTAAATAAGGAATCATTGCATCGAATGTAATTATTGTAAGTATAATATTTTTGAGAGAAATTTCGCTTCTCATGCATTCCTTGTGCCTTATTTTATAATGACAGTTTCGATTGTCGATTACCTTTACACACTGTAAATATTAGATTAATATTACTTCCTTGTTTCTGTTTAAACATAATTAGGTCATAGTAATACATTATTTTTTTACATATAAATATGAATAGTATTAACAAGTTTATTATTAGTAACGACGTATATAATATGTATGTAAGTAGTCCTTTTGGTAACAAATAGAACTTGTAAAATGAAAGTATTATATATCTATCTACCTAGCAACCTACCAAACAACGAATCTAACAACGATTTCCACTTTAGTAAAGTATAAGATAATATTACTTACCTATGTGGGTTATTTCTTGTCTGAAATAAATTACTATTATCTTTATATACACGTATACAATTCTTCTCTA

**ORs:**

>SinsOR1

AGGTATATCATTATGTTCGCTTATAATTCAATAAGCGTACTGTTGGTAGGACTGGTTGATGGCGCAGGAAACTTCCTCGTAGCAGGACTACTGGTGCTTATTAGCGCTCAACTGGACGTCCTCAGGAGAGAACTGAACGAGATAAGCGCAAACCGAAGTGACGAAAACGACTTAAACTATCAAAAGACGGTGTCGTGCGTAAAATATCATCAGCATATCATTGAATATGTTGAGGAATTAGCAGCTAT

>SinsOR2

AGACAATAATGTATCGATGCTTATATATTGTTAACTTTCTTTGGTTAAACACTGATGTATTGGGTGAGGTGCTCTGGTTTGTAGATTCGGTGAAAAAAGGCGAGGGCTTTACAGGAGTGGCTTACATTGCTCCATGTTTCACCATAAGCATGCTTGGTAATTTCAAATCTATCTTTCACATCTTAAACGAAGATAAAGTCAGTCAACTTGTTGAAGATTTAAGAGAATTAGAAAGAAAGGAAACAGATCGCAATAAA

>SinsOR3

AGAAGCTTCACAACTTCAATACCATTCAAATATAGACGAAAAAAAATGAAACCAAAAAATATTGTATTGAAACCTCCAGAAAAGAAATTTTACACATTTAGCGAAACATTTCGTTTCTGCGCTTTCGCATTAGCAATTGCTTTGATATATCCTAACAAAAATAATATTATACGAAGATGGATTACAGTTATTTCAGTAATTTTCTTCAACTCTGTCATATTATTCTGGTTTGTGTCATACTTGATCAAATGTCTGATCAGTGTGGATATATACAACTTTGCAAGGACCGTTACAGTAGGAGTCGTTGTCGTACTTTTCTTATTCAAATCGTTTTATGTGAATTGGAAGAATGAAGAATTTGAAAAATTGTTAAATAAAATATCCAAGGATTTGCTTAAAGGAAACTACATGGATGAGGACTATCAAAGAATATACGAGTACCATATAAAACAGGCCAAAATTCCACAAATATGCTGGCTAATCATACCTACAATATTAAGTCTTCAATTCCCTCTTTACGCCAGTACAGGTTTGATCTATGAGACTCTAAATAGTGATGTTGGTAAGAAGTATATGGTCTTTGAAATGCAACTTAAGTATATTGAAGACAAACAATATGTGTCACCTTATTATGAAATTATATTTGCATACAGTTTAGTACCGTGCTTAATCCTAGTGCCTAATTTTGCTGGTTTCGATAGTTCTTTTTGTATAGCGACAACGCATATGCGTCTCAAACTTAAATTAATGACGCACAAAGTTCATAGAGCCTTCAAAGATGCAAGAAATCGTTCAGATCTACAGATGAAGGTGAAAGAAGCGATAAAAGATCACCAAGAAGCTTTAGAATTCCATAGAGGCATACAAGAAGTGTACGGTGGTTGGCTACTGGCTGTTTTTCTTTTAACATCTTTCTTAATTTCATTCAACATATATCAAATATATATAAGCAAGCGTATCGATCCGAAATATGCGATTTTTACTTTGAATGGTGTACTGCATATGTATATGCCTTGTTATTTTGCCAGCAGTCTAATTAAGGTGAACGAGGAGTTGTCGACAGATCTATACAATGCATCGTGGGAAGATTGGGCCGATCCAGCCGTCACCAAATTACTTGTTTTTATGATGGCCAAATCTCAACAACGTCTCGTGATCACGGGAAAGAGAATTGTCGTATATAATATGGATCTTTTTATTTCAATATTGCATATGTCATACTCTTTTTTCACTTTGATTACTGCAAAATAGATTTGAATCAAACGTGACTAAATATACAAATAGTTTTGTCTTTCGCATTTTAATGTAATTGTTAAAAAATATATGAATATATATGATGGTATCTGCAA

>SinsOR4

ATTTTGTAAAAATTGATTATTTTGCGGAGAAATAGAATAAATATAATTTTATTTGTATTAGATAAATTTAGTTTAATGCCTCACTTAAAAATTAAAAAGAAAAATTTTAATTAAAAAAAAAGAAATATACAACAATGAATAACTACTTAGATTCTACGGCAATTTTACTTTTTCCGTAAAATGTTTCATATTTCAATTATTATTGTCGTCTTCTAAAACAGTCATCACCAACATGAAGAATGACATGATGCTTCGTATGAACCAAGTAAAAGTGGGCAAAGTCAACGTAACAGATCCAAAAATATAAACGGGCATGCCATGACTGACGGCCATCTTGAAAATTTGAACACAAACCTTGTGTTTTTTATCGAAAATATACCACTTTTCAAGAACAACATTTTCTAAAATGTCACATTCATTTTTCAACTGCTGGCCAAGAAAACAAAACAGCATTACTATGAAACCAATACAAAATGCGTGAATATAATATTTTAATGTATCTTCGGAAAATCTATCTGATATAGAGATTTTCACAAAACAGACACAAACCAACATAATCGCATCCAATATAAGACCCAAATAAAATGGTTGACATATGATATTTAGACGATTGTTAAATCTTTTCAATTTTATATAATGGTCGATGCACTGTGACAGTATGAGAGTACTGTGCTTTTGCCATCTTTCATCTCTTTTCCCCGTCAATTCAAGTACTAACTTGTCTAGATTCTGAAGTATGAAGATTAGTACTTTGAGTTGGCGTATCATCTGACCAAACAAAAGAAATATAGTTGATTGTATACCGCAAAATGACAAGCAACATATGTAACCGCCATATAGATTAAATAAAAACGATGCCACGTATACATAGTCATTTTTCAAACCCCATGGAAACCACATTTCGAATGCCATTATACGGACATCCTTATTTTCTCTTCCAATAAATGTACGATAAATGGGTTTGCATGTATACAAAACGTAGCATACAGTCGAACTCCCTAAATAACTTCTTATCAATATGTCCGATAACTTAAACAATTTGTCATGAGAATAAATAACTAATTGTTTTAATTCATTCAATTTCCCACCGAGAGTTAACTTCAAAATTTGTTCAATAAAAGGCATATTCAACATTGGATCTTGAAATACAATCTCTGTTAAAAACGTTCTAAATTTCTTTCGATAAACAAAAAATAATGTGATAGATATGAACATCAAAATTCCACTATTCCAAACGCAGGCGGCTTGAATACATATCAATAAATCTGAATAGTTTTGAATCATAAATACTGTGCAGAAAATAAGGACCAAAAATCCATATATAAACATAAATGTTTGTATTATTAGCCAAGGTATCGAATCCCATTTTTCTCTAGCAAATATCGAAATGCCAATCACTGAAAGAATTTTGTGGTACACGCTCAATGTTACATCCATGATCGCAATCGTCAAG

>SinsOR5

ATTTATCGACAACTTATAGAATTCAATTGAATTATCGATCTCAAACGTTAATGAGGCAAGTCGATGAGAACATTCAAGACGTTAAAGAAACCCTACCTAAGTTAATAAATCGTCATATAATGATACTGGATACAATTGATAAACTAAAGACATTATATAGCGTTCCAACAGGTGTAGACTTTGGCTCAAATGCCGTTTGCATGAGTTTATTCTGTTTTTTGAGTTTACAAGAATACGTCACATTCATGACAATTATACTATATGCTTTTGCTGTGTTCTTTCTCTATTGCTATCTATGCCAGTGTCTTATAAATGCTTCGGGAGATTTCGAAAGGGCAGTATATAGTTGTGGATGGGAAAATCTGGAATGGAGAGATAAGAAGACAATATATGTGATGTTATTGCAAGCCCAAAAGCCGATTGAATTGTTAGCGGCAAATATAATACCAATTAATATTGCAACATTTGCCAGCACTATCCAAGCGATATATAAATTTGTAACTGTTGTGAAATTTTAAACGATTTATATCGTGAAATAAAACTAGCACTAAACAAGCAAACCTCGACTAAGACCTATCACTGCTTTGTGTAAATAATTAGAATACACTGACTGAGGGTGTAAGTTATGC

>SinsOR6

AATTTTCGAAATGAAGCCGTCTCTGAAAATTGATTTAATTCAGTTCATCAGATTACCAATAAAATCCCTGGAGTTCTTTGGTTTATGGGTATTTATACCGTATCCAATAACATCTATCGGATTTTGGATCGGCGTTTTAGTTCGTTTCATTGTAGGAATTTTGATTTTAATTATACCAACTGGTACGCAGTTCTTGTATTTGGTTACTTTAATAATGTCAGGAAATGCTGAAATAAGTGAAATTGCCGGAATTATAAATTTAGTTCTCACCGAACTGCTGACATCGGTGAGGCTCTTGGATTTACGCTTACGCCGGAAGTCACTTACGCAGCTGATGGATCAGCTCGTTAACATAGAATCTCATTGTTTTGTTGATGCCCATAAAGAGATATTGGAAACAGCTATGAAGAAATCAAGGAAACTGTACCTGTGGTTGCTTTTCTTGACGATTTTTGATATTACGGTGCATGTCATCATAGTGCCTGCTCTGCAAGGTTTTCAAACATTGCCTCTGAAGATGGATTTTATAATTTTTGATGTAAATGATGAAAGTTATTTTAAATACATTTGCGCTTATCAGATTCTCTATAAGCCGGCAATGCTAACCACATTCGTTGCTTTACTATCATTATTGTGGTCGTTTATGATGAGCATAATCAGCCAATTGGATGTTTTAATTTACAACTTTGAGAACATGAATGGGCTTGTTGAGGCGATGAAGGCAGAGAGGCTCTGTGATGAAAACGAAGCTTTTAAGGAAATTTTTAAAAGATGCGTCCTTCATCATCAGGCTATTATAAGATACTTAACAACGTTTCAAAATGCTTTCGGAGGGCAAATGTCAACCACTTTGATTTTGAGTGCGACCATTATAGGTACTACTGCCCTGCAAATTTTATCTATAGAATCACCTACAAAAAACATTACGGTAATAATTTGGGTTTTACTTTTTCTGTTCCTTACTGTGTTTATACTTTTCGGCGATTGTTATTATGGAGATATCATTAGGGTTAAAAGCTCACAGTTAGCAACGGCGGCTTTTGCATGTCCCTGGCTAAATAAAAGGAATGAGTTGAAAAAGAATCTCTTAATTTTTATAGCCAAATGTCAGCAACCATTAATTGTGATGACACCAATATTAGTGCCAGTGACTATACAAACTTTTACAATGGTAATGAATTGGACATACAAAGGTTTTGCGGTGTTGAATCAGATGAAGAAATAACTCAATAATTTATTATTTTATTGTAAAGCATAGACTAGGGAAATATAGAATAG

>SinsOR7

TGAAGTTGTTCTGACATAATAGAGTCAAATTATATCAAAGGCTTAAATAAAAATTGTTTAGTTTGGAACAGATTCCACGTCTTTGGTAGCGGGTAATTTTTGAGTAAAGACAAACAAAATGGAAAATACAAAGAAAGTCCAACCATTTGATGCACTTTCCGTGTCATATAAGATATTGATATTATGTGGATTTTTCAAACTTATGCGCCCTACAACACGTTTCAAATATATCTGCTTTCAAATATATAGGCCATTGTCTTTCTTGATTGTTCTGGTATTCATCGTGCAGCATTCAATTTATGCTGTTATGAAAGTGATGGATAATGAACTTGATAAAGCGTTAGATGCGTTTATTGTAATTCCGCCTGAATTAAATCTGTTAAGTAAGTTTCTTGCTTTGAATTTGCACAGTTCTACAGTGGATAAACTTAATGATGTTATGCGTGATTCTATATTTGACGCAAGGAATCAAGAAGATGAAATAATTCTTACTAAGTTTGTGTCTGATATGCACCAACTAACCAAAAACGTGGAGATCGGTATGGTCGTTGCTGAAATTCTTTACATACTGTCGCCCATTTTCAAAAGAATATACGATCCAGCTAGTACAATAGCTTCATACTATCCTTTTAAAGTGGATAATTGGGGAAGGCATACAATTACTTTATTATGGGAATGTTCATTTTTACCATGGATAGGAAACGGACACTTATCGTTAGACTGTCTCATTGGTATATACTATTCTCAAGCAACCACTCAATTGAAACTTATAAAATATAATTTAGAACATCTTTTCGATTCAGACGAAAGGAATAAAGATAACAGCCGGAATATGGAACATCAATATATCGATGTTGTAGATAACACTATACAGGAACGTTTCGAGCATTATGTTGAACGTTATTCAAAAGTCAAATGGTATATACAAGAACTGCATAACGTGTTCAGCGGTGCAATTATTTACCAATTCGCTTCAACTATTATTATTGCGTGCCCAGTAATCTACAAAATTAGCTTTATGGATTTTTTCTCCGTTCAATGTATATATCTAACAGGATATTTAATTATGTTAGAAATTCAAATCGTACTATATTGTTATTATGGAGGCCTAGTCGAATACGAGAGCATCTCGATTAATGATTCTCTTTACATGAGCGAGTGGTTGTCGGCATCTCCTAAATTCCGTCGCCAGATGTTGATCGCCATGGCGCAGTGGTCGCGTCCGCTCACACCACGCGTCTTTGCCATAGTACCACTCTCTCTTAACACTGTTATTGCGATGCTTAAATTTATTTACTCTTTATACACAGCACTAATAAGTACCAACAACATAAATTAGTGAGAAAACACATGCATATAAGTAGTCACTGGTACGTATGTATGTATATTCTTAAATTCTTAACTAAACGAATATAGAGTAGCATTATATAAGTAGTAGAGTTAAACATTGCTTATTTTTTAAATTAAATGCTACGTACTCAAAAAAAA

>SinsOR8

ATTTATTATGAATTAACATTGAAAATTGTTCACATTCTATCATTGTCGTTGTTAAAAGAATCTCTATTTTTGTGTCATCTTTCTTTTTAAATATTTTTTAAAATAAAGTAGATATTGTAAAATGAAGTACGTGGGATGTTTTCGAATACATTTTACAATATTAGGCATAGCCGGTATTTGGATGCCGAGATCTTTTGAAAATAACGTCAGATTGAAATTTTATTATAGTGTTTACAGAATATTTTTTTTGACCTTATTTTTGGTCGGCATTGTTTATACACAATTCAGGTTTTTCATCCTGGTGATCGGTGACATCGAGAAAACAGTCGATTCAAGTGTGTTGTTTTTCACAATTTTACCACATATAATCAAAATCTACACGCTCATATCCTGTCGGGAGAGAATCATTCGTCTTTTGGACATAATAGAAAGAATTCCAGATAAAAATAATATATTACAAATGTTCTCCAAAAATGTAGCCCTAATATCAAGTGCATACTTCTGCACCTGTATAGGTACAGCTATATTATGGTGCGTTTATCCTTTCATGAAGCCAGTTTTGACATTACCTTTTTACTATCCTTACATCTCTCAGGAATCTTTCTTGTTTCCAGTTTTATATGTTTATCAAACGTTTGGAATTATTATCAGTGCTCTTACGATAGCTAGCGAGGATTTTCTAGCTGGAGGGCTAATGGCGTTAGCTGCAGCACAATTGGAATTGCTGTGTTGTGATCTATCCACCATTGGAGAAAACAAAGAGAATATAAATGGGAACATTGATCAATATTATGAAAAAATTGTAACATGTATCAAATTTCACGCCAAAATCATAAGTTTTGTAAAAGAATTGTCAACCATATATGGACTTTCTGTGTTTGGGCAGTTTCTATTTAGTGGCATACTATTGTGTGAATCAGCTTTTATAATTATAACGAGCGATGTTTTTACTGAATCTGTAACGATGTTTTTATATTTACTATGTCTTTTGGGACAGCTACTGCTATATTGTTTTTGTGGAAATATGATAAAAACTAACAGTGATAAGGTAGCAGCGGCGGCTTATAGCAGCAGATGGGAGATGACCTCGTTGCCGACTCAGAAGGCGTTGTTATTTCTCATCATGAGAAGCCAAATGACACTCACCGTTACCCCAGGAGGAATATTTGACTTGTCTCTAGTTACATTTTCTGCTGTTTTAAAATCTTCATATTCGTTTTTGGCAGTACTTAACCAGAAGCACAATTCTTAAAACGAAGTATGTATAAGTAGAGAATTAGATGACGAGCACATACATCATATGTATTTAGGTAATGTGTTAGAATAGAAATAGCGATAAGCGATATCTTCACGGTTAAAGCAATGCTTAAAAAATAAACTGATTATGAACACACGGCCTATGTTTGTATATTGCAAGTTACCCGTTAAAAGGTAAGTACCTACCCATGATGTTCTTAAATAAAGAAGATTAATGTAAGACTAGCTGGTCACCCGAGCTACCATCGGGCCTTCGGCTGGATGAATTTTCAAAAATAATTTCTACGTCACCTACGTTATAAAAGAAACTCCCATACAAAATTTCATGCATTTTAGACTCAGCGGTATAGGCTGTGCGTGTGATTGTCAGTCAGTCTGTAGATTTTTA

>SinsOR9

AACATATTAGCGCAATCTCTTCTTGGCATCTCCAGTTCGCTCTTAAAGAATACCATCATGACCATAGCATTGGTATCATAAGACATGATCCATCCACTACCATATATAGATCCATAACTTTGAATCACTTAAGCAATCGAATACCCAGTTATAGTATTCTTATCGAATGGTACCCAGGAGCTTACAACTGGCAAATAAGTTTCGTTACCATTCCTAACATTTTGTCTATAGTGTGGTGATGAGAAATACTTTGTGATGGGTTGTAGTATAGCAATTATAACGGTCGCGTACATAAGACACCAATACAGGTAACTGATTTTCCTGCAATACTTTATGAATTTCTTAATGATAGTTTTTTGACACGTCCGATTCCGTGACGTATCTAATGATCTTATTCCAAACGAGGAACGTGCTGAGTTTGCAGACACTAACAGTTGCAAGTGATGTGATCTTCAAATTGTGCAAAAGTAAACTGATGTTGGATTTAACGAACCATATTATGTCAACACATTCTGAAGCAACAAACAGAATAACAGATAAATTTAATAAAATGTACATTTGTCTCATTATGAAATACTCTGGCAATATTAAACCAAAAAAATTTAACGCTTCAAATTGGATTCCAGTAGTGGCCTTTTTAAGTCTTCTAATTTACTTAGAAGTCTTTTTAACATCTTTGTTTTTTTTTTTCGATTAAAACGGGTAATATCAAAACGCGAGTGTATAGAAATGAAGTTAAAATCAATGTTATATTTTTAGATGTATTATTGAATAGGAACATAATATGTAGTATGCATGAGAAATTAGTTGATTCTCATCTTCATTATAAGGTGTTGTAGGTACAATCACCAACTAGTGTTGATATAGACATCTAAAGTTTCAGAAAGATGTAGCGAGTTGCTCCGTTATCCCACAATTATTCTTACTTTGCCTAAATTCTATGTTTAATTATTAACAAAAATCATTATGCTGGGCCACATAATGATTTTTGTGAGAGTTAGTGAGTCGCTTTACAGAATTTATCGACTTCTATTGATCTTCTTTGAGAGCCTCCACCAATCCTTATTTTTCTCCCAGACTTGATTTCCTGATGGAAAGGCCACTATTACTCGTATTTCTCTCATTCTATTTATTTATTCTAATACTTTCTATAATATACATTATAATTCACCAATTTCTTGTCCATTTTGACGTGGATACGTCTAT

>SinsOR10

TTTTTTTTTTGATATTCTACTTTATATTATGACAAAACGTGCTTATAATAAATCGTGGTTGTGTGCATAATGTCTTAAATAAACATCGTATTTTGAATTTTGTTTTTTCAACGACTGAAGATACGTGATTTTATTCTTTTTGCAGTAATGTGTTTGTAGACTTTTGATTTAAAAATAATATCTTGAGCTGTATTTAATTATCCATCGAACGAAGAAAAGCAAAATAAGACAGCGAAGACTTAAGTATCGTTGCCATGGATTGTACACCAACTGGTACCAATCCCATAGCTGTAACTTGTATGGGAGTTTGTACTCTATGGAGCAGGATGCATACTGATCGCTGGTTACTGATATTCATGCTTTCCCAAGGCATGCAATATATGGCATCTATCAACTTTTCACTCATATAACCAACTATCTCAAAGATTATCGATATTTGAATAAGTTGTCCAAAAAGTATGACAGTGAGAGGACCATAGCGAGCAAAGGCGTCAGCACCACGCGAACATTCAAAAAGCAATAGACAGCCGCTGACAAGGTGAAACAGATTGTAGGAAGCTAACACCGGACCAAAAAAGTTCGATATATCATCTGTAAAATTGACTATAAGTCTGTGATGATGAACGATATCTACTAATTTGGTATGAATGGAACCGTTTTCTTCAGCGCTGAATGGCATACTATAAGACGGCTCGGTATCATCTATTTTATTAGTGTTTCCAGTAACCGTTCTCGGTTTTGTTATATTTTCGATATTATTTTTTAATATTTGTATGTGTCCAATTATTTGAAATATCATTACAGACAGATATAAGTCCAAGAAGCAGACGCATATTGCAGTACAAAATGATAAATAGAAGTTGTAAATAGTGCATACTATAAAATAATCTTCGGCATTAAATCCAGGGTAAAAGTAATAATATATGGAAAATTCAAGCGTAATATTTTCACGTTTCTCGCCAGTAAAAGCGCCCATCTTATAATTGTTGTAAACAGGCGACCCATTAAATAAGATTGCGCCGCACATTGTTAATAACAATAAGTATATTGTGAAATAATACGATATCTTGTTGACAATTCCGTAAATCTTCTCATGATAATCGCTTTTGTGTTTGAAATGAATCAAATGAAATGCCATCAAAAACTGTCGTGATATATCGCGGTATTTTTTTAAACATACCGTTATTCCTCTAATACATATCAACACAGTTAAGAAGGTCGTGATATACATGTGTCCCATTGAGAATATATTCAACACCTTAAAGTATTTAACTAAATAACACACTTGTGCTATAAAGAGACCAAGCGCTTGGAAAGGAATATAAAGTCTGTGACATTTCAAAATAAGAGGTGTTTTCTCTCCTAATACTTCGCCAGGCCATGCGCCGTTTAATGATAAGATGATTCTCATTATCTTTAAATGTTTAATTGCAAGGATTTCTTTTGCAGATATTAAAGTATTCATAATGAATTGTTAAATATTTTCTGTAATATTGAACGTATATTTTTACAGTGTGCAGCAGCTGTATAGGCGTTTATTATGTAGAGAATGGCTCTTATATTACTTCTTTACTGAAA

>SinsOR11

ATTCTCGGTCGGATACTCTAGCACAAAACACAAAAATGTCTGAGTCTCCAACTGAACTCGCAAAACGTGAGATAGATGAATCTCTAATACTTTGTAAGTTCTGTATGCGATACATAGGTTTATCGTTTGAAGAACCGAAAAGCAGACGGGGATATCTTACTCAAAAACTAATGTTCTTACTATCTGTGTGCGTTATTTTCTACCACGTCTTCAGTGAAATCGTTTACATAGGTCTCACGTTGTCCAATTCGCCACGAGTCGAGGATGTTGTTCCGCTTTTTCATACTTTTGGCTACGGCGCTCTGAGTATTGCAAAAATGTCTGTATTATGGTACAAGAAAGAGAAATTTGGAGAACTCCTGCAAGAGCTGGCAGGAATCTGGCCGATGCCTCCACTCGACAAAGCTGCTCAAACCATCAAAAGCAACAGTCTTTCCGCTTTAAGAATGGCACACCGATGGTATTTTACAACTAATATAGCGGGTGTGTGGTTCTATAATTTAACACCAATTGGTATCTACTTCTATCAATCGTTACAAGGACAGAATACTGAAGTCGGCTATGTTTGGATGTCGTGGTATCCATTCGATAAACACGAGATGATAGCACACGTTGCAGTTTATATCTTCGAAGTATTCGCCGGTCAAACGAGCGTATGGATTATGGTTGGCACTGACCTACTGTTCTCCGCTATGGCGAGCCACATTGGACTCTTACTGAGACTACTACAACGTCGTCTAGAGTCACTAGCGACGACCCATCAGACAGATGAGGAATATTACCAGGAAATCGTATCCAATATCAGGCTGCACCAAAGGCTTATCATGTATTGCAATGATTTAGAAGACGCTTTTTCTCTATCAAATCTTATAAATATAGTGATGAGCTCTCTTAATATATGCTGTGTCGTATTTGTAATAGTGCTCTTGGAACCGTTCATAGCTGTTAGCAATAAACTGTTTTTAGGTTCAGCTTTAATTCAAATCGGTATGCTATGTTGGTATGCGGACGACATTTTTCATGCGAACTTAGGCGTGTCCATGGCCGCATATAACAGTGCATGGTATAAAACCAGCCCTCGTTGTCGTCGAGCGCTACTTTTCCTTATCAAAAGATCTCAAAAACCTATTGCGTTTACTGCAATGAAGTTTACTAATATTTCACTCGTTACTTATTCTGCGATACTAACCAGATCTTATTCATATTTCGCTCTTCTCTATACTATGTATAGCGAACACTAAAAACCCTTATTTTTAGTAGTATAACTAAAAAATATAACAATCGTGAATATTTAAAGAAAAAAACTTTGTTATTTATGGTTAATTGAATAATATTTTGAAATACACAGTTTTAAGATGGTTTTAATTAGAATTATTCGTCTTATTTATAATTTAACAATTAAAGTTATTCATA

>SinsOR12

TCTCGTGTGGCATTCGAACCCACGATCTATGTGTAGAGGCAGCGCCATAATATGGCTTATCTTGGATTTATATTATAAACCACTGAGCCAAGAGATCTAAAATTTCAAATGTGTTTACAAGATTAACAAATTCGATAAACATATTTTAAGTTATTTCGCTGTAAAAGGATTTTAAACATGAATCTGTACATCATTTTTCGTTGCTGATTATACTTTATTTGTATATTTTGAAGATTTTCTTATTATTGTAAATACGTAGCTTATCGAGACTGCATATGACGAAGCAATGTAAAGAAGGAATAAGCAGTATTCATTATAGAAGTAAATGTAGCTACTGAAAGAAGAAACATTTTTCCGCCAGTGATTGAAAGCGGTCGGTTAGCTCTCTCTATGAAGAATCGTAGATTGCTCTTGAATTGAGGCGATCGAGATATCCAATCACAGTCGTAGATCGCGCGAGTCAACAGGTAACTCTTCTCTATGACTCGTGTGCCAAACCAACATGGAACCATTATTTGGATAATCATGATGAACATATAAGTACTTAAAAATATATAATATTGCATGGGCGCGCGCAATGTACATCTGAAAAGACATACGCATATGATACAGGAGGACGTACTAAATTGTAAAAACAGTGCAACGCTAAACACTTGTTGTATTAGCGCACAGAATTTGCCTACTTGATCGAAATGTATGATAGATTTCTTTAATTCCAACAAGTAATAACTATCTTTGCATCTCAATTTTTGAACTACTTTTGTATCCATATTCCCATCAGGCGTTTTCTTAGTGAGTTTTCTTAGTTTTAAATCGAGAATATCTATTTGTGCTATCGTTAAAATGATTATACCAAGTAAGAAAGTATCTATGTTGACATTATATAACATATGAAAAAACATGCCGAAACATTGATAGAAATATATAGGATAGATAAACTTATTTTTGAACTCTTTAGATAGAAAAGAATAACTACATATAGGTAATTCGAGATTCGCTGAGAAAATTAAATGAATAATTAATGGGGATAACAAATGAGTTACATTAGATACAAAAGACACTATCGCTACAATTTTCCAGTATGTTACGTTAAATTTCTTAGCTTTAAGTATTATATCGAACCCATCGGGAATATTAGGCTGGAACATGTCACTTTCCAATGTCTTAAGTATCTCTATAATTTTATTTCGCATGAAAAGAAACGTGAAAACTTTAGCCGCAACCACCAATTCGGTGAAATAAAATATCATTTCTTCTATGAAATTGTCCAAATGTCTCGGCAAGAAATAAAAATTTATTGTGAGTAATATATAGTACAATATGACAAAAAATGAGACGAAACTCTTTGAGTAAAAGCCGTAATAACGACAAGTGTCACCGTCTGGCCAGATTGCAAGTAATTTCCAAAACTTCATATTTATATGAAAACAGTCAATTTGGCGAAACTGTTCCATTTATACGGAAATATTTAAAAACTATATCACTATAGTTATGTTCAAAACGAGTACTGTACAAGACTACTTTCGTAGTCTTGCTTCGCGTTAAATAGT

>SinsOR13

CGAAATTGACAGGGACCCTTAACACAAAACTCAGACCCAACTTATATGAAACAGTAAATATGTAAGCATTTTGAAGACAAAAAAATATTTTTGGTATCCTAAATAAAATGATCATGTAATTTTCATATATCAAGATTTGTTTTCTAAAGATCAAATTCTGAGACTAATACGTAGTGTTGTTACAGGTTTAAGAGATTCTTCCATTTTCACGATCTTGAACATTACGAATCAATGCAAAAAATGAATACGCCGTTTTCATTATCGAAGTGAAAGTTTCAAGAGACAGTGGAAACATTTTGACGCCTCTCAATATTATAGGAACATTTGCGCGTGCCACAAATAATCTCATGTTTCGTTTAAATTTTTCCGACCTTGGTATCCATTCGCTGTCATAAATAGCAAATACTGATTCTCGACTCTCATAGCTGAGTTGTGTCCCCATCCATGCTGGTACGAAAATTTCTGCTACCATAACAAACAAATACGAACATACAAACATCAGCGTCCCAGTGGATGACATCATAAGAAATCCACATAAGGAAACACATATAGTGGCCGCTGACATTCCAAACTGAATGAGTATAGTTACGTTTATCAAATTTTGAATTTTCGTACAAAATGTTGTTATTAAATAATGATGTTTCAACCAGTCATTGAGCTTTTGATATTGTATATTATCTTGAATTTCTAGGCTTAATTTGGAATGTTCTGCTGTTACTTTTAAATTACTTAATTTATAATTCAGAACCTTCAATTGTGTTACAGCCATTAATATCAAGCCAGATATTAGTGTATCAGCATTAACATTGTATGTCATTTGTGCATACATTCCAATAGATTGGTATATAAATAAGTAATAAAAATAACGATTTCGCAACTTATCATCAAAAAAATAATATTTACAAATAGGCAATTCTAGATTTGTTCCCATTAATTTAGCTATAATTATGGGTAGTAAAACTAAGAAAAAATAGGACAAGTGAGATAACGTAGAAAATATTTTCCAATAAGTTTTATAATAATACACATGCTTGTCAATTATTTTTCTACTGTTCTCGTCTGTTGCCTTGAATTCCTCACAATCCAGATGATTCAAAATATCAAGTAGTTTACTTCGAAAACCCAGAAACATAAAAACTTTAGCAGTGACCACTATTTCAGTAAAATAGAACATCACTTCGCGTATAATCAACTCGATTTTCCACGGCGTGAAGAACAGATTTATTGTAAGCAGTGTATTATAAATCAACAGCAAAACGACAACAATAACGAAAGTGTAGAATTTATAGTAATTGTTCAATGATTTTCCGGACCATACTCCAAACATTTTTAAAAGGAAAAAGTTTACATTAAAAGTGTGAGATATGCTAATCGACTCCATGCTGTTAAGCGGATATGAAACGACAAAGCGAATACATTTATCTATCACTTAACACATGACGTCATTAAACTTAACACCCATGACACGTATCATCATACACCAATTGAATTCTGG

>SinsOR14

GAAAATATCTCATTTTTAAGGGAACATAGAGAATACAGCGAAGCGTGGCTGAGTTAGCTAGTGTTAAATAAAAAGAAAATCAGGGCAAATATAAATCATAAACGATATCATAAGTAACCACTGGACAAATTTATCGTTAGTCAAAATCTTCGGATTTTTGAGATTTTACTTTTTTATTTTGTTCGTGGATTTGGATTGTTTCAAATTGATGTTCCTTTTAAAAGTATAATTATGTTTATTTCATTATTATATTGGAAACTATAATAATTTTACTAAAAAAATGTTTTCTGACTATTCGATGTAAATGTCAAATCCCCTTAAATTTAATTCCGATATTACAATTAAATATTCTCCAATTCAGATATGAACCTTAGTTTTAATTATTCGACTATTTTTTTTTTTCTTTACTACAGTTTAACAACTGTAACAAACTTATAAATGGCCTGCATTGTTTGAGCGAAAGTATTAATACATATAGGCACTATATCGGCCGCTAATAAGACTACTGGTTTCTGAGATTGCTTTAACATGAGAAATATTATCTTTTGCTCTCTAAGATTAAAGTTGGTCCATCCACAAAAATAAATAGCATTCTCAAATTTCTCAGTGGCTGTTGTAAGCCGTTGACCAAGATAACAATGAGCAAAAAACACTGACAAACTATAGGCTATTACTGCTGAGAAATTGAAACACACATCAATCGGTAAAACGAAAAATAAACAAGTAATAATTGCATACAGACCAAAATCAACACCTATAGCGAAACTGTAAATATTTTGTAATTTTGCAACTGTATGAAGCAGCAAGGAATGTCGATGCACCAGGTTTTTCAGACGATCCGCTGTTACGTCTATGAGTGTGTTTTCAAAGTTGTTATCATTCAGACTCATCATATCATAGTGAAGTATTTCAAACATGGCTGTAGTATGAGTAAATGAAAATATTAATATCATTTCATAACT

>SinsOR15

TTTCGCTTCACGTATTAATATATCAAAATCTTTCTGGAGAAATCATCCGTTTGCCTATTAGAGCCCCATCATTAAATAAGTCATTTTAAAAATTATCTGTCATAGTATCGTAGATATTGCCGAGGACATAGCGTAAAAGGGCAAAGGGCGAGGGGGAGGAAAGTGTGTCCGACTCCTACCCCATAATTAAAATGAATCTCTTATTTTAAAAACAAGATTCATCTAAAATTTAATAGACCTGACCTGCACCCCTCCACTTTCCCGCAGCAAAATATATTGCTACGTCCTTGGCCGAGGGTTTTTTCTTAAGTATGTATAATATTTGTAATAGTAGAAGTACATATTAGACTTTTATCCTAATTTAGATAAACAATTTAAAAAATTGTTATCGCAATGGACGAAAACCTACGATTCCCCGAATACACAAAAGGTAAAATTTTCCCGAAATCTTTACTAACGAGGGCTTTGTGTACAGAGGATAGTCACACGCAAACTTTACTATAACTTTGCTTTACTTAGATAGGTAGTTGCTTTACAAGTTTAAGTTTCAGCAGAAGGTAGGGAGGTGAAAATTTGAAATGAAATTTTGCTACGCGCTACTTTATTACTACTGGTCACGGATTTCGTCATATTGACACACGACGAAAGATATTATTACAATATTTATTTTAATTGTAATTTTATTATCTACAATTTACTATGATATTTCGTTCAATCCCATACTGCGTTTAAAACGTTAAATATTGCGTAAGATTGATGACAGAGCTCAGTGAAAGTATCTAAACAAACAATACGGAACACTGTTTGTATTGCTAGAGGTTTAGCAGCTCGTGACAACATCAGCACGAGAGTGGAGCGCGTGCTGCGGTCCCAGTAACGTTCCCAGCCACTCATGTATACCGCCTGTCGCAGACTCTCGCCCTTTTCCATCAATAGATCGCCGTAATAACAAGGCACATAACAGAAAGTTATGCCACCGATAATAAACGTACAGAATTCTAAACTAAATCGACCTTGTTGGAATGAGTGTATAACCTCATAAAACGCTATTGGTATTACAAATACAGTTGTCTTTAGAATTATTTCATACAAAACTTCAAAACTCTCTTTGATGTCATCTACAAAATGATATATTTCTTGCAAATGTTTAACAATATTCTTAAGTTTGGTCCGTGTTTCACCAAGGTCATCTCCATTATTTGCAAATAGATTTTGCATACGTTTCTTTAGAATTTCTAATTGTCCACAAGCGTGAAGCATGAAAATGGGTCCCATAGGTGCAAAACCGATATACATTATGACAGAAATAAAAACGTAGAACATCACAATTAAAAAGATAATAGTGAAACCTATATAATTATCTTTAATGCCTTCTATAAGAGGTGGATATGTCAAATTGTACATTTGTATAAGTCTAGATTCGCCTATGATAACGTAATATCCCATGAGGATGAAAGCTTTTACTGGAAATATGAGACCAACACCTCCAGCAGTAAGGAACCACAATTTTGACACCCATCGTCCTCTTTTCGCGTACTCCAACACTAAACATTGCTCCTCATACGATAGATTTTTAGCAAATTCAAAATCTAGCTTCATCTTTTCGATTAACCGCATCAATATTTTCTGATACCACAACATAACAAAATATTTAAAAGTGACTACAGTGAAAACGACACACAGGATTCCATTTCTGCAAGCTTGAGTGAAATCATTGTTTTTCAAATCATAAAATATAATACAGTACGCTAAATAGGAAAATACGAAAAAGATTATACTATACATGAGGATAAATTGTAAGAACCATTTTGTATCCCTTTTTACAGTTGGGTAAGAGAGATTTGATTTTAAACAACACACCGGTATTTTGAATATCTCATCAAAATTTAACTCCATTTCTTTTATCTTTTTTTTTATATTTCCACATACGAGATACTGATAATATCTGATACGTGTATATAAATACGTTTCAAATGTAGCAATAGAGCTTATTTTG

>SinsOR16

ATTTATAATATTAGTATGGATATAAGGACGAGCCATTTTAAATTATACCAGATTTTGTATTGTAGGGATTAAAAAATTAGGTCTCACGGCTGCTATTTTATTTTTATATGAGGCCACCTACTCTATGACACTCAGACTGTCACAAAGAATCAAATGATACCTCATATATTGAAATTTGTTTACACAATGGGGCTCTAGAGTGGATCTTAATAACAAACACTACCTCATGCATAACGACAAATCAATCACATTATTCTCCTACTTAAGACTTCGCCGTAATCGGATAAAATCTACCAGTATTCCCATGTTTCCTTTTCTGTATATTTGAAATGTATATTACTAGGCATATATATAATCGTCCAGACCCTGGATTTGTTCTATATCTGGGGAGACTTGACCCTGATGACTGGTACTGCTTTCTTGCTCTTTACGAACTTGGCTCTGTTCACCAAGACCGTAAACGTCATCGGTAGAAGATCAGCTGTTAGAAAGATTGTCGACGAAGTGAATAGCGAATTGCAGAGTGAACAAGAGGAGAAGAGAGTAGCGATTGTGGAAAGCTGCAACCAGGAGACCAAGCGGCACCTGTGTCTGTACATCGTGCTGACGGCTGTCACGGTGTTCGGATGGGCCGCTAGCGCCGAGAAGGATTCATTGCCATTGCGGGCGTGGTATCCGTATGACGTGTCGCAATCACCGGCTTACCAGATTACATACGCGCATCAGAGTTTCGCATTGATCGTGGCAGCGTCTCTGAACGTGAGCGTGGACACGTTGGTAACGTCGCTGATAGCGCAGTGCCGCTGCCAGCTGATGCTGCTCGGATGCTCCCTCAGAAACCTGTGCCGTGGCATCCCAGTCAGAGATATGCAACTAATGTCCCCAAATGAAGACAAAACTATCAGCGTCCTACTTAGGAAATGCGTGTTACGTCACCAGGCAGTTCTGGAGACAGTCAAACTATTGGAGGAGTACTTCTCTACTCCGATCCTGGCACAGTTCACTGTTTCTACTGTTATCATATGTGTTACGGCTTATCAGTTAGCCTTTGAAACAATGAAGATGGTTCGTGTGATATCCATGCTTGCGTATTTATTGGATATGATGCTGCAAGTTTTCTTCTACTGTTATCAAGGACACCAACTGTCTGAAGAGAGCACCGAAGTCGCTGGCGCCGCGTACTCCTGCCCGTGGTATTCGTGCTCCGTACGTATCAGAGGCGCCATCCTGATCCTCATGACACGAACCACGAGGATCGCCAAGCTTACCGCCGGGGGATTCACCAACCTTTCTCTATCCACCTTCATGGCTATAATCAAAGCGTCTTACACTTTCTTCACGGTACTGCAGCAAGTTGAAGGCAGGAAGCCATAAAACTATCAACTGATATAAATGTTATTGCAAAATAAAAATTGTAGAATTTATCAATAAAAAAA

>SinsOR17

GTTTGATGTTGGGGTTAGTGGTAAAACGAGGGAGCCCGTATACGTATATATAAGGCAGGATTTTTTTTATACCTCATTTGTCTTCACACATTCACTAATATCGGGTGATCAGAATGGTAAAAACTTTAACTGAACGATTGGAAGATCCGGAACGGCCATTCCTAGGTCCTCATTATTGGTTAACCAAAAAAATTGGACTGTTCCTACCGAAAAGTAAATTAGGAATTGTCACTAGCTATGTGATTCATGAAATAGTGACATATTTTGTCGTAACGCAATATATTGAATTATATAAACTAATCGTGTTAAAGGCCGATGTAGATATTTTACTCTTCAATGTAAGATCTTCGATGCTGAGTGTTGTTTGCATTGTAAAGTCGAATAGTTTTCTTTATTGGCAGAGTAAATGGCACGACCTCTTCGAATATGTCACTGAGACGGATAAATTCGAAAGAGAAACTCAAGATGAAGTAAGAGTGAGCATAATTAACAATTATACCAATTACTGCCGACGTATTACAAATGTTTACTGGTCTTTAGTGATCTTTACCAATTTTACTGTGATTTTTACGCCATTGATGAAATATATGACACTCTCAGATGAACATCTTAAAGCTATTGAGAATGGTACTGAGATGTTTCCTCACGTATTTAGTGCCTGGACGCCATTTATTGATAAAGAACACTCGCCTGGCTGTTGGATCACAATTCTATATCATGCGATAATATGTAGCTTAGGCACTTTAATGGTAATCAGTTATGACATGAATGCGGTTGTCATCATGGTGTTTTTTGGGGGAAAACTCTTACTGTTTCGTGAGAGATGTAAGCAACTGTTTGATAGCGACGAAGCTGGTCTCAGTGATGAAGAAGTTCGGAATAGGATACGCGATCTGCATTTAACGTACGTGCGCCTTGTTAAGTATTTCAACTTGTTCAATTCTTTGTTGTCGCCCGTAATGTTTTTGTATGTGGTGATGTGTTCACTTGTGCTCTGTGCCAGCATATACCAGTTAACTTCTAGCAAGGACACGATGATGACCAAATTCATAATGGCACAGTATTTTATTTTCGCATCTTCACAATTATTTCTGTTTTGTTGGCACAGTAACGACGTCCTGGCTATAAGTGAAATTATTATGTACGGACCGTATGATAGCAAGTGGTGGGCAGCGAGTGTTCGTCAAAGAAAATATGTGTTGCTTTTAATAGGGCAGCTAAGAAAAGACTTTACTTTCACCGCTGGGCCGTTTACAGACCTGACTTTGTCTACGTTCATAGCGATATTGAAAGGAGCTTACAGTTACTATACTCTAGTGAGAGATTAAATTTTTATATTAATGTATAGTTTTCATGTGAAAATGATGACAAAGTATTTTATTTTTTTATATGTAATTGTAGAAATTGTGTAATTTGCATATTTTATAACCCAAACAAAATATGTTTTTCAACTGACTTCAACAAGAAGGAGGTTCT

>SinsOR18

AAAAAATATATATACATTAAAAACACGCTTCATTCTCCAGACCTTTTTGTCATATTTAGTCATTGGCCGTGGAGTTCAACAACTTTTTTTTACTGGAAACGGCCATAAAAAATTAAAACAATATCTAATAATAAATGACTCAATCAAATCAATCAAATGACAACGATACGACAAGTTACCCTAATCATCTACATCTACGTTATTAACTAAAGTTTTATCCAAATTATACCATATATTTCTCTCTTTTTTCAAAATCTCTATATATTGAAATTTTGTCCAGCTATTTCGATTTTCTGATCTGGCGATTCCTAAACATGATACATAAATTCGGCTTGGAGCACTGTGACCTGCCAACCATGCTGTGGAATGTATCGTTCCTTCTAAGAGCTCTCACGTTGAACATTGATGGCCGTTATAAAAAACGTAAGCATTACCATCTACTTAATAATACTCTTACACTAACGAGACTAACAGACAAGGTACCGCCTGAATCGGTGGGTCCAGGAACATAAAATTTTGCATAGAAGAACATAAAATATTTCCTGGTTTAACGAAAGTGAGCACTAAGATTTTTGAAAATTCATACCCTAAAGGGATTAAATAGGCACACTAGACTTTTATTTTACCGATATAACATATTTGAAGTGTTATACCAGCATTATTATCAGCCGTCATAAATGTTGTCACTTGATGCCGCTATTATAATAAATTTTTGGCGCACGTGAAACTTACAGTCATTAATATATAATATTTAATGTTAATGATTGTGTAAGTTATTGCATCTAACAATGACGTCGTTTAAAGTAACAACAGTGGCTCTACATCCGCTCGACCATCGGCCGCAGACCCACCGCTTTCTGGGACTACAACTTTTTTTGTGCAAACCGTAATGATATTTGCCCATCTTGATCATTAGATTGCCTCGACTCGACATTATGAATTTTGCTCTACCTTGTGCATCACCTACAGCG

>SinsOR19

GACTATTGCAGTATTGAAATCTACAGCCTATATCAACAGTTTATGATACTCAACTGGTATTCTGTCAAAAGTGCGTTATGATTTTTCAAAGAGTAATTTCGTTCGCTAATGGACTCGAAGACCCTAAACATCCATTATTGGGACCAAATTTGAAAGGCCTCTATGTTTGCGGCCTTTGGCAATCTGGAAGCAAATTTCGCATCGCTTGTGTTAATTTCATATATTTTCTAGGGATCATTTTTGTCTGCACTCAATTAGTCGAACTTTGGATTGTGAAACACGATTTTATGAATGCACTCCATAATTTGTCCGTAACTACTCTCGGTATTACTTGTGCCGTAAAGAGTGCCTCTTATATATTATGGCAATCGCGATGGAAGGAACTTGTAAATGCTATATCAACGGAAGAGATATCGCAAATGACGCAAGAAGATAGTGTTACGCAAAAACTAAGAAAGAACTACACGACATACGCAAGGGTTGTGACATACTTATATTGGTATCTGGTTGTGATGACGAATATAGCAATGATATTTTCACCGCTGTTAAAATACGCAACCACTACAATCTATCGTGAAGAAATAAGCAATAGAACAGAGCCATATCCATTAATCATGAGCTCGTGGTTTCCTTTTGATAAAACTAAGATGCCTTGGTATTGGATTAGTGTTGGAGTGCATATATGGATGAATATTCAGGGTGGTGGTACCGTCGCAGTATATGATTCGAATGCTGTTGTTATCATGATCTTCTTGAAAGGTCAGATGAGGATATTGCGAGAGAAATGCAAAACATTGTTTAATGATAGCGAGAATATTCAGCGAGAGGATATTTTGGATAGAATCAAGGAATGTCATAGGCATCATGGCTTTCTCTTGACACAAAGTGATTTGTTTAATTCCGTTCTATCTCCGATTATGTTTCTGTATGTTTTGGTGTGTTCTATAATGGTATGCTGTAGTGTAGTGCAATTCACCTCGGAGCAAGCGACTACTTCTCAAAAATTGTGGGTATTGGAATACACGACGGCGCTCGTCTCCCAACTTTTCTTATACTGCTGGCATAGTAATGAGGTCCTTGCTGAGTCAAATGAGATAGACCGTGGTGTATTTGAAAGTGATTGGTGGAAAGCAGATGTGCACATACGCAAACAAGTAATACTGCTTGCAGGGAAGATGGGTCAACCCTTTTTGTTGTCTGCGGGGCCGTTCACAACTCTATCTGTGCCTACATTTATAAGTGTCATCAAAGGCTCTTACAGTTTCTACACTTTATTCACACAAATGCATGAAAATAAATAAATTTTTATAAAAAAATTATGTTTATCAACAAAAAGGCAGCACAACATTGTTTATAATTGTGTCGCATCTTGTGTTTGTTAAATGTTGAAAAAAATTCAATTATAGTATTTTTAATTCAATTCAACGTGCGGAAGCAACAACGTCGCGA

>SinsOR20

TAGATAATAAGTATTTAAAAGATGACATGTTGTTATTTTCTCGATTCTTCCTAGACTTATATAAAACAAGGTTTTACTTAATAGGTGCATTGATTGTGATTACCTAAACATTTGTTTGGTTTTTAAAAATAAATTTTGAATGAAAGTAAATCGTTAATATGTTTTTTTTTAAGGAGAATGACATGAACACAGTAAAGAGGCCTCAAGATCTCCGATACATGAAGCAATTACGGTTCTCTCTAAATATTGTGAGTGCTTGGCCACATAAGGAAATCGGCGATCCTGAGTCCAAATTTGTGTTTTGGTGGAGAGCATATTATGCATTTGTGGAAGGATTTTTTTGGTTTCTTGGAATGGCTTATTTAAAAAACCACTACGGGAAACTAAGTTTTTACGAATACAGCCACACCTTAATAACTCATTTCATGAACACCATTACTTGTCAAAGATTGACTTTGCCGTTTATGAAGAAGTATCGTGATTTCATTGGTCAGTTTATAAAGAAATTTCATCTTTTCCATTACAAAGATAAATCTGAATATGCCATGAAGATTTATCTACGAGTGTATAAATTATCAGATTACTTCACTATGTACCTTCACATATTAATGTATGTAGGCATAATGTTTTTTAATTTTACACCGGTTTACAAAAATATTATCACCGATGCATACAATAACCCAATAAATGCAACATTACAACATTCATCATATTTCGAATTACCCTTCGACTATAAACACAGTTTGACTGGATATGTTCCACTGTTCTTTTTTAATTTGTACATAACTTTCATATGCGCCTCTTTCTTCTGCATGTTTGATTTATTGTTGACCGTGATTGTGTTAAATGTTTATGGTCATCTGAAGATTCTTGTATACCATTTAGAACATTTCATGAGACCATCAACGAATTCGACATCTCATAAACATAAAAATAAGATGTTCGATACAATGCAATTCTCTGAAGAAGAAATGAAAACAGTAACTATTAAATTAAAAGAGGTTATATCCCATCAGCGTCTTATAACAGATTTCATAGACAAAATGTCGGATATTTTTGGGCCGATGGTATGCTTAAACCTCATGTATCAACAAGTCAGCGCATGTATTTTACTGTTGGAATGCTCGCAAATGGATTTACTAGTTTTGTTTTCCTATGGCCCGCTTACATTTTTTGTATTTCAAGAGTTGATTCAGTTATCCGTTGTGTTTGAACTTATTGGAGCGACGAGTGACCAATTAATAGACGCCGTATATAGCGTGCCCTGGGAATGTATGGATACTAAGAATAGAAAAATATTGTACACTATAATGATAAAGTCACAAAAGACGACAAAGTTTAAAGCTATGGGCATGGTGGATGTTGGCGTTAAAACTATGGCGGCAATATTGAAGACGATAATTTCCTACTTCGTTATGCTTCGAACAATAGCTTTACAGAATTAACAACTGCTTTGTTCTATCATACACTCATATATGTATTTATTTATAAACTTATTAATGTTCAATTCATGAATTAAACAAATAGAATATTAAGAATTTATAATACATTAGATTATACGTATAACAACGCAAAATGGATTGTATTTTTTGACAAACAACACGTTAGCACGTCGAAAAGCTGCAGATGCATCGTTTTGGAATAAAGTTATAAAGCAAACCTCGCTTAAATACAAACATTGCGAACTAGGATCCTTTCAAGACAAGTGTGAATGGGCATTCATTGGGCCAGTGCGCACCATCTTCGGCCTTGTCGTCGTTTGCCATCAGTATGTATATGTAAGAAATAACTATAATTGGTGCCGTCCCAGAGGCCGCCGGAAAAGGCAAATGAAGAAAATACGGAGGTATTGGCTCCAGTCCATGGGGTCCAGAGATGTTAAGCTTTTTAAAGAATTGACCATGCGCAAACACCCTGTCGGCGAATCTGATGGCCCTTCATTAGGAGCCAAGAAAACGTAAGATATTTCACGGGGACTAGAATTCCAATAAAAATGCATTTTGTTTCGAGGTTACTTTTAAGAGCAAGATAAGGTGTTCGTCGGTTAACCTGCTTTGAAGACAGTTCTTCCGCAGCTTCATGGTGGAGAACATATCTTTTTTACACCTACATATTTGTGTCGTGTCCGAGGGCCATATACTCCCACTTTAGAAGGGACGTTTGCGGACCAGGTTAAAGCCCTTCGCGAGTCGTACTTTGCCCACCCCTGCCTTAGCTAACATTTTTCTTGATATCGCATCTGCATCGCAATTGTACATGGAAATGTTTACACTGCTGCCAGCAGGATGGCGCAAGGGCTCTTTTTAAAAATTCTAATGACATTTTTTAATTTTTGTATGTACATTAATAATGTGTAGGTATATTATCTACCTACATAAAGTGTAATGAATTCTTTAAGCAGTGGACTGTAGAATTTTTATATATTATATTTTCTTTACCATTTTTTCAAACTTGTTCGTTGAATTCGCTAAGTTATTTTCTTCGTTGAAATAAAACACCGAGTGAACAGGAAGGCGAGATATTGCTGAGCGTGTTAGATGTGAACTCCAGTGGTGGGGGGCAGTAGTGAGCAGGAAAGTGGGAGATGGGCTTGAGGAGCCCACCCAAATTTGGTTTTGGCTTTAAAGTGCAAAATGAATTTTAGAGTATTTTTCACATCACACGTACCCATCAAGTATCAGTATTTATTGAATAATTTATAATTCTTATTATATATTACAGGAAATTTGCTTACATGTAATAGACAATAATGATACACATATGTACACATAAATTTCAACACTAAAACATACTATGTGTGAAGAAATACAACCTTTTTATAGTGTTTATATTTCTCATTTGATATTGTCGCTTAGCAATGTTGTTATTTGGTCAATGTCAATGTAAAATAAAATGTATTAATTATTTTTTTCTGCACAAATTCAAAATAAAGTTAATCTAATTTAATCGCATTTGTCCTTCAATAAAATTCAGAACA

>SinsOR21

GTAAATAATAATTGCAATATTTTGTAATAAACACACATATCTAGTAAACTGCGGCTCATAATTAATATGAATTTTTTAAAAATGCGTAGAATGTATATGATGTACGCAGGATCGTAAGAAAAGTATTAGCTGAAAGTGGTATGACAAACCCAGCCTTAGGTTGAATTGGGCGTTTAATTCTCTCCATAAAAATAATCAAGTATCGTCGTTGTGTAGGCGCAATCATGAGCCAGTCCATTGCGTATGCAGAGTTCATAAGTTTGTCACTCTCATACATTAGTTCATTGCCATAGAAGCAATATAAATATGGTTGCGTCATTACACAAAGTATATACAATATCATAGACAGAAACTCTATTGAACCTGGACTCATATTGATCATTCTGTATGCCGTGGTGCAAATAATCCATCCGGTAAAAATAAACTGAACAAACACGGCGTTTCCAAATATTTCTTCAATTTTCTGACTCGATTGAACAATATCATTGTAATGTTGCATAAGACCGATAAAACGTTTCTCAAAAGCCTTTGCAAAAGAGATCCGCAGGGTGGTAGAATCATCAATGCTTTTCTTAACAATGTTCTCGAGATTTTGTCTGAGAATAAAAAATTGAGTTGTACATTGAGCCAGAAAGAACGCCATGGTAACATCCATTGTAGAATTGTTAAAAGCAAGCCATGATGTTTGGAAACATACATAGATAACAACGAAGTAAAATTGCGGATTCTCATTGGAATCAAAAGGCAGCCATATTCCAAATTCTACAACCCTTCCTCTTAAGTGAAATATCACTGGGAATATGGCCCACAGAAGGCAAGTTATTAGACACATGATGTTGAAGATGCGAAGTACAAGTTTCGCTTGTCGGACAGTTCGTATTAAAATTTGTTTGTGTTCTTTCTGGTTATAAATGGGACCTTTCATTATATTCATCAACCCTTTAATTTCATCAGCTTTATTCCAGAATGCAGCCTCTTTGATAAGACAATCACAATAGGTCAAGAATAAAAACATTGAATCCATTATTTTATTTATATTACGACGCCACTGATAAAGGTTTATTATTTGTTGCGTAGTATACAAAAGTAGAAGAGATACACTGAAGCGATTATATAATTTTTGCAGAAATTTTTGTAATTTTGGAGCTTCAGGATCTAACGGATAAAAACCAGTTTGGCGTAATTGTTTGAAATGAGGCACTAAACTGTAATAAGCTAATTTCTCTTCCATTTTTGTATGAAATTCAAATTTAAAAACACTTTGCTTTAAAACACTTTTTTTCCGAGACAGCGT

>SinsOR22

GAATCATACTCGCATTCTCGCGTTAACTACTCTGCAAGTGTCAAAATGGGAATTAATTTTCAAAATATTATAAATAAATATTCAAAAACTGAAGGCGACAGAACGGAAGTGGATGAAATAATGACCGTAGCATTGATTTTGCAAAGAATTTTCGGTCACCAAGTTTTAGACCCCGATTGGACTTGGAAAAAATACTTTTTGCATCAATTTCTTACTCTTCTACTATTTATTTATGTATTTTTTGGTACTTTAGAAGTCGTCAGCAGCACATCCGATCCAGAATTAATAGCAGAAGCTAGCTACACGCTAGTACTAATCATAATATGTCCAATAAAACTTATAGTTTTCATAAATAACCGGTTTATATTCAAAAAACTTTATGTGATGGCAAAAACTACACTTTACGAGGCTATAAAAGCGGATTCCAAGGCGAAAATCGAACAAGTCTTGAATAAAGGAAGGAAAGTGACGTATACATTGTTTGGGATGGTCGTAATCCCGGTTTTTGTATATGAAGTTACAACAATTTGGAATTATTTCAGAGGACGAAAGATTCTTTTATCAAGATCGACATCAACTCTCATGCCTATGACCACGCCATGTTATGAAGTAGCAATGATTTTTCATAGCGTTTTTTTGACTGAAGTATCTTCTATTACGATAGTAATGGATATGTGGTTCGCTTTTTTAATGTTTTTCTTTTGCGTAGCGAGCGATGGTTTGGTGAATATTCTGGAAGTTAAGTCTAAGGTAAAATATGAAAGCGGGCTAACTTACGCGAAGCGTCTGAATGAGAGTTTGCGGAAGTTTCACAACGCACACGTACAGCAAGTCGAATATTTGAATACCGTGAATGCGATGTACAAATGGTTAGGAGTGATGCCTCTCTGTCACGCGGCTATGTCTATCTGCATTGTTTTACTGGTTATAAGTAACGACATAAATTGGAGATTCACTGTTCATATGTTTCCATTGTTTGCTGAAATTTTTGTCTACAATTGGTTTGGAGAACAGATTAAAATCAAGGCGTTCGATTTAAAAACTGCTCTCCTCAATTTCGACTGGATGGGCTTGGAAATTAAAGATAGAACCCGTTACTATATCATAGTAATGTATATTTCAAAAGAATTTGGAATCAAAACTGCCGTTGGGAATTATTTGTCCATGATCACTATGAGCAATGTGCTCAAAAGCAGCTATCAGGCGTTTACGGTTTTTCAATCTGTCAGCTATTAAATATTTATCAATTACACTCATATCATATAGACGTTAATATCATTAAATACAATTTTCCTCAAGAAAAAAACTCGCAAAAGTCACTTAAATATAAATATATATAATATGCAAAAAATATTTGCAATTTATTTTATTACTTGCCTATAAATAAATAAATATTACGTTACGAGTCAAATTTTAACATAATGATTTATAGGCTTAACATCTAC

>SinsOR23

ATATTGCGCGCACGGAGGAGTGTAGGATTTGGAATAAGTAATTAAAGTTTATATGAAGAAGGAGTGCAGTAAGCTAAAATTTCAAACTGGTCGATTTCTTTTACGGTCCATTTACTGGACCACTAGTCTCTACAAAAAGCCAGAAATTGCAGGTTCATTTCACTATTTGAATTTCCAGTTAAAAATCTAAAATTTTCAAAAAGAAAACAAATAAAGGAGAAATTTAACATGGAAAAGGAAAAAATTCAAAATTACCACGATTATGCAGACATAATACCATTTAAACTCTCTGCTTTTCTGCCATGGTATATCAAACCCAGGAATGAATATGAAATGATATTCAATAATGTTTATTTAGCTTTCGTGTTATTTATTTTAATCAATTTGTTGGTCACACTTTTGGTCAATTTATACGTGGACTGGATTGATTTCATGTCATGTTTGAATCAAATAGCTGATGGGCTACCGTTGGTCGTGTCAATTGTTGTGGTCGTGTACTTCGCTTATTATGACGGGGAAATGGCGCAACTTCAGCGTTTCATGAGAGATAATTTTAAATATCATTCGGCGAGAGGAATCACTAATACAACGATGCTGAATAGTTATAAAGTGGCGAGGAACTTTGCCCGCTTTTACACTGCCTGCTGTCTCTTCAGCGTAACGATGTACACTTTCATACCCATGATTGTGCACTTATGGACTAAGTTGCCACCTCAACAGTGGGTGTATGTGGAAGCCACGAGATTTCCTTACATTCAGATCATATTCTTGAGACAATGTCTAGTGCAAGCATTAGTGGGACTGATCATTGGACAATTAGGTGTATTCTTCGCCACAAACTCCATTCTGATCTGCGGCCAGCTAGACTTGGTCTGTTGCAGCGTGCGCAACGCGCGCTACACCGCGCTGCTGCAGCATGGCGTACTGCACTCCGCGCTCGCCGCGGCGTACGCAGACATACGCGACGACGAACGACACAGTTATACTTATAATATAGCAGATATAAAGGATTCCGTATATCACTATGATAAGAAAATGGTAATATTATACACGGTCAATTTCATCGACACGAAAACGGAGTTCGATATATACAGCCGAGACTTCGACCAACAGACCATCGAGGCAATCCGTGAGTGCGCCCGCTTGTGCCAAGCGGTTGCCAAATACAAAGACATGTTTGAGCATTTCATCTCACCCGTTCTCGCCTTGAGAGTCATCCATGTTACGCTTTATCTTTGTATGCTCATGTATTCCGCTAGTGTGAAATTTGATATGGTCACCGTTGAATACGTCGCTGCGGTCGCCTTCGATATCTTAATTTACTGTTACTACGGCAATCAAATTATCATTCAGGCGGATCGAGTATCGACGGCAGTGTATCAAAGCGCATGGCACACCATGGGTCCCTCGCCGCGCAAGTATTTACTCCACATCATGCTGTCATATAAGAGACCAGCTAATCTACGCGCCGGAAGATTCCTTACCATGCACCTCGAGACTTTCGTATCTATCATGAGAACTTCATTTTCATATTATATGCTGTTGGTCAATGTAAATGATAAATAATTGAGTATTCAAGACCTTACTTTAATGTTGAAACGTGAATAGATCAATGTTTTAGAACAGCATAAAATTACATGGATAAATACTGTCGTGAATAATGTTATAATATTGTGTGTAGTTATAGAAAGCGATGTGTTTCTAGTTATAGCTGATTGTTAACGTATTTGCACCTGATCATAAAATATTTTATTAAAGATTATAAAAGATAAATAAAAGTGTT

>SinsOR24

CGCACACGAAGGAGTGTTAGATTTAGCATAGTTAAATTTTATATGAAGAAGGAGAGCAGAAATAAGCTTCTGCTTTTTTTTTTAATTTAAAATTTCGATGTTTAAACTTAAAAAATTAATAAATAACTGTCGAATTTCGACCGTTTGCAGGTTGGATTATGGTCGAGTTTCGACCGTTTGTTGGACCTATAGATTTTATAAATAGACGTTAAATGTGTAGTAATTTTGATATTTTTAAAAATGTTACTTATGTCTTTTTTTAGCACGGCAGTGTGGGTGCGACCAATAAATGAATGAAAGTATTTCAATATGACATAGAAGAGTACGACGTTACATATGCTGTTCCAAAATCAGTTTTGAGATTGGTAGGCCTTCGTTTTATGCGGAAGGATTCAGCTTTTGCAGATATGTGTTGGAAGGTATTTTACTGGTTCGAATTCACAAATCTCTTCATTGTCACTTGGCTGGAGCTCATAAATATGGCACAGGTTGCACAAGGGGGCTCCTTTGCTGATGCTGTCGAGATCTTCAGGATGATGCCGTGCGTCGGATACTTGTTGCTGGCAATGGTGAAGTCATATAGGATTGTATTGTACAGACCGGTGTATGAAAATTTATTAAGTGAGCTCCGTGGTATGTGGCCAACAAGTGCTATAAGCGAGGAAGAACATGGTATCATGGATAAAGCATTAAAACAACTTAAGCATTCCATTAAGAGTTATTATTGGTGCAACAACGCTTTGCTAGTGGTGTTTCTCTCGGCGCCGTTTGTGGAGATTATAAAACGCGCCATGGGAAGGAATGTGCCGCTGATCTTACCATTTTTCTACTGGTTTCCCTTCGACCCCTTCCAAAGAATCTTGTATGAGATTATTCTGGTATTCCAAACGTGGCATGGTTTGATATCCATTTGGTTTATGGTTGGCAGTGATCTGTTGTTTTGTATTTTCCTGAGTCACATCACAATTCAGTTCGATCTTCTGTCAGTCAGGATTCGCAAACTGGTCTACGTACCTGTTGATAATCAACTAATTGACGACTATCCACTTGCATCCTATAGTCACGAGTACATCCAGAACAAAAAGGGAATGGCTGAAAATTACAACGATACGGAATGGGAAACCTTACATATCGGACAACTGTCGGAAATTATAACAAGACATCGCACTTTAATTAGACTATCCGAAGACGTCGAAGACATCTTCAGCTTTCCACTGCTGGTCAACTTTTTCAACAGTTCCATCATCATCTGCTTTTGTGGTTTCTGTTGTGTTGTGGTTGAGAAATTGACTGAGATGATATATAAGTCGTTCCTAACGACAGCTCTGTTACAAACTTGGGTGCTTTGCTGGTACGGACAGCGGTTGCTCGATTCAAGTACGGGAGTTTCGGAAGCTCTCTACGAAAGCGGCTGGTACAAGCTATCTAAGAGCATCCGGTCAACCATACTGATTATGATTCACAGAGGCCAAAAAGAAGTTCACGTAACTACATATGGGTTTTCTGTAATATCACTAGCCAGCTACACTACGATTATCAAAAGTGCCTGGTCATACTTCACATTACTTCTTAATATTTATAAAAACTAAATATCAATGTTAATAAGCACGATATGTTTATTTTATCCATTTTAAAATTTTGTAATTATATGTGTTATCGCTTTACCATATCCAGCCACTGTTGGCCATCGGCCACCTCTCTTTAAATCATGTGTCCTTTGAGTCAAAGGAACTAGTTATAAACAAGCGTATTATGCGCGCGTTCGAGACGCTGCCTACTACTTTCTGATTTGTAGTTATAACTAGTTTCCATAAGACTACTGTAACCTGGCGCGTATACAGGGCTAGGGGGAGGTGGGGCAATGGGGGCAATTGTCCCCCATCGCATTTGTTTTGACCTTTGAAATGAAATCAT

>SinsOR25

GGATTTTGTTCTTCAAATTTATACGGGACCACCTCGGAAGCACCATTTCTATGCTTTTTCCTCAACTTGCTGCAGAACAGTGAAGAAGGAGTATGACGCTTTAATTATAGCCATGAAGGACGTAAGGGAGAGCGTGGTAAACCCGCCGGCAGTGAGCCGTGCCACGCGGCGTGAGCGCGTCATCAGCACCAGCATGGCGCGCCGGAGTCGCGCTGAGAATGCGTACCATGGAGCAAAGTACGCCGCGCCTGCTATCTCGATGCTCTCCTCCGAAAGCTGGTTGCCTTGATAACAATAGAGAAACACTTGGAACGACATGTTCAGAAGGTACGTGCCCATGGAGAACAAGCGGACGAGGTTTCCGGTGTGCGACACAGAAGCCAGCTGAAAAGCAGTAACGCATATGATAACGAGAGACACGGTGAACTGAGCGAAGGTGGGTGCTGAGAAGCAAGTCTGCAGCTCCGATGCCGCTGTCAGCGCCGCCTGATGTTGGCAAACGCAGTTACGTAGCCTGGAGCTAACCACTTGCTCTTCGTTAGCTGTTAGTAGATGGTTATGAGTGACTTCTACATCTCGGCACAACGTCCTCAGCGCCAGACCCAGGAGCCGAAGCCGGCAACGGCACTGCGCTATAAGTGCCGTCACTAGGGTATCCTTGCCCACATTCAGATACGCAGCAATGAATAAAGCTCCGACCTGATGCATGTACGTTAGCTCGTACGCTGGAGACTTTGATGTGTCGTAGGGATACCAAGCTCGCAAAGGCAACTGGTTCTTCTCAGCGCTGCCAGCCCAGCCGGACACGGTGACGGTGGTCAAGCAGAAGTACAGCAGTTGTTGCAGACTCGTCTCGCGGTTGCAGTTTTTCACAATAATTCTCCCCTCCTCAGTCTGCTGTCCTTTGAGAACCAAATCCGCTTCCGTTATGATTTTTTGTATGAGTCGTTTCCTCGACAATATGTTGATGATCTTCGCGGCTTGCGCTAGGTTCGTGAAGAGGAGGAATGCGGTACCCGTCATCAGGGGAAGGTTGCCCCATACGAAATACAAATCTACTACTTGTATTATTAAATATGTACCCAACAAAAACATAAAAGAAAATAGTGCATAACAGTTATATAAGAACTTGCTGATGCCGTTCAAGCCGTCGGGTGCCCAAAATCCGACCAATTTCAAAGCTGTAAGTGACAAACTAATTGATAAATTAGCATTGTCCACTAAAATCTTCATCTTTATTTTTTTTTTTCAAAAAATACAAATAGAATAGACCTAAGATCTGAACAGGCCGAGTGACGAAATTAAAA

>SinsOR26

TCTAATTATCGTATCGGTTTGAAAATGCAATTCTAGTGACAATAGAATAATATGGTAATAATTAGAGAACAATAGCATAGACAGTAACAATAGATACACATTTATTTCACACACATTACATTCATACAAATACATACAATATGCATATGAATCTAACAAATTAACACCAAGTAACATGAGTCGCAGCGCTGGTTTTCAGCGAGTTCCAATCTGATACACTAGTGAGGTGATACATCTAGGGTTACCAGGTGTCCGGCTTTAGCAGGGCTTGTCCAGCTTTTTACAGTCGTGTTTACTCCTGTATTTTTTTCTCTATATCCTGTATTTTTTTTTCAAATTTTAGGCGTAATCGTTTCGGAGATTAGAAGCGAGGAATGTTCAATTTTCATCTATTTTATACTGAAGTCTGCTGCTGAACAAAGCTGGTATTTCTTCTAGTTCTGTGCGCCAACTGTATGTAAAAGTACTAATCCCCCAGCCTCGAAGAACGCCATGCGTGAGTTGCTCTTGAGCGAGCGAGTTATACATTAATAAATGTCGGTACGGTAAGTTCCGTAAAGGGTCCTGCGGTAAAAACGATCCTTTTTCTAAACTGAGCTATCAGGATGAATATATTTTTGCGATATGAAATCCTATGCATCCACCATGTACTTTCATATGGTCCCAACATGAGGTCTTGACTAGCATATAAAACATCATTGCTATGCCAACAATATATAAATAATTGTGCAACACCGAATACAAGATATTCCGCTGAAAGGAAGCGTTGCATAGGATTCGTTTCGATTGTAATTTGATATGCGGTGACACAAAGCATTACGGAGCATACAAACATGTAGAGAAGCATGATAGGCGACAGGCAGGAATCAAACAATCGAGCGTGCTCAATTAATGCGACATATCTTCTATGGCAATCTTTTAATCGTTCCTTTGAAACCTGCTCAGTTACTGGAGAGGATTCTGTTCCGAACATATTAGCGCAATCTCTTCTTAGCAGCTCCAATTCTCCCCTAAAGAATACCATGATGACCATAGCATTGGTATCATATGACGTGATCCATCCACCACCGTATATAGCTCCATAACTTTGAATCACTGAAGCTATCAAATATCCAGTTATTGTATTCTTATCGAATGGTACCCATGAGCTTACAACTTGCAAATAAGTTTCGTTGCCATTCCTAACATTTTCTCTATAGACTGGTGATGAGAAATACTTTATTATAGGTTGTACCATAACTATTATAACGGTCGTGTACATAAGACACCAATACATATAAGTGATTTTCCTGCAATACTTTGTGAATTTTTTAATAATGGTTTGTTTGATGATATCATTTGTTTTCCTTTGCATAATGTCTGCTTCGATAACATATCCAATGATCTTTTTCCAATCATTCTGCCAGAAGAGGAAAGTGCTGATTTTGCAGACACTGACAGTTGCAAGCATTGTGATCTTCAAATTGTTCAAAAGCAAAGTAATGTTGGATTTTACGAACCATACGTCGACGCATTCTGAAGCAGTAAACAGAATCACAGATAAATGTAATAAAATGTATATTTGTCTCATTATGAAATTCTCTGGCAATAGTAAACCCCAAAATTTTAACGCTTTCAAATTGGGTCCCAATAGTGGTCTTTTTGGGTCTTCTAATTTGTGAAGAAGCCTTTTTAACATCTTTATTTTCTTCTTAATTATATTATGTTAATGGCGATATCGAAATGCGAGTGTGTAGAAATGGA

>SinsOR27

TTTTTTTTTATTTAACTTTTTTATTGATTACATTTTTAGATACGAGTGATTACTACATAATTTGTGTCTAATTCTATTTAATAAATTATTCAAAATACGTGCTCCAAGAAGCAAAATATACGTTTCAATTAAATCGTTGACTTCAACATGAGGTAATACGAAATAGTAGATTTTATTATAGCTACCATGGGTGTTACTCCAGTGCGCAGTCCTCCTGCACCCACAAACGCCAACTCGGTCTGCAGCTGCGCCAAGATTATAAGAAGCGATTTTTGATTAGAAACAGTCCAGTTTTCCCACGATATGCCATATAGAGATTCGGGTAAATCGTCACTCTGCTTACGTATTTCCTCTAAGATGAAAGACATTATTATCAGTCCTCCCATGCAAATTACAATGGTTATACCAAATAACAGTGAAGTTCTGTCTCCTTTTTCCTGGTTCATTAATTGATATAGTAGAATACTATCTATTAGTAAATTGTTAAAATAATTCGCAGAAATATTTATACCAAATACAGATTCGACATCTTTAAATAATTTGATAATAAAACAGTGATATTCGACCAGATCTTTTAATTTCTGTTTCACTTCTTTGGCATTTAGTTCAGTCGGGAAACTTGATACTATTTTGTGTTTCAGAAGTATGACGTGTCCAATTAAATGCATTGCTATTGTATAGTTGATGATATCGGGTATAGCTAAAAAAGCGCACCCTGTACCTATTAAATATATGTTAATAACATGGGTGATAAGCCAATTCTGGAAATCATATTCGTAATCGATAGGCATCCACATGTAAAGGATCGATTGCGTAAGCATAGTTTTGTTATGAATAGCTTCTCTGTTGTTCAAATTGTTGACAAGTGGTATCACGGACCATAAAATCCAGCTGAAGTTGACAAGGAATATGATGCAATAAGCCATCGTACGAGTGTAACGTTCTATTTTAATGATGGTTGCTTTAACAACACTATCTTCTTCCTTATAATTGTGTAAGTGAATATTGGTCATGAATGACTTCATAAGATTTTCATATTTTTTCGTTTTGTGCACTCGTATTTTTATATTAACCAAAGCTGTTATTGGTAGCATTGAAAAAATTGTACCAAGCACATTTAATGGTAGAATTCTGAAATTCTGATATAAGTATATTATCTGTAACGTTAAGCTAAGTGGTCCGAAAATTGGTACAAGCAAGCTGTTGATATACACTATAGTGCCGTAGGGTTTGTAACCCCACCAGGTCTGTTCGCCCAAAGACAACAGTGACATCATTTTAGCAAGCGTACGGTAATATAACTGCTGCGTCGGCGGTTTTATAATATATTCTGCTTCCATATTTGTTATATGCGGCATTTTGTATGCAGACCGTAAAGTCAACTGTAAGTTTACACGTAGCGCTGTTAGAAT

>SinsOR28

GTTTGTTATAAGCTACAGATTTAGTAACATCATGTCCGAGTTAGAAAAGTTAAATCCGGAATTTGTTAAATCCTATCGAATGTTATTATTGTTTCTAAGATTTAACTTCATCGATATAATGAATCAAATGAGTGACATGCCGTATTTTAAATATTGCTATTTATTGATGTTTTCAATATATACTCTTGCTTTGATATGTCATATAAGTGGCATATTAGAAAAGATTTCTAAAGGGGCTAACTTACTTCAGCTATCAGGGGATTTGTCAGCTGCTGTGGTTATTTTACAAGGGTCAATATTGTTTATGAACATTTATTTTAATAAAAATAAGATTAAGAAATTTATACTGAGACTCGGGTTGGAATGGCGCGACGACAACAATTTGCGACCGGAACTTGTGATGCTGAAGCGTAAACAACTTAGAGCTGTTTACGTGTTCACTTTTGCATTTTACACTGGTTTAAATATCTTTGTGCATTCTTATATGATGATACCATTGATTTACAAAATGATTAAAAAATTTATATTGAAGCTTGAAGTTTCGCACACAGTGCCTTTTTATGCAAAAGTACCTTTTAATTACGAAAACAACTTTCTTTTGTACCTCATTGTTTATATGTACGATTATGGAATTATGTATAACTTGGGATATTTAATATCTGCAGATATTCTATTGATAACAGTCAGTATGAACAATATACGAATACTGTTTCAATTGTTAGGAGATGATTTCAAAAGTATTTTTAATTCGCTAAAGAATGGAAACACTCCACGTAGCCACGGTGTGATAGAGAAACATTTAATTACAACCGTAAGAAGACACATAACTTTATTAGAACTGATGCAAGAACTCAGTACTGCATTCAACGGCATCTTCGCAATCCATCTTATATTCGTCTCAGGGACGATTTGTTTCTTTGGATTTGCAGCTGTGTTTGGCGGCACTCTGGAGGATTATAAAAATTTCGTAGCTTCAGTAATCGTCATTGTTTATATATTTTTCTGCTGTTTACACGGACAACACTTAGCAGATTCGGGATTGGATATTGCAACTTCAGTTTATGACAGTGCGTGGTACCAGTTACCAACTAAATACAAAAAGATTATATTAATTATTTTATTGAGGTCACAGAAAACTTATTACATTAAGTCGACTAGCTTTACGGAGATTTCTTTGAAAACATTTACAAGGATGATGAATATGACATGGACATTTTTCTCCTTGATGAAAACCATCTATAAAACATCATAAGAAGGACCAAACATGAATAAATATATGTATTTAGTTTCAATTCTAATTACTTTTCGTTACGATTTTGCTATATGTATAAGGTACAAATAATAAATATCTTAATTAAAGAAACATTTGAGAGCACTTAATCAAGATGAAGAATTTCACAAGAAGAGTCCGTATTCTAACCTACATCCAAAAATTCTAAGTCTATGCAATAAAGGCTTTGTAAGGAAAAGCTCCACGAGTAATAAAACTATCGAAGTACGCCGTGTTCAATTGATAAGGTTTGATGGCTGAGGGGGAGATGAGCGAAATGAAGTCCTCGTCAAGAACTTTATGCCACATTCATAGATGTATCAAAAGGAAGAATGAGGACCTCTCTTCTAAAAGTTTCGTAAAATAGATTTAATGAAGAAATTAAATTAGAAATAATAATTTTAAGGGTATCGGTTACGTGGATAACTACGGCTTAAATTATGTACTACGGTCTCGCACTGTTAAGATTTTGAATACTTAATTTAAAAAAAGTAATATAACAATAACATTTCCTGCTTAGG

>SinsOR29

CAATGTCTAGATTGACCGGACTATTAAGAAGTAAGTCCTTTAAACGAATAGACAGGCGGTTGTATCATTTTAATTTAGCGGTAAGATTTACTGTTCCTATTACCTATGATTACACAATACTTTGTTTAAAATGTGATTGGTAATGTATTTTTTATATATTTCTTGCATTGTAACAACAAGAAAATTTTGAAAATGCATAACACGAGTTGTCTTAGATTATGTCTAACCATAATGACAGTAACCGGAGTATGGCTGCCGACGGCCTTAAAATCTAGCCATTTAAGACATATTTATTATATCTTCGCATTTATGTATCACCTGCTCTTCGTGTTACCTATTATTTTCATGGAGTTCGCTGTCTTTTATCAAGTATTGGGAAATATAGAACAAATGGTGGACACCAGTTTACTTTTAGTAACGCATATCGCACAATGTGTTAAAGTATGCGTGATGTGGTACAAACAAGACGAAATACATAGCCTTCTCATGACATTAGATAGTCCTACTTTTACTAGAGAAGACTCCAACAAAAAACAAATATTAAAGGACGTCATACAAACTACATACACCGTCAGCAAAGTATTTATTTCTCTCGTTGTAGTCACGGGAATATTCTGGGGCATATACCCTATGCTTAAGCCTACTTTATGTTTACCCATCCAATACCCTAATATACCACCTGAATATAAAATATTCCCAATTGTTTATTTATACCAAATTGTAAATATAACACTTATGGGTATCGCTATAGCCTCAATAGATTTTCTTGTTGGTGCATTGATGGCTATATTGTCGGCTCAAATGGACATCCTTAGCTACGAATTATCACTTGTGGGAAAAATAAATAACAAGGACGATAACGCAAACACCGATATTAGACAAGACTATCTAATCATTATTTCATGCGTGAAATTCCATGAAAATATAATAAAGTTTGTCAAAGACTTGGAAAAAATATTTGGCTTTCCAGTATTTTTTCAATTCCTAACAAGTGCGATTATTATTTGCGAAACGGCCTTCAGAATCACTGATTCTACTGAAACAACGGAACTAATTACAATGATGTTTTACTTCATGTGTGTAAACACTGAACTTTTAATGTATTGCTACTATGGAGACCTTTTAAAACGTAAGAGCGAACGAGTGGTCGAGATGGCGTACTGCTGCAACTGGGAGAACGCGGACGTGCGCATGCAAAGGGCACTGCTGCTGCTCATGCAGCGCGCTCAGAGGACTCTCGTCCTCAGGGCGGGCAACATGTTCGAGTTATCCATGATGACCTTCTCTGCTATATTGAGAACATCCTACTCTTACTTTACTGTACTCAATGAAAGGCGTAAATTAAAAGGATAAGTTTTAAATGTACCTACTTGTACAAGTATTTATACAAATAATATTTGATTAAATATCAAGATTTATGTTTTTAGCTTGAAAAATATAGAAATATTAAAACTGTAACTGCATTTATTTTTTAAGTAAAATAATAGAAATATAAGTAAAATAATAGAAATAAATAAGCAAGCATTTATTAAACATTCGCAATTTCACAAAGTAGATAATAAGTATTTTTCATCTGATCTAGAAATTTATGAAAATGTAATTAGAATTTCTAACAAAATTTAATGTCTTTTCAATTTTTCAATCCCATAATTTTG

>SinsOR30

TTTTTTTTTTTTCAAAATTCATCCCCTAATAGGGTCAATTAGGGGATGAAAGTTTGTATGAGAGTTTCGTTATTTTCCAAGTAATAATGACGTAAGTATTTCAGGTTTTATCGAAATTCGTGCTCTTAAGAGGGGTAAATAAGAGACGAAGTCGCAGGCGGAAAGCTAGTATAATAATAGATATGTACACTTATTACGAAACAACTACTAATGTTTTTCTTTAAGCAAATATAAGTCTAAATTGCTTCACCTTGACTATCTTGAACATTTCGTACGAGAGTAAAGAATGAATAAGCCGTCTTCATAATCGAAATAAAAGTCCCCAGACACAAAGGGAATAATTTCAGGCCTGTTAGGGTGAGCGGGGCATTAGCATGTTCCATAAAAAGCCTTAGGTTTCGTTTAAAACTTTCCGACCTCGGTATCCATTCGCTATTGTAAGCGGCAAACACTAACTCCTGGCTTTCGTAACTTAGCTGCGTTCCTAACCACGTCGGCACAAATATTTCCAATGTCATAGCAAATAAATACGACACCATAAACATTATAGTTTCAGTTGTCGAAGACAAAAGTAGTCCACACATTACAACACAAATGATTGCTGAAGCCACTCCGAATTGAATGAACATAGTTACGTTGATAACTTCTTGAACTTTATAAAAATACTTCAATAATACATCATAATGCTTCAGACACTGGTTCAGCCTTGTCATTTGTATGTTTTCTTGAATTTCTACTTTCAATTTTGATTTATTTTTTTCGAATTTAACGTTTCTCAAATTATGATTAAGTAACTTTAGTTGTGCCACCGCCATCAAAATCAATCCAGACATCAACGTATCTATATTGACATTATATATCAAATGTCCAAACATTCCAAAAGATTGATACAGAAGGAAAATCCAGAAATATTTCTCGCGGACTTGATTACTCAAAAAATAATATTTGCAAATAGGCAATTCTAGATTACTTACGTTAAATATAAAATAAATTATAATTGGTAAAAACACTTGTGAAGAATAAGCAAATAACGATAACACTGTAAACATTTTCCAATAGCTTTTGTAATACGAGTTATATTTTAGTATAATGTCTGTACCGATAGCATCTTCTCCATTAAAAATGTCATCATCTAACATATCAAATAGGACGACAAGTTTCTCTCTCATCAAGAGAATCATCAAAACTTTAGTCGTAACCACCACTTCGGTGAAAATAAATATTACTTCTCGAATTAGCAGCTCTACTTTTTGAGGCGTGTAAAATAAGTTGACAATTAAGAGGATATTGTAAATCAAAAGCATGCTGACAAAATATACAATAGAATAATATTTATAGTATTTGTTTGTTGTAGATCCTGGCCATATTCCAAAAATTTTTAACAAGACAAAATTAAACTTAAAGCTTTCTGACTGTTTTATCGGCGACATTGTTA

>SinsOR31

GAAGCAATAGAGGAGTAACAGGCATGGGGATACACAAAAGATCTGTAAGGGTCCAAGTGTTTCCGCTCCCGTTAATCGGGAGTGGCTCCAGGGTGAAGATAAGTAAAGTAAGTAAGTAACTTCTAACGCGCGTACGCATAAGTACATGCACCCATTTTTTAAGTTACACTTCTTTTGCCGCGTTAAGAGTAAAATTTTTGTATGCAACGCATGCAATACTTAAAATATAAAATATATAAATCTGAAATAGAAAGGATTTTCCAATACTCCTTTAAGAAAATTCTAATTTCTTTTATAAGTTAGAAAAACCAATGCTATTATTATTATTAATTACAAAAAAACTATTTATCTAAGAACGTGAAGTCCTCAAATGAGTGAATACTCGATTCACTTATAGATAAATTTTCGTTTTGCTTATAGTCTCAATATGCCTACGTTACGCGCAGATATTTCGCGATATGAATGTAGACATCTTGGTTTTATGGCTTTTTCTGTAGCAGCAACGATTACATTTTCGCCATCGTATGCAAGCTATAATTTTGAAAATATGTCTTCATACTTGATATTGGGGTTCGTATCTTTAAACAAGAGGCTTCAATATCTTTAAGTTAAATATAATGAGATGTAATAAATAAAGTCCATTATATAATAAACGCTGAAATCGAATGTATAAGATAAAATTAATATGTGAAAGTGAAAATCAATAGATTTTACAAACAAATTTTAAATGATAAGGAAGCATGTTCTTCATGTTGTTCAGATATCCATATCTTAATTATATTGTAAACTACATTAGAATTCATAACAAATGTTTGTTGTTTTTTTAATTATAATGAATGGAAAATAGCAAAGATTGAATGCTGAGTTTCTTTTAGTTTTAATATTGCTAGATGTCATTTTTTGAAATGTTGGTACATTTTGCTGTGTATATGTTTGTGGCACATTTGTTTAATTAAGATATTTTAATTTCGATTTTTATTAAATAATAAATAGGTTTATTAAATTATACAATTATTTTTATTTTTAATAATGGATTTCTTGCAGAATATTAAGGTTGATAAATTGTCCGAAGCAAGTTGAAGAATTGGTATGAGGAACTGCAAATCCGAGTAAAAGTATCAAGATTCATAACCAAATATCCATTTGCTGTTAGTTGAAGTGGTTTTTGTGTTCGCATTAATAACATTAATAACGTGCGTTTCGTTTGTTGATCAGCATACAACCAATTCGAGGCGTGTATTGCTTCAGCTAATTGTTCACTACTATCGCTCAAAGACTGCCCCATTATACATTGATCCAATACCGCTACAATTAGTGCAATGAAAAAGAAGAAATAACGCAAAGATTTGTATAAATCATCAACTACCGTAAACGTAAAAGCTGTTAAACAGATGAATAAAGAGCCGAAGGTGTAATTCACAAGCATTAGATCACCTAAGGTTATATTTAACTCCGAGGATATCTTCGCTAAAAATGTATGTCTTTTTATGGTAACATTCAATTTTCTTTTAATATTTTCATGCGTTCTGCTAATATTTTTACGTCTTGTAATATTTTCGATATCTTCAGGATTAATAAGATTTTCGAGACTGTTTCCTAATAATTTTAATTCCATACATATGCAAGCAACAAATGATATGTTAAGCGCGTCTGTAATAGCATACACCAAGATAATGAGACCAGTCATGAAGCTCTCCCAAATATAAACCACGAAATACCAATTCACTTTGTCAAAAGGATACCAGCCGTCGAAAGGCAATAATAATTTTTGTTCTTTACCGACTTTGTTTTCGTAAACCATGAGAATCCATGGAAATATAGTGACAATTGCAATTAACGGCAAAGAAAGATACAATATCAATGTTCTGTTTGTTAATTTAAATATTTTCGTATATTTTGTAATTGTTTTCAGATCTTCAGCTGAATATTCTGATATAGTTTTCCACAAATCTTCACGGAAATGCTCGAATATATCGTAGTAAAGTGCTCTATTAGAATTTACCTTGGCGTATTTTATAGCACACATCACACAGACACCTAGGTTAGGGGAAATGTTAATTATTTCAATGAAATCATTCAAGCCAGTTAAAACAAGCGTAAAGCATAACTGAATCGATGTAAAAACTATTAAGAAATAAGTCGAATAGTAACCGAATTGTTTAAATTTCGTTTTACCAAAAAATGGAAAGAGAAATAATTTAGATAGATAAAAACTCAATAAATGGATCTCGTTTAAAAAGATACTTCGTTCTTTTCTGGGAGCAAACATTGTATGATAGCTGCCTTCAAATGCTTTCAAAGAGTGAAGCTCCCTCGTGGTTTACT

>SinsOR32

TTGTAAAAATTTATATTATTATGTGCTAAATTGCACATTAGATGTATATATTGTATTTGACATTTATTTAATTATTCAATTTGAACTTCAGTTGAAGGCGTATAAACAGTTTTGAGAATAGTGAAATAAGACCATGAAGTGCTTATTATCTTGATGAAACTTCCGTACCCAATAACTGAGAATTTATAAGCTGTTAGTTTCTGAGGTGCATGCGATCTCGTCATTATGTATAAAATGATTTTCTTGGATATTTCGTCGGTTTCATACCATTTGGAAAAAAATGCCGATTCGCCGACCTTCGTGCTCTCTCTGAGAATGTTTTCCCCGAAAAAGCTCATCATGAATATTTGTAGAAGCACGGATAAAAGAAACAGTATACATCCCGGTATTTGGGCCCAGTCTCCAGTCGTTAAATTAAAACCTAGCGCACAAATTTCCAGTGATCCTATTAAAACATTAAATAAGTTACAAGCAGTGAAGATATCTTCTAAATCTTCTGAGAGTTTGATAGTGTATTGATGTTTTTTAACGATATTTATCAAAGTTTTCGGGTTCATGGTTTCCAAATGTCGAATTTCATTATTTATGATAATAAAATTGCAACAAATGTAAGAAGTCATAATGCAGTATAGTGAATCAATTGTTGTGAAGTATAGGACACAGATGAATCCACTTGTAATGGAGTGAATGTAAATAAGCAGCCAAGTTGGCCACATATCTGTCGAAAAAGGCACGATCACTGCATATGGCAGGCTATATAATATTTCTTTCGTGTTGGTATAATGAATGCCAATGAACACCAGTGTTGAAAAATTATACACAGTAATAAGTATCAGATTTAATATTGTGTAATATTTTATCAATTTATTTACAAATATAATATCCCGTTTAACTAGAGTTTTCTTATTAACGTCGCTTGATATGTCATCGTGAAGTTGTTCGAGCATATCTGTTAACTGACGAATTTTCTGCCTTTTAAAACATAGCGTTGAAAATTTTAGAAGCGACAGAACACCGATGCAGGTGCAAGGGGCCAATTCAGTGAGTTTCAATAAATCTTCTGAGGAGATTTTCGATACTAAGAACGCTGTTTCTTCTATGATTATTAATAGCAGGGATATATATAAAAGAAAAAATCTGATTGTCGTTTTTGTATTTTCTAAAGGGATTCCAGAAAGCCAGAAGTTTCTTTTTATTAATTTAAAAGATTCTTCAAAAGTAGGAATTTCCATTTTTGATCACTATTGTACCTATATTTGTTACACGTATTCGTCCTGTAGCGTTAGATATTGAAATGTG

>SinsOR33

GAGAAGTAAAAGATGGAGCAGAATTACTTGTCCACTTAAACAGGTAAGGTTTCAAATTAAGCCATGGGCCTCGTAGACTCTCTGTGGAGGAAATTAACTCAAACTAAAGCTTTAGAGGAATCAAGTGGGAAACTGGAAACTTTGTTCTTTGAATCCGTCTATCGAGTAACCTACTTAGCAGGTCTGTCCTCCAGCGACACACATCCGGTCTACCGCGCTTACAGTATCGTCGTAAAGTTGTCGATAATTCTGTTCGTGAGCAGCGAACTGTGGTACTTGGTCAGCGAGACATCCAGCATGGACAACATTATAGATAACATCAACGTTACACTCATACATCTCATAGCTATATATAGATACAAGAAACTGATGGACCACAAAGATATGTACAAGGAGCTAGCAAAATCTATGGAGTCGCCTCACTTTGATATCTCTACACCTAAGAGGAAAAAATTGGTACAGTTTTGGGTGATAAGGAACGAAAGATATTTGAAGTTACTGCTTGGATTGGGAACTTGCACGCTTGCTGCTTGGTATGTCTATCCTCTTGTGGACGATTTGGAATACAACCTCTCGGTAGCTGTGCGTCTACCGATCGAGTACCGCACCCCCTCCCGCTACCCCCTCGCCTATATTGTGGTCCTGATAACATTCAATTACATATCCTATTTCGTCATGGTGAACGATCTCATCATGCAGGCACATCTGATGCATTTGCTCTGTCAGTTTGCAGTACTTGCTGATTGCTTTGAAAATATTATAAACGACTGTAACGTCGAAACCCAAGGTATCAGCCAGAATAAATTGTTTTTGAGCGAAAAATTTAAGGAGAAATACCTTTGCAGATTAAACGACCTGGTGGATCAGCATAAGTTTATATTACATCATACAATGACATTAGGGAAGATATTAAGCACACCAATGTTGGGTCAGCTGGCGGCTAGCAGCATGCTTATTTGTTTTGCTGGTTACCAAGTTGCAACGACGGTGACGATAAGTCTTACAAAGTTTCTCATGAGTCTTCTATATTTGGGATATAGCATGTTTGAACTATTTATATTCTGTCGTTGGTGCGATGAGATCAAAATACAGAGTGAAAACATACGACTGGCGGTATACTGCTCGGGTTGGGAGCGCGGAATAGCAGCGGTGCCCGGCATAAAAACGAGACTCATGCTGATAGTCGCTCGCGCCAACAAACCCATGATCTTAACTGCTGGAGGATTATACGATCTGTCACTCAACTCTTATACCACTGTGGTGAAGACTTCGTACAGCGCGTTGACAGTTTTACTTCGGCTCCGTCAGGAATAGTCATGTATTGTTAAATGAAAAAAAAATCTTGGCTTAGTAAACACTATTTATTAGTTTACTGCACGCTTAAGAAAATTGCGAAGTTAAAATAAACACACTAGCTACCTAAACGTAATATGACAAACGTTACAAATTAATTGAAGATTGTTTAACGTGATGACTTCTGTAATATTTGTGAATAAGTAAAAAAAAATTACATCTTCGCCATACAGATATAAATAGACAAAATAACATTTACATTATTATGTTAATAAACGAACGATTGTCCGTACTTATTAACATTCACACAAAACATTACCATGCGATGTACTTACTTGAATTAGTCAACGTTGTCATGTCCGAATACAAATTATAAATACTGAGAAATTGACTCGAGCCTCAAAATTCTGGAAGCAACTGTACTGTGTACACATCACTAGTGTTGACGTAGTACGAGTGTTTCAAATATTCAATATGAATCTCGTGGGATATTTAATCACACTTGCTAATTTACAGGTAAAGTTTACACATACGTAATATTATGTTTATCTACACTTAAGCAATATTGACTCTACGAAATAATTAGATATCACAGTTAGCTATGTACATGTGTACATCCTTCTTAATGGTACATAGAATCGTATCCTTTCCGTGTAGATACTATTATGTTTCGACTATTATTTTGTACTTTTTTAGGGATTCGTAGTCCTTATAGTTTTTTTCTATCCAAATATTATAAATTAAATTTCCTCCTTATGTCTAAGCTCTCTCAGTTCCTCATTTATAGTCACTCAGGCATGTCATCTCTGGTTGTTCGATGCTGGCCAATACTGAGTATTAACATAGACATAATCAAGCAGCCAAAATTATACCCGTACTGCTCGTACATTCATAGCTCGGCCTGAAGTGTGGTCTCTCTGATCGGAGACCTTTTTTTAAATATACCAGACCCATGTGCGTGCCTTTTTTTGCACTATGTGTTTTGGGTAAGTCCAAGCGGTTTTGGTCAGTTGGAATTACGTTTCTACATTGAGAGTGCATTTGTCCGTTTCGTGGCGTTACTTACATACACTTTACACACATACACTTTCCACATCCGGACTTACCCCAACGCACATTACATTATATTCATTTCTTTTTTACAAATAAATATCACTTTTTCTTGTTTACAAATAAATATCACTATATAACTGATCGGAAACTACCTTATTATAAGTACAAGTATGTATGTACAAATCAGAACTTGTGCTGAAAAGTGATCGTGCTAAACTGTACTGGGAAAGATCGATCATCATAGGCGGGACTATTATGCCAAATAAACCTGACATCGTGCTGATGAATCAGACAAAATTTAAAATGTTTTTGGTGGATGTCACAATCCTTTATGACGAAAATCTTGTGAAAGCTCACTGAATCAGATAAGAAAATTAAGTATCCACCTAGATCAAGCACATGAGGTTGTAGACATTGAAGTATGACACCCGCGGAAATCATCCCAGTTGTTGTACTTATTGTCCGCCAATGGTCTCCAAGCCACTCCACGTCCAGCCTCACCAAACACTTCAAGAGGCTGAATATTTACACCAAGTCCGTTGTCGAGAAGTTCCAGCGAGCAATCTTGTTAGACAATGCCGTATAGTTCGATAATTTCTCTCTTTACCGTCCTAACTCCGGCCGACTGAACTGTCTGTTGGAGTCAACAACCGCCTTTTTAGGCAATTTTTCCCAGTACCTACATAATAATCACTTTATTGCAGCTATAGTTGTTGCAAATGTTTAGAGATCTTTTAAATTCTAATCCATCCATCACGATATATTCGCTTCCAAGGTTGATTACAGAATGCTCTCCCTCGTTCCTCCTCTAATTTCTTAACTAACGATGCTAAAAATCGACAATATAACTTTAGGTTAGTACTTTAAGAGAAAATTATAGCCTAGGACTGTGTAGCATTAGCAGGAACATTGTAACATAGTATTAGATTTTTTATTGTTTTTAAGTAAAAGAAGTAAATTAGACATGCTAAAATGGCTCTTATTTCTGATTTTAATAAAGTCAAGAAAGCAGTAGTGAGTGTTACT

>SinsOR34

TTTTAAACTTTACCGGTAAGACTAAAAAATTCTTATCTAATATATTCATGAAACTTGACCTAAATAAAATAAGGCAAGCTTATAAATTACCTTAACAAATTATACTGTAAACACAAGGTCATAGTCATGTTTTAAGAAACGGAATTATCAAAAGCCAATAATAATAAATATACGCAGCATATGATTATAGGTTCATATTTTCTTCTCTGTACTTATAAATATTACTTAGTATATACACGAGTGAATATGTAACAACGTATTTATAAAGACAACACATAATTTTCCTTATCATTCGGATTTTAACAAGAGAACTCATAATACATATTTTTTTAAATGGAGCCTTTGAATAAATTCCCATTGGAATATGTGAAACCATTTCTAAAATGTTTCACTTTATTAAATGGCTGTGGTATTAGAATTTATTCTAATTACAGTACGTGGTCTTGGAAAGGCTTTTGGCATTCATTGTTTGCTGCGTCGTGTTCTGTTGCATACTCTGTATCACTGATAGCCTATTTTTGTAACATATTTTATGGGGATTTGGAATTTTTTCAATTCGCGCATTCAGTACCAGCGTGTTTAATTACTACACATGGTATGCTGAAATATTTGATTGTCTTATCGAATAAATCAGAGATAAAGGCTTTAATTGATGATCTCGGCACTATATGGAGGAAGACAGGTTTATCTGAGGAACAAATTAATAAAAAGGATTTATTTTTGAAGAAATTGAATTTTTCTAACTCAGTTTATTACTGGATACACATGATGGGTATATGTCAGCATTGTTTGACTCCATTATTTGAAACTGTATTTCGTAAACTTATTTTGAAACAAGAATCGAATTTTCTATTAGCATTCGATTGCTCTTACCCCTATGATTATACCAGGAATTGGTTTGTTTACATTGGGACATATGTCTTTCAGATTTACGCAATGAGTCGCCTTTGCTACATTTATATAGGTGCTGAAATTATAGTTGTTACACTAAGCGCCCAACTTACCATCGAATTTATGCTATTGCAAGACAATCTACATCGCGTCAGACCAACGTTCAATAAAAATGAGACACGTGACGTCACATCAATTTTGAATAGTAACATGCGACGTGACGAAGTTGAAGACTACGGCATAAATGATATTGTAATAAGACATCAGAAGTTAATAATGTTATGTGAACGGCTGGACAATAGTTTCAACAAGATGATTTTTATAAATTTACTCATAATTACTATCACTATATGTTTCATCGGATTCATTATCAAATTCACTCGCCATCCTTTAGATATGGTAAATTTTTTCGTCGCTGCTGTGGCGTGCATAGTTTCCATATTTCAGTTATGCTATTACGGCGAAATGCTTTCGAGAGCTAGTGTAGAAATAGCAGATTCAGCTTATGAAAGTTTGTGGTATAAATGTAATACAAGCCATCAGAAGGCATTGATGTTTATTATTTCAAGAGCTCAAAAACCTTGTAGCCTGACCTCACTCAAATATGCACCGATTACTCTAAATACGTTTACTAAGGTAATGAGTACAACTTGGTCGTACTATTCACTCGTTAGTACTGTCTATGAAAGGGAATAAGCGCAATTATAATTATTAATGTATTTAAATAAATATTTGAAACTTCATTAATATTTATTTATAAAATATAACAATATAAATAAGATTTTGTTTTAAAAAAATAATTATTATGATAAAATGTTCATTATGCTTTCGCTTCTTTTTTTGCTTAGAAGAAATAACTGTTATTGTTTTTATTGTAGTTATTATATCCGTATAATGAAATAAACAATATTCGTCATTAAAAAAAA

>SinsOR35

GAGCTGACACGCACATCTTTTTTTCTGTGTAGACAAAATTGCACATTGCGCTATTTACAATGCAATACTTTTGAAATTCATCTACAAAAGTGAGTGGTGAGTCGAGGACAAATCAAGTTCATGTACAGTAAAGTAATCTAACAAATGTTATATTTAGCAAAATTGTCGAAAATAGAATATGAAGTTAAAAAAAAAAGTCTAGCAATTCATACTTGTGCTATTAGTTTTCCGTACTACCGGTTGAAATAGAATTTATAAGTATCTACTCGTATTTTAATTATCAATAAGTTTTTCAAGTAAATAGTTCCAAAAGCAAAACAAACCACGTCGTAAAAAAAAGAAACTGTTCAGAATAATTAATATGTAATTATTCTGAATAGTTTCTTCCCCGATCCCGATAATTTTCATTCAATCGACACCATATTCTCAATATTCACGTTTTTATTTTTAAATTCTCAGTGCTACTTTTGGAAGTAAGGTGGGGGACAATTCGTAAGCTTTTTTCGAAAATGTGTTAAAGAAATAATACAAAAAGTAATGTAATATATTATACATTTTTGAATTCTTGTTTCAACTAATTTTTTATTTGCAACCAACACGACGACCTCGACGTTAGATTTTAACGTCCCAATTAACCTTCCAAAATTAAACCCAAAGTCCAAATTTTACTTTAAATGTTCACGTTTGTATTTGGGCCATTACTTACTAAAATTTGTTCTCAATTCCATTTGATTACGTTAAATATTCTCAACACACGGTGTGGTAGAATAATTATCATTGAACACATCAATACGATGAGGCATGTAAATAATGAATTCTTGGAAAAGATTAACACCGTAAAACATCATGACGATTTTCTTTATAGCAAAAAGTGATTTTTTGTGTTTAGTTCTTAATAAAAGAGAACGTCTCGCTAATTCATCGCTTTTATATACTTTCTTTAACACGGTTTTTACTAATGACCTGCGAAAATACGAATAATATTATTTCATAGATGAATTTTAATGCTAAAATATGAAATGATTTTCTTAATTAAACAGTAGGTTTTTTCAGTAATATAAACAAAACATTATTATGTATACAATACTTTTTAAACTATTCAAGGAACCAAAGAAAGGTTTTGATTTTAACAGTCTAAGTACATTATGTTTGTATGTCGATCGTAATTCCTGTTCTACTAGAACCAAACATTAAAAACCTTATCTAAACCGAACATATTGTATACTATCGAATATAGTCCGGCCGGACCACGGATTTGGTTTGGGACATCAAATGAGTGGATTTGGCAACGCACAGCCAGATCTTATTTTTATTCATTATCAAATTGTTGAAAAAATCAAAGTAGGTAGAAGTCGAGCTACTACCGCTAGTTTTGTTCCTGAATTCGATCATATCACCGATTACATAGTTGACCGCGACCTGAAGCGCGGAGTTGGTAGAAAGCGGATCAAACCCCCGTATTTTTACATATTTATGGCTATCAACTATAGCAATTTTTGGGCGTATAAGTCCATACCCACTGATTCACTCAAGCTTATTGCTATAGTGATCCAATTATACGCTTTGGTGACTGCCAGATTTGATTATCTATTGCAAACAGCGTATTCGGTCCGCCGAACAGAATCCGTGGTCTGGCTGGACCATATTTGATTGTCTGTTATTATTATTGCGTTCAACGATTTAATTTATATGTCATCATTATATCTATCGACCAACAAATAAGTAATATCCGACCGAACTTTCGGATTTGGTTTCGGTTTCAGCAGTTTCAAAATAAAACTGGTGTCTCGGCTTAGTTTCGGTTTCGGCAGCAAACCTGCCGAACATACATAAAGCGAAACTACGTTTGTGAGTTTTCGCGATGTCTTCAGTGGCAATCGGCATAGCAGGGAGCGTCGATCGTCGACTTCACACCCTTCTACGGCGACACTGCTAACTGTTGTTTTCTAGTGTGAATTTAGTGGTTATATGATTCAGATTGCATTATTTCCAATCAGCTAAGTTGTAGTTATTTGATTGACAATCAACTTTATTTCTATAATTGTATTTTTTCTTTTTTATTACTTGATGTAATTTTGTGACCCTGTCAATGGTTTATACTGTTGGGTCTATGAATAAAATATGTGCCATTTATAATGATGGCTTGATAAATTGATTTTTGTTGTTATAAACTCGAATGTTCCTACTTTGAATGTCAAAGAGACTGATAATAAATTATGTGATTTGATATTGGTAATAAGTTCTCATTTTTATAATGATTATTTTTCTGGGTGAATTACATATCTCACCTCACCCCATACCATATACAGTAGAATTAGAGCAAGAAAACTATTATTGCTTACATATATTGAACACAAACACATATTTGCAACAAACTAAAAGAGAAATGTAAAAAATGTAATCATTCTTTTTAAGAATTATCCTAAAAGGATGTTTCGGTTTCGTTTTCTACCGAAACCGAGACAGAAACCAAAATACAGTTTCGGTTGTAAAAACAACTTGCGATCGGACATATTATAGTAGGGGAGCCCAAGATGC

>SinsOR36

TTATCGATCTCAAACGTTAATGAGTCAAGTCAATAATTAATAGAAGTTAGAAATGTGTTTGGTCATATATTTTCTATATCAATGAATTTGGCAAACAAATTTTCATCATAATTGGTCGTTAATTACAATTCTTCAAAGATTTAAACTTAATGGACTAAGGGCGTTATTTCATTCGATTTACTATAATTTAAAAATTTATTTACCATATTTTTCTCTGTCCTACATAAAAACGTTTTATACGATTTCACTATACATAATATGTATAGTGAAATCGTACAAAACGCCCCAAAACTGCGTAAAAACTACCCGCGTACGTGTAGTTTTTACGTAGTTTTAGCGCGATGGCACACACACAATATATTATGTAAGGTGGCACACATTCCCGGCCGGAGCTCCGGTTGAAATCGCCGCCTCCGGCCGTGTGATTTCGGGCTTAGTTTGATTCTTACCTGAGTATTTACTAGCTGGATCAGGATAAAACTTATCCTCTGTCCTTTTCTGTCACTATAGCTATCCCTATATACAGCTATGCAAAGTTAACCTATTGCTTTGTTATAGCCGGTTTTATGCACCAATAAACAAACAAATATACTTCTATATTAGGATAGTAAACACGGAAATTACAGAAAATTATATTTAATTAGGTAGGTACTTCTAATTCCTTCAATTTAGTTATTAAAACCCTGTAGTTATTCACATTCACCTTTTAAAATTAAACATTAAAAAATAGTCATATTTTTAATTCACCACACAGTTTTAGGCATATGCAGCTACTGTATTTTCATTTTACTTTCTATAAATTATATCAAAGTAGCACAGGAACACAATATACGTTTTTGAAAAATGAAAGTCTTAAAATGTTTAAAGAATACTTATTTGGATATAAAGAATCATCTCCAAGATGATAGTTTCGATAGCCTATTATGGCTTGTAAATATAATGCCTAGTTTAGCTGGTTTTTCTCTACGCCGAGATAAAATAGCTGGTATGTTAATCACTTCATTACTTTTTTGTATTTACATACACTTAATTATGTTTCTATACGAATTTCTCTATGAAATCAGTGCTTTACACATATTATTATTAGTTGACTTTTATAACACAGTAGGGTTATAAAATATACTAGCCATTACTCGAAGTTTCGCTTAGGCACAAAATGTTTTTTTTTAATTCCCGAGCAAACTTGCTCCATCAGGATTAAAATTATCGGAAGGCATTTACATCGAGCCTTTGCTCCGTTATCGTGTGATTGTTAATATTATCAGCTTTGCCGGCCAAACATGTAAATAAATAGACTTTCGCATTTATGATATTAGTAAGGACTAGAGAATGCTAGTAACCGTGACTTCACCCGCGTGGACTAGTTTTTTTTGTCTACGTGAGGGAATTACTTGCTAAATCAAGATAAAAAGTATGCTATTTGTATCTCGATTTGAGAAATAGAGAATACAACATTTTTTTTTGCATTGGAATAAATATGGATGTATGTATTCGTGTGAAAATTTGAAATTGATTTATTGTTGCGTTAAGCGTTCGATCGTTTCGATCAAGAGCGTTTTGGAGTAAGCCCGTTTCGTGTAGTCATACTTATGGGTACTTGGCACTTACATTTTTGTTTTCGAGTTATCAATTTGAGTTTTTTAACGATCACATCCATTATATAACTTTTAAAACTATC

>SinsOR37

TTTTTTTTTTTTTACATTTTCACAAATGAGTTTATTTAAAGTTCTACACAAAAAAAACTACATGATGCACTTAATGCCATGAGGAATTCTCTTCCAGTCAACCATTGAGTTGAAAAGAAATTAATGACAAGGTGGTGTATAAAGTGGAATTTTATCGAACAAACAGAACTAAGAATATTTGGTAATATTTATTACAATATATGAGTTAAAATTACAAATAATTATAATATCGTCCGTAGGAATCAATTGTGGAATTTCATACCATTTTCAAAACAGAATTTTATATGTAACAATATAAATTGAAATCGTCTGACGAAACCTATCTATAAAGATAAATTGTATAATTCTCAAAATTTCAATGGCTCCTATCACGGAATTACCTATTGTAAAACCAATGTATCCCGCCGACAACAATACATATATGTATAATAAAATATTTGATGACAATTATGTGCTTCTATAATATTTAATACACTAAATCTACGTGCTTATGTATTTAAAAAAAATTAAACGATTTAGCATTTATTTTCAAATTCAATTTCCTTTGTTACTCAACAATGTGTAATAACTATAAGCACCTTTCAAAATAGTAATAAAAGTGGATGTAGTAAGCTCCGTGAACGGTCCAGCTTCGAACACTATGTTTCTTCGTAACTGACCACCGAGTAACACTAAGGAACGGCGCGTGCGCACATTTGCCGACCACCAGTTGCTTGCATATACACCCTCTTGGACTATCTCACTCACAAATAATACGTCGTTGCTATGCCAGCAATATAAGAATAATTGTGCTATTAGTGCCACAACAAACTCCGCTATATAAAGTTGCTGCATACCCGTCGTACCCTCTGTAGTTAATTGAATTGCACTGCCACATATCATCAACGAACAGATGATAACGTACAAGAACATAACAGGTGATAAAAGAGAGTTCAAAATCTTTGAACACCTGATCAACAGCAGGTGGTGATTATGACAATCACGAATTCTTTTCATCGCATCTTTATGAGTGACAATCTCGACGCCATCGCCGAACAATCTTGCACAATTCGCTTTCAATAACTTCAATTGCCCGGCGAAAAACGACATTATAACAAGAGCATTAGAGTCGTATGTCGCAACGATGCCGCCTCCGTGGTAACAAACGAATGTAGCCTCTAACACTGTGAACCAATATCCATAACCGGATGATTTGTCGAACGGAACCCAGGAATCCATTATTTGTGGATAAGGCATTGTCTCATTTTTTATATGTTTATGATGTTTAGACGATAAAAAGAAAACGAGTATTGGTGCAACAGTAAGACTAAAAACGGTCATTATGGCCAAAAACCAATACAAGTATGTGATATTTCGCGAATATTTTATGTAATCCCCGATAAAAGTTTTTGTTACATCGTCTTTCTCGGACAACTGACGTCTCTCCAAAGTTGATACGTATTCAATGACATCTCTCCAGTGTTTTTGCCAAAATACGAACGTTCCCGCTTTTATAACACAGAAGGTAGTGACAACCGTTTTAGAAAGATTTGTAAGCACCAAATCGAGGTCCGATTTGACGATCCAAAGTTCTACATATTGTCTCATAATGAATAATGCTAAAAATATGTGTTTTAAATTGTAAATAATATTGGCCACTCCTTTGGTCGGCTGCCACAATCCGAAGGCTTGGAGACCCCACAAAGTGGGACCAAGTGAGGGATGATCTGGGTTTTCTAATTTAGACACAAAATTTTTCAACATTATTAATTGAAACTAGATATGTCTAACGTTCAAAAGTATTGTATTAAATGATGTTTTTTTCATTACGACTAGATGTTATAAAATCATTATAATTTTAATTACTACTTCGAATGCTA

>SinsOR38

GTATATTGTCCCATATATTTTTCACTTTTGTAATGTGACAATAAACTAGTTCTGGTTTTTACGACCTTTGTATCCGGAACAGAAGATATAAAACATGTACCAAGTTCGTGACAAAAAAAATGCTGCTACTTTTCTTTGTGTTATAATTATAGAAAAAAGTTTCATGATTCAAAAGCATCCAACTTCGACCAAAATATTGTCGATATTTCTAGCAAACTATTGTAAGACAATTCCACGTTCAAAATAAAGCAATTTGGTGCTACCGTCAAACGAATTTATTGTTACGCCAGTTCCCGCGTCTATACACGTACACGTAAAAAGAATTAAATATAATATAATATATGAAATATGATACCACGTGGATAATGGAAATGTGTATCGTATACTAACATTAACCTTTATGGATAGCTACAATTATTATGAGACGATATTTATTTGTAATATTCTCTTGCATTCATTTGCTATTAGACGTGTAATCATTTGCACATTTTTATTCTGTACAAAATGAAGAAAATTCTCAAAAATGAAACTACTGAGGTCCAATCACCAAAAGACCAATTTTTTTATCGGGCACTGGCCGCGCTAATGACATTCATGTGTTTGGGAAATCAAGTCTGGTGGGGTTACGAGCCTTACGGAAAAATTTTCAAAATCAACAGATTTATAGTAACATTTACCGGTCCAATCATGGCCATCTCGCAGTTTATGTATCTATATGTATATTTCAACCAATTAACTGCGGATGCGCTTTCTATCGTTTATTGTATGTTACCTGTGACATTATTAGCGAATATAAAGATTAGACTAGCCAAAAGAGATATTTATAAAAACCTCATGCTGGACTTTATGACGAAAATTCATCTCTACAATTACAAGGGCGAAGAATTCATAAATAAAACTATAAAAAAAGTGGAGCGTTATAGTCACCAGATGGGGTATTGCTTGATAGGAATTGTCGCTTTCGACTCCTTGCTGTGGTGTATAGTTCCAGTTATCACTAATTTGATTCACGAGGAAGCTATCAAAAACAGGACCATGCAAACGCAGACCTGCCTTCATGTTTGGGCTCCTTTCGACTACAGATACGATTTTCATAAATGGCTCATTGTTCATATTATTATAAACGTATACGTAGTTGCACACGGATGTGGAATATTAGGTATTTTCGATGTCGTATTTTACATCATTGTGTTCCATTTGATTGGTCATATTAAAGTTTTGAAATATAAAATCAAAACTCAATTTGAGGGTGATCTGGATGATGAGGAGGTGAAAAAACGACTCGTAAACGTGATAAAGTATCATGCGTTTATTATAAAGTTTTTCAAAGATGTGGAAGCTGCTTTTGGTATTAACGTTTCGGGGAATTATCTTAATAATTTAATTGCGGACAGCCTTATGTTATACAATTTAATGATTATAGCTCAGGATAAAGGTACGGTAATCATATTTGTCGTGATGACAACAGTTTGTATTACCGAACTAATTTTGATGTCATTTATATTGGAAGAAGTCCGTATACAGAGCGATGATCTACCAGAATTAATATATTTCATGCCATGGGAAAATTGGTCACTGAGTAATAAAAAAATGTTAGTGCTCATTCTTCTACGAATACAACCAGAGCTGGCCTTTGTGGCTGCCGGTGGCCTCAGAGCTGGAGTACGGCCTATGACATCAATAATAAAATCCACCTTTTCTTACTACGTCATGTTGAAATCAAGCATGAGGGAGTGATAAAATTTAATTCGATACATATTTTTCCATATTTCATTTTTTTGGCATTATCATATGTTGCGCCCCACACCAACCCACTCCCATCTTCGTGGCGATGAGTGAAAACATGAAATAATTTTCGAAAGCACATTCACGAATAAACAAAACAAAATGCACTTGCAATATTAATTTATATTAAAACAAACAGATTTATATTATGTAATATGTATACAATATATTCTGAATGAATATACAACAATAGAGATGAAAAAAGAAAAAATATTCAAATAATTTGTTAACACGAACTATCCAATAAAAATATTCAGGAACTTAATGAGCGTTTCATTTTGATACTTTAGTCTAATTTTATACTTGACTAGCTGCTACCCATGGCTTTGCATGCGTTTATATCAGTTAAATTGTTTAAAATA

>SinsOR39

CCTTGAGCTGTCACCCGGACCGTGCTTTATTATCATTTAAATTAGCGCCTATTAATAGGCTTTACTGCACTGTTCAAATTAACCCTAATCCCCTGCAAATGTCATAAATTAGACAAATATATATCACAAATGCATACATTTTCTTCGTAGTCACATCGAACTACTTTACTGAAAAAAATACAAAATAATAATTGAACATCCTTGGATAAGTCGATACAAAAATGGCCGAGCAATTGCATATAGATAAATTATTACAAAAAATAAAATTCTTATTCCGATTCAATGGTTTAAGTCTGGAGAGCAGGAAAAGAACCAAAGTGGAGACCATCATTTGTCGATGCTTATATGTTTTCAATTTTTTATGGCTGAACATGGACGTACTTGGTGAAATAATTTGGTTTCTAGACGGAATAAATAACGGCAAAAGCTTCCTTGAACTCTCATATACCACGCCGTGCGTGGGCATAAGCGTGTTGGCCGATTTCCAATCCATTATACATATACTCAAAGAAGATGCCATCAACCAATTAATTGAAGAACTAAGAGAACTTGAAAAAAAAGAAAGGAACCTTGGCAAAAATTCAAATGAATTAGAGTTAAGTAGAGAGTGTGAGAGTGAGTTAGAGAGATTAGAGAACGTAGAGAGAAAAGAAATAATTAAAGAAGAAACAAATATATGCAACTTCGCAGTGATGTCTCAGATGTTTTGGATTTTCATGACGAGTTTAGTTTTCACTCTCATCCCTTTGATTATTACGGCGGTCAAATATTTTAAAACGAACGAACTGGATTTGATTCTGCCGTTCCCTGTGCCTTTCGATGTAAAGAAAGCGAGGTACTGGGCTATCATGTACATGCATCATGTTTGGTCATCTTTCTTAGTAATACAGAACGGAACGGCATTTATCACCATTTTCTATATTTGTTGCTCGTTTATACGCATACACTTTCGACTGCTGAAATACAATTTTGAAAGAATCGTCTCAGATGTCGACGGCTGTGAAATAATTTCCAATGAACATAATAAATTCCGATGTAAATTAATAAATTTAATTAAATATCACCAGGATATTATAAGTGCAGTAAACTTATTAGAATTCGTCAGCAGCAACATAATTTTCTGTTACTTCACTGCGAGTTCTGTGCTCATATGTCTCACTGGATTTAATCTTACGATAAGCAACGATATGATAATTGTGCTAACCTTCGCGGTGTTTCTCCTGTTGAGTTTGACGCAGATATTCCTTTTGTGCTACTTTGGCGACATGATTGTGAGTTCGAGCATGGAAGTAAGCGACGTTCTGTACAACTCTCATTTGACTCTTGCAGATCCGAAGCTGGCAAAACTATTACTTCTTGTACAAATCAGAGCGCAGAAGCCATGTAAGCTGACGGCCGCAAACTATGCTGATCTCAATCTACGAGCGTTTACGAAGATACTGAGCACGTCTTGGTCGTATTTCGCTTTGCTGAATACAATGTACTCTACAGATATATAAAATTTATTGATTGGTTTTCTACAAGGTATTAATTTGTATCTTACTTAGGAAGACGTAAGTTTCATCAAAATCAATGCAGTAATTTCGGGTAATAAAATGAACGAAGAGACAAACAAAAATCTTTGTTACGAATATTACGACTTTATTATGGTAGGTATAGATTTTGCAAATTTCAAACAAGCACATGTCTAAACTTTTAGGGTTCCGTACTAAAACGTAAAATGAAACCATTAAAATATTTAGACATTCTTATTTAATCTAAATATTTTAAAACCTACGCTCATCTGTATAATTTAGTTGAAGTTCTATTAATCATAACTAGAAATTCTGCAAACGGTCGAAATTCGA

>SinsOR40

AGTTATTAACCGACTTCAAAAAAGGAAATTTTCAACTCGGCTAATATTTTTGAAATGAAAACTTCTTTAGCACACCATACACATTTTTTTGGTAATGAACGAAACTGAGCCGTCATGCACAGATTGGGCAAACTCGGCTCCAGTCGACTTTAAACGTCGTTTAATTATGTATGTATATTTTAGAATTAGAAAATCGTATAAGTGTCCACCTCTATCGTGGGGTCTTATGCACATGATTTTTTTTTTCTATTTTATTTTGTGCTGTTGATTAAAAATTCTAATTTTTAGAAGAGGAAAGAAGGAGCGTCGCTTTAATATAGGAAAGAAAGAAAGAAACTGCTGCGGTGCATATTTTAATATTCTATTATATACTAGCTGTAAACCGCGGCTTCTCTCACGTGAATTAAGTCATACATTTCGTTAATACAGTTTTTTTACTATCCCGTGGGAACTTGTTGATTTCCCGGGATTATTATTATTGGGACATCAATGCCACCTAGCGACACTCGACAGAGTTCATGGCGGGCGGCGGCGCGGCGGAGCAAACAGCCGACATATCGCTTCGTATAATGCTGATCTTAGGTTGAGTATATGAGGCGGTAAGTCATTCCAGCAGCATGATCGACACAAATCTGAAACCGCCACGAAACGCAGTAGTCCTATGGGTGAGGATATGCAGCATTTATGAAGAGGCTCTGTGTTAAATTAATTTTAGAAGCGAATATCTCGTAATTAAAGTGTTAAACTTAACGTCTCCGAACATTTTTATTGCGTTTTCAAAAGCGTATCTAATGACAAACCACTCATAGGATAATTGCGTTTTTTTTTGTATTGAAACCCGAATTAAACCGAATAACCCGGAACCTGCATAAAGTTTATTCACATGTCCGTCCATTTGTCTTCTATCGTGTTAACATTTTTTTGTTCGAAATCATAAATCTACACATACTCTTCTACCATATTATGTATGTCTAAAATAAGGTGTTTGAAATATAATACCAAGGAAAAAAGCTTATATTTTGACATATTAAGTGACTAAGGAATTTTTATTAATTAACAAATTTGGACTAACGAGGCCTACGCGCATTGACCACGCCTCTATTTCGCCTCGCGCCTAATTAAGGAAAAATATATTTTATAATCATGAAGAAAAAGTAGCTCGAGCGTTAATTACAACTGCCGCTGTAATCGCACGGAAAGAAATTTACAAATTTAATTTGTCTAACGAACCCCTATACAGAGCGCGACCTTCTGCGAGAAACTCAATTAAGCTCCATGTCAAATGGAGGTGATAAATAAGAGATTTAAGATAAGTAATGTGCCAATGCCTGAAACGAGTTCAAAGAGTTCAGTTTGAACGATTGAAGCGGAGGTATTAAATATATATCCTAAGAAGACATTTAATAATTGATTACAATAATTATTTCAATTTAACGTCCAGCTCGTAGGTAACAATCCAATACTAGTCAGTGTCTGTAAAATAATATACGCGTATCCTCAAGATTACAATTTTTCGTAAATATACTCGCAGAACACGAGTTCTGACATATAGTACCGGTACACTCTTCGGAGCACGTGATCAGTTCGTTCAGCAACAGAATATATCGCACAGTGGATTATATGGATTCTATTGCGCCACGACGACCGGCGATCCAATCCTCTCCGCGGATTAGAACTCCACCTTCGCTTCCCGCCCAATTTTTCCTCCTCGCATTTACTCACTTCAGATCTCGGGCGAAATACATTTATTTTATACAATGAAATACTTTCGCTGTTTAATTTTTTAATGTACCTAATATCTCTACTTAAACTTATCCTAAATGTTTCTCCATTCTCCATTCCTCTCTCATCCATTCTACTTTGTCTGTCTAAGTACAGCGTAGAAAGTATAAGATGATTTTAATATAGTGACGAATGTTTCGAGAGAGAGCGGTATGATGAGCCCGGCGACAGGACGCAGCGGGCGCTTGGCGCGCTCCATGGCCAGCACCAGCGCGCGGCGGAACGCGACCGGCGTGCACAGCCACTGCATCGCGTACAGCGACGACATCAGCCGGTCGCTCTCAACTGTGACCTCGTTCCCATAAAAACAGTAGAGGAATAATTCGGTTAAGATGCAGATGGTAAAAAGGATCATTGAAGCGAATTCGATGCTCAACAAACTCAGATCTACTATCTTATAAGCTGTCATGCAGAGAATCCAGCCGCCTACACCGAATTGGACAAGTATCGCACCACCAAATATGTCCTGGAGCAACTGTGCCGTCTCACTTATCTTTTCATAATGCTCTATACTGTTTACAAATAACCTCATCAGTGCCTTCTCGTATGGTTCATTTGTATGTTTCTTTAAATCATACGCTCTCTCGGGAAGAATCTCTAAGTCCATTCGTAGGATTCGGAGTTGTGTCTTGCACTGATAGAGAATGGTGCCCATGAACGCGTCCATGGTGGTGTTGGCGACTGCTACTAGGGTCGTTACGTAGAATGAGTACAATAACGTTGCCACGAATACTATAGTTGAACTATTATAATCGAATGTTGTCCAGAAAGCAAAATACACGGTGCGGCCTGTGAGTCGGTGCATGATGGGGAACACGATCCATAGGATGCACGTCAAGATGGCGGTGTTGGAGTAGACTCGCTGCAGGCGGCGCGCGCTCGACGCCGTCGCCGCCAGCAACCGCCGCCGCGACGGCTCCTCTGGGTTGTACATCGTGTTATCGAATTGAATAATGAGGTGGTCGATTCGATCGGCATCAACGTAAAACACGATCTGTTTCACGAGAGAGGTGATGTGACACAACAAGAGGAACATCACGCGCGCCAACATCTTCATGTCATTCCGTGCCTGGTAAGAATAGATGATCTCCTGAATAAGATAAACCGTTGTAACGGTGAGTGCGAACAATTGGTAGAAGGTGTGCGTGTAGCGCAAGCGGGCCGAAGCCGTCGACGCCTGTCGGCAGAACCCACAGCGACGGAGCACGCGCAGGTGGGGCGCCACACTGCTGCCCGCTAGCGACATTTTCTCTTCGGCTACGAAGAGAAATGCAAAATGAAAGAAATAAATAAAACTGGGTGTACATAAATCTGACAGTGAGACGTTTAATTATTAA

>SinsOR41

TTTCATACGGTGCGTTTTTGGGCATATAAATCATAATTATAGTAATTAATTTTGTTTGTGTGAATATAGGCATCACTGCTTTCACATGCCATGTTCTATTGCGGCTGGCATCTGTGTGACATGGACAAACAATCTCATCACGACATCCGACGGCTCGTGTTAATCGGTTGTGCGCAAGCGCAGAAACCTCTCATACTTAAAGCTTTCGGCATCCAGGACTTGTCCTATGAGACTTTTGTTTCGGTGGCGAGAATGACATACTCAATTTTTGCAGTATTTTATCAAAGGGGAGAGCAAAATTAAAGTATCTGTCTGTTCATTAAAA

>SinsOR42

AAAACGAATAAGTATTATAAATGTTCTATGCTTTCCCCCTCCTCCCTTTTTAAATTAATCTTTTGATCCAGAACATGTCTAAGAGCACAAAGTCTATACTGAGGGCTTTTTGTAAGTATGTTTATTACGCCGGAGCCGGAAACTGTTGGTACGAGGATACATACCGAGAAACATATCTGTACAAAGCGTACGCCCTTATTTCTTTTTCTATTTACACCACAATGATTTTTCTTGAAAATCTCGCTGCTTGGTTTGGTTCTTTCCCAGAAGTGGAAAAAAATTCAGCTGTCATGTTCGCCGCCATCCACAACATCGTTCTCCCTAAAATGTTTCTCCTGTTATATCATAAGAAGTCTATAAGGATATTGAACTACGAAATGGCTACTGTAGGGGAAAAAATCGAAGAGAAATATGTCATGGAGAGACAGGCCAGAAAAGCGAAAGTTGGGATTATACTATACGTGATATCGGTTTACCTTTCGCTGGGCGCGTATGGCGTGGAAAGTACCCGGAAAGTGATAGTCGAAGGTGCACCCTTTTACACTGTAGTGACATATTTGCCTCAATATGACGACAGTACCATAGTGGCATCAATATTCCGCGTAGTTTTTTACATCACTTGGTTATATATGATGTTACCGATGATGTCAGCTGATTGTATGCCGATTACACATCTGATTACTATGACGTATAAGTTCATTACACTGCGTCATCATTATCGTAGAATCAGAGAAGAGTTTGATAAAGATCTCCTAACGATGGATAAGAGACAGGCCGCCGAGAAGCTAAGAGCCGGTTGCATAGAAGGTATATTGATGCATCAGAAACTAATGTTTTTGGCTGACGAAATAAATCGTATTTTTGGAATAA

>SinsOR43

CAGTCATTCAGCAAATATGGACGATATGGAATTGAGAAAAACACATCCACAAAAATATTACTTGAAATTCATTTGTAATTCTTTATACGTTTTAGGATACGGAAGTTGTTGGTACGAGGAGACTCCTCGAACCAATTTCCACAAAATCTTCTACAAAATATGGTCAGGAATTGCAAATTTCTTCATAGTTATCATAGTAATAAATGAGATAATGGCAAATTTCAGACCAAATTTGACAGCGAAAGAGCAGAATGATTTGGTACAATTCACATTTGGGCATAGTCTTATTGTAGCGAAGATAGTAACTATGTATTATCAAAGGGATCGCATCAAGGCTGTGCTAAAAAAGCTTCTGGAAGATAACAGGACAATATTCATTTCAGCAGACATAGATAAATCATCGGTAAAGAAAGTGAAGGTATACTGTATAGTTTTGGTTAGTACAGTATATTTAACTATAGTCTCTGCATATATTGACGGTTTCAGAGCTCATTTTAATGAAGGTATACCTATTCGAGGTGAGATAACTTACTACCCAACCCCTTTGGATTCTGGCATACTCGTGAACATACTACGATTCATTGTGGAGTTTCACTGGTTGTACATCGTGACTGTCATGAATTTGATCGATTGCATGTCATACTGCACACTCATCTTCTTATCGTCCCAGTTTAAATTAACACAAACTTATTACAATCTTTTGAGAAAGAAGTATACAAAGAATTCTAATAAGAAGACTTGTAATGTTCTAGGAGAAGAATATAAAAAGGATTTTCTAATTGGCATCAGGCTGCATGAAAATGCTTTGTGGTGTGCTCATCATGTCCAGGCCTCCTTGGGATATATCTACAGTAGTCAAATATGCCAGAGCATAATTCTCATTGTCATGTGTCTTGTTAAATTTGTTACGTCAGCTCGTAATATGACGGTATTGCTGGCTAATATGACGTACTTATCGGCTATGACAGTGATGACTGGAGCATACATGACGGCTGGAGGAGATATTACTTATGAGGCATCGCTAGTATCGACGACAATGTTCCACAGCGGTTGGGATCTGGTTGTGTTTGACAAGGAGCTGCGAACGCTGGCGGTGGTGGCCATCCAACGAAGCCAAGCACCCGTATACATGACCGCTTTTGGAGTCATCATTTTATCCTACAACAATTTAATTATGGTGTTGAGATCATCATATTCGTTCTTTGCCGTTATGTATTAATTAGAAGATACATGAATATTATGGCATAATCGTAATAATACACATGTGGTTATGATATGAGCACACTTTAATGAAATAATTCGCTTTAGATTGTTCTTACATGTTATGTGGAAATGATTAAATGTATAAGAAATACGAGAAAAATAATGTTTTGTAAAAAAATGACCATTTTTAGCCTACTTCAAAAAGGGAGTAGGCTCTCAATTCACCTGTATATTTTTATGTATGTATGTGCGTCTTTTTTATATATACTCAGGACTCCAACAAATATACATCGATTTGTCGATTTTTGTTTGAAACAGTGTCTTTCCG

>SinsOR44

GTATTGTTAGTGTAACGCAAGGTCCTATGTATGTCAGTTCGAGTAGACTTTTTCCCATTATAATACCCTCTACAAACCACGTTATTTCACCACTGAGATCTGAATTCACCCAAAGAAAATTAAAACAATAAAACCAGCGATTTAATAAACGGCCAGTCATACTTCTAGAACTGTCTTCGCCGTTCAAGTAAAGTCCTGTTAACTTTAGTAACATATTAATTTTATGCAATGTTCTTTCAAATAATATTTGTTCTTGGGACATTTTTAGGAGATATCTAATACGCCTCAACCTTTTGTGTAGTAAAAATAATTTTGACTATATGATTCATATTTCACTGGGTTTTATAATAACTAGCAATCAAAAACTCACTACCTACTTAAACATCCAAAATAAGTAATTAACTCTACTCGGTTCTTAAGACTAATAAAGTAATTATAATAAAAGGTGTTCAGATTTCACATTAATAGTATTTGTCGTATGATAGTAAAAGGAGTTTTACTTTTTACTAGCTGAATCCCGCGACAAAGTACACGCGCGTTCAACGCGGACTCAAGTTACAGTGGTTTACGGTTTATCATTTTTATGAACACAGAGGGTTTTTATGAACACGGAGAATATATTTTATAATATTTCCTGTAAAAGAAAAAAGGTTTAGGCGCATTTTTCCGGTATATTTGGATTCTAAATAATTTTGACCACTTATTGACGCAGTGACTTCTTATTTTCTTTTTAGGTTGGAACAA

>SinsOR45

GATACAGCTTGACATGTCCTTAGTGGCGCATATATGGTACGAGATCTTCTTAAACTGGTTGGGCTCCGACTTCCATGCGGGCGAAGCCGCGTGGTAAAGCTAGTATTATGTATAATCAATTTCATGTGTCAGTGATATTTTAAATTGGTTATTATCACAATACATTGATAGTATAATCTATAAAGATTATTCTTTTATATATTATTCTCTCAATATAGGTGTTAAAAAAGATAAGTTTTAAGCACGATATATTGTCTTCAATAGAGCGAAATAAGACCACGCTGTGCTTAAGATCCTAGTAAAAGCTCTCAAATTAACTTCAGCAAAGCCGTATGCTGTCAATTTACAAGGTTTCTGAGATCGAGTTAGGACTAAGAGCATATTTTTCGTCATCACTGCATCTACTCTCCACCATTGGCAGTTATACACGGCACTACTTACAGCCATACTCGATCGCATTATCATATCACCGTAATAACAAAAGAAATAAATTTGTAATAAAGACATGAATAGGAAAATGATGAATGGTACCGCTAAAGGTATATTTGTTATAGCCATAATATTAAAACCAGTCAGGCATATGAGAAATGAACTAGTTACAAAATTGAAAAGAGTAGATTTAGAATATGTTGCTTCAATTAAGTTCACGCATCGTATTAGCTCTCTATGTCGATTCACCAAATGAACAAATTCTATCCAAAATGGATCGCTTTTTCCGGTTTTAGATTCGTTCGTGTCTGGTTTCACAATTCTTTCTATATCATACTGAAGAGATCTAAACTGAATATGAATGTAAGCACAACAAGCATAATAGAGACAATCTGGGCCGACAACGGTAAATATAGAGACAGCGGCTGACCAAACTTGATGAATGAATACAAATGGCCAAATCCTTATATCACGAGCATTGAACGGATATACAATAAGAAAGGGTAGTAACAAATCAACTTCACCCCTATTAACATATTTTGATATTGTTACAGCTAAAGGGCCAAATGTAAATGTTACTATTGCCATACTGTTTATAAACGTTACTGCATTAATAACTTTGTGTAAAAATGGTATTATCTTTTTACACAATTCATATTCCACTTGGTCTCTACGTCTATAATTTTGCGATGATTGTAACGTGCGAAGAGAATTAACTAAATCATTAACGTAGTGTGCGTATTTTATAAAG

>SinsOR46

TTTTTTTAGATGTTCAATAATTGTATTCTAGTATTTAATCTCAATACCTACATAGATAATTAGTGCGGTATTCATTAATACGATATTTGCAAGTTAGGTACAATACATTTTATGCTATTTCATTACAGCTTTAGCTAGTCAAAAGCATATCTACATCAACATTAATGCTAATAAATTCTAGAAATAATAATCAAATTATGTAGATTGTGTTTGATTCAGCACTGCAAAGTAGGAATATGACATACGCAGAATCGACACGAAAGTCTGTCTAGATAGACTGATGTAGTGTCCGGCGCGCAATACCATTGGTCGACTCGTGCGTATCATTACGAAACAAAGCGCTCGTTTAAATTTCACGTCCTGCTCATACCACATGCTTTGGAACAACACGGCATGTAGATCTTCACTAGAAGCAGTGAGTTCATGTCCACACCAGCAGCACACGAACAGCTGACTTATCATGACCGATAAGTATGTTACTATTGACAGAAACTGGATGCTATACCAGTCCACAATCAAAATAAGTAGGGCTGACATGCAGATGAGTCCAACGCTACCGCTGAGTTGAAACAACAGGTATGAATGGTACGCATTCTCCACTAGGTCAGTGAATGTCACTATAGCCTGGTGCTGCACCACGCATTCGATTAGTTTTTTATAGTTTTCTGATAGTATTTTCTTTCTTTCGTCATTACGCGCGTGTGATACAGGTTTGATACTCAAAATTTTATCCTTAACGATGTCGATTTGGGCACACCCGAATATGACCATGGACAGGGCTACACTGTCGATTCCGAAGTACATATAGGCGCTCATACAGGCGGTGAGGAACTGGTACGCGTAGCCTATCTCATATTGCGGTGAGAACTTCGGACTCATTGGCATCCACATGTCAAATGGAAATGCTCGAGTACCAGCATTGTCAAACAACGGTTTCAAAGCCCACAATCCGCATGTCACTTGGGAACTAATCATGAAACCTAGCAATAGCCTCTTAATCCTTTTTGCCTGTAATTTTAAAATTTTCTCGTGTTTAAGAGATAACGGCTTGAAAACGTCAGAATTCATTCGTTTTAATAATTCTTTAAGCATATCCATATTTATTTGAAAGATTGTCACTTTAAAACATAGGCAGGCTTCAGTGAAATACAGGTATAATGCCTGAGAGACAGCCTCGAGGTCACCCCAGATTTGCACTATGTAAATAGTTTGAAATAAAAGAAATAAATATTGCAAACTCATCATGAAAATTCTGTACATTTTGCTTATCATTAATTCGTTTTCAGATGGTATCCATACTCCTAAAATTTTCATTACGAATTTAGCTCTGTTTAGATAGAGATTCTCAACATCAATCATTTTTAACAATAATTTTAATGAAACAAGTAAATAGTCGACAAGACTAGATCTTTTATACGTTTAGTTTTCTCGAATTATTAGCAACTGTTTACTTTTATTATCATTTTATAATGCGATATGGGGAAACATAATTTGAG

>SinsOR47

CGTTGTGAAAAATAATATTTTTTTATTCTACGCAAATTTTGTCTTGACTAAATTCTTTGTGCAGCGACATAGTAGGAAATATAAATAAATTTCTGTAATATTCTATAATTTTTTTTGTGTTAACAGGATTTCACTGTGCTTAAGTGCACTAATTTAAAATATTTACTGAGCGAGAGCTCAATAATTGTATCTCATGTTCTACAGATATACAATATCTAAAGTAATATATTGCGGTACAGTTTCTTTACTGAAATGAATACAATTCAATATTAAAACGAAATTTCCGACATATATTTTGGACATCTATAAATCGATAAAATATCTATACAAGTAAACTTAATTAGAACGTGAGGACGTATATACTGTGTTCAATAAAGCGAAGTATGACCAGGATGTGCTCAGTATCTTTGTAAAGGCTTGAAGATCAACGTCCGCATAACCAAAAGCCGTTAATTTACACGGTATTTTTGCTCTAGTTTGAATAATAAGTAATTTTCTTGCGAACGTAGGATTAGTTGAATACCATCGACAAATATATGCAGCGTCAGCTATTTCCGAGCTTGATCTGACTATCATATCACCAAAGAAACACAGAAGATATATTTGCAGCAAACATGTTAGAAGAAAACTTGTAAACGTCATAACTACTGCGGTTTCGTCATTTATCGTAGCATTAAATCCACATAGGCAAATCAGAAACGTACTCAAAATGAAATTCACCAAAATTGTTTTCGAATTGACAATGTCTACTAAGTTGACTAAACGTATCAATTCTTGATGCCATTTAATTAAATCCCTTAATTTAAAGCGGAGTTTACTTTTAGAAATTTCCGAAGTCGGAATTATTTTTTCGATATCATGTTTTAATAGTCGAAACTGAATTTTAATAAACGCACAGCAAATAAAGAAAAAGGAATCAACAGCTCCAATGTTTAACATTACTAAAGTACCAGACCAAATTTGGTGAACATAAATAAACGGCCAGTACTTGATATCATATGTGTCGAAAGGGAAAACAACTAAAAATGGCAGAACAAAATGTATTTCATTCGTATTATAATATGTTAGCCCTATTTGTATCAAAGGTCTCAAAACAAAACCGACAAGAGGAACAATATGCAAGAACATTTCAGCTGTCAATACAATGTTTAGAAAATTAGTTTCCTCTTCAATTATTCTTTGTTTTTGTTTGTTTTCTAATTTATTTGCATTGCTGCCGTTAGGATCTGTTCCTTTATTTTCTAATTTCCTTAAATCTTCGATAATGTTGCTGATATAATGTTCGTTGAGTTCGTGAAATATAGCTTTGGTATTGGCTAACAAGCACGAAACAATGCACGAAGCGATGTAAGTAAGTTCAGTGAAACTTTTTCCATTCTTTACCCCATCTATCAACCAAAGCACTTCGCCGATAAAATCTGTATTCAGCCAAAGAAAATTAATAATATACAAGCATTTGCGCTTAAAAATCTCTTTTCTCGTTATGTTGTTATTATCTAGATTTAATGCATTATATCTAAACAAAAATTTTTGTTTCTTTAAAAAAAAATCAATTTGTAAATTTTCAATCATAATTATTTCTTTCAGTTGAAGCACAAACTCGTGAACTAGGTAACTATTTG

>SinsOR48

GCCTTGTTTCCGTGACCTTATTTTATTTCAGCATAAATATATTTGAAAGAATGTGTTTACCTACTTTGGTGATATTGTGCAATTTATGACTATTATTTTTTATAGGCACATTTATTACGTATTTGTGTAAAGAAATCATATTTTTTTTAACTAATTGTGCATTGCTAATAAAATTATTTCTTCATATTTCTCATTACTGCAAACGCTTTGTAAGCCCAATTGATAAGATCAGTGTAGGTGTCGAGGGATAGCGTAAATATCTTGCCAGCAGTGACTCTGAGCGGACGTTTGCAACGCTCCAAGAATATAATGATGAACATGGCTGTCTTTGGGTTGACATTTAGCCAGGGCCCTTCAAATGCCGCTTGTCGGAGTTCCATGCTCTTCCAAATCAACACATCGCCAAACCAACACAGTATGAATACTTCAATCAACATACAAGTTAAATAAATTGCCATCCAAACAATCTGCGTGGGATGACTGGATGGATTCTCTATCGACAGAAACTGAATTCCAACCAAACAAAGGACCATAACGCTCAGCATAAATTGCAACGCTGATGCCAAACTGAAAGCGCTTTCGATCATCGACACGTATCGAATAATGCTATTGTGATGTTTAATGCAATCTTTGAGCACTGTTTTCATATAATTTTCTTTAGAATTAACTTCCGTATTCATGCGATTACTATCTCTAATGCGTTTTCTTTCTGCAATCACGTCGAAATTTCGTAGATTGTACGCTAATATTTCTAATTGTCCGATTGCAGAGGCAACAAAGGCAGCCAATATGGTATCCATTACACCATCGATTATACAAGTGGCTGGCTTGTAGTAAATATGATGAATATACATAGTTTCGTAGACTGGAGATTTGTACACATCGAAAAATGGATATTCCACATTTGTTGGTAACACTTGAAAATTTTCACTTATCGGTATAATGCAGCCCACAGATACGGCTCCGACGCATATAGTAGAATAATATGTCATCACGAAGCGCGCGCTTTTAATGCTCTTCCGCAAAATTTCTTTGTGTTCCGTCTCTTTTGGTTTGAATTCTTCGCAATACAAATATTGTAATAGACTTACAATTTTATCTTTATTCAACCAGAATACGCCTATTTTGAAATACGTGTACATTTCACTCAGAAATAAATACAACCCTTCCGCGATGTCACCAATATTATCTCTCCGCCTCAGTAAATAAACGACTTCTCCGATGCCGGGCAAAAAACACACTAGCGATAACACGATCATCGTATACATCAACTGGAGCCAGAAGAGGGTGCTTTTTGGCGAAAAATTGTTGTACCACATCCCGTAGAATTTGAGCCAGTAAATGTGTACGGCATGATACAGTCTCGGATCGAGATTTTCCCAAGACTCCAAAACTCCATAAGGAAGTATAGGCAATAGTATAGACAATTTTTTCTTTACATGCTCGGTTAAAATTTTCATTTTTATGCTTATATTCTATGCGAAGTGAAGCCTTTGACGCGTAATTTAAGAATGCCTCTCGAAACTCATCATATGCTTCTTTGTA

>SinsOR49

TGTATTTAGATAAGGTTTTGCTTTGTAAGTTTATCTATATTGGTGTATTTGATGAAATAAAACGCGATTTTATCAAAAACAATATGTCATGCCATCTCATAAATCTTTGTGGTACTTGTATTGTTGCTATTGGATGTAAACTTACTGGTACCTCAAAATTTTATGAGATAGCATTATATGTAACATTATCCGTATACGAATACGTTAAATAAAAAAACGCCGAGCAAACCTAGATCACCCAGGTACTTTACATAATAATACGCACATAAATGTAAGAGTACCTACTATTATTTATAAGTAATAATTCATGTATAACAATAATTATATAACATTTTATTTGCGATCTAAAAATGTAAACAGTGTATAAGAAGACCGCAAAACCTGTATGTAAGTTTCCAGAGACATCGGTATAACGTACGCGATGCGCGGAGTGATCGCACGTGAATACCGCATCATCATCAGCAATAGCTGGCGACGGAACCCGGGCGACAATGCCAGCCAGTTGCCGAAAAATATTGACTGATTCACAAATTCGCTCTCGAATTTCAACTGCGTTCCGTAATAACAATAAATGAAGAGTTGTGCTAGCATACATCCGAGGTACATGGCCATCGATATAAACTCTGCTGACATTAAACTTAGACCAGCAATCTTATACATAGTCATACAAATCACCCAAGCCATAACAACTAATTGGATAACCAACGCTTCGCCAAATATGGATTCAACTTCATTAATAAACCACACAATTTGCTGATAATGTTTCACGCAACGCACCAATCTCTCCTGGATCTTCTGTTTAAATCTTACATTTTCTATATCTACATAGGTGAAATATTGATTATCTGCTTCATGTAACTTTGAGTCTATTTCTTTAAATCCATCCTTGACGTCGACGAGATGTTCCAAATCGTATCTTAACATTTGCAGCTGTATTTTGCCTTGTGCGTAGAATGCTACAATTGTACAATCCATGGTAACATTACCATATGCTTGAAGTGTTATTAGGAAGGACATGTAAGCGAGCGCTAGTTCGAACACTGGTGAAGGCGTGGTGTCAAAAGGAAAGTAACCGGTGAACTCGACATTTTCGCTTGTAGCTCTGTTCACCAATGGAAATATCGTCCAAAGTGCACCACAGATGAAGATGGCTATATGATAAAGTAACAGTAATCGAGACATCGTAACAGCATTCTCCTTCAATACGCGCACATCGTAAGAGTTGGTCGGTGCAAAATAAGGTCCGTTTATAGCAGTGATGAGGCGATCGATGCGACGGCTGCGCGAGTTGAATGCGATCTGCTTGCCCAGGGTGTTGAGCGTCGTCAGCAAAACGAAGAGAGTGTCCACCACCTCCTCCGTGCTGTGACGGCTCTTTATAACACGTATGACATGTTGCAAATTATACATGACCACTAAAGTCCATACAATAAAACGATATGAAAAATGTGCCCAATATTTCATTCGGGACACAGGTTTCATTACAATTTTAAAGTAAGCCACTCTTGCCAACCCATCGAAGTGGGGTCTGAAAGTTTCAAAGGTGGAAAATATT

>SinsOR50

CACACGACCCATTATTTAAATATAATTGCATATCTATTTATGTATACGTACAACCGCTGATCATCATAACTGTTACTGTTTCAATTTTAAATCTGAACCCTAAACTGTTTAAACGTCATGTCAGGTGGTGCTACTGGTCGTCCTCGACGCTATTTTGCTTTACATTTTCTTCTTTTAAGATTTCTTGGCCTAGGTTGGTGGCACCATCCCGATGAAGGAGACACCAGCAACTTCCCCGGCTGGTATCTATATTATTCTATCATTACAGAAGTCATATGGGTCGCAGGTTTTGTCGGCCTAGAGTCCATAGATCCTTTCATTGGTCAAAAAGATATAGACAGATTCATGTTCAGTCTATCGTTCGTGATAACTCATGACTTGACTTTGATAAAACTCTACATCTTCTTTTTCAAAAACGGCGACATCCAAGACATTGTACGTACCCTAGAAATAGACCTGCATCAGTTTTATCAGAATGATAAAATAAATCGCGCGACGATTAGAATAACGAAAATACTGACTGGAGCCTTTCTATTCTTCGGATGGATCACTATTGGTAACACTAACGTGTACGGAATTATCCAAGACATTCGTTGGAAAGCTGAAGTTGCCACGTTAAACGATTCTGGACTTCGACCGCCACGAACTCTTCCACAGCCGATTTATATACCCTGGAATTACCAATCGGACGTGTCCTATATATCCACATTCGTTCTGGAAACCGTAGGTTTGCTATGGACGGGTCACATCGTTATGACAATAGACACGTTTATCGGATCTGTTATACTTCACATGAGTTCTCAATTTTCAATATTACAAGAAGCAATAACGACTGCATACGATCGCACAATGTCACAACTTTATGGTAATTTACGACGTGACTTCCGCGAACACAATCGTGATCCGTTAACGACAATCGACGGCTCCCATGATATGGATGAGAACGAAAACAGAGAAAGATTTGTAAGAGCACGTTATAGTGAGAAAGAAATTGAATCGGCATTAGAAGAAACGTTAAAGAATTGCTTCCGTCAACATCAAGTTTTGATAAATTGTGTGGAAAAATTTGCCAAAACATATTCATATGGTTTCATGACTCAACTTCTGTCCAGCATGGCGGCAATTTGCGTCGTAATGGTCCAAGTTTCGCAAGACGCATCCAGCTTTAAGTCGATAAGATTGGTGACTTCGTTGGCATTTTTCATGGCAATGATCATACAATTGGCTATACAATGTTTCACGGGTAACGAACTCACACTTCAAGCAGGACTGGTGTCAGACGCGGTGATGGCTTGCAATTGGGAGCGGATGCCGGCACGCCTTCGTCGAGATCTGGTTTTGGTTATGGTCAGAGCGCAACGCCCCTTGCATCTCACAGCCGCTGGATTTGCCTACATGGATAACCGCTGTTTTCTAGCTATCATGAAAGCTGCCTATTCGTATTATGCAGTACTAAGTCAGAAACAAGTTAGGTAAATTAATAATGAAACAAAATTAGTAATGTCATTTTTTATTTTAATAAATAATTACATTTAAAAAAAATTAACAATATTAATAAATTGCACATTACGTTCTAATTAATTTAAAGTCGTTGTTACACATTTCTACCACTAAGTATACTATCACAGTTCTTTGGTAGGTATAAAATACAAAATTCGCAAATAAATAATACAAGTTTACATTGTATTTAATTACTTCTAGAGTCTAGACCCTTACTTACCATATTGCTTTTATTTGATTTTGTATTTTTTATAATTTTCCTATTAATTTTTTTAAACTCTGTTACGTCATACTAATTTTCATTAGTCTTTATATCATTAAAATATAATTTGGAAGCTAAAGCCATTAAAATACCGCGATCGTTTTAAAAGAAACCTAAAACCATATAAGTAGATATTTGCTCATGTGCTAAAATGAAGGGGCGGACCAAATTTTGCAGGGGAGAGGGGAAATTACCTTTATCTGCAAAAAAAATTATCTTCAAATCTTACTAGATATTTATCAACTTTTGATTAATTTTACAACTGGTTTATATTCTAACTCTGAAACGTCATACAATTGTTTTTTTTTTATTTGAAAGCAGATTTAGATTATATAACTTTAAACCTATTTTTACCTGTGACCATGGCGCATGCAA

>SinsOR51

TGAAAGCGGAGTTCAATAAACTTCAAATGTCAATTGAACCAACAAAATAGCTGGAAATGTCAAATCTAGTGTTTCTTTTTATTTTGTGTAAATGTTGAGTAATTTTTTTTATGTAAATTAGTGTTTTTTTTGTGATATACCTAGTGTGTTTGCTATGGAAAATCAAAACTGTCAGGAACGAACTGTGCCGGTGTATGTGCGGAGTTCAAGAGCATTTCGTCAATTTAAAAACCCACCGCAGCCACATATGTGTATTCAGGATACTATAAAAGATACGGCGGAGAAATTGTATGTAAACGTGTTGGGATGGCAGAAAATCGCGAATCCGAAGCAGTATTCGGACCCCATTCCTCTGTACGGCGGCATGCAGGTGCCTCATGGTTGTGGTCCGAATAGCAACAAGCCGCCTCTTTTAGTATTTGCTGTTATGGTCAATCCAGACATTTTGAAAGCCAGTGGAAAAAATGCTACAAACCCATCGGATAGAGAAGCATTAGTAAGTCTTCTGTGTGACTTCGTAGAAGCGATGAATCCAGGACTTGTCCTGGCTCGTAAGCCAGTCATTCTGAAGGATAGGGACTTGACGGGCGAGCTGAAGGATGTATGGCACGCTGTTCAGAATAAACGGGAACGGGAGAAGGGAATGAACCAAGATGTCATGTACAAGGTTTATGACATTGACGGCATAGGAAATGACGATCTCAATGAAGATGATAAACAAAACAGCCCCAAGAGTTGCAAATCGAATGATGGTAATAGTTCACTATACAGAGGATCCAATGGTGACAAGAGGAATGTGGTGAAGTCTTCAAAACAGATCCTGTTGAATGCTGGACAGAAATCAGAATTCGATTCGGGGATGAACAATTGTCAAATCAGTCAAAATCATTTAAAGGAAAACAGATGCGGAACTATCAATGCGGACACAACATATTGCACACCTGTTTATGGTCAAATAGTCTCAACGAGAGAGAATCACAATCAAATCAATGACATACAACAAAAGTTTGCTTCAAACGAAATGAAACCTTCGACTTCGTTTAGGAAAGACTGGAACACGGTGCATGGGAAAACCACAGAAGGCTGGGACGAGTTCTCAAAAAGAAATATATCCACAATAAAAAACGGCGATGGTGATCTTAAAAGACAGAGATGTTTCAAGACTGAAAAGACGAAAGATGTAACTAATGGTAAGTCTCATTACGACTTTTTCCCAGTTTTCGACAATAAAGCCACTGTTAATGATAGTACGGTTACCATCAATGATGGTATTGATGAACAAACAAACAAAGAACACGCTAGAAACGATGACAATTCTAAAATAATTATAGAATCAATGCAGAAATTAGTTCTGCGCACGACTGATAATAAATTATGTGATAATAAAACGAGTGCTATCAGCTCATAATTAAGCTTTTTACGAACGATATCCTTATTTTTTTTTACAAATGGCAACTGACTCGCTTGCGGTTTCTAAATGGTTTGTTGACAAAAATGACAAATGTGTTTTTCGTATATGACGTCATATAACATTAATATAATAGTTGTTCATACATTTTTACTGTTTTGTTGTCGTAACATGTATTGTGAACATTTTTTATGAATATATTATTTCAACATGTTGCAAACAGTTAATTTTTTTAATAGAACCTGTATTTAAAAAATTACCGAATAAACTTACATCATCATCATGCTATCCATATATTGCGTTGATTTGCAAATACTTTAAAAAAATACATAGTAGGGAGTTTATATGCCTAACGAAAGTCACTTTTGGGAGAATACTCATTTCTTTGGACAAGACATTTTAGGCATCACAATAAATTGTTACTGGAAGTGACATTTCTGCAGGTGAACAATTTTAAATAGGTCATTGACTTAGTACTGCAGGAATAACTTTGAATACATTTTTAGCGTTTACAATATTATGACGTCATATAAGAATGATACTCTATGTTTAGTATCAAAATAGAAACATATTTTTTTGTAGTACTTACTAGTCAAGTTAATTTAAAAAAAAATCGATTTCCTAAACTTATATTCAATAATTATAGCGTTTATTTACATTGCTTGAACTAGTTCAGACATGTCCCAAAATTGTATTTTTTGTTTTGTTTTTATAATAAGTACAAATAGCAATCGCACAAAAATTTTCATTTGATCTCCAATTGAAAATATGAAAAATTATGCGTGTTTTGACAGTAGTTACAACTAAATGCAAATAAATTGAGGACTATTTACAAATCAATTTTAAACAGTTATTGTCCTAATAAAAACGTCTGGAAATCTGGTCAGACGTATAAGTGTAATGAAATTGATGTAAGTACAAGAGAGCCTAAAAACCTTGAACACACATTATAAATGATAAGTTTATTTGCAGTTAATTTAACTACCGTCTGTGTGAATGCTTACGTTATATATACTATATATTTTTTAAAGGTTGTATACTCTATAATATGTGTGTTAAAAATGTCTAAGAAAATGCCGTGTTCACACTTGCACACGCCCATGGATGGTGATTACGAACCGCTATGTGAACGTGTCCTAACGAGCCCACAGGCGTTATAAAGAAGGAACGTCTTGACTGTGAGTGGGTGCACGTAGCGTCCACACGAACTGTTCGCGAACGCGTGCATAGAATGTTGTTATGATGCATGTAGACTATTTGTGCCCGTGCGCAGGTTTCGACACGGCTAAAGGTAATGCCATCATTGAACATAATGTGCATTTATAAATTGACGTTATTATAAGATTTTTTGTATATTACTACATTTTTCATTGTTGTCAATTTACTATTATATAAAAATATATTACTATTACTATATTTTTTAAAATTGACAATGAAAATTTTAATAATATTGTAGCATGATATATGTATTGTATGTCGTGCTAAAATTTGTCATTTGCATGCGTTTCTTGATATAAAATTATTATGTAATGTTGCCAAATATGAATAATATATAAATTTCGATTTTTAAAATTTAGGGAAATTAGGCATCCCTTCTGTAAATTAACAAACTGGCGATTCGGCAGTTTGATAAATTACACAAGAGATGCATAGAGTGAACTGGAAATTTATAATATATCACTCATTTGCTGCAAAAGCTACATAACTGAAAAACTAATTTTCTAAGAGGATTAATGCAAATTTTTGCTGTGAAATATGCATTTATTAGAATATATAAAACAGAAATTAAGATAAATTCATATAAAAAAAGAAATAATCATCTTCTAATGAATGCTAGTTATTACACGCCTAAAGTTTGAATTGATGCTCTTGGAAAATCACTGTTTTTAATGTCGCTGTTTCAACAAATGAGCGATATATGTTGCCTTTCTCAACGAAAATCGTCTCTTAATATATACATATATAATTTTACCTACCGCTAAAACCTTTTGCCCATTTTTTAATTGTTTTCGGCACCTCTTGGTGTTAATCCGTGCGTCCCTAATTTTTACGATTCAAATATCG

>SinsOR52

TTTATTAAATTGGATCAATTACATTTAGGAAACTATAGAATCGTGGAGTTGAAATTAAACACTAAAATTTTGTGATTTTTATTTATGCCGATAGGAATCAATTGATTTCATATTATGAAATAGGTTACCATTTCTGTCAAGAATTACATAAAAGTTTTGAGACCCCAGTTAAAACAGCTCACTATTCTCGTCCTATCAATAACCTAAACATGTTTAAAAAAATCATAAGGTATTGTAATAAAGAAAATTTTGATTTTTCCACGGGAAATATCGATGCATTTGACTTTCACGAAGTGTTTTGTATGTTTATGAAAAGTTTTGGATTTATAAAAAGCGAACTGAAAGGAATATGGATTACTATATTTATTTTAACAATAACAACTTCGGATACACTTCATTTCATTCTGATGAACATCACTATGTATTACGCTATACGTATTTTGAATATAGCATTGATAACTGAAGCCAGTTTATTCGCCCTTCTAATGGCGTATTTGCTTATTATATTAGCTTCGCTTAAATTGAATTTCTGCCAATACAATCGACTATTAGAAAGTCTAACGGATGATTTTCACTACATTTGTAACGAGGGTGCTAAATATAGAGAGAGATATTTTAAAAATGAACTTGTAACTCGGAAAATGTGTTTGGGTTGTGTTATATTCACGCTGGCCATGGGTTCAAGCATGTGCATATTTCAAATTATTAGCAGCGTCGTCTATTGTGCTACTCACAAACCGGGAGAGAGAGTCCAGAAACCTATACTGTATCCCTATTGGCTGTTTGGATACGATTATAATGCTGAACCTATATATAGCATATTACTATTTTTGAACGTTACACTTATTTTGTTTTTTTCTTACTCATATACTTTTATGCTGCAAACCCAAATACTTTGGATTAGACATTTAGCAGCTAAAATAGATATAGTAGTGTGGAATCTCGAGGACTTGCTCGAAGATACTTATCAACCAACTAATGAATTTGAGATGAAACAGTTCTTAGACCTAATTAAGAAGAGGATGAAACAAATAATAATATTTCATCAGTCCATGTATAGCTTATTAGATGACTTCGCAGCGGTATATAAGAAAATGTTAATGTTCGAACAAACAGTGACTGCGCCGCTTACTTGTATGACTGCTTATTCCTTTGTTCTGAAGTGGAATGATGGCGAATTTAACGCACCGCTTATTATACTATGTTTTTCAGTAATTGTACAAGTGTTTATTCCTAACTATCTTTGTACTTTTCTTTCTATGAAGGTTCAATCAGTATGTGACACCTGTTTCTCGATTCCGTTTTGGATGATCGATCCCACAATACGCTCTTACCTGGTGTTGATGATGCAGCGTTCTTTGCGACCTTTGCCGCTTCGAGCGGTAGGTTTCGAAGAAATGTCACTTAAGACATTTTCAAGCAAAATGGCATCTGCTTACTCGATGTTTAATATGTTGAGACAGATTAATCTTTAAAAATGTTTAAACATTCAATTATAAAGTAGATTCTATAGTATATAGAAATACATAATTCGATATTGTAGAACAAATCACATCTTTTAAAGAAACCGCAACATTTATAAAAGAGTTGATATTTTCCCTGCACCATCGCACGATTGATATTAAAATTTAAAAATCCATTTTACAGTACATACATTTTATTTAAAAATTCTTAAATATTTCTTTTGTTTCTTTCCCTACAAACTCTTAACAACATTATTAACCTTGGGTGTTAATGTCTATCTTTATGTTTAAAAAATGTATAAAAACAGAGTTTGTACATTAAGATACCATTATCTCGTCACAGGAATTGGAGTTTCCTCAATGTTCTTCTCAA

>SinsOR53

ATTAGAAAACAATAAAACATTTATTTATATCATTATTTGACATAATCACAAATAGATGCGCATGCCGGGTGGAGCTATTGTCGTGCACATAGCGAATACTAATGATACTCTAATTCTATTTTCCTATATTCTATTTAGTTTTTCGCGGTGCGTCTGGGACAGCCACTGCCGGAGTCAGGTGTCGGGTCGATGATAGTCGTTCTTTTTGGTCGTGGCTGATCTAGGCGTTGCCTAGCTTTTATTGGGGTTGGTCTCACGTCTCCCCCAGGCCTAGAGGCGTCGAATCGATTTCGTGTCAGCCGGGTTAGGGGAGAGGCCATCCTCAGCAACAACAACAGTATGTGTGAGGCTTGCCTTAGTTAATTTGTTTGATAAATTATAAACAGAAAATTAGACAAAAAACACTGGTGGTGTCCCGGCGAGATAATATTCTTCAGCGCTCTTTATTTCTTATAAACTAGCATTTGCTAAACTATGCTTGGGTTAGACTCCTTGAACCGGATTGACCCAAAAGCTGGTACTGCCGGGATTTGCTATTATATGGCAGTTTCTTTGCGAATTTGCTCTAATTTTTAGTTTTACTTAACAAAGTGTAAAAACTGTAACTTGTTCGTAAAATCCCCACAAAAGTTGATAGCGACATTTCATTCAAAGGTCCGGCTTTGAAAACTATTTTTTTTGTTGTTATTAGGCCCAGTAAAGCAATTTCTTTCTGCAGGCTTTTATCTAACTGCATCCACTCGCTTTCGAATATCCCGTAACCTACTAGATTGTTCACTTCAGTGACCTCATTGGCGTACCAATAGAATATTAATAATTGGATAATGCAAGCCAATACGAATAAACAACTCGAGACGAGCGTGACTATGTCGTCTTGTAACTGAGCGATTTCAATGATGCAAACACCAAGATTGACAGAGATAATTATTAAATATATAAACATCACAGGCGATATCAACGAATTGAACATCTTTTGGTAGCTAACCAAAGTAGTGTAGAAATGATGACATTTTACAATATTTTGATGGCTCTTCTCAATACTGTTCACATCGATAACTTGAACGAAGTACCAACGAGACACTTTCAGCTGTCCGCTGAAGAATACCATGATGGAAACAATTAAAGTGTCCCAAGCAACCACGTAAGCACTCACTATGTGACCAAAAATGGTTTGCAAAACCATGGATACGTAGTAACCCGGTGGTTTTTCTTGATTAAAAGGCGCGATACCATCGAATATCTTCGTATAGACAGCAGTACTATTCTCCATAACATGATTCTTCTGGTAAGGTTCTGAGAAAATAGCGAAATTCGAGAAAAAAGCTAAGGCCCAGAAGAAATAGGTGACGCGTCGACTTTTCTTTATGTATCCGTTCATTATCGGGATAAGGTCTGTTCGTCTGTCAGCTAACTGAGCACATTCTACTGATGACATGTAAATGATGAGTCTTTCCCACTTTTTGTAATGTCTTTGAAAGTAGCATGACTTTACTATGCCCATATGAAATGGAGCATATTGCAGAATAAGGGCGAGAGAAATAACATCAATGTTGAAAAGACATTTAATGTATTGACTGCAGAAGAACGTTACAGTGACATAAAAAAGGATTCGCTTGAACTTTGTGACGGGTGAGTTTTTATTTGGGTGCCAGAGACCTGTAAGGCCTAAAAGTTTGAGATGGGGACCTATGCATGGATGATTTGGAATTTCAAGGAAATTTAAAAGATTTCCAGTAAAAATACGAATATTTTCTTTTTTATCCATGCTGATTTAGTTTATGTAAGTTAGATCGTTGACAAGTTGAACAGATCAGTACTTAAATGAAAACCCATTGTTACGTTTTCGTATAAAATAG

>SinsOR54

ACAAAAGTAAAAACAAAAACCATAATAATATCTCAATACTAATATACTTTATTATTTTAGTGACGTGGTAGACGACGTAATTTTCCTAGATAACTATTTATGTAGACATATTATTTAAATAATATTAAAGGTAGTGCTACTTTTTAGTTTTTTTAAATAAGAACCTTTATTAAAAGCATTATAATGTGCATTAAAAAATATTAAAAAATATTAAAAATTAATCTGTCGCAACATGTTGAACATGGAATAAGAGGATGCCAAATTGCTTGAATAGGTCTTAAGTGACACTTCTTCGAAACCAGCCGCTTTTAAAGGTAGAGGGCGTAGCGAACGCTGCATCATCAACACCAGATAAGGCCGTATTATGGGACCGGTCTCCCAAAACGGAACCTCAAAACAGGCGTCGCATACCGATCGGATCTTTGTAACAAGAAATGTGACAAGGTACGATGGAACGTAAGCCTCAAAAATGACAGTAACACACACTTGTAATAATACTGCGTCGAATTCTCCGTCATTTAGTTTCTGGACAAAACAGTAAGCAGCCATACAAGTAATAGGACTGAAGATTTTCTGCTCCAACAATAACATTTTCCTAAAAACTTTGGAGTAATGTATAAATAAGCTGTACATTGACTGGTGAATTATTAATATTTTCTTCATTCTATCTTGAATTACATGTGTAAACTGCTTTCTCTCCAAATCGTTGGTCGGTTGTTTCAAATCATCCAACAGGTCTTCAAGGTTCCACAGCACTAAATCTATTTTTGCTGCTAATTGTCTCACCCATAATATGTGGGTTTGTAGCATGAAGACATAAACGAATCCGAAAAGCAAAGTCGCTATACCGCTAAAAGTCTGTATTAAACTGTAAGCCGGTTCAACGTCTAAATCATAATCGAATATCCAATACGGATGCAAGTGGTATTTTGATCTTCTTACACCCGGCTTATAGGTGATCAGATACCATAAGCACTTAAGTATTGCAAACGTGCCCATCCATACAACAGCAACCGCTGTGACAAATATACATGCCAAACATATTTGAAACGATTCAACTTGAATTTTGAAGTATCTTTCCCTATATCTAGAACCCTCGTTGCATATATATCGAAAATCTTCTTTAAGGTGATCTAATAGATGTAAATAATGTACTAAATTGAATTTAGTAGATGCCAATATGATGAAATAATAAATCATAAGCATTATATAAGTTCCAGCTTCGGTTACCAGGGCTAAATTCAACGTCTGTACTCCGTGCACGGTAGCTATTATAACCAAAATCATGTGAGGTGATCCAAAGATCAGTAATATTATAAACAAAATGGTCACATATTTTTTTAACGATTTATCCTTTATTATTCCGAAAGCTTTCATGTAAAAATAAAAAGTGTTGTGAAACTCAAACGGATTGACTTGTCCATTTAAATAGTCAAAACCTTTTTTATTGCAAAAGTTTTTGAATTTTTGTAACATTTTTCCCTTTTCTATCTTAACTATAGAAGTAAAATTGAAACATTTTATATCAGGATCATAATTTATAGTTGATTGTATTAGTATTACTTGATCTATTTCGTGTTCTAATACCAACTGTTATTTTATTGCTATATAGAATCGCTTTGTGTGATAGTAACTATACATAATTACCTACTCACCATAAGTACTTTTCTTCAAACTGTTCTTTTTATTATACTAGCCTAAGCCCATGGTTTCGCCCGTGTGTATATCATTTACATATAATTTACAATATACATCCATACATACATACATAGGCTGAT

>SinsOR55

GAATATCCATTGTACACCATCTGTGAAAAAATGATCGCAAATTAGATTTTCGTATAAACAGTTACTCTCCATATATTTGCGTTACAATTATTTTATTACTTATTGTGCATGCAATATATTACAGTCATGCATGCAACCAATGTAACTATATGTAGACAATTAGAATTGTAAAGCTCCGTAAATACCTACCTACACATTGTATACATTTGGTTAGAAAAAATGAGAGCTCGTGATTCAAATTTGAAAACTTAAAAACATTTTTCCAAAATTATATAATTCCACAGACGCAGACTCGGACTTACTATGACGAACGGAACTGCTGCTTCAAACGCGCCCGACTAGAAGAAGGCGCCAGGCGCCAACATTAAAAAAGACAGTCATAATTTTACGAAAGGACAATTTAAATGTCGCTTCACCTGAATATAGACTGTTGCTCACACTGCAAAGACATGAACATAATTTACATTATTTATTATTACGAACAGTCTAAATATGATTTCACCTGACTATATGTTTGTAAAGACATGGTAGTGATGCCGGCGGCGTTTAATTCAAGCGGTTTCTGCGCTACCGTGATTACAATCAGCAAGTCTTTCTGAAGGTCCACAGGCCAAGAAGTCCAAGTCGATTCGTATGCAGCCAAAGCTATACCATGGCTTTGTATAATTAGCTCGGTACCCAAATATGAATAAATATACGCATTAATCAATGCAATAAATAAGAAAGCCACAAAACTCGATAAAAGTGTTATTTCTTTTAAGTCGACCGATGATATCATTAGCGCGTTTACACACAATTCCATTGTCACTGCCAAATTTTGTATCAAGAAATATTCTTTGAAAATGTTCGTCATTAAATTGGAATATCTTATCATACGCACATGCTTCTTCAAAATCTCTTTAAACTCGGCTCGCATAGCGCTTTCATATTCCGGCGAATACTTTATTTTTTTAATCCTGTCCTGCAACTCAACCAACTGGTCGCATATATACATTATTATACTGCACACGATCCCTTGCATCGCAATGCCCCTGTACAAACACGGCACGAGAAAATCTATTATTAATATTTGTGTTGCTAAGAACAAATTCAGTGTACTCTTCATCGGATCAAATGGCATCCAAGCTTCAATTGTCATTTCTCCATTATATAGAGAAAAACAACTAAAAATTCCACTTAGAGTAGTAGTCAATGTAGCCATAATCACGACGTATATAATCATTTGTTTAATACGTTTTTCATCGATTTTATCGTCGGAATAGCTTATTTCTGTGATTAGAATCTTCAATTTTTCTCGATTAATGATAAAGAAAATTGTTTTCAAGACATAGTTGTAAGCGACCAAATATTTGTCACAACTGGATAACACGACTATTATATCGTTTCTATGCTGATAACTGCCTATTGCTGTTATAATTATAAGTTTGAAGGACAAAATATGCAATAGCCCTTCATAGATTTTATACAACGCCTTTTTAATTTTTGACCAATTTGAATTAGGCCATAATCCCGCGTAAAATAGACATTTTAAATTAAAAGATAAAAAAATCTTTTCCCTCTCATTTGAGTCAACCAACGCACTAACAGCCATTTT

>SinsORCO

GTTTTCTATTTACAAATTGTACAGAACTCAAACAAACAACGAGTCAAGTTAAACAAAATACAAAGGAAACTCTAAAGCAAATAGTGTAACAAACGAAACAGCAAAGACAACAAATGAAATGAAATAAACAGTAGTAACTCTAACATTGACCGATTTAGGTATTGAAAAACAGCATCGATCCGTTAATCGATGTCCACATAGAGAAACTATTAATTTCATACACACTCTGACTAACACAAGCGAAGAATCATTTATAATTAAGGAGAAGAAATTACACTCTCTTCATACGATGGCACCCATATGTTCCTTCCCACGTGAAATTTACCACCCTATTGGTCAGAATGACTTTGAAAACAGTACACGTCAGTGCAAAACTAGTAATGACAGTGAAATAAAAAAAAAATCGATCACTCGCTCCACTGTGGATGTGAAAAACACACGTGCTAGACGAAATTTTGTTTAAAATTATATTCGTGCAACAAAAATTTTTTTTTAATTAATCATAACGAAAATGGGGACCCACATTTTAGGAACAAATTAAAATTTATGAAAGATTTTATTTTATAAAGAGGACTATTGAAAATCAATCAAGATGATGACCAAAGTGAAAGCCCAGGGCCTCGTGTCCGACTTGATGCCCAACATCAAGTTGATGCAGATGGCCGGGCACTTTTTATTTAATTACCATTCAGATAACAGTGGCATGTCGACTTTGCTTCGCAAGATATATGCTAGCGCCCATGCTGTCTTCATCGTGACCCAATATCTTGCGATGGTGGCCAACATGGCCATGTATTCTGACGAAGTTAACGAGCTAACAGCCAATACCATAACAGTACTATTTTTTGCTCATTCCATTATAAAGTTGATATTCTTCGCACTCAATTCCAAAAACTTTTACAGGACTTTGGCAATATGGAACCAGTCAAACAGCCATCCACTTTTCACTGAATCTGATGCAAGGTATCACCAGCTCGCTCTCACCAAGATGAGGAGACTTTTGTACTTTATCTGTGGCATGACAATACTCTCGGTCTTGAGTTGGGTGACGATAACATTCTTTGGAGAATCAGTCTATATGTTGGTCAATAAAGAGACGAATGAAACTCTCACGGAACCTGCTCCGAGGTTACCTGTGAAAGCTTGGTACCCTTTCAACGCAATGAGTGGCACGATGTACATCGTAGCATTTGTTTTTCAGGTGTATTGGTTACTCATCGCGATGGCAATAGCAAACTTGATGGACGTGATGTTCTGTTCCTGGTTGATCTTTGCTTGTGAACAGCTTCAGCACCTGAAGTCCATTATGAAACCTTTGATGGAATTAAGTGCCTCTCTTGATACATATAGACCCAACACTGCTGAGTTGTTCAAAGTTTCATCTACAGAAAAATCGGAGAAAGTGCCCGATCCGGTCGATCTTGATATCCGGGGTATATATTCTACTCAGCAAGACTTTGGCATGACATTGCGCGGTGCTGGAGGCAGACTACAGACATTTGGACAGAATTTAAACAATCCAAATGGATTAACTCAAAAGCAAGAAATGTTAGCCAGATCTGCAATCAAATATTGGGTGGAACGTCACAAGCATATCGTCAGACTGGTCGCTTCTATCGGAGATACTTATGGTACAGATCTTCTGTTTCACATGTTGGTGTCAACTATCACTCTCACTTTACTGGCTTATCAAGCAACGAAGATTAACGGATTAAATGTATACGCATTCAGTACGCTTGGCTATTTGGGTTACACGCTCGGTCAAGTGTTCCACTTTTGCATTTTCGGAAATCGACTTATTGAAGAGAGTTCTTCTGTAATGGAAGCAGCATACTCGTGTCAGTGGTACGACGGTTCTGAAGAAGCGAAGACGTTCGTCCAGATCGTGTGTCAGCAATGTCAAAAGGCGATGAGTATCTCAGGAGCCAAATTTTTCACCGTTTCTTTGGATTTGTTCGCTTCGGTACTGGGAGCGGTAGTTACATATTTCATGGTGTTAGTGCAACTCAAATAGAAACGCGTAGTGCTCATTGTCTCTACGAAATGTTGCCACTGTTATTGTTATGGCCAAGTAGCGGTAAAACTTACTCTGAAACCACATTATGGCTTTATACAGGATGTGCATCTTCTAAATGTTCGCTCTTGACTATTACCTATTTTGGAAAATTTACGGAATCGTATTGAATGAATGAAAATGAAAATTAAAGAACCGACCAACTAAAAAGTTCGAAATTTTTTAACAACTATTTTAAACTTAGAGATCACAAAACTAACCTAACCTATTAACCTACCTAAATAGGACCACCCTGGAGGTATTCCCTATCCAATAAAAAAAGAATCATTATATTAGATGAATCATTATAGAAGATGGTGGCGCGGGGACACATGTCATTCAACCAATCAGCGCGCATCTTGCGTTCCATCGCAGGTTCATTCGCAATTTGACGGATGGACGTGGATGGATTATAAACTGTCGAATATTAATGCGTTATATTGTGTGTTGTGCAGTGCTGTTCACAGTTTATCGAGAGTTTGCAATCTCGCTAGATTTATTATAAATTAACGGTGTTTGCAATTATCCGG

**IRs:**

>SinIR93a1

TCATCGTAGCATTATTAACTTGACCAATTCAATAATCCAGTATTCTAACGTGGTACCTGTGGCACGTGAACACTTCCTAAGTTTTTTTCCATATAATTCTTCTCATTAGGAATTCACGTAAACACACCGAATCAATTTTACGTAATATAATGTTGTTCAAATTTAGATAAAGAAAATGTAATGTTTCTAGCGTTAATTTTGAGAACAACAACCTTTGAGAAGCATGGCATTGGTTTTTACCCGACTACGGTAAAGTTAAGGTACGGGCTGCCCGTCGGAACGATCATTGCCACTTGTTCATCCAGGAATTCTTCGCTACTGAGCGCAAAGTCGCAGGTGTCGGTGGCGAGGTAGTCGGCGCGCATCAGGTAGCTGAGCCTGAGGCGCCAGTCCACCAGCACATGGCGCTGAGTGCGCACGCGCTGCAGTAACGACGACCCTGATCCAAAATGTCCTTCGATGCCACTAATTCCTGTAGTGAGCTCCGCTCCTTTCAACAGAGCAATGTATTTGGGTTCGTCTGAATTCTTTAATTCCGTCTCCAAATAACTTCCTTTATTAATAGACCAAGTGTATATGGCGCTGTTTTCTATTAGTTCAGAAACGGTGGTTACCGGTACCTCCTGTTTAGGGAACGTCAAGAATGCCACCAAGTTACCAGAGTACGTGGTTACGACAACCAGCACCACCAACCACCAGGTTCCAACAACTAACCGACCGCTGTCCGCTCGGGGAAGATACATCCCACCTTGTTGCAATAAAGCGCCATATATGTACCACAAACAATTGTGGATAGTGGAGAGACCACCCTGACGAGTGACACCCATCGCCTCGTAATATGGACTCAGACGATGTATTACGAACAGTGTGGGTCCCATAAGTATCACTGCAAACCCCAAACATAGCCACGTATCAGTAGTAAATGGCAGCATAAATAGTAGAGCTCTGCTCAGATCGCGAGGACGCGCTATCATGAACGAATACGGCTGAGTGGTCACAGGTATCGTGTAGTTTATACCCGGATTTGGATCCGATACTATAGTGAAAGCTGCCGCAGCTAAAGCAGCTTGCCCTTTAGCGACAGCGACTAACGTCAACATAGCACTTTGACTGTGCATCATATCTGTAGAAGTTTCGTTAGAGAAATCTTGTTTCACATTTGACGGCAGTAATAGTTTAATCGTGAAGTTTTTATTTTTTGCAAGTTGATTGACTATGTCGAATATCAAACCGCTGTGACTTATAATTGAACCAGATTCGTTGGCTTGCAGAATTGTCCATGGAGGATTATGGTAAGTGACAATTGGAAGCTCTTTCCCACGAAATCCATGCTCCACGTGTGGGAACAGAACATCTGTCTGTTTAAAGCCGTCCACCGGACGCCAGTATCCTACATCCAACAATTCGAAATGTTCAATAACCCCAGTAGTATCATTGGACGTAGGGGCAGCAGTAGCAAGTATATTATCCGCATGGTCTCTATATGTAGCACCCCAGGTGTCCGCCGCTAGAGCACGCCACGCGCTGCAGTTACCGCAACGGCTGTTAACCGCTATATGTTGCTGCATATGTTTGAGCAGCATGTTTCGTCTCTGCAGCTTGGTAGGTCGAATAGCTTCCCATTCTTCGTCAGAGACCTGTGCCGCCACATCCAACTCCTCCTGCACTGCTGCGTCTAACGCTGATATGAACGCGTTCATCATCTCATGGGCGTAACATGTCATGTCGTACTGGCATTCGGGACGATCTTCAGTAATATTGTAGAAGTACGCGATATTCTCCCCCTCGTAGAGAGCGTTGATCAAATGCGACAAATTCCCATTGTGCATGTTCGTGTTGGATATGACATAGAGCCACTGCGCTTGCGTGTGCGTCATGCCCAGGTCTCGGGCCGTCTCCGACAACGTTGACATCACGTCCGTCGTCACTATTGCTATAAAATTCTCTCCTATTCTTTTTACAGGCAATTTAGACAGCACTCTATGTATCTCCTTCCTGCGCAGATATTCATTGACTTCATGTTTCATCTTGAAAACGGTCACAGTAACCGAGGGTACGTCGTTATTGTCTGTCTGCGACGTCAGTGACCGAACTGCACGGGACACCATATCGCGATTTAAGGTATCATCATGCAATATTACAGCCGATTTCCATTTAAACGCTCTGGCAATTCTCAAGTCAAGAAGAATTTGTGGCAATTCCTCTCCAGGATCCATGTACGTCACAGTTATTGCCGAATTGGAAGGAAGACGAGGACAATCCACTTCTGTTAATGCAAATAGAAGCAGTTCTTCGTCTTGCGTTCGAGAAAAGAGGGACCAGGTATCTTCGCATGACGCTAAACTGAATACTGCCAGGAATCCTTTTTTGAGGCTGATAGTGGTCCATGAGTAATAATGGACAACCACACCACCATGCTTGAGCTCCACTCTCGCCAATTCCTTGATGTAATCTCGCAGCTCGTCCAGCACAGGCTGAAATTTTTCGCCCAGGTGTTGCCGGTCCAGAACTACAGCTAAGGATGCGTTTGCCGTGATCAACGAGGGGAAATCTTCCGCTCTCACGTAAACATGCACACAAGCGAAAATCAATGTCCATAGTTGCATTTTGCGATAACACATGCACACTACAATATCTGCCCAACAAATAAAAGTTCCCAATCATCATTTGAAAAAATTAATTATCGCACACTGATTAAGAATTTTATTATTAAAGTAACAATTAAGACTTGTTAATGAGCGGAGCCCAGGTGTTCGTGTTAACATTTGATATTTTCTTTCTTTC

>SinIR68a

AAAAAAAAAAGGTTTTTATTCTATTTCCAATTTTATTTGCATTATTTGGACATGTTAAAACTAAAACTAGCCATTTTTATGTTTCTGAGCATAGAAGTTAGTACCGATATTGCGTCTATTTTGAAACAACTCCAAGAACGCAGAGACCTGGAGTATGTGGTCACCGACTTGGTCAATGTGGTGACACGGTATGATGACGTCACGTGTATCGCCATTATTTGTGACCAAGTCTATTTGAACGTGTTTGAGGGGACATTGTTCAGGAGAACTCTTGCGGTGCCTTATGTTATGATTGTAGTAGAAGATTATGAAGATTTACTATCGCCCAATTTCGATACTTTGGAGTCTTTACGAGAGACAAGGAAAGTCGGTTGCAGTATTTATATCATATTACTTGCTAATGGCATTCAAGCTAGCAGACTGCTGAGATTTGGTGACAGATACCGCATTTTAGATACCAGAGCAAAATATATTATGCTACATGACTTCAGACTATTCCAAAGCGATCTTAATTACCTCTGGAAAAGGATTGTCAATGTAATATTTTTACGTTATCACAAAAAAATTGTGGGAGTTTTAAAGAGTAAAGCGTGGTTCGACCTATCCACCGTTCCTTTCCCGAACCCAATCAAAGGGGTCTTTGTTTCACGAAGAGTTGACATATGGAAGAATGGGAAATTTCATTATAATAGAACCTTATTTGCTGATAAAACGAGGAACTTAAATAAGGAAGTTTTGAATGTGGTATATTTGGATTACGTTCCGTCGGTTGTGGTGATAAAGGATAATGATACTAGTAAAATTGGTGGCGTGGAGATGGAGATACTGAATATGATAGCCGAAAAAATGAATTTCAAACCCAAATTATACCAACCCATGAACGTAGAACTTCACAAGTGGGGTCAAAAGCAGCCCAACGGTTCGTTTTCTGGTCTTCTAGGGGAAATGGTGAATGGTAATGCCGACGTGGCGTTAGGGAATCTACAATATACTCCGTATCATTTGGATCTGACAGATTTGAGCGTCCCTTATACGTCGCAATGTTGGACGTTTTTGACACCAGAGTCTCTGACTGATAACTCTTGGAAAACTTTGATACTGCCATTCAAATTATACATGTGGATAGCAGTTTTATTGGTGCTTCTGGTGACAGGAATGATATTCTACGGCTTGGCGAGGTATTATATAAACTTAATGGCATATAAACATGACCCATCGGACACGGGCGTCAGCGTACCTAGTTCGAAGGGTAAACACGAGGAAGTCGACCAATTTGACGAGAAACCTGTCGGCCTGTACTTGTTTGGAGAAATAATTAACAGCATCCTTTACACGTATGGCATGTTACTGGTCGTGTCCTTACCTAAATTGCCTACGGGATGGTCCATCAGACTTCTCACCGGATGGTATTGGCTGTATTGCATTCTTCTAGTTGTCTCGTACAAAGCTAGCATGACCGCCATCTTAGCGAACCCTGCACCGAGAGTAACAATTGACACTTTAACAGAATTAGTAGAAAGCAAAGTCACATGCGGCGGATGGGGAAGCGAAACTAAGAATTTTTTCGAAAACTCTCTAGACGATGCAGGACAGAAGATCGGTCAAAGATTCCAAACTGTCGACGACCCTAACGAAGCCGCCAAGAGAGTAGCACAAGGTCATTACGCTTATTACGATAATAAATATTTTCTAAAATATTTGAGCGTGAAAAGAAGTAATGTTTTCATGAATATGGAAATAGAAATGGATAATTCAACTATGAATGGCACAGAAGTCACTGTTAGAACTGAAACCGAAAGAAATTTGCACATAATGACAGATTGTGTCGTAAATATTCCCGTTTCTATAGGTTTTCATAAGAATTCGCCATTGAAACCCTTAGCAGATATTTATATTAGGAGAATAGTAGAAGTAGGTTTAGTAGAAAAATGGCTCAATGATGCCATGGACTCAATAAAATCTTTAGAAGCAGAAGATGAAGAAATTAAAGCTCTGATGAATTTAAAAAAGCTATATGGTGCTTTTGTTGCTTTAGCTATAGGCTATTTTTTGAGTGTGGTTTGCTTGATCGGTGAATTAATACATTGGTATTTGATTGTTAAAAAAG

>SinIR75q2

GAAAAAATGTCTTATATACATAAATGTCGCAAGTCAAAACAAGATTATTGTTATGTTACATTTATAAATCAGTGCCTCAGTGCCCAATTATTATCGTCTAAATTTGTTTGTTTATCATAGAAGCCTTTATAAATATCTACTTCTGTATTTGTATAAGTAATTGCTAAGCTTTCCATCGTTTTTTAATAAATAATTAATCTTCAATTTGGTAATTATATCATGTTTAATAACGTTAAATTTATACGGATTACATTATAATTATTCTCATAATATAGTTAAAGTAACAGACTAGTCTCTACATCATACACTGTTTCAAATATTGCTAGTACAAAAATAAATTTTATTATAACGAATTTGCCTAAGAACAATATGGTGGTTCTGGAATCATATAACAATTTCTTATCATTAACATTTCTCTTTTGTGTAGTAAATGTTCAATTATTAATAAAGAAATAGATACTAATGCTCCATAGAATAACACTAGAAAAGCTGGATAACAATCAACAATGCTAACTGATACAAAGTTAGATCCCGTTCCAGTGCATTTAGGTCTCTTTTCGTAAAATAAATGATTTTCTCGACTTTGTAGTCCATGTTCTTGTATCCGTTTTAACCCTATTTTAAACATTTCCTTAAAGGGTGTATTTTTCCTCACAGCCAACCAGGGATCTATTACTTGTAAATATTCTATTTCCTTTAGTCCACATTTTTCAGCTTCTTGAAAATATTTTCCTACGAATTTATATCCGACTCCTGTCTCCATATGAAAAGCGAATAAGCCCTGTTGCATTTTTTTAACTCCTTCTTGCATTGTCATAAATCGAGGTTTTGTGCCAGGAGGTGCTATTTTTGTTTGATAAATAGCTTTTCTAATTGGTTCAGTTGCAGTCGAGAAGTAATATCTGTTAAAAACAGTATCGTGCACTCCAAATTTAATTCTAGAATGAAGTAAATCTTCGAGAGTTCTTATTTGTGATGAGCTAGATTGCAGCAGAGCGACAATGTTCGCCGAATACGAAGTATACAGGAACATGAGCGTCAGGAATAGGATCAGCATTACTACACGACCTAACGAACTTTTAAGTTCAACTGGACTTCCTTGCTGACATGCTGCTCCGAAAATTAAAAGTGCTACATCTGCAAGACTTGGTCTTAGTATATCAGCATCTTTCTCTTTCAATTGATCTCTTTTACGTTCCCACATCGCGACGATAAACAATGCAAGAAACAGTAAGAAGATTAGGCCTAAAGTGCTGTACCAAACCGTAGAACGGAATGAAAGAAGGAACAAATTGTTTTCATAAGATAATTTTGGTTGCTGAAAAATGAATTTTGAACGTGTAGGAGTCGGGCTTGATATGTAATCGACTATTGCTATACGTTCACTTGTAAAAAACATTGGCGAACCTCCAATTTCGACTTCTTCTCTAACCAAGTATCCTGTCATACCATTCCAGGTACCATTGACACGATAACCCCACGTCTCCACAAAAATATATTTCCTCTTCGCATTGAGGAAATCGAGCAAGTGATTTGTCGTGGGGAAATTCACTTTGGTAATAGTATCAATGTGATCGTTTACTCCATCGGTTAAATGGTTAATGCTGTCATTGTCGGTAAGAACATAACAAATGTTAATCTCATATCCATTTAAATTGAGTCTCCGTAAAGCAGCTGAATCAATCAATTGATATGCCTTTTGAAATCCGTACTCTATGCTCCAATTTCCGTAAAATTCTATTTTCCAATTATTTGTATGACTTATTTTATAAACTAGGTGTAAGTCATAAGTACTATTATCAATAGGACGAGCAATTAGAACTTCAGCATCAAGCAGTACCTCGATGTTACTTAAAGATTGTGGTATTATAGATTTGTGGGTACCATTATTGAAACCATCAATGATGATCCATCGAAAGGGCGATCGAAAATGATTTTCTATTTTACTCCTTACGAAATATTCATAAATATCTGGACAGCCAAGGTCTACTAAGAATATTATGTGCTGGTCTTGAGAATGACGCTGTGGAATATGGCCCGGTTTGAGAATTTGCATCATGTTGATTTGGACAACATTCTCTCCAGCGAGAATTGAGTACAATTGAACTTTTTTGTGAAGAGGCCAGCAGAGCGTGGCGATTACGGCCGATGGCCTTTCCATAGCATGGATGACGTTGGCAATCATCATTACTTCTAAATCAGTTTCCGCGTGACAACAAGTTATAACGATTAAAAGAAAAACAGTTAAACAAATATTCTTCATGGTAATATTGTTTTTTTGGTCGATATCTTTGAAACC

>SinIR75p2

TCCATCTAATTATATAAAAACAGGTTAAATAAATTCTATTCGCGTATGTATATACACTTGATAATATTCAATTTCATATTCCCTGTTGCGATTTGCCGCTTATTTAATTTATGAAATATTATTTCGATAAACATAACGCCTATGGAGACAGCAGCCCCGATAAGCATGAGGAACATAACGGCTCTCAGATCCAACAAACCAACGCTGCTAAATGCTGATACTTTCTCGGTGCATCGAGGCTTAGGTACTTGAAGTCGTTTGTAGACAGCCGATAATATACCGGATTCGCGAATTTGTTTGATGACAACTCGCAATAATTCCGTATACGGTGAATGTTTCTTTATAGGCGTAAATGGATCTAAACTGTTTAGAAAATCCACTTCGGTGAGGTCACACTTCTCAGTTTCCAAGAACGTCTTTTCTATACGACGATACACCGGTTCAACTATGGAATGAAATGCAAATAGGCCCTGTCTGATTCTCTCAACGCCTTCGTTCATGTCATAGAACTGTGCTTTTCCATTTTCAGGTTCGAGTTTTTTTTGAATACTGACTCTAACTGGATCTTTGAAAAGTTTAAACACAATATGATTATAATCTACATCATTAGCGGCGATAGTGACTTTGGAACGTGCCAGTTGAGACAATGTTCTGATAGAATTCGACGGGGCTTGTAGCAAGACCACGATGTTGGCAGAATAAGCCGTGTATAGAGCCATGAGGGAGACAAACACGAACAACATTATTATGCGACCAGAAATCTTTTTAGGCTCCATAATACATCCTTGTTGACTTACTGCACTCATTGTTAGAAACAAGGAATCTTGAAACCCATCCAAGTGTGTTGGACTCTTTCCAATAGCAATTTCCCATTTGCTGGCCAAATATAAAGCAACAGTCGAGACAATTGCGCATACGACTATGGCTATCCAGACTCCACTCGAGAATGGCAGGGAAAATATATTAGCGACGTAAGATAGAGGGGGTTGGCGGAATATAAAACGCACCCTGTACGGAGATAAACCTTCAGTATAAGTAATTACATCCAAACGGTCTACGGTCATTAAGAGGTTCGTACCCACATCTGCTCTTCCTGAGAGCAAGTCATCCACCATTCCCGACCACTGACCGTTCCTCCTGTATCCCCATCTATGACTGAATATGTAGCGCGGAGTTGCATTCAACATTTGAAAAGCTAATTTAATATTTATCCAACATATTTTAGTGAAAGCATCATATTGAAGTTCCAATCTGTCTTCTTTGGACAAATGATACTGAGTAGAATTGCTGTCCTGTATGACATTAGACATGGTAAGAGCATGTCCCATGATGTCTCTTCGTCTTCGAAACAACTCTCTGTGAACTCTAATATCTACCAAAGTTCTGTCGTAATATCCTCTGGGTGTATATTGTATTGAATAATTAGGTGACGACTTATGAAGTTCCACTAATTTGTATCCATTTTGACTTTTCTCAGCAAATACCAGATCGCTATCTGACAGTAATGGACATTTTAATAATGATTCAACTTTGTATTCCTCAATTGAAGAAGAATGCGAAAGAACTAACCACCGATAAGGGAATTGGAAGAGGTTTTTAGCCGTAGCATTGAACAATATTTCCTCAGCGTTTGGGCAATCCAGATCTATAAGAAACAGCGAGTGATGATCTTGATATTTATTTAGTTTTATCGATGTAGAAAATCGAATGAAAGAATTAGACATTTCTTTTGCCAATTTTATTTGAACATGTTTACTCCAACACAAATTGTTATAAATCAAAGAAGTCGGTTTACCTTCATTTATAACGAAACGTTTTATAAAATCGGTCATAGAATCGTCCTTTGCGGAGATCACGTTTGAAATTATTACAAAAATTAAGAAAGCATATTTTAAAATAACATTCATATTTGTTTATTCTTCCAGAGATAAGACTTGTAAGTAATGAGGAGTGAAAATAATTCTATGGATAGACTAGTCACATTACATTTTATGTCCAACCGCATCGAAAGCTTTTTAATAAATAATTGATAAATTCGCCTAAGCACAGTTGATGCGCTTTAACGCTAAATACAAATCCAAATTGCTTATAATTATACACTATGGTACCGTATTAAGTAGAAAATGCCTAATGTTCTAGTGAAACACCGATTTTGGTTGCAAACGCTTTGTTTGATTGATGTTTAAACAAAAGTATTTATTTCGAAATATTGTTTATATTGTTGTTTGTTCTATTAGTTAGGTAGGTACTATTTATTCGGATCCATCGTTTTATGTAGCTGTCTCTTTTGTAATTTATATAACATAATTTCAATAATAACAATTCCCACGGATAGTCCAGCTCCAAACAACATGAGAAGGAGTAACGGCTTTAAGTCCAAGAGTCCAACGCTGCTGAAAGCTGTCGATTTTATGCAGAGAGGTTTAGAAACTTGTAGCCGTCTCAAGACAGCAGATAGGATGCCCGATTCTCGGATTTGCTTAAAGCTGGAAACATAATTTAGTGCAGTTTATAAAGTACAAGGTTTGAAGATTAATGCTGTATACACAAAGATACGCTTGGTGTGAAGTGTTTTAAAAAGTTTATTGTTTGTTTATTTTATGTTACATTTTAGTTATATTTTGAAAGGGTCTTTTTAGTGAATTGCAAATTTTTCTTTTCATTGGGCGTACATAACAACGAATTAATAAAATAGTGATAGAAGAATGCTAAAAATGGGATTCAAGAACTCAATAAAAGAGTTTATATACAAACACAACTCTAAACAATTCCAAATATGGCGAATCTTTCTTCGCTGGCATGTGAGGGTCGAAGGTATTAAGAAAATCCACTTCCATCAGATCACATTTCTCTGTCTCCAAAAATGTATTTTCAATCTGTCGATATACTGGTTCTGCCATACAATGAAATGCAAATAAACCCTGTCTGATTCTCTTAACTCCTTCTTCTAACTCATAGAATTGCGGTTTCTCGTGGTTGAGTTCGATTTTCTTATATATTTGTAGTCGTACTGGATCTTTGTAGAACTTAAAAATGAAATGATTATAGTCGACATCATATGCGGCGAGTGTGATCTTAGAATTTGATAGCTGGGCCAAAGTTCTGATCGAATTTGATGGAGCTTGCAGAAGAACCACAATGTTAGCAGAATACGAGGCATAGAGGGCCATCAATGCAGTAAACAAGACGAAAACCATAATACGACCTGCAGAAAGACGTAGTCGAAACTGTGAGCTTATCAATGATTTTTCTATTTTGAAAGTTTGAAATGTTCAATTGTTAAATACCGGGCGTTTTCTTCGGCTCCATAATACTACCCTGTTGACTTACAGCACTCATAGTTACCATTAGAGCATCACTGAATCCATGTAGCTGAGTTGCGTCCTTAACAGGCACGTCTTCAGCGTTAACTTCCCATTTATTAGCTAAATATAAAGTAATAGTGAAAATAGCCGAGCAAACAACAACCGCTATCCACAAGTTGAATGTGAAAGGCAGGGAAAATATATTGGTAATGTAAGACAGCGGTGGCTGACGGAATATGAAACGGTGTCGATAGGGGGCCACTACATCTGTGAACGTGACGACTTCGACGCGATCCCTGAAGAGAACACAGTTTGTGCCAAGATCTGCCTTATCTGTTAGTATGTCATCGATCATGCCGGACCATTGGCCATCTCTCTTGTATCCAAATCCGTAGCTGAAGATGTATTGCGGAGTAGCATTCAACATTTCGAATCCTATTTTCACGATTGTCCAACTAATCTTTGCTACTGCATCGCGATGGAGTTCCAGTCGATCTTCACTCAGTAAATGAGATATGCTTGTATTGCTATCTAGTATGACATTGGACATAGTTAGAGGATGTCCGAATAAATCTCTTCGTCTTCGAAATAATTCTCTATGAGGTCTCGTGTCGACGAAGTTTCCATTGTAATAACCTCTCGGTGTGAATTGCATGACGTCATTTATTGAAGATTTATGCAATTCAGTTAGTTGATATCCGATTTCTGCCTTAGTTGCCAATACCAAATCGTTATCTGGTAACAGTGGGCTATTCCATAGAATATCCAAATGCTGATTAGTTCTTGAGCTGAATACTAGCCACCGATAGGGTGCTCGAATAATATTTCTTGCAGTTGCATTAGAAATAATTTCTTCCGATTTAGGGCAGTCTAAATCTGTCAGAAATAACAATTGGTGATTCTGATACTTGGAACTCGGCTTCATCGATTTTGACGTTCGGATTCCTGCTTTTGACATATCTACAACTAATTTCATCTCCTGATACTTATTCCAACACAGGGCGTCGTAGATAAGGAAAGTCGGTTTTCGTTCATTGTCAATGAATTTTTTAATAAAATCAATAGAGTTTATGTATTTTGTAGCAATTATATTTTTTGATATCAAAAAAATTAACAGGATAATACATTTATAAGAGCTCATGATTTTCTACACAACTTTCTTTAACAAAATAATTTTAAATACGTTCAATTCAAATGAACGATCAACATTAAAGCTACTGATAATTCTTAAAGCTAAAATAACATTTTAAGAACATAAATAAATCATATTTACTTAATAAAGCTTAATAACAAACAAAAGCTTGGAAATGGTTAAAATCATTAAAATAACGAGCACAGTTACTTTACTGAAAAGGAAATCGTTTGTAACTAATTGAGTAACATGACATTCTAAAGTAACCGCCTTTATAAATTTTAAACATAAACAGCCCGTTGGGTGCCTTTTTGAATATAAAACTAAAGAAAAATATTATCTTTATAAACAAATCCTGTACATTGTTTGTTTATTTAGTATTTTCTCTTAAATGTTCACCTACAATCTTGTGCCTCTAACGGCACACAACTTTTTTAATGTTAATTAACAAATTGCACAATTTGGTCTCCTACTCGTCACGTAATAAAGTAGGCAGTGCCATAAACACACGCAGCGGTGACGCCACTAGTGCTATCTTCGCGACGGACGGCACAAGCCTTCAAAAGCTTTTAATCTTGTAAGGTAACAAAGATCTTGTGCCGTCGACGGCACAGGATATATGACTTCAACTAAAACTTGAAATAATATTTTTTTAGAGTA

>SinIR93a2

CATATAAATTTGATCAAAATCTAACCATGGATTTCGAAGATATCGCTACAGAACTCCTCAATGACAAACAGCATACATTGAAGTCGATTCTTTTAACAAAAGTTTTACCTGTATAAAGACAAAAAAAGATTTGTCACAGGTTTTATGTGAAGGATTTATTCATATCATGTAGACACAATATTGTATACCGGTAAAATAATATTATCTATTCCACATAAGGCTTTATAACAATATTTTCGCTTCGTTTCTTCCGCCTGTTGTACGCCCATTCGATAAATAACACCGTTGAAGCTGAGAAAAATCCTAGAAACAGTACGAAGAAAGATCCTTGCATGTCGCTCAAATTGACCGTATGGTTGTTGACCTCCTGTGCCGCTGAAGATGCCTTCCAACAACGATCTCGTTTTGGTAAATAAGCCGACAACCATTTTGTTATTAGACCTGCTTTTTGCATACGATTTATTTCTTTGTTTATGACTGACAGGTACGGGCTGCCCGTCGGAACG

>SinIR75q1

GGTCACATCACATAGTCGAGATCATAGATCTCTATATAAATAGAGATTTATGGTCCTTATATGTAGATGGTGGTCTTTATAGTTCAATGTCTCTCTTAATGTAAACAACCATACATTGAGTTTGAAAGGAAGTAAGAATAAATTGTATTGATAGGACAATGGTGGTTCTCGAAACAAAAATTTCACTGTTAGTCCTGGTGATGGGTGTATTAAGTAATCCAAGAAGTCGATACGTTGTCTTGTTATGAACATTACTGAACCTGCAAGATCAGCTTTTCCACTAACCACATCTCCAATCATACCATTCCAAGTTCCATTTATATAATAACCCCAAGCGTCTGGGAATGTTAAGACTGTTGTTGCGTTCATGAAATAATATAGAGGCATAATCTGACGATAACTTGTCTTGGACACGCCGTCGACCTCAATATCATCATATGAAAAACAAAACTACCATTGCTTCTGTTTCGTGATATAATAACTTCAGAATCTGTTAATATGTTTAAATGATTAAGTCTCAGAAGTACGTCACTACTCTCATTCGAGGTTTTTCAAGTATTAACCATCTATATGGGGCATTAAATTTTTTAGAATTGTTGGCCTATAATAGAAAAAATATATTTAGAAAAAAAAGAACATTACATAATCAAAATCACGAGGAAAGATAAGTTTACCTGGGTAAAAAAGTCTTCAATAAAAGAACAATTAGTATCGATGACGAAAAATACATTTTCCTTATGATTAAAATCTTCAGTTATCATATTATTATTGTTAATAAATTGAATCATTTTTGGAGTTTCCGTGTCACTCAATTCAGTCGCCAATTTAACTTTTTGCACTAAATTAAAAATAAGGCATTGCTTTACAATATTGTGTAGATTCTATTAAACAATCAGAGCTAAGGCTAAAAGTATTACTCACATGTCGGCCAACAAACGTTGGCTATTACACAAGTGGGTTTGTCGTAGGATTTAATTACATCAACAATCATTGCCAATTCATTGTTTTTATTTAAGCTTGAAGTAACTAATTTTGTACCAAATAAAATCGTAAACATTACAACGATTACTTTCATAATAGTTACGATACCTAATTTAGTCTACGTATGTAAGTAAAAACTCTACTTTATGTAATAAATACAGTATGAGAGTGTTGATAGTTCGCCAGAACTCTTACCTACATAAATATCATCTACATTACGAACAAAACGTGGTCATTGTTAATTCACATAATTGAATCAGTGCTATTGATATCTAATTACTAGTTATTACCGTCTAACTCTTCTTGTTAATCACAAAGGCGGTTATATCTAATTTTACGTTTGTACAAATAATCGCTAAGGTTGACATAGCTTATTAAAAATTAATAGGTACCTGTTTTCGATTCATAAATGTTGTCCGTCGATACAAGCAGTAGATATCAGTTCTTACAAAAAATATGTTTTAATAAATTATATATGTATTTTAGGTCATTGGTGGCAAATGAGATGAGCGAGCATAAATATTTATAAAATTAAGAGTTTCTATCAATTTTTCGGTCCAAAAATAGTACATTATTCAGCGCA

>SinIR41a

GTGAACGAAAAAAAAACCCGCTGAGTTTCCGGACTGTTCTTCTCAATGTTGGCTCGGGTTCCGTACAGTCTGTAATTTTCTACTGTTATTGAAAGTTTAATCTGAGATCATTTTGTTAATATAGATAATACTCATTATATATTTTATTCATGGCTATACAAACACAAAAATTTCACCGTGTAGGTATCTTAAATAATTTCGTGTAACGTAAGTAAGTAAGTATAATATCGTAATAAGTAGTAATTTTTTTCCATTACTGATTAAGTTTTTTCTTGGTTTTATCATTAATACACATCTCTGCCACAAATATGACTATAGAAAGTGAAACACCAATTGAATATATGATGAAAATTCCCACAACTTGTCGAAAACTTAAAGGCTCTATATTATCAACGTCTCTTCTGGAACGCGATAATTTAACTTCTAGTTGAACTTTGTAATTTAAATGTTTTAAAGCAACCTGTGTCTCCCAAGCCAACATGAGACCAGATTCATGCAGCCGCCCTACAAGATCGCTGATCTTGGACGTATACGGGGAGCTCTTGCGCAACATTACTACACATTGTTCGTAATAAAAATCTTCTAACATTATTTCCAAGTCTAACATTGCTTCTTTCGTAATGTATTCGCCTACCGCAAAGTAACCTGCTGGTAATTTTTCTATACTGAAAGCCATATTTCTTGTAAAGCTCTTCCGTTTTAATTCCTCAGCTGAATAAATTTTAAACTGACTAACCAATTGTTTTATTAAAGGCTCTGTTGAAAGAGTGATCGAAAAAATCCAGGCATCATGAGTGGCTCCCCATTCCATTTTTCGATCGACTATATCTTGAACGGTATCGATAGATGTTTCATACTTAGGCACAGTAAAAGTGGATGCTAGACCACCTGAGTAAGCATTGTCGAAGACCAAACCAGTAATCAACAACCAACCTGTGATGCTACGAACCCGCCAATCTGCCCTTACTTCCGGCTGACATTGCGTTACCATCATTCCAAATGTAGTTATGAATACATTCTTTGACGAAAATCCTTTTGCAACAATAAGAGCGACAGAAGAGAAAAGAAAAGTGAAAATTAATGCAATCCACATATGCCAAGTAAAGGGCATTAATGGCAATTCCCAACTTGCTAGTAACCTTGGAGCCGGTGCTACGCAGGTTATAGCAGTTCTTATACATGGAGCAGAGAAATCCAGAACAACATATTCTTCATACCAAGAATAGAGAGCGGTAATGCCAAGATCTGCACGATCTTCTAGAACATTTCCAAGTACACCGACACCAGTTTGATTGTCATAAATTTCACCCCATTCGTGAGCATCATCCCTTACAACTTCCATGGTGCAATTTACCCATCTGCAAAATTCCTCTACAATCCTCAATTCCATGCCATCACGACCAGTTGGTGTATCGAGATCCAACAAAACATAAGGTTTATAGGTAAAACAAGCCACCTTTACCGTTTTGCCATACAAATTCGACATGTCATGAGGAAATAAATTTACATTCATGACAAATTTTTCCGTACAAGAATTCCATCGATCTAAATACAGCGGTTGATTAGTTTCCTTGTCTGCTCCGACATATTTGTGTGTTATTATATCATACAATTTGCATTCCGCTGTCGATTCTCTAGAAGGAATCAACAATAAAATATTTGCTACAAAGCTAGTTTCCTTCGTTGATAACAGATTTAACAATGGTAAGATATTTTCATTTGTAGTGTTATCATCTTCAAGAAATGGCAAAAATATTAGTTTTTTATCACTGCGTCTGGCATTTCCCAAGTAATTGACTGTCTCATATGCATTCATGAACTCCTGAGGTTCCTTCATTCGCACAATGTAATCAGAACAACCTTTTTCGGATACTTCCAACAGTTGATTCACGAGATTTTCCGGATCGGGAACAATACATGTGAACGTAATGGGAAAGTTAATATCTAGTGGTATTTCTGAAACGAAAGTTACACAGTAAGAATTGTGTAAATATTGATTAAGGATAGTGTGAAGTAGTAATTCAATATGAAACACATTTGGCTGCAGTGTCAACATCGTCAATATTCAGTTCTGAAAGTGAATATTGAAACTAATCCTTTTGCAGTTTAAATACAAACTGACTTATACAGTTCATAACTTACCTATAAATGCGTTCACAATCACATCTTTAAGGTTCCGCAATGAAAAATAAACAAAATCTAGTTTATACAGATAAGTAATATTTCTTTGCCGCTATACAATGTTACTTTGACAATGAAACCTTAAAAATAAATTTCCGTGTTGTAATTATTAATTTCTAAGAAGCGTTTATTAATTTATCGAACTTATTTTCGGAGGACATACCTATTAATGTTTGATAGAATTATTGTAATCATCTCTATCATAATTTAATGGTGAAT

>SinIR75a1

GGTAGATAAATATTTATATTTCTTTTGTAATTTATGTTATGTTATTAAATAAATTTACAATATAATATTGTTAAGACGTCATTACATGCAATATATATTAAGTAGTTGTTCGTATTTATAAAATACCTACGACATTATCCTAAAAGAAATGTGCCAATCAGGAAGGGAACTAATTAGGAAAACAATCAATACTTTTGCTATAAATAGGCGTTAGCTATTGAGACAGACGGTTATCTTCAGGAAAGCACAGAGCATATTATTACATAGGTACTGAAACCGAACGACCGGTGTGGTGTTAGACTCACAGATAAGTTATTTGTCTATATTGAACATTTTATAATATTGTTATTCTATCTACTATCAATACTTGTTACAATTAATTGTTTATAATCGCCTTATTTAATGTATTGTTATTAAATGTAAGTGTGACGAAAGACTAATTTAAAAGGGCTCAATGCAAAAAAAAGTGATCAAATAAATAATTATTATGAATATGTTTAACTATAAACTATTATAATATAGCCTCTTTACTTACTGATTATTAGTACAGTCTCAACAATATAAAATTTGACAAAACCAACTTAATCTAAGGAATATTGTTGTGCATTTTTTATACATTTACGTAACATTCGAGTGGACTGGCACTCTTGCTTCCATATCAAGTCACTAGATATCTAGATAAATGTTGTAATTCTAGCACATTGACGTAGATCATTTTTTGTAGTGAGTACTTAGCATAAACATAAATAATCATAATTATTTGATACACCACCATCAGTTCACAAAAGTGATATTTCTTTACGAGACAAGACGCCCTATAAAAATAGGACGATTATTTTTAAACGTGAATATAATTTTATCTGAAACTATTAAAAAGTCCACAATTGCAAATCGAAAATAAATACCTTAGGATATTATAAATATGTACGACATATAAATATTTATGTGAATGTTTTTACGTTGATTTAATGGAAATGTGAAATATAAATATTTGATATTTGTTCAAATATCCCGTTTTTTTCCTTAAATGTACAAAATATGTTTAGACATATTTTATGCTTTAATAAATAAAATACACTCTCTACATATAAATAGTGATGTGTATAAAGTTAAAAGAAAAATTAAAATGAAATACTTTAATAGCTAAAAATACGAGCGACACTAGAGATAAACATCTCTATCCACTTAGTGCATCAAGTGGGAAGTTATTCAATGAGCCTCCGTCGGGAGTGAAGAGGAATTGATCGCATCATTTATAAGGCACGTCCTGGAGTCGCGACGTCCCGCGTAACGGAGACGTCGGGACGTCCCGCGTCCCGTCGTGTCGACGCGACGCTCCATCGCTCACCGCCCGAGCCCATCAAACATCTTCCCTTTTTATTGTTTTTTTATGTGCCCCGGTATTATTCGTTCGAAGAGCGCTCTCTTGTGCGTTTATGTGCTCGATTACGTTGAATAGGCGGTGCGGTTAACGCCGTTTGTTTCTTAGCTCGACCCTTTGCCACAAATGTTCGATAGCCAACATAAACAAAGCTAAAATTGTAGCTTCTAACAGTACGGCGAACGCAGGCGCAGCCTGTCCCAGTGCCAGCGCACGCGGAGTGAGTCCTGAACACGTAGGGAAGTAACCGCTCACACGAGCACGAGACGCGGACACGTGTCCTCGCTCCAACAGGCGCATCATACTCCAAACAAAAAACTGCCTATACGGACTCTGTTTCTGACCCATAATGTAAACATTAGCGGGCTTCATCATTTGCAAGGCTCCCAGCTCGCACAGCTCTTTGTCGTCGTAGGTTTTGCCGATCACCCGATTCGCTGCATATGGCTCAGTGTGATACGCGTAACCTCCAGTCCGCACCAGCTCGATACCGGTTTCGACTGGTTGCAGAAGTGGCACGGTACGCTTGGCATACTTCACTTTCTTGTCTCTTAACACGTCTTCTTTTCGGTTTTCAATGCCGCTGTAGTAGAAGAATCCCGGATTATCAAGCCATCCTCTATTATAGCCAATATCTTCAAAGATCAGTTCAAGGTCGCTATCTATGAGACCGTCCATGGTGGCGATGGTTGGTGTTGCAGCATTCAGCAGCCAGGAGACTACGCTACTGGTATAATAGTTGTAAAAGAGCATGCTAGTTAGACCTATTACTAGGAGCACCAGACGTCGACCTGATGGAAAGTCGTAACAATTGGTGTTAGTTCCATATGATAACGGTCTAACGATTATGTAAGTAGAGTCAACGAACGTTGTAAACTATGATCTATTATATACAATAATAGAAAAAAATTCAGTGTGCGAAATAGTTAGGTCAAACATGTACAGAAGTATATCTCTAAAATTTAATAGAATGAATTAATTCTATAATGATTATTAAATCTTTATTCAAATGGTATTAACAATAGTATCCAAATCTAATATGAACCACTCACCTTGACTAGAGTGAGCTTCGTCTGTTAACTGAATACCGTCTTCGTAATCTGTATCAGTAATGTTTTAATTTAGGAGTATTATCGTTTCATCATGGAACGGACAGTAGTGGGTAGAAATATCATTCTTAGGTAAGTCAATGTACCATTTATTCAAACAAAGCCGTTTTCCAATTCATGTAACCCACGCATTCCTAAACAGTAGATCTTCCCACATCAATTATTTCCCTATTTCTCTAACATGGAGCACTTCAAAATTAAAGTGAATAGAATATAAGGCTCATCCTTGTGCCAAAATTAGAAGAATAGGGCCACGGTATTCCCTTCCTGTCGTAAAAGGCGACTAAAAAGGGTCTGTGAAGCGACCCCGGAGTATGGCAGCGTGAGAGCGAAAGCGCTGCCATGAGAGCCCTGCAAATGTACCGGGCAGGATCAAAAGCCTTTCCATTTGTAAACCATGTCGTTAACTACCACAGAGTAGAGTTGAGGTTAAATGTTAGCCATTTGCCGTAAAATACAAATCATATCTAACTTTGTTGACAAGCTGCAGCGAAAACACAGAAGAAGGCATACATTCCCGGTTCACTACGCTTTTCCAGAACAGCACCAGCGGCCAGCAACAGCCCGCACGCAAATGCTGCTGCAACTGCACACCACCACACGCCCGCTGACATTGGCGCAAGGAACCTGTCTTCGTACTTCCCAGCGCCTTTGTCCGGAATTAGATAGTAAAATTTTGTCCTGGACAGAACAGATTATTCAAACAAATTAAAAATTGTAAAAGATAGGTAGATATGTATAACGACAACATCAACCCACATACGTCCTTTTTGCTAATTATACAATATTAATTAACTATATACTCGTATATATACTCAAGATATGTGGCCGGTGGAAAAAATGGATCCATCCATTGAAGCCACGCCGGGAACAGTCTTGTGCACGTCGATGATATGTCCTGCTCGCGCCAGTACAGAGAGTTGGTCACCGCCAAAGTAGAGTTTCTTTCTGGAGATCCGATCCAGCGTCCGACAATAGTATAATTGAATCTAAAATTGTGTTGCAATTTGAGAATATTGAGCAACTGAGATCCGACTTTTGTCATAGACGATACTCCAGGCGTGTGCCCTTCTTCTGACAACATTTCTGGGTAAAATACTTTCGGCTGGTCTAAAATCACAGACACTGCTCGTAAAGTCAGATTGTGAAAATCCCACCGATTGTAATATTTGAATCCTTTCAACGAAACGAGCAAACCGCTGTTTCTCGTCCAAACGCCGACGCGATTCTTCTCCAAATTATTTCCTTGAATTCTGCCAAAGTTGAAAACATCAGTGAGCTCATAATGGGACCCACAATTTGACGCGTAGATTACATCGGCATCGATGCTTAAATTCAATCCTTGAAACGTTTCATCAGTGTACTCGTAATGCGTCATCGTACAATTATCAGAATCTTCGTTTAAAACGAGCCACATGTGCATGGAACCTAACAGCATGCTTTTTGAAGCCTCCTTGAGGGTATCTTGTGCGCTATCACAAGAGCCATCCAGCATGACACCAACGGCTGCATCCCACTGGTACAGCAGCCTCTCCAGATCTAACTCCAGGTCGCGATGGATCAGACCCACAGACACTCTTACGTTATGCTTCACGAATTTCTTTGCTATATTTTTGTTATTAAAATGATTATCGCATGTCAAATAACATAAATACTTCACTTCTCTATACGAATAATAATCGAAAGCCAAACTGAATGCATCGGAACGTAATCCGATAACATTTTCCAAATGCAAAAGTATGTAAATGAAATACCACATTATCCGAGTGAATAAATTCACCGGCTTATAGCGTGGGGCGATCGGG

>SinIR21a

ACATACTCTATTTTAACGAACCATCGACATGTGCATAAACATATAAAATTTAAAAGGCAGCAGATCGAAAATTTAAAATACTGGTAATGGAACTGTTATTAAATATATTAATTTTGAAATTCCTTTTTTATGCATACGGTCAAGAGATTGAATATTACCCCTCGCAGAATGTGTTAGATAATTCCGTTGTGAAAATTGGTAGTAATTCCAAACAACAGCTAATAAATACCGAGTATAATGAATTATTTTCTAAAAATGCTCACGATAAGATACAATGGAGATACTTTAATGAAAACGAATCGGATAAAATCAAAAATATATCTAAAAGGGCAACAGATCCGGTATTTTATGGACATCCGAAAACCACAGAAGAATTATGGAACGAACATTTTCTAAACCAAAGCTCAGCTTTCGATCAAATGCCATCACTTATAAAATTAATTCATAATATAACATTGACATATTTAAGTGATTGTATTCCTGTAATACTTTATGACAAACAAGTAAAATCACAAGAAAGTTACTTATTTGAAGATCTTTTCAAACATTTTCCAATTACTTATGTACATGGCTACATCAATGATGATGATACATTAAAAGAACCAAAATTATTATTCTCGGATCAAAACTGCTTACATTTTATAGTGTTCTTAACAGATGTGAAAACATGCACAAAAGTGTTAGGGAAGCAATCTCAAAGTAAAGTGGTAGTAGTTGCGCGGTCTTCACAATGGGCAGTACAAGAATATCTTGCTAGTCCACTCTCTAGAGTGTTTGTTAATCTGCTGGTAATTGGACAAAGTTTTAAGGATGACGACGATGATAGTTTAGAAGCTGCGTATATTTTGTATACACATAAATTGTATACCGACGGCTTGGGTGCTAGTCGGCCTGTAGTTCTTAATTCTTGGTCTCACGGCAAGTATTCTAGAGAAGTTAATTTATTTCCAAAAAAAATGAGAAAAGGTTATGCAGGTCATAGGTTTTTAGTTGCAGCTGCTAATCAACCGCCGTTTGTATTTAGAAGAATAAAAAGAGATGAAGAAAGTGGCAATCCGAAAGTCGTATGGGATGGCATAGAAGTTAGGCTGCTACAATTATTGGCAGACAGAAATAATTTCTCAATTGAAATAGTTGAGCCCAGAGAACTAAATCTAGGCCCAGGAGATGCAGTGGCCAAAGAAATAGTAACAGAAAGAGCAGACATCGGAATAGCAGGAATATATTTGACTGAAGATAGAATTCGTGAAATGGATGTGACTTTCGCGCATTCGCAAGATTGTGCAGCATTTATTACTCTGATGTCTATTGCATTACCTCGATATCGAGCCATTCTCGGCCCATTTCATTGGCACGTTTGGTTAGCTTTAAGCTTCACTTATATCTTCGCTATATTCCCTTTAGCTTTTTCGGATAAGCTTACATTACGTCATTTAATACATAATGGCGGAGAAGTAGAAAACATGTTTTGGTATGTGTTTGGAACATTTACCAACTGTTTTACTTTTGTTGGTAAAAATTCTTGGAGCAAAACAACAAAGATTACTACTAGACTGTTGATCGGTTGGTATTGGGTGTTTACAATAATAATCACGAGCTGTTATACTGGTTCTATCATAGCTTTTGTGACTTTACCAGTGTACCCTGAAACAGTGGACTCTGTCAGACAGTTGCTATCAGGATTTTATAGAATTGGAACTTTAGATCGCGGTGGTTGGGAAAGGTGGTTTTTGAATTCATCGGATAAATACACGAATAAGCTTTTTAAGAAAATTGAATTGGTGCCTAGTGTTGAGGCTGGTATTAGAAACACAACCAAGGCTTTCTTTTGGCCTTATGCCTTTTTGGGTTCACGTGCTGAGCTCGAGTATATTGTTCAATCGAATTTTTCTATGACTAAATCGAAACGAGGCCTACTGCATATTTCTAATGAATGTTTTGTACCTTTCGGAGTATCATTTGCTTTTCCAAATAATTCATTATACACAGCTAAATTCAGCAATGATGTAAGAAGAATGTTACAAAGCGGAATTATACAGAAAATTGTTGATGAAGTGCGGTGGGAAATGCAACGTAGCAGTACTGGAAAACTGTTATCGGCCGGTATAGGATCTTTAAACACATTACCGATCGAAGAGAAAGGACTAACTTTAGAAGACACACAAGGCATGTTTCTTCTTTTAGGTGCTGGATTTCTTATAGCGGCATCAGCGCTTATATCAGAGTGGATAGGCGGTTGTTCAAGACTATGCCGTTTGAGTAAGAATAAAAACCCGCCCACCAGTATCAATTCTGGAGATCATCTGATACCAACTCCTAAAACTGATACACAGAGTACGATAAACATAATTTCTGATGGCACTGATAGTAGATTACATTTTGATACAAGACCGCAAAGCGCCGATTCAAGAGATACTTTAGACGGTCAAATAATAAACGTTACTGAAGAAAATATCACAGTGCATGATAATTTCAAAGTTGATGGATGGGATTCAAGAAGATCCAGTTCTATAGATTTAGACAGAGAAGTAAAAGAGATATTTGAGAAAGATCAAAAGAGAAGAAGGATCTTTTCAGATGGTATGATCGAATTATCTGGTAATAAAAGGCATCCAACTGCGTCCAAAGGAGCATTTGGAGATACTGTAGGAAATTAGATATTTCGAGAAGTGTTTACAAAAGTAATATTTAGAATTTTGTGAAGCATATAGAAAAGTGACAGCCCACAGAAGTATCCCACTTCTAGGAAGTGGGATACTTCTGCGGGCTATAAAAAAGTTATTGGAATGCTGTATTTCTTTTGTCAAAGATTTCCCTGAGCAACAAATTATAAAAAATATGTATATGTACTATATAAATTAAAAATCTACGAATATAAAATAAAAAAACACACATTTATAATATTATTGGTTTATTGAAATTTTTAATTAATTTCCTGGCCCTACCTTTTACGTGGATTTTGGAATTAAATATGACAATAACTACAATAAAATCAAAGACAAAATTTGACATCCAACTCTTTATTATCATTCGAACAGTCATCAAAATAATTTATAACAATTTTCTAACACTGTCGATAAACATTAATCTTTCATTCAGCCACTGAGTTGTTTTTCATCGTACATTTTTATACACAGGGCAGAAATGGCTGCCTCAACATAGAAATAAAGGCGCCGTACAGACCTAAATTAATGCAATATTTGGCCCGGCCTTATCTAAATTAAAAAATAATTATAAACTATGACTTAAATTTTTGAGAGACACCAAAGAAAAATCTTCAACACCTCTCATGTATTATCAGTAAAAATATCGATAAAATACAACAGATTTTGCATTCTTTGTAAATTCAAACGAAAATATACCTAATACCTACCTAAGCATTTTGATACATATGTATTGCACAACTGATGTTTACAAGAGAAAAAATAATGTGAAATCGACTTGAGTATTCAATAAGGATATGTATATTCCTATATTCTATTTGGGTTGCTCACATTAAGCTGTGAAGCAATAGCAAAAAGAGCGACAGGATTCCGGAGCTCCATATTACACTAATGTGACGAAATCAAATGAGACTTCGGATATATGTATGTACGTATATAATTATAACTTATGACTACGCTTTCATTAAATGCAATGAATTACTCTTCCTAAAAGGTAAGAAGTTAACTGAAAAAAAAAACTTTTTGCTATTGGCACACAGCAGATTAAAATACAGGTAAAACATACACAAAAGTAGGTACATCTAGAAAACAAACGCAAACTTTTTTTACAATAATAATAAAATTGAACACACAACACAGCTGTGCGTTTAAATGAATAATAAAAAATGTTACAATAATTATTTTCTATTTATTATTTATTAATTAAACTTGCACTCATTCCCGATAGCAAAGCTCACAACCTCGGAAATAATGCTCCCAATACCAGCTTACTGTTGAAATTTTTTACACGCAACAAACAAATGAAATTTGTACAAACGTTAATGAATATAGATTGTGAGTTATACACAGTTACAACTTCTCAAATATACATACGTTTGCACAAATGTCATTATATTAGTTCGCGTAACAATTTCGTTTTGTGCAAACTACTATTATTTATAATAAAGACAAAGTCAATAGTTTGGACTTTTTACAGTGTACTTTCATGTATTGTTTAATGGACATCTTGTTCAGTGCACTCTGTGATAGGTATTTAGGTAACAGCGAAATGTACGACGTATTACTGTACAATTTTTCATATTAGCTATTCATATAATTGCTTCAGAATGTTGTGGTATATAAAGCAATAATTAATTTTTTTATTAATAAAGTGGTATTTATAGACTCATATAGAGATGGTTTGCTGATTCTGCGGTGTAATGACTATATTTAGATATTTCAATATGTGTGGATAGAGCTTTGTATGTATAGTGGACATAAATAAATGAACATCGTCATTATACTATCATAAATCAACTCACTTGTTGAGTGGCCTAAAAATCAAATTTAATTAAGACTACCACATTAGACATTTTTATCATAGGTATATAAGGCTGTCACTGCATTGACGGCGAGCGGCAACGCAACGCGATGAAAATGAGACTGAATGCATAGAAATATTGTTAGCGTTGTGCAGCGCATCTTTTGGTTGACTTTAATTTTTTCTGCATAATACTCGTATATTATATACATATATTAGTGCACTTCATTGCGCTGCGTTGCGACTTGCGCTACGCCGCCGGTGGAGTGACAGCCTAAACAAGTACAATCATATGTCAGCTCCAATAGCTGACATAACATAGCATAAAATGTTGTATGATCCGATTTTATCTTGGTTATCCTAGAAAATGTCTAAGTAATGGTCAAAGTACGAATAACGAATACGATTCTGAAGCAATTGGATAAAGTTAAGGGTCCGATTCAGATATAACAAGCTTCAATTAAAATGTTATATTAAGAAAATTTCGCTTTATCGCCAAACAAACTTGATTGAGCAAATTTTAATGAACAAAGCACGTCGTTTTCATTTTGAAAAAGGAACTATTAGGAACGAACGACCGAAAAAATATATATATAATTTTTTTTTCAAATAACGCAACTTTAGAATCTTCAAATACAAGGACTTGTAACAGTGTAGTGTATTTATATCACATTAACAATTTAATCTAACATTTAAAAGTGTACATGATTTTTTTTTTCATTATTTTAAATTAGGTAATTCCCTAAACACGGGTCAACATTAATTATGCTCCGGTATTAAAATATTAATTCGTTTTAAATACTAATCGTGTATATTATTTTACAATGCGAAAATACATTTCTTATGTGTTCAGACTTTTGAACTTCAAAACTGATTACTTCCCTGTACAAATTTAACAACTATACATTAACATTTTGTTATTGTGATTTGAAGAATTTATTTCTAAGGATTCTCTTACAATGCTAACGATGCAAAGATTTTATTTTTTAAATTTACTTACAATTTATACAATTACAATCACATAGTTTCAATTTTTTTTTTCAACAGTTTGACTGTTTTCAAAAATGTGGCATACAAGGACGATTGATCCTATAATGGCAGAATTTTATTAATCATTATTTATAATCCGCAGTTCTTTTTCCACGGACAGGTATGGATACAAAGATATAATATTTTCCTATTTTAATGACTATAGGTACATTGTACAGTCATAACAAATTTTTTCTTTTTTTATACTTAATATTATCAGGAAGTGAAAAATATTGAAGCACAAATGCTATCGTATCATACAAATTTGGTACAAAATGGTACAAATGTGGTACATTTAGGCACTGTGAGTAAGGCATCTACAGTGAGATGATATGAGGGAATAGTCGGAGGATTTACAATTGTTACGAGTTAAAATAAGGGAAAATGATTAGGTGGCTCTAAAGTCGAATCTACGGGAATAAAAAAATCGTATATGTAAAGATATTTTGATTTTGTTAAAACAAAAATTGCTCGCCGCAGATTTATTTTCAGGAGCCCACAACGGTCTTGTAGTTTGATTATTATTAACAATTAATGTAATATTATTGTACGTCCTATGCACCTCCATCGATTCTCGCCCGTGAAAGAAAAAACCATTTTGTATATTTTTTGTCAAAACTAAGCAATTTACGTCAATGTAGACTCTACTTTAGCAATGTGCAATGAAAGCGGAATGATGGACGGGAAATGTGGGAAATGATTTTGAACCATGCACACATGGATTTATTTAAAACTAGAAATCCCGTAAATGGTCAA

>SinIR75a2

TATTGAATAAAATTTTCATGGTTGTTTTGTATTAGGACTCTACAGAAACAGTCTACAGTCGATATACCTATTATAGTTTTTATTATTACATTTCAAAATTATCGCCAAGCAGAGCTATATTTATGATAGTTGATCTGTTCTGCAATTATTCACAAAATCTCAAACTTTTTCTTTTTTCTTTAGATCAGCAGTCCATTCCTCGTCGTCCTGCGACGACATGAGATCGTGAACCAGTAACACCATGTAGCCGCGTTCTCTGTTGCGGAAAACCTTCCAGAAGCAATGGTGAAAGTTGAATGTCCTGGAAACGTCTGCACCGATTTTCATGAAATATATGTAGCTGTGGGTGTTAAAATTGTAATTCGTTCACTCGCTTAGGAGTTGAGAGGGTGCAATGTAAAGAGATGTTCTTCTTCGTATACTATATTTGCGACTAATTCCAAAATGCATTCTAATGCACAAGGCCATCGTACCTAACTTATATCTGGATCTTCGTTTGTAACTTATTATGGATCTTCGTTTATAACTATGTTGCTTAGTTTTATGTATATTTGTAAGTGTTCAAATAGTACAAACAAAACCAACTTTTAAAAGCCAAAGCCAACATTCAAGCCAACAAAAAATATCCCATTCAAAAATTATCCACCCCATCTAAAGTTATGATGCCACAGACAAACAGACCTACTATTGCATCAGACGTACAACATTCCTCTTTTTGCATTGCAAGTTAAAAAGTGACCAATTATTGTAGTGATCCGATCCAATACTTAATTAAACCTGAGAGTGGCCTTTTAACACAAATTCTGAATGGTTTTCATTCATTTAAGAAAAGTTCAAAACCTTTTTTCGTGCAATATAGTATGTGTATTTTATCTAATTTCTCGAGTTTATTTGAACATATTTTATCTTTAATCAGGTTCAGTGATGTTTATAACTAAACAACGTATAGACGTCTTGGACTACTTAATACATCCCACATCTGGACTAACGGTGAAATTTGTGTTTCGAGAACCACCATTGTCATATCAGAACAATTTGTACTTGTTACCGTTCAAACTCAACGTCTGGTTATGTATCGCTGCATTCGTGATGATATTAGCGTTCATCCTCTATGTAAATGCATTTTGGGAAACCCAAAAATCTAAGAGCAATGACAAGGATAAATTAGATAATACCACATTGAAGCCAAACGTGAGCGACATTGCCATTTTTGTTATCAGCGCCATGTCTCAACAAGGAAGTGCAATGGAACTGAAAGGAACCCTCGGTCGCGTGGTAACGTTCATTTTGTTCCTCACATTTCTATTCTTGTACACGTCATACTCAGCCAGTATAGTCGCGTTACTGCAGTCCAGCTCCAAACAAATTCGAACGCTTTCTGATCTTCTACATTCAAAATTAGAACTCGGTGTGGAGGATACGCCTTATAACAGATATTATTTTTCGACTGCCAAAGACCCTGTAAGAAAAGCAATCTCTCAAAAAATTGCGCCTCCAGGGTCTAAGCCGAATTTCTTGAATTTAGAAGATGGAATTAAAAAATTACAAAAGAGACCTTTTGCTTTTAATATGAACCTTGGTACTGGCTACAAAATTATAGAAAGGTATTTTCATGAACACGAAAAATGTGGACTTCAAGAAATAGATTTTATACCAGACAACAAACCGTGGCTGGGCTGTAGAAAATACTCACCATATAAGGAAATTTTAAAAATTGGATTATACAGGCTTCAAGAGCATGGTCTTACAATGCGTGAGAATCGTCTGATGTATTCCACGAAACCGGTGTGCACGGCTCGCGGTGGCAGTTTTGGCTCCGTCGATATCCTCGACTGTTACCCGGTGTTACTCATGTTACTTTACGGAATGATTCTTTCTTTCTTTTTATTATTTATAGAAATTCTGTTTCATCGTAGACAAAAGCTGCTACGTAGAATAAGAGATATTCGTTAAAATAGGTATGCAGTTTGCTTGTATGAGTCAAAAAACAAAACATTAAAAAAGAGAATTTTCTAAAGTAGTAAATACTTTAATATAAATGTATCGACGATTTGATTAGATTATATGATTTGTTGTTATTAGTCAGCGGTGTAGGCTATAAATTAACATGTCCACTAAGTATTATGTAAAAAATTTAAAGGGAGGCAAGAGCACGCAGTAGTGTTGTGTTGTGATCCAAACATCTGTACATAGATAATATGATACATACATATACGTTTAAGTATATATAACTTGTTTATCAAAAGAAACTTAAATATATTTGAAGCCGAGTCAATTTGTATCGTTTACCAATGTTGATATTTGGACAGCGTTTTAGTGTTCTGCTCTGAGATTCCTCCATACGACTCATACTAAAGTCTGCCTCTACATAGAACTACACTAGTGGTAATTCTATGTTTCAGCTGTATGTAAGAGTGCCATAACGTAGGATTACGTAGTTTATCACGAAAATTAAAAAACTAACCTTTATTAGGTCGCAAATTATTTGGTGTTAAGAATTTCTGTCAATTTCTTCGCGCTAATAGGTAGACGCGCGTGACAGAAGGGATTGGAATAAAATAATTTATCGGACAACGTCCGTTCACAATCTGCGCTATATTCTGACTCACTTACAAAACATAATTATGCCTTAT

>SinIR76b

CATTTAGAACAAAATCCTTAAATATAGTGAAAAAAACTTAAATATAGAATATTAATAATGAAATATTAAATACTGAATCATGTGACCATAACAAAAAAAAAAATGTAGTAACAAATTTTTTTTCATCATCAGTCATGACAGTTTTTATAAGAAAAAAAACTGTCATGACTGATGATGTGTTTTGACAGGCTCACCGCAATAGAAAGGTACTATCCTCTCATGTTTATAACTTCTTTTATGAGTCAGTCAATTAACTTCAGTTATGAGTCAGTCAATTGGGACTTCTGTCTTTGTTATAGATACGCAATAAAAACCATTTCGCCTTCACCGATTTGCTCGTTTTCCAAAATCACTATTTCAGTGTCTAAAAATATTGATATTAAATTTTTCATACGGATAGCTGTAGTAAAAGTGTACGATATAGGATACTTTTAATCTGATATTCAGTAAACAGTCTCTCGGGAAACGATTAAACAAAACTGCGTAATAATAGTATATCAAAGATGGCGAAGCTGTAGACAGAAGATAGCTTTCGATAAATCTGAACATGTCGGTGGTCAAAATATGTCTTGTTTATACGTATAATTATACATAGTACACATATATTATAAAATTTGCATTTTTACTAGAAACTTGTTATGGGGATATAAAAATACATATAATAATAGGTAGGTATATACCTATCTATTATTATATATATTATGTATTTATTATTTTTATGAATGATAATAATATATAATGTAAACAGCGTACATGTGAATACTGTTTCTTATACTTAAAAATTGTGATTATTTTTCATAGATATTATAAAAGAAGTAAGACATGAAGAGACGCGTCATATATTGGCTAGCATTTTATAATATATGATAAAATATGTTTCTTTGTTTTAGAAGAGAATATGTGCGGGTAACACAAAAACATTGAATTTTTATTTTTAGATAAATAAGTATCACGATAATTGTATTCATTTGCACTCGTTTGATAAGACAAAGTTAAGGCTCGCATTTCTTTTAAAATCTAGATTCCGATACAAAGTTATGATCTACAATATTTACGGTGGACTTTGATACATTGTCTAACAACATAATAATATGATGCTATACGTTAACACGATAATTTTGATACACAGTTATCAAAATACACATCAGAAAATAAAATAACGTCATAATACAATTATCATATTTAAACAACTTGTGTTATTTATTGATAAATTGTCAAAAACTTGAACATCTGGATATGTCTTATGAATTATTTTATTAATGTTAATAATACATAGATCATGTTAATGACAATAAAATAAATTCTAAGACAAGACATTGAAATTTGTAAACATTTTTTTTTTTCGCAATAGGTTGCTTCATAAATTTTAGAATAAGGTTGACCACTAGATTTAATTTCCGCCGAAGCCGCAAACGCACTGGACGTGCGTGATGGAATATCTCTCGTACCCTCTCATGAGTACTAAACGCTCTCAACTCATATAGAGTTGATTCGCAATGTGCAAAGCCAAAGTTCGAATCTCATCTGAGATACCCTCAGGCTAGTTGCAGTTTTTTCAGCTTGTGCACCATAAGTGTTCGCCCCCTTTTATCTAGTAACGCGTAAAGCTATCGGCAATAATGTCATTTCAAAAAATAAAAAGCATTTTATCTTAACCTATTGATAACAATGGCTTTGCGTTAGGTATTCACAGTTTATTCGTTATATACTCTGTAAATAAATATAAATAAAAAATCATGTATTCTTAAACCCAGATTTATAAGAGTTATTGCAATATAGATATTTGTAATTTTGTTTCTGTTCATGCGTTATTTTTTCAGAGGAAAATAAAAACGCATACCGCATTGACATTTCATAACTAATATTTAAGTAATATAATTTTGTTTGAATATTACTTTATTATTATTATAGTAACCCTTATATGTATGTCTGATCTTATAATTATAATATGGATAGCAGCTTACTGCCAATTTAAAATTTATAAAGTCGATAGATTTATTAACACTTTTTGCTATAATAAAGGGTAGTTTAGCTTACAATTAGCTATTTCTGTAGTTAAAAAGATACTATCATAGTGAATTTGAGCCCAAATTCTGTGATGAATTATAATTATTGTATAATAAAATAACATTAGTTTCGAAATCGGATCGTCTTTAACATTTTTAGGTCTTCGTTCCGTGTTGAGAATATTGTTTTAAATTTTAATTTTCAATCAAATTATTTATTCGTTATGTTAGTAATTAATGCACTTGAAACACATTTGTGTTCTTTTTCTACACATAATCAATTTCAAAATCATTAATTACCTATTTATATTATTTTTGTTATATTTCTAGTGTATTGTAAATTACATATAATGTATTAAAATTATAGAAAATGATGAGGTTCTATAAAAAAACAACTTAGAACGTATGTAAATGATGAATTGTAGGTATTTATATACGGTTTATTAAGTAGGCGGTTCTATGACTGACGTACGACCACGATCGATCGTGACGATCAGTCGCTGCTCAATATAATTATGTATTATATGTAGTCATACTGTGTATTTATGTACAAAATGTACATAGGTTATTATTATTATGCGTTGTACGGACGCCGCCTTAAATGATTACGTTCGTTCTAAGGGCCTGTTTCACAACATTTTGTCAGAAGCTGTTTTGTTGGCAATTTACAATAAAAAATAATAAATTAATATTAATTATTTTATATTTATTAATAAATCTTAACAAAAAGTCGTCAAGCGGGCCCCATATTATTTATAGTTCTTTTTTTTTATTTATACGATAAGTAAATAATTTTAATTGTTTGCAAAATGAAATGATGTTGAATATCACGATTTGAATGCGAGATGAGTTTGTGCAAATGTATTTTCACGGTTAATAAAAATATGTATTTGAGATAATTCTTTTATTTTTAACATCCACAATATAATATACATAAATGTTCTGTTCCAGATTTAACACTATAGTGCTTGCATTGTGCATATATTCGTTTAATATTAATTTTATAATAGTTACCTCATTGTAATATAATATATCAATGAGTTACCTACTCTTAATTAATAACTTCTCAATGACACTCGAATCTAGATTACATAAATATTTTCTCTAAAAATATATTATCTAAAATAGTTTTCAAAAACTCACAAAGGCAATAATAATGAATACAAACACGAGAGGTATTTTTCATGCAAACTGTTATGATGCAATAATATCAAATTCGTTAAAACATTTTTACCTACTTAATACTTATTTAATTCAGGTCAAAATATTCAGGATCATCAACATAAGTAGGTACGTACTAACGGTAGGCGATAATAATAACAATGAATTTAAAAAAAAGTCATTCGATTATGCATTGTCGAACTAATAGCAGAGTCTTGTACATTCATGAGTGAATTGCGCAACTCTGGTGGGGCGGGACAAAATTGCCTTATTTTTATGTCGAAAGGTGGTAAACGAATTGGCGATTCGCATAATGTTGAGCGTTCATCGACGCCCATAGACATTTGTAATCGCGGAAACACAAATGCATTACCATCCTAAAGATCAGAGTTTGGCAATTATATGAACAACTGAATGTAACGGGCGAGGGGGGTGGTACTGGCACTCTTATGTACAGCCGAAACACAGAACCACAAAAATAGGGAGAGCACAGAAGGTAAACTCATGCCACGACCACGTGTACTTCATTTGTTATACCTACACGCCGACACGCTCGGCGCTCGACCGTCGGAGCGTCGTACGTACTACGTACGGCTCAGATCAACTACTGAAAGTAGGTGAAGTAGGTACTTAGGTAGATATTTCTATGGAGTTTTCAGATTATGGATCTTTACATTAAACTTCTATAGATCTTTAGATTATAAAATGTATTTGGTTATATTATCTTTTTAATAGGTAATACGACTGTGTAACAACAGCGCAGAGAGAAGTAAATAAAAAATAGTAATACAAAGATAGCGTAGGCACTTACATCGGAATCCAAATACCTCCTGACATCCGTTTTACTTATTTAGTCCGTTGAAAAACTAATATATTTTTTGTTATCATAAGCAAATAACCAACAATTTTAATAAGAACCACTCGTCCCACACGGTATAATTAGCCAATCAGCACAGAAGCGTTACAAAGAAGGAAATGTACTCTGTTGCTCTTGCTCTTGCTCAACAGCTCTTGCTTCTATAAGAGTTGTATATAATAAGAGCAAACATGTATATACCTACTTTGTTTAGTAACTCTTGCTCGTATAAAAGTAAAATTGCTCTTATACGGTTTATACGAGCAATACAAAAAAGGAAAGGTACTCTGCTTAGCAGCTCTTGCTCCTATAAGAGTTGTACATATATAAGTAATAAGAGCATAAATGTACCTACATACTTACTCTGTTTACTAATTCTTGATTGTATAAGAGTAAAATTGCTCTTATACGACTTATACAAACAAAAGCTGCTGAACAGAGTACTTAGGTAGGTACATTTCCTTCTATATAACGCTTCTGTGGTGGTTGGGTATTATGAATTCTTATGTCTACATACGCCTTTGACGTTGATATAGAAACGCAGACGGCGTCCTCATTGGTATCAGCCTGGTGTCCCCGCTCACCGTGTCCACGACCCAATATTCTCTGCCGTTAATTATCTTCGTTTGGAATTGATCTGTCATCTTGTACCTCGAGTTACGACCGAAAAGTGATTCGTAGGGCGGCGGTTGGGTTTCATCGTGATTTTGGAATCTAACTCTATTCGCCCTCGTCCGTTTCGGTCTTGGTCCTTCTGTCTTTAGTCGTATATGGAAACATTTTTTTATTATAATCTCCATTATAAACACTGCTATGGCAGATGCCAGACCGATTACCATTATCAAATAGGTCATCATCAGATCGCTGTTCCGGAGACGTCGGTCTTTTGACTGCAGGTCCAGAGGGCAAATTTTCGTGCTCGGCAAATCCCGATTTTTAAGGAAATTCACTATCCCTGCTTGCAATAGATTCGTGAGGATTGGATCGAAAAGCTTTTTCAATTTACTATTTTTTGTAAATATGAAAGCTCGATTTTTCTTCATGAAGGAATTCGGAGCGATGACATAAGTACATCTACTAGTTTCTTCGATCCCCTGCCTGGCTTTGTTCAAATAATCTGCATACATCATGTGGTCTATGCCGGTCCGATCTTTTACAAGAACTGCGCCTCCTTTCACCATGGTCAGATAGTCTTCGCTGTTGGACACTGAACGGAATTCTGCTCGACCCTTTGATATCATTTGATTTAGATAATGAATATCTTCATCGGGATCTTTAACAGAATATTCTACAGTTCCGCCTTGCGTGGCGACCCATCGATAGTTTTTCTTATACAAATCCCTTGGCGTTTCTATGTCTAATGTAAATTTTGAGAGGGTAAGAAATGCGGTTAGGTTAGCGGTGTAGAAAGCAGACAGTAATATTATGAACAGCCACCAAGTTGCGAAGAGAACTCGGGTTGTGTTTGCCTCTGGAGACAAAGTGGTCCCCTGCTTGATGAAGGCTCCATAAACGAACCAACAGCTCGGCGACAACGGGATGTAGCGCTCGTGGTCTTTGATTAGCTTCGAGCGCAGGCGCGTCAGTAATGTGATGCACGGACCGTAAGACAACACCGCTGTTAGAATTAGATACCACACGTGGCTTTCAAACGGCGCGAGCAGGCCTGATCCTGCGGCGGACTCAGCGGGCCTCTTCAGCATCATTACCCATACTCCCTCGTCGATGTCAACCGAAGACGAAACTAGATCTTTCAACTTATACAATGTCGGCATAAACGCAGCCACCATATCCACTTTACTGCTATTAACAAGACCTATCAAAGAATCTTCTGGTCTGGACCCGCCAAATTCAAAATTGTTATTAGGGATAACAACTTCAAATGTGAAATTGAATTTTTCACGCAAAATATCTATAATAGTGAACGCAACCCCACGACCTATTAACGTTCCGTTGTCTGCCCTTTCTGTCCAGCTTAGCGGATAACTGTTGTATGTGCCGATTTTTAAATGTTTGCCGTTTAGTTCCTCGGCCAAAGCCAATAATTCGATTTGTGTCTTCGTTAATTGCGATTCTATTAATGGATTGTCATATACGGCCTCGCAAAACGTGGCATTGCAAATTGATGAAATGATAAGCTCCATGCCGGTGCACGTGGCATTATAATGCGGTCATGACGTTCAGTGGTAGTGGTCATTTGGTGGTCGTACGGCGAGTGATGATCATGACGGTGCAACACTATGTGACAGTATCCGCCACCTTTGACACATTGTCGATGATCGTCATGACCGCATTATAA

**SNMPs:**

>SinsSNMP1

ATCCTTACAAATCTCTGCCAATCCGCACTGGGCCCGCGTGGTGGAATATGGGCCGCACTCTCTCACAATTGACGTATATGGTGTGTTGATGTTGTCAGTACCGCTTTGTCGTACGACTGTACTGCACCCCGCCCATAGATTAGCCCCATTGCAATAGAGTGGACAAGTATTCCAACAGCTGTCTCCTCATACGGACCGCTGCAGTCAGATACTATTCGCTGAAGATGAAATTGCCTAAGCATATGAAAATCGCCATGGGTGCCGGAGGAGCGGCCGTTTTTGGCGTATTGTTCGGTTGGGTTATATTCCCAGTCGTACTCAAAAGCCAACTCAAAAAGGAAATGGCATTATCACAAAAGACAGACGTAAGACAAATGTGGCAGAAGATACCATTCGCTTTGGACTTTAAAATATATTTATTTAATTACACAAATCCGGAGGAGGTCCAGAAAGGCGGAATTCCAATTGTAAAGGAAGTTGGACCTTATCATTTCGATGAATGGAAAGAGAAAGTGGAAGTAGAAGATCACGAAGAAGATGACACCATCACGTATAAAAAGCTGGACGTGTTCTACTTTAGACCAGACCTTTCGGGCCCTGGACTAACTGGCGAGGAGATAATTGTTATGCCTCACGCTTTCCTGCTGAGCGTGGTAACTATCGTATCTCGTGACAAGCCAAGTATGTTGAACATGATAGGCAAAGCCATCAATGGTATATTCGATAACCCTCAAGACGTGTTCATGAGGGTCAAAGCGATGGACATACTTTTTAGAGGAGTGGTCATCAATTGTGCAAGAACTGACTTCGCTCCAAAAGCGCTTTGCACGGCACTGAAAAAGGAAGCAGTCTCAGGATTGGTAATTGAACCAGACAATATGCACAAGTTCTCCATTTTTGGTACGCGCAATGGAACAGTAGACCCACACGTGGTAACCGTTAAACGTGGGGTGAAGAACGTGATGGAAGTGGGACAAGTAGTGGCAATCGATGGTAAAACCCAGCAGGACAAGTGGAAAGACTCTTGCAATGAATATGAAGGTACTGATGGTACCGTCTTCCCGCCTTTTCTGACCGAATCCGATCGGCTGCAGTCGTTCGCCAGTGATCTATGCAGGTCATTCAAACCCTGGTATCAAAAGAAAACATCCTACAGAGGAATAAAAACGAACCGATACGTTGCCAACATTGGTGATTTTGCAAACGATCCAGAACTACAATGCTATTGTGAATCTCCAAGTCAATGTCCTAAGAAAGGGCTGATGGACTTGACAAAATGCATAGGAGCGCCAATGTACGTCTCCATGCCACATTATTTGGAGAGTGATCCTGAATTATTGCAAAATGTGAAAGGTTTAACTCCTGACATCAACGCGCATGGCATCCAAATTGATTTTGAACCTATAACAGGCACTCCGTTGGTCGCGAGACAGAGAATTCAGTTTAACTTGCAACTTTTGAAGAATGACAAATTGGACCTTTTTAAAGATCTGCCTGACACGATAGCGCCTCTATTTTGGATTGAAGAAGGTTTGGCGCTCAACAAAACGTTCGTGAACATGATGAAACATCAGCTTTTCATTCCGAAAAGAGTAGTCGGTGTTGTTCGTTGGTTGCTGCTGTCTTTTGGCATTCTTGGCGTCTTAGGAAGCGTCGTCTTCCACTTCAAAGGTAGAGGTGTCCAGACGACCGCATAATGAGTTTCGCCGTATCATCCGGATCTGCCTCCGTAACGAAAATCAAGCCTGAAGATGTGGAACAAAAAGAAATCAGCGTCATTGGCCAAGCTCAGGAATCACCTAAGTTCAGCATCTAATCCAACATCAGACGACTCTTGAGCCTTTATGTGCGGTGGAGTCGTCGATACGGTATCGTTCATACTAGGATTTGGACGCAGACTTTTCGTTTCCTCTTAAGACAATCAAGGCAGAAGCGTTTGAAGTTTTAAATAGGTCGAGAATTATTCCAAAACGTTACCTATAGAACTTAAAAATAATTGTTCATTACTTTTGTAGATTTAAAAACCACAATTATTGTAGAAATGATCGTATTGTAATGGACGATTTGTAGAAAATTTGCACAATCGTAAATAATGTTTCATATCACTGTCTAGTCTATTTTCGTACTGTAGAATAAATGTAACAAACAAATTATTTTGTTAGTAGTTTTTCAGTGTTATATACACGGTTAGTTTTCATTCGTTTTCATCCCATGGCACAGTGATTCCCTTTTAGTTGTACTATCGATTAAACTATCGATTAATAGTGTAGTTTGAAAAAATACATTGGAAATAAAGAAATTGAAAGTTGAGATAATTATCTTTCTACGACGCTAGAAACCGTAGTAACGCCGCGCGTGAGCGCGGCTCGCAACTCGCAAAGCGGCCAGCTGGGAACGAATAGAGTGCGTTGACCCGCGCGCCGCCCGGTTGCGAGGCGCTCGCACCACGATTGGCGGATCGGTGCACACGGCACCTTTGATACTTTGCACAGTAACTGAAAAGCGTTACTATTAATTCTCACAAGTTCGAGTTGAGAGTGATTTATTTTTTAATTTTTATTTTCTGTTGCGTTTTTAAAAAAAAATTAACCCTACTAATCGATAGTACTACTGAAATAGAATCACTTCGCTGCGGGATGAAAACGAT

>SinsSNMP2

TTTTTTTTTTTTTTCTCAACAATCAATAGCTTTTATTTCTAACAGAAGCACTAACACCCGACCTTAATATGAAACTAACTGAGTTACTGTTTCGAGGCCAACCAAGGATCGAAGATGAGCGCGCCAATATAACAGCGCTGATGATTGTCAGCGCTATACCGATGACTAACAGCATCCAGCGGGCGGTCTCCACGTAACCCAACATTGAATGTGATTGGCGAAGTTCATTTTGTATGGATTCTGGTATTTCTGCGCCCTCTTCAATCCACAATAGCGGGAAAAGTCCAGTAGGTACATTCTCCAATTGTGGCGCATCTTTCACATTTCGTAGTTCAATATTGAACTGAAGTCGTTTCAAACCTTTCAACACTACGCCCGTCACCGGGTCAATATAGACGTAAGAATTGTGTTTCTCCTTGTCCGGTTGAATTCCCTCGTCGAAGTACTCCAAGAGTTCCTCCGAAGCGAGGTAGAAATGTGGCATGGAGGCGATGGCTGGTGCGCCCTGGCACGGCATTAAGTTCAGCACACCCATAAGCAAGCAACCGTCGTGGTTGCCACTCCAATTCCTTTTACAGAAGCATTTATTGTCAGGATTAGCGCTTTTCGCTGCCAAGGCGTCGGTCATTTCGTAATAATATGTAGTTAAGTTGAACAAACTTCTTTTGCCTATTAAACCGACGTACATCGATCTGCAAATGTCCGGTTCGAATGTGTACAGTCTTCTCGGCACGTTGTTTTCATCAATAGGTGGGAATATGGTTGAGTCTGATCCGTTGATTTGACCGCAGTAGGGGTCACCCCACTGGCTCATAGACGTTTTACCCTTGTAGGAGACAATGTGTCCCAAATCGTACATGTTAACTCTACCTCGCACTATTTCGTATGGACCAATTTCTGTTGTGTTTAAGTGAGAGAACATTGAGAAGTAAAAACCATTTCCGTCCTCAGAAGGACGCATCGTCGGTGGCATATCCGCTTTGATTTTACCGCATACGAGTCCCAAAGCAGAATTATCTCCTACGCAGTTCAGATAAATTCCGTCAAAGAATAAATCTTTAACTTTGACACGCAAGAAAGGATCAGTAAGGTTGGGGAAAAATTGTTCCAGCGCTTTGTTGATCATGCCTATCATGCTGGGCATCAGCTCGTATACCGACAGTATCGCAGCCATATAAGAAAAATTGATGACTGTCACCTCATCATCTTCAGACAGTTCCGCGGAGGCATTATTATCAAACACAAATCTCTTTTTCACGGTATATTGAATTGTATCGTTTGGTCCATATCCTAATATTGTTTTTTCTCTGTATTCTTTATAGACATAAGGTCCTATTTCGGCTAATTTGGGCTTGGCACCGTTGTTGACGTCTTCGGCATTCGTCACGTTGAAGACGTATATCTTGAAATCGAAAGGTATCGGCATTTTCCGCCACTTCTCGAACATTGCCGAGGAGTTGTCCAGTTGTATATTCTTTTGAATTTGATTTTGGATGATCTTGGGAAATCCCCACGCGGCTAAAATGATAGCCACCACGAGCACCACCAGAGATATCCCGAAAAACAATCTCGAATGCTTTCCAATCATTTTATTTAACACAAAGTTTTAATTTAAAATTAAAAAAAAAACACCAACACTATGAAACGCAAAATTAAAAACAAATAGAAGAAAAATTACAATTGAGTTTATTTAAACACTAAATTACTTAATATAGTAATTATTCAAAATGGAAACTCCAAGTTTAGAAAATCGATTTTCTAAAAATCCAATTCATTTCTTAAAATTCACCATAAACGAGTCTTACTTGGGACGAATCTTAGCAGATATTATCACTATTCTGTCTCTCAAACGACGCATACGCGTACAATTTTCCTTGTCATATTTTTTTCCTTTAAACACAACAGAATGGCGTTTATGCGGATAAATGGTCAGACATATTAGGTTCTATTGACACAGCAGGTATGATTCTACGGTACGCGATAACATAATGCTTTAGCAATCCTTTATCGATGAATTGAATGTATAAAATGGCATTTTTTTTTCTATAGATGCGACTGCGTGATGACCCTGAATAATGAGGAAGCGTGAACTAATTTCATGCGATGGCTTTTTAATACCTGTCCATTTAATGTATTTGTTGTATTGTATTGTACATTTATATTTCTGTATGTACAGATG

**ODEs:**

>SinsCEX1

CAAATGGGCACTAGCTCCGCCAGCACTATGGCCGCCTATAGTCACTTCGTTCGGGTCACCGCCGAAGGCTCGAGCGTTGCGCTGCACCCATCGCAGTAGCGTGACCTGATCACGCAAGCCATTGTTCCCGGGAATGCTCGATGAATTGAGAGACAGGAAGCCGAACACATTGACCCTGTAATTAAAAGTGACGACAATAACTCCTTTGCTGACGAGGTACTCGGGGCCGTGCACATCGGCGTCGCCAGAGCCTAGAGTGAACCCGCCTCCGTGTATGAACACGAAG

>SinsCEX2

TGGACGCGAGTGAGGAGGGTCCCTCATGCCCCCGTCCTGCGTCGGAAAACTATTACCTCGACGAGGACTGTCTCAGGCTAAACGTGTACACATCCAGTAACAACAGCACCAACCCGATGCCGGTGATGTTCTTTATCCACGCGGGCGGGTTCTACACGATGTCGGGGCGCAGCGACTTGGTGGGCCCCCACTATTTGCTCGACAGGGACCTCGTGCT

>SinsCEX3

ACAGCTTCACCAGCACCGGCCAGTGTCTCGCTCGCGGGACGAGGATAACGTGCAACATGTACAAGTTGTTGTCTGTCTGTGTTTTTGTGTGCGTCGTGAGTGCGTACGCGTACAAGCAACAACATGCCGTCAACAAAGGGCCTACAGCTTCACCAGCACCTACTGTTGCAGCTTCATCGCCGCCAACACGTGGAGATGGCCCCGTGACAGTGACTCCATCCGGGACGATACGCGGCTCCTGGATGGAGACGCGCAAAGGGCGACGCTTCCAGGCTTACCGAGGCATCCGGTATGCAGAACCGCCCGTTGGCGAGTTGAGGTTCCAGCCTCCCAAAATGATACTGAACTACAAGGGAGAAGTGGACGCGAGTGAGGAGGGTCCCGCTTGCCCTCTCCCCGCGCCGGAGACCTATTACGTCGACGAGGACTGTCTCAGGATCAACGTGTACACTCCCACTAATAAAAGCACTAAGCCGCTGCCGGTTGTATTCTTCCTCCACCCAGGCGGGTTCTACGCCATGACGGGGCGCAGCGACCTGGCGGGTCCGCACTACTTGCTCGACAGGGACCTCGTGCTCGTCACTATTAACTACCGGCTCGCCACGCTCGGTTTCCTGAGCACGGGCGACGAGCTCGCACCCGGCAACAACGGCATGAAGGACCAGGTGGCAGCGCTGCGCTGGGTGCAGCGCAACATCGCCGCGTTCGGCGGAGACCCCAACCTTGTCACCATTGCTGGCTGCAGCGCCGGCTCCATCAGCGTCATGCTGCATATGATCTCGCCTATGTCTAAAGGTTTGTTTCACCGTGCGATTTCAATGAGCGGCTCGCCGATGCGCAAGGAGCGTGCAACGCCGCGCCACGAGCGCCATCTAGCGGTGAAACAGGCGCAGCTGGTCGGCTGCCCCACGAACTCCACCCGAGCCATATACGACTGTCTCAAGACCAAGCCTTGGAGGGAGCTTGGCAACTCCTTGTCGGGCTTCTTTGAGTACGCATACGACCCAGTAGGCATATGGTTGCCTGTCGTAGAGCTGGACTTCGGCCAGGAGCGGTTCCTGTCCGTGGAGCCATTCGAAGCTATCAAGCAAGGGAAAATGCATCCCGTGCCATACATCATCAGCCAGACCGAGGACGAATTCTTCTGGTTCGCTTTCTCAATACTGAGAAACGAGACGCTCTTAAAGGCAATGAACACAGAATGGAATCGCATGGCGCCCATATCGTTCATGCTCCCCCAGGAGAACTTGGAAACCGCCCTAACATCGCTGCGGAAGGTCTACCTGAACGACAAACTGCTGGCTAATGACGATGTCAGCGCCAGGAACTTGGGCAGACTGTACGTCGACTCCATTGAGTCGTTCCCAGTTCACAGACTGGCGAACCTGATGTGTCGATACTCATCACAGCCGGTGTACTACTACCTGTTCTCGTACATCGGCAACCACAGTCACTACGAGGACCCCACCACCGGGAAACCAGTCGGCGCCGCGCACCACGACGACTTGATCTACCTGTTCACCCTGAGCTACCGCTTCCCGACCATCCCGCTGTCCAGTGCGGACGACACCATCGTGGAGAGGATGACCGCCCTGTGGTACACCTTCGCTAAACACGGGGATCCAAACCCGCGCGGTGATTTACCGGAACTGACGTCACTGTCATGGCCGCCTATGAAGCCCGACGACCGGAAGTACCTCCGCATCGACAAGGAGTTCTCCGTACACAACAATCTTGCTGAAGACAGAGTCAAAGTTTGGGAGGAACTCTATCCTATTGAGTATTGATTTTATGTGCCTCTCAACTGTACTGAGGATAGCTAGTAGTTTTTGTAAAAATATCTTAGATATTTTTTTATGTTTCTATATACTGAGCTTTCTATGAATAAAT

>SinsCEX4

CCACATTTCATGCTTTTAAGATGGCCGTCCGCGTCCGTTCTCTTTCGTCTAACAATAAAATAAGAGAACTAGTTACGTTGAGGTTATGTATTGGTCGATGCGAGTTACGTGAGTTACGATAGCATTAGCATTAAATAGTACAATACGCTCATTCAATACGCATTGCATTACGGTCTGATTGGCTGTATTTTATACAAATTTAAATATGTCGAGTCCCATAGTTGATCTAAATGAAGGTAAATTAAAAGGAAAGGTGTGCAAAACCTTAAATAAGATACAATATTACAGTTTCAAAGGTGTTCCATACGCAAAACCTCCGTTGGGTGAATTACGGTTTTCGGTACCTGTTCCATCCGAACCATGGAAAGGAATCAGAGATGCTACGAAAAATTGCAATATTTGTGCCCAATTCGACCGAGACACCAATGGCATAATTGGAGATGAAGATTGTTTATATTTAAACGTCTATACGCCAAAGTTATCAACAACCAATTCCACGCTTTTACCTGTTATGGTTTACATTCACGGAGGCGGTTTTGTCTTCGGAAGCGGCACCGATGACGTTATTCACGGACCAGATTATTTAGTGGAAAAAGATGTAGTTTTGGTTAGCCTTAACTATCGACTTGGAGTTCTTGGTTTTTTATCATTAGACTGTAAAGAGGCTCCAGGCAATGTGGGTTTAAGGGATCAAGTCGAAGCTCTGAGATGGGTTCAGAAGAACATTAACAAGTTTGGAGGTGATCCTAAAAATGTAACTATTTTTGGAGCAAGTGCCGGCGCCGCATCTGTAGAATATCTCCTTTTGTCGCCTATAGCTAAAGGACTCTTCCATAAAGCACTTGCTCAATCAGGATCCTCATTGTTGCATTGGGCTCAAACGAATAAAGCAAAAGAATTTGCTACGAAAATAGCGAGTTTGAAAGGAAAAGTTTTGCAAGACAATCAACAGTTACTGACATTTTTGAAATCGTTGCCTATAAAAGATTTAATTGCGACATCAATGTTAGTTTTAGCTGCTGATACAGGGCGAGGGGGCATAAATTTCGGTTTTGTACCTACAATAGAAAATCCAGTCGATTGGGAACCATTTATAGATAAATCTCCCTATGAATTACTGTCTCGAGGGGAATTTACAAAAGTACCGTTTGTGAGCGGTTTCTGTACTCGTGAAGGTCTCTTAATGGTAGCTCGAGGAGAAAATGTTCTAAATAAACTAGTCAAAGAAAAAGATTTTATAGAATATTTACCATTTGAAATAGATAGCTCACAGCATACAGAATTAGAAAATAAAATAAAACATATATATTTGGATGGGAAGAACAAGTATCAAGATGAGGATGCTTTCGCGATAGACTTCTTCACAGACGTCGATTTTCTTGGTGGTGTGTACGTTGCTACTACGCTTATAGCCAAACACAATTCACCCGTTTATTTTTATGAATTTTGTTATGATGGCAATTTAAATTATTTAAAGAACAAGTTGAATATTAATCGTCAAGGTGCTTGTCATGGAGACGAAAATGGATATTTAATAAAATCTGATAAGTATACAAAAGGACAGCCTAGCGAAACGGATAAGCTAGTAAGAGAACGAATGGCTGAAATGTGGACAAATTTTGCTAGACATGGGGATCCTACTCCAAAGACGGATAGTAAGGTGATTACCACAAAATGGGAGCCACTCGCTGAAACAGGAATGACCTGTCTCCTTATTGACGACAAATTGACAATGCAAGACAACGTGTATCCAGCCAGAATGAAACTATTTGAAAGTCTTTATAAAAATGCTTATGCCTCTGAATAATAGTTCGTATAAAATAGTAATATAAGTGTAATACGTGCAACCGATATTTTTTTAGATTTTGTCGTATTATGGGCATATCACAATCAAGGAAAAGTTTGTTTATTCACTCAGAAAAAAATATTCCTTTGCTTAAAACAACCTAAGATATATCATTTTTTTAATACAATTGGAGACAAACGGGCTTATAATAATAATCAACTTGACCCATATACATTTGCAACACCAGGTGTGATGCCAATGCATTGCCAGCCATACAAGACTCTTCTTTTGAAGAATGCCATGTACGCAACGCACGCAACGGAAAAGCCTCGTAAAGTGAGGTTGTTCCACAATTTGCGAGTTCCCACAAGAAAGTAATCTATAAATTTAATGCAAACACTAATCAAGATAAATGATTCTTATGTATTCTAGGTCTACACCTGGGATGACCAAAGTGCCCACGTCCCTTGTAAAGTAGGGGTGTATGGCCCTCGGAAATATATTTTGAACGCCTCATCGTCACAAATTGGTGTAAAACAGATTTTTACGTATATAAAACTGCTAGTGTAGCCTACCATCGCGCAGGGCCTTCAAACAAAAGTTAAATTAGTTTTTTTTATAAGTAAGTTTCTAACTTGAACATTAATAATTAAAATATTTCAAAACAAAATACATTTGGCAGGCTTATTGGCTCAGTTGGTAGTAACCCTGTCAGCTG

>SinsCEX5

AACAAATATTTGTCCCGTGTGGGAGTCGAACCCACGACCTGTGTGTCAATAAGCAGCTGCGCCACCACTGCGCCAAAAGGCTGTCAATTCCCAATCACTTATTTTTCGTTTATTCAAATTATTAATGATTTCTCTATTGCAGTACGGACTCCTAATAAATGTTTACGAGGTCAAAGTAAATTGTTCGAGTCATTATCATATTAACCCAGTCAATGGAGCTCAATGTTAATCAAAAATGGACGTTTAGGTCACTACACACTTTTATTTTATGAAAAAATTACATTAAAATGCACCAATTAAGAATGTGAGAACCCACAGTAAATAACAATGGTGCATCATTCTTTTTCGTTTAATAATTTACGTAAAAATACTACTTTTTTTCACATTTTGCCGTATAAGCTTATAGATAACTCCTCTCCTGTGCATTTTTTGCTATATTATATATGCTATATCATGTTTTTATTTCGTATTAAACTCTTTAAAAAATGAGACCCGTTTAAAGTACTTTGTTTACTTGCAAATAAAGTTTAAAGTTAAAAAAGTGAAGTGCTTTTAACTTTCAACATTAAATAACTCGGGAAAATAAAACAAAAACTTAAAAACCGATCTAGCTGAAATACACACTCTTTGATTTCTTACTAGAGCAACAACTTTTGTATTTTCGGAATGTAGGTGTGGAATTTCTAAATATCTTCTAGACTGACGCGCTTACCTTATACGTGTCCATAAACACGTGTTCAATACACTATTCTTACATAATCGTTGTTTGTTGTCGATTAAAATAATCATATCATTCGCCATGGTTACGTATACGATAACAACTTATCATCTATTTGAAAATATAAGTACATAGTACATGCAAAGTACGTACATATTATGTTTATCAAAATATGTGTATTATCATTTTTTGTAATCGCGCAGTCAATATGTAAGTGTTACAGTTGTAACAGCTGTTTCTTTCAAAAGAAAATTTAAAAAAAAATGAAGTATGTAAAGAAATTGGTGCTTTTCACTTTGTTTGCTATAAACTTAGTAGAGCAACCAGCTCCGGAAGTAATTATCGAGCAAGGGATTTTGAGCGGTAAAATAAGTGAAGATGGCTCGTTTTTTGAATACGTTGGAATACCATACGCTAGTACGAACATCACTACGAGATTTCAGCCTCCCCTACCTCCTCCAAAATGGGACGGAGTCTACAAAGCGACGCAAGAGTATTTTTGTGCACAAGCATTACCATTTACTGTCTTGGGATCCGAGGATTGTTTAAAAGTGAACGTCTATGTACCAGTTATGGCTAAAAGACCATTAGCTGTGATGGTATACATCCATGGAGGAGCATTTATACTCGGGAGTGGTGGAAAATTGCTGTACGGACCAGAATTCTTGGTCAAACAAGACGTTATATTAGTAACTATTAATTACAGATTGGGTACATTAGGTTTCCTATGTTTAGGGATAAAAGAAGCGCCTGGTAATATAGGTCTTAGAGATCAAATAGCTGCTCTAAAATGGGTCAAGAAAAATATAGCAGCTTTCGGTGGAGACCCTGACAATGTAACAATTTTTGGTGAAAGCGCTGGAGGAACGTCTGTGGGCTTATTGTTAGCAAGTAATGCAGCTAATGGCTTGTATAATAAGGCCATAGTGCAAAGTGGATCGTCTTTAACAGTTTGGGCGATAAATAGGGAACCGTTATTAACAGCCAAATTAATAGTTCAAGCACTAGGTTACGAATCCGCAGATCCTTATGAAATTTATCAAATACTTTCAAAGGTGCCTTATAAAGAATTGGTCTCAGCTAAAGTAGTAAAAATTCTAGATTTATTCTTCGATCCAAAATTGCTACATTTGCCGTGCGTTGAAAAACCTTTACCTGGAATAGAACCAGTGCTACCTGACTTGCCATACAATATTATGAAAAATAAGAAAATAGATGTACCACTTATGTATGGTTCGAACAGTAAAGAAGGCTTCTTTTTAATATCAAGTGAAACTTCACCAGAGACTCTTGAAGACAGAAATGACAAATACTTATTTGGATCAGATTTAGAATTCAAATCTAAAGAAGAAGCTGAAATAGTAGCGAAGAAAGTTAAAGAATTTTATTTTGGAGATAAAAGAATAAGTATGGAGACGATTTTGAACATGACCGATCTTTATACGCATTTGTTCTTCGAAATACCACTTATATTGGAGTCTGAAATTTACATAAATAATAATAATACTTCAGTTTATCATTATCATTTCGATTACCATGGTGGAAGGAATTTTCTTAAAGAACAATCAGGATTTGTTAAAGAACCTGGCGCATGTCATGCTGATGACATTTTGTATTTGTTCCAAGGACGAATATGGCCTTATAGAGTGAATAAAGAAGATCAAGTAATCATAGATTGGATGACGAAAATGTTTTCCAATTTTGCTAAATACGGAGATCCCACGCCGCCCTCATCAAATGATCTGCCAGTCAGATGGGAGCCCAGTACTAAAGAAGAATTAAAGTTCTTGTATATAGATAAGGAAATGAAAATGGGGCCTATGCCATGTCCTGCCGCGTATAGTCTATGGAAAGACCTGTATGAGAAATATCGTAGAAAAAAGTTATGATTTTAATCTGTACGTATTTACTAACATATAAATTTTGTAAATATTGTGATGGCTAAATTAAATTCCTTCATTTTTTATATTAACTTTTTATTAGTTGTGATAAACATATAATTGGTACATAGTAAAAGTTCATTCTTGTAAACCATATTACCGAAACCTGGACATTATCTATACTAGTATATGCTAATATAAAAAAACCGGTCAAGTGCGACTT

>SinsCEX6

GTGCAAGGGTGTAAATAACTTGAGATCCTTATAAATATAAGGATCTCAAGTTATTTTACATTTTTGCACATATCAATGCAGTCTGATCCAGGTAAATATTGGAATAGAGGTAGTCAAAACGTGGACGTTTTCTTCATAACTTTTCAACAGATTTTGACAAATTGACAGTCGATGTAATAACGAAAAAATAATACATGGCAAACACTAAACTGAAGAAATATTAATCTCACACATTATTAATTTCTTATAATTATGGTGAACATAATGTTTTTGCCTTTGATTAAATTGTCTCGAGAAGTTAGTGGAAAAAATCATTTTCGTGTTCACTCTTGTTAGTTTTTTCAACAACCACTATTCCAGTACCTGACTGTAGACTAAGACTTGTTGATTGACGCCAATATCGGTTGTAAAAACAGTACACATTTCTTCTTATGAAATATGTTTAAAGATAAACATCAAAATACATAGCTATCAAGTATACAAAAATTACTAGCAAACCTTTAGAAAACTCGTAAACCTTTGTATATCTGAGAGGAAGCAAGCGGTCCGATCAGCCCGATGTGTCGGTATGCGGGGTCCGTTAGTCCATTCGCATTTTGAATGTTGTAAGCACAGTCTACGATGCTTCTTTTATTTGCTTAATGATACCGTTTCTGAGATAACATTGATTTTCAGATTTATAGAAAGTAGATAGATAATACTGTCGGAATAAAATCGCCGTTCCCCAATTATTAATATTTTTTTATTTTAATATAACAAACTTATAAAAATATAACACTAAAGCTCTGTAATCATAATGGCAATAATATTAAATTGCAATTATTCATATGTTATAATATTCGTGTGTATAATTAATTTCATAACTCAGTCTCATTATGCCAGAATAATTTAAACAGCAGTGGTACAATTATTAACACTGTAGATGTAAAGGCAAAAAAGATTTTGAAACGTAAGCAATTGTTCGTTTTGTAATTTGTCTTTTACTTTTTTTTTTTCATTTTATAAGAAGCAAGGTAATGCTCCGTCAATATGTTCAGCATAATTTATTAAAAACTTAACGTATCGCGATATATAAATAATAACTTTGTGAAATATGTTTAGAGACAAAAATCTTGTGACGATCATTTCTTTTGTGCATATGTAGTCTCTAATTCGCTACATATGCTAACAAAATACGAGGGACTGATCTCGTCATTCGTCAGTGACATCGAAGATACAGACATAACGCAGAATACAAACCCACTTATTAGGAGTACAATGATGGTGAATAATTGTTTGTCCATAATATGTAATAATTGGATAAATCAATTGAACGCTAAGGTTTATTACTTAATATTTTAACTAAATATTGTAACAGTTTTCTAATATTACATGACAGTAGTCGATACTATTCGATTTTTACTGTGCTTGTTTAATTTTTTTGGTGAAACGTACCACTGCTGTTTGGTACTGTTTACCCTGTACTATCATCGGTTATGATGAAATATTAATTAGTTGATGTTATGGCTATTCATATATAAAATAAATGAAAATATAACACAAATAACTCTTTGGATAGGGTTACAAAATATTTACTAGGATGTGAAATTATGGATTTTTTTTGGCAAGGATTTCATTTATTCCTTTTGATTTTGTCATTATATAATTACATGATAACCAGCATAAAAATGGAGATTATTGTAAAAATGTTTAATATGGTGAACCCTGTCCCAAGATTTGTTTGTGTATCCATATAATATCTAAAATTATACCAACGCTTATATAATTGCCTTGGTACAAAAATGCACCACAGTGGAATGTAAAATAAGCAATATACAATAACATTTGAATAAAAGTTTTACTTTAAAATGTATTTTTTAATGATTTGGAAGACCAATTTGTTACAAAATGAAAACATAACATTTTTTAAAAACAATCATATCTTTAGTAAAATAAGGCCCCTAACAATCGACATGTATACATTAACGATTTCATTGGACATTATTATAGTTATACTCGAAAAATGTGATATAAAAATATCCATAAACACATAAAAACAATTTAGAACACAATCTTATATAACTATACTATATACACATTTAAAATATATAAATTCGACTCTCATTTATTGCATTTGTGACTATATTAGTGATAGTACCAAGTATTAATCATTTTCATTGCATTCATATAATAACGCGTATATCTTTTCACCAGGAAACTCCTAAATCATTGAAATAAACCAGCACTCTTAGAAACGTATGCTCTTTATGGAGCTCGTCCCAAAACGCCATTCTTTCATGGAATGGATTTCTGCCTTTGGTGAGTTCTGATCCTATGCTTAGATAATTCAACTTGTCTTTGTTCTCAACTGGATCCCACGTAATTTGTAAAAGGGGGTCATCATCATTAGGCGTTGGATTTCCAAATTTTGCAAAATTGGTCCACATTTTTACAACTCTGCTTCTTGTTTTCATAGAATCTCGACTACCCTTGCAGATTGGTGAATTAGGCATTTTTAAGCGAAAAATGAATGGAATTTCTGCACAATGTCCTAGAGAGTTGAAAAAAGTTAAACAATTTGGCACAGTCCATTCTCCTGCATAGTTTAAAACATAGTAATATATGGGGCATTCTGGTGCGAACTGACTATGTAGTCTGACAGTTTTGTTCACATAGTACAAGAAGTAGTAGTCACTGATTAGCTGTAGATATTGAGGTTTAGTATCTTCTCTTAAACATTCTTCACCACCTAAATAAAATTTTAAAAGCTGATGACCTATATTTTTTGACAACTTCGGTTCTCCAGCAAGCGATCTTGGCACTAAGAGGCAAACATTCTCATTAAACTTCTCAAAGTCATTGTAAAAATCTTTTTTTATAAATTGTAGTACTGAACCTTCAATATTGTTACTGCCAATTATTAGAGGTACTTTATTGAAATTACCCGATTTAATTATTAATGGTGGGCTTTTAGTAAGAAATGCAGATTGACCTTCAAATTCATCTTCTATGCATGGTTTAAAAGTGGAATCAAAAAGATCTGTTCCCTCCTTCTCAAAACCCCTTTGTTCTAGATCAAAGAAAACCTTCATTATCTTTAAGGCAGGAAGAGACCGCAATTCTTCAACTACTTCTACTTTACAGGTTTTTGTAATACCTAATTCTTTGGCTAACTCCATTGCTGCTTGAGATGGGTTTTTTGCTAAAGACCAGTTATTTAAAGCACTTGCACTTTGTATTATTGCTTTATGAAACAGCCCCGACGACATTGGTGATAACATCATAAAGTGCACTATAGCTCCACCTGTGCTATTTCCAAATATGGTGACATTATTTGGATCTCCTCCGAACACACTTATATTTCTTTGAACCCATTTCAATGCCATAACTATATCTTTCAATCCACAATTGCCAGATGCTGTAAAATCATTTATTGATAGGAATCCAAATGGTCCCAGTCGAAAGCCGCAGCGTACAAATATAACACCTTGGTCATTTATCAATAATGGGTCGAGTATTGGATCCATTGTGCTTGAAAAACCATAACAGCCTATCCAAAACATAACTGGCATAGGCTTGTCAGGTTTTATATTTGGAGTAGACAAATCAATGTAAAGGCAATCTTCAGATCCTTCTATATCATGGTCAAAAGTAAAATGCAAGGGTAAGGAAGCGTCTTTGGTGCAATCAATTTCTTTTGTCCATGGTTTTATAGGAGTAGGCGGTTGAAATCTTAAATATCCTAAAGGTGGCTGGGCATAGGGAATACCCTTAAATTTGTAATATATCTCTTCATCTACTTTATCAATGTATCCACATACTGGTCCATCCTTTGTTTCTACCACGAATTTTGATTTCTCTGGTATTGTAGCCATTTTCTAAAGTAATATTAAAAAAATTATTACTATTTATAAGTTAAAAAATGCTGTAATGATTATGAAATTTACATTTTTGCTTTTACTTTCCTTATAGATCGTTGTTTATTCTTTTATACTTTTTACTGAAAAAACGAAACTGTCAAAA

>SinsCEX7

TATATATTTCCATTCAGAATTTAATTGGCAGAAATAAATTTCGTGTTAAATATAAATATGACACTTCATTTGTTCATACTTCACTTTAGTAGCAATATTTATTTTAATAAACAAACTATAACTTTTCAATAGACTATATAAAAATAACATTACATTTCCCACGCGTTGCTTTGTTTAAGCGAGATGTTAAGACATGAGAACCAGTATTATATGTAAACTTACTAAACTACGTATAACTCTTCTTGTTACGATAGAATCGGTGCCAGAATCCTCTAAAGTTTCTTTGCAACATTTAGTACCGATTTCAACAACTTTGGGATAGAAATCCGTCCAAAACTCAAACGCTTTGTAATTTATAGTATCCGGTGTGCTCATTCTAAAATTTAGACGACTAATTATAAGATAACCAGGTTTCTCCTCTTCAAAAGGTTCCCATTCAATAATTACACCAGAGTTAGTAGGATGAGAATACCAAGCGAAATTGGTCCACATTTTCATCATTATCTCAGAAATACTTTTATCTCTGTAATCCCATTTACTAAAATCTTCAGGTAAAGCCAGATAAGGCATGCCGAAAGTGAAAATTAAATCAAAATTATGTGGCACCCCAATCCAATCTGATATACCTTCATTAGCTATGTCTGAAAACGGTTTTAGGTCGAATCTGTAAAGATAAACAGGCGCGTGCTTACTCAGATGTTTAGCTAACTGATAAGTGGTCGCTCCATGGACTTTATCAGTGTAAAAGTCTAGAAATTGTTTTCTTAGAATAGTTTCGTTGGTTGTGGGCGGTATCGGCTTATAGAAAAACGCTACGGCGTCTTGAATGTGATGTTGATTTGTAAAACAGGAACTGTTGTCTATTGTTATGTCGGAAAGTATTATCTCTTCGCGCATCAGATCGTATTCTCGCTGGCTAATTCCTTCCTTTCCATCTTCTTTGTCAAGTAAGAGAGCGTCTTCCATATTAGTATACCCCGTAAGAACTGGTACTGGAGTAAAAATTTCATCTTTAAACAATTTACTCGGAAGATCTGGTAAGAAAGCACCGGATACGTTACTGAAGCCGCTGTCTACAATGGGTCTCCATTCGGCTAACGGCCCTCCAATATCTAAAAGAGCTTGATAATTAACTCTTCTGAGACAGTCTAATAATTGAAAAGTGGGCTTTCGGAAACAACTGAATGCACTGGCTACTTTATCGACAGTTATGATTTCCTTTCTAGCTATATTTACAGTGCCTGGTGAGAGGACGTTGCCACTCATAGCTATAGCTTTTTGGAAGAGTCCAGAGGAGTAAGACGATATGAGATGAATTCCAACGCTTACTGCGCCTGCGTCGTGGCCAAAGATACACACGTTATCGGGATCACCTCCGAAACTTTCAATATTGTTTTTTACCCAATTCAGAGCTGCTACCTGGTCTAATAGGCCGAAATTACCCTGAGCTTCGGTGTCGAGCGTCGAAAAGAAACCGAATATGTTTAAACGGTAGGCGACGGAGACAAAAATTATCTTTTGTTTTAAGACTAGCTGAAACGGATTTAAATCATTGGGACTGCCTCGAACAAAATCTCCACCATGAAACCATACCATAGTTGGAAATCCTTCAATTCTCGGATCGTCTGGTACATAAACGTTGAGATAAAGACAGTCCTCCGAATATTCTTTCTTTCGCGGCTCCTCCAATTGCGATTCTAATAATTTCGTAAATAACTCATCGTGTTTGCTCAGAGGATTCTGTACATCTTCTTTCTTGGGGTCACTTTGCATACAATCTGGGGCATATATTGTCGCATTTCTCGTACCTTCCCATTGGGGCAAGTCCGTATATTCAGGTGGCGCGAATCTCCTCAAATCAATTGGAGGCTGTGCGTAAGGTATGCCAACGTACGCCGCTATGCGCTTTGTTCGAAACCTCGTTATGTACATACCGGCAATTGTCCCTTGATTCGCCAAAGTTATCTGTTTTGGTTCTTTAGCCGACAGAACCAACGATGTTAACATTGATAACACAATAACACGAATCATTATCGTGGCTTTTTAATTTTTCAAATTAACTAGCAACACTAAATGATCGTTTAACTGAATAGAAATTCCTAACAGCCCAAAGAAAGAAGATTCCAGAAAGGAGATTTTAAAAATCGAACAGATGCGCAATTCAATTAGCTTTTTCCTTTTTGCAAATCGAACGTCTGTATTGGATTTGAATTTTAAATCGGTTTTAATTTTTTTTTCTTCAATCGCGTATTTGTGTTATAGCCAGTGTCGGCCGGCAGTGGGCGGTCGAGTTCGACGGTGGTGCGTTACTGTTTCTCGTGCAGACTTCGGCCCGTAAACAGTGGGGTTGTACGTCGTTATAGCGACCAAGGAATTGCTATTGTGTATTTAGAGTGGCTCAGAAATGACTTACTAAAACAATAAAGACAGTTAAAAAAAATATTAATTGATTCAACATAGAATTTACACTTCATTTTTGTTTAAAAAATTATAACTTGATTGACATTTGATTTTGCTCGCATATATGTAAG

>SinsCEX8

GGCGATGCATTTTCTACCCATTCCATGCCCATCAGGCTAGCGGAGAGCACGACTTCTTGGTTTATTAAACCTTTAAAAACAACTTAATAAACAAGTTCATACAATGGTCCAGGTAACAGTGAGTGAAGGTATCCTGGAAGGAGAATTAAATAAAAACTATTTAGGAGGTGAATACTATAGTTTCAAAGGAATTCCTTATGCTGAACCTCCTATTGGAGACTTAAGATTCAAGGCGCCACAACCAAAGAATGCTTGGAATGGAGTACGTAGCGCAAAGGAATTCGGCCCTATCTGCTACCAATACGACTTGTTTTTTCAAACTCTGCCTAGTAACGGAAGTGAGGACTGCTTATACCTAAACGTTTACACTCCAGACCTCAAACCATCGAAGCTTTTACCGGTAATGTTTTGGATCCACGGCGGCGGTTTCACTTGTGGCAATGGTAATGACGACTTTTATGGTCCAGAGTTTCTCGTCAGAAATGGTGTAATTCTTGTCACAATTAATTATAGAGTCGAAGTTCTCGGATTCCTATCTCTAGAGACGGAAGACATCCCGGGCAATGCTGGCATGAAAGATCAGGTAGCAGCACTTAGATGGATAAAAAATAACATACGAAACTTCGGCGGTGACTCAGAAAACATAACCATTTTCGGCGAAAGTGCTGGAGCCAGCAGTGTTTCGTTTCATTTAATGTCTTCCATGTCAAAAGGTTTATTTAAAAGAGCTATAGCTCAGAGCGGTTCTTCATCTTGCTGGTGGTCACTAGCTATAGAACCACGCGAGAGGGCGCTAGCTTTAGCCAGAAAATTAGGATGTCATTCGGAAGATGACAAAGAACTGTATGAATTTTTCAAATCTCAACCTGTTGAAACCTTGGTTAACATTCAAGTTCCAATAACATTCGTTGAAAAAGCTAAAATTGCGGTCGATCTTAAATTCGGCATTGTGAACGAAAAGAAATTTGGAGATAATGAAAGATTTTTCTATGGCGATCCCTATGACGTTATTCGAAATGGTATACATGAAGGTGTTGAGATCATTAAAGGGTACACTGAGCACGAAGGAATTCTAAACATTGGAATCTCTAAAGATATTGATGCGATGATTCTACAAATTAATGATTTCCGTGAATTCTTAGTACCAAAGCCGATAGCACTTAATTCTCCGATAAATGAACAGTTTGAAGTAGGTAGAAAGTTCAAAGAATTCTATTTCAAAGGTAAAAGTGTTTCCAAAGAAAACTTAATGAACTTAACCAATTTTTTTTCCATGGAAATGTTCGTTTATGGAATAGTACAGTGGCTGAAGATCATTGCCAGTCAAAATAAAAATAAGGTTTATTTGTACAAATTTACGTGCAAATCTGAAAGAAACGTTTTTACCCAAGTTTGGAAAGCAAGTTCAATTGCTGGAAATCAACCTACGGTGTGCCATGCTGATGATTTACCATACATTTTCCCAATCAAACTTTTGGGTGAAGTGGATCAAAATTCCGATTCGTTTAAAATGATCGACAATGTGACGAAACTTTGGACAAATTTTGCAAAATATGGGAACCCTACTCCTGACAACAGCCTAGGCGTAAATTGGATCCCATTTTCTCAGGATGCACAAGAATATCTGGACATTGGCCAGCAGTTAGTGGCTGGATTAGCAGCTGACAAAGAAGAAATTGAGTTTTGGGAAGACATCTTTAGAGAATATTGCCCCCATTTCGTAGCTAAAAAGTCTTCTTAAGAATTTATCGAATAGTTCTTTCTTGTTAATTTTATTACACCTATAGTATTTTCCATTTAGAACAGAATGTCCTACACTTAATAATATAGCGACTCAACTTAAAATTTATGATTTTCTACGAAAGCTCAAAAATCGGCCAGGAAATTAAGACAGGCATTTTATACGCAAATATCATAGTTATTATTGGGTCATGAGACAATAACGGGCTCTAGCCTTTCATTGTTTTTATGTTAGAGTATTTGTATATTGTACATAGATGAAGTCATAAGAAAATTTTGTCTACATAAATAACAGATCCTCACCACGCGGGCCCACTGTGATGCGGCGGAGATTTAGAAGGCAACATCACTATCAGAATTTATCTAGCCCCCTATGGTAACCATAGGTATGT

>SinsCEX9

GCTCGGTCGCCCAGGTACTCAAGTTTTTAACTACATACTACTTTACGTGTTTAGTTAATATATGTATAATTGTAAGAATAAATAGAATCGTAAAGATAAATGTTATCTACTGACTTATTAAGTACCTAAATAAAATATAAAAGCTCACCCATGTAAGTCGGTATACATGGGTGGATGAGCTTTTATATTATACAGGTATGTATAATGAGTTAAGAAATAGTAAAAAAAATGTTATACGAATGTAAATAAACAAGCATTATTTAGGCAATTTATCAAAAAATATGTTTATTATGCTAATTCATACACACGTATGCTAATTCACAATAGACATAAGCTGCGTCTACCTATTTCTACGTATAAATACTTTTGTGAATAACATACTTGAACGATAAACTGATTCGAGCAGACACGTTATTTATCTTTACCTACATTTTACTTTTAGTTCAATTAATAATTTATAATGGAATCACACGCGTTGTTAGCTTTGATAATTTTTTGTTTATGTTTATTTTGTAAATTATGTGCCTCGCAAGATTCCACTGAAATTGTTCTTCCGCAAGGGAAATTAGAGGGTGTATATAACTTGTACGGAGACTACAAACAATATCTTGGAATACCTTACGCTTCGATTAAAGAAAGATTTCAAGAACCGGGGGATCCCCCATCGTGGAATGGCACGCTAGACGCAAGACGTAGATTTATCAAATGTAAACAAATGTTCTCCCCTTTAAAGATAGCAGTCGGTGAAGAAGATTGTCTCGTCTTAAATGTGTATACACCAACACTTAAAATGCCAAAACAATTACCTGTAATGGTGTTCATACATGGAGGCGGTTTCTATTGGGGCAGCGATACTGATCTCATTTATAATCCAAAGTATTTAATAGAGAAAAAAGTTATAGTAGTCACTGTAAACTACCGCCTAGGAGCATTTGGCTTCCTATGTTTAAGACAAAAAGAAGCACCGGGTAATGTTGGTCTAAAGGACCAATTAGCAGCACTTAAATGGGTCAAGAACAATATTCGTCATTTCGGTGGTAATTCTAATGCGATTACATTATTTGGCCAAAGTGTTGGTGCCGCTGCGGTCAACTATTTAATGTTATCCCAATCAGGTAAAGGGTTGTTCCATAGAGTTATTTTGGAAAGCGGCTCCGTGTTAATGCCGTTTGCGTTTGACGATGACCCCATAAAAAGTGCTGCTATAGTGGCTTCGAAACTCGGTTATAACACAACGGATCCTAAAAAGTTATTAGAAATATTCAAAAGTACTGCAGCAGATGACATCGTTCGAGCTAGTTATGTGGATCCATTTAATAATGCTTTAGCGCCTTATATTTTTAAACCTTGCTTAGAAAATCCAGACGTCAGTAAGGAAACTATAATAAGAGCACACCCAAACAGATTGCTTGAAACAATGAAATTGGATAATGATATTAGTATGATTATAGGGTTTAATAACAAAGAAGGTTTGAGATATGCTGCTCATTATAGCTTCGAAGGTATAGAAAATCTTAATAATGACTTTTCTAAAGCTCTACCTAGTAATATATTTTTTAGAAGCAAAGAAGATAAAACAGACTTCATAAAAGAAATTAAAAGTTTCTACTTCAATAACAAACAAATAACATCAAATGGTTTAATCGATTACTTTTCTGATGCACTAATCGTTTATCCAACAATAGCCACAGTCCAATATCTTCTAAAAAACACAAATGTTTCTGTGTACAATTATTACTTTAAGTACGATTCATTGAGAAATTTGAATAAATTAATTTCTGGATTACCGTTAACGCCAGGAGCGAATCATGGAGACGAATTATTTTATTTGTTCAATCCAATCATATACCAACCATTACCAATGAGTCCAAATGATTATACTATGGTAAATAAAATGACAAATTTGTGGACAACTTTTGCTAAAACTGGTAAACCAACATTATCGTCAGCAAAATATGCATGGAAGAGAAGCGTAGACAAACAATTGAATTTTCTAGAACTAGACAAAAATGTGAAAATGGGAAAAATGGCCAATGTAAAGCGCGTGAAGTTTTGGACGAAAATGTATGAAAAGTACAGCTTATAGGACGATTGAATAAATTTAAAAGAATCACTTTATATTAGGTATTAATTTGAAATGGAATCTGACAGTACGACTGTCGATTTGTCCACATGTCTAAAATCGACACAATCGGCAATAGGTATATAAGATATAATTATATACCTACCTAAGATATCTAATATACTGACTCTTCTAATATACAAGTATGCCGTATGGCCTGGTTTAGAAGTCAGAATTCATATTTATGTAATTTATTATGAAAAATAAAATGTTTATTATTATCTTTCTCTTTACATAATATAATAAAAT

>SinsCEX10

TGTTACAAAAGCTTAGTCTTTTTTAAATTAATGTTAGCGCTATCTATTGAGTCAATGAAACCAAAACGCTTAGTGTTAACACTGGATTTTAGTACTCATCAAATTTGCGTACCGGGAGCTTTAAATGAAAATCGATTTTTAAGGAGTAAACTCCAAAAATGTTTTTGTTAACCCTTCCGCTGCTCCAAAACTGCCCCTCAAAATAGAAATTAAGCTTTTTTTACTTCATTTATGTAATTGAACCATTAATTGCTATAACTGTGCCAAGCTTTAACTTCCTATGTCTAACAGTCTCTAAGAAAAACCTGTGTGACAGACGGATGGACAAACACCGAGATGATACCATAAGTGTTCCGTTTTACCAATTTTGGTACGGAACACTAAAAATGTATTATTCACGTGTTATATAGTGTCAGCATACTTATTTTTTTTTTGTTTTATCAATATTGTGAATTTCTCAAAAACAACAAGACCTAATTTAAGATCATTGCTTTAGTTTTCAAGTATAATTTAGACAGAGTTCTTTAAGAGTAGTTTCAGAGATAAGAGAGAGAAAATGTTATTGACGCATATTTTCAACAATATTTCTGAAAGTAAACTTCGACTTCCGACAACATTTTAAAAAATATACTACTAGTCTTTTGAAACGATTTTAAATATGGAACACAAATAAGATACAAAAATTTTCAATCTTTAAACTAACCCTGTTAAAAATATTTTTTTAAATACAAACTATGAGAAAACATTGTTTGGATTATTATGATTCATTACTATACATTTCACTCTCTACTTCAAGCCTTCCCTGAGAAAAATGGCTGTGAAAGACTTACGGACATAAGGGTGATCTTATAAGGGTCCGTTTTACCTTTTACGTAAGGAACTTCTTAAGAAACTAATACTAATTTAGTTTTCAATTAGGCAACTTTCAAAATTTTTCTATTCTTATTGAAAATATTAACTTTGATCTTCTTTCTTCTATTATCTAAATTCTTTGGTCACATCACATAGTACTTTTTAAAGGACATTCACTAATGTTTTCTCCGATACTTCGTATAAAGTTCGTGCCAATAGCGCAAAGTCTCACTACCCCACAACGGGGCAGCACTAAACTCTTCATCTATCACGAAGGCCTGAGGCAACGAACTGTTCGTAGGTAACCACTTCTGCGGTAACAACTCCGTAACCACCGGTGTGGGATCACCAATCTTGGCGAAATTGGTCCACAGTGTTGTCATCCTATTAATCATTTTAGTTTCAAACAACGTTGGTACAGGTAAAGGGTTAAAAAGATAAAACAGGTCGTCAGCATGCGTCGCGCCTGGCGCGTCTTTAAACGGTTTGCCCGAAATTATTTTGGGTAAATTCCTCCGACCGCTGTAACTCAAAACATAATTGTACACTGGTTTATCATTATTCTGTAGCACCAGTTCAGTCTCAGCCAAAGCAGGATAAGCAAAATATGGCTCTCCATGTAGCTTTGACAATTTCAACAAAGTCTCTTTTGAAATTTCTTCATCTCCCATTATCAGTTTTTTTAGTCTCTCAGCCGCTTCCTTCTTCTCTCTCTCCGTTGGAAACGCTAAATCTTTAGGAATTGATTTTTCAAACTCTATCTTTGGTAACGTTGTATCGTTTTCTAAACGTGCAAAAAAGAAACCCTCCTCGCTATTCGTACCAATAATCATTGGAACCTTATTGTAGTTCCCTTTAGCTAATATATCATAAGGTATTTCAGTCAAAAATGGTTCTATACCTTCTATAACTCGTTCAGTGCAAGGAACATATAGCAATTCTGATATTATAACATTACCTTCTTTCCTAGGCACTCTACTGACGATTAATTCCGCGTCAGATTTGTTCATAAAGAAATTGTAAAGTTCGTAAGGATCTTGTGATTTATATAGCATAACTTTTGCAAGTAAACTAGCCATATAAACGGGGTTGAACTGATATGCCCATGCAGCTAAAGAAGAGCCGCTTTGTAGTATTGCTTTGTGAAACAGCCCTTTTGACATAGGCGAGAGGATGTGGTATGAAACAGATGCTGCACCAGCGCTTTCACCAAATATAGTAACATTGTCAGGGTCTCCACCGAACGCTCTGATGTTTCTCTGAACCCATCTTAAGGCAGCAACCTGGTCTTTCATACCAGCATTTCCAGGTGCTTCCTTTATACCCAAACATACAAAACCTTGTATGTTAAGTCTATAATTTATGGTTACGAGTACAATTCCCTTATTTACAAGATACTCAGGTCCATATAGGAAGGGCGTACCGGATCCATCATAGAATCCTCCACCGTGAATGAACACCATGACTGGCACAGGTGCTTCTGGAGTGGCATCCAACGGTGTGAACACGTTTAAAGTGAGACAGTGCTCTTCTCCGATAACTATTGAAGAACCAATTCGTTGATGACATTTTACGTTTTCGTTGATCGCTTCGAAAACGCCATCCCATCTTGGTTCTGGACCAGGAGGCTGGAACCTGCCAATGAAGGTAGCATATGGTAGGGCAAGATATCGCGCATGCGAACCATCGCGTGCCAGTTCGCCGCGCACGAGTCCGCTGGAGATGCGCATCGGCGGCGTCGGATCCCGCGCCAGCCGCGCCGCCCACAGCGACACCAGCACCGTCCAATGCAGACGAGACCCCATCGACAAAAATCACAGTTCTCGATAGATAAAATCAGTTTCAATTTATCTAATCAGTTGGACATTTTTTAGAATCTCAAACGTTAATCACTCATTTGTACCTTTTTAACCTGGATTTTTTACACTTTTAATTAATTATTATCAATTTGGCAAAGCAGTATTTATCAAATAATACACATACATTTGCATTTTTATATATAAGGTAACACTATTAAACACTGTGCACGAGCACGACACAATTACCTATCATTACAGCTGATTGATTGTGAGGACACACTATAACAAAATAGTTGGAAAACGGTTACAACTATAACTCGGAATTTTATGGAAAATATCGCTTGTTGGACACACGTGTGTTACGAAGCAGGCTTGTG

>SinsCEX11

AACAGACACAGACAGCAGTAACGCGCGATCGACCAGCGCGTGCGCATGCGTCCGTTTATTTTATTTAATTGTAACTATAAAAATAGTTTTTAAAATGACAATTTTAAGGTTATTTTTATTTTTCATTGTTGCTGCGTTAACCACAGCGCAGGGCTCAAATCCCATAGTTAGGGTAGCACATGGAATTATCCAAGGATCTTGGAAAATATCGACCAAAGGTAGAAATTATGCTAGCTTTGAAGGGATCCCATATGCAAGACCTCCAGTTGGAAAATATAGGTTTAGGGAGCCACAACAGCTAAAATCCTGGACTGGAGTTTGGGATGCCACGAAAATACTTTCACCATGCATCCAATATGATCCTTTCCAAGGGCAAATGAGGGGCCAAGAAAACTGCTTGTTCATCAACGTCTACACACCGAAACTGAACGCTGGCGCATCCCTTCCAGTGGTCGTGTTCATCCACGGAGGGGCATTTATGTATGGCTCCGGTGGAGACTATGATCCAGCCAATGTTTTGGATTGGGATGTAGTTGCTGTCACTTTTAATTATAGATTGGGACCTTTAGGATTCCTGAGTACTGGCGACGAAGCAGCCCCAGGCAACGCTGGACTGAAAGACCAGGCCTTCGCCCTCAAATGGATACAAAACAACATCGCAATGTTTGGTGGCAACCCTGACAGCGTGACGCTAACCGGGTGCTCGGCAGGCGGCGCTAGTGTGCACTATCATTATCTCTCCCCATTATCTAAAGGAACATTCGCCAGAGGTATAGCTTACAGCGGATCTGCGTTCACGTCATGGACACACGCAGTTAAACCAGCACAGAAGGCTAGACACCTAGCCGCCATTGTTGGCTGCTCCACAGCAAACACCCGGGAAATGGTCGACTGCCTCAGATACAGACCTGCTGAAGTACTCATTAATGCACAAATTGACATGTTTGAATGGAAGATACACCTGTTCACGCCGTTTGTACCAACAGTTGAAGCTCCAGGAGTAAGGGATCCCTTCCTATCGCAGTATCCTTACCACTTAGCCCAGGCCGGAGCTATGCACAAAGTACCTCTTATCACGTCAGTTTGTTCTGAAGAAGGACTTTATCCGGCTGCTGCTTATCAAGAATCACCGGACATATTACCAGAATTAGAAGCACAATGGGATCGACTGGCCAGCAATATCTTTGAATACAATGACACACTACCTCTCAAACTTAGATCAAGTGTGGCAGCAAAAATCAAACAGCAGTATCTTGGTGGAAAACCCGTTTCACAAGAAACTTACAGTCAATTAGTTGAGGCACTGGGTGATCGTTTATTCGTGGTCGATGTAGGCAAGCTGGCCCAGATCCACGCTGCCAAGTCGGGCCAACCAGTGTATTTATACCGCTACTCCTTCCGAGGGAAGTTCAGTCTCTCTCAAATAATGGCCCGTAATGATAAGGACTATGGAGTGAGCCACGCTGATGACCTATTCCATATCTTCAAGTTCCCCGGTTTGAAAACGTCGCCTGAAGATGACAAAATGAGCGAAGCACTGATTAACATGATCTACAGTTATTCCACTACCGGGGTTCCAAAATTAACAAACGACGGCCCAGAATGGCTTCAAGTGAAACCTGGATCTCAAGAATTGAACTATTTAGAAATATCATCGCCGTCCAACTTTCAGATGAAATCCAGCTCTGATTTCGGTCAAAGATCTTTTTGGGACAGTCTTGGATTTATTGAGAATGAAAACTATCAGACTCATATCAGAGACGAACTTTAAATAATATTTAAAGCTTGGTCCATAATATCATCAAATAATTCGTGCTCATTTAGTTACTTTCATTTTGAAGATTCTCGAAAAATAAAGTAATATAAAATACTTTTGCCCACGATTTCGCCCGGCGTGCCTGTCACATCCCAACCAGTTTAGAAAGAATACCATGAGTGCGTATACAAAAAACATATCAAGTTGTATCTCCGGTTCTACCCAATCGATTTTATTCAAACTTATAACTTATACAGTATTTAAATTTGATACACACATGGAATCAGACTTTCCCATGCAAACTTCAACACCCCTTGCGGAGTGCGGTGGGTCCTTAGAGATTGTCTTGAAAATCCTAAAATACACCAATTTCAAGTTTCAATGTCATACACGCAGGAATTACGATTTTTTTCTATAGAAACTTTAATACCCCATTTCACTTCCTTAAAGGTTGATTTTGGTAAAATCCTTTCTCAGCGCACCTAAGGAACATATTATTTTTTTAAATTTCACGTCTCTACATTCAGTAGTTTAGGTAATCTGTCACCCAACTACGCGTAGTCTGTCACCCAATCACTCGCGGAACCGGATATATTTTATGTATAGCCGTTGCTCGCGACTTCGCCCGCGTGAACGCTGCATTTTTTTATCTCAATGTCCTCAGAAGTTGAATTTAGAAAAATCCTTTCTTAATATGTCATATTAGTTCAACTCAATATTACAATGGAGTATTTTAATATCAAGTAGCACCCAAATATGTAGGGTATAGGTATTCCAGAGAACATAAATAACACAAAGAGGTTATTGTTCTGTGATAAAAGAGGTTATAATAGATCTGAGACTGCTGTGGGATACATACATACTTAGCCGCTAGTCGATAACAAATGGCGCTAGATATGTATAGGCGATATAATATTTTTAAATTCTTATTTCACGCCCAATATAGTTGCAAGCAACAGGTAGAAATATTATAAACGTTCAATTGCATGTGTTGGGGGCCCAGAACATGATAAAGGGGGCGATAATTTCATATAGCATCATTTAACTACTTAAGTTATAAAACTTCATTCGTACACCCATTGTTATAATTCATGAATAGTTGAGGCATAGTGTAAAGAAAACGACAAGTGAGTAATCTCATAAGAGCGCGCGCGATGCAGACGCGTGGCGGCGGCGGTCGGTATAGCGAAGTCTTAGGGTTGACACATTTACAAATTATTTCATTGCGTTGTAAGTTTGCAACTTATATCAATATCAATATCTACTCTCACATCACCGAGCGCTTAAATCGTGAAAAAAAGGCTTAAGCAACGTTACATACTAAGTAGCGTCAACATATTAAGTACACAATATTTTAAAGTGGAAAGAAATTAAATTTATATTTTTATGCATTGTCAACATAGGTTTTTAACTATACGGAAAGCAAGAAAAGCATTACAGTACTTTAAAGAATTATAGTGTTCCGTCTAAAACCCCGTGTTTACGAAATAAAAATAAAGAAAATACGTTATTATGAAATAGACAGAAAAAATAAACATACGGAAAATATATTATTTTAGAGGTACAACTTGGTCTGCTTGAAATGCGTAAAAATAGAATGCTTTTGAATTGAGACAAAATTGCAAATTGAGACGTTAACAACAAATTACATTCGATAAGAAAAAAGGTTTTCTATGCGTTCCACAAACATAGCCTGAAGCTTAGAAAGCTCCAATAAAAATTCCAAAAACAAAAGTGCGCTTGATGACGCCTGTGTTGAATTTAGTAACACTGTGAGACTCTGCAGATGGTTTTTGTTGAACAAAGCGACGACACTATTGAATTTATTAACATCATAATTTTTTTGATATGCCAAACTCCAGCGTCAGTATATTATTATCTTACTACGACCTAACAAACCGTACAAGAATTTCGTGAATTTTGACTAGAAGTTTAAAAAATCGCAACTCATTGTTTTTATACGACGCAGAAATGTATCCATAGTATGGATACATGGATTCCATACACCATACAAGTGGTAAAAACCATTTCGACAGGCTAAATAACGTGACAACAATATATGCACACGAATTAATTTGTTTCGACACTACTACCGGCGATCACCATAACATTTTGGTACCACACCGCAGAGAGCGGGGGCAGGCGGGCAGGCGGGCGTGTAGAGCCCGCTGCCCGCTGCGCAGGCCCCGCACAACCGCTGAGTGACATCACTAGTCGTTTTCTTTACACTATGGTTATGTTTCGGGAACTTCTCCTTAGCCTCACTTATGAAATCACTAATTATTATTATGTACTCTTTTATTAATTATATGTATATAAAATTGTATATTGTT

>SinsCEX12

GTATACGAGTACTTTGATATTTTAGCGAGCAGGTATAGCGCCAACATGGCAAGGCATACGAGAGCCACAACAGTCTCATTGCTCGATAACGTCAAAAGTCGAGGAATGTCTTCAGTTAGATGTCTACATGCCATTATCTACCGTTTCAATCTGGCCTGTACTCGTATGGGTAACTGGTGGTACTGGACCTTACAACCCTGGCAAACTAGTCCTTCAGGGAATTATCGTCGTTGTCGTTCAGCACAGATTGGGGCCACTAGGATTTTTGTGCTTGAATGAAGAAAAAATCCCAGGCAATGCCGGTGTTAAAGACGTAGTCTTAGCTTTACGATGGGTAAAAGATAACATAGTCGCATTTAAAGGAAATCCAGCTAAAGTGACTGTGGCTGGTCAGGGTTTCGGTGCAGCCATGGTAGAAGCATTAATGTTATCTCCCATGACACATGGTTTATTCCATGGAGCGATATTACAAAGCGGAACAATTTTAAGTCCATGGGCCTTTAATTATGATGCAAAGGAAAGAGCACGGCAATTAGGAGAAATGTTCCCAGAAAGTGATAACATAACATCAGTTTTATACGCAGCTGAAGGTTTAGATTTAGCAGAAAAATTCAAGAAAATAGATGTCCCTTATTTTCCATTTGGGATTTGCATAGAGAAATCTTTCAAAAACGAAGAAAGATTTCTTTCTGAATCACCTTTCCATTTACTATCTAATAAAAAAGTAAG

>SinsCEX13

GCGAGTTGAGGTTCCAGCCTCCCAGAATGATACTGAACTACAACGAAGAAGTGGATGCGAGTGAGGAGGGTCCAGCTTGCCCTCACCCCGTGTCGGAGACCTATTACCTCGACGAGGACTGTCTCAGGCTCAACGTGTACACACCCAGTAACAACAGCACCAAGCCGCTGCCGGTGGTGTTTTTCATCCACGCGGGCGCGTTCTACTCCATGACGGGGCGCAGCGACCTG

>SinsCEX14

CTGAGCTAAATTAAGTCAATATACCGTCACCTTACTAAGGAAAACGACGTAACGTACAAACAGTGTTACATAAAAAACAACATTTTTTATCCAAAAATATAATAGACAGATCGAAACGTGAATAAATGTTATTTATAAATAAGTGGTTTAAAAAGATAAAATGGTAAAAGTGAAAGTAAATGAAGGTGAATTAGAAGGAGAGGTGATAGAAAATGTTCTAGGAGGATCGTATTACAGTTTCAAAGGAATACCTTATGCAGAACCTCCTGTCGGAGACTTGAGATTCAAGGAACCACAACCGGTAAAACCATGGAATGGAGTACGCAGTGCAAAAGAGCATGGGCCAATATGTTATCAATACGACATGTTCAGTCAAACTATACGTCACGAAGGTAGCGAAGACTGTCTCTACTTAAATGTTTATACACCAGACTTAAACCCTAGTGAACCTCTCGCAGTAATGTTCTGGATCCACGGAGGTGGATTTGCGAGCGGTAGTGGCAACACCGATTATTACGGACCAGATTTTCTAGTCAAAAATAATGTAATCCTCGTCACCATTAACTACAGACTTGCAGTTCTCGGTTTCCTCTGCTTAGATACTAAAGAAGTTCCAGGGAATGCTGGTATGAAAGATCAAGTAGCAGCTCTAAGATGGGTAAAGAAGAATATTAGCAATTTTGGTGGCGATCCAAATAATATAACGATATTCGGTGAAAGCGCCGGAGCAGCAAGTGTTACGTATCATTTAGTATCCCCCATGACCGAAGGTCTGTTCAAACGAGCAATTGTACAAAGTGGCTCCAACCTTAGTGAATGGGCGACGGCCTTCGAGCCGAGGCTAAGAGCTGAAGCACTGGCTCGACAATTAGGTAATGAAACCAGAGACGATAAAGAGTTATATGAATTCTTCAAATCTGTACCAAAGGAACAATTAGTGGCCGTTCAAACACCTATCACGACGGAAGAGGAGGCCGGTTTTAAATTAACATTAAATTTTAGTGTTGCAAATGAGAAAGAATTCGATGGTAGCGAAAGATATTTCTATGGTGATGTTTATGAAAGACTATGTTCTGGTATTCACGAAGGAGTAGAAGTCATAATAGGATACACAACACATGAAGGAATAGTCAATTTTGCTTTATTACCAAACGTATTTGAAAACTTCAATAAATACCTCCAAGCTTTCGTGCCTTTTCCCATGATATCAGATTGTTCGAAGAGAGCACAATTAGAAATCGGAAGAAAAATGAAAAATTATTATTTCCACAATGAGAAAATCACAAAAGAGAATGCTATTGCCCTCGTTAAATTTTTGGGAACCATTCTAGTTGCATATGATTTGTTACAATGGACAAAAATATGTTCAAAAACAAATACAAATAAATTGTACCTTTATAAATTCGATTGTTACTCAGAAAGAAATATATTTGTTGAAATATTCAGAACGGGAGAGTTTGCGGGAAGAACCAAGATCGTGTCACATTGCGATGATTTAATGTATATATTCCACATGAGGGATTGTCCTCCAGACGTTAACTCAAATAGCTACAAAATGATCCAAAATACAACAAAACTATGGACAAGTTTTGCAAAATTCGGGAACCCAACACCAGATAACAGTTTAGGTGTAAACTGGCCTGTTTTCGATTTGAATAATCAAACGTATCTACATATTGGAGAAGAACTCGAGTTAGCTTCACATCCAGAGAAAGAAGAAATTGAATTTTGGGAAAGCATATACAAAAAATATTTACCCCACAAATACTATCTTTAATATCTGCTATACTTTGTATTTATGATAATACTGTACTTTTATATACCTACATAATATATTTAATTTAATTAAAAACATATTTTATACATGCAAATAGTATTAAAAATGAAACTTATTAAATTTTTGCTAAAACAATCTATGCCTCGTCACTTCCCAATAAAATTTTAAGAATTCAAAAAAAGTCTTCCCCATTTTTACTATACCATGCTATACCACTATCCCAAATAATTAGAACTTCTAACACGTGTACATGAACATTTTTATAAATATAAAACCCTAACCTTTATACTATTTTGATTTATGCAGGAACCCAACACCAT

>SinsCEX15

CCTACTGGACCAAACAGATTTAAGCCACCGCTTCCACCGGCAGTGCGACTAAGTATTTTAGATGCAGTTGACAGAAAAATAATTTGTCCTCAAGGTGGAGTAATGAATATGAATTTAGCAACAAATAATTCTATGCAAGAAGATTGCCTAGTCGCTAATATCTATGCTCCCGATACAGATGAAACAAATCTTCCAGTTGTAGTCTATGTGCATGGTGGCGCTTATCAAGTTGGATTTGGAAATTGGTTAACGCCAAAAACTTTAGTGCAGAGCAAAAGAATTGTCGCTGTTACGTTTAATTATCGTCTCGGAGTGCATGGGTTTCTCTGTCTTGGCACTGAAGACGCGCCTGGTAACGCAGGTATGAAAGACCAGGTGGCCCTGTTACGTTGGGTTAAAAAAAATATTGCTGAGTTCGGTGGGAACCCTAACGATGTTACCATCGATGGATATAGTGCGGGATCTTCATCTGTTGATCTGTTAATGTTATCAGAATCAACGAAAGGTCTGTTTCACAAAGTGATTCCAGAAAGTGGTGCTAACGTTGGAGTCTGGAGCGTCCAAATTGACCCAGTTGAAAATGCGAAAATCTATGCCAAAGCTCTCAATTTTTCAAATGTAGATGACATATATGCCCTAGAAGAATTTTATAAAACTGCATCCATTGAATTACTCTTGACTGATACGTTTATGGACAGATATGATTCTACTTTTCTTTTTTCACCATGTGTAGAACGTGAAACTAGAAAAGAAGTATTTCTCAAAGAAGCACCTGTGGATATTTTAAAAAGGGGCAAATATAGAAAATTTCCTGTGCTATATGGATTTGCTAATATGGAAGGTTTGATAAGAGCATTATATTTCAATAATTGGAAAGATAGAATGAATGAGAAATTTTCAGACTTTTTGCCAGCTGATTTACAATTTGAAAATGAAGAACAAAAAGAAGAATTGGCAAAATCGATTAAGAAGTTCTACTTTGACGATAAGCCCGTATTAAATGAAACTTTTGTAGCATATATAAATTACTTTTCGGATGTTATGTTCACATATCCGACTCTTAGGTCTGTGAAACTGCAAGTGGAAGCTGGTAGTGACTCAATATACCTGTACGAATATTCTTTTGTTGATAATTTTGTCTCGAGACTTGTAAATTTACCAGAGTTTAAAGATTATTTCAAAGATATAAATTTAGACTCTGTGTCTGGAGCAAATCATTGTGCACAAACCTGGGCTCTTTTAGATGATAATAATCTTTTTGTCATTCCTAATGAAATAGTTTCTGATGACACCGAGAAAATGAGGTCGCTGATGAGAGAGTTGTGGCTGAATTTTATGACGACCGGCAAACCAGTTCCGAAGGGATCATCTCTGCCTGATTGGCCATCAGTGGGTGCTGACTGGAGCCCACACATGTCTTTAGGAAAAAATATTAAACTTGGCGGTTCTCTTCTGCCAGAAAGGGCGCGTTTTTGGGACGCAATCTACGAAAAACACTACAGGACACCGGCGCCACCACCACCTCCACGAGCTCGTAGATACAATGAGCTTTAAGTTGCGACATGTTTGTACATAAGTATATTTAATTAACACAAAAACTCAAGATTTCAAATTCAAATTAATTTGTTAAAG

>SinsCEX16

CTCATTTTTGCTAGTATGGAGATTAGTGATAAAATAATTCAGTAGGAGGTTCCTTCCGGCTATTTGGGACAAGTTAAGTAACTGTATAATTGTATATGCAGTAATGTTTCGACTACCGTTGTGTTGTGTTGTGAATGTGGTAGTGTTGGTCGCTTGGTGGCCATCGCCAACCAATGCGGTCGTTGGAGGAGCACCAGCCGCTGCACCGGAACCGGACGCTGCGGTGGTTTTTGTCCAGCGATACGGTCGCAGTGCACGTATCGAAGGCTTTAAAGATGATAAAATGGGCTACTACAGTTTCCTTGGCTTAAGATATGCGGAACCACCAGTAGGTCCAAAAAGATTTCAGCGTCCCATACGACAAATTCTATTTGGTGAACAGTCTGCAATCAGGCAATGTTTGCCATGTCCCCAACGTGATCCACTACGTCCAGGAAGAATTATAGGAAATGAAGATTGCCTTTGTTTAAATGTTTACGCTCCCAAAATGCCAGGAGATGAACAAGGCTGTCCTGTTGTCTTCTTTATTCACGGTGGAAATTATAAGAGTGGTTCTATTGTATCTTACGGCGGACAACACCTAACCCAAAAGGACACCATACTTGTTACTGCTCAATATCGTTTAGGTATATCTGGATTTTTTAGTACTGGACAAAGAGATGCTTCTGGAAATACTGGGCTCTTTGATTTGCACGCCGCTTTGGCTTGGATAAATGATTACATTGAATTCTTTGGCGGGGATCCAACTCGTGTAGTTGTAATGGGACAAGGTTCAGGTGGAAGCGCTGCCTCTTTACTGGCATTGTCAGGAGAGGGTCGTAATGCGGCTGGAGTGATAGCTTTGTCAGGAACACCGCTGTCACCGGGAGCAGTACGAAACGATCCCGCTAAACACGCGACAGAATTAGCTAACCGTACTGGTTGTCCGTCAAAACCAGCCGAAAGACTCCTCATCTGCTTGAGGAAACTTCCTATAGAAAAAATCATTCAAGCGGATTTAGACCTTACAATGGGTATGGTCGATGCGAAATCATTCTTGAACGAGATTTCTGGTCGTACTGGGTTTGGTGCTCGTGTTGAGGGTGAACATGATCTTAGATCTTTGCCACCAATTGTGTCTGAACAACCAGCGGAATCTCTCGACAAAAAAACACAGCGTTGTCCATTACTAACTGGTGTTACTTCTGCAGAAACTTCTCGAGCTGTGCTAGGAAAATATTCCGGCTTCCTATCGGACCAATTGTCGAAAGTTCATGATTTTATAAAGAAAGATATTATCGGAGGTCTACATGATGTAGTGAACGACGTGCGGGGCCTTATACCTGTGAATCCAGCAATTGAAAAAGTTATACCTATTACTGATTACTATCAAGCTCTCTTCGACAGACAGATGAATACTATGGACGGGCTTATTCAGATAGCGGAAGCAACAGGAGATGCTCTGTTCAATTTTCCCGCTTATCAAACCGTGCGAGCGTGGAGCGCGGCCGGTCCCGCCTACCTGTACAGTTTCGAACACGTTGGCAATCTGAGCAAAGGTGCGCATTTTCTGCCAGGAGTACCGCTCGCTGATGAAGGTGAAGGAAAAGAAGTTTACAAACAGAACGGCCCCTCGCATGGAGACGAACTCGCATACATATTCGAACCTTTAGACAATGATGGGAATCCGGTGGGAGAACTGGTTTCCAGCGTCGACGGTCGTGTCAGGGAAAACTTTTTGAACCTCATCTCAAAATTCGCACACAATCTGAGTCCAGTGCATGAAGATAACAAAACGCTATTGGACCTGTTACCATTTTCTCAAGACAATGAACAGTTTATAAAGATCGGACAAGAAATAAAAACTGATTCTAAATTCAGGTTTTGCGAGATGGGTCTATGGGGAGTTATGACGGATAGAATAACTGGTGATAGCTGTCAAAATATCATTGGAAACTTATTAAAATTGCCCAAATCGCCTCTGAACCCACCTACAATACCCGCCATTGTTCCGATACCCGATCCAGTGGCAGTTGTCACGCCAAAACTACAATTAGTGCCCAATCTAATTAATAATAACAAAGAAAAACAAACACCTTCTATAATACCTAAGAAGTTGCCGTTTGGATTTATGAATTATTGAGTTTGATTGATTCTGTACTTATTTCTATTGCAATTAAACAGATTTTCTGTGT

>SinsCEX17

TTTTTTTAATTCATTAAAATGCAGAAAAAACAATTTATTAAAAATACAGGTAAGTACATGCTTAATAAAAGCTTATAAAAAATTCCGCATAGTGTAAAGATTTTAGATATTTCTTTAGAAAATAACTATGCATGGTTCGTGAAAAATATGTATATACATAATAAAAGAAGTTTTCTGTAGTTGTATTGTTGCTAATAAGAGTCAAGTTTTGCAAAGGAATATTTATTTATTTTGTCTGTAATATATAATTATTGTTTTTTTTAATGATAAAATAGTTAGCGCTTGGCCTCAGTCTCACTTGATAGTAACTGACGAAGAGGTCTAATATGGTGAGCACTGGCCCAATAAAAGCCTCTCCACTCTTGTCTTGAAGAGACCTAAGTTATATGAATCGGGAAATATAGACGCAGGAAGAGCGTTTCATAATTTGTTAGTATTATATATAATACTAGCTTTCCGTTTATAATGACAAATAAACTATCTAAAAATAGTTCACTTGCGGCTTTCTCTAAGCCCGAAAACACACGCACCGATTTTGCCGCCTGGCAGCGTGTGCTGCCTAGCCACCTTCCATACAACATATTCTATATTGCCATTGAAGTCAGCGCTGGGCCGGGCCGGCGGTGGCTGTCGGCCGGCAAAATCGGTGCGTGAGCCTCTAGGCAAAATAAATAATCGAAAATTACAGAAACGATTTAGGAGCACGATAGCGAATCAGTCAAGTTCCTGTAGTCTGTTGTACATTTTTTAAAAGGACAATCCAGCCATATCATATATGCTCTTCCAGAATTGTACGCGTTCTGCATTCAAAGCTTTTCTAATGGTCAAAGTGTCGCCGATATCGCCGTAAGTCTCTGTGACGTTATCGTATTCGGGCCAAGTGGCGCCCAACGATGTGTCTGGAGTTGGATTCCCATATTTTGCGAAGTTAGTAAACAAGGTACATGTCAGATTCACAAGCTTGTATTCTTTGCTGTTTTCATTAATATCTAAATTGGCATGTTTCGGATCGAATAAGTATGCCGTATCGTCTAAATGACTGGCTCCAATTATACCATATTTGATGCCCTGGTTTCCGTAAATATTTCTTTCAGATACACAAGAAAACTTGTAAAAGTATCTTCTAGAATTGCCCACTTTTGGCAGAAGTCTTGTGTACCTGTGGATGTCATAAATGAAGCTACTTTCCGAAACGTAAGTTATAAATTCTTTCATAGTATCAACATTAATGGGTCGATTACCAAAAAAGTGTTCACGAATCTTGTCTGATATATCCAAAATTTTATTAGGAGTGCTTTCGAGCAAAATCTTTCTAGGCACAACCAATTCTGGATATTTATCGTATGCTTTCAAAATTCCTTCTTCGAAGGCGCTGATTCCAATAATTGCTTCTTCACTGGTGTAACCAATCAGTACATCCACTTCATTGACTTTACCTTTCTTCAGTGCTTCTTCTGGTGTTTCAGTCAAGAAATGTTCTCCACCGAAATCTTTTTCGACAACTGGTGTGAAATGATACATCTTAATAATGTTATTGGTTACTTCTTCCGACGCTAACACACAGGGATTAGTGCTCACTAGCTTTTCAACTGGAACACTTTGAAGGAATTCAAGAAGTTTTGCGGGATCATCAGTATCGATACCTAATTCTCTCCCAAGAGCAAAGGCACGTCTACTAGGTTTATAAGCGACAGACCAATCGCACAATGGCACTCCACTCATCGGGATAGCTCTTTTGAATAGACCTTTTGATAACGGCGAAATTATATGAAGAGCAGTGGACGCGCCACCAGCGCTCTCTCCAAAAACAGTCACATTATTTGGGTCACCACCGAAATTCACAATGTTTACCTGGACCCATTTCAAAGCGGCTACTTGATCTTTCATTCCAGCATTACCAGGAACTTCTTCGGTGTCCAAGCAGAGGAACCCTAGAGCTTCTAAACGGTAATTTATTGTAACTAAAACTACATCATGATTGACAAGAAAATCGGGTCCGTAAAAATCTTCGTTCCCTGATCCACTTTTATAACCACCTCCATGGATGAACACCATGACTGATAGAGGTTTATTGGGTTTTAGTTCTGGTGTGTATACATTTAAATACAGACAATCTTCGCTTCCAGTTTTTATTTGGTTTGTAAAGATGTCTTGTTGGGGACAAACTGATCCATGTTCTGTTGCTTTTTTTACTCCGTCCCATGGTAGCGGTGGTTGAGGCGCCTTAAAACGTAGTTTTCCAACGGGTGGCGCAGCATATGGTATACCCTTGAAGCTGTAGTAGAGACCAGTACCAGCGCTGGTGGTCAACTGCTCGCCCTCCAACCATCCCTGTTCTACTTTTACTTTCGCCATTTCTGCGTTCAACTCAATCGAAGATCTCTGGTCGCGGACTGGCCATTAATAATAAACAAATGCACACACACACATTTAGAGATAATGTATGTTATTATGTACTATATTTTTATAACTCTATAGTACTATAGGCTTCCAGTCACAAAATCGAGTCGTAACAACATACCAAAAATATCGATATCGGTCAATTTAATTTTATTGTGAAAAATATTCAAAACCGTTTTAAGACAATGTTTTTATGTCTGCTAGCAATAAACTCTTGGTATAGATAAAATTTAATATTGGTGGTGGTGGTGTCTATAAAGCTAGCAAATCAAACACCAAAGCAGGATATCTACTCCGAACATATATCGTCATCGTAA

>SinsCEX18

TTGCAGAACTGTGTAAGTACATAATTTAAGCTGTATAATATTATTCTTATTAAAAATACAGACAACATTTAAAAAATCTATATAGGTAAATATGAATACATAAAAACTGTTAAGCAGTTATAATTGTAAATTATTTGTTATAAAAGTAATGCTTACAAACTAAACACTTATTAAAAATGTAATTTAAATGCTAGACAAGTGATAGACACTTATTTTAACACTCCTTTATTAATTTATCTTGTATTTAAGTAATCAAATTATGCAATTGAAATTTGATAATTAATTATCATTATAAAATATCATTAGAATTTTCGTTCCGTTTTCGATCATGTCCCATTTTAGGTCTTTTTTTTCAGAACAATCATTCATTCACGAAAATACTCTTAGGTAGGTTCCGAACCTGTCCAAAGAAATTTCAAATAATATGGGTAAATTCATAGTTGAAAACTGAACGAAATTTCGTCCGAAATTTCGAAAATTGATTTCACGGTAGTCCCATCTGTAGGATATCTGTAGCCTTCCAACATAATTTCAAAACCATGACGGTTCTTTGGTTACAGTAGACATGGCGTGTTTATAATGGTTCTAAGGGTATTTTTTGATACTACGTATAATTACAATGATCAATCTCCATAAATAAGATCAACAAAAAGAATATTTTATTGGAACGAATTCTTATTTTAGGTATTTTTCATAAATTTCTTCAAATATTGCAATTCTATTTGGCTGATAATTAGACCTTATTTCTAATTCTTTATCGATAACCAAGTAATTAAGATTATCATTGTTGTACGTTGGCCAACGTATAGGTATCAAATCGCTGGTATTCGGTACAGGGTTCCCAGATTTAGCGAAGTCCGTCCACATTTTATTGATTCTTTGTCTTATCAATAAATCTGTTTTATTTGGGTTTGCTGTTCCTGGAGGACTGAAACTGCTTATCGTGTAATTTGTATCATCACCGTGGCAGGCACCCTTCGCCTTTATTCCAAAAATCATTTTTATAACATTCAGAGAGCCATCATACGCAAACATATATGTATGTACTGATACACCATTCTCAGCCATTAGTTTAGCGCTTATCAATATACCACTTGTAAAATCGAAGTCGCCAATAAAGTCGATAATAGGCTTATCATCATCGTCTCCTACACCATTTTCTTCAATATATACTTTTTTGAACTTTCTATTATATTTATCAATGTCGTCAGAATCTAAGGGATATGTCCAAAAATCAAGAAATCTTTGATTTTCCATCAACTCCAAGACGTCTACGGGTTTCAGGCCCTTTAATAAGAAACCTTCATATTCGCAGAATCCTCTAATCACAGATACTTTGTTAAATCTCCCCTCTTTGAGTAAATTGTACGGATATTCAGTAAGGAACGCTTCGCCATTTCCAAAATCTTTCTCGATGGACGGTACAAAACCAAAATATGTGCCTTTTGCAGTATTAATATTAGAAACAGTGAAAGCTACGTCTGATAAAGATTTTGTTGGTTGTTCAAGTAAAAAGTTATATATTTGAGCGACATCGTTGCTAGGTCCCTGGTATCCAAGTTCTAGTGCTAACTTGATGGTTAATTCTTTAATATTATAATTAATGGTCCAGTGATTAAGGGTCGAGCCTGATTGTAATATAGCTCTATGGAATAGATCTGCACTCAAGGGTGATAATATAAGATATTCTATAGAGGCGCCTCCGGCGCTAATACCAAAAAGGGTAACGTTATGTTTGTCTCCACCAAAATGGCCAATATTATTTTGTACCCATTTCAAGGCCATCACCTGATCTTTCAAGCCCATATTGCCAGCAGCTTCGGGTATGTCCAATGAAAGAAAACCGAATACAGTCAGCCTATAGTTGATAGCGACAATTATCACGTCGTTTTCTAATAAAAATTCAGGGCCGCTCTCAGTTTTGTATGTTCCACTGCCGCATACGAAACCACCTCCATGAATGTAAACCATCACAGGAATTGGTTTCGAATTGTCTGCTGGTAAAACGGGAGTGTAGACGTTAAGATAGAGGCAGTCTTCGCTACCTCGGGTCGTTTTTGATATCAAATCAAATTGTAAGGAAACATGATCCAACTTCGTCGTAGTAGCATATCGCTCTCCCTCCCATGATTCAGGTGGCTGAGGATTCTTAAATCTTAAGTTTCCCAGTGGTGGTTTTGCATATGGTATTTCAAGAAACTCAAAATATTCATATTCATTTTCAGTTTTACATTTCTTTCCTATTAACGTGCCTTGGTCTATAGTTACTTTTGCCATGATTTGGTGAATTCTTAATTTATTCGTTTTTGTTATATTATTTATTAATATTTAATTAAATAGTTATGAAAAACGTCCGCGACGTACTTAGTTTTTGAACAGTATGGACA

>SinsCEX19

GGTATCGTCACCATGATATGAAAGTTATGATGATAAGGAGAATTAATAACCACGGATTCGTACATAAATAGTATACGACTAACAAACGAATCAGACGACTCAGTATGCAATGCTCCACAATGAAGTGTAGTAGTGTGTTGGTGGCGGCGTGCGCGGCGCTCGCGGCGCTCGCGGCCGCGTCGCCGTCTCAGGAGGGCGAGGAGCGACAGTCCCGGGTGGTGCCCACCAGCGAGGGTCCGGTGCGCGGCTACAGGGAGGACAATGGGCTCTATGTCTTCTACAATATACCCTACGCCAGTGTCCCTACTGGATCAAACAGATTTAAGGCTCCACTACCGCCGGCTATTCGATATAGTGTACTGGATGCTGTTGATAGAAAAATAATATGTCCACAAGTCATAAATATGATGACAACACTAAATAACAACATGCAAGAGGACTGTCTTGTTGCAAACGTTTACATTCCTGATACAAACAAAACTAATCTACCCGTTGTAGTCTATGTGCACGGTGGAGGTTATCAAATTGGTTATGGGAACTGGCAAACGGCAAAAACTTTAGTGCAGAGTAAAAAAATCATCATGGTTTCATTCAATTATCGTCTTGCAGCCCATGGTTTTCTGTGTCTTGGCACAGAAGGCGCGCCTGGTAATGCAGGAATGAAAGACCAACTGGCTTTACTTCGCTGGATTAAGAAGAATATTGCTAATTTCGGAGGTAACCCTGACGACGTTACCATAGAAGGGACCAGCGCAGGTTCTTCAGCTGTCGACCTTCTAATGCTCTCAGAGTCTGCTCTAGGTCTTTTTAATAAAGTTATTCCTGAAAGCGGTGCCAACGTTGCGGTGTGGAGCGTTCAAATAGATCCTTTGGAAAATGCAAAAAGTTTTGCTAAAACACTTAATTTCACTAATGTTGATGACTTATATGCCTTGGAAGAATTTTATAGAACAATTCCGCTAGAACAACTTTTCACTGATACATTTATCGACAGACCCGATTCGACTTTCGGTTTTTCACCGTGTGTAGAGCGGGATACAACGGAAGAAGCATTTCTTAAGGAAGCACCTGTTGACATTTTAAAACAGGGAAAATTTAAAAAGTTTCCTATATTGTATGGATTTGCTAATATGGAAGGCTTAATACGAGGAAATGTTTTCGATGTCTGGAAAGAAAAAATGAATGAGAAATTTTCAGATTTTTTGCCAGCAGATTTGCAATTCGAAAGTGAAAAGCAAAAGGAAGAAGTAGCTAAATCAATTAAAAAGTTTTATTTTGGTAATAATCCGGTATCAAAAGAAACTATGTTGGGATATGTAAATTATTTTTCAGATATTATGTTCACGTATCCACATCTTAGATCGGTGAAGTTACAAGTTGAAGCGGGTAGTGATTCTATATACTTATATGAATATTCTTTCTTTGACGATTACAACGAAAGATTTAAAAGTTCAGAAATGCAAAATTTTATTAATAACGATTTTGACGTTTTACGGGGCGCTTATCATGGCGCTCAAGCTTTAGCTGTTTTTGACGACAGTGTATTAATCGCTTCAAATGAAACATCTGACAAAGTCAAGAAGATGCGAACTTTGATGAGAGAATTGTGGCTCAATTTCATGATTAATGGCAAACCTGTGCCGAAGGAGTCCTCGTTACCCGAGTGGCCACCAGTAGGAGAGAATTGGAGTCCTCATATGTCTTTGGGCGATAAGATCGAACTCAGAGGCTCTCTTTTGTTGGAAAGGATGCGATTTTGGGATGCCATCTATGAGAAGCATTACAGAAAACCAATACCACCACCACCACCACCACCGCCACCTCCTCGTAGACGCAATGAACTTTGAATTATCGTCTTAGATTTGATAAATTTAAACAGTTTTATCAATAGAGTAATTTAGGCGAAACATAACTTATAGAATTATTGAAAATAACTATAGAAATATTGAAAATTATTACAACTCAATATTTATGCAGTGGCAGGCCTCAATCGAGGTCAGTCCATCTAGCAACCACCTTCAGAAGTCTACCGCTAAACAGAGCCACACTTGTGGTAATTCTTTTCCGTTTATCAGCAAACTTTTTTAAAAGAGTGAACTGGTAATGAGAGGAAATTAATGCTAATAGTAAAAAAAAAATACAATTTGCAATTCTACATGCTACGCTGGCCTATGCTAGGCATGAGCACAATTGCTACAAGGAAATATATCAAAATTAAGACAGAATTGGAGTTGTCGAATAAAAGCTGATGTAATTTAACGAGGCAAAAACAGTCACCGTTAACTACGTACATTTGTGACGGCTATAGTGTTACAAATAGTTATAAGCAAGTATTTAGTGTTCCGTACCAAAATTGGCAAAACGGAGCACTTATGGAATCACATCGCTGTCTGTTTGTTCGTCAGTGACATAAAAAGCTGAAGCTTAGGCATTGTTATTGCAATTAATGGGTCAATTACACAAATGAAGTTAAAAAAGGGGTAAACAAAAAAAATATCTACATAATTTTGTAGGTTATGACCTATTGAACGCTTATGAAAATACGCGTCCAATAACATTGTTTGAAAAGTGAACGTTTTTTTTTACATTTTTTTTTGCCAAAATCTCAAACACGCTATCCCCATCTGTATTTCATAAAAGTATACCATTACTTTGGATAGTTTTATTTGTAC

>SinsAOX1

TGCCGCCCACCAACGAACTGAAGACCTTCTCTGTTAACTCTTCCACTCAGACGAAGTCGCGGGTGACAGCTAGTGTGAAAATGTGTTTATTTTCCTAGTGTCTGGTGTCAGTACTATCTTGTCAGGGCTGGGAGGTGATGACTGTAGAAGGTATCGGCAACCGTACGCATGGCTACCACGAGATACAGAGCCGACTCGCCAAATTTAATGGCACGCAGTGCGGATACTGCACACCTGGATGGGTGATGAATATGTACAGTATCTACGAGTCAAATAACAAAAAGTTAAATTCGACACAAATAGAAAACTCTTTCGCTGGAAATCTATGTCGATGTACAGGTTACAGACCAATAGCGGACGCCTTCAAAACATTCGCTAAAGATGCTGACGAGCGACTGCTTCATAAGTTGGCTGATTTAGAAGATTTGGCAATCTTAAAACCTTGCGGAGTCAAATGCAATAAAAAATGTCCACACAAGAAGAAAGACGAAAGTGTACTACTTGGTAAATTAAATTATTCCAATCAAGAAACCAAAGAAACTATATCTGAAGACGATTGGTGCTTCCTTGAGAAGGGTAATAATAAAATGATCGTAATAGATTGCGGTACAAATAAATGGTTTAAAGCTTACACTTTGGTCGACGTATTCAAGGCAATTGGGCGTTGCAGTGATTATAAATTCATAGCTGGTAATACGGGACAAGGTGTATTTCACGTTACGAACTATCCACGCACAGTGATAGACATATTCAACGTAGCCGAACTAAAAGGGCATTCGATAGATGTAAATCTTATATTAGGTGCTGGAATGCCTTTATCAGAAATGATGGAATTGTTCCTTAAACTTTCTAATGAAAACGCAGACTTTTCTTATCTGAAGGGTTTTCACGAACACATGGATTTGGTCGCGCATGTTCCTGTGAGAAATATTGGTACAATTGGTGGTAATTTGTACATGAAATATACTACTAATGATTTCCAATCGGATCTGTTTTTACTTTTTGAATCAGTTGGAGCTACATTAACGATAGCGGAAGCACCCAACAAAATGAAGTCGGTAACAATGCTAGAATTTTTAAAGATTAATATGAAAAACAAAATTATTGTGAATATTATGCTTCCACCACTATCAAGTTGTTGTTCTTTTAAATCGTATAAGATAATGCCTCGTTCTCAA

>SinsAOX2

TAAGATAATGCCTCGTTCTCAAAACGCACATGCTGTAGTGAATGCAGCATTTTTATTCCAATTCAAAAAAGATACCACTATTTTGGAGAAGGCAAATATAATATACGGCAGCATTTCAGCTAATTTCAATCATGCCACTAAAACAGAAGCAATTTTAGCTGGAAAAGATCCTTATACGAATGAAACATTACAATTAGCTTTTAAAACATTGTCTGACGAGATATCTCCAGAAGAAGCGCCACCGGAACCGTCTGCTGCCTACAGGAAAATGTTAGCTCTAACTCTGTACTATAAGGCTATTCTATACTTATGCCCCGATGAAAGAATAGATCCAAAGTATCGTTCTGGAGGTGAAGCAATTAAGAGGCACGTTTCGCAAGGAAGCCAAATGTTCGACACTGACAAAAGCGTATGGCCTTTAAATCAACCAGTGCCCAAATTAGAAGCATTAGTGCAGTGCAGTGGAGAAGCTACATTTGCTAATGATTTATCCACACAAACTGATGAAGTTTTTG

>SinsAOX3

AACTCATGGCGGAATTGAAATGGGACAAGGAGTTAACACCAAAGCCGTACAAGTTTGTGCTTACTTATTAGGTATATCAATAGAAAAGGTACAGATTAAAGGCAACAATACGTTTATAGCTCCTAATGGCTTTATTTCTGGAGGTAGTATAACGTCACAGAATGTAGCTATAGGCGTCCAAAGATGTTGTAACGAACTCCTAAGGAGATTACAGCCTATAAGAAGTCAAATGACCAATCCGACTTGGGAAGCTCTTATAAAAAGAGCTTTTGAAGCTGACGTTGATTTACAAGTCCATGGTTACGTAGGACAGTCTGATGTGCAAAATTATAATATTTACGGAGCAACTTTGGCTGAAGTTGAATTGGATATACTGACAGGAGAATCGGAAGTAAGAAGAGTTGATTTATTAGAAGATGTCGGACAAAGTGTGAGTCCTGAGATCGATATAGGCCAGGTTGAAGGTGCTTTCATCATGGGTCTAGGATATTGGACCTCAGAACAGCTGGTCTATAATCCAAATACAGGAGAACTTCTTACCGATCGCACGTGGAACTATCACGTGCCCCAAGCTAGAGACATACCTCAAGATTTCAGAATCTATTTTAGGAAAAAATCTTATAGCAATGATACTATTTTTGGTGCTAAAGCAACCGGCGAACCTCCGATCTGTATGGCGATAGCGGTGCCCTTTGCCATTAGAGAGGCCATTGCAGCAGCAAGACTCGAATCGGGAATACCCACCACGCAATGGTTCACTATTGATGGACCGTACTCATTGGAAAATATACTGATG

>SinsAOX4

ATTTCAATTCCGCCATGAGTTATAGCAACTGTGCCGTCATCGTGGTAGACTGTTAAATTGACATCGAAATTTTGATATCCTAAAGGCGTCCAACGTAGTAAGGAGAATCTTAATCCTCTTTTTTTCCATCTATTCTCTTTATTAAAATTATCTACTGCAGCTCTTCTTTCGGTATACTGGGAGTTGGTTTTAAGTGTGTCAACCATTCCAACAATTTCGTTATGTGTACTACTAACATTGGCTAAACGTACTTCAAGTGGATCTAAAGATAATTCATAAGATATTCTTTCCATAATTGTTTCAGCAAATGCTATAGCTTCCAATGTGCCTGGAGATCGGCACCAAGTATTCTTAGCGTTGTCAGTAATCACATTATAACAGCTGTAAGTCCATCTTTTATTATCATAACAGTTATAGAAGGAATCAACCGTGAACTGAATTAGCACCTCATTCACCATATATCCATTGTCTTCATATAAATTTTGAGTCATATACTGTATTACGCCTGTACTGTTCACACCGACCTCAAAATTGCTGACACAGGGCAATCTCTTGCCCAATGCTTTAGTATTGGTCGTCATTGGCAATATGAAACGACAAGGTCTATTCAGCTTGAAGCTCACGAGACTACAAGCCACAGCTATTTGAGCGGCTCGTGATATTTTGTGGCCGTAACCACCTCCTAACCGACGCACATGTA

>SinsAOX5

ATAAACCTCCATTCCATCTTCAGTCAGTTTCGTAACACACGTTTGAGGCTCCATGTAATAGTGGTATTGAGTATCTATTTTAAAATCTCCATGCAAAACACATTTGACATCATGTCCAATATCCGTTGGTTCAACTGTCTTATCAGTAGTTACTCTTTGATTCTTCTCGGGAGATTTTAAAACATCTTCAATAGTTATCAAAGGCTTCTTTATGTCTACACTTTGGTATTTAATTTTAACTAATTCTGCAGCTCTATTCGCCACCTTTTCTCTATTAGCAATTATAATTCCAACTGCTTGTCCATAAAACTTTATTTCCTTTGAACACATAATTTCTTCTTTGACTGTTAAAAAAGGCAAATTTAACGGAGTGAATGAATTATCCCCAGGTATATCTTTTGCGCTATAAAAGGCAACAACACCAGGAATCTTAAGAGCTTCAGACGCATCGAATTCCTGGATAATACTGCCTGGTTTGGCATCAGCACAAACAAAGGCACCAAAAACTTCATCAGTTTGAGTTGGTAAGTCATTAGCAAATGTTGCTTCTCCACTACATTGTACTAATGCTTCCAACTTAGGTACAGGTTGGTTCAAGGGCCATACGCTTTTATCAGTGTCAAATATCTGTGTTCCTTTTGAAGTTTGCCTTTTTATTGCTTCGCCCCCAGAACGATATTTCGGGTCTAGCTTATCAGCAGGACATAAACTTAATATTGCCTTATAGTATAAAGCAAGTGCCAGCATTTTCCTGTATGCAGAAGAAGGTTCTGGCGGTGCTTCTTCTGGGTTTATTTCATCAGATAATGTTTTTAAAGCTAACTGTAAAGTTTCATTGATATATGGATCTTTACCAATTAAAACAGCTTCCGTTTTAGATGCATGTATGAATTTTGGCGAAATACTGCCGTATACTATTGTAGCATTCTCTATATAATTTGTGTTGCGTTTTAATTTAAACAAAAAACCAGCGTTAACAACAGCATGAGCATTTTGAGAACGAGGCATTATCTTATATGATTTAAAAATATGGCAGCGTGATAGTGGAGGAAGCATTACATTTATTATTATTTTCCTTTTCATATTAGTTTTTAAAAATTCTGGTAAGCTCAAACTTTTCTTTTTGTTGACATCTTCAGCTATAATCAAGAGAACATTGACTTGTGTTTTATTATCAAATTTTACATCATATAAACACAACAATTCAATGATATATATCATAATACAATGGAATATTATGTATTCTAAGCACTTTCTTGTCTTGGAGTATGAAAATTTAGATAATTATAGTATCTATACATAAATACATTTATTACATAAATATACTATCATACAAAATCAAATATATAAATATATAATACATGCATGTATTGTGGTAATGTGGAAATGTAAAATAATTACGAAAATTTCATCAACTAAGAAGTTTCATAGTGGTTACATGTACTCTGAATACAAATGAATACGCAATTATAAAAGTTGAAGTTAAAATGATACCAATAGTAATTGTTGCCCCAACGGTTTCAAATAACAGAAATAGGTCTGATTGAAAATCATTATACGTATATTTCATGTATAAGTTTCCGCCAATTGTGCCGATATTTCTGACAGGGATATGCGCTACTAAGTCCATATGATCATAAAAATGTTTCAAATACGAGAAATCTTCATTTTCTTGCGATAATTTTAGGAAGAGTTCCATCATTTCTGATAATGGTATTCCTGCACCTAATATTAGGTTGACATCTATTTTGTAACATTTTATTTCTACTACATTAAAAATATCAACAACATTCGCTGGATATTCTTTTACATGATATACACCTTGTCCAGTATTTCCAGCTATCACTTTATAATTATCACAATGTCCAATTACTTTAAAAACATCTGCTAAATTATAAGCTTTATACCATTTATGAGTACCACAATCTATTATTATCATTTGGTTATTTATTGTTTTATCAAAACACAGTGGCGCCTTAAAATCTTCTGTGATTGAATTTGGATCTGACTGTAGCTTATCTTTGTCGTGAGAGCATTTTTTATTACAACTTAGTCCACACGGAGTTCGAAGAGCTAAATCTTCTAAGTCTGTTAGCTTACATTTAAGATTCTCATCAACATCGGTTGCAAATGTCTTGAATGCATCAGCTATTGGTCTGTATCCAGTACATCTACATATGTTACTTGCAAAAGAGTTCTCTACTTCTGTCATTGTTAAGTTCTTATTTTTTGATTCGTAAAGGCTGTACATATTCATGATCCAACCAGGCGTGCAGTATCCACATTGTGTACCGTTAAACTTGGCTAGTCGGCTCTGGATTTCGTGGTAACCTTGCGTGCGGTTGCCGATACCTTCCACTGTGGTCACCTCCCAACCGTGACACGATAGTACCGACACCAGACAAGAGTTAACAGAGAAGATCTTCAGTTCG

>SinsAOX6

GGCTGTGGCCATCAGTTGAGTGGGGACGAGTTGTTGCGGCAACTACTAATCTAGAATATTTATCAATGTTAAAATATAAGGAAAGTAATTCGACCATATGATAGTATGTTTTTATCGACGTACAGCGACATATGTTGTAAAATTTATATATTATTACTTTTATAAGTTATAATTTTTGTTTTTAAATAGGCCCAATAAATGAAATATAATATCATTTTAACTTGAAATGTTCAAACTTGTGCCCGATTGCCATAAAGATATTTTCTACGTTGCAAGGATTTTCTATTTCTATCCATTGATCTTCATAGCCAGCGTCGAGTCGCGCTGATCTAATGGCTTCTCGGAAAGCATGAATTATTACCGTTGCGAGATTCAGTGATGGTTCTCCAGTAGCTTTTGATTGTAGCACACCAAATTCATTTCGGGCATTCCTTCTGAAATAAATTCTCATGTCGGCAGGTATATCTTTAATGCCAGGAGGTTTGTATGTCCAAGTACGATCAGTGAGCAGCTTTCCTGTATCAGGGTCGTATACGATCTTTTCTGATGTCCAGTAACCTAGTCCCATTACGAATGCTCCTTCAATCTGTCCAACATCAATTTCTGGATTTAAACTTCTTCCAGTATCTTCGAGCAGGTCTACTCTTCTAATATCATGGTTTCCAGTTAAGATGTCAACTTCAACTTCCAATACACAAACTCCGTATACTTTATATGGTTTCACATCAACATCATTGATATTGGAAAACATATAAGTGGCTTGTAAGTTTAGCCCTAACTTGTGGGCTTCACTAACGAGAGTTTCCCATGTGGGGTTATTCAGTTTTTCTCTAACAGGTTTCAATCTCTCTAGGATTATTTGGCATGCCTTCAATGTAGCAAATCCAATGCATTCACTGCCGATACTGCCTCCAGTGATCATAGCATTTGGTGATAGGAAACTCGCGCTTGGCTTAACATTGATTTTCTCCAGAGGTATTCCAAAAGCATATGCACAAACTTGTGCTGCTTTTGTATTAATACCTTGTCCCATTTCAATACCAGCGTGAGTTATTGTAACTGAACCATCCCCATGATACACAGAAACTATTGAGTTATAATTACCAGGATAAAATATATCTACCGTCATCGGAAGTAATTTTATAGCTCTTTTCCTCCATCTGTTCTGTTGATTGAATCTTTTGACTTCTTCAACTCTTTCATCATAATTTGACTCTTTCCTCAACTCGTCTATCATATCGGGAATTGGATTGTTTTCTTTAATCATATTCAGCAATCTCACTTCTAAGGGATCTTTACCTAGATTGAAAGCTATTCTCTCCATCATGTACTCTATCATAGCAATACCTTCAGTGGTAAAAGGAGCTCTGCACCAAGTGTTAGAATGAATATCTGTTAACACAGTATTAACTTGAATGTACCATCTTCTGGAATCATAACAGCTAGGGAAATGTTCAACAGTAAGAACTGTATTTGTTTCGTTGGGTGTAAAACCACCGTTCTGATAGATTATATTTTTTAAGTATTGAATTTCGCCTTCATGGTTTACACCAATTTCAAATTTGCAAGTAGTCGGTATACGCATTCCAGCAATTTTCATATTCGTTTGTAAAGGCAAAATGAACCTGCATGGTTTGCCTTGCAAATGTGACACTAAGGATGCTGCACACGCTAACTGAGAAGGACGTGTTATTTTTCCACCATATCCACCACCTACTCGACGTACGATTACGTTAATCCTGTTAATAGGAATGTTTAAACATTCTGCAACAGCTATATTAGCCATATCCATCCATTGAGTAGCTAA

>SinsAOX7

TCCATCTGGTAGTTCCAGAGATTTGCGCGTTCATACTAACAAACAAACTCTTCAGCTATATATATAAGTTATCATTTCAATTTAAAATGTTCCAGTTTGTGTCCGACTGCTGTAAAGATATTCTCCACTGTGCACGGATAATTTATGTCCACCCATTGATCGTCATATCCAGCATCCAGTCGGGCTGCACGCAGCGCTTCACGGAACGCATGAGTTATTACAGACGCCATGGCGAGAGCTGGCTCTCCAGTAGCTTTAGATTGCAGTACTCCAAAAGGGTTTGTAGAGTTTCTCCTGAAATAAACTCTCAAGTCGGCCGGGATATCTTTGATGCCAGGAGGTTTGTAAGTCCAAGTACGATCAGTGAGCATTTTTCCACTTTCAGCATCATATAAGGTCTTCTCAGATGTCCAATAACCAAGACCCATGATAAAGGCACCCTCGATCTGAGCTACGTCGATTATTGGACTTAAACTTCTTCCAGTATCTTCGAGTAAATCTACTCTTCTGACATCATGATTTCCACTCAAAATATCAACTTCAACTTCTAAAGCACAAGTACCACAAATGTCATATGGTTTTACACCATCATTATTGGAATACATGTACGAAGCTTGCAAATCAATTCCAGCATTATACGCTTTACTGATCAGGTCTTCCCAAGAAGGCTTTCCCATTTTTTCCTTAATAGGTTCCAATCTTTTTAGAAGTATTTCACAAGCTTTCATAGTGGCGAAGGCGATGCACTCACTTCCTACACTGGCACCAGTACACATTGTATTTGGTGAAGTAAAACTTGAGCTTGGCTTAACACTAATTTTCTCCAAAGGTGCACCAAGCATGTAAGCACAAACTTGAGCTGCCTTGGTATTGATACCTTGTCCCATTTCTACACCACCATGTATTATTGCTATAGAACCATCGATATGGAATACTGAAATTATAGAATTGTAATTTCCAATATAAAATAAGTCGTATGTCATCGGAATTAGTTTAAGTGCTCGTTTCCGCCATCGATTGTTGTCGTTGAATTTTTGAACTTCCACTAAGCGATCTTCATAATTTGAATCTTTTTTCAATTGTTCTATTAGTTCTGGTATTGGATTGTTCTCTTTAGCCATATTTAGTAGTCTAACTTGCAAAGGATCTTTACCTAAGGTAAAAGCTATTCTTTCCATTATGTATTCTATCATAGCAATCCCTTCAGTTGAAGATGGAGCTCGGCACCAAGTATTCGATGGTGTATCGGTAGCAACACTATTAGCTTCAACATACCATCTCTTGCTATCGTAACAGTTGAAGAAATGATTAACAGTTATAGGACTGATGGTTTCATTTGGCGAATAACCATTGTCTTGATAGAAAGTATTCTTGAGGTATTGAATTTCACCTTCTTTGTTCACACCAACCTCAAACTTGCAGTTAGTTGGTATTCTTTTTCCTGTCATCCTCAAATTTATTTCTATCGGCACTACAAACCTGCATGTTCTGCCCAATAAATGTGTCACAAGTGCCGCAGCGCAAGCAGTTTGACAAGATCTAGTTATTTTACCGCCATAAGAACCTCCAACACGACGAATTACGATGTTGATACTATTCGCAGGCACCTTCAAGCACTCTGCAATAGCCACATTCGGCAAGTCTAGCCATTGAGAAGCCGTATAAACTTCCATTCCATCTTCAGTGGGTTTCGCAACAGTTGTCTGCGGTTCCATATAAAAATGAAACTGTGTATCAAATTTAAGCTCTCCATGTAGAACACTCTTGACATCATTCCCAATTTCCGTTGGTTCAACTGTCCTATTATTTGTCGTTCTTTTATCTTTTTCAGGCGATTTCAGCACATCTTCCACTGTTAATATGGGTTTGTTTTTACTTATAGAAGCGTAATTCACCTTTACAAGACTAGCTGCTTTAATAGCTATCTTTTCTCTTTTAGCAACAATGATTCCACACGGTTCTCCATAGTATTTTACTTGATGAGAACATAAAATCTCTTCATCGGCAACTATTAGTGGAACCTTTGGAGGTGTGAACGAGTTAACACCTGGTATATCTTTGGCTGTATAAAATGCTACAACGCCTGGAACTTTAAGAGCTTGAGAGGGATCAAATCCACTAATTATACTTCCTGGCTTAACATTAGCACAAACATAAGCTCCAAAAACTTCATCAGTTTGTGTGGGTAAA

>SinsAOX8

AAATAAAAGGTTAATATACATATTATATATTTAAATAAATATGTTAAAATATTTAATTATCTAGTGGGAAAAAGAACGAATAATTAAATTCTATTCTGTAACAATGAACTCCCCCAGCTTCACGTCCAGAGCCTTCAATATGGATTCTGTGTCGTACGGAATATCAATATTAATCCATTGCTCAGTGGCATATCCGGAATCCTTTCTAGACTCCAATATGCATTGGCGTAGGGCATGTGTGATTCCGTGTGCAGTACATAGGCCCATTTCACCAACGGCTTTAGAACTTAGAACTCCGTTGGGATTCTTCGAGTTATATCTGAGTTTGACCCTAAAGTCGATCGGAATGTCCAATGCTAATGGCACATGATAATTTAGCGAGCGATTAGTTAAGAGCTTGCCAGTATCTTTGTCAAAGACGAGTTTTTCTGTAGTGAAGTAACCAATACCTTGAATATAGCCACCTTCAACCTGCCCAACATCTACGCTGGGATTAGCGCTGAGGCCAACATCTTCTAAAATATCAGCTCTACGCATCTCGTACCTTCCAGTTAGAACATCCAATTCGACTTCTAAAATAGCTACAGCAAAAGCAGTGTATGGTTTAAGATCTTCCTCTGTATCTTTCATCATATATGTAGCTGTCAAATCGACTTGTTCATCGCCAGCTTTCTTGACCAACTCTCTCCAAGTTGGATTTGTCATTTTCTCTTTAACATCCTTCAATCTCTCTTTTAACGTAGCACACGCTTTGATTATGGAGTAACAAACACTTTCACTTGTAATACTCGACCCCGAGAACACATTATTCGCTGCCACAAATGAATAGTTGGGTATAACTGATATATATTCTAGAGGGATACCCAATTCGTAGGCACAGACTTGAGCAGCTTTAGTATTTAATCCTTGACCCATTTCAACACCTCCAGTGCTAACAGTCACTGTACCATCACCTCTGTAAATGGAGACCATAGCACTATAGTTCCCATAATACACTACTGGGAAGCCCATGATGTTAATTTTAATGGCTCTCTTTTTCCATCTGTTTGCTTGGTTATATTGTTCTATCTCTTTTACTCGTTTGTCGTAATCTGTGTCCTTTTTGAAATCTTCTATGAGAGATGGTAAGTCGTTGTCATCTTTTCTCATATTTGCTAACCGTACGGCGGTTGCGTCTTTCTTTATTTCAAACGCTATATGCTCCATAATGTGTTCTATAGAAGCCATGCCTTCACATGTTCCTGGAGATCTGGCAAATGTATTTGATGGCAAATCAGTGAGCAAATTAGCAGTTTTCACTGAAAAATAATCCGTGTTGTAGCAGTTCTTGAAACCTTCTATGGTGTAACTCAAAATATCTTCATTATTCGAGCTGCCGTTGTCCTCGAGTATTGTTGCGGTAAGATATTGAATCTTGCCATCATTGTCGACGCCCACCTCATATTCACATTGGCACGGTAGCCTTTTCCCTGTTATCGTCAGATTGGTCTGCAGCGGCAGTATGAAGCGACAAGGCACGCCCTGTTTCTTGGCGACGAGGGCACACGCAGTCGCCACTTGGGAGCTGCGACTGATCTTGCCGCCAAAACCTCCGCCTATACGACGGACCATCAAATGGACATCGCTTTCTTTCATATCCAAACAGGTGGCTATAGCTATCTGGGTCAGATCCATCCATTGAGTCGAATTGTACACCTCAAGACCTTTGTCCACTGGCACAACAACACACGTCATCGTCTCCATATAATAGTGATACTGAGATTCTATCTCATATATACCTTTTATTACTTTCTTAACATTATCTCCTTTGCCTTTAGACTCAATTGAAGCATCACCAGCTACGTATCTCTTACTATCTTTCTTAGCTTGATCTATTGTTAAGACCGGTGGCGAGGAGCTAACGTTTTTGTAAGTTACTTTAACTTTCCTAGCAACGGAAGCCGCTAATTCTTCTGTATGTGCTACGACTATTGCTACCGGTTGACCGTAGAATTTCACATTCGAGCTAGCTATTATTTCTTCTTCTACGATCTGCAGTTGAATTCCTGGTACAGTGAATGTATTTTTTCCTGGTATGTCTTTTGCTGTGTATACAGCTAAGACGCCTTCTATTTTCAGCACTTCAGTTGTGTCAATATGATCGATTTCGCCTACGTGTATAGTCGATAACACAAACGCTCCAAATACTTCACGAGGAAGCCATGGGGTGTCGTTCGCGTATTGAGCTTCGCCGGCACTTTGGATCAACGCTTCCAATTTTTGAACTGGCTGGTTAAGGGGATAGAGTGACGAGTCCGTTTGGAAGTCTTGACTTCCACGAGAAACTGGCCGTTCTATTAATGTGGCCCCGGAACGTAAACGAGGACTTGTCACACCGGCTGGGCTGATGCTGAGGATGAACTTATAAAATAGTCCAATAGCAAGTTTCTTCCTGCATCCAGATGATGGTTGCGGTGGATTCTCTTTCGGCACCAGTTCATCGCTCAACACCCTGATAGTATCCTGCAGCGTCTGATTATTGAATATGTTTTTACCTCGCAAATAATTCTCAGTTCGTGTGGCGTGTACAAAATCTGGAGATATATTGCCGTATACTATACTAGCATGGTCTATTGTTTTTGTCTTGTAATTCCACTTTATAAGGTAACCAGCGTTCACTATGGCTAATGCGTTTTGACTCCTCGGCATTATCTTATAACTGTTGAAAATATACGAATTGTTCAATGGTGGCAATTCAAAATTTACTATCAGCAATCCTTGCATGTTACGTTTCAGGAAATCTGTCAATGAGATTGTTGTTCTTCGACCCTGACTGTTACGTATTGTAACCATGGCACCAATAGCTTCGAACAGAATGAAGACGTCAGACTGATATGCGGGCATCGCGTGTTTAAACATCATGTTACCAGCCAGCGATCCAAGCTTTCTTACTGGTACATGAGCAATCAGCTCAAAATGGTTAGCGAACTGAGACAAATAGTTGAAGTCACTGTTCGTCTTGGCTGTATCTCTGAATAACTTTATACAATCTTCTAAAGAAATGTTAGCTCCGAGTATCAAATTCTGATCGAAACTGTATGTCTTCAAAGATTTCACATCACTTATATCTATAAGGACACGCGGGTACTCGAATACTTCCACTATTCCTTTACCAGTATTTCCATCGATAAGCATATAAGAATCAACACCATTTTTGTTTAAAATATCAAAAATTTCTTGTTCATGGTATACTTTAAAAAAATGATTTTTTCCAAAATTTAAAGCTTTCACTTTGTCGTCAGTAGCTTTGACTAGATCATTAACGATAATCCAATCACTATTATCAGTTTTAGTAGAACACTTTCTCTCGCAAATCTTTTGTGTTTTACTGCAAATTTTTAGATCTTCTATATCTTTTACCCTTTGACATAGTTCAGGACTTGCATCTACTGCAAAAGACTTGATAGTGTCTAAAATAGGTCTGTAACCTGTACATCTGCATGTGTTACTGCCGAACGATTTTTCTAATTCAGCCATTGTCATGTGCTTATCTTGTAAACTGTTCAGATGCATCACCCATCCAGGTGTGCAGTACCCGCATTGGGTGCCATTGAAAGCGGAAATGCGTTTTTGAATCTCACTGTAGCCGTCTGTGCGGTTGCCAACGCCTTCTACGGTA

>SinsAOX9

AATATCCCAATGGTGGCACGACAATATGTGTACTAGACACGAGTTAACGGCAAAAACTCTTTTCTCCTTAGTTACTGGATGTGTTTGCGACACGGATACGACACAAGCACCGCACCCTCCCTCGTGACACATCGCTTTAGTGCCGTGCAAGTGCAGGTAGTTGCGCAAATAGTCATTCAGTGACGTGTCTGTACCAACCTCCGAGCCAGATAATGTATAATGCTCTCCATTTACTGTAAAGTATACTTTATCCATTTTATTTAATTTAGATCAAATATTATACTATTGTATTTTTTAAATTATAACTTCACTACACAAAGTTTCTGTTATATATTGTAATGCCTTTTGATTGACTCTATATATTAATGACGTTAATAATTATAGCCAATGCTAAAAGGAAAAAGAAAAATCTCAATTTTGTAGATTGTAAATCGATCATTGAACTTTCGCGCTGTAAAATAA

>SinsAOX10

GACTTATAATGACTAATACATTGTATCGCGACCGCTAGATACCTCATTAAGGACTATAAAGAGTTACACAACTATATTTTTTATCATTATATCATTAAATCTCAGTAAAATATGACCATAAAATTGTAATAAATGTGTTTTATGTATAAGTTTGAAATTTAAATAGTAATCTCAAAAGTAATTTAGCAATCTCGTAACTCGTGAGAAGGAAAATGTCCAAAATTGTTTTTAAAATTAATAACAAACAGTATGAAGTGGACGGTAGTTTCGGTCCGGACGTGTCGCTGAACGAGTACATCCGGTCGACGGCGGATCTGCGCGGCACCAAGGCGATGTGCCACGAGGGCGGGTGCGGCGCGTGCGTTGTCGCCGTGCGCGCCGCGCTGCCGCCCACCAACCAAATGAAGATATTTGCCGTTAACTCCTGCCTAGTGTCGGTACTATCATGTCACGGCTGGGAGGTGATTACCGTTGAGGGTATCGGCAACCGCACGCAGGGTTACCACGAGATCCAGAGCCGACTCGCCAAATTCAACGGCACGCAATGCGGATACTGCACGCCGGGTTGGGTTATGAATATGTACAGTCTCTATAAAGGGAAAAACAAGAAATTGACTATGAGTGAAGTAGAAAACTCTTTTGCAAGTAATATTTGCAGATGTACAGGATATAGACCAATAGCCGATGCGTTCAAGAGTTTAGCAAATGATGCTGACAAGGAACTCCTCGATAAAGTGTGCGATTTAGAAGATTTGTCTACGTTGAAATCTTGTGGTGTCAATTGTGCTAAAAAATGTACTCACAAAGATACCAAAATTAAATGTAAAGATAAAAAGGAAAAGATTAATGTAGACGAATGGTATGTTCTTGAATCTAATGAGAAAATGGTTTCAATAGACTGTGAAAATCATAAATGGTACAAAACATATACTTTAGAAGATGTTTTTAAAATTATTAGCAATAATGACAGTTATAAATTAATAGCTGGTAACACAGGGCAAGGAGTATACCACGTCACTGAATATCCGCAACATGTCGTAGATATTTTCAATGTGCAAGAATTAAAAGGATATTTATTGGATGTCAACTTGGTACTGGGTGCTGGAATGACTCTAACAGATATGATGGACCTTTTCCTAACACTCTCAATGGAAAATAGCGATTTTTCCTACTTAAAAGAATTTTATGATCACATTGATTTAGTTGCTCATATTCCAGTGAGAAATATAGGTACCATAGGTGGTAATTTATATTTAAAATATGTAAACAATGAATTTCAATCGGATTTATTCTTGTTATTTGAAACTGTTGGAGCTGTTTTAACTATTGCTGAAGACTATAATAAAAATGTACAAAAGTCACTGCCTGAATTTCTCAAAATTAATATGAAAGGCAAAATAATTAGAAATATTATGCTTCCACCACTACCTCTTTCTTGTTCCATTAAAACATACAAGATAATGCCTCGTGCTCAAAATGCACATGCCGTTGTAAATGCCGGTTTCCTGTTTCAATTTAGTCATAACTCACACATATTAGAAAAGGCGACTATAGTTTATGGTGGTATTTCATCCGCATTCATTCATGCTATTAAAACAGAAACAATTTTAGTGGGTAAAGATCCATACACAAACGATACCCTGCAACTAGCTCTACAAACTTTAAATGAGGAAATAACTCCAGAAGAAGTGCTACCAGAACCTTCTGTTGCTTATAGAAAAATGCTAGCAATAACTTTGTATTACAAGGCAATACTAGTGCTTTGTCCTGATGATAAAATGAATCCTAAATACCGTTCAGGAGGCAAAGCTATGAGACGTAATATATCCAAAGGTACGCAGACATTTGATACAGATAAGAGTATATGGCCTTTGAATCAACCGGTGCCAAAATTGGAAGCACTAGTGCAATGTAGTGGAGAAGCGACATTTGCGAATGATTTGCCGAAACAAGCCAATGAATTGTATGCAACATTTGTGTGTGCTGACGTTCCTGCAGGCAGTAAAATTGAGAGCTTTGATGCATCTGAAGCTCTTAAAATGACAGGTGTTGCTGCATTTTACACAGCTAAGGATATACCGGGAAGCAATACATTTACACCTAAAAGCGTACCATTTATGACAGCTAATGAAGAAATATTGTGTTCTGAAGTAGTCAAGTTTTACAGTCAACCAGTAGGAATTATAGTTGCCGATAGAGAAAAAACAGCAAACAAAGCTGCGAAACTAGTTAAAATAAAATACACAGCAACTACAAACAAACCTTTATTAACAATAGATGATGTAATTACATCTCCAGAAAAAGACAAAAGATTAGTAATTGATAAAGTGGTTGAACCAACTGATATTGGAAACGACGTTAAACATGTAGTCAATGGAGATTTTAAAATGGACACTCAATATCATTATTATATGGAGACACAAACTTGCGTCGTGAAACCAACTGAAGATGGAATGGAGGTTTATTCTTCCACTCAGTGGCTTGATTTAACTAATGTAGCTGTTGCAGAGTGTCTAAATATTTCTGTCAATATGGTAAATGTAATTGTTCGACGAGTTGGTGGAGGTTATGGTGGAAAGATATCTCGTGCTACACAAATTGCATGCGCTGCTGCAATAGTTTCACATTTGCAAGGCAAAACTTGTCGATTCATACTGCCTTTAGAGACCAATATGAGAATAATCGGGAAAAGATTACCAACAAAATCTAACTTTGAAATAGGAGTTAACCAAGAAGGTGAAATACAATATTTAAAAAATAAGTTTTACCAAGACTGTGGATGTTCTTTCAATGAAACGATGTCTCCAATTACTCTTGGACATTTCCGCAACTGCTATGATTCTAAGAGATGGTATATTCAGGCTAATTCTGTAGCTACCGATCTACCTTCTAATACTTGGTGTCGAGCTCCTTGTTCGACTGAGGGTGTGGCAATGATAGAATATATGATGGAAAGGATAGCATTCGATTTACACAAAGATCCTATACAAGTCAGATTATTAAATATGATCAAAGAAAATAATCCAATTCCCGAAATGATAGAACAATTTAAAAAAGATGTCAGCTATGATGAGAGAATGGCAAAAATAAAAATATTTAATGAAGAAAACAGATGGAGAAAGAGGGCTATGAAGTTGCTTCCATTGACTTTTGACATATTTTATATGGGAAATTTTAATTCGATTGTTTCAATTTATCATACAGATGGTTCTGTAGTCATAACTCATGGCGGAATTGAAATGGGTCAAGGTATCAACACAAAAGCAGCTCAAGTTTGTGCCTATATGCTTGGTGTACCACTAGAAAAAGTTAGCGTCAAACCAAGTTCAAGTGTAACATCACCAAATGCTATAGTTACTGGAGGTAGTATTGGTAGTGAATGTGTTGCTTTCGCTACTATGAAAGCCTGTGAAATAATATTAAAGAGATTAGAACCTATTCGAAAAAATATGAGTAAACCGTCATGGGAAGAAGTTATTATAGAGGCATATAAAACCGGATTAGATTTGCAAGCTTCTTATATGTATTCTAATGTCCATGATAATTTGAAAACATATGAAGTGTATGCTGTATGTGTATTAGAGCTTGAAGTCGACATTTTAACAGGCAATCTTGATATTAGAAGAGTAGATCTGCTTGAAGACACTGGAAGGAGTTTGAATCCAGAAATTGATGTTGCACAAATAGAAGGTGCTTTTGTCATGGGGTTGGGTTACTGGACATCAGAAAAATTAGTGTATAATGAAAAAACTGGCGAATTACTAACGGATCGAACCTGGACCTACAAACCACCTGGTATTAAAGATATTCCTGCTGATATGAGAATTTACTTCAGAAGAAATGGTAGAAATGAGTATGGAGTATTGCAATCGAAAGCTACAGGTGAACCAGCATCTTGTCTAGCTACAGTACTAATACACGCAGTGAGGGAGGCGTTACGGTCTGCGCGTTTAGATGCAGGATATGAAGATAAATGGTTCGATATTGAGAATCCGTGTACAGTTGAGAACATCTACTCGGCTGTTGGACACAAACTCGAACATTTTAAACTAAAATAAATATCTTATTCTAAAATATATAACATCAACCTATATACATACTTTCCACTGTTGTACGGGTCTTACCTCCAATTATGAGAGGCCGGACCCTCTGGCGGAGTGTGGTCCGTAGGACATACATACATAAAAAATTATTATTTTATATTTTAATATTTTTTTCTAGAATTAAAAAAACAACATTTGGCAATTATTACTATTTTTTAAACAATTTATAATACTTCTGAATATACTGAATTTTTCTTTCTATATAATTGCATGTCAGTTTGAGTTAGTTACATATGGTTTGTATCAAGAATTTTTAGTTATAATATTTTTTAAGACATCGTACAAGTAGCCTTTAATAATGTAGCCTTTAACGATGAAACAATTTTTGGAATTGGTCTAGTAATTTTCGATTTTACATGATACATGCAAATATATTTTTTCTCTTTATATATTAGTATAGATAATATAAACATTAGTTTGAATATAAAAACTAGGTTTAAAAAACAAATATTTTAATCTAAATACTTAAAAAATTAATCCCTGCGGGAATGATCCCAAAATGCTGGCAACATTCCTCGTTGCATTACGTCGCTGCTGATGCGTTGAGCGAGGAATATGGCAGCCAGGGTCACCAGATACGTAGACAATTCTTGCGTATGTATATTATATTATATCATAGATTAGATAAAAGTTTTGAATAAATAAACGTTTTATGTAATTTAAAAAAAAAA

>SinsAD1

GTCCGACGTCTAATGGTCCTTGGGTGGAATGGAAAAACTTATTTTTTGAAATGTCTTTATCATAGTTTCCTTCTGAGTACTTGAAATATTGAAGTATATGGTCGAAACTCCAACCCTGGTTACCGTTAAACGCCCACTCGTCATAATCCGCAGGGCTGCCTCTCATGTATGCCATATCGTTAATTGATGAGGTTCCTCCAAGAACCCTTCCTTGGATAACTTCGCATCCTAATTCCATTCGATACTGACAAAACCTAAAAT

>SinsAD2

AGTATTCGATTGAACGTCGCATAAGACGAATCTAGTCGCACCGGCCCCACTAAGAACACATTAAAACAAAATGGCCCAAGGACTGAAAAACAAAGTCGTCGTCATCACTGGTGGAGCCGTCGGCATCGGTCTCGAGATCGCCGACAAATTCCTGCAGAACTTCGCGAAGGTGGCCGTTCTTCTAGACATCAACGAGGTCCAAGGCGCTGAAGCTGTCAAGACCCTAACCAGCAAACACGGCAGCGGAAAAGCAGTATTCTACAAAGCTGACGTCACTTCAGATTTGGACAAAATCTACAATACTGTAGTAGGCACATACAAGAACGTAGATGTCCTTGTCAACAACGCCGGCATACTCGACGATACTAAAGACAAGTTAACTATCGATATCAACGTCACAGCTCTCATCCTGTGGTCCATGAAGTACTGGGAGCACATGAGGAAGGATAAGGGTGGCAACGGCGGCACCATCATCAACTTGGCTTCGATTTACGGCTTCAGGGTCGACCAGTATCTGCCAATTTACCAGGCTTCGAAGTTCGCAGTTTTCGGCTTCACCAAATCTCTGGGACACGTTTACAATTACAACAGAACCGGTGTGAAGGTAGTAGCAATTTGCCCCGGATTTACCGAGACGAAGTTGACAGCGTCTCCGAAAACGTGGCCCGATCAGCAACTACAGGCAGACTTCACCAAGTTCGTGGCGCAACAAGCTTGGCAAAAGGTCGACGCTGTCGGCAACGCCGCTGTAGAGATTTATGGAAAGCCCAGCGGTACCGCCTGGCTGATCGAAGGTGCCAAACCCATCGTCGAGGTCTGAATTGGTGAGGTAATGTTTCAGAAAGTATTTGTATATAATTGTTGAAGCGGACTGTCTGATTAAAAAAAAAATCTTGTGCCATGACTTGTAATTTGTATTACAAGTCGATATAGTAAACTATTGTACTTTGTTGAAACCGATGGAAACAATTGTCAAATAATTAATATTGAATTATTATTAACTTTACATTCCGTCTCCAGAAACAGTAGTAAGTGTGGTTACCAAATATTACAGGAAAGTCCATTTATGTATGGAATGAATGTAATTAATTATAAAGACTTGAATTTTTCTAATAATTTTTTTTACTCTATATTGAGGCTTTAATGTTTCTTTTAATTACATAATGTTTTTATATAAAATGTAATAGCATTTAATGTATATTTTTTGTATTCACCTA

>SinsAD3

GCATCGCGTCGGAAAGGACGGCCGCCCGCGCCGCTTGGTGCCATTGTGGTTTTATTCGCACAACACTAGACATCTTAAATTGTTTAATTTCATTTGTACTGCTTTTTATGTGAAATAAATTTTAAAATTAATTACCAAATAATGTCCGTACCGGAGTACTTTTTGTTGGAAGATGGGGATCGCCTACCTCGAATTGGTTTTGGCACTTGGCAGGCATCCGACGAAGTTCTGGAAAAGGCAGTGGATACTGCACTGGCGTGTGGATACAGACATTTCGACAGTGCGCAAGCTTATGAAAACGAGGCCGCCTTAGGCCGCGCGCTCCAGCGCTGGATAGGAGGCGATGCGGCTAAAAGGAAAGAGCTATTCGTTGTTACAAAACTGCCTCCTGGAGGTAATCATCCAGAACTAGTTGAAGAATATTTCGAGACGTCCCTAAGAGATTTAGGTCTGGATTACTTAGATCTCTATCTGATACATGTGCCGTTCGCCTTCGAACACATTCCTGGCGATCTACATCCGAAAAACTCTGATGGCACTATGAAGATGGATACCAGTACAGACCTCGTTGCTGTTTGGAAGGCCGTGCTAAAATTAAAGGAATCGGGCCGTGTGCGACACGTTGGAGTGTCGAATATGAATGAAGAACAAATTTCTCGTCTCTGCAAAGTGTCAAAACCGGAGTGTCTTCAGATAGAAGTGCACGCGTTATGTCAGCAGGTCCCATTGATCGCCACAGCGAACAAATTTAACATACCGGTAGTTGCATATTCGCCTTTGGGTTCCAAGGCTCTCGCTGACGCTTTAGCTGCTAAAACTGGACGCGAATATCCTGATTTGTTGCAACTGCCCACCGTGCAGCGTATCGCCGAGGCGCACGGACGTACTCCTGCGCAGATACTACTTCGACAACTATTGCAACGCGGTGTCGCTGCCATTCCGAAAAGCACGAATCCTGAACGCATCAAGCAGAATATATCAGTATGGGACTTTCAGCTCAGCGATGGGGAGATGGCTGAACTAGCTGCACTCGATCGCGGGGAACGCGGACGCATCTGTGACTTTTCTTTCTTCATGGGAATTGAGAAACATCCCGAGTTCCCTTTTAAAAAATAAACATTGTTTCATTATTAAAGAACATAAAAAATAACTCTTATATTTGTGCAAAATTTATAAATAAAATATGTTCTTGAATGTGATACCAAAAAA

>SinsAD4

GTCAATAGATGGCGGCGTCACGAAGTCCCCAACGAAAAGTTTCACTTTTAACGCACGTTTACGTTAAGAAAGTATAACATCTAACGCGTGTACATAAGTAGATTTATTGACCGATTTCAATGCAGTTTTCAAAAAAAATAACAGGGCTTAACTTGAAGCCACATGTTACATAAATAAAAAAACGAGCATATTTTTTATTGAATTTTAGGAAACCTATTGTTGACGGACACATATTATAAGGGTATATTACTGGTCTACGGAAAAACTAAAAAAAGCTTGAAAAATCAAGCTTTCTTTTCGAACGGATTGGGTAATGGATTCTCGATTTTCTCGAACGGGTAATAAGGATGGTTTTGCCAGAACGATGGCAATGTGTATCTTATTTGCTTATCATATTTGTTAATCTTTTCAATTTCATCTTTATCCAACTTGAAATCGAAGATGTCGATGTTTTCTTCCAAACGCTTGAGGTTCACCGTTTTCGGTACAGGCACGATGTCTCTGTCCACCAACCACCTTAACACGACTTGAGGTGTGGTCTTCCCGTGCTTCTTGGCAATTTCCACCAGTGTTGGGTCATCTATACTTGGCCCCGAACCAGGTGGTACTCCGAACCTCGATACAAGAGAGCCGAAGGGACTGTAACCCATGACCACTATCCCTTGAGACTGGGCGTAGGAGACCAGATCCGACTGGATTATTTGTGGATGGACCTCAATCTGCAGAACGGCGGGTTTGATGCTGCATTCTTTGATCACTCTTTCCAATTGTTCCTGGTTGAAATTGGACAGGCCTATGGACTTGGCGAGGCCCAGTCGTTGCATGTCTTCCAATCCACGCCACGTCTCCATGAAGTCCACGTCGGAGTTCGAGTAGTCTTCATTGAGTCCAATAGGCCAATGCATCAGGTAAAGATCGACATAATTCAGACTAAGTTGCTTCAGCGACTCGCGCAATGCTTCGGCGACCTGCTCCCGCTTGTGGTGGGTGTTCCATAGCTTAGTAGTTATAAATAAATCCTCTCTAATGACTTTGCCCTCTTCAATTTTGTTCCTCACGCCCTCGCCCAACTGTTCCTCAGTGGAATACACGGAGGCCGTGTCGAAGTGTCTATAGCCGACGTCGATGGCGTGTTCCACCACTTTCACCACTTGCTTGTCTTGCGGTTTCACTATGCCGTTCTGGTCAAAGCCCAAATAAGTGCCCAATGCAATAGCCGGCATTTCTTTTCCGTTATTTAATTTGAATGTTGGCACGTCCGGCGCACAGTTCGCCAACTGAACGCACAGCGCCGCAACCACTGACACCAGACGTACACCTCTCGTCATGTTTGAAGTTCAAGTAACGCTTGAGAACTCTA

>SinsAD5

AAATCATTAAAATCGGTTCACAACCAGCAAAGATATTACGTGACATACACGAAAAAAAAAACCTGCCGAATTGAGAACTTCTTCCCATTTGAAGTCGGTTAAGTATTGTGTTTTAAGTTATGCAGAAACGTTTACGTATGAACTGAAAATAGTGTATTTGAAAAATCAACTCCCATATTTAACGGATCACCTGAACGTATTGTAGACGCATGATATTGTGGTACGTGGAGTGTGGGTTCTAAGGAAAGCGAATAACTGTGGTTCCGATGGAGTCGTCGCTACACGCGTGCGCCAGCGCCGTCTCGAAGTCTTGGGGGAGATACACTTTATCGAGTACCGGCACGAGCTGTCCAGACTCGACCAACTGTGTAAGCTGTGCCAACCCTCCGGCGAGCCGAGCCGGGTCCTCGAGCCAGTCCGTGTGGCTGCCGAAACCAGTCACCCACCTTAACAGTCGGAAGGTGTAGAAAGAGGCGGCGAAGACGAATGTGAGTGGCGTGGGGAGGCGATCCGATATAAGCGCGCGCGGCCTCAATTCCACTATAGATTTGCGGGATGCTGTCGCTTTGAGCAAAGCGGCCGCGTTGTACGGGTGTCCGGGCGGGACCCCGGCGCCGGCACAAGACACGACGCAGTCCCAGGGCCCGGCGCGTGCTGCGTGAGCCTCCACCGCTTGCCATGAGTACGACGAATGACTGTTTGTGCCCTCAGCCTCAATGTAGTCCTGGGCGCCTAGATCCTTAAGTATGAAGACGGCCTTCCTGGGCGCCAGCACGGTGACGTGCGCCCGCCATGAGCTCAGCAGCTGCACCAGCGCGCAGCCCTCGCCGCTGCCGGCGCCGCAGATCGCTACGCGCTTGCCCTTGCTGGTGTCAGACGTATAGCGCAAGTCGCGCAGAGTAGCGAGCGCGAGAGTGCCGCCCCACGGGAGCGACGCTGCGCTGTCTGCGCTTAAGGACAATGGTCGTTTGCTTACACGGGTACTGCGCACAGTTAGCAGCTCGGCGGCAGCGCCACCTGCCCACTCGCTGACGCAGCCCCACACCTCGTCACCCAGTTCCAGATTTGTCACGCCGGGCCCTACATCCAACACTACCCCAGCAAATCCCCGTCCAACTGTGAGGTGTGTGCGCGTAATGAGTCCTCGTAAAGTGGCCGCGTGACCGCGCAGCACACCCCTGTCTATCGGACAGACACTGAACGCTTGAACTCGAATTAAAACCTCGCCAACACCCGCACCTGGTGTAATAGCGTCATCGACCAACATCACGGTCTGGTCCGGGTGAGAGTGTAACGCCCGAGCGTGCGGGCCGGTGGGTGCTCTGGCCGCGACCCGCAAGCCTGCCGCTAGTCCCGCAGCGCTACCCAACACCGCGCCCATGGCGAATATCATCACGCTGCGACGCCATACTCTATCCTTAAGCAGTGCGACAAGTTCCTCATATAAGACCATCGGAGAGAGTGGTGGAGCCCCTTCCCTGATTCTCTGAGCTGCCTCGTGCGCCCATGCTGAAACGCGAGCCCGCCCCTCCGTCATCAACTCATGGTGCCATACCTCTTGAAATGCTTCTCGGAGCTTTCTGATAGCTTCAATTGTCTGGCTAAAGACATCTTGAACCCGAGATTTGCTGCTCCCTGCAGCCGTTACAGCTGCTTCATGTAATGCACCGAGTTTTTCACCAGCTCTGTATTTAAATTCGTCCATGTCAAAGCACCACGGGACGTTAGAGGGTCGCGCTAAACCATATGAATGGTTAAATGCATCTAAACCTTAACCTTTGGACATAAATCGGCCTAAATAACTGCAAGCTCAAAATAGACCGACGGGTCATCAATATTAACCAATGACAAAAGTTTTATGGTCGAAATTTTGAAAAAAAAGTGTCTTACTATCTAATTTATTAGCATGTTTATCTCGAAATACTCGATAATATTGTGTAAAAGTGTATAATTACTACCTTATTCAAACATAACCTTTTTGTTTTTTTGACAATGAG

>SinsAD6

AATGTCAATCCTTCAAGAATTTCAAAATATTCAAATTAATAAATAATTACTTTTAACTAATAGTTTACTACATATAGATTCGAAGATTTTACACCATAAACAACCTATGTCATGTGAATTTTCCGTTTTCCGTGCACGTTTCAACTTTTAAATCACATAAAAAAACATCGATTAAAGCAATGAGAAAACTTCGTAAGTATACAGTTAGGAATGCATTCAAAGGTTTCTTACCAAAACGGTCGGACTTCGAAACAGTCGAGGAACAGATCCCGACACTCCAAAATGGTGAGTTTCTCATCAAAACCGAGTACATAAGCGTCGATCCATATATGCGTTCGTTCGCTAACGGACGTCGGACACCATACGACCAATTCGGCTTTCAAGTCGGAAAAGTTATAGAGAGCAAAAACTCCGAATACCCTACAGGCGTATACGTAGTCTCTCACTCGGGTTGGCGAGATTTTGCCGTTTTGAATGGCGAACCAGACGATATGTTCAATATGAAACCTTACCAGCCTCCGATCGGAGATTTACCCATATCTATTTCATTGGGAGCTCTTGGGATGCCAGGAATGACTGCATATTTAGGCTTACTAGAGATATGCAAGCCGAAAAGAAATAATATTATTTGTGTAACGAGTGCAGCTGGTGCAGTTGGCTTGCTAGTCGGACAAATAGCTAGATTAAAAGGGTGCACAGTCATCGGATTCACTGGTTCAGATGAAAAGGTACATATCCTAAAGCAAGAATATGGCTTCCACCATGTATTTAACTATAAAAGTACTACAAATATACGAGATACATTAAAATTGATCGCTCCGAGTGGTATAAATTGTTTTTTTGATAATGTAGGTGGAGAATTATCAGCGGCAATTATGGATTGTATGCAGGAAAACGGTCAAGTAGCCATTTGTGGGTCTATCAGCAATTATGGTGACTCGTCATCGCATCATATAAAACAAAAATATCACTATAAAATTGAAGCGTTCAGTTTCACACAGTGGGAATGGTCTGAGCAAAGCCATGCATTGACACAACTTAAGATCTGGATTGACAAAGGAGACATCAAAGTAAAGGAAACCGTTACAGATGGCTTTGAAAAACTTCCACATGCCTTTGTGGGTATGTTGAAAGGGGAGAATGTAGGTAAAGCTGTTGTTAAAATTTGATTTCTATTCCCTTACTGATTGGGTATAAGTTTCATTACATTGCAACTGAATGTTGTAATACCTAAGAAGAATTAAAAAACATGAAAAGCTCTCAAAGAAAAGATTTTTCTTTATTTGTTTTGCCTAAAGGCTATGTGTCCATTGTTCATTTCTGAGGAAGTTAATGATTGAAAGCGAGAAAAGCTTCCTTTGAGTGTAGTCCATTTTGTAATAATTTACATTTTAGATTACATTGTTTATTTTCTGAGCTTAGAGTAAACAATACCAGAGTTGAATTAATTACCGTAGCAACTTCTGTACAATTTGTGTGTAGAAATAGACTTAAACTATTTATGTTTTTGTTATTTTGCATTAAAACGGAACACTTAATTCATTTATTCACTTACTCCTTACAAGTCTACATAAATATCTGAATGAAATAAGTAAACTTTAAAAAACAAATCTCCTA

>SinsAD7

TACGAGTAGTGGGGGGAGGTCAATGTTCATCATTGGTTGATATATTATATTGATATTAGGTATATACATAACACAAAACATTTGCAGTATCTACCTTTAAAGTGAAGAGTTTCCCGCACCACGGGCAAAAGTACTTCGTGTCGTGATCTGGATGCACCTTGTGTTTGTCATTTTAAGGAGGTAGATATCATTTCTATTTTAAAGCAATGTTGTTTAAGTATTTATATACAACAACTCGTTTCTATTCTGGCTCAGTTGGTAAAGTACGAGCACAAAAATATGTTCTTACAAAATATTTTCAAGGTGAACCCAAGAAAAGCGATTTTAAAATAGTTGAAGAAGAACTTCCGGAATTAAAAGATGGAGAAATATTGACAGAAGCACAATACCTTAGCGTCGATCCATACATGCGTGCTTATATGATAGGATATAAATTACCGACGGATATGATTGGAGGACAAATTGCCAAGATTATAGCCAGTCGTAACAAAAACTTTCCAGTCGGTAAATATGTTACTGCATCATTTGGTTGGCGCACTCACACTGTGTGTAATCCAGAAGAACCTCCCAGTTTAGGCTTTCTGCCTCTGACTCTTGTACCTGAAGTTAAACCGCATCCAGTTTCTCTGGCGTTAGGAGTGCTTGGTATGCCGGGCAATACGGCGTATTTTGGATTGAAAGAGATATGCAGACCTAAAGCAGGAGAAACCATTGCTATAACAGGTGCAGCAGGTGCTGTTGGCTCTCACGTTGGTCAAATTGCTAAAATATTGGGGTGCCGAGTCATAGGATTTGCTGGAACAGATGAGAAGTGTCAGTATTTAAAAGAAATAGGCTTTGATCATGCTTTTAACTATAAAACAGCTGATATTAGAGCAGCTCTCAAGGAAGGTGCACCTAAACGCGTGGATTGTTATTTCGATAATGTGGGTGGAGAAATAAGTTCGACCATTATTAATTTTATGAATAAAAATGGACGAGTGGCTGTTTGTGGATCCATATCTTCGTACAACGATAATATGTTACCAAAAGCAACTATTTTACAACCTGCCTTAGTGTTCAAAGAACTTCAAATAGAAGGATTTTTAGTTAACCGCTGGATTGATCGCTGGGAGGAAGGCGTCAATGCAAATTTAAATTGGCTACATGAAGGAAAGCTACAATATAAAGAAAAAATATATCATGGCTTCGATAACATGGTTGAGGCTTTAGTTGGCATTTTAAGAGGAGAAAACACTGGTAAAGCAGTTGTTAAAGTAAAATAGATTTTTGATAAAATAAACGCATAATAAATATGTCTATAAAGGTTTCATTTACTACCATCACTTTATGTCTGTCCTATTGTCCATATACTTCATACATAGACAAGACAAACTCAGCTTTCGAACTATGTAAGTGGCTTAGATTATATACCTGGTATATTATATACCAGGTATATAATCTGAGGTAAGAGGCATATCTTAGGGTTAGA

>SinsAD8

ACAAACTTCAGCTTTATTATATTACTATAGATTTTATTTGAAATTACCGTTCGTAGAAAATCAGCAATTTAGTCAAAATGAAATGACTTATTGCATGTTGCAATCAAGTCTAAGAAAGGACTACCTAATGATTACTACTATAGAACCTCAGTACCCAACACGCTCTTGGCCGTTTCTATATTCTTTGAAATTGTCATTTTACGTGCCCGAATTTTAAATTACAAACAAATAAAAAAGTTATTCATTATTATTTTTGCGTCCTTTTTTTTATAAAAATCCTCAAATACATACATACATCCTCAAAAACATACTTTCAAGTCCAACTCATTGGCGTGATGACCCCTTAAACGGAGTATTAGACGTTCACACGATGAATGGCCGTGTGTGTATCTACATAAAACGCAAGCTAGTATTATAATGATTATTTCATATCTGACCGCTGCACGAGCCATGGCCTCTTGTAAAAGTGCATTAATATGAGTATGGTTTACTTACCATAGCTATATTTTAGATCATATAATTATTTGTCTTAATAGCTACAACAAATAAAGTGACTCTTTATACGTATATAAAATTACATATATAGCATTATAAGTTGCTACTTTCCATATTTCTTAAGTCAAAATGTATGAAGTGAAAGATAAGGTGGTGCTGGTTACCGGAGGTGCGACTGGAATTGGAGCCGATATAGTTCGATTTTTTTTAAACGAAGGAGCTAAGCATGTAGCAATATTGGATATAGACGTAAATACCGGTAAAGCTTTCGAAACCGAAATGAATGCTAAATATGGGGCGAACAGATCCAAATTCATAAAATGCGATGTAACTACTGACGATTTAAACACAGCCTATGAGCAAGTACTAAAAGAGACGGGATATATAGATGTTGTTGTCAACAATGCCGGCATTATGAACGATAGTCCGAATGTCTATTTGAAGATGATCGCTGTGAATGTGACAGCATTGATAACGAGTTCGCTTAAAGCTTATGAATTGATGCGTAGAGATCGTGGCGGAAAAGGTGGTACTATAATAAACATTTCGTCAATAGTCGCTTTAATGCAGTTCAGCAGTTTGTCAGTTTACAGCGGTACAAAGAGTGCTGTTCAACAGTTCAGCAATTGCCTTGGGAAGGAACCGCATTACTCCCGTTCAGGAGTTCGTGTTTTGAGTATTTGTTTTGGAGCTACAGATACTGCTTTACTAACTAGAACAAAATTCGGAGGAATTGACAAGGAAACGGATGAGGATTTCTTTGCAGCATTATCGAAACTACCTGTTCAAAGTTCAGAATCAGCAGCTCGTGGTTTGGTAGAAGCGTACAAGCAAGGAGAAAGTGGGAGCACATGGCTGGTGACCACAAATAGACCCGCTGAGGACATCACCAATAATGTCCAAAAAGCATATGAAATATTAGGCCAAGGAGTGTTTTCTTAAAGGGCACAGGCAATGTAATTGATAAATAAAGTAGAAATTGTCTTAAAGAACAGTCGAATGTCATTGATAATCTTAATAGAAATAATCTTAAAGTACTTTAGTTTATATGTCGCATTATTTAAAAGGTTCAAAAGTTTTATTCGTGTATAATAATACTACAAAACCGTCGA

>SinsCYP1

GAGAATGGTGGTAATGATACTTATTCTGAAAGTCAATTGGTAGCTATGTGCATGGATATGTTCATGGCAGGGACAGAGACTACCACCAAGAGCATGAGTTTTTGCTTCAGTTATTTAGTGAGAGAACAAGAGGTGCAGAAGAAAGCCCAAGCTGAAATTGATAGAGTTGTCGGAAAAGATAGAATGCCCTGCTTAGATGACCGAACAAACATGCCTTACTGCGAAGCGGTAGTTCATGAATGTGTACGCCATTTCATGGGACGTACATTCGGTGTACCACATCGTGCCACAAAAAATACCACGTTAGCCGGTTATCATATACCCAAGAACACGATGATGG

>SinsCYP2

CTTTTAAATGGGTTATGAGGAACCTACGCTGTTCCTTCCAAAGTTCTCCGTCTGTCAGCATTAAACCTCGTCTTTCGCCCCATGTCCTTGTCTGATAGAATATACCTTTCGGTCTTCCATCAAAATCATCATTGTAAAGCATTTCTTTGTTAGCTTCTAAACTGTTGACTATGACGATACGATCTTTTCCAATTTTGAGACTGAGACATGGACCACCGTTGCTATATCTTGTAGATAGTTCTTTGACAGCCTTGTATAGGTAACCTGTTTTTTCTCGCATTGTATAAACTTCAAGAGCACTACCGAGTATAGGAATCCATTTAGGACCAGGAGGAAAG

>SinsCYP3

GCGGTTTCACATATAGCACGCAATGTAAATTCGTCCATGTAAGATGAAATATTAGTTTTCGACTTTCCCACTTCATTTTCTAGTTTATGAACCATACACCGGCTATTTTCTTCTAAAACTCTATTGAAATGGCATAGAACGTCAAAATGAAATGCCGGAGTAAGAATTTTCCTACGATGATGCCATTTGGAACCATCACTTAAAAGCAGACCTTCGTTTAACCATGGTGCGAGGAAATCATACAAAAATCCTTTACTATTGTATTTTGTACCAGATATTACTACTTCAACATCTTCAGGATTATACACGACGATAAGTTTTTTCGGTCCCAACTTTACTCTAAACAGCTCTTTATATCTATTAGTTAGCGTTCTAACATAGTAGAATAATTTTACAGGGTCCATTAAAAAATCCAACGCATTTTCAAAGATAAATATTCCCCGAGGTCCTGGGATCTTCTCGAACTCTTCATTTTTTGACCATCGATCGAATGCAAGAAGCAGAACCAATACAAGTAGACACGCCACTAGGAACAATGTCCAGAACATTTCAGTTTTCTGTAATTTCCTACCACTTTGCTACCACTTAATAACAGGACCTATA

>SinsCYP4

GGCAAATTGGTCGAACAATCCATTGAAACCAATAATGCTGATGGTTCAGCAAAAATAGTTGAATTAGATTTAGATGATTTGATCATAACTGCACAAGTTTTTGTTCTTTTCGGCGCTGGTTTAGAAACATCATCTACTCTAACAAGTTACACTTTACATCAACTGGCATTTAACCCTGATTGTCAGGAAAAAGCACAAATAGAAATCAACAGAGTGTTAGCTAAATACAATTACTTACGAAGCCGTTAAAGAAATTATTTAT

>SinsCYP5

AGAAATTTCAAATATTGGGAAAAGAGAGGCGTCATACATGATAAGCCTATTCCTTTCTTCGGCAATAACTTCAAACAATTCACTATGCAGATAAGCATCACCGATCGACTCACAGAGATGTATAAGAGATTCCCTAACGAGAGATACATCGGATTTTACGAAGCAAACGTACCGGAGCTCGTTTTGAGAGATCCCGAGCTG

>SinsCYP6

ACTTTCAATGAAAATTATAACAGATTTTTGTAAAAAGAGAAACTATTTCAAGAGAATAAGCAAATTATTCAAAGTACTTATAGCTCTTATATTATTTGTTATAATAAAGTCTAACAACGCAGTAGTGAGTATTGTTACGCAACCCTACTATGAACATGTCCTTATCATGAACACTCTCTAAAATGAATTTGACCTTGATCTCATCTAATGTTAATCGATAGCGAGGCAGTAAATGGTACGCTCTGATGCAATAAATGAAGAAGAGTTAATTTGAATGTTCTATTACACCATTAAATCCCTAGGAATTAATTCCAAATCTACATTCTTCGGGCGCAGTTGCACTTGTGACTTATTGTACCTCATGCCGCCTTTGGAAACCACAGTACGAAAGTTGAAATGTCGCAGCACATGAACTAAACCAGCTTTCACTTGCAGTCGCGAGAATCTTGAACCAATGCACAATCTACCACCTTTACCGAAAGGCATGTAAGTAAGATCGTTAAACTCGTTGATGCTTTTGTCAAATCTATCCGGATTGAAAACTTCAGGGTCTGGGTAATATTTAGGATCATGATGTATTTCAAATATGGGCGTGAATATTTTCGTGCCTTTATCAACTTTGATGTTACCCACTGGCAGCACGGTGTCCTGGATGCACATTCTCGTTAAAAAACCGATTGGAGGATACATCCGCAATGCTTCAGTTAAAACATTGTCTAAATATTCCATTTCTGATAAAGTGTCATATGTGATATTTCCACCGTTTTTCTCGAAAACGTTATCAATTTCTTTGTGAACACGTCGCATTATGTCAGGGTTTTTACCAAGTTCTATCAAAGTGCAGAACATGGTTGTCGCTGAGGGCTCGACTCCTGCAATAAAGAAAAAGAAAGCTTGAGCTGCCAAGAGCTCATCGGTCGGTTCCAATTCCAAGCCAGAATCTTGGTCTTTTATGATGCCATTCTTTTGTAAACTTATGCATATATCGGCAAAATCGTGTTTCATTACATTTTCTTTTTCCCTGTAACGGATCACTCGTTTTATTGAACCAATGAAGAAGTCCTCATATTCTCCGAACAATTTCAATCCAAGTAATTTGAAAAGCGCTGGACTGACATTTCCAATGAAAAATTTCAAATTATTCATAAACGTTGGGTCTACAGCTTTTCTTGCAACTTTGAGAAAAGGCGAGTCGAATGTTGATTCCGTGCCGATTCCGAATACTGCGGCGCAAATAGCGGCGCAACAAAACATATTCAAAGTTTCATATACGCCTCCCTTTAATTTTTGCGAATTAGCTTTGAGATAATCAATGAAATCTTGAGCACTCTTATCCATGATGTAATACATGTTTTTCAATTTTGCAGATGTAAAAAGGGGTGTCATGCTTTGACGCATCGCTTTCCACCTGGGTCCGTTCATG

>SinsCYP7

TCGTCTCTATGTATCCCAAATACTGGTACAATTATAAGCTGGCCTTTTTCGATTATCAAATTGCCAACAGGCAAAACACTTTCTTTAGTACATTGTCTCTGAACATAACCTATTGGAGGATATTTCCTCATTGTCTCATTTGTGACCATATCTAGATATACCAATTCACAAACAGCTTTATAATTGAAAACATTTTTGTATTTAGTGCTAACTTTG

>SinsCYP8

ATAGAGCTAGAATGGTGTACACAGAAGCAGTACTTCTAGAAGCGCTAAGAATATCAAGCGTAGCTGCAGTGGGTATTCCTCACATGGCCCTCGATGATGCTAGGCTTGGAGACTACATTATACCCAAGGGTACATTTCTTCTGCTCGCCATGTATGACCTTCACAATGGTAGTCATTGGAAAGATCCCCATTTATTTAAACCAGAACGCTTCTTAACTAAAGATGAGAATTTAATACAAGACGAATGGCTGATGCCTTTTGGCACTGGCAGAAGACGTTGTATTGGAGAAGGATTGGCCCGCTCCGAACTGTTTCTGTTTCTAACACATCTACTGCAAAACTTCAATCTCGAACTTCCCATCGAAG

>SinsCYP9

GGATTTGCGGTCAATTCTTTAGATGATCCGAACAACTGCCTCTTCAGACTCGGCCAAAAGGCCGTGATTCAAGACACTACGCAAGTGATGAAACTCTTCGGGTACGAGAACATGAAGACATTTATGAAGTTTCTAAGAGTGAAAATGATTCCAACAAGCGACGCCGAGAAATTCTGTCAACTCTTCAAATCCACTTTGAACGCACGAAGAAACAATTTGTCTCAAACGCGTCTAGATTTCATTCAGACATTGGTAGATGCCCCCCAAGGCAAATTGAAACAAGAAACACAAGACGACAATAATAATAACGGCGAGGCGCACACAAAGACTACGGAAAAATTCACTGACGACGACTTAGTAGCACAAGCGGTGTTATTCTACATAGCTGGCTACGATACTACGGCTAATCTCACCAACTATTTCATATACGAAATGGCTAGCAATCCACATTTGCAGGAGAAATTGTTGGCTGAACTAGATCGATTACCTGCTGACGATGATATCGAAGATATTTACGAAACTATACAGAGCTTAGAGTATTTGGAGATGTGTGTGTGTGAGGTCCTCAGACTATGGCCACTGGTACCCACTGCTGATAGACGATCAGTGGCCACTTACGACTTTGGACCACCTCACCCTGACAGCAAACATACACTTGTGACGGATCCTGGAGTTCACATATGGCTTCCAATATACTCTATACACCGCGACAGCAGATACTGGGAGAATCCCGAGGTGACGGATCCTGAACGATTCGCTCCGGAAAAGAAAACAGTCATCGC

>SinsCYP10

ATAACCTATAAAATTCAGATTCCAAATCGGGATTAATGTTAGATTTTTTTGCATTTAGCTTCCATTTACTTATAAAATTTTCAATAGTACGTTTTACAGGAACTTCCAACCGTTTCTCAGCACCTTCTAGAAGCAAGTGTTTATTCATAACGCGCCGATTTTTTAACCATTCTTCACCATTCATGAAAAAGAGACCTCGTTTAGATCCATATAGTTTTTCATACAAAACCCATGGTTCTGGTAAAATGTGCGCTGGATATTTTCCTTCGAGTTTTATAAAAACAGTCTTTATGAGTGTAGGATCACTAATAAATACAATATCTGCACTGCCGTCTAATCTCTCGTAAAAAATGGGTCCGAGTTGATTATGTCGTCTATCTATATATTCATGCAATTTCGTTCCGCTTCCTGCAGTTAACAGGGCTAATTTTGTTCCAATTAAAGGTAACGATGTTGGATGAGGCATATCTTTAATAAATAACGTGGTTTGCCCAGGATGTCCGGAATCTTTTACATAACTTCTTATATACGATTTTATATTATTTTTACCA

>SinsCYP11

AAATATCTGGAGCGTGTTATTAGGGAAGCACTCAGACTTTATCCACCAGCCGTATTTTTTGCCAGGAAAATGTCTAGGAACACTTTTCTTCCCGACGGAACCGAACTTCCAATCGGATGCTGTATATTAGTGGTACCTTACTTGTCTCAACGTAACCCAGATATCTACGAAGACCCGGAGCGTTTCGATCCAGATCGTTTTTTGAACAACAAAATCCATCCATACGCTTATCTTCCATTCAGTGCTGGCCCAAGAAATTGCATTGGTCAAAAGTTAGCAATGCTGGTACTTAAATGCTGGGTGGCAGTACTGGTACG

>SinsCYP12

TCGGCTTACAATAGATGTCTCCAATAAAATGTTGCTGTTCCTGGTCGCGGTAGTGTTTGTGTGCGTGTTGGGTTGGCTGCGCGCGCGCTGGTTGCGGCTCAGCTCGTACTGGGCGGGGCGCGAGGTGCCGCACGCGCCCCCCACACCTCTGTTCGGCAGTTGCACTTTCTTGCAGAAGAAAAATCCTGCAATATGGTTTCGCGAGATATATGACGAATTTCGCTCTCCCTATGTCGGCATATGGGTGTTCTGCAGACCAGGGCTGGTCGTTAATTCGCCAGAAATCGCTCGTCGGATTCTGGTCAAAGATGCTGATATATTCCGGGACAGATATGTTGGCTCTGGGAAATCAGATCCTATTGGATCATTGAACTTATTTTCGGTTAATGATCCGATGTGGACATCGCTCAGACGTCGTTTCACTCCCGTGTTCACCGCGGCCAAACTAAGAGCTTTGCATGGTCTTATTTCTACAAAAGCATCGGAACTTGTTCAACGTATTCGAACTGAAACCGAAGAGAACAAACGTATAGATATTAGAG

>SinsCYP13

GACAATATATCGGCTGGCATTAGGAACAAGCCATTTTTCTCTAGTTCACAATCTAAAGGCTGTTTAGCATTTGGCCAAGGACAAAATTCAAAATTAAGAATCAATGAAGCCAATCCGAAACGTAGATTTTGATAGGCGAAACGTTCTCCGATACAGTGTCTGGGTCCTTCACCGAACGTCAAAAACGTGAATGGAGTCAATTTATGTTCGTTCTCAGGTAAGAACCGGCCTGGATTAAATTTTTCCGGTTCCGGATAATAGAGTGGATCGTAATGCATGCCGACCGCGTTGACGTACACGGGAGTCCCGGCGGATATGGTTAGCTGATCGTCAATTTTATAGTCCTTGCTCGCTTTGCGGTCCAACCAACCCATGGCTGGATATTTTCTGAGAACCTCTGAATGTGTGGTTGGAATGCCAACTCGTAAGTTGCAAATGTTAGAACGACAGAGGAAGTATCGAAGCCTCCTTGGAGAAAAATAAATGCTTGAGCAATCAAGAGATCTTCCGATATACCTTCCTTATCAGCCTTCCGTTTTATATTCAACAAAGCATCTAACAAGTCGTTGTTTGTTTCCTTCTCTCTGTCATAACCGCCTCTCTGCGCTACTATTGTTCTGAATATCTTGCTCAGGGAATCTATTGTGTTTTGTGGGAACATTTTAAACCTAAATATATCGACCAAGCTAGGTATAAAGAAAATGCTCGACCAAGATATGCCTCTATACCAGCTGTATTTCATAAATTCCTTCGTGACACTACGTATTGGGCCTTCCCCAGTAAGAGTGGCGTCGCAGACCACGCCGAATGCAGCTGTTCCAATGATATCAGTCGTGTAATCTGAATACACTGCTCTAATATCTATACGTTTG

>SinsCYP14

TGGCTCGACTATTGGAGACACCCATGACTGTTTCTGGGTACAAGCTGCCTGCTGGGACTTTCGTCCTAGCGCATACGGGCGCGGCGTGCCGCAGCGAGGAGAACTTCTGGCGTGCGCGGGAATACCTCCCGGAGCGCTGGAGCGAGGTGCGCGAGCCGCACGCCGCCTCGCTGGTGGCGCCGTTCGGCCGCGGCCGGCGCATGTGCCCCGGGAAGAGATTCGTTGAACTTGAGTTGCAGTTGCTACTTGCCAAG

>SinsCYP15

AATGGCGCAATGGGTAACAGACGATAGGCTTCGTTGATGGCGGCGCGCACTGACGGCGCTGCGGCCAACTGTTCCGCGGTGAGGTCGCCGCACGACGGCAGCTCGGCTCTCATGCGCCGCTGCCAGTCCGCTCTACTGCTCAGCATGTACAATAAGAACACCAGGCTGTTCGCTAGCGTTTCAATTCCAGCAGTAATAAAGTCTATAATTGCAGCCTTTTTATCTCGAATGTCGAGTGCGGGATTCGCGAGTATTTTGAGGAAGATCTCCTGCATGCCGTCGTCGTGGGCCGAACCTTGCGTACGAGTCTTTGCTTCATCCATCATTTCCGATAGTGTGTATAGTATCTTCGCTGTTAACAAATGTTCTGTAAAGCCTTGTGGGTGCAAATTTCCACAATGGAGCGCCATAGTAGGAGTCGCGCTGTGCCCTAAAGTGCGCCTTCACCGCGTGGGCTAACGCTGACGCGCGACCAGACATCAAGCGTTCCAGAAAACCCAACCGTGTACCCAGCATTAAGCCGCAAACCGATTCTAGTCCCATTCTGTTAGTGAGTTGCTCAAAACCGTTCACGACACCATCAGTTCGACGACA

>SinsCYP16

GTAGCCGAAATATTTTTAGACAAGACAAATTGGTACTTTGCCTGTGGCAAAGTACCAATTTATCCCGTTTTCTCAGTGTACATAGAATATTTTAAATACAACGTTGTAGCGTTCTTTGTCATATCTAATACTTATTTACGTTTTTTGGAACCTTATACTACCTAGCTACCATATCTCTGGCGATGTCATCTAGGCCTGGTGTGTAAAGCTTGACCAGTTTCGGTTTTAGTAACGCGGGATTTACCTTTGTACGAAAATCACGCCATTTTGAACCTTGTGCTGTTGTTAACCCGTAGACTCCATCAAAAGTGCTTTTTTTCATTTCTTCTCTAAAATAGACCACAGTAGCGAAACCAGGTCGTAAAGGATTTACTTCTTCCGCTCTGAATATCTGTTCATACAAATCAGGATCATACAATATAACCATATTGGCTCTGGCAAGTAAGCCATCCAATTTTACTATGGGTCCAAAATTTTTGTATAGATATTCGGCAATTTCATAGAAATTTTCTTTAGGTCCAATTGTACCAATTACAGGCATAAAGTGATAG

>SinsCYP17

TTTTTTTTAAAATAATATCCAATTTATTTATATTTTATTATAAAAAAACAAGTAATAATTATCAAAATGAATTCTACTAATAAGTAGGTATAAGATATTTTTCTAATGTCTCGATATAAATTCTGCAGTATATGGCCTGGGGGCTGAAATTAAGCCTATTACTGGCTCATCTGAGGGCATTACACCGTTACTACAATCGATGTTGAAATTTTGCAAAATGCCTACAAATAGTATAAATATGAATGACTTTGCTAATGAATCTCCAGGACAGCGTCTACGACCTAATCCAAACGGATACATGTGTTCAATATTCTTCAGAGCGCCATGTTCATTTATAAATCTATCAGGTTTAAACACTTGCGGTTCTTCCCATATTTCTGGATCAAAATGTAAATCTCCAAGTGACATCAATACAGTTGTCTCTTTTGGTATTAAATAACCATCTATAAACGTGTCATGCATAACCCGTCGAGGTCCTGCTAGTGGCACAATGGTAAAGTATCTTTGAATTTCCAACAAAAAAGCCGATGTGTAGATGAGTCTGTGACTATCCATCCAACTCGGAGTTTCGTCTCCAATTGTTTTATTAATTTCGTCAAAAATTTTCTCTTGATAAACTTTATTTCTCAATACTGTGAGCAGAGCAAATTCGAGCACATTGCTAGTTGTTTGTGAACCAGCTATAAGAAGGTCCAAACATATAGATTTTAACTGTTCTTCCGTGTATGTGTCCTTGTTTACTTGCATTTCTCGCAAAAATGAATATATAAAATCATCGCGTTCTACAGCTCTAACTTTATGTTTCTGTATTGCTTCCTCTATTATATCAGATATTTGCTGATTCATCCTAGTTATAAGCGAGTAGCCACTCAACTCTGGAATAAAGAATCTACACCACGGGAACAGATTTAACCAACCGCCGGCTATAGAGAAAGCTTTCGATCGAGCATTCAATAGGTCCAGAAGAAATTTCAAGCGATCCTCTTTTATCCTCTCGCCAGCAACGTAATTCCATAAAACATTCATGACTGTCATAGCTAAGATGTTCTTTGGGTTTATAGGTTGGTGATTATTATGTTTTATATAATCCACTATGTTCCGCATTTCGTTTTGAATTTCCTTCTCCATAACGGTTTTACCGAAGCCAACATTTCTCAAATTTTTCACAGTGAATTGTCTGTGCTCTTTCCACAAAGGACCATCGGCAAAGGTAATACCCATTCTTTTTCCAAGACATCGTAGTCTAATAAAAAAACTGTTAGGACGACCTTCGTATTCTTTTTCCGTGAATACCTGTCTTATGTTTTTTTCACCGTAAACTACTACCACTAGTTCTGTACCTAATTTTAATCCTAATACTTGTGTTGAATACTCTTTTGCTAATACTGATAACGCTTTCCATTGGGAGCCATGTTTTTTTATTTTATCTTGCATTATATTGGTACAACCTATAAAAGGATACCATTTTGGACCTTAAATTGTAAATGGAAGCCAATAATTGTACTACATAAGAACTTCATCGTAACTTATTTAAATACAGGATAGATATCCAGTTATGTTATTAATTATAAACTTAACAAGATCGAAGCAAAGTTCTTCTGTATCAAAGTTTAGGCGTAAGTTTCAGTTGAGGGAATCGTTCTGTTAGAGCAAATAAATATTTCATTCTTGGGCCTTATAGTAGTTCCTACAGCTGTAAGTACAACAAAGCTCATTGAGATGATAAATTAAATGGTACTGTAAATAGCATAAGTGGTCAAAAAACTTGCATGCTGCGACTAGAATTAGAGCAAAGTAGTTATAAATTCGTATGGATGAGGAATGGTAAAATATTTATCCGCAAAAATGAGATACCCTCGGCTAACCTGATCATTTCTCAGATTAGTCTCGGAAAATTACTCGGATATAGTGACTTTTTTTTCAACAATCTAGAACACTGCAATCTATCCAATGAGACATTCAACAATGTTATTGATGAAATATGAGTGTAGTTTGCGAGGTATACACTCAATGATGTATCCCAAGTTGATTCAATATATGGTCATTGTAATGATGCGAGATCTTGAGGTATACTTCTTTAGGTGGTTTTGAATGTTGGTG

>SinsCYP18

AAAATGTTTTTTTTTTGCATGAATACTTATAATGTTATATGCAGAAATATATGATTTTTCTGTTACAGTAGACGCACATCTAAAATATTAATAAATAATTTTCCAAAATTTGTCAACAAGCTTATTTATTGTTTTAAACGATTCCGTATTCAGACAGGCTCAAGTAAAATACAAAACGACAAGTTTTAAAATTATTTGCTTGTTCGTCTGTTGCATGATATAAAAACTATGGACCGATTTCGATAGTACTTCTACTGGGATAATTCTTATTTGAAATAAGACTTGCTACTTTATATTATGAGCGAATGTGTGCTTACCGTTCCCAAATTCGATAACTCACATATCATTTAATTGTAACTTGCGTTCTCGTTGTAAGTGCGTCAATGGCATGAACTTAGAGTGATCATATGAATCAAGCCGTGAGCATTGGCTAATCTAAAATGATATAATATCTATACAACTGTGCGCTCGAAACGGCATTCAACGAGTAGTATTGCGCAAAATAATTCGCAAGCATTTACTTATAACCGAAAATACAATAGATAAAGAAGATTAATTCATTTTTGAATAATCTATCGAATGATAGGAGTGTGGCGACGTTTAAAATCATTTTACACATAATCCTGTGCTTCGTTATGGTCAAGTAATAAATAAATCGTTCTTCTTCCACCAGCTATGCGTTTTGGGAAAATGAAGATACTGGCCGGCCTCGTGACCTTGCTGAAAAAGTGTCGCGTAGAGCTTGCACCAGACACACCCTGGACCGTGCAATATGAGCCCAAGGCAGTGGTCATTCAAGCTAAAGGGGATATGAATTTAAAATTTTTGCTACAAGAGGGGTGGGAGCATAATACTTACATAATAAATTATTATCAAAAAGAAAACTTTTTAAAAACAAGTTTTTTCTCCTTTTTCTCAGCAAACCCTTTCTTTCATTTAATACACATATCATGTGCATAAAAAATAATAAGTCCGCCAATTCAGTTGCAAGATTTGACTGATACTAACAAGGCAAGGTAAATAAAAGCTTCTACAACTAAATATAAACAATAATAATTAGTTTCAAGTGTCAT

>SinsCYP19

CACTTGTATAGTTGTCGTCGCCACTTCTGTCAACTAGTCTCGGAAAATAATAATTCCAATGCAACTTGTATTATATAAATTAACTTTACCGAATATAACATTATCATCATTTTTAGTGAAGTGTTCTGTTTAGTGTGTAGTTATTAAATATTGATATTGTATATAAAAAAAAATCAAAAAATAATTGCCGATTGATTATTATCGTATTTCTCTAGTAAATGTTTAGTTAAAATTCGCGCGAAATGGCTATTTTGCCGATACATTTATTTGTGCACGAGTTAATTGGATTGACACTTTTTGTGTTTTTCTTCGTATATTTATGGTTCCAACATAAATTTTCTTACTGGAGTAAAAAGGGGGTCACCGGACCGAAGCCGGTTTTTTTATTCGGTAACATACAAGATGTGATAAAGAGGAAAGCGCAATTTTTCCAGCCTTATTGCGATAATTACTATAAATATAAACATTTACCATACGTTGGCATGTATTCTTTCCACCGGCCGGTGTTATCTATAAACGATCCAGAAATAGCGAAGCTTATATTGATCAAAGATTTTGATCACTTCCAAGCTCATGGTATATTCTCTGGAGGAGTCGGCGATCCTTTAGCAGGACATTTATTTAACATACATGGTCAGCGGTGGAAGAGTCTACGATCTAAAATGTCTCCAACATTTGCATCGGGAAAACTAAAAATGATGTATCCCTTGGTGGAGAACATTGCCAATGAAGCACTAAACTATGCTGATTTGCTGTGTTCAAACGGAGAACTGATAAATTTTTCCGATTTCTACGCAAAGTATTCCATGGAGATAATAGGAAGCGTTGGATTTGGCGTGGAATGTAACGGCTTTAAAAATCCAAACTCCGAGTTTTATCTTCGAGGACACGAATATTTCGAGCCGCAATCTCTGTATTGGACATTCACGCGAGCATTAGCCTTCTTTGCTCCAGATTTTTTTAAGAAATTAAAAATCAGACGTATTAGTTCGGAAATAGTAAATTTTTTCTACAATTTGGTCAAGGAAACGGTTGAATACCGCCAGGCGTATTTTTATAAGCGTAACGATTTTTTGCAAACACTTATTGAATTGAAGAATGGACAAGTCATAGATGAGAAAGGCAAATCTAAGCTCATACAAGATTTCCCATTCTCTATGGACGATGTGGCTGCCAATACAATGTTATACATGTTCGCCGGCTACGAGACTTCGGCCACGACGGGCCAGTTCGCCGCCTATGAGCTGGCTCGTAATCCGCATATCCAGGCCAGGGCGAGGGAGGAAGTACAGAGAGTACTAGCAAAATATAACGGAATATGCACTTACGAGGCGCAGAATGAAATGGTCTATTTGAATATGGTGCTCGATGAGACGATGCGAATTCATCCACCAATGCGAGCTTTATTTAGGAAATGCACGAAAGAGTACAAAGTACCAAATACAGATTTAATTATAGATGAAGGAACGCTAATATTTATTCCAAATCAAGCAATACAAATGGATCCAGATATATTTCCAGAACCGGACAAATTTGATCCTGAGAGGTTTACGCCCGAAAATAAAGCAGCATTGCATCCTTGTCATTGGATGCCTTTTGGTGAGGGTCCAAGAAAATGTTTAGGTCTTCGTCAAGGGTATATCCAATCAAAGATGGCATTGGTGAAGTTATTGAACAAGTACCAGTTGTTATTAGACAAAAGGACGGCGGTGCCGATAAGACTCAAGGCTTCGTCTTTAGCGTGTGCTCCTGAAGGTGGGGTGTGGATCAAGTTAGAGAAATTAAAACGTATTTAAGGGTTTATTTGGTGTTTTCTCGATATAAATTAATAACAGGGTAGGCATTCGTTAAAAGTGCAACGGTGGGTATATATATATAGGCGGTTCAATGAAATTATATATTTTTTTTGTACAACAATTCTTCTGTAATGACC

>SinsCYP20

CAGTCGAATTTCGACCGTTTGTAGGATGAATTACGGTCGAATTTCGACCATTTACGGGATTTCTAGTAGGTATTAATTATCACTACTAGTTATTAGTACAATAAAATTCAAACATTTAGTTTATTTATAATAAAATACCTGAAATGAAAAAAAGGTATAAAATATAAACATAACAATATTTTGTACGATAAACGAATTGATTAATTTTTAATAATATTACGTATAAACATCTATAATATACAATAATAAAATATTATATTAAAAAAATAGTGAACAATAAAAACATATTTGATGTATGATACCACATTTTCACATTTATAATACAATATTAGTAAGGAAGTAAGGATATAGTAAAGTTTAGTTCCTAGGCACAATATTTAGACGAATTCCGTTATCAGGACTGAGTATTACTCGGTAAGGATTGTATTCCATAGTTAACTTAGTGTTTGGAGACGGCTCGACGCGAAACCTGGACAGCATCTTCACTATACACACGCGGGACTGAGTTTGAGCAAAACGCATACCAATGCAATTTCTAGGACCGATGCCAAATGGTATGTAAGCACAGGGATGCCTGCTATTCATCTTCTCTGATGAAAATCGTTCTGGATCAAATATTTCCGGGTTTGGGTAATATTTTTCATCGTGATGTATTCCTAATACCGGTATTAAAACTGTTTGTCCCTTCTTAATTGTTACGTTGGTGCCTGGCACTTTATAGTCGATTTGTGCATTTCTTTGCAAAGGATCCACTATGGGATACATTCGGAGTGTCTCATTAAATACTTTGTCCAAATAAGTCATGTCTTTCAAAGTGTCATAAGTAATTTGTCCATTATGTATTTTCAAAACTTCATCTATCTCTTCTATGACTTTATTTTGAATGTCTGGATGTTGTGCTAACGTATACAACATAAATGCCATCGTAGTGGCGCTAGTTTCATAACCAGCTGCATAGAAAACAAATGCCTGAGCTGCTATAATTTCGTCAGTCAATTCTAGAATACTTTGTTTTTCATCTGTACTTCTCTTAGTGCCCTTTATTTCACCACATTGTTTCATCTCTAATATTAAATCCATGAAATCTTTTCTGTTTGTTGGCTTACCATTTCTTTGAGTAAAAACAGTTTTAACTAAGTCGTTGAAAAAATTACGTACGGCAATCGGGAAAATGGACATGTTAATTTTTTTTAATATACCAGGATACATCATGTCTAATTCATTAGCATAGTTACTAGTAAATATTAACTTATCCATCCTCGTAAACAATTCTACTTCCTCGTTAAATATATCAGTATCTAAACCGAACGCACACGCCGAAATTGTAGAAATCGTATACTTTTGGACCAGCGAATGAACTTCCTGTTCGCGCTGTTTCAAGCAAATTTTACTAATGTAATCAACGAACTTATCACCTTGCTCAGTCATGAGGTACATCATATTTCTGAGTTTTCCAGAAGTGAATATAGGAGTGAATCGATTTCTTAAAACTCTCCAAGTCTCACCGTCAGCATGAAACAAATTAATTCCCAAACCTTTTTTGCTAAATTCAATTCCCCTGTCCACAAAAGCATCGAAATCTTTGATCATTATATGCTTAATTATATCTAGATCTCGTATCAGTAGACATGGTGTCGTCATTCTATAAATTCCGATTACTTTTTCATTAGGAAACTCATCGTACATTTCCTTGAAAACGGTACCAACAGCTTTTCGCCGAAGGGCTGTTTCTTTTAAATTACCAAATAGAGAAACCGGTTCAGGACCGATAACATTATTCTTCTTCCAGTACTTAAAATTTCTATTTACGTAAAGGTATAGACCGTATCCGATAACTAGGAGCGCGGCCGATAAATACAATAACACCATTTTTACTAATCGCGTTCGC

>SinsCYP21

TAATGATGCAACCAAAGACAATAAAACTTTACAGTTCTGCACTCGAAGAAGTCGCACAAGATATGATTACTAGAATGAGATCCACACGTAACGAAAAGAATGTGTTAGAAGGCAAATTTGACATTGAGATGAATCTATGGGCGCTAGAATCAATCAGTGTAGTGGCATTGGGTGGCCGGATTGGTTGTTTCGATCCCAACTTGGCGCAAGATTCTCCCGAACGCAAACTCATACAATGTGTACACGATCTTTTTAATACGGTTAACGAGTTGGATTTCAAGCCGAGCTTATGGAGATATATTCCAACGAAACAGTTTAAAACGGCTATGCAGCTTTATGAAGACCAACAAAATCTAGCCAAATACTTTGTTAGAAAAGCAGTGAAAGAATTAGAAACGAAAAAAATGTCTGATGGAGAGGATAGTGTGCTAGAAAAACTGCTTGCGGTTGATGAAGAGATTGCCATTATCATGGCATGTGATATGCTATTCGCCGGCGTTGACACGGCTGCCAATACAATCACTGCAACTCTATACCTGTTAGCAACAAATCAAGACAAGCAGAACAAATTGAGAGAAGAGGTGACGTCGTCGAAAGACAAAAAACCTTATGTTAAAGCGTGTATCAAAGAGTCTATGAGAATGATGCCAGTTGTATTAGGAAATGCGAGGGAAGCATCTAAAGAATACAATATACTGGGATATAAGATCCCTAAAAAAACTAATATTGTTATGGCTCATTCGTATCTTTCCATGATGGAAAGTCAATATCCACGACCAACTGAGTACATTCCGGAGCGATGGATAGCTGATAAAGATGATCCTCTCTACCACGGTAACGCTCATCCGTTTGCCTTCAGCCCATTTGGCTTTGGAGTACGAAGCTGTATAGGTCGTCGGATATCGGAATTAGAAGTGGAAACATTCCTTGTGAAAATTATTGAGAATTTCCAAGTGGAATGGTTTGGACCCCCGCCTCAAGTTACCCCCACTACCCTGAATTACATCACTGGACCATTTAATTTTATATTTAAGGATCTTTG

>SinsCYP22

ATCGAATCCGAATAGTTAGTGAATAGCGGGGCAGATATCACAGCGAATATGAGTTTTGCGTTTGCCGAGTGGTGGATGCAAAGGTGTGTGAACTGTATTAATCGTTTATTGTCTCAACTCCATTCTAATCCAGCTGCCTCCTTTAGCTTTTATGTTAAAATCTTCAGTATGCAATTCAATAGGATCAGATGTCTTCTCACATTTGAGGAGTTTAAAATTCAACACAATCTTGTAGACAAGAAGTTTTATTTCTAGAAGAGCAAACCTTGAACCGATACAGTTTCTTGGTCCCACACCAAAAGGCATAAATGTGAATGGTTTTATTTTGTGTTTGTTCTCATCAGAGAATCTGTCCGGGTCGAAAAGTTTCGGCTGCGGGTAATATTTGGGATCCATGTGAATCGAATATAGACTGTTGTAAACAAAGTCACCAGGTTTCAATTGTAAAGTTTTAGCTCCTTCCCTTGGCGGTGGCAATTCGTAGGGTTTTACACACACTCTGTCCATGATCATAGCTGAAGTCCACTTTCTCATTGTCTCATTCAGAACACAATCTAAATACTTGAGCTCGGTTATATTGTCGAAAGTGAGGCTAGAATTTTTCTTGTGGAATGTTCTGATCTCTTGGTAGAGTTTTTCCTGAACTTCAGGGTTAATGGTCAACTCATGGATACACATAACCAAAACAGATGCAGAGGATTCAAAACCGGCGATAAAGAATAAAAACACTTGACCACATAGTTCATCTTGGCTCCACTGTCTTACTGCTCCTTGCGGTTTTACAATTTCCTTTGTTGCTGCAAATCCAACATCTTTTTCGTTCGAATCAGCGGTGTGTTTAAGAATACCTTTGGAAGCTTGCATGAGGAGCTGAATTATATCAGGCCTTTCTACATTATTTTTCTCTCTGTACTCCATAGTACTGGTGACTAAATGTCTAAAAAATTTAATTGTCTTCTCTGGGAACAGATTAATCTTCAATATTTTCAACAGTCCAGGGAAAATAGCCGTAGCAATGAAGAGTAGTTTCTGAACAGCATTGAATTTAAACAAGTTCTGGCCTGCCATGTAGAATTCGTTCTCTTTATCTTTCAGCGAATTCACTTGAAGACCGAAGCCTGCTGATGCAATGACGTCATTAGTATACCGATGCATTATATCACCCAAATCTATATCTTCCAACTGATGATCTTTCAAATGTTCGATAATATTATCACTGATCTGAGCCATGAAAGGCATTATTTGTTTCATCTTGGAGCTGGTGAAAGCAGGACTTAGTGTCGCACGCATATCATGCCATTTTTCACCTTTCATCATAACAAGACTGCCTCCAAACAATTCTGAGTTTTCAAAGAATGATCGATGATCAGTGAAATGGTCAAAGTCTTTGACGGTGATGTTCTTTATCAGCTCTGGATCTCGGATGATAAGGGCCACTGTAGAAGTTTCGATGAATCCAACATATTTTTCATCCGGAAATGCTTTGTAAACAGCGTCGAAATCTTCACATGGGTGTCTTTTTTGTAGAGAGGCTTGTAAAGAATTACCAAACAAAGGTACACCAGGCACATATTTCACTCCTCTTTTATCTAGATAATAATGTGATCTTTTATAAGTATATACGAAATAAACTATAATAGTTGTAAAAATAAGTACTAATAATTCTGATATCATTTCTTTTTTTTTCATAAATAACAAGTGTTATCAAATAAACGGTCTGTTATTATCAGTCGTGTACGACTAAGTGAGATGTAG

>SinsCYP23

ATCACAGATAACTAAGAAGTAAGAACTCTTTGTTGTGAAATATATTATTACAATAAATAAAATAATATTGTTTGTAACCAACTTTAAACATGGATCATAATTTACAAGATTGGCTTAAGATGCCTCCCAAGTATACAACGTTTAGAATAAACAAGCTAAAAAATTTTGACAATACAACTTTGGAAAACTTATTCAAAATGCAAAGTAAAGAATTGAATACATTTGAAATTCCTAACTATTATACACTAAAGCACGACTGTTTGGTGATTGAACAATGGCCCCATGATGTTGTTTTAACAAAAACAAAGAATGAAGTGATTGTTGATGCATTATGTGCAGCTGCTGTGTTACGCGGCTCGCATGTGTTTGCTCCTGGAGTTATGGGCTTACCTACTAATTGTCAATTAGATGAACAGATTGATATCTATGGAGACCTGGAAGGCCAATGCAAAAGGGGTCTCAAAGTGCAATATCATGGTAAAAAGTTATATGTAGGCACCGGATCCTTAAAGATGTTACGTAGTGACCTGTTTGATAATGGCATTCAGCCTAGTGGAATAGCAGTTCACACATTACTACCTGCATCACGTCTTCCTGTTGTAAATGATACTACGTTGTGTTCTAAAGGAGAGATACTCTTGCAAAATTTACCATCAATCGTTTGCGGGTGGGTTGTGGATGCTCAACCTAATGAATATATTCTAGATATGTGTGCCGCTCCTGGTAACAAAACTACACATTTAGCAGAAATGTCTAAAGACCAAGCATTTATAGTGGCCATTGACAAAAATATACAGAAAGTAGTGAAGATTAAAGAAAATTGTAATGCTCATGGGATTACATGTGTTAATGCATATGTTTATGATTCAACAAAATGTTGTTCAGAAAATGGTAATACCGTAGACAAAGGTCCACCCTTTCCGCCGAATACTTTCGACAAAGTCCTCTTAGACGCACCGTGTAGTGGTCTAGGCCAGAGACCACAGTTAACAAATAAGATGACGCCTAAAATGCTTCATTCTTATAAATTTGTACAGAGAAAATTATTTGATGCAGCAGTAAAAGTTTTAAAAGTTGGTGGCAAATTAGTGTACAGCACATGTACCGTGACTGTGGACGAGAACGAGGGTATGGTAGCGTGGGTACTTGAAAAATATCCATCTCTAAAACTAACCCCAGCTACACCATTACTCGGAGGACCAGGACTTTCTAACAGTCATTTAAATGATGAACAGAGATTAATGGTACAAAGGTTTGGTCCCGAGAATGATCCATTGAAGCCTGTTGAATCCATATATCGCGATACTATAGGATTTTTTATAGCCTCATTTATAAAAATACAATAAAAATAATTTAGGTGATATTTTTAAAAAGCAGCAACTTACTTATGTCTATAGTTTTTCAACATGCACCCATAGCCCATTTTCTGGACTTAGCATTAACCTTTTCTTATTAAACTTGACAGGAATTTCAGTTTTAGGGCCCGTTGTAAATTTAAAATTCTTCAAAAGTGATACCATTGCTAATTTAACAGAAAACATAGCAAACCGTGAACCAATACAATTTCGTGGTCCAGCGCCAAAAGCCAAAAATAGATGAGATGGTCTTTGATTTTTCTCTTCACCCATAAATCGTTCGGGTCTGAACTCGTGCGGGTTGGGATATATGTCAGGATCCATGTGGACTCCACACAGAGGTAGAGCAATAAGATCGCCAGCTTTAATCCTAATAGATGTCATTGGCAAAGTATAATCCTTTGTACATACTCTATCTACTCGCGATATCGGTGTATACATGCGTAAAGTTTCTAGTAAGAATGCCTCCAAATACGAGAGCTGCGAGAGGACATCGTAATCCAGGTCTTTTCCTTTTATAACTTCTATAACATGGTTCCTCAATTTGCTCTGTAATTCTGGTTTAGTCGCCATGACATAAATAGCAAATGATAATAAAGTACTAGATGTTTCATAACCAGCTATTAGGAATAAAAGAGACTGTGCGTCTATAGTGTCGTCATCCAAATGTATCTCTCTCTTGCCGTTTTCATGCCTTTCTTCATCAGCTGCATCAAGAACAAGTTGTAAGAAATCATTTCTTTTGACATTAGATGCCCGTCTCTCCGCTTTAGTAGCTTTTAACATTTTTACCAATTCACCTGTCGCCTCAGGGTTTATAAATGATATATTTAAATATCTGATCATTTTCGGCATAAGCATTAGTACTAATAAAATATAAATTCGCTTGGGTAAGGATATGTCCATAAATTTTTTAACCACTTTCGCAAAATGAGCATTTTCGTCTGATAAGGCATCACATTTAATACCAAATGCACAGACACCTATAGTTTCTAGTGTATAATATCCCATAGCATCTTTCATTTCGACATCTTTGTTATCATATTTATTCAAATACTCAATCATTTGCTGTGAGCATGATTCAAGAAGGGGTAACATATTTTTCAGTCGAGAAGAACTAAAAGAAGGTGTGAGTATGCTTCGAACTCCTTTCCATTCTGATCCCTTCAAATTAAGCAGAGATCTACTCAAAAATCTCGGTTTGTTACTATTTAAAGAATTGCGATCAACAAAGTAGTCAAAATCCCGTATAGTTATAGCTTTTAAAAGATCAGGATTGAATATATATAAGATTGGTATTGTTCCTTCAAACATACCTCCACAAGGACTTCCTTTAAAATAATTATAGATATCCAATTGATTTTCATAATATGACTTTTTAGGGAACAACCTTGGGCCTAAATTTCCTAAAAATATCGTGGGTTTCATATACTTTATACCTTTCTTCTCAAAAAAGTCAAATGTACTCGTATAGTATGAATACAAAGCAAATATAATAGTTAAAAGTAAAATTAGTTTCCATTCTTCCAAAATAAAAAATAATAGTTTTTCTGTTCCAACGCCTTTCATTATAATTATGTTTATTTTTTTTTTATTACGTTAATAATTTTCCGTATAATATATAATAACAACTACAATAAATAAAACAAGAACAACTACTGAAATAACTGAAAGTTCTGAAAGACTGAAACTTAGTATAGCTAATTATACTCTTTGAATGAAACTTTAAAATTGAAATCGAAAGTGACAATGTGTGGTAGTGATAGTGATTA

>SinsCYP24

TACGTATTCGAGTCACGGGCGGGGCGCGGTACGTCAACACGCGCGGCCTTGACTGCGCGGGCGCAGAGGGCTTATCTTGGCGTTCGCCGCGCCGGCGGTACTCCTCATTCGAGTACTGACCGCCGCCGCCACTGCCGTAGGCGCCGCGGCAGGTACCGGCGCACGCGCATCGTTTTGTTTACACGCACTCGACACGCTCCGCTCTTTTAGCCGACATTTCAACTATTTGTATGCTAATTTAAATCGTTACAGACTCGTTAAGTTTTTTAGTGAAGATGTGGCTCGGTGTTATCTTATTAGTGATATTATTGACGCTTCTAATATTATTAGCGACTTTTCATTATACAACAAATGGCAGAAAATATTGGTTATTCAGAAAAGTGCCATACAGGGAACCCTGCCCTATATTTGGAAATTTTGGTGCTACATTAACAATGAGAAAAAGTTATACGGATATGTTACAATACTTTTATGATCGTTATCGTGATGAGAAGTATGTGGGAATATTTCAAGCTCGCAGGCCGACGCTTATGGTGATAGACCTGGACATCATCAAAAGTATACTTTCGAAGGATTTTCAGTCGTTCAGTGATCGTATATCTGTGTCGACAGACACGCTAAGAGAACCTCTACTACGAAACTTAGCGAATATGAGCGGCGCTGAATGGAAGGTAATGCGACATATCGTAACGCCGACATTCTCATCCGCTAAGATGAAGGCGATGTTTCCTTTGATAGCGGAATGTGCACACACGTTAAACAGTACTCTGCTTGAGGAGCCCTTAGACGAGGTCGATGTACCAAAATTGATGAGTCGGTTTACGACCGACGTTATAGGCAGCTGCGCTTTTGGTGTCGATCCAGGTTCATTGAAAAACCCCGATTCACCTTTTTTGAGGATGTCACAGAAAATGTTTAGAATCAATCGTTCTACTTTATTGAAACGTTATTGTCGTACGTTTTTCCCGCGTCTATTCAAGTTTTTGAATCTCAGAACGTACTCTCCCGATGTCGAAGCTTTCTTCAGTACTATAATCAAACAGGTTTTAGCGGAAAGAAGAGCTACGGGAATTCAGAGGCACGATTTTCTTCAGTTGATGTTGAACGTACAAAAATCTGAAAACGAGTTTTCTATGACGGATGAATTGATAATATCCAACTCCTTCATATTCATGTTAGCCGGTCTGGAAACGTCGTCGACAACTTTGTCATTCTGTTTGTACGAGCTGGCTAAGGACAAAGAGTTGCAAGATAATATCAGGAAGGAGGTGATGGAATGCATTGAACGTTACGGTGGATTGAATTACGAGGCAGTGAGTGCAATGCATTTGGTTACACGAGCTGTTTTAGAAACGCTGCGACTCCATCCTCCGACGCCTTTCACTACAAGACTTTGTACAGCTCCGTGTACGCTCATAGGCACAGATTTAACATTAAAAGTGAGGGATCCTGTTCTCATACCCTTACATTGTATACAAATGGATCCACAATATTTTCCTAATCCAAAGAAATTTGATCCAGATCGTTTCAATGAAAATTTGAATCCTCCTGGATTTTTAGTCTACGGCGACGGCCCCAGAAGTTGTCCAGGAGCTCGTTTCGCCCATTTGACAGTGATGGCGGGACTGGCCGTGATGCTTAGTGCGTTCGCAGTGGAGCCTTGCGCTCGCACCACGCCGCAAATACATTACGATCCTCGGAGTGTTATGCTCAAGAACAAAGGAGGCATATGGCTGACATTTACACGACTTTAACGGACATTTCTTGTTTATAGAACTTTCTTATTATGTCATGTGTTTCGTCGCTATGCGTTTGAGTCGAGCGTACCGTACCTACTAATTCACCAAAAGTTTGCATTATATGTACATAGATCTGTTTGCATAATTAATTAATTATGATGGCAGTGTCTTCTGTGTCTATAGTAGGAACGTGCTCAATAAAACTATGCAAGTATATATTATAACTATACAAGTGTTTCTTGTGAGAATAAATATGGTTCTAGAGATTGTTCAACGATGCAAGGCATGATGAAATCTTATCGAAATTCCATATCTATCATACGCATTTTCACGATTTTTGTACTTAAGTAGATAGGTATTGATTTAAATATATTACAATAAATATGAAATGTAAAAAGATATATATTAAAGAGTTTTATGTATCTAGTTACGTTGTTATTTTTTTTACGGTCAGACCTGTTGCTCTGGTCCCAAACTAGAGTAATGAGAAACAATGAAAAATTGAACACAGACACGGCGCAAATACTTGTTTTTCCAACAAATATTTGTCTCGCGCGGGTATTGAGCACACAACCACTGCGCCAAGAGGTTATTTGTTCACTTGATAACGCCCAGTTGAATCGTATGCGGTTATCGTAAATTGTTTAAATGCAGTTTGTTATGTTATTAGGTCATGTTACGCAACAAGGGAAAACCTCTCGTATAATAGGCATAAACTCCCATTTCATGTCGGTTACAACTTGTTTTCGATCTTTTCCTTATTTGTTTGAGTATGAAATTTTCTTGCGTAGAAAATATATTGTGTTCACAAACTTAACTGTGTTATATACTTTTACCCATAGTTCTAGCTATTATCTAGGATTCATACGTGTATTAAACTTCCCTATGTGAAAAGAGGAGTGTTATAAGTTTTACGACATACCGTGTCCCGTCTGACTGCTTGTTTGCGGCATCATATCTTGAGAACGGATCAATCGATTTGAATTGGTGTCTGTCTTTGAAAGAGATATTGTGATATGGTTCTTAACTATGCTTGGTAAATTAGTAAGATCTTGAGCTTTCATCTATTTTTCTATTTATTTTATTTTAAAAGAAACTAACCTACCAAAGTACCAAATTTCAAATTTATAGGATTTATAGTTCCGTGATGATGAGTCGTCGTGAAGATATCTTGTATTGGTGGTATTTCTTGTTTATATACAGAATGTGGCGTAATGAATGGAACAATTATGATGTCTCTTATACGTACGTACAAGGTTATCCATTCATTACGCCACACCCTATATAAAGTATAGATCATTTTCTTTTTTTGAAATGATCATACATATGTATTACTCCCAAGTAAGTCCCAAACTAGGTTATAACCTG

>SinsCYP25

CTGCATTTCATGTAGTTCGTGTTTCCGACATGCTCCCATAACTCTAAATATAAATACCACCTGGAGTCGAAAACGCAGAGCCGCAGAGCGAAGCGTTCGTTCTGGAAAGGTCCGTTCATAAATTTTTATTGATTTTTGAAAAACAAATTTCCCATTTGGAATAACAAATCCTTATTTGAATTATAAATAATATTCGGCATCGAAATGTACAGGAGCGTGAGGCCGTGAGCAGTCGACAGGCGGCGGGCCACGTGACCAGTGGTCTCTGCTTACAACAACTAGTTGGCGTCGTGTGATCGCCACCGGTCGAAGTTCGCAGCTGTGCCATTTTGTGACATCACTGGTGAAGATCATCAACGAACCACCTGCAGAAGTCACAACAGGACAAAATGGACTTTTTCTTCATATGGCTGATGACATTTGTCGTAGGATTTTGGATATTCAAAAAGATGAAAGAATGGCAAAATTTACCTCCCGGTCCATGGGGTCTACCCTTTGTTGGATACCTACCTTTCATAGACAAGCATGAACCACATTTAACTTTGACGCAGTTGTCCAAGCAATACGGACCGATTTACGGCATAGGAATGGGTAACATTTATGCGGTAGTGCTCTCCGATCATAAACTGATAAGGGATGCTTTTGCAAAAGATTCATTCTCTGGACGAGCGCCTTTGTATCTAACCCATGGGATAATGCATGGTAATGGTATTATCTGTGCCGAAGGAGCATTATGGAAGGACCAAAGGAAGTTGATAACGATGTGGCTCAAAAGTTTTGGTATGAGTAAGCATAGCTTATCTAGAGAAAAGTTAGAGAAACGAATCGCTTCCGGCGTATACGAACTACTCGAGAACATTGAAAAAGCTTCTGAGTCATCAATGGATTTAAGTAAATTGCTCACTAATTCTCTCGGCAATGTCATCAATGAAATTATATTCGGATACAAATTTCCTCCTGAAGATATAACTTGGCACTGGCTTCGTCAAATACAAGAAGAAGGTTGCCATGAAATGGGCGTCGCAGGCGCTATAAATTTTTTGCCTTTCATACGATTTATTTCATCATCTACTCGAAAATCAATGGAAGTTATAGTGCGAGGACAGGCTCAAACACATAGACTATATGCAAGCATAATTGCTAAACGCCGCAAACTTTTGAATATAGAAAAACCAAAAGGTGCAGAATACACACCACACGCTTATCTCTTCAATGAACATCCAGAGGGACACATTAAATGTATAAAATACAGCAAGCACGCTTCTAATACGGAAGTCCATTACTTTAGTTCAGAAATTTTAATTAGAACTGACGGAGAATGTATCTTGGATAACTTTCTTGTTGAACAGAAAAGGAGATTTGACTCTAACGAAGAGTGTGCACAGTATATGACAGACGAACAACTACTTTATTTATTGGCGGATATGTTTGGAGCTGGCCTTGACACTACATCAGTCACGCTCGCCTGGTTCTTGCTATATATGGCTCTTTATCCGGAGGAGCAGGAAATTGTTCGGAAGGAAATTTTATCCGTATACCCCGAAGAGGACGTGGTTGATAGCACCAGACTGCCCCATTTAATGGCAGCTTTATGTGAAACGCAGCGCATTCGTTCAATAGTACCTGTTGGAATTCCTCATGGATGTGTACAGGAAACCTACTTGGCAAATTATAGAATACCAAAAGGTACAATGGTAGTACCGCTCCAATGGGCGATACATATGGATCCCGATGTTTGGGAAGATCCCGAAATATATAAGCCGAGTCGCTTTTTAGCCCCAGATGGCACCTTGCTAAAACCACAAGAATTTATTCCATTCCAAACAGGTAAGCGTATGTGTCCTGGTGATGAGCTCTCACGGATGTTGTCCTGTGGCTTAGTAGCACGGCTGTTTCGTCGCAAAAGAATACGTTTAGCATCCGAGCCCCCTTCTCCTGAGGAAATGCGTGGCAAAGTCGGCGTCACATTGTCACCGCCAAGCGTAAAATACTATTGCGATCCTCTATAACACATATGATTTTTTTTTTCACTATTGCCAGTTTTTATGCATACAGACTTGTATAGTGAATCGAAACTTCTCAACTTTTGTTATACATAAATACTTATAGAAATTAGTGTATACTCAACTTCACATGTTGTAGTTGTGTGGTTTCCTATTTTATATTATAGGACACTAGACCTAAAAGAGTAGTGTGTGATTAGAAGTGATTAAATAAAGGGAA

>SinsCYP26

GTATAGGTAGATATGTTACACAGATTACAATTTACTGTGCTCCTATCTAAAACTACGAGTATTAAAATTATAATAATATATACGTACTAGAAAAATACGTATTACGTCTATATCTTTAGGATTATATCATATTATTGTTATCTTGTGTTACACGTGTATTTAGATCGTAGTGTGTGTATTTATGTTTATCAACGAAAGAAATCCTGGACATGTCGCAATCGTCTCGAAGACTGAGCGACCGAGACTCGAGATGTAACATACTATGTATTATGTGTTATTATTTGAGTAAAACAAGATTTTTTTATTGTGTGGGAAAATATGAGTAGTTTTGTGATTTCATATAATGTTTTCGTTTTGTGCTAGTGTCAAATTGTATAATATTAGCTAAAAGCTTATATCTGTATTTAATGATAAGGGCTCGATAAGAGATTACCCATTCAATTACAACACAATCCGTCTTCAGCGATATATTGTCAGTCTTTTATATTAATTATTTGTTTAACATTTACTTTAACAAAATGCAAACGACTAAATTGTCATTGTTAATTAAGTTGGCTGCCAATAATAGAAATATTGTCAGAACAGTAGTATCATCAAACAGTGTTACTGTGAACGTAAGCGACCAACTTAAACCATTGACACAAATTCCAGGACCTTCATCGTTTCCGATTATCAATCAATTACATCACTTTTTGCCTGGAGGTTCGTTGTACAAGATAGACGGATTCGAATTGTTAGAGAAATTTTATAAAGAATATGGTCCTATTGTACGGTTAGATGGTATAATTGGTGGTACTCCAGCTTTAGTGATTATTTTCGATGCAGAAAGTGCAGCTCAGGTCCTTCGAAGTGAAAACTGGATGCCAATACGTCCTGGATTTCATTCACTTGAATATTATCGAAAGCACCACAATAAGAGTAGGGAGGACTCTCTTAAACCGACAGGACTTATAACTGATCATGGTGATAACTGGAAACAATTTCGGTCTAAGGTAAATCCTATCATGATGCAGCCGAAAACGATTAAACTTTACAGTTCGGCACTTGATGAGGTCGCCCTGGATATGATTGCTAGAATGAGGTCTATACGCAATGATAAGAACATTTTAGAAGGTAAATTCGATATTGAGATGAATCTATGGGCCCTGGAATCCATCAGCGTGATGGCCCTGGGAGGACGAATCGGTTGTTTGGATCCTAACTTGGCTGAAGATTCTCCCGAAAGAAAGCTCATACAATGTGTACATGACATTTTCAAAGTATCCGACGAATTGGACTTTAAACCCAGTCTGTGGAGATATTTTCCAACAAAAACATTTAAGAGAGCCATGAATCTTTATGACGAACAACAAAAGCTAGCCAAACATTTTGTAGACAAAGCAGTCAAGGAATTGAAAACAAAAAGTAAGTCTGATGAGGAGGACAGTGTACTTGAGAAATTGCTTGCGGTTGACGAAGAGATTGCGTATATAATGGCAAGCGATATGCTATTCGCCGGCGTTGATACGGCTGCTAACACGATTATTGCAACACTTTACTTGCTTGCGACGAATCAAGAAAAGCAGAATAAATTGAGAGAAGAAGTGACGTCGACTCATGATAAAAGATCATATCTGAAGGCTTGTATCAAAGAGTCTTTGAGAATGATGCCAGTTGTATCGGGAAATTTGAGGCAAACAACTAAAGAATATAACATATTAGGCTACAGGATCCCTAAGGATCATTACTTAACATTCGCCCACTCATATTTGTCGATGATGGAAAGCGAATTTCCACGACCTACGGAATTCATCCCAGAGAGATGGATAGCTGAAAAAGATGATCCCCTCTATTATGGAAAAGCCCATCCGTTCGCGTTCAGTCCATTCGGTTTTGGAGCGCGGAGTTGCATAGGTCGGCGGATAGCTGAGTTGGAAGTGGAAACATTCCTCGCGAGAGTCATCGAGAATTTCCAGGTGGAATGGTTCGGACCACCACCTAAAATTACCCCCGCATCTCTGAACTATATTACTGGGCCCTTCAATTTCATATTCAAAGATGTATGAGTGAACATTATACCTATGTATAATATTTAATTTTTTTATTTATATTGAAATAAAATTCGGTATATTTATGAAACAATAAGATTTTCATTTTGATAAAATGATACAATATAGTTTTAAACTTTACAAAAGAACTTGATCGATACAGGAAAACCACAGCGTTTTATCAAGTTTTATTGTTCGTGCCGATTCTTTGATCCTTTGATATCTACCCGCAAACTATTCCAGCTATTTTTCCTTCTGATCACTACGCTTACTTTGAACGGCAGGGCCTTGGACGGCATTGACACTGACGTATGTGAACTAATTCATATAAAATGTATGTAACGATATATTTTTGAGTCTCTGATGGAACATGGCCCTGCCACGTCTCTGTGACTGCACTGATATGTGTGGTTGGTTAGACCGTAACGGTTACAACTCGGCAATGATAAAATTAGTCAGTACAATCAGCTATGATGTCTCTTCTTACAATTACTTAGTTTTATTTATCGATATAGAAAATCATTATCAACACATGGGATCTTCATTCAAATGATTATTTTGTTAATCGATAAGTATAATTTTATATAAGTATTTCGTTGTAATAAGAATGCAAATAGCACGGAGTGTATACAATATTATAGTCACATATTACGTTTTATATTTTTTGAACATATAATGGACCTACTTCAAGATGATAAATATTAATTTAAAATGCATAGATAAGCATCGTACAACATAACGGTAACGTTGTCACTAATATACCTAGTTACATTCTACGTATTAACAACGTAATTTCGGAACAATATACATAAACAAATATCACTATAATACGTATTTGTTATTACCTTAGCAACATATTCTTCTTTTAACGTCTTATTACTAAGAAATTAATTTGTGTAGTATAGAAAGTTAAGTTATATTCAAATTGAGGATATTTGTTAATGTTTAAATGCTGTAAGAGCTAGAGTTGCTAGACATCTCTATAAAATTATCAGTGTAAATGATACCTACTTTTGTTGTGTTTGATAAGAAGTATAAATATATAATACCCTACTATAACATAATCATCTATTCATTTGCGCGAATAAGTGTTAGTTTGATAAAATGCAAAAATTAAAGTTAAGAACGGTGTCGAATTTAGCTGTAAGGCGAAACTTTACTCGTACTAGGTAAGTGTTGCTTGATTTTAATCCACTGTCTAGTTACTGTTAATCGAAGTTTCAATGGTTTTATTAATTTTACTTGTAATTACTCATCTCTGTAGTATAGTGTTATAATATCTGTCAGTACAGTACATATTATACAAAATGTACATATTAGATATACCTACATTATATATTTAGTATGCATATTTGTATTATTTTTAGTTATTATGATTTTTTATTTTATATAAGTGGAAAAATAAAGAAAATCTAGAAA

>SinsCYP27

TTTTTTTTCATTTTTTAATGTATTTTACTTATAATTATTATAATTCATGGATCTTATTATACATAATTATGGTCGACATTTTTTTTTTAATTTAATCATCTCAATTTGATACATTCCTAAAATACCATTAATTTAGTGAACAACATTAAAAGTCGTGTCCAGATTTTTTTACATTTAATGTCTGATCTCCATTCTAACCCAACTACCTCCTTTAACCTTGAAATTAAATTCTTTTGGCGTCAGTTCGACAACTTCACTGGTCTTCGCACATTTTAGTATTTTGAAATTTAACACCAGCTGGTAGAGCAACACCTTCAATTCGAGAAGCGCATATCTTGAACCAATGCAATTTCTTGGTCCAAGACCGAAAGGAATAAATGTCAGAGGTTTTATTTTATGTTTATTCTCATCGGAAAATCTGTCTGGATCGAAAATATCGGGTTGTGGGTAGTATTTGGGATCCATGTGAATGCTGTTCAGACTGTTATAAACAATGTCGCCGGGCTTCAATCGGTAAGGTTGAGCACCTTCTCTCGGTGGTGGTAACTCGTAGGGTTTTACGCAAATCCTGTCCATTCTTATAGCTGGCGACCATTTCCTCAAAGTTTCGTTCACAACGCAATCCAAATATTTTAACTTACTTATATTATCATAAGTGAGATTAGGGTTTTTCTGTTTAAACTCTTTGATCTCCTGGTACAGTGTTTCCTGGATTTTTGGCTTTAGAGTCAGTTCGTGGAGGCATAAAACTAAAATCGAAGCTGTAGATTCAAATCCAGCAGCAAAGAACACAAACATTTGTCCACATAACTCATCTTGAGTCCACTTTCTGACTGGTCCTTGTGGCTTAAGAGCCTCTTCTGTTGTTGCAAATCCAATATCCTTTTCGACACTATCAGCATTATGATTAAGAGTACCTTTATAAGCTTCCATAAGAAGCTGGATCATATCAGGTCTTTCTACGTTATTTTTCTCTCTATACTCCATTGTGCTGGTGACTAAGTATTTAAAGAAGTTAGTAACTTTTTCTGGAAATACTCTTAACTTCAATTTCTTAGATAATTCAGGGAAGAAAATCAATCCCAAAAACCCTAATCTTTGAGAAATGGTGAGTTTAAACAAATTTTGACCAATATTATAGAAATCGTTGTCTTTATCCTTCAATGAATTCACTTGGAGCCCAAAGCCGGTTGAAGCAATAACGTCATTGGTATAACGACGTATTATATCATTCAAATCGATGTCTTGTGAATGATGATCTTTTAAATATTCGACAATATTTTCACCAATCTCCATCATGAAAGGCATCATGTGTTTCATCTTAGAGCTAGTGAAAGCTGGACTTAGTGTGGCACGCATATCGTGCCATTTCTGACCTTTCATCAACAATAGATTGCCACTTAACACCGGTTCGGCTTCTTCGCTAAAAAACGCTCTATGATCCGTGAAGTGATCAAAGTCTTTGATAGTGATGGCCTTTATCAACTCTGGATCACGGATCATGAGGGCGAGCGTCGTGGGTTCTATGAAACCCACATATCTTTCATTTGGAAATGCCTTGTAAACAGCATCAAAATCTTCCCACATATGTCTTCTCATAAACATACTCTTATAAATATTACCGAATAAAGGGAGACCGGGCATATATTTCACTCCTCTCTGATCCAAATAATAGTGAAAATTTTTATATGTATATAAGATATAAACTAATAGAGTTGTCAAAACAAACACTAATATTTCTGCTATCATTTTACTTGAAAAATGGAGTCGTGTGATTGCTTCAACACTACTAGATATACTGAAACGACTCTAAAATTACGTAATTTGTTTGGTATATCTTGTCAAACATACATACAACATATGTAGTGAAACGCTAAAATACTAGGAAAACATAATCATCATACGTATTCCAACGTTTAATCATAGAGTGCCTCCCCCCTCCACCCTACCCTACCTACACATATTCGAATGACCTTAACCGTCAACGGCTTGTTATTCAATTGATGTAATCATTTTAGTACTCTCAATAGAGCTATTTACGAGCACGAATTAACTCGGCGAAGCGCTGCATCCCTGGTGTTGTAAATGTTTATAGGGAACTCAGGTCTATTGTCAAATGGGACAATTGTACAAGACCGATTCAATTCTCAGATATTGCTAATTTCTATAGACACTCATGGCCATTTAACATTAGACATATCGCAAGCTTGTTTGGTACAATATGTGTGACGCCATCGAAGAGTTCCATGGCGTAGCTTCGTACTCGTGATTTAAGCT

>SinsCYP28

GCCACTGTATGATAATTGATAATACAAGCGTATAAATAATCACCACTACTATGATTCATCATTATTCGTTCAATCACCAACAGATAAAGTCATGATGTTGGTTGCTGTTGTCACGGTGTTTATGTGCATCCTGACTTTAGTTTATTTTCTTGTAACTAAAAAAAAGAATTACTGGAAGAAGAAAGGCCTGCCGCATTTGAAACCTTCACTTATCCTCGGCAACTATGGGGATTTCATATTATTAAAAAAGAATCTAGGCCTTGTCATTGATGATATTTGTAAACAATTTCCCGACGCGCCTCTCGTCGGAGCTTTTTACGGTACGGAACCCGCTCTTATAGTCAAGGATCCTGAATATATCAAGCTCGTTACCACCAAGGATTTCTACTATTTCAGCGGAAGGGAACTGTCTGAGCATACCCACAAAGAATTATTTTTTAAGAATGTTTTCGCCGAAAGCGGCGATTATTGGAAAGTGGTCAGACAAAACTTAACTCCATTATTTTCATCGGCCAAAATGAAGAAAATGTTCTATCTCATAGAGGATTGCACTAAGGTTTTCGATAGACTGCTCGATGAGGAAATTCAAGCCAATGATATTCAAGAAGTTAGATATTTCATGGCTCGCTTTACGTTGGATTGTATCGGCTCTTGTGTATTTGGTCTGAATAGCAAAGTTATGGAAGACAACCCCAATAAAAACCCATTCAGAATGATCGCCGATAAAGCTTTGAATTTCAGTCCCGATCAAATATTGAAAGCCATATTAAGGAATGTATGGCCAAGTATTTTCTACATTCTAAGATTTAAATCGTTCCCTGAAGAAGTCAGTAATTTCTTCAACGATCTTCTTGTAAACATTTTTAAAGAAAGAAACTATAAGTCTACCAGTAGACACGATTTTGTAGATTTAATGTTGAATTTGAAGGAGAACGATTGCATCATCGGGGATGGATTGAAGAATCTAAAAACAGATTTAGAAAATAAAGAAAGGGTGAGTTTGAAAGTGGATGATGAGCTTCTGGTGGCGCAAAGCATTGTTTTCTTCTTAGCTGGTTTTGAGACTTCTGCGTTGACGTCGAGCTTCGCCTTATACGAACTGGCTAAAAACGAAGATGCCCAGCGACGGGTCTTGGAAGAAGTAGATGATTATATAAGAAGACATGACGGTCAACTCGGCTACGAGTGCGTCACTGAGTTACCGTACCTCAATGCCTGTCTTGACGAGACTTTACGTCTTTATCCAGTGCTGACTTTTCTCACTCGCGAGGTGGTTGAAGACTACACGCTCCCTACCGGTCTGATGTTGGAAAAGGGATTACGAGTACATCTACCTGTGTACTACATGCACAGAAACCCTGATTACTTCCCAGAGCCGGATCAGTTCCGTCCTGAGCGGTTCCTAGGCGAAGAAAAAGAGAACATTAAACCATATACGTATTTCCCGTTCGGAGAGGGACCGAGAATTTGTATAGGTATGCGTTTTGCAAAAATGCAGACAATGGCAGGTCTGATCACCGTGCTGAAGAAATATCGGGTGGAACTAGCGAAAGGCATGCCACGCACCGTCGAGTTTGAACCCAAAACTTTTGTCACAATATCCACAAGCGGTATCAACTTGAAGTTCATATCGCGAAGAGTTGTTTCTAATCGCGGCTAGTTTCTTATTTTTATTATTGACGTTATTTTACTGTTTACCATTAGTTGATGTTAGAAAAAAGGTTTTATGTGGTTCCTTAGTTTACAATGAAACCTTTGACCCAAAAACTCACAAGAAGAGATTATTGAAAGAATAAATTAGTCTTACCGCTTCTAATGTCTAAACTAAAGACAGCTAAAATCTGAAAAATATTAGAATTAAAAAACATACATTGAAGTCGGTTTTTTT

>SinsCYP29

CGGCCCGCCAGTGCGCAGACGTCGGTCGCACGCGATATAACTACAAGTACGACGTTCACGTGCGGACCGTCGCAATTTATTCGCGATCAAATGTTACGCTCGGTTCGCAACTGACGAACAGATTCAATTTTTGTTTTACAATAATATAATAAAAAAAAACTTTTGCAATGTTGAATATGCTAACAAACTCTAGACTGTTGTGGGGTCTGTGGCAAGTGGTGAGCTACTGTGCGTCGCGCACGTCCCTCCCGTTGGTGGCAGCTGCATGTGCAGCGCTGCTGGCGGCGCAGCTGACGGCGCGCCTGCGTGCAGCGAGGAAACTGCCGCCGGGTCCCTGGGGACCGCCGGTCGTCGGCTATTTACCTTTTCTCGGCGTACGACACAAGACTTTTTTGGAACTAGCGCGCAGCTACGGAGCATTGTTTTCCACGCGGCTCGGAAATCAGCTAGTCGTGGTCCTCAGCGATTACCGGCTTATTCGTGAAGCTTTCCGTCGTGAAGAATTCACTGGTCGCCCGAGTACACCTCTAATGCATACTCTAGATGGTCTCGGAATCATCAACAGCGAAGGCCGTCTCTGGAAGAGCCAGCGGCGTTTCCTGCACGAAAAGTTACGAGAATTTGGGATGACTTATATGGGAAACGGCAAGAAAATCATGGAGGGAAGGATTAAGAATGAAGTACATGAATTGATGACAAATTTACACCGCACCGAAGGCAACGCAGTAGATGCAAACCCCTTGCTTGCTCTTGGCGTATCCAATGTGATCTGCGGTATTACCATGTCCGTGCGATTCAGTCACGGCGACGAACGTTTCAACAGGCTTAACCACTTGATCGAAGAAGGCATGCGATTATTTGGAGAAATACATTACGGAGAATACATTCCTCTTTACAATTACCTACCCGGCAAAGCTCAAGCGCAAGTGAAAGTTATGAAGAATCGTGAGGAAATGTTTGCATTTTATCAAACTTTAATTGACGAACACCGCGAAACTCTCGACATCAATAATGCTAGAGATCTCATCGATGTTTATTTAATTGAGATCGAGAAAGCCAAAACTGAAGGAAGAGCTGGAGAACTCTTTGAGGGCAGAGATCATGAATTGCAACTGAAACAAATACTCGGAGATCTTTTCTCGGCTGGCATGGAAACTATCAAATCGTCGCTTCTGTGGATGATAGTATTCATGTTGAGAAATCCAGATGTGAAGAGACGCGTGCAAGAAGAGCTCGATGCTATAGTCGGTAGAGGTCGACTACCTACTATTGAAGATATGCCCAACTTGCCTTATACGGAAACAACGATACTAGAGACTTTACGCATGTCCAGCATTGTACCTTTGGCAACGACACATTCTCCCACTAAGGATGTGCATCTAAATGGTTACAAAATCCCGGCCGGTTCACAAGTTGTACCACTCATCAATTGTGTACACATGGACCCAACACTCTGGAACGAACCTAACAAGTTCAATCCGAGCAGGTTCATAGATGAAAACGGAAAGATTCGTCGTCCGGAATATTTCATGCCTTTCGGCGTGGGTAGGCGGATGTGCTTGGGCGACGTGCTCGCACGCATGGAAATGTTTATGTTCTTCGCGAGCATGATGCATCAGTTCGACGTGCAGTCGGAAGCGGGCGCCGCACCGCCGTCGCTCGAGGGCACGGTGGGTGCTACGATTGCGCCGCAGACGTTCCGCGTGAGGTTCGTGGCGCGCGAGCCGCTGCTGCCGCCGGCGGCGCCTACGCCCGACCACCCGCACCTGCGCCACGTCGGCGCGCACTAGCCGTCGCGTCGCGCCGGACGCATTGCCACTACACATACACCCACCAGTATTCATTGATCACTCGTTTTAGAGCGCATCGAAGACCATTTATTTGTTTATTACGTAAGTGACGACATCGCGCGGACGATGTTCGAGGCTGTGCAAAACGTGTTCAATTTAGAATTAACCGAACTCATTATTTTAGATATATTGCAAAGTGTCCATCGTTGAGATTAAAAACAAAAAAGAAACAAGCGTGTGAATTTGCAGTATTAAACGATGTACCTAGATTGTTATTAGGATTTTGATCCGAGATCATTGCAAATCACGTCGGATATAATACTTCTATATGATATTATTAAGTTAATTGTCGGGTTAAGTTTCTAGTATACCAAATTAATGTACATTCTAGATCATTATTTATTAAGATCACTTGTGTGAATGTGATGTCTGTTGAGTGTGATCTCATTCGTGTATGTTGTTTTGATAAGAAAAATCGTTAAAGTAGGGTATTGTTATGACATACAGGTGTCCCTCTAGCTAATATCTCTAGCAATATCTGGAGTAGGTACATGATAACATTCCATCTAGTGTAAAAGTAAAAGCCAATCCGCACCTAGTCTAAGAGCGGTCGGTCTCTATAACTAATAGTTGTTTATAATATTGTAACTTAATGAGACATATAGGTTTTAGGCTAAACTTTCATGTTGTTTGCAACAAAGTTGCTATGTACTGAATTACAATATTAGATATACAATATAAGTCTGTTTTAACTTGCAACTCACCGTTAAATAGTACATTACCATTATTTACGCTTTGTAAATATTGCTTAAAATATGAGTACCAATTTGTAATGAAAATGAAAAATAAATGAAGAAAATATTTGACGAA

>SinsCYP30

CAGCGATTTGTGCTTGCTGGTTGCGTTGAGGTCGTGCGATAAATAATATCTCTGTTAGCAATGTATACAGTAATTAAACATAGAAGAAATTCATTTATACTAGGAGATTATATTCGACTTAAATACTTTTCGAGTTCCATTACTGATAATGGTGACTCTACAAGCGTGAATCTCGGTAGCAAGAAAACTATTAAAAATTTCGACGATATTCCTGGACCTAAAAGTTATCCGGTTATAGGAACTCTTTATAAATATTTTCCTTTCATAGGAGATTATAACGCGGAGACTTTAGACAAAAACGCCTGGTTGAATTGGCGACGCTATGGTGGGCTGGTACGCGAGTCACCAGGTGTGAAACTTTTGCATGTGTATGATCCTGACAATATTGAAGCTGTCTTTCGTCAAGATGATCGCCATCCGGCAAGGAGAAGTCATTTAGCTATGCAGCACTATCGTCTTAGTAAACCGCAAGTATATAACACAGGCGGATTGCTCTCGACAAATGGTCCAGAATGGTGGCGACTGAGAAGTACGTTTCAAAAAAATTTCACTAGTCCTCAAAGTGTCAAAGGACATGTGGAACAAACAGATCAACTAATGAAGGAATTTATACAATTTATAAAATGTAAAAAAAATTTGCACAAAGAAGATTTTTTGCCTTACCTAAACAGATTGAATTTAGAAGTAATTGGGATGGTGGCTTTCAATGAACGATTCAATAGTTTTATGCCAGATGAACAGAATTCTAAATCAAGAACCAGTAAACTTATAGCAGCAGCTTTTGGTTCCAATAGCGGTATAATGAAGTTAGATAAAGGTGTCCTTTGGAAATTTTTTGAGACACCTTTGTATAGAAATCTCGCCAAGTCGCAAGAATATTTAGAAAAAATATCAATGGATATTTTGCTGAGAAAAATACATTTCTTTGAACAAGAAACTGCTGAAAACGACAAATCATTATTAGGATCGTTTTTACAGCAACCAAATTTGAATTTAAAGGATATTATTGGAATGATGGTCGACATATTGATGGCTGCTATTGATACGACTGCGTATTCAACGAGTTTCCTATTGTATCATATAGCTCGCAACCCAGAATGTCAAAAGAAACTTTTCGAAGAGATTGTAACAATACTTCCCAAAAAAGACTGTCAGCTAACTGCTGAGATGTTGGCGAAAGCAGTTTATGCCCGCAGTTGTGTAAAGGAGAGTTTGAGGTTAAATCCTGTGTCTGTGGGTGTAGGACGTGTGGCACAAAAGGATATGATACTGCAAGGGTATTTGATACCAAAAGGGACAATAATAGTAACGCAGAATATGGTAGCGTGTCGTCTGCCACAATACGTGCGTGAACCTCTTCGCTTCAAGCCGGAGCGCTGGTTGCGCAATACCGAAGAATATGAGAACTTACACCCTTTTCTTAGTTTACCATTCGGATTTGGTCCTCGATCTTGCATTGCCAGGAGACTGGCCGAACAGAATATATGCATTACATTAATTCGGTTGGTACGTGAATATGAAATGAAATGGGTTGGTGAAGAGTTGGATGTGAGAACACATTTAATTAACAAACCGGATAAACCTATAGCATTATGTTTAAAGTCAAGGATTTTATAAAACAACGTACAAAAACTTGTGTTTTCGTGATAATTTGATTTATTATAAATAAA

>SinsCYP31

CTGTTTTTTACCACCTAAAAATAAGTCAGTAATTATTTTTACACAATGTTACTTGGTTTAATGTATACAATGCGTACATTTCGGCTTAGATTGGTATTCTCTTACACCTATGTGTATTTAGATACTGAACCCTAAATAATGGTTTTTGTAATTTTTCTCTGTTTATCTGTTTGTGTGTTCGCGCACTAAATAACAACTACTGAACCGAATTGAATGTCTTTTTTGTTTTCGAAAAAGTTTCTTGAACATTTTATGTTGCATTTCAGGAGATATATTCAACGAGAAAAATTATTTTTCAAGTTTACTTTTTCTTGTATGAGAAAGGGGTAAAAATATTTTTTCCAAAAAATCGTCACTAATTTCCATCTGTGTGTTGAATTTCATCTCTAGATAGAAATGAGAGAGATGGACGAACGCGTACAGAGAAAGTATAACGTATCTATTGTGAATTACAGAACTGTAAAAAAAATGTCTAGATATATTTAACAGCTGGATGTCTTAGCTTTTGTATTTCCCTAAATCACAGTAGATTGCCATAAAACACAATTATTTCTGTCTTCGAACAAATTTGACGTAAATCGGCTCTATGGGGCGTAATACCATGTCTACAATCAGCGCCGGTCTGTAACCCTTCTGTCGTGGCACCAGAGCGAAGTTACGGCATATCTCACTAAGGATACACTTCATCTCCTGCATTGCGAAACGCTGGCCTAAACAATTTCTAGGTCCAGCGCTGAATGGCACAAAGGCATACGGGTGCCTGAGTTCTCCGTTCAGGAACCGCTCAGGCTTAAACACCTCCGGGTCCGGATACAAGTCTGCGCGATGGTGGAGATCGTAAATGTGGATACCTACTTCTGTACCTTTTGGTACTAAGACATCACCCATCATAAAATCTTCAGTGATTCTTCTAGCAATGAACGGTGCGCTCGGGTACAAACGAAGCGTTTCCTTGATCACCGCTTCCAAATATTTCATTTGGGCCAAATCGGTCATGCTCGGAGTGCGATCTGAGTCTCCAAAGATCTCCTGGCATTCCTCATAAATGCGCTCCTGAACATCTTCATGGTCAGCCAATAGCATCAGACCGAATGTCATAGCGGTGGCAGTAGTATCAAGGCCCGCAAACATAAAAGTGTTCACTTCCTCCCTAATGCCTTCCAAATTGATCTCTCCTTTACTTTCAGCTTCTAGAAGAAGATCCAACAGGGCTAGTCTTCTTTTACCACCGATGACTTCTTCATCTAATGCTTTTGTTACATCATTTTTATTACTTTGATTACTATTCTTTTTCCTCTCCATGATGACGTTATCAACAAACGAATGGATCTTATTCAAGCAAGTAGCAAATTGTCTGCCTATATTTAATTTCTTAAAGATAAATTCGTTCTGTAACCACACTTTGGTTGTTCGGTTTAAAACATATCCACCCATATCTAGAACGGCGTTTTTGTAAGTGACAGCTGCTTCCGACTTGTCAAAGTCGAGCTGGGTTCCCATTGCAGTTTCGCATATAATGTAGAGAGTAAAATCGCTGATGAAAGGCATGAGGTCCACTTCAGTGTCATTGTATTTAGCCTTGAGTAATTCCACCATGTGACGGCTTTTCTCCTCCATGACGGTCGCGAAGTTCTTCAGGATGTTGAAGTGGAACGTCGGAGTCAGGATCTTGCGGCGTGTGTGCCACTTGCTGCCTGTGGTGACCAGCAGTCCAGTGCCCAACCAAGGCCTCAAAAAGACATAGTGTTTGCTTTTAGTGATATTCCTTGAATGTGATAAAACAATCTCAATCTCTTTTGGTCCATTAATTTGTATGATCCTCTTACTAAAGATCCTTAGTACGAACTTATCGCCATAGTCCTTGGCGTATTGGCGCAGTATGATAAATAATTCACGCGGAGAAGCAAACATGAGGTCCAAAGCATTTCCAAATAGGGGCTTAGGTGGAGGTCCCGGCACCCTGTCTAGGGGATGTTCCTTCTCATTCAGGAAGAGTTTCCAAAGAGAGTACACGGTGAACACAGCTAGGAACACTTCACATAGCATTATGATTTGATTTTTTTCTTCGCTGGTTCAACAGTGAATTGGTTGTATTTAGAAATGATGATAATAAAACCACGTCCGCTACTTTAGTTTAAACACGTAAACTTTTTAATGTAACTGAACTGATTTCTTTTTCAAAATTTCTATTCACCGGTCACACAATCATTAAGGATTGGCTGCGGGCATGGCAATG

>SinsCYP32

ATATCTTTATAAAAACTACAGAGAGACTGTCTCTCTTTCTCTCTTGAAGCAGTGAGTGTAGAGGGAGAGTTTAAGACCTAACGCTTTTTCGTGTCATGTATCATATTTTCTGGCCAGCTTGCCGCCAACCTGCGATAAAGGACTTCGTTCCAAAAATATTAAAGAAAGAATTGAATCCATAATAAAACAACATTGAAAGAGTACCTAACATCTGACTGATTTGTTTTATTCATTGTTCTTATTAAGTTCTAGACGTAACTCGAATCCAGAATTCCGGTATAGTCGTTATGATGCCTTGGATTCTTTTTGAGGGCTTGGTGATCGGTTTACCATCTGCAGTGGACACGTGAAACGCTTGCATGAATGCGGCAAACACTTGGAACATCGTTTGCCGGGCGAACGTCTCTCCAGCACATAAACGTCGACCTGCACCAAAAGGTAAGGATTTGTCGCATGATAACTGTAATTTTCCATCCACGATGTACCGTTCCGGTCTGAAGTTCTCCGGATCACCCCATACATCCTTGTCCATGTGTAAATTCGTGAAATTTGCTGCAACAAGGGTGTTCTCAGGTATGTCGTATCCTCCAATATTAGTGTCGGCCATGGCCCTGTGCGGCACCCCCAGCGGCACCAGAGTGTCGTATCTCATGGCTTCTCGTATGCAAGCTTCGGTGTAAGGCAAGTTTTTTCTGTCATCCAAATTGGGCAACCGATCTCTGCCTATGATTCGGTCGATCTCTTCGTGAATTTTGTCCTGAATGTGTGGACGCATTAAGATATGTTCGATGAGCATAGTTAACACCGATTGTACCGCAGATGCCGCGGGGAACATGTAGTCTGTACAAGTAAGCACTAGTTGATCCACTGAGAAAGTAGTGCGTTGCTTCTGTCTAACTTCCTCTTTCATTTTCCTTATGTAGACATCTAAAAAGTGGCGATCGTGTGACTCGTCGTGTGTTGCCATTACATCATCAATCAATTTCCTGAAGAAATCCAGCAAATATTGATTTCCAGTTTTCAATCCTGTGTAACCGCTATAATTTGGCATAACGTCTTTTAACCATGGCGTTAGAGAAAGAGCGCCACCCAAATCATTGGTGCTTCGTTGAAACAGCAGTGTGAATCGTGCCATGTCCCATAAAACATGATAATCAGGTCTAGCCAACGTAGTTCGAGCAAAAACATGAAGCATACCGTTGATGAATGGAACGGCTAAATAAAATGGCAAATATACTAATTCTCCTTTTACTAATTCCTTTTCAGCACGATATTTAGGTCCACTAATCCTCATATCTAGCATGTCTTTTATCTCGTTGGCCACCACCGATTCTAACGTTTCATCGCGTCTTCCAAAACCGGAATCTCTCAAATATCGGAGAGAAAACCTCCTCTGGACATGCCAATAATATCCATCGGTGAAGAATATACCTAACTTTTTCCAATAGGATCTTAATCTGGCAATAATGATATCATTGCGTCCATCAAATTCCTCGCGATTAAGCATTTCTTTAATTAAATTTGGATCATTTATAACGATGGAAGGGATCGCTCCTAGATATAATCCCACTATTTTCGTTTTATATTCTTTGCCGAGTTTGCTGAAAGCAACTGCCAAATTTTCGATTTCTTTCAATAACACAATCCAATAAGCGCCGTACAATGGGAGACTCGGTGGGCCAGGAGGAAAGTTTTCCAATCTTTTAAAAGCATTTTTGTAAAAGTGCACAATTATAAAGGGAATAAGTGCAACAAGTAATATTACACCGATTATCATTTTACACTTCAAAAGATTTTTTTATTATTATTAAAATTAAATATATTAAATATTTTAAAATATATAAACAATGTTCCTCAGTGAAATGTTGGCAACTTACTAACAAGTTATAGGCTTATAGGCCGTAACCCGTCCTGTTATTAAAACATATGTTCCTCGTATACATAGT

>SinsCYP33

GTGTGGGAATCGAACCCACGCCCTTCACGAGTATAGGTAGACCTACAAACCACTGTTCCAAGGGACCGTTATTTATTTATAGAATCTAAAACGCTGTTTAAAAGTTTTTGTCCTGTGCGGAAATCGAATCCACGACCTTCGCGGTTACAGGCAGCTGTACTGTTACTGTTATTTATTTATAAAGCTACTTAAAAGTTTCCTGTGCGGGAATCAAACCCACGACCTTCACGACTATAGGCAGACTTCCAAACCATTGTGTCAAGAGACCGTTATTTATTTATACAATCTAAAAAGCTATTTAAAAGTTTTGTGCTGTGCGGGAATCGAACCCACAACCTTCGCGACTATAGGCAGCCATACAAACCACTGTGCCAAAAGACCGTTATTTTACAAATATCGGATCGGTTTTTTGATTTCACCATTTTCAATATGGCTGCAATACAAAAGTTTTTTTTAAGTGACATCAGATTTTTTTTAAAAATTATAATAGGTATTTATTTTTACGCCTTCATATTTCTTCTAAAACAAAACAAAAAAATAACATTGGCTTATTTTATATTTACGTCAAACATAACATCATACTGTAATTCATAAACTATAAAACACGAAATATGAACTTATCTAGTTTTGCTGTGCGTTGCACTATAAACGACTACGTAAAATCGGAAGTGGCAGTTGCGGCACAGAGCGACGTAACTTCTCTCCGAGCGCTTCTGGACGACTAAATTTTTCGCCCGCCTTCACGTCTACTTTAGAGAACACAGCGCGCCCATAGTAAAATTGTATGGCGAATTTATAATGTGTTTTTTAATAAATTGTTGTAATTTTATCGGGCACTGTTTATTATTATATGTATATACATAAACATATATATACCTACAAGAATAGCTCGCTTGAAAGTTAAAGTAATAAGACTGTATAGATAGATAAGTATAAATATGAGAAACTATTGTTTATTTGAATTTAGAATCTCATAAAAAATTGCCTTGATACAAAAGGAAAAAATAAGGCAAAGTAATTTATAAATTAATAATTTTATTTTAAAAGAACGAGGAAATATAAGTATAAATTTTATATATTACGTACTAAATATTATATGTAGATACATGCACGTGTACAAGTAAAGTAATGCGCAAATTCTAAACACGAGGCGACAATTTTATAAACACTCCATTTGTAGATCTTAGTATAAGATCTCCACATAATTCAGGCTCTTTTCCTGATGGCAATATATTAAAATGTCTGACTATTGCCGAAAGCAATACTTTCATCTCCAACATTGCAAATTTTTGTCCAATGCAGTTTCTGGGGCCTGCGCTGAAAGCTAACCAACTATATGGATTTTTCAAGTTCGACGTCTCAAATCGTTCCGGTCGAAATTCCAATGGACATTCGTAAACATCAGGATTTCTTTGCATCTGAATTATGTTAATTATCACCGAAGTGTCTCTCAAAATATTGTAACCGGCTATTTCAATATTTTTATTAACTGCCCTTTCTATTAGTGGCACAGAAGGATAGAGTCGTAGAGATTCCTTTATAACTAACTCCAGGTACTTCATTTGTTGAAGTTCTGTGTATGTTGGGTCTTTTTCCAAATCTGAATTTAAAATTTTCATTTGTTCATCAAGAATTTTTTGTTGGACATCCATGTTTTTAGAGATACTATAAAGACAATACACGATCCCAGATGTTGTGGTGTCATGACCCTCAAACATGAAAGTGTCCACTTCTTCTCTGATGCTTTCATCATCAAATTTTTGACCATCTAACTCTGATAATAGCAGTAAGTCCAAAAAGGCATGCTTATTTTTCAAACCAAGGTCAGTGCTGTCACTCAGGTTAGTGATATTTAATTTTTTTAGATCTTGTCGTCGTATTTCCATTACGTTTCTTGTGTGCTCGTGTAAGATTTTTAATGCTTTTTCTTGTTTATTTTTGTATGGGAGCAAGTTGAAAATTACGTCTGCAGTTACGAATGGGTTCCGCATTCTTAAAGATACAATTTCGCTTAGAATTTCAATAGCCTTCACGTATTTAGATTCGGAATGGTTTTGCGCATTGAAGGAAACCCCCATAATGGTTTCTGCAACATTATCAAGAGCAGCCAAAGCTATTATGGGAAATAAGTCAATGGGTCTCCCATCGGCAAGATTTCGTAGCTTGCGTCTAAGAATTTTCTCATTCTTTAGAAACACCGGCAAGAAAGTTTGCAAAATGTTGTAATGAAAGGCAGGAGTTAAAAACTTGCGATGTGTTCTCCATCGATTGCCTGAAGCAGTCAGAAGGCCTTGGCCCAACCAAGGTTGTAAAAACGTATATGAATTTCCCTTAGTAATCATCTCTGTACTTGATACTATACCCTCTATATACTTTGGATGAGACAAAATAACGTATCTTCTTGATAAAATATGAGCACGAACAATATCTCCGTAACGTAAAGTTAATTGTGAAAGCCTTTTTAAAAAATCAGATGAGTTTCCCAAAAACAAATGTCCATTTCCTACTATTGGAAGCGGAAATGGTCCACGTATGTTTGTTTTCTTATTGTCTTTAATCAAATTTAACCATGAGTTCAAAATAACCACGATAAAAACCAAAGCGATTATGAATAATATCATGTTTATAGGGCGAAAAAAATGATGTGTTTACTTTGTAGAGCTTCAGTAAGGACTGGCGAGCATTTTAGCGCAGAAACCTATTATATATTAATGTTATATTAATTAACTATATTAATGTATGTTCTTAGGTATAAATCTTTCTTAGCAGATTCTATTTTGGTTTGATATTTTGCATGTCAGCTTTAATAAGTTCATGAAATAGTCCTAAATTAAGAATGTATCCTGTCAAAGCTATAGGTAATACAAAAAAAGACAGGTAATAGGGCATTGATTTCTTGATTTTCACCTCGATCTCGAGGCCGATGTCTCAGCTGATGGAAGACGATTACGAGGAAGAAGATAAAATGCTTCATTCTAGTTTTGAATTTTGATGAGACCCTACATTAACAATAGGTGGGATATATGACTTGGCAAATGTACCTAACATAAAATAAACCAACATTTACAGCTACGAGGGACGGCTGATAAATTCG

>SinsCYP34

GGATTGCAGTACATTTATAACAGTAGACGAATCAACACGTGTGATAATATATAAACTTTTATAATCTTTTATTTATTTAATTAAGCAATAATATTTTAAAAAATACGAGTAATTGACTCATATTTTTAGCAAAATGATAGCGGAAATAGTGGTGTTCCTTTTTACGACATTAATAGCTTATTTTTTGTACTCTTATAAAAAATTCCATTTCCACTTTAAGGAGAGGGGTGTGAAATATTTACCTGGTATTCCGATTTTTGGAAATGTTTTAAAAAGTACGATATTAAAGAAGCATATTTGGGAAGATATTGACGTCGTGTACAAAGCTTTCCCCGATGAGAAATATGTAGGATTCATCGAAGCTATCACACCGATCATCCTCATCCGAGATCCTGAGCTGATAAAAACCATTACTGTCAAGGATTTCGACCATTTTACGGATCACCGGGCATTTTTTCCAGAAGAAATCGAACCTCTGTTTGGAGGCAGTATTTTTATGATGAAAGGGGATAGATGGCGTGATATGCGCACGACATTAAGTCCAGCATTCACGGGCTCCAAGATGAGACACATGATGCCGTTCATGCTGGAGATCAGTGAAAATATTGTCAATTATTTGAAGGATCACCAGACAAAAGACATTGATGTGGGAGAAGTGATGCGTCGTTACACCAACGATGTGATAGCTTCAACCGGTTTTGGACTTCAAGTGAATTCATTAAAGGATAAAGACAATGAATTTTACAAAACAGGCCAAAATATATTCACATTCACCTTCATGCAGAGGTTTTGGATGTTCTTTTTGGCAGTATTTCCGGAACTATCTAAGAAAATAGGAGTAAAAATTTTCCCAGAAGAGACAACGAAATTCTTTGAAAATCTGGTTCTTCGCACCATGGAATACAGAGAGAAGAATAATGTGGAGAGGCCTGATATGATCCAACTTCTCATGGAAGCTTCTAAAGGTACTCTCAAACATACGACTGAAAATATAGAAAATGATGTGGGATTTGCAACAACTGAAGAGATTCTCAAGCCACAAGGAAAAGTTAGAGAATGGACCCAGAATGAGCTAGTTGGCCAAGTGTTCCTATTTTTCATTGCTGGATTTGAGTCCTCAGCCAGTGTTATGGTAATGTGTATCCATGAGTTGGCACTAAATTATGACATTCAAGAGAAGCTGTATCAAGAAATCAAAGAATTCAAGAAGAGGAACTCTAAACTCACCCATGACAACGTAATCGAGTTGAAATATTTGGATTCCGTTTTGAACGAGACTTTCAGGAAATGGTCTCCAGCTTTGGTCATGGATAGAGTGTGTATAAAACCATATGATTTGCCACCACCAAGGGAAGGAGGTAAACCTTATCGAGTGAATCCAGGTGATATAATTTACAATATGGTGAACTCCATTCATATGGACCCGAAGTACTATTCTCAGCCGGACATGTTCGACCCGGACAGGTTCTCCGACGAAAACAAACATAAAATACAACCATTCACATTCATGCCTTTCGGAATGGGACCTAGGAACTGTATAGGTTCAAGATTCGCACTTTTGGAGCTGAAAGTGATCCTTTATGATTTGGTGTCGAACTTCAAAATCCTTAAGTGTGCAAAGACCACAGATCCAATCGTGTTGCTACCGAAAGACTTTAACATCAAAGCCAAAGATGGAACTTGGGTAAAATTGGAGGCTCGGAATTAATTTTCTTTTTTATTGTACATATTAGTAATGACATGACGCTGAATATGGTAGCAAAAGCCAATGCCCAGCAGTAGGAGATATCACAGATTTTTTATTTCTTTTTTAACAACTTTAGATGTGTGTTCCTTACATTGTAAACAAATCTTACAATT

>SinsCYP35

AATTATGTCAGTTATTACATGAAATGAATCTTACGTGAAAGAACGTTTCACATCAGAATATTACTATCTTTTATATACCCAAACAGTAACATCTAATCTTAGGAGAAATTCTTATTTAGTGCGAGTGAAACATTAGAAAATTATCTATCAACTAACCTAACCTAACCTCGAATTTGAACAATTTTACTTTATTCCAGAAATATTATGCAATCTAGAAGATATAGCAAAATTAGCATATCAACATAAAGGAGTTGTCAAACTATGGCTCGGTCCTAAATTATACGTCGCTATTGGTAATCCAGAAGATGCTCAAGTTATACTGGAAAATTGTCTCGATAAAGATGGTGTATACCGTTTCATGAGGCCTTGGCTTGGTCACGGTTTATTCGTAGCACCTCTAACTCTATGGAAAACGCATCGAAGAGTTTTACTACCCGTGTTCCATAACAAAATTGTTGAAGAATATCTTGGTGTGATATCTGAACAAGCAAACATTCTGATCGATAGACTTTCTGAACAATCTGATAAAGGAGAATTTGATGTCCTCAAATATATCACAGCCTGCACTTTAGATATTGTGTTCGAAACAGCAATGGGTGAACGTATGGACGTGCAGCATGCGCCAGAGACGCCATACCTTCGTGCTCGTCACACTGTCATGACGATACTCAACATGCGTATTTTCAAAGTCTGGCTCCAGCCCGATTGTTTATTTAATTTGACACCTTACGCTAAACAGCAGAAAGATAATATTGAACTTACTCACAAGTTCACCGATGAGGTGGTTAAGAAAAGGCGCATCGAATATGAGAGGCAAAAGAATGAAGGAAACATAGAGAAGACAGACAAATTACGAGCCGTCCTAGACTTGCTGTTTGGTCGGGAAATCGAGTTCACTGACGAACAACTGCGCGAGCACATCGACTCTATAACAATAGCCGGCAATGATACCACGGCACTGGTCATCGCTTACACGCTGACACTACTCGGAATACATCAAGATGTACAAGATAAAGTTTTACAAGAACAATATTCCATATTTGGAGATTCAAAGCGAGGTACCACTAAGGAAGATTTACAGCAGATGCATTATTTAGAGAGAGTCATAAAAGAGAGTATGAGATTTTACACCGTTGTTCCAATAATTGGTCGCACTGTTGATAAAGATATTTATCTTCCCGGATGCGGGGTAACCATACCGGCTGGTGCTGGTGCTGTAGTGGGTTCATTCGCGATCCATCGATCAGAAAGCGTATGGGGTCCCGACGCCAATAAATTCGACCCAGATCGATTTTTACCAGAACGATCTGTTAATAGACATCCGGCCGCATTTCTGCCTTTCAGTTATGGTTCTAGAAACTGCATAGGACGCAACTTCGGTATGTTAATAATGAAAAGCTTACTATCGAGCATCGTGAGATCGTACAGGATAAGCGCAGATGAGATCGGCTCCCTCAAGATAGAAATGCTTTTGTTCCCGATTAAAGGTCACCAATTAAAAATATCTAGACGATAAGAATACATTAATTCATTTACGTGTACTTGTTGTATAAAATAATCTTTTTATAATTATTATTATTTTATAGACACAACTAACCACAGAACATATAGGTAATAGTGTAAAATCCCAGGGCCCCCAGACGG

>SinsCYP36

GTAAATGTATGGTCAATCTTGTTTTGATATATGTACAACATGAGGCCGCGTATATAATGAACGGCGTCAACCTGATATCTTTTAGTTCATTCATATAAAGTAAATACGAAAAAACACCTCGGTCGAGTCTTTGCATAAATATACATACATATATACATAAAGTCATTGTATAAACGGACAAATATGATCGATATTATTTTACTTGCAACAATCTTGGTGTTAGCTTTGATCACAGTGTATTTAGCATCAATAAGAAAATTTAACTATTGGAAGAAAAAAGGTGTACCTCATTTAAAGCCAACGCCTATTTTAGGCAATTTTAGCGGTTATATACTATTAAAGGAATATACGGGCGAAGTTACACAAAGGATTTGCCAGAAATTCCCCGATGAACCATACGTCGGAGCTTTTTACGGTACTGAACCCGCACTCATCGTTCAAGATCCGGAATTAATCAAACTCATAACTACGAAGGACTTTTATTACTTCAATAGCAGAGAAATTTCAAAATATACGACAAAGGAAGTCATTACACAAAACCTGTTCTTCACATATGGGGAGAGATGGAAGGTTTTAAGACAAAATTTGACTCCATTATTCACGTCGGCGAAAATGAAGAAAATGTTCTACCTTATAGAGAAATGCGCTCACGTGTTCGAGAAACTCTTAGACGAGGAAACTGCTGCATCAAATGTGTTAGAAGTGAGAGAGCTAATGGTAAGATACACCATGGATTGCATCGGTTCTTGTGCCTTCGGTGTCGAAACAAATACAATGAGAAAAAGTTCCAACAAGAATCCCTTCACAGTAATAGGAACTTTAATATTCGAAATATCAAATTCTAGAGGATTCCAAACAATCGCACGCGCTATCTGGCCATTCATTTTTTACGGGCTAGGTTTCAAAAATTTTCCGACTGAGATAAACGATTTCTTCGGAAAATTGTTGACTGAAGTTTTCCAGAAACGACAATACAAGCCCTCGACGAGGAATGATTTCGTTGATTTGGTCTTGAATTTGAAACAGAATGAATATATAACTGGTGACAGTTTGAGTAATATGAAAACGGGCGGAAATGAAAAAATTAGTTTGAAAGTTGATGATGATTTGTTAATAGCACAGTGCGTGGTGTTTTTCGCAGCTGGATTTGAAACTTCAGCTACAACAATGACTTTTGCACTGTATGAGCTAGCAAAGAATGATAAGGCCCAAGAGCGGGTTCTTGAAGAGATTGACGAGTACCTTAGCAAACACGGAAACAAACTAGGTTACGAGTGCCTGAAAGAACTTCCTTACCTTGAAGCTTGTTTTGATGAAACTTTGCGTTTGTATCCAGTATTAGGTGTCATCACTCGGGAAGTAGTTGAAGACTATACTTTGCCTACAGGCTTGCGTTTGGAGAAAGGTGATCGTGTTCATCTACCGCTCTATCATCTGCATCGTGATCCTAGGAACTTCCCAGAGCCAGAACAGTTCCGTCCTGAAAGATTTTTGGGGGAAGAGAAACAAAATATTAAGCCTTATATTTATTTTCCGTTCGGTGAAGGTCCAAGGATATGTATTGGTATGCGCTTCGCGAAGATGCAGATGCTGCCCGGCCTCGTGACCTTGCTGAAGAAGTACCGCGTAGAACTTGCACCAGGCACACCCAGGACTGTACAGTATGAGCCGAAGGCAACTGTCACGCAACCCAAAGGAGATATAAAGCTGAAATTTTTGCTACGAGAAGGATGGGAAAAAAGGATATATGAAAAACAAAATGTTTATACCAATAATAATAATTAGGTTTAATGTTATTTATCCTTAATTAAAATAAATTACTAATTTAATAAAAAAAAAAAA

>SinsCYP37

TAGCCTTCCCGTTTTCTCAGTGTACCATGTATGTACATGCTTTTAATAAACTGGTTAAATTGTCTATTTCGGAGGTTTTCTTTTTGTTCTGAACACAGTTTGCCTCTCCATCAGGACCCAGGACTATCTATCACATGATATAACACACGAGCGCTTCCACGTGGTCCCCTGAATAACGGCCGCGACCCTACCCCATGCAGACTCGGACACAACCAAAACTTCCGACAAAGACTACATCTCTCAATTTTACAATGAAGACGAGAACTAAAACAATAGGAACGTAGTCTACATGATGCCAATCCAATACGTCTCCAGATGGTCCCTCATGCACTACTATTGTCACTGTTCAGCAGTTCACTATAGACTTTTCTGTAAATGGTTAATTTGAGCCTTTTTTCAGGTTAATGAAATTGAAAGATGACAATAATTATCTTCAGAACAATTTTGATTTAGAAATGACGAAATGGTCTTTAGAATCAGTGGGATTGGTTTCTTTGGGCACTAGACTCGGGTGTTTGCGAGATGATTTGCCAGAAGACCATCCAGCGAGACAATTAATAAAATGTGCTAAAGACATCATGGAACTTGCGTACAAATTGGAATTTTACCCGAGTCCCTGGAAATACATTTCCACACCCAATTTTAAGAAAATGATGAAGACTCTGGACTTACAATGGGTTTTAAGTTCAAAATATATTGAGCAAGCAAAAAAACAGATAAATGAGAGAGGTCATGTCATCCCTGAAGAAGAAAAGAGTGTTATCGAAAAATTATTGGCTATTGATGAAAAAGTAGCCATAATGATGGCAAATGAAATGCTTATGGCTGGAATTGATACAGTTGGTGTTAAATTATATTCATTTGTTAAAAATTATGCAACATAATAAAATGGTTATTATTCATTTTTATAGTCATAATATTTTTAATTGTTTTAGGTGGCATTTGCGACTACAGGACTGCTTTATCAGCTCGCAACGAGTCCTAAAGTTCAAGATAAATTACGACAGGAAATAAGATCAAATGATCCCAATAAACGGTATCTCAAAGCTTGCCTTAAAGAGTCTCTGAGGCTGTGGCCTGTGGTGCCATCTAATTTGAGACGAACAACCAAAGATCATATTGTAGCTGGCTATAAAATTCCTAAAGGAGTAGGTTTTTTTCTTTCACTTTGATTTCTTAAAATAGACACTCCATTTTCAACAGAATTCCATAGTCAAATCCATTTCAGCCTTATTACGGATTCTTGCTACAAACTGTACGTTGAATAATACTCCATAAAACAAACATTTCTCTGCAATTTCAGGTAGAAATAATATCAGCCAATGAATATTTGTCAAGGATGGCCAAATACTACCCGCAACCAACAGAATTTATACCAGAGAGATGGCTAGTAGAGAAGACAGATCCTCTGTATTATGGCAATGCTCATCCTATGATAACTTTGCCATTTGGATATGGTGTGAGAACATGTATTGGTAGACGAATTGCGGAATTAGAAATAGAAGTATTAATAACAAGACTAATAGATCAAGTTGAAGTGAGCTGGAACGGACCACCAGTAAAAGTTGTCACTAGAGTCATAAATTCATTTGTAAAGCCATATCATTTCAAATTTGAAATTGCTAAATAAAACAGACTTGCTATTTTAAAATCCAATATTTTAAATATATTATATTACATATTTATTTTTCTTTTTTAAAACTAA

>SinsCYP38

GCGCGAATGTATTCGTTGGCCGACTTTATAACTTGTTGTAGGACGCGATGAGCGCATGGCCCGAGTGAGTCACAGTTCACTCGGGCCGTGACAATCGGGGTTGGCACGTATTAATTTTAATTAATTACGCAAACGCTTGTGATCGCAGTTATATACGAATACCATCTAATGATATATGTATTTGTACATCTTCATAGACAGTAAATAAAAGACATAGAACAAATATGTGCCATTAAATCGAATCATGTTTTTATTATATGTCAAAAGTGACAACCCTAGGATGAAACGTTATATCTAAACGATAACTGCATTTATTGCGACATCGAAGGATATGGACCATTGTGTAAATACTATAACGGTCAACTTAAACATGATAATTAAATAATAATTAAAAAATGACTAAAAAGACTTTTTAACTTTGCCATAGAATAAAAAAACAAAACAAAAATCTAAGCAGCATTTTGTAAGACAAAATAAAATGGCGGTTGCAACCTATTGACCCCAGTAGTGGCAACATTAGGAGCGGGGCCATGCCATTCCAGTCTGTATTTCTCAAGTAGCCTGGTTGCCAGAGTTGCCAGCATTTTGACAACGACCCCACTGGCAGGACAGGATTCGCCAAAAGGCATTGAAGCTAACGGATGGGCTCTGGAAGCTCTTAAGGGTTGCCAGACTTCGTTACACCAACGTTCAGGAACGAAGGCATTCGCTCGACCCCACTGTTTCTCAGATTTGCTTGTGACGCCGTGAGCTAGTACTATGTCCACCCCAGCCGGCACTTCATATCCCCCAATCACCAGATCCTCCCTGCTCCTCCTGACCACTCCCCCTGTAGCTGGGAACAGTCTGACAGCTTCTCTGACGGAAGCCGCGATGTATGGCAGCTGAACACGTCCAGCTCCAGCTTGCACGGAGGCCGTCGCCCAGGAGACCTCGTCGTGAGCTCGTTGTTGTTGTGCCGCGTGGAGAGATAACTGATACAACATGCTTATTGCTGTTTGGACTAGCGGGTTTACTCCAGCCAAGAATATATCAGCGGCTAGAGGCAGTATCCGTCTGTCCAGAGGTCGCAGTTTGTCTAAAAGTATTTGTTCTGGTCTAAAGTCTCCACTGTTCAGAGAATTGAGTGCTTTCAGCAAGAAGTGTTCTGTTAGACTGAAATGTCTATCGAAGGCTTTCAAAGCTTTGTTGAAAGTTTTCGTATCAGGTCTAAGCGTGGATTCACTGCGCACCAAGTAACCGCCGCGCGCTATTTCAATCGAACAACGAACTAATCTTTCGCCAGGAGTTAGTTCCTCGATCGAACGCTTTGATAATGAACATAATTGACGAATATCATCGTCCAATGAAGTATTCTCTGGTGTTTTATTTTCTGCAGTGGGTTTATGTGTGTCTCCATCCAAACAGCCAAGTCTAATGCCAAATATGGTCATCCCTATGGTCTCGATCGCCCATCGATATATTTCTGTCTCTAATTCTTCGTTCAAGGCATTTTCCTCATTACGCAATTCACTCAACCTTCTCGTAGCATCAGTTGCGATCTCGTCAAATGCCTTTTCATAATTTTTAAGTAACGTGCCATCTTCAAGGAGTGAGCGAATCGCAACCCATAGAGCTGCAGTTTCATCACCGTGGACAGGGCAATGGCTACCAACACTGCATCTGTGCTGGCGTAGGGGAGACTTTTCCCAGCGAGGTGGTTCAGTTACGCCACTTTCGTACACCTCTCTCATCATTTCAGGGTCGAAGACATACAAGACAGGTCTAGTCCTAGACGCCTTGGCCAACCTGACCAGATCTCCGTATCGGTCGCGAAGACCTTCCAGCAAACCCAGTCCTACTGTGTGATGGAAATTACCTATCCTGGGCAATACATGTGCTGAATGTCTCAACATAGGCAGCGCCAGGGGTCCCGGTATGTCCGTGAAAGGTTTCACATCAACGTGAGTGGATTCAACGGGCGCAGCCATGGCCGAGTGGCGCTGTGGAGACGTCGACGAACGACGGCATGATGTGGTGGCGATGTGTCTTCTTGATGGACGAGAAAGAACATACTGTCGAATTAAAATTGTCGATTTCGACATCGTAATTTACTTTTGTGAAAACAAAAAAAACTTAGTTCTGGAATAAAATAATACCAAAATAGAACACAAGGAATAAAAGTTATAATTATTTTCGTCCAACACTAATCATTTTCTTTCACAAATCACAATTTTGATAGGTTTTTTATTTATATCTTTCGTCTAATTGCAACGCAACGTGACTGTAAGCTCGCGCGAG

>SinsCYP39

GTATGTATCTAAAAGTCGGTCCGTATGTCGACAGCGATATCGTAACAATATATAACGAGTTATATTCAAACGTGGCGGCTTAGTACAACCATAACTGTGTTGAGGACAACACATTCACAGCATTGACATATCATCATTTTTACATCGAAAATGATAATAGAAATCATAATATTCCTCGTGACAAGTATCGTAGCTTACTCTTTGTACAGTTATAAAAAGTATCATAATTATTTCAAACAGAGAGATGTGAAATATTTGCCTGGTATTCCGATATTTGGGAATGCGTTCAAGAGTACATTTTTGTTTAAACACTTGTGGGAGGATATTGATGAGATTTATAGAGCGTTTCCAAATGAAAAATACGTGGGCTATATTGAAGGGATGACGCCGATAATCGTCGTCCGTGATCCTGATCTGATAAAAAGCATCACCGTCAAGGATTTCGATCATTTCACCGACCATCGCCATTTCTTCACCGAGGAGACCGAACCTTTGTTTGGAGGCAGTCTTTTGATGATGAGAGGTGAGAGATGGCGAGACATGCGCACGACACTCAGTCCAGCTTTCACCGGCTCCAAGATGAAGCTCATGATGCCCTTTATGACCGAAGTCTCCAACAACATCATTAATTATTTGAAAGAACATCAGGGAGAAGACATAGATGCCGCAGATCTGATGCGTCGTTACACCAACGACGTCATTGCTTCGGCGGCCTTTGGTTTACAAGTAGATTCACTCAAAGACAAGGACAATGAATTTTACACCATCGGTCAAACTTTGTTAAATTTTACTGGAACTCAGCGATTAATCATGTATATTTACGAAGTTTTCCCTCAATTAGGCAAGGTGTTAGGCTTAAAATTTTTCCCAGACAGGACTGTTAATTTCTTTAGGGAGATTGTCACCAGCACCATGGAGTACAGAGAGAAGAACAAAGTAGAAAGACCTGATATGATTCATCTTCTCATGCAAGCTTCAAAAGGTAATTTGAAACAAAATAGTGCTGATAGCACGGAAAAAGATGTTGGTTTTGCCATAGCAGAAGATATTACCAATACTCAAGGGAAAATAAGAGATTGGTCTCTAACTGAACTCACTGGTCAAGCTTTTGCATTCTTTGCCGCTGGTTTCGAAACTTCGTCAACAATTCTAACCATGTGTTCTCATGAGTTGGCTATAAACCCCGATGTTCAGGAGAAACTGTACCAGGAAATCAAAGAGTTTAAAGAAAGAAATTCGACGCTTACTTTTGAGAATGTGGGCCAATTGAAGTATTTGGATTGTGTTTTGAACGAAACCTTCAGGAAGTGGTCTCCCGCTCTGATCATGGATCGAGTTTGCACTAAGCCCTACGAACTACCTCCGCCAAAGGAAGGTTCTAAACCTTATCGTATCAAGCCTGGTGACATTATCTACAACAGCGTAAACTCTATCCACTTGGATCCTAAGTATTACCCTCAACCAAAAACATTCGATCCAGAGAGATTTTCAGAAGAGAACAGGCACAAACTAAAGCCTTTTACGTTTATGCCTTTCGGTATTGGTCCAAGAAATTGCATTGGATCAAGATTCGCACTTTTGGAAATAAAAGTATTTCTCTACAATTTGATACTGAATTATAAAATCCTCAAGTGTAAACAGACAACTGATCCTATTGAACTGATGCCTGGTGATTTCAACGTTAAGGCCAAAGGAGGCAGCTGGATCAAATTAGAGGCTAGAAGTTCAATTTAAGCAAACGTCTTACTACAAAAGACGTAAGAAAAAGAAGACTGACTGACTTACAATTGTCACTGCACAGGCTACGTCAAACGTGAAACTAATAATGCTATTGTTGAAGTTATTTAAAAATCTAAAAAAGGATTATTCTGAATTTCTCTTGAGGAACTTAAAGCGATATTTCATGCGCACTAAGATGTGGACTACAGCTAGCATAAGATTTAGGATAGTAAGTCAGGATGTTAAAAAAGTATTTGTTAACACATCTACATTAGAAATGAATCCTTGTGTAAAAAGAATCTTAACGAAGCCAAGAGCCACGTATTTTTACCGTCATTGCGACGATTTTTTTTTGTACTGAATCATACCTGGAAATCGACTGATTATGGACATATTGAAGATCAAAGTCACAATTTTCTTGTTAGGTTTTTTTTACGAAAACAATCTTGAAATGTTATTACTTAACTTTATCCTATATAAACATTAACAAAATTTTAGATATTACTTAGCAAATGAGGCTTTTAAAAATACAAGACGCAATGAGTATCGAGCGGGCACATACAGCCCTCGCCTCGGGCCCGCCCGACCCCGCTCTACACACACACGTACAGTGTGTAGCGCGCGTCACATTGCTTACTAAAGATAACAGCTGTTTTTTACCACTTAAGAATAAAAGTCCTTTCCCAGTAATTATTTTTGCACAATGTTA

>SinsCYP40

TTTTTTTTTTTACAATTTCATTGATTTTATTTAATAACAATTCTTCAAACGTCATTACAAAAGTAACAAAAATACACATATGTATTTTAAAATAGTAGTGAAAGAAGTAATCAGCACTATAAAACTGAAGAGAGAACAAACTCTATTTATAAAAACAAATCCCTTATCCGCGCACAAAATATATAGAAATACACAAGACAGCTACGATATTAACTTATTCAACGTAAAAATCTTATTAAGAGCACATGTGTTCTACTGTGGATATGTATCCAACGTGAGAAAATGCAAACAGCAATGCAACAGTTTAGTGCCTACTATACATGTAAAAAATTATAGCACCACATATGTTATCCTGGTCCGCCTCAATGTTCCTATAATGCACTTGAACCCACTATTGTCTCCTGTATTGTGAATTTTCAATTTCATGTGTCGGGTTGAAGGCATATTCAACCCGACACGTGAAATTGAAAATTTAAGCGCAAGCTCGGAGCTTATCTATAGCTGCTATAGTATTATCAAACAATTCTTTATTCATTCACTGAAATTCAACTAAGAAGAAAGTAACGCCATCTCCTTTTATGCCCAAGGTTTGTTTACAAACAGGGATGAGAGTTAAATATCCTTGAAAATACTGCACGGATTTCCGTGAAACAGACCGGAGGACCATTCCACATAAGCCAGCTTTCAAAGGAAGAAAACCGTTTTCAAATCGATCGATCCGTTTTGGAGTTATAAAGAGACAGACAGAACGACTGACAGACCATGGGGTCTTAACTTATAAGACGAGCGGCCTCATGAGTCCGTGCTTAACATATTCGTAAACTATTGTCTTATATATTACTTATTGATCTGCAAGGGGTTTTCTTCTTTCCAAGGCTAGCTCGTAGCCATCCACAGCCTTCATCATGATGTCCAGCTTCACCCTAATCTTGGGTATAGTACTGGTCTCTCTTTCACCGACTACTCTGAACCGCCTAAGAATTGCTGATAGTGCCGTTTTTATTGACATTAAAGCGTATTGATAACCAACACAATTTCTAGGACCATTACTAAAAGGCATAAAGCAACACGGGTGCGGTAAATTCAGTCGCTCCGGCAAGAATCTATCTGGATCGAAATGATCAGCATTCGGACCCCAGTACTTGGGGTCCCGGTGAATGCCCCAGATGGATATGACGAAGCCCGAACCGGCGGGGAGAACTCGACCTGTCGGCAATGTAATTTCTTCTAAAATTTTTCTGATAATAAAAGGAACTGGTGGAAACAGACGAATAGATTCTTTCACCACTCGCTCCAAGTACTTGAGTTTTAATAGATCTTCCTTAACTAACGGCCGGTCCGATTCGCCGAACACCTCATATAACTCTTGGTACACTCTCTCTTGTACTTCGGGATATTTTGTCAACAATTTCAATGTAAACCCAACAGCATGAGCTGAAGTATCTGTGCCGGCTATAGTCAAAGTAAGCACTTCTTCTCTTAATTCTATATCATTATAGCCAGTTTCGCCTCCAGAAAGCGTTATTAGGAGGTCCAGAAAGGTCTTTCGTTTGTAATATTTTAAATCATAATTTTGATTTGTCTCTATACTCTTATCTTTTATAAATTGCAATTCTTTACGTTTCTTCTTTATTACCTCGTCAGTAAAGGCGTGTAAAGTGTTTAAGCATCGCTGATGTTCCGTGTATTGCGGAAACAATTTGAACATCCAGTCTGGTTGCAGCCACAAATGGAAAATCCTTTCACACACCAAATTTAATAAACGACTCAACGCCTTCAAGAATGGGGAATCAGGATTCGATTGCGTGTTCATCTTGATCCCCATGGCTGTCTCGCATACACTGTCAAGGGTATAGGTGGAGAGAAAAGGCCAAATACTGAATTTTCCGTTGTCAGCGCTTTCGGTCAAGCGTTTTGCAAGTTTCTCACTTTGTTCTGAGAAAACTTCAACGAAATTCTCAACAATTTTTGGACTGAACACAGGCACTAGTATCTTTCGACGCCGTCTCCAAATGGTAACCGGAGCGAAGATGCCTCCATATCCTATAATTTTCCTAATAAAACGGTGTAGATCGTCCTTTTCAAGACACACCTTCAACACCATCTCAACATCCAAGGGATTTAGTCCCAAAAAGTAGAGTATATGATTTAGCCACCCTTTGAGGAGGCCATCGCGTTCCATTGCCGCATAACTATAATGCTGCAAAGCAGCCATAATGTCTTCCGTATCTCCCATCAGCGTGTGCGCTACTCCAATAACCGGTAGCTCTTCGTCTGGTCCCGGAATAAGAGATGCCAACTTGTACATTCTTCTCCTTCTGATCCTGTACATCGTAATCCACAGCCCTAGCACTACTAGTAGTAACCAGAAAATCATTTTGCACTTTATTTTACTTATAAACCGAATAATGTGTTTCGATACCAAATAAAATATATTTTGACGTCCCTATTGCGACTCTGCGATCTGGTAATTTTTTCAGTCTATAAAACTGAACCCGAAATGTAATCGTAAATATAATCGAATATACTCTTTTTAACAAAAAGCTCCTGCTTCAAGACTCTACTGAGCAGTTTGCAAAGTTATTTAAAAATGTAACTGCATATTTGCGTGGACACTAAAATGCGTACGCGACATGTTTGGCGCACACGATCGGTGCTCGCGTGAACGTGAGATGAAAAACCGCACTGTTTTTATACGACAATTTTCCTGCACCTGAGTGTCATTGTTCACGCACTAATTCGCAGCTGGTAACGTTGCCAGGTCGCAAAGTTCACGGTGCAACTCGCAGCCTTCGCAGACATAAGGAAGATTGTCATAGTGGTCACATTCATTTAACATATAAAGTTTGTATGTAGTATATAGACACATGGTCACATTAATGTAAATAACAAAACAAATAGGTATATATGCGCTAACAAGACAAAATTCCTAAAAGTATAATAAAACTGTACCAAACGTAATAGTAACAAGACGCCCAAAATTACACGGTAAATACTAAAACATATTGTTAATGGAACCACAATTTTATTTAAGATATGTACTTCGGGGTATGCGGTCCATGGATAAAAAGCAACTATTATATACTTTACAACAACCCATTTCCCAATTTATATTATACTAGAGGCCGCGTGAACTAGA

>SinsCYP41

GCTACTCTGTAGAACACGTAGATAAAGTATGCATGTCTCTTCGCCAGCAATATGGTAAATGTGTCAAAATGGCCGGTCTGCTCGGTAGACCCGATATGCTGTTCGTTTTCGACGCCAATGAAGTTGAAAGGGTTTTTCGAGGAGAAGACGGTACGCCGCACAGACCTTCAATGCCATCTTTAAATTATTATAAACATGTTCTGAGAAAGAATTTCTTTGGAGCTGAAGAAAATTGTGCTGGAGTAATTGCTGTACATGGCGATTCTTGGGCAGCGTTCAGAACCAAAGTATCGAAAGCGGCTCTGAGTGCCGGAGCCGCGGCGCAATATACAGAACCCGTTAGTGAAGTGGCTGATGCATTTGTTGACAGAATACGAAAAGTAAGAAATGAAGAACTAGAGACTCCGGGTGACTTTTTAAATGAAATACACAAATGGTCACTGGAATCTTTAAGTCTGATAGCACTGGACACACGTTTGAATTGTTTTGAAGCCAACGATGGATCTGAGAGCCAACGTCTAATAGATGCTGTCAACACATTCTTCCTGTGCGTCGGCGAACTCGAATTACGAACGCCATGGTGGCGATTGTATCCCACCGCTATGTTTAAGCGATACGTGGCAGCGCTTGATACCATCCTCAGTGTAACTCTGAGTCACGTAGAGAAGGCTCTTGAGGAGTGCCAAAGGAACGGAGGCAGCAAGTCCTTGCTACAGGATCTGGTGACCGCGGCTGGTCCCAGAGTTGCCGCTGTAGCCGCACTAGATATGTTCCTCGTCGGAATAGATACGACATCGAATGCAGTTGCATCAACCTTATATCAATTATCGCTAAGACCTGAACTTCAGGAGCGATTGTATGAAGAAATTGATAATGTTTTACATGGACGACCTATGAAATCCGGTGACATCAATCAGATGCCATTATTAAAAGCTTGTGTTAAAGAAGTTATGAGAATGTATCCTGTAGTAATTGGTAATGGACGTCAATTGACCAAAGATACAGTTATTTGCGGCTACAATATACCGAAAGGAACACAAGTAATATTCCAACACTACGTTATGGGGAATGACGATGAATACTTCTGGAATTCATCCGAATTCCAACCAGAACGTTGGCTACGCAGATCGTCGACTCAGAAACATCATGCATTCGCGTCTTTACCTTTTGGTTTTGGAAAAAGAATGTGTCTCGGCCGTCGATTCGCTGAACTTGAAATACATACAGTGATTTGTAAGATGGTGCAAGCATTCAAAATGGAATATCATCATGAGCTTATGGATTATCATGTTCATCCCATGTACACACCCAATGGACCTATACGTATAAAACTGATTGATCGGTAATCTATATATACTACCCGCAATAATGAAAGAATACAGGGGACTGACATGTAAGATGGTAAATTTTTGAAAATAAAAAAATATTTCGTGTGATTATTTTGTATCGTCGAGGACCCAAAATAAAAATTGTTAAAATTATTGTCTTATATCCAGTCCGTGGGAAAACATTCAAACCATTAAACCAATATTAATCTTATTCATGTTTATTTTACTTTGCGCGTTGAAACTTTCAAGGTTTCAACATAATTAACCCTGGAATCACAAGGCA

>SinsCYP42

CTCATATAATAATATTGTCTCTGTCGCTATCAACACATTTGTTATTCAGCGTTCAACTGCTACACACACATTTTCTGTTCATGAACAAATTATTTCTAACAAAATTTGCGTGATTACAAATATAGAAAAAGATGATTATTTTAACGTTAGTAATAATATTGGTGACGCTTCTCTACAAATATGGTACAAGAAATTTTAAATATTGGTATGAAAGAGGCGTGAAACACGACAAACCCATACCGTTTTTTGGTAACAATTTCCGACAATTCACACAGCAGGTAAGCCTAACTGATGTATTCACGGAACAGTACAAGAAATATCCCAATGAAAAATTCGTAGGATTTTATTCGGCAAATGAGGCTACACTTATTTTGAGAGATCCCGAACTAGTGAAACAAGTTCTGGTGGCCGATTTCCATTATTTCTATCCTAGAGGATTGAATCCTCATAAAGAAGTCATTGAGCCTCTGTTGAAAAACTTGTTCTTCGCCGATGG

>SinsCYP43

TTATTTTCTTGGTGTAAATGTAGCGTGAAACCCGTTCAGAGATGACGCTGATATGGCCATCGCTAGTCTGAGATCGTTATATGTGTTCAAGGAAGTTATTTTGTATTCCCGCAACACTTTTACACATATTGTCTTCACTAGTTTTGTTCCGAAATATCTACCCAAACAATCCATAGGTCCTAAACTAAAAGGTATATAACAAGTCGGGATACGTTGCTTCACATTCTCTGGACTGAATCTTTCCGGATCAAAGTCGTTGGGTCGCGTCCAGTAGCGGGGGTCGCGATGAAGGTGATATATTGGAGCAACTAGCGAACATCCTGCTGGCAATGTTCCTGCACTTATGACAATATCCTCCGTTATAGTTCTCTGCAGCAACGTTCCAATAGGGAAAAGACGTAATATTTCTTTGAAAACCATATCTAAATATGGCATTCTCTTCAGATCTTCTTCTGTAATTGGTCTATCTTGATCACCAATTACGTCTTCTATTTCTGCGTACAATTTTTCCTGTGCTTCTGGATGGTAAGCAATCATTAATAATAAGAATGACGATATCTTAGCTGATGCTTCTTGACTCGATGTAAAAATTGTAAAGGTCTCTTTTAATAATTCTTCCCTTTCGAGTTCTTCTGATAATATGAATCTGTCTATAACACTCAACTGCGTATTGCTCACTGAATCTTCTTCTGTGTTCAAAAGATCTGTCTTATTGTATTGTTTTATTGTTTTAAGTGTCTCCATTCTATGCTCTACGATTTTTTTACTAAAATGTGTCATCATTGCAATAAAATTCTTCTGCTGTTGATAGGAACTTGTTAGCCAAAATACAGGGTCAAGCTGTAAATACCACTTCGTCATTCTGGCGAACACTGTGTCATAAAGCCTGGGAGTCTCATCAATGATGTGTTGTAGATACGGTAAACTCATTGTCTGTTCTTTAGATAATCCCATTAACGTTTGACAAACAGTATAGCTAGTTGTTTGAACGATACATTGATAAAAATCAATCTGCCGACCGTTTGGTTTCATTCGAAACTTCTCGAGTAAAAGGTCCGCCTCTTTGTTAAATACGTCGCTGTAGTTCTCGATGGCCCTTTTGCCGTAGTTCGGAGTGGCGATCTTGCGATGTTTACGCCAACATGGTCCAGATCCACTTAAAATCCCACCACCAAGGCAATCTGCCATATATTTGTATTGTGGACCTTTCACAGTAGTTTTTTGGCTCGAAAGTAAAACCTTAAGGTCGTTTGGGTCCTTAACAACAATGTTTAAGTCTGGTCCGAGCCAAAAACTTGTAACATCCCCGTACTCGTTCAGAATGTCGTCAATTACTTTAATTAAATCTTCAAAATTACACATGAAAAGTAAAGCGTTTCCCACAAGAGGTAACGGTTTCGGACCAGGCAATTTGGCGGCGATAGCCAGCAACTTTTTATTTTTGACCCGCCAATGAATCCAGAACGATAGCGCAAGGAATACCAAAGCTAAAAGATGCCAGATCATTCTCTTAAAACTAAAGTATTGCACACAAGGAAACCCTTTCGACACTCGACAGCGACTATTCGGAGTAAAG

>SinsCYP44

GAAAGAAATATCATTTGATGTGACAACACAGGGTTTGATGGCTGTGATTGATGGAATGTGGCGCGATAACATATGATGCCATATTCCTTGATTGTTATTGGCTAAGAATTTGAAGCTATACTGTGTAATTATTAAAAAATTATTCAACCTGCAATTCGACTGCGTGAACTCTAAATCCTTGCCCATAACAACAATAAGTATAAATAAGCGTGCCCCAGACGCTCTTGGTCGGCTATCTCCATTCTCATTCAAATATCCATCTCATAAATAAATGTCATGCTCTTCGTTCTATGCTGATATGGTGACCCGAAACAGGCTTCAGAAGAATGTCCAACTTGAACCGAAGCTGACTGTGATCAGATAAGATCCGATAGTGGCGAAGCAGATGAGCGAGAGTTGCTTTCATTGACATCATGGCGTATATTCTACCGATACACATGCGTCTCCCGAAGCTAAATCCAGAAAACGCGTTAGGATTTTCTGGAAGGGAAGCTGGATTCAGCCACCGCTCTGGCCTGAATACTTCGGCGTCCTCTCCCCACATCTTATGGCGATGCACACCATGAATAGCCAGAAAACAGCTACTGCCAGGGTGCAACGTATAGTTTTTAAGTTTCACTTCTCTCTCGACTTGTCTCGCGACGATTGGAGCTATTGAATGTATGCGAAGCGATTCTTTCAACACCGCTTCTAAATACATCATCTTCGATAGGTCCTGTTTTTCGAAATCTCGATCTGTTTCTACGACTTCTTGTATCTCTTTAAAGGCCTTATCCTGAACCTCAGGATGTGATCCCAATAGTATCAGCGTATACATAAGGATAGTCGCAGAAGTATCGTGCCCGGCTGCAATCATAGTGTCCACCTCTTCTCTGATTTCTCTATCAGTGAATGCATTCTTCTCCACTGTTAATTCTAACAGTAAATCTAGGAACGCCTTACATTTATTATTCGACGTTTTTTGTCTATTTATTTCGGAAAATACATTTCCGTTTAATTCTAATTTTCGTCTTTGTAGTACCGAATTGGACATTTCATGCAAGACTTTGACAACTCGTTCTTCTCTCCTTTTCTGGTTGGTCCATTTATAGACAAAGTGATTATGCATCCAGAAGTTCTGGAGTCTAATGGAGAAGATCTCCAATATTTCTCCAATGGCAAACACGTAATTGTTATTAAGAATGCTGTCTTCTCCGATGTTAACACCCATTGCTGTTAAGCATATTGTTTCCAAACTATTATGTCCCAAATAATACCACTGATCAAATGGTCCTTTTCCTACGACAGTTTCCAACTCTTTAACCAAACGTCTTGATTGACTGTTGAAGACACCCAGAAAGCCATCCAGTACATGCTGACTGAAAGCTGGGTTGATTAGTTTACGGTGATGTTTCCATGTTGGCACAGTAGCAGTAACTAAACCCTCGCCAAGCCAAGAGTTAGCGAAACTATACACGAAATCTTTTTTCAAGCATGTGTTAGCTACCGTCAAGCAATCGTCTGGGTCTGTTATCACATAAACTTTCCGTGTTCCTATCCTCATTAATATAATGCCTCCTGCCTTTAAACTCTCGTATACCGCTTCCCTTATTACTTTCCATAAATTAGTACTATCACCCATCAACATATAAGCATGACCCAGGAGCGGTAAAGCACCGGGATACATTGGGGGTTCAGATATCGATGCAACTCTTCGCCGAAGCCATATCCACCAGGCAAGACCACCACCACACGCAAACATACAAATTAATAGCAACACTAGAACAGTCATTGTTATTATTTAACTATTAATTGGAACCCTAAATATATTGTATATCATTATTAAAGAACACAATCATTTCACATGTATTTTCATGAATTTATGATAAAATAATAAATAATCATTCAATTTTCATAAATTTTCTATTCAAAATTAACAAGGATGCGTCCATTTTTCTATATTATTAACAATCAATAATTTAAAAAATAGATCGAATTTATTAAACAAAAGTATTTCCAGAGATATTATAGGCTGGACACGTATATCTTATAAGATAGAGGTTGCGCTACTGAAAGTACTTTATCGTAACGACAATTTAAATACTGGTATACAGGGTTACTGGAAAGGTTTCCCGATCCTTTTAAGGGGTAGT

>SinsCYP45

AAATAACTAAAATTTGCGTCTCTTTTCAACTGTATACAAACGCGAGTCGCGTTGTAAACACTTATATAGTGCTAAAACTTCTTTTATTAAAAAAAAACAGTTTTTAGTGTGTGAAAGAAATTGAGCGTTAAAACAGTTACCATGCTGTTCCTATTATGGACAGCTGTTTTACTCGCAGCATTGTACCTGTATTTCCATCAGCGCTACTCCAGATTCTCTCGGCATGGAGTCAAGACAGTGAAGCCTACACCACCTTTTGGTAACATGGGCCGAATTACACTCCGTATAGAACACTTTGTAGACATGCTTGAACGGCTTTATAATGAATACCCAGATGAAAAGTTCGTCGGGATGTTTGAATTTGTGAATCCAATGGTGTTAATCCGAGACATAGAGCTGATCAAACGAATTGGCATTAAGGACTTCGATTCCTTCCTTGATCATCGCGTCATAGTCGATGAAAATATTGATCCGTTTTTTGGAAGAAATTTATTCTCATTGAAAGGTCAAGAATGGAAGGACATGCGTTCAACGTTGAGTCCTGCCTTCACAAGTTCCAAGATTCGTCTGATGGTGCCATTCATGGTTGAAGTCGGTGATCAGATGATTCGATCACTTAAAAAGAAAATTTCTGACTCTGGAGTCGGCTACTTAGACGTGGATATGAAAGATATATCAACACGATACGCCAACGACGTGATCGCTTCGTGTGCGTTCGGCCTCAAAGTTGACTCTCACACGATCGAAAACAACAAGTTCTATGAGATGGGCAAAAGGGCGAGTTCATTCAACTTCTCACAACTCCTTAAGTTCTTTGGTTACACGTCCTTCCCTTCCTTGATGAAGCTTTTCAAGCTATCTTTATTTACTAAAGAAACGAAAGACTTTTTTACTGAACTAGTGACTAGCACGATGAAAGACAGAGAAACGCATAATACAATTCGCCCTGACATGATACATCTCCTGATGGAAGCAAAAAAAGGTCAATTGACCCATGAGGACAAGACTGCGCAGGACGAGGACGCCGGCTTTGCTACTGTTGAAGAATCTTCTGTTGGCAAAAAAGATATTAACATAGTATGGACTGATATTGACCTCATAGCCCAAGCGGTGATATTCTTCGTTGCTGGCTTTGAGACTGTATCGTCCACTATGTCGTTCTTGTTACACGAACTGGCTTTCCACCCTGAAGTACAGGCGAAGCTTGTGGAGGAGATACGTGAAAACGACAGAAAGAACGGTGGAAAATTCAATTATAACTCCATACAACAGATGACGTATATGGATATGGTGGTTTCAGAACTGCTAAGGCTGTGGACTCCAGGTCTTGCGTTAGACAGATATTGCAATAAAGATTACAATCTTGGCAAACCCAATGACAAAGCCACTGAAGATTTCATTATCCGAAAAGGCGAAGCAGTGTCCATCCCTGTATGGGCCATCCATCGAGACCCAAAATATTTCCCGGATCCCCTTAAATTTGATCCCGAACGTTTTTCTGAAGAAAACAGACACAAGATCCAACCTTTCACCTATTTGCCTTTCGGTATGGGACCTAGAAATTGTATAGGGTCAAGGTTTGCTTTATGCGAAGTTAAGGTTATGGCGTATCAACTACTTCAGCACTTCGAACTGTCTCCGAGTGAAAAGTCTTGTCACCCAATCAAGTTGTGCAAGAGTACCTTTAATATTCGCATCGAAGGTGGCCATTGGATTAGATTGAAAGTCAGAAACTAAAAATTTGAGACTAATTATTTCAACACCCATTTTCAACTACCTGCACACGCTGAATACCTAGTTACCTGAGACCGGTTAACTGAGATGAAAAGTATGCTGTATACCTGGAAGGCTAATCAGGACAATAAATTCTGGAAACTTCAAAATGACATCTCGGAGGTCAAGAAGCAGACCAATAATATTGAAAGAATCAATTCCGATATTGAGAAATCCATTGATTTTTTGGCTACTCAATATGAAGATATCAAGTGCAAAATTCTTGCTCTTGAAGACCGTAGCAAAGAGTATCGCCAGCAGATCCAAAAACTTGAAGACAAAGTTGAAGAAATTTGTCGTCAGGCTTGCATGAATTTTATTGAAATAAGGAATGTGCCCTGTAGAACCAAGGAGACCTAAGAAAATCTTCTACACATCGCACAGAACCTAATGAAAACAATTGGTTTTAGCGTTGCTTCGTCCGATTTGCGCGATGTCCGACGTCTATCAGGAAAACCAGAGTACTCCAAACCTATCATTATCAACGTTTCCTCAAATGTAATCAAAAATAACATTCTAAAGGCTGCTAAGTCCTTTAACGTTGCCAACAATACGAACAGACTAAATTCGTTGCATGCAGGCATTGAAGGACCGCCCACGCCTATTTTTATTGCTGAATACTTGACCCCTAAAGCTAGGAGATTACATTTTCTGGCGAGAGGTCTTGTTAAAAACAAACTGTATCGATATTGTTGGACATCTAATGGAAAAAATTTCCTTCGTAAAGATGACGGATCTAAATTGATTACTATTACTGACGAGGAACAAATTAACATGTTAAAGGCCCACCAGCAACAATGACTAGTCTCCATTCTGGCTTGTTTACACCCTGATATGCTTTTTATCCTTGGGTATCTTATTGTGCATCTATGTTTTTATATCCCTATTGTTTTAATTTGCATTATCATTCGGTGTTATATTTATTTATATGTTCATATTGTAACTCTAATTTTATGTTATTCATGTCTTATGATTTATTGTATTTTATTATTTTTAATTTTAATTTATAGTGATTGTAGTCTTTTTGTTACTTATGACCACGTAACGAGAACGTCGAGTGAAGTTGAACACAGCTCAGGAATCCCTTTGTGCGATTCTAGCAACCTTACTAATGAACACTTAAAATGCTTCTTTTTGTTGTTATCTTTTAGATGCTGCCTAGTATACTATTTGTGTCTCTCTCCTTTATCTAATAAAATTTACAGGAGAAGATATTATTAAAAATATCGATACAATATCAGTATCTGATTCTAGCATATGTGATCCCGAACAATGTTATTTATCTTTACCTAATCCCGATAAGTATCTTAAAATATTAACACAGAATATTCGTAGTGTATCCTGTAATATACCCGGTTTTATGGCACTCCTGTCACGCATAAATATAAACAAGGATATTGTGGTATTAACTGAATGTTGGCTCTCGTGCTCTCCTAATGTTCCCTCTCTTGATGGATATACAACTTACAATAGCTCAACTACGCACAATCAAAACGATGGCATAGTCTTATATATTAAAAATGGTCTAAATTTTATAGTCGTAGAACCTAAATTTATGGAAACAAATTGTTTAATTCTAAAATCTAATTTATTTAGTACAGCTGTCATAGCGATCTATCGTCCTCCTTCATTCAAGAATATTACTAATTTTGTTCATTCATTAGACTCCGTATTAGTAAACCTAAAAAACTTTAAAAACATTGTTCTGGTTGGCGATATCAACTTGGATATAAAACCTAACAATGATGATCCCCGTTCCAATGAATACCTTAATCTAACTGCTTTCCACGGACTCCTGCCCGCTCATGTATTTCCCACTAGAATAAACAAATGCCTTGACCATGTCCTATTAAAAACTAAAATTCGAGCTTTAACTTTTGTTGTTCAATCTTCGCTTACAGATCATGATGCTGTACTGACTTGCTTGACCCCCAAAAATAAGTTTACTATTCAATCCACTTCTTACTCCAAAATTAACTATGAAGGCCTTGTCTGTGATATTAGTAATTTAAATTTCGAACCAATTTACAATTCTTTGGATGCCAATTTTGCTGCTTCTTACTTTGTTAAATCTATCTCCCATGCATTACAATCAAATACTATTACATTTTCGATACCCCGTCGTCGTTATATCATTAAACCATGGATAACCCCTGGTTTACTACGTTGTATACGCACTAGGGATAAAATGCATCTAAAACTAAAAAAATCTCCTAACAATGAAATTCTTAGAATTACCTATTTAAGGTACCGTAATTTCGGTAACTCTCTATTTAAAAAAAATAAGCATGCTTATGAACGGGATAAACTGCAAAAAGCTGGCACTAATGGTAAACTACTATGGAATACTATCAAAGATATAACTAACTTTTCACCAAAATCTAAATCTTCTGCCATTAATTTAATTACCTCTAGTGATACCCCGTTGTGCTCTGTTAATAAAGTAAATGAATTTTTTGCGGGGATAGGACAGACATTGGCGGAGAAAATTGTCAGCAATAGATGTATACCCGTTCTTGCGGATGAACTTGCCGGTTCTAATTCTAACAATAGTGTCAACTTCTCTTTTGGACTTTTGGAGACTGATGAATCTGAAATAGAGCGCTTAATTATGTCGATGAAGACAGATTGTGCAGTTGGCTGGGATGGCATATCTGCTGGTACACTCAAGCAGTTCAAATCTATTTTTATTCCTCCCTTAACCTATATTTTAAATTTATGTCTTTCTTCTGGAACTTTTCCTAAAGACTTTAAAAAGGCTCTTATTCACCCTATCCATAAGAGTGGGAATCAAGACTGTCAATAATTATAGACCTATATCCGTTTTACCTGCACTATCTAAAATATTAGAACGCCTAATTAACTTTAGACTGGTTGCATATCTCGACGAGTTTGACCTGTTCTCAGTTAACAAATATGGCTTTCGTTCTCGTAAATCTACAGCTGATGCTGTATTGACGCTAACAGATTACATCGTTAGAGAGTTGGACTCTGGCAAGAAATGCATTACCACTTTCCTCGATTTAGCTAAGGCTTTTGATACCATTTCAATTCCTCTCTTGTTGAAGAAACTCGAAAGCATCGGTATTCGGGGAATTGAACTTCAGCTCTTTCAGGATTATTTAACTGAAAGAACGCAATGTGTTAGGATAGGTGACATTGTCAGTAGTGACTTGCCTATCTCGTATGGAATATCCCAGGGCAGTATTTTAGGTCCTAGTCTTTTTCTAATATATATTAATAGTTTATGTAACATCACATTGACTAATGGTAGAGTCACATCTTTTGCTGATGATACAGCCCTGACCTTTACTGGGCACACATGGGCAGAGGCTTTTCATTATGCCCAGGACGGCTTTGATTTGGTTCGTTTGATATCACCTTTTCCTTAAGAAATCAATCTCTCCCTTTTCCGAAATTATTTTCTCTAAATGCCCATCATTGCTCTTCTTCTTTACCTAGTATATGTTCTTGTCCAACTTTAAATAGCACCCCTGCTATTAAATACCTGGGAGTTGTTTTGGATAATAACCTTTCATTTTCACGGCACATTAGTCTATTAACCAACAGGGTTAGAAAATTAATTTACGTTTTTAAACATCTTCGTCATGTTGCAGATTACAAAATTATTAAGATTATATACTATGCCCTCTGTCAGTCTCTAATTATTTATTGTATTACTACATGGGGTGGAGCTGCCAAAACTCATATGCTGTCACTTGAAAGGGCGCAAAGAGCATTGCTCAAAGTAGCAACTTTTCGTCCCTTCTATTATTCAACTAGTGTACTATACAGAGACTGTCAAGTTCTTTCAGTACGCCAGTTATTTGTCTTATACATCACTCTTAAAATGCATTCCACACTTACCTTTGACCCTAAATTTACTCTAAACAAAAGAAGAATCCATTCGGTCTGTCCAACTAAGTCCTTCCACAAAAATTTCGTCAACAAACATTTTTGTTTCCTTGCACCTTACATATACAACAAGATTAACCAAGTTCTTTCCATTTTTGCTCAAACTAAATTTCTTTGTAAAAAAACAGTTTCCAAATGGCTTCAAGACTTAACTTATGATGAAACCGAAGCTATTATATGTCTAAACAGATAACATCATTTATGTACTATCTCATTTACTATATATCGCATGCATATTTAGCTCTGTTCAATTTTACTACATCACTCATTTTTTTTATGTGCAATTTTTGTTTTATTTTCTGTTTTATTACTACATATTATTGCTATTATGATTATTA

>SinsCYP46

GCGATTAGTGGCCGGTAAAAGGTACGATCTAAAAGATGATCGTCTCAAGAAATTGTGCGTTCTCATTACACGATCTTTCAAAGTGGTAGATATGAGCGGAGGGATATTGAATTTTATGCCTTTTTTGCGATATATTATACCAAAATATATTGGATATACGGAATTAAAAGAAATACATGACACTTTATACAAATTCCTAAGGGAGACCATAAAAGAGCATCGGAAAACCATCAATGTAAAAAATCCAAGAGATGTCATCGATGCTTTCCTAATTGAAATGATAGAACTGAAAGATAAGGCCTTTACAGAGGAAGAACTACAAGTAGTCTGTCTGGACTTATTGGAAGCTGGTGTGGAGACAGTGAGCAACACAGCCGTATTCCTTCTATTACACGTGGTACGGAACGAAATGATCCAGAAAAGGCT

>SinsCYP47

AAAGCAGTCATCGCCCCATACACTTATCTGCCTTTTGGTACAGGACCAAGGCATTGTATCGGGTCACGTTTCGCTGTGACAACGTCCAAGGTGTTCATTGTGAACTTCTTCAGAAAGTACCGCGTGCGCGCCTTGCAGTCAGCGGCCCGGCTCGCGCCTCGAGCGTTCATATTGAGACCAGCCAAAGGATTCAATCTCCTTATTGAGCCTAG

>SinsGST1

GACCCAGTTGCCGAATGATGTAGCTCTGTATTTCCCTCGATATCGGACCTAGGTGGCCGAAACCCGACGCGATGCTGTTGATTTCCACCTGTTTCCATGAACAGTACGGTGTGTGCTTCGCGCACTGGTTCTCTGTGTGGGGACATCTGGATTCCAACATGATGTCCGAACGCAGCATGCCAAGTGATATCGGCTGTGCTATACCTTCCTTTCGAAC

>SinsGST2

GAGGATATGGTCTTCGTCAGAGAGCGTGTCGCAAGTGGCGGCCGACCGAAAAGTACCGACTTCCCTTGTGGTGGAAGGCCACCATCAAATTTCGCCTGGCTAGCATTTATCTTCTTACTAAAGTCGGGCCTCAAAAAAAGTGGAGCTCTCTTTAGCGGAGTAAGAGAACTCTTCCTTTCGGCCAGTTCCCTCTATATGAAGAGGGCAATCGTTCCTTGAACCAGTCGCTGGCCATCGCCCGCTACCTTGCCAGCCAGTACAAACTGCTCCCTTCGGATCCCTGGGAGCAGGCTGTGTTAGATGCTGTCGTTTATAACATCTACGATTTTTGGGGAAAGGTGCTGCCTTTTATTAAGGAAACAAACCCAGGAAAAAAGGAAGCCCTCAGAAAGGAAGTTCTAGAAGAACATGTAGATTATTATTTCTCTAGATTTGAGAAGACCTTAAAACAGAATAATGGCCATTTTGGTGGCAAGTTGACATGGGCTGACTTCATCCTCGTCAATATCGTTGAGGCAGCCAACCTCTTCCTGGATGCTCAGATCGAGACGAAATATCCAACGGTCGTGGCTCTACTCCAAAAAGTCCGATCGTTGCCTGGCGTCAAGGAATACGTTGCAAA

>SinsGST3

TTTTTTTTTTTATAAACAATCCCTCACGATTTTATTAACAATAATAACAAAATGCATATTAGTACAAAACATTTTATCTTCATACAACTCGTAAGAATTATAGAGCAGTGATATAATATAACACAACTTGCACTCCCATGTACACCATGATGACGAAGGGAATGAAGAATGCGATCGCTCGCGATGGCTGCGGCAGGGGTTTCACAGCGTAAACTATTGTGTGCAACACGCGCCCGGCGGTGTACACGCGGAACAGCAGCGTGGCCCAAGATGCTACTGGATTTGTGGTTAAGTACAGCGCGCCCAACACCCAAAAAGCTGGAATGTTCTCCAAATCGTTGAGATGCGCACGCCGTATCCTCTCCACCGCAGGGTCATCGAACTTCACCTTTCCTTTCTTGAATGATTGCACATCCTCGGGGTTCGCGAACACGCCTCGCGCCATACGCGTCATACCGGTCAAGGGACTCAAAAATAGAAGCTTTAAAGCCAGTATTGCAGAATATACTATGTACGATTGAACAACGGGGTTATCTAGTGATATGACGGCCATTTTTGCCGGCTTGTTTATTCGAAACGACAGATACTGACGTCTATTCGGTTCGCTAGTCACAGCGAGAATAACACAATAACATGATTAACTATTCGAACACCAAGTGCTCGATAACAAGAGTAAACGAGTTACTTATTTCGGAGATTATGATTTAAATACATTACACGGCTCTCCTGACTTTTATTGCTGTCTCATTTCGTCTTTTATGCCCAGTAATGTATATGAAACACGGCACTAAACCTTTGATAAAACTGATCACATGTTTTATAATATGATAGAGGGGTAAATG

>SinsGST4

AAAACTCAATAATTTATTTGTCCAAAAATTTATTCGTGAAACATATTCCGTTAACCACACTAAGCTTCGCTTTTTTCTAAGCTCTCTTTAATGACTTTAACCAAATCGTTCAAACCCGGTACATTGCCTTTCTGGTACCAATTTTCGCCTTTCAGCCGATCGAACCAAGTTCTCAACTTCACATATTTCTCATCGATGGGCACTATGCAATTCGAAGATGACACACTCGCTACACACGACACGTCTGCTAAAGTCACATGATCAGTCACCAAATAAATGGATTTCTCCAAATATTTCTCCAAAAACCCATACGTTTCTTCTATATCTTCAATTAATTTAGATTCAATTTTCTGTCCTCTAAAAATTGGAAATGCAATAGCACGTAATCTTGGAAACAATATTGAAGTGTCGAAAAACAATCTCTGATCAATAGTGGCTCTTAGTTTTAAATCGGTAGGGTACAATTTTTCCTTCATATCTTTGCCATATCGACTTACTAAATAGGTTATGATAGCATGACTATCACTAAGTATGAAATCTCCATCTTCAAGCAATGGTACTGTGTGTATTGGATTCTTTTTAATATATTCTGGTTTAAGATGCTCACCAGCAAACGTATTTACATCTATAGTTTGCACTTCAATTCCAAGAATTTCAATTGTCATCAAAACAGCCCTTACTGGTGGACTTCTATCAACTTTATACAAAATTATAGCCATGTTTCAAAACAGGCATTGATTAGACAAAATTTTTAGTTATATAAGGTATTAATTAGTTATATAAGGTATTTATTTACATATATCTTATCTTTGTAGTCTTTAAGTTAATTTAGTTTTAGATAAACCACTGTTGTCCAAGAGTTACTACTCACCATTATAATCTCAAAATCACTAATGTGTTCCAGGATAGTCAGTCTTAAACTGATTTATACAGCACAATAACAAACAAGGGATTTGGTAAAGCAATTATTTAACAAGCGATATGCGTTCCGAATCGTAGACGATGAACTCACGACTAAAGGTAGAGGTAAGTGCGCACGCGCAGATAATAT

>SinsGST5

CTATTGAGAAGTACTGTACCTACCTATAATATGTAGCGAGCAATTATATTTTAATCTGTGAATAGAGACTAGAGAGAGTAATTGCTAATTTTGAGGTATATAATATTCTTATCTGTTATTTATTTGTAATAATAACAAGACTTATTGTATTTATAAAGAAGTACACAAACGCCGTCAAAATTACTTCTTACTACTCCAAATACAACGGAAATATATCCGCCATGGCCAAGCCAGTACTATATTCGTACTGGCGAAGCTCATGCTCCTGGCGTGTGAGGATCGCGCTCAACTTGAAAGAAATTCCATATGACATCAAGGCGGTTAGCTTGATTAAGGGTGGAGGAGAACAACATTGCAATGAGTATCGAGAAGTCAATCCTATGGAACAAGTCCCTTCTTTGTGTATAGATGGACATACATTGGTAGAATCATTAAACATAATGCATTATTTGGAAGAGACAAGACCTCAAAGACCGTTGATGCCTCAGGACTGCTTCAAAAGAGCTAAAGTCCGTGAAATTTGCGAGGTTATATCATCGGGTATCCAACCGTTGCAGAACCTCGTAGTTCTGATCTACGTTGGCGAGGAGAAGAAGAAAGAATGGGCCCAGCATTGGATCACGAGAGGGTTTCGAGCTGTTGAGAAACTGCTCTCCGTTTCGGCTGGGAAGTATTGCGTTGGCGACGATATCACTTTAGCCGATTGCTGCTTGGTACCACAGGTGTTTAATGCTAGAAGGTTCCACGTAGACCTTCGCCCGTTCCCGATAATCTTACGCATCGATCGTGAACTGGAGAACCACCCCGCGTTCCGCGCCGCTCACCCCTCGTCGCAGCCCGACTGTCCGCCCGAAGTCGCCAAATGAACCCACCAACTGTGGCCCACATGTTGGCCATACATTCCATCAACCGATATATTATAACATTTTTTTGTACATACACATAATGACGCGTTCTCATCTGTTCTATTCTATCGGTCTATAGCTTACATTGAGTCTTTCTAATATCGATGTGTAACATCCTGTATTGGCAATAATTTCGTAA

>SinsGST6

CGATGATAAGTGAATATCCCTAATTCTCATCACAGAACACTAAATTCCACTTACCTACTTATCCATAAAGTTCATTTATGAGAAAACACTTTCTTTATCTGTTTCATAGAGTACTACAGTTCATCACCATTAATCACTAATCAGTTTGCTATTGGAAAGGAAACTGGTAGTAAAAATAAAGGACGATCGAATTGGTATAATATTGCTTATAGGAATCCATCTTTATTATTTTAAAGAAGTTTTAAACAAAATTGTTGTTTGTTCGGTATAGATCTTCATAAATACCAGGATGTCAGAGAAACATTTACAAACCGGTGATGTGCTACCCCCATACAATGGGAAGATACGGCTATATGCAATGAGGTTTTGCCCTTATGCTGAACGGGCAGTGCTCGTGTTGAATGCAAAAAAATTGGAATATGATTTAATATTCATTAACTTGGATCATAAGCCTGAGTGGATTTTCAACTTTAGCCCTAGAGGTACTGTGCCAGCACTCGAATATGAACAGGGGAAGGCTATTTTTGATAGTAACATTGTAACTACATATTTGGATGAAAAATACCCGGAAAATCCCTTACAAGCTTCAGATCCATTACGTCGGGCTCAAGACAAAATTATTGTGGAGAATTTTGCTGCTGCTCAGTCGGCCTACTATACCGCAGCATTCAACCCTCAAGCACTAGAGCCTTCGTCGGTGGAGAAATTCCACAAAGGCCTGGAAGCTTTCCAAGCGGAACTCGAAGGGCGTGGCACTAAATTTTTACATGGAGATGAACCTGGATGGGTAGACTACACCATATGGCCATTCTTGGAACGTTTTGAAGCTCTACCCTTGCTTGGAAAATCGGAGTTTGCGATAGACAAGACCAAGTATGAATTATTGGCGAACTATATAGAAGCCATGAAGAATGTGCCTGCCGTCAAAGCTTACTATTTGTCAGCAGAAACACACGCCAAGTTCACGGAATCTCGCAACAAAGGCGACGCCAACTACAACATGTTAGACACCAGCGCCGTCTGCTGCATGCGCCCCAGGAAGAAGAAAGAATAAACAGAACAACATGTTTGTTTTTAACTGTCACTGAACTGAACTAAATTTAACATTAAATCTCACTGAACATTCGATATTTTGTAATATTACTTATTATACTTTTTTGTTGCAATAAAAGTACAAGTACACTTATAAAAAAAAA

>SinsGST7

GCTTCCTTTGCTCCGCCGTAGTCTGAATAATATCCTATCTTATTAGTAATGGATTGGGAAACCAAAAAACAAACACTGAATAGATTTCTTTACTTTTAGTTTCTCTTGTCTAAAAACATTTTAAAAACAAACAATACACCTAGTGCTTGTAAAGTTGTAACATAACGCCGAATTGGAAAGTGGATAAGCATATAGAATTTTAAATCCTCCTGTGTTTCATGTTTACTCGTCCAAACTGTGTTATCTACCACTATGTATGTATGTAGGTATACGGGTATGACTCGTTAGCACGAGTCGACACCCTATATGAAGCGGCTATCGACGGAACCCGTTAGTATCACGCATTTCATTCGACCACAAACAATGTCTCGTGTTCAGACTATTACCGTTTTTTTATTCTGTTTTGTTTTTATTGGCGAATATGCGTTCGGTAATATAATACAAACCAGATGGAATGCCGAGGACGTTGTCCAACTACTCGACGTTATTGGAAATGACGCCGAACTGCCCATGCCTCGTAATGAAGTAAACGAGGAACAGGATTCTAAGACGCAGTCACCTATTGACGTCGGCGCTAATCTCCAAACTGACGAGAACAGAAAATGTGCTGAAATAGGACAGTTTTGCATGAATCACAAAGATTGTTGCTCTAACGCGTGTTTGGGATACATGAAGAGGTGCGTCTCGGGGAAAAGTGACGAACCATGATGTGATGACAATACGTTACACAGTTACACATCATACTTAAACTATTTATTAATTTTACGAGCTGGATGTATGGGATGATTCAGATGGGATAAGTCAGGCGGATTCGCTGCAAAGTTTTGTATCTCTTTCATGGCTTCGTCTGAAATTTTCCATAAGTTAGGATGATTCTTCTTAAAATCATCATACCATTTATGTATCTTAGCGTATTTTGAGAAATCAAATCCAATTGCCTCAAGGGTCATTGTAGAATTTATAAGAGGGAAGTCGGCAATGGTCAAATGGTCTGCTGCCGCGTATTTAGTTCCCAATCTCTCTAAATATGTATTGAAGACGTCCAAAGCTATGTTAACTTTCTTTAAACCGAGTGGCGTACGTTCGTAGTCGAAAAATATTGGAGCCATTGTATATGCGGATATGTTTGCATAATAACTAGATAAGTTGAAACACAGTCGTTGATTTACTATAGCTCTTACTTTAGGATCTTTAGGATATAAAGGAGATTCTGGTTTGTATTTGTCACAAATGTATTGCAAAATTGCATTACTTTCGCTCAAGTAAAATCCATCATCATCTAATACAGGAATTTCTTTCTGAGGATTCATCAATGCGTATTCTTCAGTCATATGTTCACCGGCTCCATAGTTTACATCAATTTGTTGAAATGGAATCTCTAGTTGTGCAAGGGCTTGACGTACAGATAGAGATGGTGGACCATCTGATACCACATACAGCTTCAAAACCATTTCGCCCAATCCTTTAATAATAACTTATTATTAACGTAAGTCTCTCGTTTTATATGTAAACCTTCGATGTTTCTGTCGAAATGATTGGAAATCCCAGTTTGCTATAGATACTTAGGTAGTACGAAACGTCTACAACCACTGAGACAAAAACAAGAACTGCCGGCCGGCGCCCACCGCCTACGTCGCTTGTACAATAACTTACACGGCACACTCCTTTCACTTAG

>SinsGST8

GGCATAGTTAAAGTTTATATCGAGGAGTGCAGAATGCTAAATGAAATATCAAACTTAAAAAATAAGTATATATATAAATTAATTACGGTCGAATTTAGATCATTCACGGGGCATTTAGTTAGTTTTAATGACTGACGAAATTCATTTAAATTATTTGCAAAAAAAGAGTAAATATAACCTAAGTGTCCTACGTTGAAAGTGTTTGTTATCGACAGAATCCGTGTATTGAATTAGGTTATGTATGTTGCGTCATTATTATTGCTATAGTGTTTGGCATTATGATAGGCTCAACGTTAAGTTTCGCTGCGCATATGTTGATCTCGTCATTTAGAATCCTGGGCCCGGTCGCACAGGTGTTGGAATACGTGTTGCCAAAATATCACAACATGGCCACTAGAGCGGTCAAGGGCAAAATTAATTTCAATACGAAACATTTGAAGAAAGGCGATCCTCTGCCGGCGTACAATGGCAAGTTGCGTATATACAACATGCGGTACTGCCCGTACGCGCAACGAACTATCCTGGCTCTGAATGCGAAACAAATCGACTACGAAGTCGTCAACATCAATTTGGTTGACAAGCCTGAATGGTTGACCAGTAAAAGCGCGTTTGGTAAAGTACCAGCACTGGAGATCAAGGACGGGGTAACCATTTACGAGAGTTTAGTGACGGTGGAGTATTTAGATGAAGTGTACCCACAAAGGCCATTATTACCCAAGGATCCAGTGAAAAAAGCCTTCGACAAGATAATAGTTGAAGCTACGGGACCTATTCACACCTTATTTTACAAAATAATTCGTGCTCCGGAAACGATAACCGAAGATAATATTGCTGGGTATGTCAAAGCCCTAGATTTTGTACAGGAGCAACTTGAAAGTCGAGGCACAACGTTTTTAGACGGCAATGAACCTGGATTCGCCGATTACATGATCTGGCCATGGTTTGAGAGGATTCTGGCATTACAAGATGACGATGACAGGACGAGAATTGATCCGCAAAAATTCAAATTACTGACGCAGTACTTAAACAACATGTTGCAAGACCCAGCAGTGAGTCAATATTTGGTGTCCAAAGAGGTCTTATTTAAAGTCCTGGAAGGTTACAAACCTGGGAATACGCCCAATTACGACTTACTCACTGAAGATTACTAAAACCAACGACTAAAATGAGAATAATGTCTTTGCACATCTGTACACGTTCTATTTGTTATAAAACATACAAAAGATAAATGCATATTACTTATGGGGTCGGGCAACAGTTTTTTTTTACAATATTAAGTTGTTTATAATGCAAAATATCAAATCAAAATCGATAAAAAATTGCCAATATATGTAATTGTATTTAATAAAATTCATTTGAATATTAAAAAAAA

>SinsGST9

TTTTTATTACAAAATCAGCATTTTATTTATAAGATAAAAACTCCTCCCCTTCCCCGCTTTTAACCCCTTTCCTTGAGATCAGCTATATCAAAAGCCTGCGTTTAAAAACTTAACTAACAATAAAGCATCAACTTTATAAAAGTTTATTATGATTACCGTACCTACATCAGATATATTTCACGTAGGTATACAATTTAGGTATATGCCTAAATTCGTAAGCTTTTGTGATTAATTAATGGCAAGCAATAATAAGCAGATAATTCTCTAAATACATTCTATTTAGCTTCAAAAAATTTCAAAAAACAGCTTAAATTACTACAAATGCTCAAAGTTCAGTCTTGGCTTTAAGTTTGGCGATCATCTCTTGCATCTGTTTAATTCCCTTCTGATTAGCATCTTCATATCCTGGCGCTGTAGATTTTACCAATTCGAACCATTTTTCAACATTAGGGTATGGGTGCAAGTTGATCCCAGCGGCTTCTATAGTGGAGACCGTAGCGACCAGCGCCAGGTCAGCCAACGTGAGGTCTGCGCCAACAGCGTATTTGTGTCCCTCCAGGAAGGTGTTGAGGAAGACGAGTGCTTCCTCTAGTTTCTTCAACTGGGCTTCGTCTGCTGGCGAGCCGCCGAATATTTGGGGATAAAAATATTGAGCAAATCTTGGGTACAATGTACCCAAGTCGAAATCCAATCGTTGGTCGATCACTGCTCTCGCCTGTGGATCTTCAGGGTACAAGTTCGTCTTCGCTCCATATTTCGTGACCAGGTATCGACTGATGGCGCGCGATTCCCACAGAGAGAAGCCATCGTCTACTATGGTTGGGACAGTATGTTGGGGGTTCAGTTTCAAGAATTCAGGCTTGAACTGTTCTCCGGCCATGAGGTCCAGCGGCTTGAGGGCGAGCTGCAGGTCGAGCGCGGCCGCCACCAGCAGCACCAGGCGGCACGGCGCCGAGCCCGCCGTGTAGTACAAATCGATCGACATCGCGCTTATAATTACAATAAAAACGACAAACACGTATCAATGTAAAAGATAAACTGAGACAAACCGGATAGAAATATTGACCTTGAAACCATTTCATTGCCTTATTACACAGCCAAATTTAGAAGATAGTCAATAAATGTTGTTACAGAGACGCTATTGAATAACCATTCGGATTCGATTTGTTTCAACTTGGGATATAGGTACATTAAAATATTTACAACCACAAGGTAGG

>SinsGST10

GTGAGATTCAGTTATCTTATTACGATACAACCGGACAGTGTGGCAGTCTGGCGACAGTATATTGCCTATAAACGGACCTGTTACAAGTTTTTAAATTAATACTTTAAAGTTTAGTTTCGTAAATATATGGCGCCGAAACTTTATAAAATGGATGCGAGCCCCCCGGCACGCGCTGCAATGATGGTGTGTGATGTTCATAATGTACCATTAGAAATGGTAGACGTGAACTTAATTGAGAAAGAACATTTAAAACCGGAATATTTAAAGAAAAATCCACTACACACTGTTCCGATGTTTGAAGATGGAGATTTCATTCTACAAGACAGTCATGCTATTTTGATATATCTAACAGAAGTATACGGCAAAGATGATTCTCTATATCCTAAAGAGACAAAACAAAGAGCTTTTGTAAACCAAAAACTTTTCTTCAACAATTCCATATTGTTCACGAGATTACGAAATATAGCGTACCCAGCAATAATGGAAGGTGTGAGAAAACCGTCAGAGAAACAATTAAACGATATCAAAGAAGCTTATGGTTTCTTAGAAGAATTCTTTTCACGTACAAAATATTTGGCTACAAATCATATAACGATAGCGGATATAGCCGCATTTGCTACAGTTTCGTCTTTGATTTATATTGTCTCACTGGATAGTAAGAAATATCCTAGGACACACACCTGGTTAAAAGAAATGGAAAAACAATCTTATAGTCAAAAATACAATGCACCAGGTGTAACACAAATAGGAGGCATGTTGCAAAACTTATTAGATCTCTGAAGAACGGCTAATTTAGAATATTATAACTTATGTACATCGAATGTTTTCTATGAATATGTCTAAAATAAAAGAATATCGTACAAGAATGACGTCCTCAATAGGGAAGGATTATATGAAAAGTGTTTATTTTGAAAACATATTGTCAAAAATCCATAAACTATACAATGTTATCCATTATTAATGTTAAAATATAGATTTTTGTTCTTATATAATTATTATTTTTTATTTTAGTTACTTCGATTTGAGAATTCTTAAAGCGTAGGTAAGAGAATGAGGTTCACTGCTCTTGACAGAGTTGATCTACGCTAAATCGATTGTAGAAAAAACCAGTCAAATGCGAGTTCAACTCGCGTGAAGATGGTTCCGTCCGTCCACTATAAACTGTATGTACTCACGTAGGTACTTAGTGATTGTCAGTTTATACTGGACATTGAAGCGAACCGAAATGAAGCAAACTATGGACTTCTTATTATTTTTCCTGTA

>SinsGST11

ATCTGTGTCTGTGATATTTATTAGATATATAACCCATAGGCACAAAGTACATACATTATTATATGATATATCCCTATAAATACATTCACATCTGTCCTGTGACACAGACTACTCCTGTGATATTAATTTTGTATTTTGCCAAGTTATGTTTATTGTATGACTGTGTTCTTTATTAAATTGTTTAGGGAAAGAGAGTATCATTAATATTACCTAACTGAAGACTTCCAACGTAGCGGAAACTTACATTTTTGTCAGACAAATAGGAACTATGGCGCCAATTCTATACAAAGTCGACACCAGTCCTCCCGCTCGAGCAGCAATGATGGTCGCTGATATTTTGGGTTTGCAACTTAATCTACGGGATCTAAATCCTGTGCTAAGGGAACAAGATACACCAGAATTTACAAAGAAAAATCCAATGAGGACAATTCCCATATTAGATGAGGGAGATTACAGCTTAGCAGATAGTCACGCCATAATGCTTTACCTTATCGACGCTTACGGGAAGCCACAACATTCTTATTTGTATCCCACTTGTAAAAGAAAACGTGCTACTATCAATCAGAGATTATTCTTCGATTGTGGGGTCTTGTTCCCTACTTTGAGGGCTATAATGGCACGAACATTTTTTGCAAAATTAACACATTTAGATGAACGCATGATTTCTAACATAGAAGACGGCTACAGAATCCTGGAAGCATACTTAACAGAGACAATTTTCTTAGCGGACAATATTATGACAGTCGCCGATATAAGCGTCGTGGCAACTGTATCCACAATGGACGGAATACGTCCAATAGATGAAAAGAAATATCCAAAATTGAAACACTGGTTAATGACCATGTCACATAAAGATTTCTGCAGGAAAATCAACGCGCCTGGATGTGATCAACTCGTGACAATACTCGGAAACTTTATGCAAAACAATACAGAGTTAGTTAGTTCGCAAAAACAGAAGGCAAAACTATAAATTAAAATTTATTATTCAAATTTGGATATTTTGTTTATAACAATGGGCATTTTTAATATACATATAACATATTTATTATCTCATTGAAATATAAGTATTTACTTCTTTTTGAATATATTACGTATTGCCTTCATTGGAATCCTGATGTCCACGGCTTCGCCTATGAAAATGTGCTAAATAGTATAATAAAAGATTTTGTCTATGTAATACATAATTTAATATTTAGTTCTTTTCCATACAAACTTTCAACGTCCCATCTTACCCCTTTAGAACTTGAATTTTAAAATTCGGGTAAATGGGTTGTTCATTTATTTATTTTATTATATTAAGAGGTTTTCCTTTTACAGAATCATATATTTTAATTCCTTTTCATACACACTTTCAATCCCCATTTAATGAAATCGCTTAGGGGATGAATTTCCAAAAATCCTATTGCGTGCCAGTACATGAAGACATCTACTGTCCCGAGTTAAATCTGACTGCAGTAATTACGGCTCTCCATCGATGTGTTTTTTTTTTCAGGCTGACAATTGCTGTTGGCCTTGAGGACTTTACAGCGTCACCGGACGATATGGGTCAGCGCTTATGGCGTTCAAGCCGAGAATAGCAACTAGCCAAAGGGTGCCACCTAGAGGTGGCTCGGACCTCGACTTAGGATGTTGGTTTGAGGGGGGCGACCGATTGAGTTGTCGACAGAGGAGGAGACGGTCTGTAGGTAGGCGTCCGTGAGACGCGGGTGTGAACGCGAGCGCGGGCGAACTGATCGGGCGAGTGGGGTCCCTCGTCGTCGCCGACGGCGACGGTGTCCTTTGGGGTGTGTGCGGGCGGGCCCTGCACGGGCGGAGGCCGCTATC

>SinsGST12

ATACTATAATAAAATGGTAACCGAGCAATCTGTTTTTCTGTACATTGTACAAGGTATGACGGGAAAAGCGTTGCATGGTTTTGAATTAATCTCGGTACACTGATTTAAAGCTTATTTATACCCTGGATACAATTTAATGACTTCTCCAGTTTTATTTCAAGGGTTCTTGACTTAAAAGAACGCCTACAAATTAGACACTTTAAGAAGCGTTGCAGCGTCTTAAAGCGTCTAATTTGAAGGCGCCATAAAACGAATCCCAATTTTGTCCAAAATAAGACACATCGTCTATTAAATTGAGATAAAGAAAATATAAAAAAATCTTTTACAGGTAATAACCCATGGAATGCGCTCTTGCATTCTTGTAATAATCGAAATATTCAGCCGGCGATGTGGCGTGTTTCTTTACGACCTCGTCCAACTGCATTTGATCGCCCCAATCTGCAAAGTTAGGGAAGAGTGATCGTTTCTCGTCAAATTTTCTCTCATTGACACATCGCAAAAGGTACAGCCTCTCCACCCATGGCCAGATCATGTAATCCAGCATCCCCGGACGGTCGCCGCCGAAGTAATTTGTTCTTCTAGAAGCCAATTCCTTCTCAAATATATCCAAAGTTTGGACAATTTGTTCGCTGCCAAAAACGAAGTTCGTGTCGAAACACTCCAAGCTGCCTTTTATTAGCTCATTAAACCTCTCGATGAGAAGCCGATCTTGGGCCTTTACGTAAGGATCTCTAGAATGGAGAGGGTTCCGTGCGTATTTCTCGTCCAAATAATCACATATCACCACGCTCTCGAACAAATATTTGTCGCCCTGGTCCGTGGGTATTTCTAAAACTGGAATCTTTAGTCTCGGATTGTTGGCTCTGAACCATTCAGGTAAGCGTAACGGGTTCAGTTTGTAGACTTCGTAACGAGCTTTCTTGGCCTCCAAAACTAGGAGGACCCTATGACCGTATGGGTTCATTTCTACATGGTACAAACGGAGTTTGTCTTGGAGGGTCGGCGGTGGGATTGCACCGGCGGTTCTATAATAAGTCATTGTTCTTGAATTAAATATTTTATATTTTTAGGCGAAACGGTCCTGTTTCCACGGATTCCGGTGTACAAATACCGAATAACGTCACATTTAAAATTACTTCGTGCTATGACATCATTCTTTTG

>SinsGST13

AAATTGTCCCATATATTATTATTAAATTATTGTAAAATGGTTATACTAATTCAGAAACGTGCGCGAGCGTGCGCACAGCAACAATTAACCAAAGTTTCATCGTTATCGGATGAATGGTATAGGAACGCATACCCTACAAAGAAAGAAAGAAAGAAAGAAACACATTTATTTCAACTGCATTATAACATAATATACAACAGACTAGTTGTGCCCGTATGTCGGACGGCACTTAACGCCGTTAGTTTCAATTTTCATCATGTTTATACATGCGGAAATTTTTCGATGGAAACCATGAGTGGCTTGATAAAAAATTCTGATGATAAAAAATTCTGACAGTATCAACAAGGTTGGCCACTGGGCTAAAAGCCGCATTACATTATTCAATTTGCAGAGCGCTAGTGAGCTAGCATGCTACATTATGTATCGTGTAAAATTAGTTAGTTAGTGATTCAATATAGTGTGATCGTATGGGCTCTGCCACGCTCGCTCGTCTAGTGCACAAGTTAGCGCGCTAATCTAGCGATCTGCGAATTGTATAATGTAATGCGGCTTTTACAAATCTCCGCAGCATCGCACTGGGCCCTGTGGTAAAATATGGCCCACACCACCTCATGGATGAGAATAGCTATGCCCGTAAATTCGTTCACATGCCATTTGATTTATGTACATGGACAATCGTCCATGTAAATTTCAAATTAGCATATCTTTTTTGTCCCGAATGCCGACAGCGATGAAACAAAATGGATGCTACTCCGACCTCGAGAGAATACATCAATGTAAATCTCATTAAAATACTTTTAGCAGGTTTTAGGTTATACGTGCACAAATAAACAAACAAAAATTACAAATGCCGTCTTAATACCGTTTATTTTATTTATTTACGTCCCCCAAGAGTTTTTCCCCTAGTCTAATTACAGATGAGCATTATTCAGTATTTTTTTATGTACAATTCTTTATTTCCTGATAATCCGTTTTCTACTGTTATAACTAATTGTAATTTAGTGTTCTACAAGTATTTTCCTCTTTATAGTCTAAAATTGCTTATATGCTCATTCCCCAAGAAAGAGGTTGTCCTAATATAGGAGGCAGAGTACCACATTTTTGTGTTATTTTCGGATAAATAAATAAATATTTTATAAATATAAATAGAAAAATTATGAATTATGAGTTACTTTTCTTCGTGTTAAGGGTGAAAAACGAGTTGAATTATCTTAAGCTTGACCAGACTAAAAAATAAAAATATCATAACTTTATTGTGAGAATTCAGTGTAAATAATAAACTACATAAAATAATGACAAGAAAGATAAAACCAGCTTATATTACTAGTGGACGTTATAAAATAAACACCGTAAATTATAACACAAAAATGGTTATGGCTGCATTGTCAGAATGACTCGGCGTTTGATTATTCCCGCCCACCGTTACTATGGTTCCCATAATCATAACAGATCTGTTCATAATAATGACAGAGTGAAACTCAGTTGTTCATTAGATTACCCGTCATCAGTACAAACGTCGCGAATTTTTCTCAAAATTCACTATAAATAAAACCACATTTCAGTCCCTACTTCAGTTATTATAAAACTGGTATCTCGTTCGATCATAGAGTACCTACATTTTTTGAAATAGCCTAGGATATTTCTAACTGGTGCATCGTACTTTATGTGACAATCAATAATTTTCGTTACAAAATTTATACTAAATAATTTATTGTTAAGTCGATTGTGATAAAAGTCTATAAACGAAAATTTATTATACTTACATTTAGATATCTATAAAAAAAAACTGTAAGAATTTAAGAAGGAAGGAGAAACAACTTTAAAGACGTTAGAGTTTCAAAAAGTTAGTCAAACTGGGGCAATGCATGGACAAAACTATCGTGGAAAAAATTCATACTACGAAGTGAGTTTGCGATAAAAGAGATACAATTCAATAATATAATTATTCTTTACTTTAAAATTTCTAAGTGCTTAATTGAAATTCCTTGTGATTTTTGTGTATTTTGTACAATTCAGTGATATATAGATATACAATAATAAGATATTCTAGGCGTTGATATTTTTGAAAAGGCCGTCTAAAGAGGTTTAAAAATGAAAATCTTTTTTCTTTTTACCCTATTTCTAGTATATGTCTCTAGCAACGCGGCTGCGCGTTCTAGGGGTAAAAAAATGCCAGCCCAACCGATAAAGTTTTATTATCTGCCTCCTTCGCCGCCATGTCGAGCTGTAATAATGACAGCGAGGGTTCTTGGTATCGATTTAGACTTGATTGTGACCAACATCATGGAAGGACAACATATGACGGCGGAATATCTCAAGATGAATCCCCAACACACTATACCAACTATGGATGATAACGGATTTATATTATGGGAAAGTCGAGCAATTATGACGTATCTCGTGAGCGCTTACGGAAGTGACGATACTTTATATCCCAAGAATGCCCGATTGAGAGCACTCGTCGACCAACGGCTGTATTTTGATCTCGGTACCTTGTATCAGAGATATTTGGATCTTTATGCGCCTATTCTCTTCAAAGGAGAAGAATACGACGATGAAAAGGCTGACAAGTTGAATGAAGCTCTGGACTGGCTGAATACTATGCTCGAAGGCAAAACTTTCGTCGCTGGCGATAATCTTACCATAGCTGACATTTCAATTGTGGTGACCTTCACCAATTTAGAGGCTCTTGACTACGACCTCAATGCTTATGACAATGTGAAGAAGTGGTTTGCGAGAACAAAGAAGGCTTTAGAACCGTATGGTTACAAGGAGATCGACCAATCTGGCGCACAAATATTAGCTTCATTATTGAAGAAAAATTAAAGTACAATAAATTATACCTTTATTATTTACTTAGATTGAAATTATTTTAGATTGATTTATTATAATTTAAATAAATT

>SinsGST14

AGGACATTCACATCAGTTGACTCGCTTACATATCGTCGTCTCAACTCCGATTGCTCTGTGGATTGAAATTTTCAATTAATAATACAAAAAAAAGGAAGAGTAAACTATGGTGTTAACCCTATACAAGCTGGACGCCAGTCCACCAGTTCGTGCCACGTACATGGTCATCCAGGCTCTCAATATTTCAGACGTAGAATACATAGACGTCAATCTACTCGAGTATGAACATTTAAAAGAAGACTTTTTGAAATTAAATCCTCAGCACACAATCCCAACATTGAAAGATAAGGATTTCGTTATCTGGGACAGTCACGCGATCGCTGGTTATCTGGTGAACGTGTACGCTGGAAACGATTCTCTTTATCCAATCGAGCCGAAACAAAGGGCAATTGTTGATCAAAGGCTCCACTTTGACAGCGGCATCCTCTTTCCTGCATTACGAGGAACTGTTGAACCTGTCCTCTTTTGGGGCGAGAAAGCTTTTAAGTCAGAGAATTTAGAGAAGATAAGTAAAGCATATGGTTTTGCTGAGAAATTCCTCGCTGACTCACCGTGGCTCGCCGGCGATGCTGTCACCATCGCTGATATTTGCTGTGTGGCAACCATCAGCACCTTGAATGAAGTACTGTCCATTGATAAAGTCACGTATCCCAACTTGACCGCTTGGATGGAACGTTGCTCTGAAGAAGACTTCTATAAGAAGGGAAATGTTCCTGGATTGAATATATTTCGACAATTGATAAAGAGCAAAATTCTTTAAAATATGAAAGCGATTTGGAATTTTGTATGGAGAGTGGCTTGATTTTCTTTTTCAGCGACCGCTATAATAATTTAAATACTGTCCAATACTACTATATTTAAAACACATGGCTGCTAAAAAAACTTTAACCTAAGTAATATCGAAGCCGAGGTATTTGATACCAAAGTTATACTTACTATACGTAGGTAAGTAAGACCCGCGTTATATACAACTCAATATAATGCAGATTATAAATGTTTTTGTTGCACGTGTAGTGCTTTAATATATTATGTTCTAATATAAAATACAAAAAGTTATGTCAAGTGTATTATATTTTATCTACTGTAAAAAAGTCGTAAGTGGCCGCAGACTTGAAAAATCGAACAACTTTTACCTAAACCCCTTTGTTACATATCGGCTTCATAGACAAACATAGTGCAAATCTCATAATTATAATTTCATGTTTCTTTTTATCACTGTATAATATAATAATAATATTTATAAAAAATTTGCTACTTAAAGTATCAAATAATTACAAAAAATACATCCAATCATCACGACCTAAGTAGATAACTCTACTACGACATCTATATAATTATAATTATTTTAATAATAATTCATTTATTCTCTCGTTAACTCGGAACATTATACAAAAAGCTTATGAAAAACTCAACGAAACGAACTATGAATATATTTATTAGAAGCGATTTTTTATTTTACAATTTTTTTTTTTCAAAAGCACATAATAATTTAACGCGATCATTTTGATTGATTTCTCGTGAAATGTTTCATCAATAATGACTTTGAAAAATGCATAATTATATTATGTGTATAATTCATAGTCACTCTTAAATAAAGCTATTGTACTATTTCACTC

>SinsGST15

GAGGGGTGAAATAATGAAACAACTTACAAATTTTCTACTTTCGAAAGTACGTTTTTGTTTTATGCAAATCAATTCTTAAGGTTTTATAATAATGTCTTCCATACAATTGTATCATTTCCCTGTTAGTGGACCTTCTCGTGGCGCATTATTAGCTGCCAGAGCAATCGGAGTCCCGTTACAAATTGAGATTGTAGATTTGTTCAAGAAAGAACAGCTAAGTGAAAGTTTTTTGAAGATTAATCCACAACATTGCGTACCCACATTGAACGATAACGGCTTTATTCTATGGGAAAGTCGTGCTATCGCATGCTATTTAGCCGACAAGTACGGAAGAGATGACCAGCTTTACCCGAAGGATTTAAATCGTCGCGCTATTGTAAACCAGCGACTATATTTTGATAGTTCGCTTTTGTATGTGAAGATTAGAGCTATTTGTTTCCCCATACTGTATCTAGGCGAAACTCAAATTAAACAGGCTCTAAAAGATGACCTCAATACCACTCTTGGCTTCCTTGAACACTTTTTGAGTGGTAGTAAATGGGTAGCAGGGGATACTATTACAATAGCAGACACATCTATATATGCATCTCTTTCCAGTATTCTTGCAGTCGGATGGGATATATCTAGTTTTCCCAATATCCAGCGTTGGATCAAAGAATGTGCTATTTTACCAGGCTATAATGAAAATGAAGAAGGAGCAAAAACATTTGGAGAAACTGTCAGAAAGAATATTAAGCAATAAATAGATTTTTTTTTGTATAAAATATTTTTCTTTATCATATAGTTTATTTAATGTTAAACTTATTATATTTCTTAAAAATAAATGAACATTTACACACATTTTTAAATATTCTTTCCTTTACTCCCTATAGAACTTCAACACTAGCTGTTGCTCATTCCTTACTTTTCTTTTGATAACAGTTTCTGATTTATATCCATGTGCAAGCATAATCTCAATCACTTCATTAGGAATGTTTTCTTCGATTAACAAGAGGTAAAATGTGCCATCATAAGCAAGTTTCTTTGGTATCATATCTAGTAATCTATCGGTCACTTCTCTGCCCTTTACTCCACCGGCCCAACTTGCTGTTATATCATTTTCACCACATTCCTCTGTTGCTGTAACCACATATGGTGGGTTAAATATGATGACATCAAACTTATTATCCATAAAAGATTGAGTTAAATCCATATTAACACAGTCCAATATTACTTTATTATAAATGGCCGTAGATTTTGACATAATACATGCTTTGAAATTTATATCTGTGCACATACAAAAAGTTTTTGGGAATGCCATACCAAAAGCAGTTATAACAACACCGCTGCCTGAACCAACTTCTAGACAAAATATGGGGTTTTTTGCTTTTAAGTATTGTAAATCTTTTTCTAGAGCATCTATTAGTAAGTAGCTGTCCTCAGCTGGCTCATAAACATAGTCAAAATCAGCCTTGTCTATGTGGTTCTGATATGGAGTATCCATAGTGTTAGTTGATACTTCCATGATTGGAGACAACTCCACTGGTGCCCTGTTGGGGTTCTTGCTGTGGTTCTATTATCACAGTTGTACGAACACTTTTTCCTTTATTGATCATCTGCCTAATTTCATTTAATCTATCTTGTGAAGCCTGCAAGATATTCAATACCACAGTGACTTTATGTTTACAGGCAATCAATTTCTTTACATAAGGGTCCAAATCTGGATGATTGTTCAATGCTTCACGAACTTTTTGGAGTTCTTCATTTAAGGAATCTATCTGTTGTTTTAATTCTAATTGGGAAATTCTGGTAGCTCTAACCCTGTCGTCGAGCTGGTCTACGGTGGGCTTCAAGAGCCCTAGCAGACCTTCAGCAAGAGTAAATTATGTTTTAAAATGAAAGTAACAACCATTTTTAAGTAAGATTATGCAGAATGGAGAGACGATATGGAGCGTCAAATGAAATCTCACTATACTATTCGTAAAACGAACACTATTAAAGAATCATATTACTGTTCGTCACAAGTAGCGCCCACAGATGGCGTTAAACAGAAAAACACAACCTATATATGCCCCTCAAATATAGTTATCAGAGAGTTCACTAAAGGCATTCAAGTGCATTTTTACAAACAACATTTTGGACACGACCACGGCGAATACATATTACCAAATAAATATAGAAAATTTTTAATAAAATCATTCATAAATACAGAAGATGAGGAACCCGAGGAGAAAGATTTGTACGTACAGTTTAAAACTTTAATGGAATGTATAATGTTAGACGCAGCAAAAATCAATGTGCACAGTTTGAAAATTCTCATTGAGAAAGCTCTAGATATGAGTTCTATTTTAGCCAATTATAATGAAGATGATGATATTTTGTCATCTAAAGACCCCAATTTGTTCAAGATTATAACAAATAAGGAGAAAGCTCTCACAGACAAGCAAATTACTAAAGCTTTAAATGCTATGATGAATTCAACAAAGCCAAAATTAACAAAGAGACCTAATATACTGACATCTACACCAAAAGTGGGTAATGTAGTGGTTGTGCCTTTGCCCAAACAGGAAGAATCGAAAAGAACAATAAAAGAAGTGTTGCCAGCAAAAAGAACTAATACCATTCCAGTACAAGAGGTAACACCCGCTAAACACACTAGAAGTAAATCCGTACATTTCGTTTCAGACGTTGTAAAATCTCCAAGTGTTACACTTGTTGAAACGCCAAAGAGTCAAATTAATTTTCCGGCCAAGAAAACTATATTGGAAGAGGAAATAAATAAAAATGACACAAAATCAGTTACGAATTTGCCCAAAACCAATCAAGAAACACAGCCATCCTCTTTCAATGATTCGTACAAAGTTTTCGTCGATACTCTGAACAATACGAAGACAAACATTAAAAATACATCAAAAACCACTTCCAATAAGACTGAAAAACTTAAGAAGTCAATATTAAAAACGAAAATAGGTCAATTTATGCCAAACACAAACAAAACGTCACCGAATAAATCGCCAATAAAGTCGCCAGTGGATTCAAAAACTAAAATCAGTAAAGTGACAAAATTCAGTAAAGTGAAAATTGACAAATATGAAGTGAGAGAACAAGAAAACGATTGTAATATATTGATTTTGAAAATTTAATCTGTGACAACCGAATGTAATAACAGACATTGTATAAAACTTTTTTTTTATAAACAGTAGATTATTATTATTATTATTATAATACTTTACACGCTTAATAATAAAGTGACTGATTGTATAATTAATCGAAATTCAGTTGTCTACAGACAGAAACGGCTCTTATAACGCAATCTATGACTATAGGGTTGCCAATTAATTAATAACATACCAAAGAGCATCTAACCACTACGTATACCGTGGAACAGATTAACAAAAAGTATATCTAACAAAAGAAAAGTAATATTTAATTAGCCCCAAATCCATGAACGATATTTTTTTTGCCCTTCGAGTTAGGTTACTGTATTATTAAGCGTGTTATTTATCATTATCAATTTGTTATACACATTGAAAATTTCATGTCAATATAAGATACTAATAATATTCCTACTATAATAATATTATAAATTTGAATGTCCGTTTGTTTGTTACGC

>SinsGST16

CCGCGGGGCACAGCTAGTATTTTAAATATATGAAATAACATATACGTACGCATAGGAGACATTTTTAAAATACTTCAATAATGCAACCATTTATAATATATTTTATATGAACGATACCTATAAAAAACTTTTCTTTTAACTGTAAATATAAAAAAATACTCTCGTCTTTACTCTTAGTGCTAACAATTAGAAATATTTTATTCTAAGATTGTATAAGACACAACACATATTTATATACTAAAAATTCTGTGTCAGTTAAAAGTTGCGACATTAAAATGCAACGTTTCAATAGGAAAGCATGTTACATGCAACATGTTGTGCGTTATGCTGCACTACATGTGAAGTCTCGTTTTTATCCAATTTTCGCTGCGCAAGCTGACGGGAGAAAGGATATAACGATATTGTACTTTGATGTCAAAATTTCGGATGCGAAATAAACGTTTGTAATTGATACAAACACTTATACTTATTCGTCTTTAGGTCATACACACCCCGTACCTTGTCAGGTTGTCACGCATAGCATTACATCGGAAATTCAATAATATCGCCTGATGTTGCATGCACTGTAGCATTACGTGTTGGGTTACATTTGTTCATTAATAAATGTCTGTCTTATACAATAAATTGAAGGCTCTTAGAAGTTAACATTAGTTCGACAAAGAAAAAAATATTAATTTCTAAATTTAGGTTTAGAAATCAGTTTTGGGTGCGGCATCGTTGTAGGCTTTGACCTTCGGAATGGAAAGGACCGTATCCTGAAGTTTCTTAAAGCCGGGATATTTAGTATCGAGGTGTGGCATCTGAAGCATTAGTTTGAGATAATCATACACACCGGCGAAGACAAAGTCACCCCAAGTTAACTTTCCAGCAGCAATGTGTCCGTTGTTCTTTATGATGAGCTTGTTCAGTTGGTCCAGCATGGATGGGTACAGAGTCTTACAGAATTCCTCGTGTCTCTTCGCCTTCGCCTCATTGTCCGGGTCGTAATGTACGAGCGCTGCCTTTGAACGTATGTCCATGAGAAACTCCACGTTCTGGTCTATCTCGAAGTCCTGTTCGATGTCGTCGCCGGCCAGCCCGTACTTGCGGCCCAGGTAGCGCGCAATCGGCACGCTTTGCGCGTATTGCTTGCCATCAATCTCCAGCACGGGCATCTGACCGAATGGGGTCTTAGGTTTGAACTCTTTCCACTCCTCCGGGCTGAGACGTTTGTCCTCGAATTCTTCGCCGCCATATGCGAGAAGAAGGCGTGCACTTTCGCCTAGGGCTTTCACTGGGAAGTAGTAGAACACGACTTTCGGCATTGTTGTTGAATGATATATTTCAACTTAAAACGTCAAATAACACGTCTTGTGTTGATGGTGCGTACTGTTCAACAATCAAAAACTAACTGATGGACCAAACTGCGTCGGACCAAGCTAATATCACGCGGTCACGCACGCCCATGAATACACACACACCCACATAAAAAGTATAGTAACAATTGACAGTCAACAGTCATTATGAAATTATGACTCACATCAGTGATAAATATATGTACGAATGTACGTATTACTTAATAAAACCGGTCAAGTGCAAGTGAGACTCGCACATCGAGGCGAGGGACTCGTTCAAAACAGGTTTATATTTCACAAAAAGTTTTAACACCTGGAGCCTCACGTTTGACCAGATAGTGCAGTGGTTCGTGTGATGGAGTGGTTTTGAAATATTTCGTTTGACTAGACGGAATTTAATGAAACTTGGCAAGTTAAAATGCTCCCTCGAGCCAAGCGCTAGGCCTGGCTGTTTTTTGGAGATTTTTAAATCTCATTACTCATTATTAGTAGTATCTCATCTCATTACTAGTAAAAAATAAATAAAAATACAATTATCTTTAATATCTGAACTGAAAATTTCCGCATTTCTCTAATCTTAACCAGACTCCTTTTTTCAGCTTCCGGTATATAGATAGAAGATAAGTGAATATACATATTTTTTGCACAAAACTATTGTCATTTGTGATATTACAATAAATAATTATCTCACACGCTCGCCAATTAATGTTATATAAACACCAAACATGTGTCTACAGTAAATGAAGCACATAGGCAGGAAATGTATCAGGCCATTTGGAAAGCGATGCGAAAAATTTTTTTTATCTAGACAATAGATACAAATAGTGAAGTCGTAAAAACTACAAATCCCAGAAATTCGGCCGATTTTCGCATCGGTCCTCCGTGCGTGCAATATTTAAAAAAAAGTTTTCTCTTCCATAGTTTTCTCTTCACGTATTATTGTATAGACTAAAGGTCCCGTAAATGGTCGAAATTCAGCGCATGAATTCGCATTAAATTTTAACTTTGAAATTTAAAAAACGTTTGTATGTAATTTTAAAATTTTCGCTTTCTGTACTCCTTCTCCATATATTTTTAACTATGTCAAATGTCACTCCTCCGTGCGCGTAATATTCTTAAAAAGGGATCCAA

>SinsGST17

AAAATAAATTGCTGTGGTGCCGTGACTGCCGTATATTCTACATTAAATAAGTAAATAAGTGATGGAGTGCCGAAGGCAAAGATACCCATATTATATGGCGACGAAGCATCTCCACCGGTGAGGTTCGTACTGATGACCGCTTCCGTATTGAACATAGAAATCGAGTTTCGGAAAATTGATCTTTTTAACGCTGAGAATAAAGCTCTGTTTTATGAAAAGATAAATCCACTACAAAAACTGCCAGCATTAGGCATCGATCACGAAATTATATGCGACAGTCATGCAATCGCTTTGTATCTATGTCGAAAGTGCGAGAATCAAGATCTGTACCCTAGACACCCCTG
