# Supplementary material for: Antennal transcriptome analyses and olfactory protein identification in an important wood-boring moth pest, Streltzoviella insularis (Lepidoptera: Cossidae)
Source: Sci Rep. 2019 Nov 29;9:17951. doi: 10.1038/s41598-019-54455-w (PMC6884542; doi:10.1038/s41598-019-54455-w)
Supplement: Supplementary file 4 — Supplementary Table S4 [file 41598_2019_54455_MOESM4_ESM.docx]

**Supplementary Information for**

**Antennal transcriptome analyses and olfactory protein identification in an important wood-boring moth pest, *Streltzoviella insularis* (Lepidoptera: Cossidae)**

**Yuchao Yang^1^, Wenbo Li^1^, Jing Tao^1^*, Shixiang Zong^1^***

^1^Beijing Key Laboratory for Forest Pest Control, Beijing Forestry University, Beijing 100083, China

* Corresponding authors

**Email addresses:**

Yuchao Yang: yangyc68@126.com

Wenbo Li: leonardolee24@hotmail.com

Jing Tao: taojing1029@hotmail.com

Shixiang Zong: zongsx@126.com

**Table S4.** BLASTX annotation against the NCBI Nr protein database for putative OBPs of *S. insularis*.

| **Gene name** | **Gene length (bp)** | **ORF length (bp)** | **Complete ORF** | **Signal peptide** | **Mean FPKM value** | | **Best BLASTX match** | | | | | |
| --- | --- | --- | --- | --- | --- | --- | --- | --- | --- | --- | --- | --- |
|  |  |  |  |  | **Female** | **Male** | **Name** | **Acc. number** | **Species** | **Score** | **E-value** | **Identity** |
| **odorant-binding proteins (OBPs)** | | | | | | | | | | | | |
| SinsOBP1 | 710 | 459 | Y | Y | 93.53 | 81.15 | odorant binding protein | AOG12854.1 | *Eogystia hippophaecolus* | 246 | 4E-80 | 99% |
| SinsOBP2 | 706 | 459 | N | Y | 3.95 | 9.3 | odorant binding protein | AOG12876.1 | *Eogystia hippophaecolus* | 253 | 1E-82 | 96% |
| SinsOBP3 | 967 | 672 | Y | Y | 4.13 | 6.84 | odorant binding protein | AOG12856.1 | *Eogystia hippophaecolus* | 210 | 2E-65 | 98% |
| SinsOBP4 | 2366 | 408 | Y | Y | 15641.59 | 19343.45 | odorant binding protein | AOG12857.1 | *Eogystia hippophaecolus* | 219 | 7E-64 | 97% |
| SinsOBP5 | 1005 | 441 | Y | N | 1375.34 | 914.13 | odorant binding protein | AOG12858.1 | *Eogystia hippophaecolus* | 289 | 8E-96 | 95% |
| SinsOBP6 | 845 | 543 | Y | Y | 1920.77 | 1742.75 | odorant binding protein | AOG12877.1 | *Eogystia hippophaecolus* | 363 | 3E-125 | 98% |
| SinsOBP7 | 821 | 345 | Y | N | 1.17 | 2.02 | odorant binding protein | AOG12861.1 | *Eogystia hippophaecolus* | 226 | 3E-72 | 96% |
| SinsOBP8 | 547 | 519 | N | Y | 0.22 | 0.13 | odorant binding protein 9 | ALT31639.1 | *Cnaphalocrocis medinalis* | 283 | 5E-95 | 98% |
| SinsOBP9 | 1062 | 402 | Y | Y | 2244.01 | 1892.96 | odorant binding protein | AOG12864.1 | *Eogystia hippophaecolus* | 273 | 3E-89 | 100% |
| SinsOBP10 | 1918 | 714 | Y | Y | 22.26 | 15.56 | odorant binding protein | AOG12865.1 | *Eogystia hippophaecolus* | 471 | 2E-161 | 95% |
| SinsOBP11 | 865 | 477 | Y | Y | 3.12 | 2.36 | odorant binding protein 13 | BAV56800.1 | *Ostrinia furnacalis* | 228 | 3E-72 | 70% |
| SinsOBP12 | 1478 | 960 | Y | Y | 11.16 | 7.8 | odorant binding protein 9 | ALD65883.1 | *Spodoptera litura* | 271 | 1E-83 | 50% |
| SinsOBP13 | 1456 | 444 | Y | N | 850.58 | 370.26 | odorant binding protein | AOG12868.1 | *Eogystia hippophaecolus* | 270 | 5E-86 | 97% |
| SinsOBP14 | 828 | 465 | Y | N | 1815.72 | 3163.65 | odorant binding protein | AOG12869.1 | *Eogystia hippophaecolus* | 266 | 3E-87 | 93% |
| SinsOBP15 | 535 | 450 | Y | N | 8012.47 | 11406.26 | odorant binding protein | AOG12870.1 | *Eogystia hippophaecolus* | 242 | 4E-80 | 97% |
| SinsOBP16 | 2353 | 420 | Y | Y | 24.82 | 28.04 | odorant binding protein | AOG12871.1 | *Eogystia hippophaecolus* | 272 | 8E-84 | 94% |
| SinsOBP17 | 556 | 450 | Y | Y | 4.95 | 2.44 | odorant binding protein | AOG12872.1 | *Eogystia hippophaecolus* | 239 | 6E-79 | 93% |
| SinsOBP18 | 486 | 420 | Y | Y | 1710.27 | 1421.99 | odorant binding protein | AOG12873.1 | *Eogystia hippophaecolus* | 262 | 7E-88 | 99% |
| SinsOBP19 | 1145 | 468 | Y | Y | 5.08 | 7.86 | odorant binding protein | AOG12874.1 | *Eogystia hippophaecolus* | 276 | 2E-89 | 92% |
| SinsOBP20 | 2872 | 333 | Y | N | 43.51 | 23.46 | odorant binding protein | AOG12875.1 | *Eogystia hippophaecolus* | 161 | 7E-43 | 95% |
| SinsOBP21 | 1865 | 555 | Y | Y | 3.98 | 4.03 | odorant binding protein 24 | AKT26501.1 | *Spodoptera exigua* | 350 | 6E-115 | 91% |
| SinsOBP22 | 1167 | 459 | Y | Y | 10.15 | 9.48 | odorant binding protein 14 | ALS03862.1 | *Ectropis obliqua* | 173 | 1E-49 | 58% |
| SinsOBP23 | 765 | 426 | Y | Y | 1940.99 | 1693.67 | odorant binding protein | AOG12878.1 | *Eogystia hippophaecolus* | 285 | 3E-95 | 99% |
| **pheromone-binding proteins (PBPs)** | | | | | | | | | | | | |
| SinsPBP1 | 1168 | 558 | Y | N | 2013.42 | 14242.28 | pheromone binding protein | AOG12881.1 | *Eogystia hippophaecolus* | 334 | 3E-112 | 96% |
| SinsPBP2 | 2151 | 561 | Y | N | 3348.58 | 16242.23 | pheromone binding protein | AOG12882.1 | *Eogystia hippophaecolus* | 306 | 5E-97 | 92% |
| SinsPBP3 | 2221 | 498 | Y | Y | 4130.54 | 4247.01 | pheromone binding protein | AOG12880.1 | *Eogystia hippophaecolus* | 328 | 3E-105 | 95% |
| **general odorant-binding proteins (GOBPs)** | | | | | | | | | | | | |
| SinsGOBP1 | 982 | 570 | Y | N | 48777.53 | 34361.34 | general odorant binding protein | AOG12859.1 | *Eogystia hippophaecolus* | 342 | 6E-99 | 96% |
| SinsGOBP2 | 1159 | 501 | Y | Y | 16510.87 | 9751.42 | general odorant binding protein | AOG12862.1 | *Eogystia hippophaecolus* | 339 | 2E-114 | 99% |
