# Supplementary material for: Antennal transcriptome analyses and olfactory protein identification in an important wood-boring moth pest, Streltzoviella insularis (Lepidoptera: Cossidae)
Source: Sci Rep. 2019 Nov 29;9:17951. doi: 10.1038/s41598-019-54455-w (PMC6884542; doi:10.1038/s41598-019-54455-w)
Supplement: Supplementary file 5 — Supplementary Table S5 [file 41598_2019_54455_MOESM5_ESM.docx]

**Supplementary Information for**

**Antennal transcriptome analyses and olfactory protein identification in an important wood-boring moth pest, *Streltzoviella insularis* (Lepidoptera: Cossidae)**

**Yuchao Yang^1^, Wenbo Li^1^, Jing Tao^1^*, Shixiang Zong^1^***

^1^Beijing Key Laboratory for Forest Pest Control, Beijing Forestry University, Beijing 100083, China

* Corresponding authors

**Email addresses:**

Yuchao Yang: yangyc68@126.com

Wenbo Li: leonardolee24@hotmail.com

Jing Tao: taojing1029@hotmail.com

Shixiang Zong: zongsx@126.com

**Table S5.** BLASTX annotation against the NCBI Nr protein database for putative CSPs of *S. insularis*.

| **Gene name** | **Gene length (bp)** | **ORF length (bp)** | **Complete ORF** | **Signal peptide** | **Mean FPKM value** | | **Best BLASTX match** | | | | | |
| --- | --- | --- | --- | --- | --- | --- | --- | --- | --- | --- | --- | --- |
|  |  |  |  |  | **Female** | **Male** | **Name** | **Acc. number** | **Species** | **Score** | **E-value** | **Identity** |
| SinsCSP1 | 564 | 441 | Y | N | 5.91 | 4.71 | chemosensory protein | AOG12885.1 | *Eogystia hippophaecolus* | 251 | 2E-83 | 97% |
| SinsCSP2 | 695 | 384 | Y | Y | 2466.44 | 3304.66 | chemosensory protein | AOG12900.1 | *Eogystia hippophaecolus* | 249 | 5E-82 | 94% |
| SinsCSP3 | 1332 | 372 | Y | Y | 393.65 | 355.67 | chemosensory protein | AOG12887.1 | *Eogystia hippophaecolus* | 252 | 5E-80 | 98% |
| SinsCSP4 | 1102 | 384 | Y | Y | 633.73 | 759.87 | chemosensory protein | AOG12901.1 | *Eogystia hippophaecolus* | 259 | 7E-84 | 98% |
| SinsCSP5 | 921 | 366 | Y | Y | 2.03 | 8.37 | chemosensory protein | AOG12897.1 | *Eogystia hippophaecolus* | 252 | 5E-82 | 100% |
| SinsCSP6 | 678 | 375 | Y | Y | 5346.7 | 6917.9 | chemosensory protein | AOG12890.1 | *Eogystia hippophaecolus* | 243 | 2E-79 | 98% |
| SinsCSP7 | 568 | 378 | Y | Y | 27.04 | 23.98 | chemosensory protein | AOG12899.1 | *Eogystia hippophaecolus* | 259 | 2E-86 | 99% |
| SinsCSP8 | 695 | 384 | Y | Y | 2466.44 | 3304.66 | chemosensory protein | AOG12900.1 | *Eogystia hippophaecolus* | 249 | 5E-82 | 94% |
| SinsCSP9 | 1121 | 384 | Y | Y | 9.72 | 8.73 | chemosensory protein | AOG12893.1 | *Eogystia hippophaecolus* | 247 | 8E-79 | 94% |
| SinsCSP10 | 1707 | 471 | Y | Y | 1477.1 | 1378.87 | chemosensory protein | AOG12894.1 | *Eogystia hippophaecolus* | 294 | 4E-94 | 95% |
| SinsCSP11 | 787 | 372 | Y | N | 0.36 | 0.98 | chemosensory protein | AOG12895.1 | *Eogystia hippophaecolus* | 253 | 3E-83 | 99% |
| SinsCSP12 | 2794 | 867 | Y | Y | 51.23 | 70.28 | chemosensory protein 14 | AKT26490.1 | *Spodoptera exigua* | 306 | 1E-93 | 56% |
