# Supplementary material for: Antennal transcriptome analyses and olfactory protein identification in an important wood-boring moth pest, Streltzoviella insularis (Lepidoptera: Cossidae)
Source: Sci Rep. 2019 Nov 29;9:17951. doi: 10.1038/s41598-019-54455-w (PMC6884542; doi:10.1038/s41598-019-54455-w)
Supplement: Supplementary file 6 — Supplementary Table S6 [file 41598_2019_54455_MOESM6_ESM.docx]

**Supplementary Information for**

**Antennal transcriptome analyses and olfactory protein identification in an important wood-boring moth pest, *Streltzoviella insularis* (Lepidoptera: Cossidae)**

**Yuchao Yang^1^, Wenbo Li^1^, Jing Tao^1^*, Shixiang Zong^1^***

^1^Beijing Key Laboratory for Forest Pest Control, Beijing Forestry University, Beijing 100083, China

* Corresponding authors

**Email addresses:**

Yuchao Yang: yangyc68@126.com

Wenbo Li: leonardolee24@hotmail.com

Jing Tao: taojing1029@hotmail.com

Shixiang Zong: zongsx@126.com

**Table S6.** BLASTX annotation against the NCBI Nr protein database for putative ORs of *S. insularis*.

| **Gene name** | **Gene length (bp)** | **ORF length (bp)** | **Complete ORF** | **Signal peptide** | **Mean FPKM value** | | **Best BLASTX match** | | | | | |
| --- | --- | --- | --- | --- | --- | --- | --- | --- | --- | --- | --- | --- |
|  |  |  |  |  | **Female** | **Male** | **Name** | **Acc. number** | **Species** | **Score** | **E-value** | **Identity** |
| **odorant co-receptor** | | | | | | | | | | | | |
| SinsORCO | 2583 | 1422 | Y | N | 437.42 | 472.06 | odorant receptor | AOG12930.1 | *Eogystia hippophaecolus* | 961 | 0 | 98% |
| **pheromone receptors** | | | | | | | | | | | | |
| SinsOR10 | 1578 | 1272 | Y | N | 20.45 | 24.77 | odorant receptor | AOG12954.1 | *Eogystia hippophaecolus* | 805 | 0 | 91% |
| SinsOR20 | 2977 | 1287 | Y | N | 10.28 | 260.09 | odorant receptor | AOG12903.1 | *Eogystia hippophaecolus* | 791 | 0 | 89% |
| **other odorant receptors** | | | | | | | | | | | | |
| SinsOR1 | 248 | 234 | N | N | 0 | 0 | olfactory receptor OR54 | AJF23812.1 | *Planotortrix octo* | 85.5 | 9E-18 | 57% |
| SinsOR2 | 257 | 249 | N | Y | 0 | 0 | olfactory receptor 11 | AIT71985.1 | *Ctenopseustis obliquana* | 102 | 1E-23 | 57% |
| SinsOR3 | 1349 | 1206 | Y | N | 1.88 | 2.65 | odorant receptor | AOG12941.1 | *Eogystia hippophaecolus* | 776 | 0 | 96% |
| SinsOR4 | 1453 | 1245 | Y | N | 12.45 | 7.43 | odorant receptor | AOG12931.1 | *Eogystia hippophaecolus* | 806 | 0 | 95% |
| SinsOR5 | 629 | 468 | Y | N | 30.79 | 10.66 | odorant receptor | AOG12928.1 | *Eogystia hippophaecolus* | 275 | 1E-88 | 97% |
| SinsOR6 | 1278 | 1215 | Y | N | 0.31 | 0.1 | odorant receptor | AOG12943.1 | *Eogystia hippophaecolus* | 664 | 0 | 95% |
| SinsOR7 | 1488 | 1221 | Y | N | 15.38 | 9.73 | odorant receptor | AOG12945.1 | *Eogystia hippophaecolus* | 763 | 0 | 95% |
| SinsOR8 | 1645 | 1131 | Y | N | 6.48 | 6.4 | odorant receptor | AOG12949.1 | *Eogystia hippophaecolus* | 600 | 0 | 98% |
| SinsOR9 | 1206 | 312 | Y | N | 0.49 | 0.11 | odorant receptor | AOG12935.1 | *Eogystia hippophaecolus* | 332 | 3E-81 | 79% |
| SinsOR11 | 1413 | 1206 | Y | N | 3.66 | 4.67 | odorant receptor | AOG12950.1 | *Eogystia hippophaecolus* | 788 | 0 | 97% |
| SinsOR12 | 1550 | 1194 | Y | N | 5.9 | 3.28 | odorant receptor 15 | ARO76421.1 | *Conogethes punctiferalis* | 436 | 3E-147 | 61% |
| SinsOR13 | 1495 | 1194 | Y | N | 4.84 | 1.84 | odorant receptor | AOG12904.1 | *Eogystia hippophaecolus* | 653 | 0 | 96% |
| SinsOR14 | 962 | 546 | Y | N | 1 | 0.32 | Odorant receptor | KOB79193.1 | *Operophtera brumata* | 191 | 2E-56 | 51% |
| SinsOR15 | 1996 | 1185 | Y | N | 9.12 | 8.67 | odorant receptor | AOG12934.1 | *Eogystia hippophaecolus* | 714 | 0 | 90% |
| SinsOR16 | 1436 | 972 | Y | Y | 44.87 | 15.15 | Odorant Receptor 16 | ARO70228.1 | *Dendrolimus punctatus* | 397 | 4E-133 | 59% |
| SinsOR17 | 1476 | 1215 | Y | N | 8.41 | 7.44 | odorant receptor | AOG12936.1 | *Eogystia hippophaecolus* | 813 | 0 | 97% |
| SinsOR18 | 971 | 198 | Y | N | 0.12 | 0.12 | odorant receptor | AOG12960.1 | *Eogystia hippophaecolus* | 126 | 8E-33 | 90% |
| SinsOR19 | 1449 | 1224 | Y | N | 1.19 | 1.64 | odorant receptor | AOG12952.1 | *Eogystia hippophaecolus* | 782 | 0 | 93% |
| SinsOR21 | 1294 | 1170 | Y | N | 8.44 | 7.81 | odorant receptor | AOG12925.1 | *Eogystia hippophaecolus* | 781 | 0 | 96% |
| SinsOR22 | 1445 | 1191 | Y | N | 2.35 | 3.04 | odorant receptor | AOG12923.1 | *Eogystia hippophaecolus* | 791 | 0 | 96% |
| SinsOR23 | 1783 | 1338 | Y | N | 17.07 | 17.52 | odorant receptor | AOG12937.1 | *Eogystia hippophaecolus* | 892 | 0 | 96% |
| SinsOR24 | 1914 | 1296 | Y | N | 2.05 | 11.53 | Olfactory receptor 42 | CUQ99418.1 | *Manduca sexta* | 450 | 7E-150 | 53% |
| SinsOR25 | 1307 | 1185 | Y | N | 12.7 | 13.19 | odorant receptor | AOG12927.1 | *Eogystia hippophaecolus* | 749 | 0 | 99% |
| SinsOR26 | 1743 | 1158 | Y | N | 3.85 | 2.5 | odorant receptor | AOG12935.1 | *Eogystia hippophaecolus* | 738 | 0 | 99% |
| SinsOR27 | 1412 | 1230 | Y | N | 91.11 | 56.75 | odorant receptor | AOG12914.1 | *Eogystia hippophaecolus* | 724 | 0 | 92% |
| SinsOR28 | 1791 | 1221 | Y | N | 7.97 | 7.12 | odorant receptor | AOG12951.1 | *Eogystia hippophaecolus* | 744 | 0 | 95% |
| SinsOR29 | 1655 | 1161 | Y | N | 8.46 | 8 | odorant receptor | AOG12953.1 | *Eogystia hippophaecolus* | 664 | 0 | 88% |
| SinsOR30 | 1435 | 1194 | Y | N | 1.54 | 1.38 | odorant receptor | AOG12905.1 | *Eogystia hippophaecolus* | 727 | 0 | 96% |
| SinsOR31 | 2325 | 1221 | Y | N | 1.85 | 0.87 | odorant receptor | AOG12916.1 | *Eogystia hippophaecolus* | 728 | 0 | 96% |
| SinsOR32 | 1299 | 1161 | Y | N | 1.3 | 0.65 | odorant receptor | AOG12924.1 | *Eogystia hippophaecolus* | 675 | 0 | 93% |
| SinsOR33 | 3372 | 1251 | Y | N | 13.38 | 9.51 | odorant receptor | AOG12929.1 | *Eogystia hippophaecolus* | 804 | 0 | 97% |
| SinsOR34 | 1817 | 1254 | Y | N | 65.09 | 27.88 | odorant receptor | AOG12913.1 | *Eogystia hippophaecolus* | 427 | 4E-142 | 56% |
| SinsOR35 | 2538 | 378 | Y | N | 7.15 | 2.01 | odorant receptor | AOG12928.1 | *Eogystia hippophaecolus* | 145 | 5E-34 | 90% |
| SinsOR36 | 1678 | 273 | Y | N | 5.14 | 1.71 | odorant receptor | AOG12928.1 | *Eogystia hippophaecolus* | 101 | 2E-19 | 84% |
| SinsOR37 | 1859 | 1209 | Y | N | 24.17 | 21.17 | odorant receptor | AOG12946.1 | *Eogystia hippophaecolus* | 814 | 0 | 97% |
| SinsOR38 | 2147 | 1233 | Y | N | 30.81 | 89.46 | odorant receptor | AOG12910.1 | *Eogystia hippophaecolus* | 474 | 1E-161 | 99% |
| SinsOR39 | 1847 | 1278 | Y | N | 8.92 | 9.18 | olfactory receptor 11 | AIT69876.1 | *Ctenopseustis herana* | 312 | 4E-97 | 45% |
| SinsOR40 | 3119 | 1173 | Y | N | 11.68 | 14.44 | odorant receptor | AOG12938.1 | *Eogystia hippophaecolus* | 734 | 0 | 96% |
| SinsOR41 | 325 | 213 | Y | N | 0.23 | 0.26 | odorant receptor | AOG12908.1 | *Eogystia hippophaecolus* | 165 | 3E-47 | 99% |
| SinsOR42 | 870 | 795 | N | N | 0.23 | 0.11 | odorant receptor | AOG12908.1 | *Eogystia hippophaecolus* | 543 | 0 | 98% |
| SinsOR43 | 1531 | 1203 | Y | N | 2.95 | 3.25 | odorant receptor | AOG12906.1 | *Eogystia hippophaecolus* | 722 | 0 | 96% |
| SinsOR44 | 744 | 261 | N | N | 0.18 | 0.18 | olfactory receptor | BAH66322.1 | *Bombyx mori* | 90.5 | 3E-17 | 49% |
| SinsOR45 | 1180 | 780 | Y | N | 1.77 | 1.54 | olfactory receptor | NP_001104828.1 | *Bombyx mori* | 310 | 4E-99 | 50% |
| SinsOR46 | 1496 | 1164 | Y | N | 11.72 | 5.33 | odorant receptor | AOG12907.1 | *Eogystia hippophaecolus* | 778 | 0 | 97% |
| SinsOR47 | 1622 | 1236 | Y | N | 0.08 | 9.24 | olfactory receptor 11 | AIT69876.1 | *Ctenopseustis herana* | 356 | 3E-115 | 48% |
| SinsOR48 | 1545 | 1287 | Y | N | 15.53 | 7.27 | odorant receptor | AOG12926.1 | *Eogystia hippophaecolus* | 842 | 0 | 98% |
| SinsOR49 | 1555 | 1149 | Y | N | 9.15 | 3.91 | odorant receptor | AOG12932.1 | *Eogystia hippophaecolus* | 777 | 0 | 97% |
| SinsOR50 | 2137 | 1359 | Y | Y | 14.48 | 14.37 | odorant receptor | AOG12947.1 | *Eogystia hippophaecolus* | 859 | 0 | 98% |
| SinsOR51 | 3472 | 1254 | Y | N | 0.77 | 0.73 | odorant receptor 17 | ARO76423.1 | *Conogethes punctiferalis* | 619 | 0 | 75% |
| SinsOR52 | 1836 | 1266 | Y | N | 7.71 | 0 | odorant receptor | AOG12921.1 | *Eogystia hippophaecolus* | 749 | 0 | 94% |
| SinsOR53 | 1856 | 1200 | Y | N | 6.51 | 5.12 | odorant receptor | AOG12909.1 | *Eogystia hippophaecolus* | 811 | 0 | 98% |
| SinsOR54 | 1778 | 1266 | Y | N | 1.44 | 35.89 | odorant receptor | AOG12920.1 | *Eogystia hippophaecolus* | 769 | 0 | 91% |
| SinsOR55 | 1594 | 1116 | Y | N | 16.41 | 10.14 | odorant receptor | AOG12944.1 | *Eogystia hippophaecolus* | 550 | 0 | 95% |
