# Supplementary material for: Antennal transcriptome analyses and olfactory protein identification in an important wood-boring moth pest, Streltzoviella insularis (Lepidoptera: Cossidae)
Source: Sci Rep. 2019 Nov 29;9:17951. doi: 10.1038/s41598-019-54455-w (PMC6884542; doi:10.1038/s41598-019-54455-w)
Supplement: Supplementary file 7 — Supplementary Table S7 [file 41598_2019_54455_MOESM7_ESM.docx]

**Supplementary Information for**

**Antennal transcriptome analyses and olfactory protein identification in an important wood-boring moth pest, *Streltzoviella insularis* (Lepidoptera: Cossidae)**

**Yuchao Yang^1^, Wenbo Li^1^, Jing Tao^1^*, Shixiang Zong^1^***

^1^Beijing Key Laboratory for Forest Pest Control, Beijing Forestry University, Beijing 100083, China

* Corresponding authors

**Email addresses:**

Yuchao Yang: yangyc68@126.com

Wenbo Li: leonardolee24@hotmail.com

Jing Tao: taojing1029@hotmail.com

Shixiang Zong: zongsx@126.com

**Table S7.** BLASTX annotation against the NCBI Nr protein database for putative IRs of *S. insularis*.

| **Gene name** | **Gene length (bp)** | **ORF length (bp)** | **Complete ORF** | **Signal peptide** | **Mean FPKM value** | | **Best BLASTX match** | | | | | |
| --- | --- | --- | --- | --- | --- | --- | --- | --- | --- | --- | --- | --- |
|  |  |  |  |  | **Female** | **Male** | **Name** | **Acc. number** | **Species** | **Score** | **E-value** | **Identity** |
| SinIR93a1 | 2784 | 2385 | Y | Y | 3.02 | 2.5 | putative ionotropic receptor IR93a | AST36367.1 | *Cydia fagiglandana* | 1223 | 0 | 75% |
| SinIR68a | 2107 | 2055 | N | Y | 0.62 | 0.39 | ionotropic receptor | AOG12853.1 | *Eogystia hippophaecolus* | 1330 | 0 | 98% |
| SinIR75q2 | 2299 | 1905 | Y | Y | 2.13 | 2.59 | putative ionotropic receptor IR75q.2 | AQM73617.1 | *Cydia nigricana* | 902 | 0 | 72% |
| SinsIR75p2 | 5097 | 1872 | Y | Y | 2.41 | 2.11 | ionotropic receptor | AOG12852.1 | *Eogystia hippophaecolus* | 1295 | 0 | 99% |
| SinsIR93a2 | 506 | 264 | Y | N | 2.89 | 2.46 | ionotropic receptor 93a | AZB49409.1 | *Heortia vitessoides* | 152 | 1E-41 | 84% |
| SinsIR75q1 | 1564 | 366 | Y | N | 8.66 | 7.08 | ionotropic receptor | AOG12848.1 | *Eogystia hippophaecolus* | 500 | 1E-63 | 87% |
| SinsIR41a | 2400 | 1803 | Y | N | 1.83 | 1.97 | ionotropic receptor | AOG12846.1 | *Eogystia hippophaecolus* | 1191 | 0 | 97% |
| SinsIR75a1 | 4295 | 972 | Y | Y | 1.25 | 1.29 | ionotropic receptor 75a-like | XP_028163179.1 | *Ostrinia furnacalis* | 658 | 7E-94 | 52% |
| SinsIR21a | 6249 | 2571 | Y | Y | 8.19 | 6.56 | ionotropic receptor | AOG12851.1 | *Eogystia hippophaecolus* | 1637 | 0 | 97% |
| SinsIR75a2 | 2633 | 1029 | Y | N | 7.19 | 5.88 | ionotropic receptor 75a-like | XP_028158431.1 | *Ostrinia furnacalis* | 436 | 1E-138 | 69% |
| SinsIR76b | 6322 | 1635 | Y | N | 109.58 | 74.5 | ionotropic receptor | AOG12850.1 | *Eogystia hippophaecolus* | 1113 | 0 | 98% |
