# Supplementary material for: Antennal transcriptome analyses and olfactory protein identification in an important wood-boring moth pest, Streltzoviella insularis (Lepidoptera: Cossidae)
Source: Sci Rep. 2019 Nov 29;9:17951. doi: 10.1038/s41598-019-54455-w (PMC6884542; doi:10.1038/s41598-019-54455-w)
Supplement: Supplementary file 9 — Supplementary Table S9 [file 41598_2019_54455_MOESM9_ESM.docx]

**Supplementary Information for**

**Antennal transcriptome analyses and olfactory protein identification in an important wood-boring moth pest, *Streltzoviella insularis* (Lepidoptera: Cossidae)**

**Yuchao Yang^1^, Wenbo Li^1^, Jing Tao^1^*, Shixiang Zong^1^***

^1^Beijing Key Laboratory for Forest Pest Control, Beijing Forestry University, Beijing 100083, China

* Corresponding authors

**Email addresses:**

Yuchao Yang: yangyc68@126.com

Wenbo Li: leonardolee24@hotmail.com

Jing Tao: taojing1029@hotmail.com

Shixiang Zong: zongsx@126.com

**Table S9.** BLASTX annotation against the NCBI Nr protein database for putative ODEs of *S. insularis.*

| **Gene name** | **Gene length (bp)** | **ORF length (bp)** | **Complete ORF** | **Signal peptide** | **Mean FPKM value** | | **Best BLASTX match** | | | | | |
| --- | --- | --- | --- | --- | --- | --- | --- | --- | --- | --- | --- | --- |
|  |  |  |  |  | **Female** | **Male** | **Name** | **Acc. number** | **Species** | **Score** | **E-value** | **Identity** |
| **antennal esterases (CXEs)** | | | | | | | | | | | | |
| SinsCEX1 | 266 | 195 | Y | N | 0.78 | 0 | antennal esterase CXE15 | ACV60242.1 | *Spodoptera littoralis* | 152 | 3.00E-41 | 83% |
| SinsCEX2 | 217 | 99 | N | N | 8.43 | 19.02 | odorant degrading enzyme CXE5 | AII21981.1 | *Sesamia inferens* | 120 | 3.00E-30 | 73% |
| SinsCEX3 | 1890 | 1731 | Y | Y | 1708.71 | 2424.85 | odorant degrading enzyme CXE5 | AII21981.1 | *Sesamia inferens* | 800 | 0 | 70% |
| SinsCEX4 | 2504 | 1602 | Y | N | 13.48 | 15.26 | antennal esterase CXE11 | AEJ38206.1 | *Spodoptera exigua* | 704 | 0 | 63% |
| SinsCEX5 | 2821 | 1626 | Y | Y | 16.16 | 15.92 | antennal esterase CXE18 | ADR64698.1 | *Spodoptera litura* | 682 | 0 | 64% |
| SinsCEX6 | 4002 | 1668 | Y | N | 9.13 | 8.92 | odorant degrading enzyme CXE9 | AII21983.1 | *Sesamia inferens* | 862 | 0 | 73% |
| SinsCEX7 | 2531 | 1845 | Y | Y | 5.91 | 4.38 | putative antennal esterase CXE19 | ARM65390.1 | *Ectropis obliqua* | 1068 | 0 | 72% |
| SinsCEX8 | 2132 | 1638 | Y | N | 206.88 | 175.26 | odorant degrading enzyme CXE10 | AII21984.1 | *Sesamia inferens* | 671 | 0 | 60% |
| SinsCEX9 | 2371 | 1626 | Y | Y | 3.83 | 3.33 | Odorant degrading enzyme CXE20 | KOB74700.1 | *Operophtera brumata* | 456 | 3.00E-150 | 52% |
| SinsCEX10 | 3028 | 1629 | Y | Y | 13.39 | 12.78 | antennal esterase CXE17 | ADR64699.1 | *Spodoptera litura* | 692 | 0 | 62% |
| SinsCEX11 | 4089 | 1677 | Y | Y | 50.6 | 17.54 | odorant degrading enzyme CXE13 | AII21987.1 | *Sesamia inferens* | 900 | 0 | 73% |
| SinsCEX12 | 729 | 600 | N | Y | 0.09 | 0.26 | carboxylesterase CXE27 | AEL33700.1 | *Spodoptera littoralis* | 283 | 2.00E-90 | 61% |
| SinsCEX13 | 230 | 204 | N | N | 7.08 | 13.16 | odorant degrading enzyme CXE5 | AII21981.1 | *Sesamia inferens* | 127 | 1.00E-32 | 77% |
| SinsCEX14 | 2097 | 1617 | Y | N | 1.07 | 0.69 | antennal esterase CXE3 | AMB19660.1 | *Cydia pomonella* | 704 | 0 | 64% |
| SinsCEX15 | 1632 | 1452 | Y | N | 0.77 | 0.89 | antennal esterase CXE14 | AKP92859.1 | *Cydia pomonella* | 645 | 0 | 63% |
| SinsCEX16 | 2174 | 2019 | Y | Y | 2.94 | 3.66 | carboxylesterase 3 | XP_028026249.1 | *Bombyx mandarina* | 786 | 0 | 68% |
| SinsCEX17 | 2717 | 1608 | Y | N | 119.03 | 78.67 | carboxylesterase | AQY62715.1 | *Cnaphalocrocis medinalis* | 785 | 0 | 68% |
| SinsCEX18 | 2387 | 1602 | Y | N | 4.06 | 3.5 | carboxylesterase | ABY57297.1 | *Bombyx mandarina* | 632 | 0 | 58% |
| SinsCEX19 | 2672 | 1743 | Y | Y | 29.92 | 29.66 | antennal esterase CXE14 | AKP92859.1 | *Cydia pomonella* | 659 | 0 | 60% |
| **aldehyde oxidases (AOXs)** | | | | | | | | | | | | |
| SinsAOX1 | 1178 | 1041 | N | N | 4.93 | 4.47 | aldehyde oxidase | BAR64767.1 | *Ostrinia furnacalis* | 511 | 4E-167 | 68% |
| SinsAOX2 | 515 | 507 | N | N | 3.95 | 2.86 | aldehyde oxidase AOX2 | AII21997.1 | *Sesamia inferens* | 266 | 2E-79 | 77% |
| SinsAOX3 | 796 | 777 | N | N | 0.83 | 0.86 | PREDICTED: aldehyde oxidase 1-like | XP_013136120.1 | *Papilio polytes* | 401 | 4E-128 | 74% |
| SinsAOX4 | 700 | 579 | N | N | 0.5 | 0.51 | aldehyde oxidase | BAR64769.1 | *Ostrinia furnacalis* | 317 | 3E-100 | 62% |
| SinsAOX5 | 2432 | 1221 | N | N | 32.25 | 15.42 | aldehyde oxidase | BAR64767.1 | *Ostrinia furnacalis* | 996 | 2E-172 | 68% |
| SinsAOX6 | 1807 | 1581 | Y | N | 58.12 | 31.97 | aldehyde oxidase | BAR64767.1 | *Ostrinia furnacalis* | 782 | 0 | 71% |
| SinsAOX7 | 2190 | 1644 | Y | N | 4.92 | 5.32 | aldehyde oxidase | BAR64767.1 | *Ostrinia furnacalis* | 1132 | 0 | 73% |
| SinsAOX8 | 3658 | 3462 | Y | N | 73.61 | 42.47 | aldehyde oxidase AOX2 | AKQ06146.1 | *Cydia pomonella* | 1735 | 0 | 70% |
| SinsAOX9 | 462 | 255 | N | N | 2.29 | 3.36 | aldehyde oxidase | BAR64771.1 | *Ostrinia furnacalis* | 152 | 2E-39 | 78% |
| SinsAOX10 | 4774 | 3846 | Y | N | 273.66 | 221.33 | aldehyde oxidase 2 | ARS46833.1 | *Cnaphalocrocis medinalis* | 1788 | 0 | 69% |
| **alcohol dehydrogenases (ADs)** | | | | | | | | | | | | |
| SinsAD1 | 261 | 237 | N | N | 0.65 | 2.17 | putative alcohol dehydrogenase | KOB67732.1 | *Operophtera brumata* | 164 | 3.00E-46 | 85% |
| SinsAD2 | 1217 | 750 | Y | N | 16.24 | 23.86 | alcohol dehydrogenase AD2 | AKQ06148.1 | *Cydia pomonella* | 327 | 7.00E-108 | 71% |
| SinsAD3 | 1209 | 975 | Y | N | 8.55 | 54.7 | alcohol dehydrogenase | BAR64763.1 | *Ostrinia furnacalis* | 529 | 0 | 80% |
| SinsAD4 | 1360 | 1044 | Y | Y | 24.73 | 31.01 | Alcohol dehydrogenase [NADP+] A | KPI96444.1 | *Papilio xuthus* | 476 | 3.00E-164 | 68% |
| SinsAD5 | 1997 | 1467 | Y | N | 3.27 | 3.9 | alcohol dehydrogenase 3 | AKD01741.1 | *Helicoverpa assulta* | 485 | 2.00E-164 | 67% |
| SinsAD6 | 1617 | 990 | Y | N | 1.28 | 1.32 | Alcohol dehydrogenase | KOB70117.1 | *Operophtera brumata* | 634 | 1.00E-103 | 50% |
| SinsAD7 | 1471 | 1059 | Y | N | 7.56 | 9.69 | alcohol dehydrogenase | BAR64764.1 | *Ostrinia furnacalis* | 579 | 0 | 80% |
| SinsAD8 | 1607 | 813 | Y | N | 39.35 | 47.48 | alcohol dehydrogenase AD1 | AII21999.1 | *Sesamia inferens* | 362 | 2.00E-119 | 66% |
| **cytochrome P450s (CYPs)** | | | | | | | | | | | | |
| SinsCYP1 | 340 | 294 | N | N | 0 | 0.68 | probable cytochrome P450 303a1 | XP_022833915.1 | *Spodoptera litura* | 202 | 2.00E-60 | 82% |
| SinsCYP2 | 338 | 279 | N | N | 0 | 0.69 | probable cytochrome P450 303a1 | XP_028034668.1 | *Bombyx mandarina* | 197 | 7.00E-59 | 83% |
| SinsCYP3 | 603 | 546 | N | Y | 0.22 | 0.46 | cytochrome P450 4C1 | AXP17212.1 | *Cydia pomonella* | 226 | 3.00E-72 | 63% |
| SinsCYP4 | 262 | 84 | N | N | 1.8 | 0 | cytochrome P450 6AB13 | ADE05584.1 | *Manduca sexta* | 101 | 6.00E-23 | 71% |
| SinsCYP5 | 201 | 147 | N | N | 0 | 0 | cytochrome P450 6a2a | AXP17141.1 | *Cydia pomonella* | 92.4 | 1.00E-20 | 57% |
| SinsCYP6 | 1423 | 1140 | N | N | 0.13 | 0.29 | cytochrome P450 CYP321B1 | AID54858.1 | *Helicoverpa armigera* | 468 | 1.00E-158 | 61% |
| SinsCYP7 | 216 | 147 | Y | N | 3.52 | 0 | cytochrome P450 6B5-like | XP_028177597.1 | *Ostrinia furnacalis* | 88.6 | 9.00E-19 | 62% |
| SinsCYP8 | 366 | 354 | N | N | 0.3 | 0.89 | Cytochrome P450 2U1 | KPI97401.1 | *Papilio xuthus* | 212 | 7.00E-67 | 81% |
| SinsCYP9 | 782 | 693 | N | N | 0.25 | 0.15 | cytochrome P450 9e2-like | XP_022830826.1 | *Spodoptera litura* | 381 | 1.00E-127 | 71% |
| SinsCYP10 | 551 | 465 | N | N | 0.21 | 0.06 | cytochrome P450 CYP315A1 | ALJ84053.1 | *Helicoverpa armigera* | 247 | 5.00E-77 | 65% |
| SinsCYP11 | 317 | 246 | N | N | 0.24 | 0.61 | PREDICTED: cytochrome P450 4d2-like | XP_011558063.1 | *Plutella xylostella* | 145 | 9.00E-39 | 57% |
| SinsCYP12 | 542 | 513 | N | N | 0.32 | 0.37 | cytochrome p450 CYP324A1 | ASO98021.1 | *Spodoptera exigua* | 209 | 5.00E-62 | 61% |
| SinsCYP13 | 872 | 423 | Y | N | 0.29 | 0.14 | Cytochrome P450 6k1 | KPJ18519.1 | *Papilio machaon* | 377 | 7.00E-95 | 65% |
| SinsCYP14 | 254 | 231 | N | N | 0 | 0 | cytochrome P450 CYP314A1 | QAX33065.1 | *Carposina sasakii* | 157 | 5.00E-46 | 88% |
| SinsCYP15 | 594 | 423 | Y | N | 0.09 | 0.16 | cytochrome P450 CYP314A1 | ABD18735.1 | *Manduca sexta* | 332 | 9.00E-82 | 77% |
| SinsCYP16 | 551 | 384 | Y | N | 0.26 | 0.06 | Cytochrome P450 CYP12A2 | KPJ08442.1 | *Papilio machaon* | 190 | 8.00E-55 | 60% |
| SinsCYP17 | 2100 | 1332 | Y | N | 15.02 | 17.95 | probable cytochrome P450 305a1 | XP_028159269.1 | *Ostrinia furnacalis* | 733 | 0 | 74% |
| SinsCYP18 | 1073 | 285 | Y | Y | 1.58 | 1.35 | cytochrome P450 monooxygenase | ARA91609.1 | *Pieris rapae* | 72 | 0 | 60% |
| SinsCYP19 | 1933 | 1554 | Y | N | 0.35 | 0.79 | PREDICTED: probable cytochrome P450 6a14 | XP_013171094.1 | *Papilio xuthus* | 781 | 0 | 72% |
| SinsCYP20 | 1888 | 1512 | Y | N | 1112.16 | 1227.22 | Cytochrome P450 6B46 | KOB75101.1 | *Operophtera brumata* | 725 | 0 | 68% |
| SinsCYP21 | 1042 | 1038 | N | N | 1.35 | 0.78 | cytochrome P450 333B11 | ADE05592.1 | *Manduca sexta* | 513 | 4.00E-178 | 67% |
| SinsCYP22 | 1755 | 1584 | Y | N | 5.43 | 4.15 | cytochrome P450 9G3 | NP_001108456.1 | *Bombyx mori* | 707 | 0 | 63% |
| SinsCYP23 | 3127 | 1533 | Y | N | 13.35 | 15.66 | cytochrome P450 9e2-like isoform X1 | XP_028157328.1 | *Ostrinia furnacalis* | 721 | 0 | 66% |
| SinsCYP24 | 3107 | 1479 | Y | N | 3.15 | 6.08 | cytochrome P450 6a2-like | XP_028161855.1 | *Ostrinia furnacalis* | 803 | 0 | 78% |
| SinsCYP25 | 2223 | 1620 | Y | N | 15.79 | 15.94 | cytochrome p450 CYP306A1 | ASO98030.1 | *Spodoptera exigua* | 931 | 0 | 82% |
| SinsCYP26 | 3446 | 1527 | Y | N | 13.45 | 11.39 | cytochrome P450 CYP12A2-like | XP_026491300.1 | *Vanessa tameamea* | 677 | 0 | 66% |
| SinsCYP27 | 2277 | 1575 | Y | N | 27.7 | 33.95 | probable cytochrome P450 9f2 | XP_026493625.1 | *Vanessa tameamea* | 673 | 0 | 64% |
| SinsCYP28 | 1888 | 1569 | Y | N | 36.99 | 71.42 | cytochrome P450 | ABB69054.1 | *Helicoverpa armigera* | 642 | 0 | 59% |
| SinsCYP29 | 2660 | 1623 | Y | N | 20.64 | 22.96 | cytochrome P450 CYP18A1 | AKP80583.1 | *Helicoverpa armigera* | 922 | 0 | 87% |
| SinsCYP30 | 1670 | 1557 | Y | N | 1.75 | 1.98 | cytochrome P450 CYP302A1 | AID54852.1 | *Helicoverpa armigera* | 764 | 0 | 71% |
| SinsCYP31 | 2236 | 1500 | Y | N | 1.92 | 2.29 | cytochrome P450 4C1-like | XP_021193950.1 | *Helicoverpa armigera* | 651 | 0 | 64% |
| SinsCYP32 | 1946 | 1536 | Y | N | 3.83 | 6.42 | probable cytochrome P450 304a1 | XP_021200659.1 | *Helicoverpa armigera* | 790 | 0 | 72% |
| SinsCYP33 | 3094 | 1482 | Y | N | 213.07 | 161.85 | cytochrome P450 4d2-like | XP_021191956.1 | *Helicoverpa armigera* | 640 | 0 | 65% |
| SinsCYP34 | 1860 | 1575 | Y | N | 32.24 | 31.95 | probable cytochrome P450 9f2 | XP_026493625.1 | *Vanessa tameamea* | 773 | 0 | 69% |
| SinsCYP35 | 1644 | 1158 | Y | N | 1.01 | 0.62 | cytochrome P450 4V2-like | XP_022823175.1 | *Spodoptera litura* | 758 | 0 | 80% |
| SinsCYP36 | 1842 | 1602 | Y | N | 3533.06 | 2035.71 | cytochrome P450 | ABB69054.1 | *Helicoverpa armigera* | 732 | 0 | 67% |
| SinsCYP37 | 1705 | 480 | Y | N | 1.28 | 1.51 | cytochrome P450 CYP12A2-like | XP_021188293.1 | *Helicoverpa armigera* | 486 | 2.00E-97 | 60% |
| SinsCYP38 | 2280 | 1635 | Y | N | 16.48 | 13.66 | cytochrome p450 CYP428A1 | ASO98058.1 | *Spodoptera exigua* | 810 | 0 | 75% |
| SinsCYP39 | 2428 | 1584 | Y | N | 32.33 | 25.58 | probable cytochrome P450 9f2 | XP_026493625.1 | *Vanessa tameamea* | 706 | 0 | 65% |
| SinsCYP40 | 3139 | 1536 | Y | N | 225.79 | 440.77 | cytochrome P450 4C1-like | XP_026750988.1 | *Galleria mellonella* | 750 | 0 | 69% |
| SinsCYP41 | 1611 | 1314 | Y | N | 0.75 | 2.59 | probable cytochrome P450 49a1 | XP_022823391.1 | *Spodoptera litura* | 773 | 0 | 84% |
| SinsCYP42 | 496 | 363 | N | N | 9.27 | 7.8 | P450 CYP6 family protein 13 | AIJ00773.1 | *Plutella xylostella* | 177 | 5.00E-50 | 76% |
| SinsCYP43 | 1576 | 1506 | N | Y | 1 | 0.88 | cytochrome P450 4C1-like | XP_028173337.1 | *Ostrinia furnacalis* | 653 | 0 | 69% |
| SinsCYP44 | 2132 | 1497 | Y | N | 1.41 | 1.06 | PREDICTED: cytochrome P450 4C1-like | XP_013185000.1 | *Amyelois transitella* | 622 | 0 | 59% |
| SinsCYP45 | 6026 | 1596 | Y | N | 13.13 | 14.39 | cytochrome P450 9a20 | NP_001077079.1 | *Bombyx mori* | 815 | 0 | 70% |
| SinsCYP46 | 426 | 333 | N | N | 0.1 | 0.22 | cytochrome P450 305a1 | AXP17177.1 | *Cydia pomonella* | 197 | 2.00E-60 | 74% |
| SinsCYP47 | 212 | 147 | Y | N | 3.95 | 0 | cytochrome P450 9e2-like isoform X1 | XP_028033856.1 | *Bombyx mandarina* | 102 | 1.00E-23 | 65% |
| **glutathione S-transferases (GSTs)** | | | | | | | | | | | | |
| SinsGST1 | 217 | 177 | N | N | 5.14 | 2.08 | glutathione synthetase-like isoform X1 | XP_028175876.1 | *Ostrinia furnacalis* | 155 | 3.00E-43 | 94% |
| SinsGST2 | 622 | 213 | Y | N | 0.45 | 0.1 | glutathione S-transferase siama 2 | AWX68889.1 | *Heortia vitessoides* | 239 | 3.00E-77 | 71% |
| SinsGST3 | 842 | 456 | Y | N | 697.78 | 734.32 | glutathione-S-transferase microsomal | AYM01169.1 | *Spodoptera littoralis* | 245 | 4.00E-79 | 77% |
| SinsGST4 | 1051 | 660 | Y | N | 15.94 | 15.88 | glutathione S-transferase 1-like | XP_026763041.1 | *Galleria mellonella* | 248 | 5.00E-78 | 57% |
| SinsGST5 | 1044 | 645 | Y | N | 18.22 | 19.04 | glutathione S-transferase zeta 1 | AKS40351.1 | *Chilo suppressalis* | 441 | 3.00E-154 | 98% |
| SinsGST6 | 1196 | 765 | Y | N | 102.51 | 112.97 | glutathione S-transferase omega 1 | NP_001040131.1 | *Bombyx mori* | 431 | 8.00E-149 | 83% |
| SinsGST7 | 1675 | 702 | Y | N | 35.87 | 40.09 | glutathione S-transferase 1-1 | XP_028161941.1 | *Ostrinia furnacalis* | 411 | 5.00E-139 | 82% |
| SinsGST8 | 1372 | 873 | Y | Y | 51.66 | 43.79 | glutathione S-transferase omega 2 | ABD36306.1 | *Bombyx mori* | 395 | 2.00E-133 | 65% |
| SinsGST9 | 1217 | 660 | Y | N | 11.45 | 17.88 | glutathione S-transferase 1-1-like | XP_026748199.1 | *Galleria mellonella* | 296 | 2.00E-96 | 78% |
| SinsGST10 | 1263 | 654 | Y | N | 267.17 | 179.12 | glutathione S-transferase 1-like isoform X1 | XP_026750985.1 | *Galleria mellonella* | 296 | 4.00E-96 | 64% |
| SinsGST11 | 1824 | 702 | Y | N | 3.71 | 5.73 | glutathione S-transferase epsilon 6 isoform X1 | XP_012548743.1 | *Bombyx mori* | 317 | 1.00E-101 | 62% |
| SinsGST12 | 1161 | 717 | Y | N | 12.22 | 12.18 | glutathione S-transferase omega-1 | ARM39008.1 | *Cydia pomonella* | 439 | 2.00E-152 | 88% |
| SinsGST13 | 2903 | 735 | Y | Y | 2348.61 | 1532.9 | glutathione S-transferase 1-like isoform X1 | XP_026485391.1 | *Vanessa tameamea* | 392 | 3.00E-126 | 73% |
| SinsGST14 | 1616 | 654 | Y | N | 5.99 | 6.74 | glutathione S-transferase 1-like | XP_021183202.1 | *Helicoverpa armigera* | 322 | 2.00E-104 | 68% |
| SinsGST15 | 3592 | 1197 | Y | N | 14.63 | 15.26 | glutathione S-transferase | AMY26654.1 | *Chilo suppressalis* | 382 | 6.00E-121 | 82% |
| SinsGST16 | 2465 | 615 | Y | N | 158.56 | 152.6 | glutathione S-transferase sigma 1 | AVC05621.1 | *Heortia vitessoides* | 310 | 5.00E-97 | 72% |
| SinsGST17 | 344 | 210 | N | N | 0 | 0.23 | glutathione S-transferase E14-like isoform X1 | XP_022837684.1 | *Spodoptera litura* | 131 | 3.00E-35 | 62% |
