# Supplementary material for: Antennal transcriptome analyses and olfactory protein identification in an important wood-boring moth pest, Streltzoviella insularis (Lepidoptera: Cossidae)
Source: Sci Rep. 2019 Nov 29;9:17951. doi: 10.1038/s41598-019-54455-w (PMC6884542; doi:10.1038/s41598-019-54455-w)
Supplement: Supplementary file 10 — Supplementary Table S10 [file 41598_2019_54455_MOESM10_ESM.docx]

**Supplementary Information for**

**Antennal transcriptome analyses and olfactory protein identification in an important wood-boring moth pest, *Streltzoviella insularis* (Lepidoptera: Cossidae)**

**Yuchao Yang^1^, Wenbo Li^1^, Jing Tao^1^*, Shixiang Zong^1^***

^1^Beijing Key Laboratory for Forest Pest Control, Beijing Forestry University, Beijing 100083, China

* Corresponding authors

**Email addresses:**

Yuchao Yang: yangyc68@126.com

Wenbo Li: leonardolee24@hotmail.com

Jing Tao: taojing1029@hotmail.com

Shixiang Zong: zongsx@126.com

**Table S10.** Amino acid sequences used to generate phylogenetic trees.

**OBP:**

>SinsOBP1

MAVKIFIAMFVAIFAATMQSTEAITEEKKTELKAKLVPILTECGKEHSITLDYLKEFRQQQKRPDESNACFFACVFQKTGLLDDKGLFVEAKAAERSQYYAEEGELEKANEAVKACVSVNEQSVSDGPKGCDRAKLIYNCFLDQKQHFGYVY

>SinsOBP2

MFKSVLVSFALFVLILGNAKAISDEYRTQIQSKIVEVGADCFKEHPLGLDDIAAFKIRKFPDGENAGCFTSCIFKKIGIFDETGILSRETAIENVKEIIEDEEELQNVRDFFAACSSVNDEDVTDGELGCDRAKLAFNCFVENSEKFGLNLDF

>SinsOBP3

MNIQITFVTILLMKFSVCVYCDPSLSDNHKRKLSHHFVTFGVGCLAKHNVSFEDIRALQNFNMPKGKDAPCFFACILKKANMIDSTGKVSPEAVAEVAKMLFTDMDEFNAVVSVFLDCSYVNDLEVSDGVKGCDRAKLVLSCIMEETKMESPSERPTILDGENVNSTSPVRTKRDLREIIGGVFRALLQLACTGVSKEYLHGRIITGLLPGLDNVDLLCRFIS

>SinsOBP4

MAMESSTLCMVLVALVAGAAAMDEEMAELARMLRENCGEETGVDLGLVDQVNGGADLMPDPKLKCYIKCVMETAGMFSEGQVEVEGVIAMLPEHMKKSEGFIRGCGTQKGADDCDTAFLTQVCWQKANKAEFFLV

>SinsOBP5

MISLNNSVFVYIIFFIILSRTFVMSMTRQQMKNSGKLLKKTCMPKNDVTEDQVGSIEQGKFIENKNVMCYIACIYSMGQVVKNNKIVFDAMIKQVDMMFPPDMKEPFKESIEKCKGVPKKYKDICEASYWTAKCLYDADPANFFFP

>SinsOBP6

MNQIVIKMTKSFILLLTIVLLWCSLAKSLNVRKDDVKMNKDEEINMSTIIPAMSMEEQNRINDVDMMAIMNECNETFHIEMSYLESLNASGSFLDETDKTPKCYIKCILEKSGVFDEEKGVFDPAKTASVFAGERGGRPMDDIEEMAATCTDRKEFCKCERSYNYMKCLMEMEIKRYEMH

>SinsOBP7

MKCHKEYPVDMSELLQLQKFIVPKKTETKCLLACAYKIDGMLNDKGMYDLEHAYKRAELTKNGDEKRLVNAKKLADICVKVNDVEVSDGEKGCERSALMFKCLVHNAPKLGFKL

>SinsOBP8

MQFTFTNFVTLITFLTSTSKICLVVSFEPLSKGEHLDKIVKMNEEVEPFRRNLTECARQVKASMVDVENFLKRIPQGTMQGKCFVACILKRNSIIKNNKISKENLIEANRAVYSDDSEVMTRLKIAVEECTDVVEGIFEICEYASVFNDCMHMKMEHILDKVTMERRMEALGQ

>SinsOBP9

MRTLLVFAVCFVVAQALTDEQKDKLKKHRSECLSETKVDQQLVDKLKSGDFKTENEPLKKYSLCMLMKSELMTKDGKFKKDVALAKVPNEADKPTVEKLIDTCLANKGNNPHQTAWNYVKCYHEKDPKHAIFL

>SinsOBP10

MVTVVAFLALLPSLVTSATEGNIHLLEDEIALALRACSYPDTNTNIVKGNEDQRQTRYVRSSNRFYDEQSPKIDSGVKEDMNQYGHEKRNATDMRIPKQIHVLNTTEYNYGCNDTERGGKRQTSSVTRPEFVNESANNTRVKRTEPLLDDSDQCLSQCVFANLEVIDSRGIPLEAELWNRVQSSLASQQSRVALRDQIRACFQELETDGNDNGCSFSNKLERCLMLNFSERKMNGTI

>SinsOBP11

MPPLVTSLALLVIIVSVHQATLGCKNCIILGKEEKAMFRVHSDACQAQSQVDSKLLESLLNGELIDDPGLRKHVYCVLLKCKMIGKDGKLQKAAILGKMAPRVDGRNATKVLESCSEQKGDSPEDVAWNLFRCGYDKKALLFDYMPSGGGGDIDNNSN

>SinsOBP12

MCHIKYYIFVVLILVSDSFALNCRSDGGPKEEELKAIYMNCLKRQEGKNSSDNRGYSGELDWNESRGQNQRSHCDKRDDSRSRDERTGSRDDRMESRDDRMGRRNDRTRNRDDRSGGRDDRISNRDDGMTARDDRFGNHDNRFGREDHLNGKDDFGLMEDDFGSDIRRYNNYGATNQPSKRFKRERRLEMNSGHRSQYNPNFSPKSSSYDDTYGNNENNSSESSSNSDSKACALHCFLEELKMMGDNGMPDRYLVTHAITKDVKNEDLRDFLQESIEECFQILYNENTDDKCAFSKNLMMCLSEKGRANCDDWKDDIRF

>SinsOBP13

MNMQKVHLFCFFIVLIDFLLFNIGFAVTRQQFKNSGKLLKKSCMPKNDVTEEQVGEIEQGKFIEDRNVMCYIACIYTMSQIVKNNKINYEAVMKQVDMLFPPELKEPAKAAAANCKDVSKKHKDLCEAAYWTAKCMYDFDPKSFIFP

>SinsOBP14

MKMASFYCVFLCYGIVALYFIDVNAVSQEEIIKIDGALLPFITECSVQNGVNIEDLTAAKKNDNYDNLNPCLIACVFKKTGMMDDKGLFNLDKALEKTKKFLKSEEDIDKAAKVAKSCASVNDQEISDDDKSCKRAKLLLDCFIKHKGQFPISI

>SinsOBP15

MCSFFFTIMERKEICLLIIIIVFSCTESMTRQQLKNSSKMLKKQCMGKNQVTEDLIGDIEKGKFIEDRNVMCYIACIYQMLQVVKNNKLNYEASLKQVDMMYPADIKEAAKATITKCKDVSKKYKDLCESSYWTAKCIYENDPKNFIFA

>SinsOBP16

MYYLIVFSFILSLAYTKSAQQVIRLAPDVNSRLQPIITQCISETGSDPSIVLELNSMKISNEEQFKKFIYCDFTKSDLTTAEGHWKIEKVLQFYPKTVEIAPLQKAMESCNKENGQDPADTTFKIFKCFIKRTPVIISF

>SinsOBP17

MSNIFLFLLCSYLISATYARSDEEIKQWFISVGMECNKDYPITPDDMQTMMQKYQIPNSKNAKCLMACVYRKANWLDSEGMFDVDSANSMMEKEHADDPAKMEKAKKLFKICKKVNDEPVTDGNEGCDRSAHMFQCLVENALKMGFKIQ

>SinsOBP18

MILILLVKLLILTTFCYAMTMKQIKSTGKMMRKSCQPRNNVEDEKIDPIQNGVFIEEKEVMCYMACIMKMANTIKNGKLNYDAAIKQADLLLPEEIKEPAKEAITACKKVADSHKDICEASFHVTKCIYKYNPDIFYFP

>SinsOBP19

MYNNFCYVCFFLVIFIIKDALAITLIQKTRIQAKFLASGIECINDYPLTANDIQILKSKVMPENENANCFVNCLFKKIGIMDDMGKMTQAGAREFAKQVFKDDDENVKKTDELYGQCSSVNEKSVSDGDKGCDRARLAFICLAENAPKFGLDVDF

>SinsOBP20

MGQDFIEICQIFFLDQVGHINEGIFVENHNVMCYIACIYKLTQAVKNNKLNLDLLIKQIDILYPTDMKEEVKKSVYACIHVQDNYDDMCEAIFYTTKCFYEFDPTYFIFA

>SinsOBP21

MVPKISALLCYICVFSISLSDTAISAEDEKRCKNPPTAPQKIERVITLCQDEIKLSILREALDVIKEEHTMPAQRIRNKREVPFTHDEKRIAGCLLQCVYRKVKAVDGFGFPTLEGLVGLYSDGVNERGYFMAVLEASRECLMRNHDKFSRTTPMDNGRNCDVSFDIFECISDRIGEYCGTSGL

>SinsOBP22

MSQAIVLLCGTFLLASIPFTSAVTDEQKAEIQAYFEKIGAECITDHQISEADITELRARKISSGENVPCFLACMMKKMGIMEDGGNLRKETALELIKEVIKDADELKKIEEYLHSCSHVNTEPIGSGEVSCERAMLAYKCMIDNSSQFGILV

>SinsOBP23

MSYTTLPVFLLLITFGYSAKEKPEFSEEIKEIIQHVHNECVGKTGVAEEDITNCENGIFKEDVKLKCYMFCLLEEASLVDDDGIVDYDMMVSLIPEQYYDRVTKMIYSCKHLDTEDKDKCQRAFDVHKCSYEKDPSFYFLF

>SinsPBP1

MDSEVADIIYKEALQKYTAKMLTQTKIVVLVIVYLAIDSRVESSKEIMKEMTVNFGKALADCKKEMGLPDSIDADFYNFWKEDYEVSNRYTGCAIICLSTKLDLVDPDGGLHHGNAHEFAKKHGADDAMAKQLIDIIHQCEKSTPRNDDGCIMMLGIAKCFKAEIHKLDWAPSMDLMVGEVIAEV

>SinsPBP2

MKDIKTAGQIQLFSAVDKMAAVTKWRAFVICLAVLAFDLHKVNSSQDVMKNLSSTFGKVLEECKNELNVGDHIMKDFYNYWREDYELLNKDMGCVIMCIASKLDLLTDEMKMHHGKAHEFVKSHGADDTMAKQLVTITHECENTHAGIGDDCSRVLEISKCFRSKIHDLNWAPPMEVIIEEIMTEI

>SinsPBP3

MAGQLQFFVALVLFAIGVTEIDSSAETMKNISSGFIKVLDECKQELNLGEHILNDFYHFWKEEYSLLNRETGCAIICMSKKLDLLDPDGNLHHGNAKEFAMKHGAEDEVASKLITLIHECEKQHTAVEDECLRKLEVAKCFRSGIHQLNWAPNMDVIVTEVLTEM

>SinsGOBP1

MHITRQGAGGTIQSLDVCQVERMERKMLKLIGILLCSLLAGSLATVEVMKDVTLGFGEALKYCREESQLSEEQMEEFFHFWRDDFKFNLREIGCAIRCMSHHFNLLTDSHRMHHDNTDKFIKSFPNGELLSQKMVTLIHECEKQFESEEDHCWRILRIAECFKTGCKQQGIAPTMEMIMAEFIMESESR

>SinsGOBP2

MAAVYCLLFSLVIFVSITDEVMGTAEVMSHVTAHFGKSLDECRKESGLSSEIMEEFKHFWSEDFEVVHRELGCAIICMSNKFSLLKDDTRMHHMNMDDYIKSFPKGDILSAKMVELIHNCEKQYDDIKDDCSRVVKVAACFKVDAKKEGIAPEVTMIEAVMEQYNN

>BmorGOBP1

MWKLVVVLTVNLLQGALTDVYVMKDVTLGFGQALEQCREESQLTEEKMEEFFHFWNDDFKFEHRELGCAIQCMSRHFNLLTDSSRMHHENTDKFIKSFPNGEILSQKMIDMIHTCEKTFDSEPDHCWRILRVAECFKDACNKSGLAPSMELILAEFIMESEADK

>BmorGOBP2

MFSFLILVFVASVADSVIGTAEVMSHVTAHFGKTLEECREESGLSVDILDEFKHFWSDDFDVVHRELGCAIICMSNKFSLMDDDVRMHHVNMDEYIKGFPNGQVLAEKMVKLIHNCEKQFDTETDDCTRVVKVAACFKKDSRKEGIAPEVAMIEAVIEKY

>BmorOBP1

MSRQQLKNSGKMLKKQCMGKNDVTEEEIGDIEKGKFIEQKNVMCYIACIYQMTQIIKNNKISYEASIKQIDLMYPPELKESAKASAGRCKDVSKKYKDICEASYWTAKCMYEDNPKDFIFA

>BmorOBP4

MTSAKTDVEIKAWFLGQAVECSKDHPVTTEELRMHKHELPDSKNAKCLMKCVFRKCNWLDSKGMYDINAAYASSTKDFSDDKTKQENANKLFDTCKSVNEENVGDGEEGCDRSLLLAKCLTKAAPQFGFQL

>BmorOBP6

MSIKWRHIERVGSFCYLGSIVDDRGGTEADIAARINKARAAFSQLRPVWSSSTLTRRTKALTEEQKAEITKSSLPLIAECSKEFSVNQGDIDAAKKLGDPSGLNSCFVGCFMKKAGIINASGLFDVAATIEKSKKYLTSEEDLKAFEKLTETCAPENDKPVSDSDKGCERAKLLLDCFVANKGSNTRIVALSKDLDEEAEDRAQWLAIGEAYVQQ

>BmorPBP1

MSIQGQIALALMVYMAVGSVDASQEVMKNLSLNFGKALDECKKEVGKMTLTDAINEDFYNFWKEGYEIKNRETGCAIMCLSTKLNMLDPEGNLHHGNAMEFAKKHGADETMAQQLIDIVHGCEKSTPANDDLCIWTLGVATCFKAEIHKLNWAPSMDVAVGEILAEV

>BmorPBP2

VEMVCGSRDVMTNLSIQFAKPLEACKKEMGLTETVLKDFYNFWIEDYEFTDRNTGCAILCMSKKLELMDGDYNLHHGKAHEFARKHGADETMAKQLVDLIHGCSQSVATMPDECERTLKVAKCFIAEIHKLKWAPDVELLMAEVLNEVSWKS

>BmorPBP3

MARYNIVVAVLVLGVVGARGSSEAMRHIATGFIRVLDECKQELGLTDHILTDMYHFWKLDYSMMTRETGCAIICMSKKLDLIDGDGKLHHGNAQAYALKHGAATEVAAKLVEVIHGCEKLHESIDDQCSRVLEVAKCFRTGVHELHWAPKLDVIVGEVMTEI

>CpomGOBP1

MSHIARLVLSLAAVALAHATVEVMKDVTLGFGEALEHCRESSGLTEEKMEEFFHFWHDDFKFEHRELGCAIQCMSRHFNLLTDGQRMHHENTDKFIKSFPNGEVLSKTMVTLIHSCEQKFDDMEDHCWRILRIAECFKSGCQERGLAPSMEMMMAEFIMESEV

>CpomGOBP2

MALYWLVGLVLVGGKMVDGTAEVMSHVTAHFGKALEQCREESQLSPEVLDEFHNFWREDFEVVHRELGCAIMCMSNKFSLLQDDARMHHENMHDYVKSFPQGDVLSAKMVELIHNCEKQYDDIPDDCSRVVKVAACFKVDAKKAGIAPEVAMIEAVLEKY

>EhipOBP24

MKFLFLCFIIAAGSLDAHNVHLSQSQKDKVHQYTLQCITESGVKPEVIAEAKKGHFNDDEALKKFILCFFQKSSILNGEGKLDVEAALSKLPSDVDKTAVKKVLEDCKNKTGKSTADTAFEIFKCYYKGTPTHVIFS

>EhipOBP23

MSYTTLLIFLLLITFGYSAKEKPEFSEEIKEIIQHVHNECVGKTGVAEEDITNCENGIFKEDVKLKCYMFCLLEEASLVDDDGIVDYDMMVSLIPEQYYDRVTKMIYSCKHLDTEDKDKCQRAFDVHKCSYEKDPSFYFLF

>EhipOBP20

MSIFFLDQVGHIDQGIFVENHNVMCYIACIYKLTQAVKNNKLNLDLLIKQIDILYPTDLKEEVKKSVYACIHVQDNYDDMCEAIFYTTKCFYEFDPKYFIFA

>EhipOBP19

MYNNNFCYVCFFLFIFIIKDALAITLIQKTRIQAKFLASGIACINDYPLTANDIQILKSKVMPDNENANCFVACLFKKIGIMDDMGKMTQAGAREFAKQVFKDDDEPLKKTDELIGQCSSVNANSVSDGDKGCDRARLLFVCLAENAPKVYTITHTKYIKLYGPTTPSNTYNF

>EhipOBP18

MILILLVKLLILTTFCNAMTMKQIKSTGKMMRKSCQPRNNVEDEKIDPIQNGVFIEEKEVMCYMACIMKMANTIKNGKLNYDAAIKQADLLLPEEIKEPAKEAITACKKVADSHKDICEASFHVTKCIYKYNPDIFYFP

>EhipOBP17

MSLGMECNKDYPITPDEVQTMMQKYQIPNSKNAKCLMACVYRKVNWLDSKGTFDVDSANSMMEKEHADDPAKMEKAKKLFEICKKVNDEPVTDGNEGCDRSAHMFQCLVENALKMGFKIQ

>EhipOBP16

MYYLIVFSFILSLAYTKSAQQVIRLEPDVNSRLLPIITQCVSETGSDPSIVLELSSMKISNEEQFKKFVYCGFTKSDLTTAEGHWKIEKALQFYPKTVEIAPLQKAMESCNKESGQDPADTTFKIFKCFIKRTPVLISF

>EhipOBP15

MTRQQLKNSSKMLKKQCMGKNQVTEDLIGDIEKGKFIEDRNVMCYIACIYQMLQVVKNNKLSYEASLKQVDMMYPADIKEAAKATITKCQDVSKKYKDLCESSYWTAKCIYEDDPKNFIFP

>EhipOBP14

MASFYCVFLCYGIVALYFINVNAVSQEEIIKIEGALLPFITECSAQNGVNMEDLTAAKKNENYDNLNPCLIACVFKKTGTMDDKGLFNLDKALEKTKKFLKSEEDIDKAAEVAKSCASVNDQEISDNDKSCGRAKLLLDCFIKHKGQFPLSI

>EhipOBP13

MNMQKVHLFWFFIVLIDFLLFNIGFAMTRQQFKNSGKLLKKSCMPKNDVTEEQVGEIEQGKFIEDRNVMCYIACIYTMSQVVKNNKINYEAVMKQVDMLFPSELKEPAKAAAANCKDVSKKHKDLCEAAYWTAKCMYDFDPKSFIFP

>EhipOBP10

MVTVVAFLALLPSLVTSATEGNIHLLEDEIALALRACSYPDTNTNIVKENEDQRQSRYVRSSNRFYEEQSPRIDSGVKEDTNQYGHERRNATDMRIPKQIRVLNTTEYNYGCNDTERGGERQTSSVTRPEFVNESANNTRVKRTEPLLSKDDSDQCLSQCVFANLEVIDSRGIPLEAELWNRVQSSLASQQSRVALRDQIRACFQELETDGNDNGCSFSNKLERCLMLNFSERKINGTI

>EhipOBP9

MRTLLVFAVCFVVAQALTDEQKDKLKKHRSECLSETKVDQQLVDKLKSGDFKTENEPLKKYSLCMLMKSELMTKDGKFKKDVALAKVPNEADKPTVEKLIDTCLANKGNNPHQTAWNYVKCYHEKDPKHAIFL

>EhipOBP7

MKCHKEHPVDMSELLQLQKLIVPKKTETKCLLACAYKIDGMLNDKGMYDIEHAYKRAELSKNGDEKRLVNAKKLADICVKVNDVEVSDGEKGCERSALMFKCLIHNAPKLGFKL

>EhipOBP6

MKCIFRKAGWLDGDKVDKEKVTAHFDQFAKDNPSWSPAVQYVKAACLATDLPAQGVYINCPAYDVVHCSLTGFFKNAQASQWSTSQECAYPRQFAQACPVCPGDCFAPAVPYGSCNACRLLPQTP

>EhipOBP5

MISLNNSLFVYIIFFLILSRTFVMSMTRQQIKNSGKLLKKSCMPKNDVTEDQVGNIEQGKFIENKNVMCYIACIYSMGQVVKNNKIVFDAMIKQVDMMFPPEMKEPFKESIEKCKGVPKKYKDICEASYWTAKCLYDADPANFIFP

>EhipOBP4

MAMESSTLCMVLVALVAGAAAMDEEMAELARMLRENCGEETGVDLGLVDQVNGGADLMPDPKLKCYIKCVMETAGMFSEGQVEVEAVIAMLPENMKKSEGSIRGCGTQKGADDCDTAFLTQVCWQKANKAEYFLV

>EhipOBP1

MAVKIFIAMFVAIFAATMQSTEAITEEKKTELKAKLVPILTECGKEHSITLDYLKEFRQQQKRPDESNACFFACVFQKTGLLDDKGLFVEAKAAERGQYYAEEGELEKANEAVKACVSVNEQSVSDGPKGCDRAKLIYNCFLDQKQHFGYVY

>EhipGOBP2

MAAIYCLLFSLVIFVSITDEVMGTAEVMSHVTAHFGKSLDECREESGLSSEIMEEFKHFWSEDFEVVHRELGCAIICMSNKFSLLKDDTRMHHMNMDDYIKSFPKGDILSAKMVELIHNCEKQYDDIKDDCSRVVKVAACFKVDAKKEGIAPEVTMIEAVMEQYNN

>EhipGOBP1

MASHSLWSISYAYNAPGSGGTIQSLDVYQVETMERKMLKLIGILLCSLLAGSLATVEVMKDVTLGFGEALKHCREESQLSEEQMEEFFHFWRDDFKFDLREIGCAIRCMSHHFNLLTDSHRMHHENTDKFIKSFPNGELLSQKMVTLIHECEKQFESEEDHCWRILRIAECFKTGCKQQGIAPTMEMIMAEFIMESERR

>EhipPBP3

MAAVTKWRAFVICLTVLAFDLHKVNSSQDVMKNLSSGFGKVLEKCKNELNVGDHIMKDFYNYWREDYELVNKDMGCVIMCMATKLDLITDEMKMHHGKAHEFAKSHGADDTMAKQLVAIIHECENTHADIGDDCSRVLEISKCFRSKIHDLKWAPPMEVIIEEIMTEI

>EhipPBP2

MLTQTKIVVLVIVYLAIDSRVESSKEIMKEITVNFGKALADCKREMELPDSIDVDFYNFWKEDYEVSNRYTGCAIICLSTKLDLVDPDGGLHHGNAHEFAKKHGADDGMAKQLIDIIHQCEKSTPRNDDGCIMMLGIAKCFKAEIHKLDWAPSMDLMVGEVLAEV

>EhipPBP1

MAGQLQLFVALVLFAICVSEIDSSAETMKNISSGFIKVLDECKQELNLGEHILNDFYHFWKEDYSLLNRETGCAIICMSKKLDLLDPDGNLHHGNAKEFAMKHGAEEEVASKMITLVHECEKQHTGVEDECLRKLEVAKCFRSGIHQLNWAPNMDVIVTEVLTEM

>HarmGOBP1

LLADINVMKDVTLGFGQALDKCREESQLTEEKMEEFFHFWRDDFKFEHRELGCAIQCMSRHFNLLTDSSRMLHDNAEKFIQSFPNGEVLARQMVELIHSCEKQFDHEDDHCWRILHVAECFKGSCVQRGIAPSMELMMTELIMEAESR

>HarmPBP2

MMGSAMSSKELLTKMTGGFTKVVDACKTELSVGDHIMQDMYNFWREEYQLVNRDLGCMIMCMTAKLDLIGDDQRMHHGKAEEFAKSHGADDALAKQLVGLIHGCETQHQAIEDHCSRALEIAKCFRTKIHELKWAPSMEVIMEEIMTAA

>HarmPBP3

MGSRHVFFALVVLAVSVRKAEPSKDAMQYITSGFVKVLEECKHELDLNEQILADLFHFWKLEYSLLGRDTGCAIICMSKKLDLLDANGRMHHGNAAEFAKKHGAGDEVASKIVTIIHECEKKHEQDGDECLRVLEVAKCFRTGIHELDWQPKVEVIVSEVLTEI

>HarmGOBP2

MTSKCCLLLVAMATLTTSVMGTAEVMSHVTAHFGKALEECREESGLSAEVLEEFQHFWREDFEVVHRELGCAIICMSNKFSLLQDDSRMHHVNMHDYVKSFPNGHVLSEKLVELIHNCEKKYDTMTDDCDRVVKVAACFKVDAKAAGIAPEVAMIEAVMEKY

>HarmPBP1

MEFHRSTMMSVRLALVVAAWLFIRVDASQDVIKNLSMNFAKPLEDCKKEMDLPDSVTTDFYNFWKEGYEFTNRQTGCAILCLSSKLELLDQELKLHHGKAQEFAKKHGADDAMAKQLVDLIHGCAQSTPDVADDPCMKTLNVAKCFKAKIHELNWAPSMELVVGEVLAEV

>SlitGOBP1

MTSKCCLLLVLMAAATSSVMGTAEVMSHVTAHFGKALEECREESGLSAEVLEEFQHFWREDFEVVHRELGCAIICMSNKFSLLQDDSRMHHVNMHDYVKSFPNGHVLSEKLVGLIHNCEKQFDSMTDDCERVVKVAACFKVDAKAAGIAPEVAMIEAVMEKY

>SlitGOBP2

MLLLLRALPLLAAVLPLRADVNVMKDVTLGFGQALDKCRQESQLTEEKMEEFFHFWREDFKFEHRELGCAIQCMSRHFNLLTDTSRMHHENTEQFIQSFPNGEVLARQMVELIHACEKQHDHEEDHCWRILHVAECFKQACVQRGIAPSMEIMITEFIMEAEAR

>SlitPBP3

MGSRNVFVALVVLTVGMREIEPSKDPMKYIASGFVKVLEECKHELNMNDHLIADLFHYWKLEYTLLNRDTGCAIICMGKKLDLLDASGRMHHGNAQEFAKKHGAGDEVASQIVQIIHDCEKKHERDDDECLRVLEVAKCFRTGIHELNWQPNVEVIVSEVLTEI

>SlitPBP2

MAFCPSVTMSLRVALVVAASLLVVVQASQDVMKNLAVNFAKPLDDCKKEMDLPDSVTTDFYNFWKEGYELTNRQTGCAILCLSSKLEILDQELNLHHGRAQEFAMKHGADEAMAKQIVDMIHTCAQSTPDEAADPCMKALNVAKCFKLKVHELNWAPSVELIVGEVLAEV

>SlitPBP1

MANARWRFVFVVYALYLTSAVLGSQDLMVKMTKGFTRVVDDCKTELNVGDHIMQDMYNYWREDYQLINRDMGCMLLCMAKKLDLMDDQTMHHGKTEDFAKSHGADDDVAKKLVSVIHECEQQHAGIADDCMRVLEVAKCFRTKIHELKWAPSIEVIMEEVMTAV

>EoblPBP2

MTKLKELLLVLVISVITRVQSSQDVMKSLTLNFGKPMEVCKKELDLPDAVTKEFLNFWREGYEVKNRLTGCAIICMSEKLELLDEGLKLHHGNAKEFAKKHGADDGMAQQLVDMIHSCMESTPPNTDPCLKTVDVAMCFKLKIHDLSWNPDPDLIIAEVLAEA

>EoblPBP1

FALVLEDCKKQENVGDHIMQDIFNFWHEEYALVNPELGCVMLCMAGKLDLMDGDDMHHGNAHEFAKKHGADDDLAKQLVTMIHDCEKASASIADRCARALETTKCFRGKIHGLKWAPSMRVIMEEVMADMNV

>EoblGOBP2

MKSVLVATVVLSVVGLAMGTAEVMSHVTAHFGKALSECREESGLTPEVLEEFQHFWREDFEVVHRELGCAIICMSNKFSLLQEDSRIHHVNMHDYVKGFPNGQVLSAKMVELIHNCEQQYDDITDDCARVVKVAACFKRDAKKEGIAPEVTMIEAVMEKY

>EoblGOBP1

MARLASSVLAVVAVAAAAVVADVQVMKDVTLGFGQALEVCREESQLSQDVMEEFFHFWREDFKFESRAVGCALQCMSRHFNLLTDSSRMHHENTHRFIESFPNGSVLAKQMVSLIHGCEQQHEAEPDHCWRILRVAECFKRRCQEAGIAPSMEIIMAEFIMETEAK

>SexiPBP2

MAFCRSGTMSVLVVAASMLVVVQASQDVMKNLAINFAKPLDDCKKEMDLPDSVTTDFYNFWKEGYELTNRQTGCAILCLSSKLEILDQELNLHHGRAQEFAMKHGADETMAKQIVDMIHTCAQSTPDVAADPCMKTLNVAKCFKLKIHELNWAPSMELIVGEVLAEV

>SexiPBP1

MAGAKWQFVCVVFALYLTSAALGSQELMMKMTKGFTKVVDDCKAELNAGEHIMQDMYNYWREDYQLINRDLGCMILCMAKKLDLMEDQKMHHGKTEEFAKSHGADDEVAKKLVSIIHECEQQHAGIADDCMRVLEISKCFRTKIHELKWAPNMEVIMEEVMTAV

>SexiGOBP1

MLLLLRALPLLAAVLPLRADVNVMKDVTLGFGQALDKCRQESQLTEEKMEEFFHFWREDFKFEHRELGCAIQCMSRHFNLLTDTSRMHHENTEQFIQSFPNGEVLARQMVELIHACEKQHDHEEDHCWRILHVAECFKQACVQRGIAPSMEIMITEFIMEAEAR

>SexiGOBP2

MTAAGIRLSMAEVMSHVTAHFGKALEECREESGLSAEVLEEFQHFWREDFEVVHRELGCAIICMSNKFSLLQDDTRMHHVNMHDYVKGFPNGHVLSEKLVELIHNCEKRFDSMTDDCERVVKVAACFKVDAKAAGIAPEVAMIEAVMEKY

>OfurPBP1

MGLSLRLLVVVAAAIFGAESSQDVMKQMTINFGKALDTCRKELDLPDSINADFYNFWKEGYELSNRHTGCAIMCLSSKLDLVDPEGKLHHGNTHEFAKKHGADDSMAKQLVELIHKCEGSVADDPDACMKVLNIAKCFKAEIHKLNWAPSMDLIVAEVLAEV

>OfurPBP2

MWLSKTLVVIAVMCSMSVVVHSSQAVMKDMTKNFIKAYEVCAKEYNLPEAAGAEVMNFWKEGYVLTSREAGCAILCLSSKLNLLDPEGTLHRGNTVEFAKQHGSDDAMAHQLVDIVHACEKSVPPNEDNCLMALGISMCFKTEIHKLNWAPDHELLLEEMMAEMKQ

>OfurPBP3

MWLPKTLVVMSVMSSMSVVVHSSQTVMGEMTKNFIKAYEVCAKELNLSEATGLQLINFWKEGHELTTRETGCAILCMSTELNLLDVQGSVHRGNTVEFAKHHGSDDAMAHQVVDILHACEKATPNEDKCMLALSIAMCFKAEIHKLDWAPNNELMFEELVLDMWNS

>CsasOBP2

MVRKISALLCCLCVFGISSSDSAISAENEKRCRNPPTAPQKIERVITLCQDEIKLSILREALDVIKEEHTMPTQRRRNKREVPFTHDEKRIAGCLLQCVYRKVKAVDGYGFPTLEGLVGLYSDGVNERGYFMAVLEASRECLMRNHDLFSRTVPMDNGRNCDVSFDIFECISDRIGEYCGNTGL

>CsasOBP3

MEQRMLSILVITLLCNGIHAMTRAQLKNSAKMFKKSCLGKVDVNEDLIVDIEKGQFVEDRSVMCYIACIYQMSQIVKNNKLNYEASLKQVDIMYPAEMKDAVKATITACKDVSKKYKDLCEASFYTAKCIYTADPKNFVFA

>CsasOBP4

MEMQALKLPKSTNGKCLLACAYKKDGIMTEDGTYNLEHGYKMAELCKNGEEKRLVNGKKLADICSKVNDAKVSDGKKGCERAALIFTCVISNAPKLGFKV

>CsasOBP5

MTPYIFLCIVMAAAGLEAQHLTKEQKDKTLQYTAKCIKQTGVKPELVVEAKKGNFENDEALKKFTLCFFQKAGIIGSDGKLNVDAALAKLPQGVDKKAVGKVLEDCRKKTGKDAADTAFEVYKCYYKATPVHVV

>CsasOBP6

MRLPILTVLSLVAFITFSIGANSPAKSVVTTITTRMPETKEADSQDDSFNLMMLMAECNETFRTETSFIESLNETGSFPDETDRTPKCFLRCILERLEVASLDGVYDASKAAQVATGMGNTTPAADIEEIAGKCADRKEECKCEKAYQFMKCIIETQIKTAQKS

>CsasOBP7

MSSVNRIVAIFSMLIVLGNAGRDKPVFSDDVKEVIEHVHNKCVGKTGVAEEDITNCEKGIFKNDQRLKCYMFCLFDEASLVDKNGCVDFDLMVKMMPDVYSARFESGVNICKKEETMDKEACQRAFDMHTCMYKSDPEFYFLL

>CsasOBP8

MKTFIVFAICLVAAQALTDEQKEKLKKHRSECLLDTKADEQLVNKLKTGDFKTDNEQLKKYTHCLLVKSELMTKDGKFKKDVALAKVPNPADKPAVEKLIDACLANKGNTPQQTAWNYVKCYHEKDPKHSVVA

>CsasOBP9

MGCKNCVILGKEEKAMFRAHSDACLAESGVEPRLVDAMLSGELVDKPALRKHVYCVMLKCKIVAKDGKLQKTALLGKLNNRPDAKNATKVLENCAQQTGDTPVDIAWNLFRCGYDKKALLFNYMPTAPRDDHLENNSN

>CsasOBP10

MKKVLLVFCCVLFISDFSLGMTRQQMKNSLKLMKKTCMPKNDVTEEQVGSIEQGKFLEERNVMCYIACVYSMTQVVKNNKLNYEAVIKQIDMMFPPEMKDPVKASVDSCKDVGKKYKDICEASYYTAKCMYDFDPKIFVFA

>CsasOBP11_

MERLHFVLVTIAMLSVAIAEFPTAEFLEALKPTVEKCEAETGVNKDLVDQFSKGTMVDDPQLKCYMKCIFVEHDLLNEETGVIKYEKMLSLLPQEMKAIAYDMGKNCVHFKGEGGSDLCQVSYDLHKCWQKADPEHYFLM

>CsasOBP12

MLMLKILVCVLPYVVEGNRPLASSDTLVDFADPKVQGHLDAMVRMAQSCVIKVRATPKDVRAYFTNSPPVSRSGQCFAACMLEQSEVISHGKVNRDLLIHLASLVNGKESRVVKKLSSISRMCLDSIEGMSDKCQLASTYNDCLNENMIEFAFPLDIAEEAVRKMPFHLISPNLPPEGRQ

>CsasOBP13

MYKIYCFLIFCATVISADLISSSKGASLKPISACCDIPELADPKPLAECSVPKLPGPCSDVQCIFQHSGFLIDDRTLNKDAYREHLYKWSQNNIAWTAAVKRAISDCVDTDLRQYLDYPCRAYDVFTCTGIAMLKKCPKEFWKC

>CsasOBP14

MPSATLPVLFVLLVFGQAAKEKPVFSAEIEEIIQHVHNQCVDKTGVAEADIANCESGIFKDDNKLKCYMFCLLEEASLVDDEGVVDYDMMVSLIPEQYYDRVTKMIFGCKHLDTPDKDKCQRAFDVHKCSYDKDPDFYFLF

>CsasOBP15

MATRLALVMCTLAALARAKTEMDPEMAELAAMLRENCGDETGVDLALLDQVNAGAKLMDDAKLKCYMKCVMETAGMFGEGAVDVEAVVAMLPEDFRSRNEAMLRACGSRPGADDCDVAWQTQRCWQDGNPEDYFII

>CsasOBP18

MNLVTVVKFLTVLGLCGAMTMKQIRNTGKMMRKTCQPKNNVADEKIDPINEGVFIEEKEVMCYMACIMKMANAIKNNKLHLDSALKQADLLLPEEIKEPAKQAIIACRKYAESSKDICEASFQITKCIYNENPEIFYFP

>CsasOBP20

MYLENYLGICAGVLVLSFVNVHSLSEEQKNSIELKLLPILTECSQRNGITILDIAVARQTKNIDNIPPCVFACVFKKIGIINNEGLFDIEKAQRKAKKLLQNDEETSSFLTVAETCSSVNKEPRSNDRNGCLLSRELFRCFYRNKEVVGL

>CsasOBP24

MALITFLTVSLAVVSSVYSITPEEKMKFKEALKPALDECGAKYGVTLEDIKKAHESGNDDAIDPCMAGCVYKKIGVINDDGFYVADKSQEDIKNMLSNQDDIDKLNLVVAECSKINEESVTDGKEGCQRAKLLNNCFKNHKELFLRSI

>CsasGOBP1

MAMEKQKQVSLVLGLVLSLLVTNIAGNADIMKDIALGFGEALKHCRDESELTPEKMQAFFHFWDDDFKFEQRELGCAIECMSRHFNLLTEEGKMHHDNADKFIRSFPKGEQIAQQLLDIVHACETKNEAEEDHCWRVLHTAECFIHSAKEQNIAPSVDMLMAEFVVAES

>CsasGOBP2

MVSWLPWLLLVVAASVKGDAVQSESMSHVTAHFGKALAKCREESGLTPEILDEFQNFWREDFEVVHRELGCALICMSNNFELYNDDVRIHHDNMHDYIKSFPKGEELSPKMVELLHNCEKQFDDIVDDCDRVVKMAACFKRDAKAAGIAPEVAMIEAVMEKY

>CsasPBP1

MEVKRCLLVGLITVSCCVFVVDSSQETLKKITSSFMKVLEVCKEELGISENLLAGLYHFWKEEYELVSKEVGCAILCMSHKLNLLDESGKLHHGNAQEFAEQHGAADSTAKQLVSMIHSCEENQEALEDQCLRALEVAKCFRTRIHELDWTPKMDVLVTEVLTDI

>CsasPBP2

MNHVKFLVSVVVALAIDRIDGSADVMREMTLRVGKVIEACKTELDIPDNKMEDFVNFWKEGYELQHRETGCVVMCMSSKLNLLDPDGKLHHGNAHEFAVSHGADDGMASQLVNLVHGCENSIPNNEDACLKVLDLARCFKDGVHKLNWAPNMDLVIGEILAEV

>CsasPBP3

MPSFVKWPTLVLCSIFLIIYVDKAISSQDIMMNLTKGFAAALDKCKKELNIQDHIMQDFFNFWREEYQLVNKEFGCVVMCMASHFDLINEDSKMHHEKAHAFAKSHGADDVLAKQIVTMIHECEKVHEGMSDDCSRVLEIAKCFRGKIHELKWAPNMETLIEEIMTEV

>EposPBP1

MMNQKELVLFAVVCLSLYQAVEPSQDVVKDMSLNFRKGLDACKKELNLPDTINSDFNRFWNDDHVVTNRDTGCAIMCLSSKLELVSDTGLHHGNTLEYAKQHGADDTVAQQIVDLLHSCAQAVPDLEDPCLKVLEWAKCFKAEIHKLNWAPSAEVMAAEMLAEV

>EposPBP2

MATVSKWRMLVLTLCLTGVWQVESSADVMKKLTTGFATALEKCRDELNLPDAVMQDFFNFWREDYELVNRDMGCAIMCMATKFDLVTEEQKLHHGNAHEFAKSHGADDSMAKQLVTMLHECETQTSSISDDCSRTLEIAKCFRTKIHGLKWAPSMETILEEVMTEV

>EposPBP3

MARLSILVALVVLGVNISEIDSSEEVMKDLTSGFIKVLEECKKELNLSESIINDLYNYWKEDYSLLNRDVGCAIVCMSKKLELIDTSGKIHHGNAEDLAKKHGADSEVAAKLVAILHECEKTHDAIEDQCMKALEIAKCFRTNIHELNWAPKMDVVITEVLTEV

>EposGOBP1

MCQATRALVLAALLAAASATVDVMKDVTLGFGEALKQCRESSQLTEEKMEEFFHFWREDFKFEHRELGCAIQCMSKYFNLLTDGERMHHENTDKFIKSFPNGEVLSKQMVALIHACEQQHDAEPDHCWRILRVAECFKEGCQQRGIAPTMEMLMAEFIMESEA

>EposGOBP2

MASYWAVCVVLVAGSHLVAGTAEVMSHVTAHFGKALEQCREESGLSTAVLEEFQHFWRDDFEVVHRELGCAILCMSNKFSLMQDDARMHHENMHDYVKSFPQGEVLSAKMVELIHNCEKPYDDIKDDCERVVKVAACFKVDAKKAGIAPEVAMIEAVMEKY

**CSP:**

>SinsCSP1

MDTEAIIIHYFTTIAETSIIRMRSLIVICLLALVGITLSNPIETYTDRFDNIDLDEILDNKRLLIPYIKCMLDQGKCSPDGKEIKKNVVEALEHDCHKCTPTQKKGTKKIIRHLINKENNYWIELSHKYDPDRHFVGRYENELTEH

>SinsCSP2

MKTFIVVCLFAVVTIASARPDDKYTDKYDNISLDEILANRRLLIPYIKCILEQGKCAPEGKELKSHIQEALENNCAKCTETQRNGTRRVIGHLINNEEEYWGQLKAKYDPTNKYATKYEDELRSVKA

>SinsCSP3

MNWLFLTIGLTLVCLSIGEQYTDRYDNINVQEVLDNKRLFNAYMKCILDKGSCTPEGRELKSHIKDAIQSSCSKCTDKQKQGARLVVNHIRDKEPKYWEELKSKYDPDDEYKEIYEAFLIAKD

>SinsCSP4

MKTFIVVCFFALVAITTALPGARYTDRYDNIDLDEILGNPRLLNPYILCLLEQGKCTAEGKELKSHIHESLENYCENCTEAQRKGARRVIAHLINNEPESWEKLNAKYDPEHKYTKKYEDELRTITQ

>SinsCSP5

MKVLIVLACLGVAAYAAEKYHSKYDNFDVDTLISNDRLLKSYINCFLDKGRCTPEGSDFKKTLPEAVETVCGKCTEKQKVNIKKVIKAIQQRHPKQWEELVQKNDPSGKHRADFDKFIQSS

>SinsCSP6

MKTIIAFCALVVVVVAFPGDTYDPRYDNFNAQELADNLRLLKSYGKCFLDQGPCTPEGADFKKSIPEALRTTCAKCTPKQRELIRIVVRAFQTKLPDVWEQITKKEDPNGEYKESFEAFLNRSD

>SinsCSP7

MRNWLLCLCALTVVVSCSGQQQHYNRYDNFNTDSIIQNERILLAYYKCVMDKGPCTKDGKIFKRVLPETLTTACSRCSSKQKFVVRKMLLGIRAKSEPRFLELLDKYDPDRSNRDALYNFLVTGN

>SinsCSP8

MKTFIVVCLFAVVTIASARPDDKYTDKYDNISLDEILANRRLLIPYIKCILEQGKCAPEGKELKSHIQEALENNCAKCTETQRNGTRRVIGHLINNEEEYWGQLKAKYDPTNKYATKYEDELRSVKA

>SinsCSP9

MKSAIALAVLVLVVATTTRADEDFYDSKYDEYDLDAIISNKRLLENYINCFLGKGKCTPDGAKFKKILPEALESTCGRCTPKQGILVRKGIRAIQEQLPESWAQLVKTYDPEGKYRNSFEKFLANTD

>SinsCSP10

MNMKTQLIYLLLVYQMKHYNAEETQTYTTKYDNVNLDEILASDRLLTGYINCLLDKGPCTPDGKELKRTLPDAITNDCQKCNTRQREGADQVMHYIIDHRTEDWKELEEKYNSDGSYKRKYLESKTAENNTSSEIKSTHNAEKGINDNKESDEKEE

>SinsCSP11

MKLFLIAVALSMFCILVNAQTYTDKYDTINLDEVLSNKRLLGAYIKCVLEQGRCTPEGKELKSHITDALQTGCSKCTSKQRQGMKRVIKHLITYEDDSWQLLVEKYDPQRIYSHKYEKELNSL

>SinsCSP12

MRAVFFLCISACAIVVGQDINTMQNMPTYDARYDYLDVDAILDSKRLVTNYVNCLINEKPCTPEGKALKRLLPEALRTKCVRCSERQKRTAVKVIRRLKNEYPDEWAKVASRWDPTGDFSRYFEEYTGRDQTNTIFNTGNEVPASSPPPPLPLNVPLRTTAPVAPAQPTSPQVPIARSTSAPPIILNRFGDDGVLIVSSSSPSNTASTVTPRSSNTIRSVIQTRATPATWAGAASDIIPTQVPVRPANEIPPSYSTAMTIIDEIGHKIIRTTELVSDILRNTVRAVVG

>EhipCSP17

MKTFIVVCFFALVAIATALPGARYTDRYDNIDLDEILGNPRLLNPYILCLLEQGKCTAEGKELKSHIHESLENYCENCTEAQRKGARRVIGHLINNEPESWEKLNAKYDPEHKYTKKYEDELRTIKQ

>EhipCSP16

MKTFIVVCLFAVVTIASARPDDKYTDKYDKINLDEILENRRLLIPYLKCILEQGKCSPEGKELKSHIQEALENYCAKCTETQRNGTRRVIGHLINNEEEYWGQLKAKYDPTNKYVTKYEDDLRSVKA

>EhipCSP15

MRNWLLCLCALTVVVSCSGQQQHYNRYDNFNTDSIIQNERILLAYYKCVMDKGPCTKDGKIFKRVLPETLTTACSRCSSKQKLVVRKMLLGIRAKSEPRFLELLDKYDPDRSNRDALYNFLVTGN

>EhipCSP14

MIMKCVIALICVLGMVIADEKYTDKYDNINLDEILGNKRLLQAYVNCVLDKGKCSPEGKELRDNVEEALQTGCAKCTEAQDKGASRVIEHLIKNEKEIWGELTAKYDPEGKYRKKYEDLAKSKGIEIPEN

>EhipCSP13

MKVLIVLACLGVAAYAAEKYHSKYDNFDVDTLISNDRLLKSYINCFLDKGRCTPEGSDFKKTLPEAVETVCGKCTEKQKVNIKKVIKAIQQRHPKQWEELVQKNDPSGKHRADFDKFIQSS

>EhipCSP11

MKLFLIAVALSMYCILVNAQTYTDKYDTINLDEVLSNKRLLGAYIKCVLEQGRCTPEGKELKSHITDALQTGCSKCTSKQRQGMKRVIKHLITYEDDSWQLLVEKYDPQRIYSHKYEKELNSL

>EhipCSP10

MISLKHCIISKRKEIIVNMKTQLIYLLVVYQMKHYNAEETQTYTTKYDNMNLDEILASDRLLTGYINCLLDKGPCTPDGRELKRTLPDAITNDCQKCNTRQREGADQVMHYIIDHRTEDWKELEEKYNSDGSYKRKYLESKTAENNTSSELKSTHNAEKGINDNKESDEKEE

>EhipCSP9

MYVNILHTKIGCTEKATMKSTIALAVLVLVVATTTRAGGDFYDSKYDEYDVDAIISNKRLLENYINCFLGKGKCTPDGAKFKKILPEALESTCGRCTPKQRILVRKGIRAIQKQLPESWAQLVKTYDPEGKYRNSFEKFLAETD

>EhipCSP8

MKSLLLIALACLVTVAWCRPGSTYTDKWDHINVDEILESQRLLKAYVDCLLDKGRCSPDGKALKDTLPDALEHECSKCTPKQKEGSDKVIRHLVNKRPELWKELASKYDPEGKYQEKYEDKIKNVKEN

>EhipCSP7

MQILLLSVICACALSLWPVWAAPAPPVPQMTDAQLERSLADRATMQRHLRCALGEGPCDPVSRRLRILAPLVLRGACPQCSPQEARHIRRTLAYVQKNYPWEWAKIVRQYG

>EhipCSP6

MKTIIAFCALVVVVVAFPGDTYDPRYDNFNAQELADNLRLLKSYGKCFLDEGPCTPEGADFKKSIPEALRTTCAKCTPKQRELIRIVVRAFQTKLPDVWEQLAKKEDPNGEYKESFEAFLNRSD

>EhipCSP4

MKIFIILFAVMAIAIAAEETYSSEYDNLDVEAVVNNPQTLQAYFGCFIDRDNCEKEPGNFKKDLSEAIKTACAKCTPAQKHILKRFTEGLKEKFPQDYETFKQKFDPEGKYFVALEPVLAKA

>EhipCSP3

MNWLFLTIGLTLVCLSIGEQYTDRYDNINVQEVLDNKRLFNAYMKCILDKGSCTPEGRELKSHIKDAIQSSCSKCTDKQKQGARLVVNHIRDKEPKYWEELKIKYDPDDQYKEIYEAFLIAKD

>EhipCSP2

MQLCRVFLFCCVAAAAAAQAQRPQVTDTALEDALNDKRFIQRQLKCALGEAPCDPIGKRLKTLAPLVLRGACPQCSPQETKQIQRTLSYVQRNYPQEWAKIVRQYAG

>EhipCSP1

MRSFIVICLLALVGITLSNPIETYTDRFDHIDLDEILDNRRLLIPYIKCMLDQGKCSPDGKEIKKNVVEALEHDCHKCTPTQKKGTKKIIRHLINKENDYWIELSHKYDPDRHFVGRYENELTEH

>AipsCSP8

MNFLVLSMVIALAGFVAAETYTDRYDHINIDEIIENRKLLVPYIKCTLDQGRCTPEGRELKAHIKDAMQTSCSKCTPKQRKGARKVVKHIRAKEQEYWNQILAKYDPENQYSENYEAFLAADD

>AipsCSP7

MKFVLLLCVMVAVVYAEDKYTDKFDNIDLDEILTNRRLLLSYFNCVMGKGKCTAEGKELKDNLEDAIKTGCAKCTENQEKGSYRVIEHLIKNELDLWRELCAKFDPTGEWRQKYEDRARANGIEIPKD

>AipsCSP6

MKLIIAVALLCMVAASWGKPASTYTDKWDNINVDEILESQRLLKAYVDCLMDRGRCTPDGKALKETLPDALENECSKCTEKQKSGSDKVIRHLVNKRPDLWKELSTKYDPDNIYQDKYKTQIESVKQ

>AipsCSP5

MQIKYALLLCCVAAMSVAQTQRPAVSDTALEDALQDKRFIQRQLKCALGEAPCDPIGKRLKTLAPLVLRGACPQCTPQETKQIQRTLSYVQRNFPQQWAKIVRQYAG

>AipsCSP4

MKVVLLTLCFALGVLAQDKYESVNDDFDVSKVLNNDRLLQSYAKCLLNKGPCTSEVKEVKAKLPEALETRCAKCTDKQKQMGKVLAQEVKKNHPDIWKELVAMYDPQGKYQEAWKEFLQE

>AipsCSP3

MNSFIVLCIASLAVMAYARPEEAKYTDRYDNVDLDEVLSNRRLLVPYVKCILDQGKCAPDGKELKEHIREALENECGKCTETQRKGTRRVIAHLINNEADYWNELTVKYDPQRKFTAKYEKELKEIKQ

>AipsCSP2

MKIILALCVLVAAVSAYDTRYDDFDVETLVGNVRLLKSYGHCFLGTGPCTPEGTDFKKTIPDALQSGCGKCSPKQKHLIRTVVKGFQTKTPDIWQQLVKKEDPHGEYKEIFTRFINGSD

>AipsCSP1

MKAVIVLCALVVAVCARPEEEKYPDKYDNTNYKEILENGRLYRAYCDCLLDAGKCTPEGKELKSRIKDALETKCEKCTDKQKEAVRYVIKYLINKKPEDWKKVCDKYDPDGKLKSQYEKELKDL

>HarmCSP25

MRAVLFVCALVYAVAAQDVSDMVNMPKYDSRYDYLDVDAVFTNKRLVRNYVDCLINAVRCTPEGKALKRILPEALRTKCVRCTERQKRTAVKVIKRLKNEYPDEWAKLASRWDPTGDFTRYFEEFLAKEQFNTIPGSAGIGSEIPTSSPLAPPRAPTVAPTVATPTAAATEPTPPRPVVLNRFGDEGELMMGSPSSAGITPRPMTQATTRPTTTMRPVNTRPVPPRPTMMTWAGAASNTQPTRFPLRPSPSDVPPPYSTAITLIDQIGYKIIKTTELVTDLLRNTVRAVVGR

>HarmCSP26

MDSRIAVVCVVLAAFAVDQTVGAPQKDAVAASGPAYTTKYDHIDVDQVLASKRLVNSYVQCLLDKKPCTPEGAELRKILPDALKTQCAKCNATQKNAALKVV

>HarmCSP24

MKTAIVLLLALFGVVLTARFDNIDIDKVLGNQRVLESYLKCMYDEGPCTPEGRDLREKAPEALETNCKDCTDNQKALVRKASLFLIKNRPDDWKKLSDKFDPEGKYKKAFDEFLKEKN

>HarmCSP23

MKAVFLLCLVVVAVSARPEAQYTNKYDNVNLDEILVNKRLLVPYIKCALDQGKCSPDGRELKSHIREALENYCAKCTPVQQDGTRRVIAHLIKHKLEEWEKLKAKYDPEGKYTHKYEKELEEVQH

>HarmCSP22

MQTRYAVVLCCVVAACVAQTQRPPVSDSALEDALQDKRFIQRQLKCALGEAPCDPIGKRLKTLAPLVLRGACPQCTPQETKQIQRTLSYVQRNFPQQWAKIVRQYAG

>HarmCSP21

MKSLLLLCLVIAAVWARPETYDTRYDDFDAETLVENVRLLKAYGHCFLGTGPCTPEGSDFKKTIPDALRTGCGKCTAKQRHLIRVVVQGFRSKTPDLWQQLVKKEDPNGQYKEVFTRFLNGSD

>HarmCSP20

MKLIVAVALLCLVAESWAASTYTDKWDNINVDEILESQRLLKAYVDCLLDRGRCTPDGKALKETLPDALENECSKCTDKQKSGSDKVIRHLVNKRPEMWKELSAKYDPNNIYQDRYKDKIEAVKGQ

>HarmCSP19

MNSLIVFCVLSLAALTIARPDGATYTDKYDNVDLDEILGNRRLMVPYIKCMLDQGKCAPDAKELKEHIREALENGCAKCTDKQKEGTRRVIAHLIKHKNADWQKLKAKYDPEGKYTHKYEKELEEVQH

>HarmCSP17

MKLLVVAIVVTLAAFAVAETYTDRYDNINIDEIIENRKLLVPYIKCVLEQGRCTPEGRELKAHIKDALQTSCTKCTQKQRKASRKVVKHIRANELDYWKQLLAKYDPDSIYVKNYESFLAADD

>HarmCSP16

MKILVLLLAAVVTAQYEEDTYGTDHDDLDIVALVEDKDQFNSFIDCFIDEAPCDDVAETFKSVIPEAVLEVCAKCTPAQKHIVRVFNESFKKKMPEKFQKFKNKYDPEGKYFENFEAAVGAF

>HarmCSP15

RPESQYTNKYDNVNLDEILVNKRLLVPYIKCALDQGKCSPDGRELKSHIREALENYCAKCTPVQQDGTRRVIAHLINHEPDYWRQLSVKYDRDGKFAVKYEKELRTIA

>HarmCSP14

MNSAIVLCVVALAGMVLARPDGDGDKYTSRWDDVDLDEILENDHLLIPYIKCSLDEGKCAPDAKELKEHIQEALETGCAKCTDKQKEGTRRVIAHLIKKKLQEWEKLKAKYDPEGKYAKKYEKELEEVKNA

>HarmCSP13

MKVLLVLCLFAAAALADDKYTDKYDNINLDEILENKRLLLAYVNCVMERGKCSPEGKELKEHLQDAIETGRSKCTEAQEKGAYKVIEHLIKNELDIWRELAAKYDPKGDWRKKYEDRARANGIQIPE

>HarmCSP12

MNSAIVLCVVALAGMVLARPDGDGDKYTSKWDNIDLDEILGNDRLLVPYIKCALDEGKCAPDAKELKEHILEALETGCDKCTDKQKEGTHRVIAHLIKYKLEEWEKLRAKYDPEGKYAKKYEKELEELKRA

>HarmCSP11

MNSAIVLCVVALAGMVLARPDGGTYTTKYDNVDLDEILANDRLLIPYIKCLLDEGKCAPDAKELKEHIREALENGCAKCTDKQKEGTRRVIAHLIKHKNADWQKLKAKYDPEGKYTHKYEKELEEVQH

>HarmCSP10

MKVLVVLSCLIVAAFAADKYNAKYDNFDVDTLITNDRLLKAYINCFLDKGRCTPEGSDFKKTLPEAIETTCGKCTDKQKNNIRKVIKAIQQKHPKEWDALVKKNDPSGKHRANFDKFIQGSR

>HarmCSP9

MNSLIVFCVLSLAALTIARPDGATYTDKYDNVDLDEILGNRRLMVPYIKCMLDQGKCAPDAKELKEHIKEALENECGKCTEAQKKGTRRVIGHLINHEADFWNELAAKYDPERKYTTKYEKELKEVEA

>HarmCSP8

MKCIYVLSFLLALAAVQAEDKYSTENDNLDIDAVVANVDTLTSFVACFVDQEPCDAVAADFKKDIQEAVTTRCAKCTDAQKHIFYKFILGLKEELPRGYEEFGRKYDPENKHFSALENAVSPA

>BmorCSP10

MACVAVTWARPESTYTDKWDNINVDEILESNRLLKGYVDCLLGKGRCTPDGKALKETLPDALEHECVKCTGKQKSGADKVIRHLVNKRPDLWKELAVKYDPDNIYQARYKDKIDAVKGSA

>BmorCSP1

MKVLIVLSCVLVAVLADDKYTDKYDKINLQEILENKRLLESYMDCVLGKGKCTPEGKELKDHLQEALETGCEKCTEAQEKGAETSIDYLIKNELEIWKELTAHFDPDGKWRKKYEDRAKAKGIVIPE

>BmorCSP2

MKLLLVFLGLFLAVLAQDKYEPIDDSFDASEVLSNERLLKSYTKCLLNQGPCTAELKKIKDKIPEALETHCAKCTDKQKQMAKQLAQGIKKTHPELWDEFITFYDPQGKYQTSFKDFLES

>BmorCSP15

MIENFYSKCTISKSVLFLCLIFLPYALNQKYYDSRYDYYDIDHLVQNPRLLKKYLDCFLGKGPCTPIGRLFKQVMPEVITTACAKCTPTQKRFARKTFNAFRRYFPETLMELRRKFDPESKYYDAFEKVITNA

>CpomCSP13

MKVVLVLCILALAAARPDEGYSTRYDNFDAQELVDNVRLLKSYGNCFLDKGPCTAEGSDFKKLIPDALKTSCAKCSPKQRNLIRIVTKGFQTKLPEIWKELAAKEDPNNEYHDAFNKFLNESD

>CpomCSP14

MQMKLSLLVVASVLACVCAQARPPVSDTALEDALNDKRFIQRQLKCALGEAPCDPVGKRLKTLAPLVLRGACPQCTPQETKQIQRTLSYVQRNFPAEWAKIVRQYAG

>CpomCSP15

MRVFLFCFLVCCVVSDEYYNRRYDYFDVDTLVENPRLLQKYMECFLDKGPCTPVGRVFKRVLPELVATGCAKCTPSQRRFAKRTFEAFKRDLPESHAELKRKIDPTNQNYENFERKIADA

>SexiCSP1

MKSFIVLCLFGLAAVAMARPDGSTYTDRYDNINLDEILGNRRLLTPYIKCILEEGKCTPDGKELKSHIREALEQNCAKCTDAQRNGTRRVLGHLINNEEESWNRLKAKYDPQSKYTVKYELELRKLKQ

>SexiCSP2

MKSMIVLCVLSVAALVVARPDDSHYTDRYDNVDLDEILSNRRLLVPYIKCILDQGKCAPDAKELKEHIREALENECGKCTETQKKGTRRVIEYLINNEEEYWNELTAKYDPERKYTTKYEKELKKIKA

>SexiCSP3

MKVVFLVFVLTAVVYSHPHDSHYTDKYDNIDLDEILNNKKILTSYINCCLDLGKCTPDGKELKSHIREALENKCGKCTEAQKNGTRKVMTHLINFEPDYWNQLCAKYDPEGKYKAMYEKEYKTLVH

>SexiCSP4

MKCIYVLSVLLAFAAVQAEDKYSTENDDLDIEAVVADLDTLKGFVGCFMDAMTCHAVAADFKKDIPDAVATSCAKCTNAQKHIFHKFLLGLKEKLPSDYEAFKKKFDPQGQYFEALEAAVASS

>SexiCSP5

MIGLNKYNVPVSIILIFLFVSTVLSQEKFYDRRYDYYEIDTLIQNPRLLKKYLDCFLGKGPCTPIGRVFRQILPEAVQTACKKCTPSQRRLARKTFNAFKGYFPETHEELRKKLDPKNKYYEAFEKAISSA

>SexiCSP6

MKLIVVVALCLVAVAWAKPASTYTDKWDNINVDEILESQRLLKAYVDCLLDRGRCTPDGKALKETLPDALEHECSKCTEKQKKSSDKVIRHLVNKRPDLWQELSGKYDPENIYQERYKNQLDAVKRQ

>SexiCSP7

MKFVLVLCLMAAAVLADDEKYTSKYDNIDLDEILTNKRLLTAYVNCIMERGKCSPEGKELKEHLVDAIETGCTKCTENQEKGAYKVIEHLIKNELDIWRELTGKYDPSGKWRKTYEDRAKANGIIIPE

>SexiCSP8

MQIVVVLVVACVGLVAGLHVQAGPQMTDAQLEQTLADKSTMQRHIKCALGEGPCDPVGRRLRTLAPLVLRGACPQCSMQETRQIRRTLAFVQRNYPWEWAKIVRQYG

>SexiCSP10

MRSWLLCLCVLTVVVSCYSQANRYENFNPDAIVQNDRILLAYYKCVMDKGPCTRDGKNFKRVLPETLATACGRCNPKQKTIVRKLLLGIRSKSEPRFLELLDKYNPDRSNRDALYAFLVTGA

>SexiCSP11

MRVLVVLSCLVVVAFAADKYNPKYDNFDVDTLISNDRLLKAYINCFLEKGRCTPEGSDFKKALPEAIETTCAKCTDKQKGNIRKVIKAIQQKHPKEWEDLVKKNDPSGKHRGNFDKFIQGSS

>SexiCSP12

MKLVIILALVAIALARPDDGFYDKKYDNFNADELIENDRLLKSYAHCFLEDGKCTPEGNDFKKWIPEATTTSCGKCTDKQKVLVAKTIKAIKEKLPAEYEALVKKHDPEHKHHDDLNKFLEKYAP

>SexiCSP13

MKSILVLCLLVTAVSCRPETYDTRYDNFDVEALVGNVRLLTAYGHCFLGTGPCTPEGSDFKKTIPDALRTGCGKCSPKQRHLIRVVVQGFQNKTPALWQQLVKKEDPNGEYKEIFTRFLNAKD

>SexiCSP14

MRAVLFLCALVHVVVGQDVNDMVNMPKYDQRYDYLDVDAIFANKRLVRNYVDCLINAVRCTPEGKALKRILPEALRTKCVRCTERQKRTAVKVIKRLKNEYPDEWSKLASRWDPTGDFTRYFEEFLAKEHYNTIPGSGSALPTSAPIAPPRVSPLPPSTTPTPGPTESTPPRPLVLNRFGDDGELMMGSPSSAGVTPRPMTQATTRPSTTTKTPSTRPIPPRPTMMTWAGAASNTQSTRFPLRPVSEISPPYSTAITLIDQIGYKIIKTTELVTDTLRNTVRAVVGR

>SexiCSP16

MNALLIAVFALAAPLVLGYDEKYDKLDVDKILGDDALFTAYIDCMLDKGPCSVEHSADFRQLLPEVISTACAKCSAIQRQNVRKTVKALSEKKPDDFAQFRTKFDPKGEYEKAFSAFVIGTD

>SexiCSP18

MKGITMICALGVLACAVASPADHYTDRFDNINIDDILNNPRLLNAYINCVLDKGKCTSEGKELKSHISDALENHCEKCTEKQRQGTRTVLAYLINNKPATWNQLTAKSIPMEICRSV

>SexiCSP19

MKYILVALVATIAVVKAQETYGTQYDNVNGEAIVSDDQQFQSFVDCFMGAATCNEPAAAFKKVLPEAIVQACAKCNPAQKHLVRVFLEAYSKKMPQEYEKFKDLFDPERKYFPKFEASVAGF

>SexiCSP20

MQIKYALVLCCVAAVSVAQSQRPPVSDTALDDALQDKRFIQRQLKCALGEGPCDPIGKRLKTLAPLVLRGACPQCTPQETKQIQRTLSYVQRNYPQQWAKIVRQYAG

>SlitCSP13

MKCIYVLSVLLVFAAVQADEKYSSENDDLDIDAVVADVDALKGFVGCFMDAVTCHAVAADFKKDLPEAVATSCSKCTDAQKHIFHRFLLGLKQKLPADYEAFKKKFDPEGLHFHTLEANVANS

>SlitCSP12

MKVALLTLCFALGVLAQDMYENANDNFDISEVLGNERLLNSYAKCLLNKGPCTPEVKQVKDKLPEALETRCAKCTDKQKQMGKTLAQEVKKNHPDIWKQLVAMYDPEGKYQQAWKDFLQE

>SlitCSPA11

MRSWLLCLCVLTVVVSCYSQANRYENFNPDAIVQNDRILLAYYKCVMDKGPCTRDGKNFKRVLPETLATACGRCNPKQK

>SlitCSP10

MKLIIVLALCVVAVAWARPASTYTDKWDNINVDEILESQRLLKAYVDCLLDRGRCTPDGKALKETLPDALEHECSKCTEKQKKSSDKVIRHLVNKRPDLWQELSGKYDPENIYQERYKTQLDAVKRH

>SlitCSP9

MQIKYALVLCCVAAVSVAQTQRPPVSDTALDDALQDKRFIQRQLKCALGEGPCDPIGKRLKTLAPLVLRGACPQCSPQETKQIQRTLSYVQRNYPQQWAKIVRQYAG

>SlitCSP8

MKFVLVLCLMTAAVLAEDEKYPSKYDNIDLDEILANKRLLTAYVNCIMERGKCSPEGKELKEHLVDAIETGCSKCTEAQEKGAYKVIEHLIQNELDTWHELTDKYDSSGKWRKTYEDRARANGIVIPE

>SlitCSP7

MKVLVALSIFVVLAAAVPITKDEFATLEAFDYDALFADEENRKMVFDCLLDKGDCGPYKQIVELSMKIILSNCAECSPSQKAKYDHVLKVLKEDYASFFNELMQKAASKKEKH

>SlitCSP6

MKSILVLCLLVAAVSCRPESYDTRYDNFDVEALVGNVRLLTAYGHCFLGNGPCTPEGSAFKKTIPDALRTGCGKCSPKQRHLIRVVVQGFQNKTPALWQDLVKKQDPNGQYKEIFTRFLNGRD

>SlitCSP5

MKSFIVLCLFGLAAVALAKPNGSTYTDRYDNVNLDEILGNRRLLTPYIKCILEEGKCTADGKELKSHIREALEQNCAKCTDAQRSGTRRVLGHIINNEEESWNRLKAKYDPESKYTVKYELELRKLKQ

>SlitCSP4

MRVVLLLCALVHLVVGQDVNDMVNMPKYDQRYDYLDVDAIFTNKRLVRNYVDCLINAVRCTPEGKALKRILPEALRTKCVRCTERQKRTAVKVIKRLKNEYPDEWSKLASRWDPTGDFTRYFEEFLAKEHYNTIPGSGSALPTSSPLAPPRVPQSPPATNPTPGPTEPTPPRPIVLNRFGDDGELMMGSPSSAGITPRPMTQATTRPSTTTRPPSTRPVPPRPTMMTWAGAASNTQATRFPLRPVSEISPPYSTAITLIDQIGYKIIKTTELVTDLLRNTVRAVVGR

>SlitCSP3

MNFLVLSIVVTMAAFVAAETYTDRYDHINIDEIIENRKLLVPYIKCTLDQGRCTPEGRELKAHIKDAMQTSCSKCTEKQKKGARKVVRHIRAKEQEYWKQILAKYDPEDQYKENYETFLAAED

>SlitCSP2

MKSMIVLCVLSVAALVVARPDDSHYTDRYDNVNLDEILSNRRLLVPYVKCILDQGKCAPDAKELKEHIREALENECGKCTETQKNGTRRVIEYLINNEEEYWNELTVKYDPERKYTAKYEKELKKIKA

>SlitCSP1

MRVLVVLSCLVVVVFAADKYNPKYDNFDVETLISNDRLLKAYINCFLEKGRCTPEGSDFKKALPEAIETTCAKCTDKQKGNIRKVIKAIQQKHPKEWEDLVKKNDPSGKHRGNFDKFIQGSS

**OR:**

>SinsOR3

MKPKNIVLKPPEKKFYTFSETFRFCAFALAIALIYPNKNNIIRRWITVISVIFFNSVILFWFVSYLIKCLISVDIYNFARTVTVGVVVVLFLFKSFYVNWKNEEFEKLLNKISKDLLKGNYMDEDYQRIYEYHIKQAKIPQICWLIIPTILSLQFPLYASTGLIYETLNSDVGKKYMVFEMQLKYIEDKQYVSPYYEIIFAYSLVPCLILVPNFAGFDSSFCIATTHMRLKLKLMTHKVHRAFKDARNRSDLQMKVKEAIKDHQEALEFHRGIQEVYGGWLLAVFLLTSFLISFNIYQIYISKRIDPKYAIFTLNGVLHMYMPCYFASSLIKVNEELSTDLYNASWEDWADPAVTKLLVFMMAKSQQRLVITGKRIVVYNMDLFISILHMSYSFFTLITAK

>SinsOR7

MENTKKVQPFDALSVSYKILILCGFFKLMRPTTRFKYICFQIYRPLSFLIVLVFIVQHSIYAVMKVMDNELDKALDAFIVIPPELNLLSKFLALNLHSSTVDKLNDVMRDSIFDARNQEDEIILTKFVSDMHQLTKNVEIGMVVAEILYILSPIFKRIYDPASTIASYYPFKVDNWGRHTITLLWECSFLPWIGNGHLSLDCLIGIYYSQATTQLKLIKYNLEHLFDSDERNKDNSRNMEHQYIDVVDNTIQERFEHYVERYSKVKWYIQELHNVFSGAIIYQFASTIIIACPVIYKISFMDFFSVQCIYLTGYLIMLEIQIVLYCYYGGLVEYESISINDSLYMSEWLSASPKFRRQMLIAMAQWSRPLTPRVFAIVPLSLNTVIAMLKFIYSLYTALISTNNIN

>SinsOR8

MKYVGCFRIHFTILGIAGIWMPRSFENNVRLKFYYSVYRIFFLTLFLVGIVYTQFRFFILVIGDIEKTVDSSVLFFTILPHIIKIYTLISCRERIIRLLDIIERIPDKNNILQMFSKNVALISSAYFCTCIGTAILWCVYPFMKPVLTLPFYYPYISQESFLFPVLYVYQTFGIIISALTIASEDFLAGGLMALAAAQLELLCCDLSTIGENKENINGNIDQYYEKIVTCIKFHAKIISFVKELSTIYGLSVFGQFLFSGILLCESAFIIITSDVFTESVTMFLYLLCLLGQLLLYCFCGNMIKTNSDKVAAAAYSSRWEMTSLPTQKALLFLIMRSQMTLTVTPGGIFDLSLVTFSAVLKSSYSFLAVLNQKHNS

>SinsOR10

MNTLISAKEILAIKHLKIMRIILSLNGAWPGEVLGEKTPLILKCHRLYIPFQALGLFIAQVCYLVKYFKVLNIFSMGHMYITTFLTVLICIRGITVCLKKYRDISRQFLMAFHLIHFKHKSDYHEKIYGIVNKISYYFTIYLLLLTMCGAILFNGSPVYNNYKMGAFTGEKRENITLEFSIYYYFYPGFNAEDYFIVCTIYNFYLSFCTAICVCFLDLYLSVMIFQIIGHIQILKNNIENITKPRTVTGNTNKIDDTEPSYSMPFSAEENGSIHTKLVDIVHHHRLIVNFTDDISNFFGPVLASYNLFHLVSGCLLLFECSRGADAFARYGPLTVILFGQLIQISIIFEIVGYMSEKLIDAIYCMPWESMNISNQRSVCILLHRVQTPIQVTAMGLVPVGVQSMATILKSSLSYFAFLRSMDN

>SinsOR11

MSESPTELAKREIDESLILCKFCMRYIGLSFEEPKSRRGYLTQKLMFLLSVCVIFYHVFSEIVYIGLTLSNSPRVEDVVPLFHTFGYGALSIAKMSVLWYKKEKFGELLQELAGIWPMPPLDKAAQTIKSNSLSALRMAHRWYFTTNIAGVWFYNLTPIGIYFYQSLQGQNTEVGYVWMSWYPFDKHEMIAHVAVYIFEVFAGQTSVWIMVGTDLLFSAMASHIGLLLRLLQRRLESLATTHQTDEEYYQEIVSNIRLHQRLIMYCNDLEDAFSLSNLINIVMSSLNICCVVFVIVLLEPFIAVSNKLFLGSALIQIGMLCWYADDIFHANLGVSMAAYNSAWYKTSPRCRRALLFLIKRSQKPIAFTAMKFTNISLVTYSAILTRSYSYFALLYTMYSEH

>SinsOR12

MEQFRQIDCFHINMKFWKLLAIWPDGDTCRYYGFYSKSFVSFFVILYYILLTINFYFLPRHLDNFIEEMIFYFTELVVAAKVFTFLFMRNKIIEILKTLESDMFQPNIPDGFDIILKAKKFNVTYWKIVAIVSFVSNVTHLLSPLIIHLIFSANLELPICSYSFLSKEFKNKFIYPIYFYQCFGMFFHMLYNVNIDTFLLGIIILTIAQIDILDLKLRKLTKKTPDGNMDTKVVQKLRCKDSYYLLELKKSIIHFDQVGKFCALIQQVFSVALFLQFSTSSCIICVCLFRCTLRAPMQYYIFLSTYMFIMIIQIMVPCWFGTRVIEKSYLLTRAIYDCDWISRSPQFKSNLRFFIERANRPLSITGGKMFLLSVATFTSIMNTAYSFFTLLRHMQSR

>SinsOR13

MESISISHTFNVNFFLLKMFGVWSGKSLNNYYKFYTFVIVVVLLLIYNTLLTINLFFTPWKIELIIREVMFYFTEIVVTAKVFMFLGFRSKLLDILNHLDCEEFKATDENSRKIIDKHVYYYKTYWKIFSTLSHLSYFFLVLLPIIIAKLMGTNLELPICKYYFFDDKLRNRYFYYLFIYQSIGMYAQMTYNVNADTLISGLILMAVTQLKVLNYKLSNLKVTAEHSKLSLEIQDNIQYQKLNDWLKHHYLITTFCTKIQNLINVTILIQFGMSAATICVSLCGFLMMSSTGTLMFVCSYLFVMVAEIFVPAWMGTQLSYESRESVFAIYDSEWIPRSEKFKRNMRLFVARANVPIILRGVKMFPLSLETFTSIMKTAYSFFALIRNVQDRENGRIS

>SinsOR16

MTGTAFLLFTNLALFTKTVNVIGRRSAVRKIVDEVNSELQSEQEEKRVAIVESCNQETKRHLCLYIVLTAVTVFGWAASAEKDSLPLRAWYPYDVSQSPAYQITYAHQSFALIVAASLNVSVDTLVTSLIAQCRCQLMLLGCSLRNLCRGIPVRDMQLMSPNEDKTISVLLRKCVLRHQAVLETVKLLEEYFSTPILAQFTVSTVIICVTAYQLAFETMKMVRVISMLAYLLDMMLQVFFYCYQGHQLSEESTEVAGAAYSCPWYSCSVRIRGAILILMTRTTRIAKLTAGGFTNLSLSTFMAIIKASYTFFTVLQQVEGRKP

>SinsOR17

MVKTLTERLEDPERPFLGPHYWLTKKIGLFLPKSKLGIVTSYVIHEIVTYFVVTQYIELYKLIVLKADVDILLFNVRSSMLSVVCIVKSNSFLYWQSKWHDLFEYVTETDKFERETQDEVRVSIINNYTNYCRRITNVYWSLVIFTNFTVIFTPLMKYMTLSDEHLKAIENGTEMFPHVFSAWTPFIDKEHSPGCWITILYHAIICSLGTLMVISYDMNAVVIMVFFGGKLLLFRERCKQLFDSDEAGLSDEEVRNRIRDLHLTYVRLVKYFNLFNSLLSPVMFLYVVMCSLVLCASIYQLTSSKDTMMTKFIMAQYFIFASSQLFLFCWHSNDVLAISEIIMYGPYDSKWWAASVRQRKYVLLLIGQLRKDFTFTAGPFTDLTLSTFIAILKGAYSYYTLVRD

>SinsOR19

MIFQRVISFANGLEDPKHPLLGPNLKGLYVCGLWQSGSKFRIACVNFIYFLGIIFVCTQLVELWIVKHDFMNALHNLSVTTLGITCAVKSASYILWQSRWKELVNAISTEEISQMTQEDSVTQKLRKNYTTYARVVTYLYWYLVVMTNIAMIFSPLLKYATTTIYREEISNRTEPYPLIMSSWFPFDKTKMPWYWISVGVHIWMNIQGGGTVAVYDSNAVVIMIFLKGQMRILREKCKTLFNDSENIQREDILDRIKECHRHHGFLLTQSDLFNSVLSPIMFLYVLVCSIMVCCSVVQFTSEQATTSQKLWVLEYTTALVSQLFLYCWHSNEVLAESNEIDRGVFESDWWKADVHIRKQVILLAGKMGQPFLLSAGPFTTLSVPTFISVIKGSYSFYTLFTQMHENK

>SinsOR20

MFFFKENDMNTVKRPQDLRYMKQLRFSLNIVSAWPHKEIGDPESKFVFWWRAYYAFVEGFFWFLGMAYLKNHYGKLSFYEYSHTLITHFMNTITCQRLTLPFMKKYRDFIGQFIKKFHLFHYKDKSEYAMKIYLRVYKLSDYFTMYLHILMYVGIMFFNFTPVYKNIITDAYNNPINATLQHSSYFELPFDYKHSLTGYVPLFFFNLYITFICASFFCMFDLLLTVIVLNVYGHLKILVYHLEHFMRPSTNSTSHKHKNKMFDTMQFSEEEMKTVTIKLKEVISHQRLITDFIDKMSDIFGPMVCLNLMYQQVSACILLLECSQMDLLVLFSYGPLTFFVFQELIQLSVVFELIGATSDQLIDAVYSVPWECMDTKNRKILYTIMIKSQKTTKFKAMGMVDVGVKTMAAILKTIISYFVMLRTIALQN

>SinsOR21

MEEKLAYYSLVPHFKQLRQTGFYPLDPEAPKLQKFLQKLYNRFSVSLLLLYTTQQIINLYQWRRNINKIMDSMFLFLTYCDCLIKEAAFWNKADEIKGLMNIMKGPIYNQKEHKQILIRTVRQAKLVLRIFNIMCLITCLLWAIFPVIFHLRGRVVEFGIWLPFDSNENPQFYFVVIYVCFQTSWLAFNNSTMDVTMAFFLAQCTTQFFILRQNLENIVKKSIDDSTTLRISFAKAFEKRFIGLMQHYNDIVQSSQKIEEIFGNAVFVQFIFTGWIICTTAYRMINMSPGSIEFLSMILYILCVMTQPYLYCFYGNELMYESDKLMNSAYAMDWLMIAPTQRRYLIIFMERIKRPIQPKAGFVIPLSANTFLTILRTSYTFYAFLKNSY

>SinsOR24

MKVFQYDIEEYDVTYAVPKSVLRLVGLRFMRKDSAFADMCWKVFYWFEFTNLFIVTWLELINMAQVAQGGSFADAVEIFRMMPCVGYLLLAMVKSYRIVLYRPVYENLLSELRGMWPTSAISEEEHGIMDKALKQLKHSIKSYYWCNNALLVVFLSAPFVEIIKRAMGRNVPLILPFFYWFPFDPFQRILYEIILVFQTWHGLISIWFMVGSDLLFCIFLSHITIQFDLLSVRIRKLVYVPVDNQLIDDYPLASYSHEYIQNKKGMAENYNDTEWETLHIGQLSEIITRHRTLIRLSEDVEDIFSFPLLVNFFNSSIIICFCGFCCVVVEKLTEMIYKSFLTTALLQTWVLCWYGQRLLDSSTGVSEALYESGWYKLSKSIRSTILIMIHRGQKEVHVTTYGFSVISLASYTTIIKSAWSYFTLLLNIYKN

>SinsOR25

MKILVDNANLSISLSLTALKLVGFWAPDGLNGISKFLYNCYALFSFMFLLGTYLIIQVVDLYFVWGNLPLMTGTAFLLFTNLAQAAKIINILSRKRLIQKIITEADLVLKGQQTEEGRIIVKNCNRETSLQQLLYFCLTTVTVSGWAGSAEKNQLPLRAWYPYDTSKSPAYELTYMHQVGALFIAAYLNVGKDTLVTALIAQCRCRLRLLGLALRTLCRDVEVTHNHLLTANEEQVVSSRLRNCVCQHQAALTAASELQTCFSAPTFAQFTVSLVIICVTAFQLASVSHTGNLVRLFSMGTYLLNMSFQVFLYCYQGNQLSEESIEIAGAAYFAPWYAFSARLRRAMLVLMTRSRRVARLTAGGFTTLSLTSFMAIIKASYSFFTVLQQVEEKA

>SinsOR26

MLKRLLHKLEDPKRPLLGPNLKALKFWGLLLPENFIMRQIYILLHLSVILFTASECVDVWFVKSNITLLLNNLKITMLATVSVCKISTFLFWQNDWKKIIGYVIEADIMQRKTNDIIKQTIIKKFTKYCRKITYMYWCLMYTTVIIVMVQPIIKYFSSPVYRENVRNGNETYLQVVSSWVPFDKNTITGYLIASVIQSYGAIYGGGWITSYDTNAMVIMVFFRGELELLRRDCANMFGTESSPVTEQVSKERLKDCHRRYVALIEHARLFDSCLSPIMLLYMFVCSVMLCVTAYQITIETNPMQRFLSAEYLVFGVAQLFIYCWHSNDVLYASQDLMLGPYESTWWMHRISYRKNIFILIAQFRKRIVFTAGPFTELTVPTFINV

>SinsOR27

MPHITNMEAEYIIKPPTQQLYYRTLAKMMSLLSLGEQTWWGYKPYGTIVYINSLLVPIFGPLSLTLQIIYLYQNFRILPLNVLGTIFSMLPITALVNIKIRVHKTKKYENLMKSFMTNIHLHNYKEEDSVVKATIIKIERYTRTMAYCIIFLVNFSWILWSVIPLVNNLNNREAIHNKTMLTQSILYMWMPIDYEYDFQNWLITHVINIYLIGTGCAFLAIPDIINYTIAMHLIGHVILLKHKIVSSFPTELNAKEVKQKLKDLVEYHCFIIKLFKDVESVFGINISANYFNNLLIDSILLYQLMNQEKGDRTSLLFGITIVICMGGLIIMSFILEEIRKQSDDLPESLYGISWENWTVSNQKSLLIILAQLQTELAFVGAGGLRTGVTPMVAIIKSTISYYLMLKSTI

>SinsOR29

MHNTSCLRLCLTIMTVTGVWLPTALKSSHLRHIYYIFAFMYHLLFVLPIIFMEFAVFYQVLGNIEQMVDTSLLLVTHIAQCVKVCVMWYKQDEIHSLLMTLDSPTFTREDSNKKQILKDVIQTTYTVSKVFISLVVVTGIFWGIYPMLKPTLCLPIQYPNIPPEYKIFPIVYLYQIVNITLMGIAIASIDFLVGALMAILSAQMDILSYELSLVGKINNKDDNANTDIRQDYLIIISCVKFHENIIKFVKDLEKIFGFPVFFQFLTSAIIICETAFRITDSTETTELITMMFYFMCVNTELLMYCYYGDLLKRKSERVVEMAYCCNWENADVRMQRALLLLMQRAQRTLVLRAGNMFELSMMTFSAILRTSYSYFTVLNERRKLKG

>SinsOR30

MSPIKQSESFKFNFVLLKIFGIWPGSTTNKYYKYYSIVYFVSMLLIYNILLIVNLFYTPQKVELLIREVIFIFTEVVVTTKVLMILLMREKLVVLFDMLDDDIFNGEDAIGTDIILKYNSYYKSYWKMFTVLSLFAYSSQVFLPIIIYFIFNVSNLELPICKYYFLSNQVREKYFWIFLLYQSFGMFGHLIYNVNIDTLMSGLILMAVAQLKLLNHNLRNVKFEKNKSKLKVEIQENIQMTRLNQCLKHYDVLLKYFYKVQEVINVTMFIQFGVASAIICVVMCGLLLSSTTETIMFMVSYLFAMTLEIFVPTWLGTQLSYESQELVFAAYNSEWIPRSESFKRNLRLFMEHANAPLTLTGLKLFPLCLGTFISIMKTAYSFFTLVRNVQDSQGEAI

>SinsOR31

MFAPRKERSIFLNEIHLLSFYLSKLFLFPFFGKTKFKQFGYYSTYFLIVFTSIQLCFTLVLTGLNDFIEIINISPNLGVCVMCAIKYAKVNSNRALYYDIFEHFREDLWKTISEYSAEDLKTITKYTKIFKLTNRTLILYLSLPLIAIVTIFPWILMVYENKVGKEQKLLLPFDGWYPFDKVNWYFVVYIWESFMTGLIILVYAITDALNISFVACICMELKLLGNSLENLINPEDIENITRRKNISRTHENIKRKLNVTIKRHTFLAKISSELNITLGDLMLVNYTFGSLFICLTAFTFTVVDDLYKSLRYFFFFIALIVAVLDQCIMGQSLSDSSEQLAEAIHASNWLYADQQTKRTLLMLLMRTQKPLQLTANGYLVMNLDTFTRICSSSYQFFNLLRTIYQP

>SinsOR32

MEIPTFEESFKLIKRNFWLSGIPLENTKTTIRFFLLYISLLLIIIEETAFLVSKISSEDLLKLTELAPCTCIGVLSLLKFSTLCFKRQKIRQLTDMLEQLHDDISSDVNKKTLVKRDIIFVNKLIKYYTILNLILITVYNFSTLVFIGIHYTNTKEILYSLPYAVIVPFSTDMWPTWLLIYIHSITSGFICVLYFTTIDSLYCIMTSYICCNFIIINNEIRHLETMNPKTLINIVKKHQYTIKLSEDLEDIFTACNLFNVLIGSLEICALGFNLTTGDWAQIPGCILFLLSVLLQIFMMSFFGENILRESTKVGESAFFSKWYETDEISKKIILYIMTRSHAPQKLTAYKFSVIGYGSFIKIISTSWSYFTILKTVYTPSTEVQIE

>SinsOR33

MGLVDSLWRKLTQTKALEESSGKLETLFFESVYRVTYLAGLSSSDTHPVYRAYSIVVKLSIILFVSSELWYLVSETSSMDNIIDNINVTLIHLIAIYRYKKLMDHKDMYKELAKSMESPHFDISTPKRKKLVQFWVIRNERYLKLLLGLGTCTLAAWYVYPLVDDLEYNLSVAVRLPIEYRTPSRYPLAYIVVLITFNYISYFVMVNDLIMQAHLMHLLCQFAVLADCFENIINDCNVETQGISQNKLFLSEKFKEKYLCRLNDLVDQHKFILHHTMTLGKILSTPMLGQLAASSMLICFAGYQVATTVTISLTKFLMSLLYLGYSMFELFIFCRWCDEIKIQSENIRLAVYCSGWERGIAAVPGIKTRLMLIVARANKPMILTAGGLYDLSLNSYTTVVKTSYSALTVLLRLRQE

>SinsOR37

MLKNFVSKLENPDHPSLGPTLWGLQAFGLWQPTKGVANIIYNLKHIFLALFIMRQYVELWIVKSDLDLVLTNLSKTVVTTFCVIKAGTFVFWQKHWRDVIEYVSTLERRQLSEKDDVTKTFIGDYIKYSRNITYLYWFLAIMTVFSLTVAPILVFFLSSKHHKHIKNETMPYPQIMDSWVPFDKSSGYGYWFTVLEATFVCYHGGGIVATYDSNALVIMSFFAGQLKLLKANCARLFGDGVEIVTHKDAMKRIRDCHNHHLLLIRCSKILNSLLSPVMFLYVIICSLMICGSAIQLTTEGTTGMQQLYIAEFVVALIAQLFLYCWHSNDVLFVSEIVQEGVYASNWWSANVRTRRSLVLLGGQLRRNIVFEAGPFTELTTSTFITILKGAYSYYTLLSNKGN

>SinsOR38

MKKILKNETTEVQSPKDQFFYRALAALMTFMCLGNQVWWGYEPYGKIFKINRFIVTFTGPIMAISQFMYLYVYFNQLTADALSIVYCMLPVTLLANIKIRLAKRDIYKNLMLDFMTKIHLYNYKGEEFINKTIKKVERYSHQMGYCLIGIVAFDSLLWCIVPVITNLIHEEAIKNRTMQTQTCLHVWAPFDYRYDFHKWLIVHIIINVYVVAHGCGILGIFDVVFYIIVFHLIGHIKVLKYKIKTQFEGDLDDEEVKKRLVNVIKYHAFIIKFFKDVEAAFGINVSGNYLNNLIADSLMLYNLMIIAQDKGTVIIFVVMTTVCITELILMSFILEEVRIQSDDLPELIYFMPWENWSLSNKKMLVLILLRIQPELAFVAAGGLRAGVRPMTSIIKSTFSYYVMLKSSMRE

>SinsOR40

MSLAGSSVAPHLRVLRRCGFCRQASTASARLRYTHTFYQLFALTVTTVYLIQEIIYSYQARNDMKMLARVMFLLLCHITSLVKQIVFYVDADRIDHLIIQFDNTMYNPEEPSRRRLLAATASSARRLQRVYSNTAILTCILWIVFPIMHRLTGRTVYFAFWTTFDYNSSTIVFVATLLYSFYVTTLVAVANTTMDAFMGTILYQCKTQLRILRMDLEILPERAYDLKKHTNEPYEKALMRLFVNSIEHYEKISETAQLLQDIFGGAILVQFGVGGWILCMTAYKIVDLSLLSIEFASMILFTICILTELFLYCFYGNEVTVESDRLMSSLYAMQWLCTPVAFRRALVLAMERAKRPLRPVAGLIIPLSLETFVTILKSSYTFYAVLRQTK

>SinsOR42

MSKSTKSILRAFCKYVYYAGAGNCWYEDTYRETYLYKAYALISFSIYTTMIFLENLAAWFGSFPEVEKNSAVMFAAIHNIVLPKMFLLLYHKKSIRILNYEMATVGEKIEEKYVMERQARKAKVGIILYVISVYLSLGAYGVESTRKVIVEGAPFYTVVTYLPQYDDSTIVASIFRVVFYITWLYMMLPMMSADCMPITHLITMTYKFITLRHHYRRIREEFDKDLLTMDKRQAAEKLRAGCIEGILMHQKLMFLADEINRIFGI

>SinsOR43

MDDMELRKTHPQKYYLKFICNSLYVLGYGSCWYEETPRTNFHKIFYKIWSGIANFFIVIIVINEIMANFRPNLTAKEQNDLVQFTFGHSLIVAKIVTMYYQRDRIKAVLKKLLEDNRTIFISADIDKSSVKKVKVYCIVLVSTVYLTIVSAYIDGFRAHFNEGIPIRGEITYYPTPLDSGILVNILRFIVEFHWLYIVTVMNLIDCMSYCTLIFLSSQFKLTQTYYNLLRKKYTKNSNKKTCNVLGEEYKKDFLIGIRLHENALWCAHHVQASLGYIYSSQICQSIILIVMCLVKFVTSARNMTVLLANMTYLSAMTVMTGAYMTAGGDITYEASLVSTTMFHSGWDLVVFDKELRTLAVVAIQRSQAPVYMTAFGVIILSYNNLIMVLRSSYSFFAVMY

>SinsOR45

MAIVTFTFGPLAVTISKYVNRGEVDLLLPFLIVYPFNARDIRIWPFVFIHQVWSAAVSIFTVVGPDCLYYACCAYIHIQFRSLQYDIERIVKPDTNESKTGKSDPFWIEFVHLVNRHRELIRCVNLIEATYSKSTLFNFVTSSFLICLTGFNIMAITNIPLAVPFIIFLFMSLLQIYFFCYYGDMIMRSSMAVSSAVYNCQWWRVDAVMTKNMLLVLTRSQKPCKLTAYGFAEVNLRAFTRILSTAWSYFALLKTIYRA

>SinsOR46

MIDVENLYLNRAKFVMKILGVWIPSENELMISKMYRIFMMSLQYLFLLFQTIYIVQIWGDLEAVSQALYLYFTEACLCFKVTIFQINMDMLKELLKRMNSDVFKPLSLKHEKILKLQAKRIKRLLLGFMISSQVTCGLWALKPLFDNAGTRAFPFDMWMPMSPKFSPQYEIGYAYQFLTACMSAYMYFGIDSVALSMVIFGCAQIDIVKDKILSIKPVSHARNDERKKILSENYKKLIECVVQHQAIVTFTDLVENAYHSYLLFQLSGSVGLICMSALLILIVDWYSIQFLSIVTYLSVMISQLFVCCWCGHELTASSEDLHAVLFQSMWYEQDVKFKRALCFVMIRTSRPMVLRAGHYISLSRQTFVSILRMSYSYFAVLNQTQST

>SinsOR47

MIENLQIDFFLKKQKFLFRYNALNLDNNNITRKEIFKRKCLYIINFLWLNTDFIGEVLWLIDGVKNGKSFTELTYIASCIVSCLLANTKAIFHELNEHYISNIIEDLRKLENKGTDPNGSNANKLENKQKQRIIEEETNFLNIVLTAEMFLHIVPLVGFVLRPLIQIGLTYYNTNEIHFVLPFLVVFPFDTYDIKYWPFIYVHQIWSGTLVMLNIGAVDSFFFICCAFIKIQFRLLKHDIEKIIPTSEISKSKLRFKLRDLIKWHQELIRLVNLVDIVNSKTILVNFILSTFLICLCGFNATINDETAVVMTFTSFLLTCLLQIYLLCFFGDMIVRSSSEIADAAYICRWYSTNPTFARKLLIIQTRAKIPCKLTAFGYADVDLQAFTKILSTSWSYFALLNTVYTSSRSN

>SinsOR48

MKILTEHVKKKLSILLPILPYGVLESWENLDPRLYHAVHIYWLKFYGMWYNNFSPKSTLFWLQLMYTMIVLSLVCFLPGIGEVVYLLRRRDNIGDIAEGLYLFLSEMYTYFKIGVFWLNKDKIVSLLQYLYCEEFKPKETEHKEILRKSIKSARFVMTYYSTICVGAVSVGCIIPISENFQVLPTNVEYPFFDVYKSPVYETMYIHHIYYKPATCIIDGVMDTILAAFVASAIGQLEILAYNLRNFDVIAERKRIRDSNRMNTEVNSKENYMKTVLKDCIKHHNSIIRYVSMIESAFSLASALQFMLSVMVLCLVGIQFLSIENPSSHPTQIVWMAIYLTCMLIEVFILCWFGDVLIWKSMELRQAAFEGPWLNVNPKTAMFIIIFLERCKRPLRVTAGKIFTLSLDTYTDLINWAYKAFAVMRNMKK

>SinsOR49

MKPVSRMKYWAHFSYRFIVWTLVVMYNLQHVIRVIKSRHSTEEVVDTLFVLLTTLNTLGKQIAFNSRSRRIDRLITAINGPYFAPTNSYDVRVLKENAVTMSRLLLLYHIAIFICGALWTIFPLVNRATSENVEFTGYFPFDTTPSPVFELALAYMSFLITLQAYGNVTMDCTIVAFYAQGKIQLQMLRYDLEHLVDVKDGFKEIDSKLHEADNQYFTYVDIENVRFKQKIQERLVRCVKHYQQIVWFINEVESIFGEALVIQLVVMAWVICMTMYKIAGLSLMSAEFISMAMYLGCMLAQLFIYCYYGTQLKFESEFVNQSIFFGNWLALSPGFRRQLLLMMMRYSRAITPRIAYVIPMSLETYIQVLRSSYTLFTFLDRK

>SinsOR50

MSGGATGRPRRYFALHFLLLRFLGLGWWHHPDEGDTSNFPGWYLYYSIITEVIWVAGFVGLESIDPFIGQKDIDRFMFSLSFVITHDLTLIKLYIFFFKNGDIQDIVRTLEIDLHQFYQNDKINRATIRITKILTGAFLFFGWITIGNTNVYGIIQDIRWKAEVATLNDSGLRPPRTLPQPIYIPWNYQSDVSYISTFVLETVGLLWTGHIVMTIDTFIGSVILHMSSQFSILQEAITTAYDRTMSQLYGNLRRDFREHNRDPLTTIDGSHDMDENENRERFVRARYSEKEIESALEETLKNCFRQHQVLINCVEKFAKTYSYGFMTQLLSSMAAICVVMVQVSQDASSFKSIRLVTSLAFFMAMIIQLAIQCFTGNELTLQAGLVSDAVMACNWERMPARLRRDLVLVMVRAQRPLHLTAAGFAYMDNRCFLAIMKAAYSYYAVLSQKQVR

>SinsOR53

MDKKENIRIFTGNLLNFLEIPNHPCIGPHLKLLGLTGLWHPNKNSPVTKFKRILFYVTVTFFCSQYIKCLFNIDVISLALILQYAPFHMGIVKSCYFQRHYKKWERLIIYMSSVECAQLADRRTDLIPIMNGYIKKSRRVTYFFWALAFFSNFAIFSEPYQKNHVMENSTAVYTKIFDGIAPFNQEKPPGYYVSMVLQTIFGHIVSAYVVAWDTLIVSIMVFFSGQLKVSRWYFVQVIDVNSIEKSHQNIVKCHHFYTTLVSYQKMFNSLISPVMFIYLIIISVNLGVCIIEIAQLQDDIVTLVSSCLFVLACIIQLLIFYWYANEVTEVNNLVGYGIFESEWMQLDKSLQKEIALLGLITTKKIVFKAGPLNEMSLSTFVGILRTSYSFYTLLSKTKN

>SinsORCO

MMTKVKAQGLVSDLMPNIKLMQMAGHFLFNYHSDNSGMSTLLRKIYASAHAVFIVTQYLAMVANMAMYSDEVNELTANTITVLFFAHSIIKLIFFALNSKNFYRTLAIWNQSNSHPLFTESDARYHQLALTKMRRLLYFICGMTILSVLSWVTITFFGESVYMLVNKETNETLTEPAPRLPVKAWYPFNAMSGTMYIVAFVFQVYWLLIAMAIANLMDVMFCSWLIFACEQLQHLKSIMKPLMELSASLDTYRPNTAELFKVSSTEKSEKVPDPVDLDIRGIYSTQQDFGMTLRGAGGRLQTFGQNLNNPNGLTQKQEMLARSAIKYWVERHKHIVRLVASIGDTYGTDLLFHMLVSTITLTLLAYQATKINGLNVYAFSTLGYLGYTLGQVFHFCIFGNRLIEESSSVMEAAYSCQWYDGSEEAKTFVQIVCQQCQKAMSISGAKFFTVSLDLFASVLGAVVTYFMVLVQLK

>EhipPR3

MNTLISASEILAIKHLKIMRFILSSNGSWPGEVLGEKTPIILKCYRFYIPLQALGLLIAQVCYLVKYFKVLNIFSMGHMYITTFITVLICIRGITVCLKKYRDISRQFLMAFHLIHFRHKSDYHEKIYGIVDKVSYYFTIYLLFITLCAASLFNGLPVYNNYKMGAFTGEKRENITLEFSVYYYLYPGFNAEDYFIVCTIYNFYLSLTTAICICFLDLYLSIMIFQIIGHIQILKNNIENITKPRTVTGKTNRIDDTEPSYSMPFSAEENDAIHTKLVDIVHHHRLIVNFTDDISNFFGPVLASYNLFHLVSGCLLLFECSRGGDALARYGPLTVILFGQLMQISIIFEIVGYMSEKLIYAVYCTPWESMNISNQRSVCILLHRVQTPIQVTAMGMTPVGVQSMAAIIKTSLSYFAFLRSMDN

>EhipOR48

MRFLDSRSRPFKRVDRRKNILKMRNTSCLRLCLTIMTVTGVWLPTALKCSHLRYIYYIFAFMYHLLFVLPIIYMEFGVLYQVLGNIEQMANTSLLLVTHIAQCVKICVMWYKQDEIHSLLVTLDSSTFTREDSNKKQILKDVIQTTYTVSKVFISLVVITGIFWGIYPMLKPTLDLPIQYPNIPPEYKIFPFVYLYQIVNLTITAIAIASIDFLVGASMALVSAEMDILSYELSLVGKINKKDDKGNTDIKRDYVIIISCVEFHENIIKFVKDLEKIFGFPVFFQFFTSAIIICETAFRITNSTETIELLAMVLYFMCIITELLMYCYYGDLLKRKSERVVEMAYCCNWENTDVRTQRALLLLMQRAQRTLVLRAGNMFELSMMTFSAILKTSYTYFTVLNERRKA

>EhipOR47

MIFQRVISFANGLEDPKHPLLGPNLKALYVYGLWQSGSKFRIACANFIHFLAFIFVCTQLLELWIVKDDFMKVLHNLSVTILSIICLVKSSSYILWQSRWKELVNAISTEEISQMTQEDSVTQKLRKNYTTYARVVTYLYWYLVVMTNITMISSPLLKYVTTATYREEISNGTEPYPLIMSSWFPFDKTKMPWYWISVGVHILMNIHGGGIVAVYDSNAVVIMIFLKGQMRILREKCKTLFNDSENIQRKDILDRIKECHRHHGFLLTQSGLFNSVLSPVMFLYVLVCSIMICCSVVQFPSEQATTSQKLWVLEYTTALVSQLFLYCWHSNEVLAESNEIDRGVFESDWWKADVRIRKQVILLAGKMGQPFLLSAGPFTTLSVPTFISVIKGSYSFYTLFTQMHENK

>EhipOR45

MSESPSELAKREIDESLILCKFCMRYIGLSFEEPKSTRGYLTQKLMFLLSVCAIFYHVFSEIAYIGLTLSNSPRVEDVVPLFHTFGYGALSIAKVSVLWYKKEKFGELLQELAGIWPMPPLDEAAQTTKSKSLSALRMAHRWYFTTNMAGVWFYNLTPIGIYFYQSLQGQNTEVGYVWMSWYPFDKHEMIAHVAVYIFEVFAGQTCVWIMVGTDLLFSAMASHIGLLLRLLQRRLESLATTHQTDQEYYQEIVSNIRLHQRLIMYCNDLEDAFSLSNLINIVMSSVNICCVVFVIVLLEPFMAVSNKLFLGSALIQIGMLCWYADDIFHANLGVSMAAYNSAWYKTSPRCRRALLFLIKRSQKPIAFTAMKFTNISLVTYSAILTRSYSYFALLYTMYSEH

>EhipOR44

MKYVGCFRIHFTILGIAGIWMPRSFENNVRLKFYYSVYRIFFLTLFLVGIIYTQFRFFILVIGDIEKTVDSSVLFFTILPHIIKIYTLISCRERIIRLLDIIERIPDKNNILQMFSKNVALISSAYFCTCIGTAILWCVYPFMKPVLTLPFYYPYISQESFLFPVLYVYQTFGIIINALTIASEDFLAGGLMALAAAQLELLCCDLSTIGENKENINGNVDQYYEKIVTCIKFHVKIISFVEELSTIYGLSVFGQFLFSGILLCESAFIIITSDVFTESVTMFLYLLCLLGQLLLYCFCGNMIKTNSEKVAAAAYSSRWERTSLATQKALLFLIMRSQMTLTVTPGGIFDLSLVTFSAVLKSSYSFLAVLNQKHDS

>EhipOR43

MSAVTTCTLWAIMPLFDNTGTRSFPFKIWMPVKPQGSPEYEFGYVYQIVTIYISAFLFFSIDSVTLSMIMFGSAQLEIIMDKVKKLQVVPMSAKLKLVERDARIKSNNELFTECIRQHQAVIKFIQTVEDTYHANIFFQLSGSVAIICIIGLRISIEERNSVRFFSMVNYMVTMLSQLFLYCWCGNELTIRSQELREVMYQSPWYEQDIKFKRALWIAMERMKRPIIFKAGRYIPLSRPTFVSILRSSYSYFAVLNQTNKK

>EhipOR42

MSGGTTGHPRRYFALHFLLLRFLGLGWWHHPDEGDTSNFPGWYLYYSIITEVIWVAGFVGLESIDPFIGQKDIDRFMFSLSFVITHDLTLIKLYIFFFKNGDIQDIVRTLEIDLHQFYQNDKINRATIRITKILTGAFLFFGWITIGNTNVYGIIQDIRWKGEVATLNDSDLRPPRTLPQPIYIPWNYQSDVSYISTFVLETVGLLWTGHIVMTIDTFIGSVILHMSSQFSILQEAITTAYDRTMSQLCGNLRRDIREHNRDPLTTIDGSHDMDENENRERFVRARYSEKEIESALEETLKNCFRQHQVLINCVEKFAKTYSYGFMTQLLSSMAAICVVMVQVSQDASSFKSIRLVTSLAFFMAMIIQLAIQCFTGNELTLQAGLMSDAVMACNWERMPARLRRDLVLVMVRAQRPLHLTAAGFAYMDNRCFLAIMKAAYSYYAVLSQKQVR

>EhipOR41

MLKNFVSKLENPDHPSLGPTLWGLQAFGLWQPTKGVANIIYNLKHIFLALFIMRQYVELWIVRSDLDLVLTNLSKTVVTTFCVIKAGTFVFWQKRWRDVIEYVSTLERRQLSEKDDVTKTFIGDYIKYSRNITYMYWFLAIMTVFSLTVAPILVFFLSSKHHKHIKNETMPYPQIMDSWVPFDKSSGYGYWFTVLEATFVCYHGGGIVATYDSNALVIMSFFAGQLKLLKANCARLFGDGVETVTHKDAMKRIRDCHSHHLLLIKCSKILNSLLSPVMFLYVIICSLMICGSAIQLTTEGTTGMQQLYIAEFVVALIAQLFLYCWHSNDVLYVSETVQEDVYASNWWSTNVRTRRSLVLLGGQLRRKIVFEAGPFTELTTSTFITILKGAYSYYTLLSNKGN

>EhipOR40

MENTKKVQPFDAFSVSYKILILCGFFKLMRPTTRFRYICFQIYRPLSFLIVLVFIVQHSIYAVIKVMDNKLDKALDAFILIPPELNLLSKLLALNLHSSTVDKLNDVMRDSIFDARNQEDEIILTKFVSDMHQLTKNVEIGMGVAEILYILSPIFKRIYDPASTIASYYPFKLDNWGRYTIALLMDCSFLPWIGNGHVSLDCLIGIYYSQATIQLKLIKYNLEHLFDSDERNKDNSRNMEHQYIDVVDNTIQERFEHYVERYSKVKWYLQELHKVFSGAIIYQFASTIIIACPVIYKISFMDFFSVQCIYLTVYLIMLEIQIVLYCYYGGLVEYESISLNDSLYMSDWMSASPKFRRQMLIAMAQWSRPLTPRVAAIVPLSLNTVIAMLKFIYSLYTALISTNNIN

>EhipOR36

MKPKNIVLKPPEKKFNTFSETFRFCAFALAIALIYPNKNNIIRRWITVVSVIFFNSVILFWFVSYLIKCLISVDIYNFARAVTVGVVVLLFLFKSLYVNWKNEEFEQLLNKISKDLLKGNHMDEDYQRIYEYHIKQAKIAQICWLIIPTILSLQFPLYASTGLIYETLNSDVGKKYMVFEMQLKYIEDKQYVSPYYEIIFAYSLVPCLILVPNFAGFDSSFCIATTHMRLKLKLMTHKVHRAFKDARNRSDLQMRVKEAIKDHQEALGFHKDIQQVYGGWLLAVFLLTSFLISFNIYQIYLNKRIDPKYAIFTLNGVLHMYMPCYFASSLIKVNEELSTDLYNASWEDWADPAVTKLLVFMMAKSQQRLVITGKGIVIYNMDLFISILHTSYSFFTLITAK

>EhipOR33

MKRTSLAGSSVAPHLRVLRRCGFCRQASTASARLRYTHTYQLFVLTVTTVYLIQEIIYAYQARNNMEMLARVMFLLLCHITSLAKQIVFYVDADRIDHLIIQFDDPMYNPEEASRRRLLAATATSARRLQRVYSNTAILTCILWIVFPIMQRLTGRTVYFAFWTTFDYNSSTIVFVATLLYSFYVTTLVGVANTTMDAFMGTILYQCKTQLRILRMDLEILPERAYDLKKHTNEPYEKALMRLFVNSIEHYEKISETAQLLQDIFGGAILVQFGVGGWILCMTAYKIVDLSLLSIEFASMILFTICILTELFLYCFYGNEVTVESDRLMSSLYAMQWLCTPVAFRRALVLAMERAKRPLRPVAGLIIPLSLETFVTILKSSYTFYAVLRQTK

>EhipOR31

MVKTLTERLEDPERPFLGPHYWLTKKIGLFLPKSKLGIITSYVIHEIVTYFVVTQYIELYKLIVLKADVDLLLFNVRSSMLSVVCIVKSNTFLYWQSKWHDLFEYVTETDKFERETQDEVRASIINNYTKYCRRITNVYWSFVIFTNFTVIFTPLMKYMTLSDEHLKAIENGTEMFPHVFSAWTPFIDKEHSPGCWITILYHAIICSLGTLMVISYDMNVVVIMVFFGGKLLLFRERCKQLFDSDEAGLSDEEVRNRIRDLHLTYVRLVKYFNLFNSLLSPVMFLYVVMCSLVLCASIYQLTSSKDTMMTKFIMAQYFIFASSQLFLFCWHSNDVLAISEIIMYGPYESEWWAASVRQRKCVLLLIGQLRKDFIFTAGPFTDLTLSTFIAILKGAYSYYTLLRD

>EhipOR30

MLKRLLHKLEDPKRPLLGPNLKALKFWGLLLPENFIMRQIYILLHLSVILFTASECVDVWFVKSNITLLLNNLKITMLATVSVCKISTFLFWQNDWKKIIGYVIEADIVQRKTNDIIKQTIIKKFTKYCRKITYMYWCLMYTTVIIVMVQPIMKYFSSPVYRENVRNGNETYLQVVSSWVPFDKNTITGYLIASVIQSYGAIYGGGWITSYDTNAMVIMVFFRGELELLRRDCANMFGTESSPVTEQVSKERLKDCHRRYVALVKHARLFDSCLSPIMLLYMFVCSVMLCVTAYQITIETNPMQRFLSAEYLVFGVAQLFIYCWHSNDVLYASQDLMLGPYESTWWMHRISYRKNIFILIAQFRKRIVFTAGPFTELTVPTFINILKGAYSYYTLLSQSQS

>EhipOR27

MEDKAIFSTFETFRPLFDGLARVAYYKIVMKPVSRMKYWAHFSYRFIVWTLVVMYNLQHVIRVIKSRHSTEEVVNTLFVLLTTLNTLGKQIAFNSRSRRIDRLITVINGPYFAPTNSYHVRVLKENAVTMSRLLLLYHIAIFICGTLWTIFPLVNRATSENVEFTGYFPFDTTPSPVFELALAYMSFLITFQAYGNVTMDCTIVAFYAQGKIQLHMLRHDLEHLVDVKDGFKEIDSKLHEADNQYFTYVDIENVRFKQKIQERLVRCVKHYQQIVWFINEVESIFGEALVIQFAVMAWVICMTMYKIVGLSLMSAEFISMAMYLGCMLAQLFIYCYYGTQLKFESEFVNQSIFFGNWLALSPEFRRQLLLMMMRYSRAITPRIAYVIPMSLETYIQVLRSSYTLFTFLDRK

>EhipORCO

MMAKVKAQGLVSDLMPNIKLMQMAGHFLFNYHSDNSGMSTLLRKIYASVHAVFIVTQYFAMVANMAMYSDEVNELTANTITVLFFAHSIIKLIFFALNSKSFYRTLAIWNQSNSHPLFTESDARYHQLALTKMRRLLYFICGVTILSVFSWVTITFFGESVYMLVNKETNETLTEPAPRLPVKAWYPFNAMSGTMYIVAFVLQVYWLLIAMAIANLMDVMFCSWLIFACEQLQHLKAIMKPLMELSASLDTYRPNTAELFKVSSTEKSEKVPDPVDLDIRGIYSTQQDFGMTLRGAGGRLQTFGQNLNNPNGLTQKQEMLARSAIKYWVERHKHIVRLVASIGDTYGTDLLFHMLVSTITLTLLAYQATKINGLNVYAFSTLGYLGYTLGQVFHFCIFGNRLIEESSSVMEAAYSCQWYDGSEEAKTFVQIVCQQCQKAMSISGAKFFTVSLDLFASVLGAVVTYFMVLVQLK

>EhipOR25

MGLVDSLWRKLTQTKALEESSGKLETLFFESVYRVTYLAGLSSSDTHPVYRAYSIVVKLSIILFVSSELWYLVSETSSMDKIIDNINVTLIHFIAIYRYKKLMDHKDKYKELAKSMESPHFDISTPKRKKLVQFWVIRNERYLKLLLGLGTCTLAAWYVYPLVDDLEYNLSVAVRLPIEYRTPSRYPLAYIVVLITFHYISYFVIVNDLVMQAHLMHLLCQFAVLADCFENIINDCDVETQGISQNKLFLSEKFKDKYLCRLNDLVDQHKFILHHATTLKKILSTPMLGQLAASSMLICFAGYQVATTVTINLTKFVMSLLYLGYSMFELFIFCRWCDEIKIQSENIRLAVYCSGWERGIAAVPGIKTRLMLIVARANKPMILTAGGLYDLSLNSYTTVVKTSYSALTVLLRLRQE

>EhipOR23

MKILVDNANLSISLSLTALKLVGFWAPDGLNGINKFLYNCYALFSFMFLLGTYLIIQVVDLYFVWGNLPLMTGTAFLLFTNLAQAAKIINILSRKRLIQKIITEADLVLKGQQTEEGRIIVKNCNRETSLQQLLYFCLTTITVSGWAGSAEKNQLPLRAWYPYDTSKSPAYELTYMHQVGALFIAAYLNVGKDTLVTALIAQCRCRLRLLGLALRTLCRDVEVTHNHLLTANEEQVVSSRLRNCVCQHQAALAAASELQTCFSAPTFAQFAVSLVIICVTAFQLASVSHTGNLVRLFSMGTYLLNMSFQVFLYCYQGNQLSEESTEIAGAAYFAPWYAFSARLRRAMLVLMTRSRRVARLTAGGFTTLSLTSFMAIIKASYSFFTVLQQVEEKA

>EhipOR22

MKILTEHVKKKLSILLPILPYGVLESWENLDPRLYHAVHIYWLKFYGMWYNNFSPKSTLFWLQLIYTMIVLWLVCFLPGIGEVVYLLRRRDNIGDIAEGLYLFLSEMYTYFKIGVFWLNKDKIVSLLQYLYCEEFKPKETEHKEILRKSIKSARFVMTYYSTICVGAVSVGCIMPISENFQVLPTNVEYPFFDVYKSPVYETMYIHHIYYKPATCIIDGVMDTILAAFVASAIGQLEILAYNLRNFDVIAERRRIRDNNRMNTKVYSKENYMKTVLKDCIKHHNSIIRYVSMIESAFSLASALQFMLSVMVLCLVGIQFLSIENPSSHPMQIVWMAIYLTCMLIEVFILCWFGDVLIWKSMELRQAAFEGPWLNVNPKTAMFIIIFLERCKRPLRVTAGKIFTLSLDTYTDLINWAYKAFAVMRNMKK

>EhipOR21

MEEKLAYYSLVPHFKQLRQTGFYPLDPEAPKLQQFLHKLYNRFSVSLLLLYTMQQIINLYQWRRNINKIMDSMFLFLTYSDCLIKEAAFWNKADEIEGLMNIMKGPVYNQKEHKQILIRTVRQAKLVLRIFNIMCLITCLLWAIFPVIFHLRGKAVEFGIWLPFDSNANPQFYFVVIYVSFQTSWLAYNNSTMDVTMAFFLAQCTTQFFILRQNLENIVKKSIDDSTTLRISFAKAFEKRFIGVMQHYNEIVQSSQKIEEIFGNAVFVQFIFTGWIICTTAYRMINMSPGSIEFLSMILYILCVMTQPYLYCFYGNELMYESDKLMNSAYAMDWLMIAPKQRRYLIIFMERIKRPIQPKAGFVIPLSANTFVTILRTSYTFYAFLKNSY

>EhipOR20

MEIPTFEESFKLIKRNFWLSGIPLENTKTTIRFFLLYISLLLMIIEETAFLVSKISTEDLLKLTQLAPCTCIGVLSFLKFSAICFKRQKIRQLADMLEQLHADISSDVNKKTLVKRNIIFVNKLIKYYTILNLILINVYNFSTLVFIGIHYANTKEMLYRLPYALIVPFSTDMWPTWLLIYIHSITNGFICVLYFTTIDSLYCIMTSYIFCNFIIINNELRYLETINPKTLTNIVKKHQYTIKLSEDLEDIFTACNLFNVLIGSLEICALGFNLTTGDWAQIPGCVLFLLSVLLQILMMSFFGENILRESTNVGESAFFSKWYEADERSKKIILYIMTRSHAPQKLTAYKFSVIGYGSFIKIISTSWSYFTILKTVYTPPAEVQIE

>EhipPR2

MSKTETVMDLIYIKQLRCCLNAVGCWPRKETGQKPIKYLSVYSVILVLFAIFMIIDGFWYIKNNIQNMDIFEIGHTYMTTFMSCSAGYRLTLPFQKKYKRMTETFIKQFHLLHFKDKSDYSMKIYKKIDKLSRYITMYFNFLPWLAVLGFQLQPLYYNYVNGLYSSNRPENTTFKHTVYYVLPFDYETNVYGYICIYLFNLYASCVSAVCFAYVDLYMLLLIFNILGHLKILLHNLQQFPKPQESDSVVDTIMFSNEEMENIFELLKESINHHRIIMDFVSLTSEALSALLCIYYGFYQIILFMVLLLCSQLDINAIVNYSIVALFFFQELILTSMIFELLGTTSDKVKAAVYELPWECMDTKNRKIVLFFLKKAQEPIELKALGILPVGVNTMAAIIKNSISYFLMLRATV

>EhipOR15

MSVAEEFDKASEIIKIFFKLMGIHLNENKTIIDHFKSYWFYYFNFIWLNIDVLGEILFVITGAINGERFIDLTYMLPCIAECLLGDFKTYHLIKYSHHVRDLTHTLRNMNYHEMFTNQEVEKKIFQESFPFMDLGVNTLKRCNIVALINFGMNPMFVMASKYYTTRKFELYLPFHIWYPFDAYNHYLYPFVYVHQVYSAYVALFTVYGPDSLFYTYSTFVGVQFRLLKHSIEHIVPPTFTSTEQELADFHIKIEKIVLWHLELVRCVKLLENIFTKSTLFNAITSSFLICLTGFNITAIDHLPFVMTFVAFLSVTFLQIFFFCSYGDMIMRLSVEVGDSVYNCQWYLVSPSTAKQLVIILTRAQMPCKMTALGFADINLKAFTRILSTSWSYFTLLKTMYSSPGAENE

>EhipOR14

MPHLKSSVEEEYIKTPPKEQLFYQFLGKMMSLWSLGNQSWWGYKPHGLYVKINSAFVPVIGPPCLISQFVYLYQNFSKLSMSTLGVIFTMIPMTFLVNVKVRVHKTKKYKKLMKVFLSEIHLYNFIEDDNNVKQIVIRIERYTRWMAYCVVMLVTVNWLSWAVIPIVNNLKFKDDVQNKTMQLQTSLYMWMPFDYEHDYQNWIIIHSFNIYLIAIGCALLSIPDVINYVFLFHLVGHVTLLNHRIVSRLSFELTDKEVEERLKDVVEYHTFINKLFKDVESVFGVNISINYLNNLIVDSLLLFQSINQEKGDTTMFIYGVAFVICMGGLILMSFILEEIRNQSDVLPDSLYGVPWENWSIPNKKSFLTILTRLQPELSFVAAGGLRTGVTPMISIIKSTFSYYVMLKSTI

>EhipOR13

MFAPRKERSIFLNEIHLLSFYLSKLFLFPFFGKTKFKQFGYYSTYFLIVFTSFQLCFTLLLSGLNDFIEIINISPNLGVCVMCAIKYAKVNSNRALYYDIFEHFREDLWKTISEYSAEDLKTITKYTKIIKLINRTLILYISLPLIAIVNICPWILMVYENKVGKEQKLLLPFDGWYPFDKVNWYFVAYIWESLMTGLIIFVYAITDALNISFVACICMELKLLGNSLENLINPEDIENITRLKNISRTHENIKRKLNVTIKRHTFLAKISSELNITLGDLMLVNYTFGSLFICLTAFTFTVVDDLYKSLRYFFFFVALIVAILDQCIMGQSLSDNSEQLAEAIHASNWLYADQQTKRTLLMLLMRTQKPFQLTANGYLVMNLDTFTRICSSSYQFFNLLRTIYQP

>EhipOR12

MKNHNILKNLCNVVFVAGSGNFWLKENHIGDDNSFLYRFYRFVLFSIYGFMTILEILAAVIGDFPDDEQSDAVTFAVSHTIVMIKIFSVIYRKELVKTLNRNMVRVCEIYETKTLMEEQYRIMKINVIAYFVTVYGSAAFFIIAGARKMREGSHFITIVTYWPGHDDDTGIATVFRIFTTVVLCVMMVTMVSIDSFAMVYLIMYKYKFITLRHYFEELRKEFDEASKSSLELASDKLTQGLVDGIEMHKKLLRLSRDIDRSFGTVMALQVCLSSGSAVSLLLHLALSKELTFVVTMKILFFGVALFFLLALFVCNAGEITYQASLLADSIFYCGWYACPSQSAPRRNIRQLVLLAVAQAQRPIVMKAFNMLELTYGTFISVVRGTYSVFTLIYAQNT

>EhipOR11

MMSLLSLGEQTWWGYKPYGTIVYINSSLVPIFGPLSLTCQIIYLYQNFRNLPLNVLGTIFSMLPMTALVNIKIRVHKTKKYENLMKSFITNIHLHNYKEEDSVVKATIIKIERYSRSMAYCVIFLVNFSWMLWSVIPFVNNLNNKEAIHNKTMLMQSCLYMWMPIEYEYDFKKWLITHFINIYLMGVGCALLAIPDIINYTMAMHLIGHVILLKHKIVSSFPTELNDKEVKQKLKDLVEYHCFILKLFKDVESVFGINISTNYFINLLIDSLLLYQLMNQEKGDKTSLLFGITIVICMGGLIIMSFILEEIRKQSDDLPESLYGISWENWNVSNQKSLLIILAQLQPELAFVGAGGLRTGVTPMVAIIKSTISYYLMLKSTI

>EhipOR9

MIGKHFKRSFQTTNLFFKLMGIRFRCNKNFFDHIQCYWLFYFNILWLNRDLLGEILFVVFGVMSGESFVDLTYMLPCIAMCLLGNMKTCLLIIYSKEVKDLIDTLKNMSFHDDDMSDERSSYVDDSNDYVYDRVDYVDGTCAANVDETRDCVDEIFKKTLPFMYSAIKVQKICNVLVVTNFGLNPMFLMVLNYHSTGQFGLFMPFHIWYPFNAFNYWIFPFVYIHQVYSAYLAAFTVYGPDTLFYVCSTFIAIQFRLLQNNLEAIIPLNYTSKIEEVNEFNMTIKRIVKWHLELIRCVQLLEKIFSKSTLFNAITSSFLICLTGFNVTAIDNVPFMLSFISFLLVTFLQIYFFCCFGDMIMQSSVEVAEAVYNSRWYMVETSLARELMIILIKAQIPCKITAFGFADINLRAFMRILSTSWSYFTLLKTMYST

>EhipOR7

MQTQTCLHVWAPFDYRYDFHKWLIVHIIINVYVVAHGCGILGIFDVVFYIIVFHLIGHIKVLKYKIKTQFEGDLDDEEVKKRLVNVIKYHAFIIKFFKDVEAAFGINVSGNYLNNLIADSLMLYNLMIIAQDKGTVIIFVVMTTVCITELILMSFILEEVRIQSDDLPELIYFMPWENWSLNNKKMLVLILLRIQPELAFVAAGGLRAGVRPMTSIIKSTFSYYVMLKSSMRE

>EhipOR6

MDKKENIRIFTGNLLNFLEIPNHPCIGPHLKLLGLTGLWHPNKNSPVTKFKRILFYVTVTFFCSQYIKCLFNIDVISLALILQYAPFHMGIVKSCYFQRHYKKWERLIIYMSSVECAQLADRRTDLIPIMNGYIKKSRRITYFFWALAFFSNFAIFSEPYQKNHVMENSTAVYTKIFDGIAPFNQEKPPGYYVSMVLQTIFGHIVSAYVVAWDTLVVSIMVFFSGQLKVSRLYFVQIIDVNSIEKSHQNIVKCHYFYTTLVRYQKMFNSLISPVMFIYLIIISVNLGVCIIEIAQLQDDIVTLVSSCLFVLACVIQLLIFYWYANEVTEVNNLVGYGIFESEWMQLDKSLQKEIALLGLITTKKIVFKAGPLNEMSLSTFVGILRTSYSFYTLLSKTKN

>EhipOR5

MSKSTKSLLRAFCKYVYYAGAGNCWYEDTYRETYLYKAYALISFSIYTTMIFLENLAAWFGSFPEVEKNSAVMFAAIHNIVLPKMFLLLYHKKSIRKLNYEMATVGEKIEEKYVMERQARKAKIGIILYVISVYLSLGAYGVESTRKVIVEGAPFYTVVTYLPQYDDSTIVASIFRVVFYITWLYMMLPMMSADCMPITHLITMTYKFITLRHHYRRIREEFDKDLLTMDKRRAAEKLRAGCLEGILMHQKLMFLADEINRIFGIIMSLQVCESSAVAVLLLLRLALSPHLDLTNAFMTYTFVGSLFLLLALNLWNAGEITYQASLLSHAMFYCGWNLCDMDKQSHHDIRRLVLIGCAQAQKPLILKAFGIQDLSYETFVSVARMTYSIFAVFYQRGEQN

>EhipOR4

MIDVENLYLNRAKFVMKILGVWIPSESELMISKMYRIFMMSLQYLFLLFQTIYIVQIWGDLEAVSQASYLFFTEACLCFKVTIFHINMDMLKELLKEMNSDVFKPLSLKHEKILKLQAKRIKRLLLGFMISSQVTCGLWALKPLFDNAGTRAFPFDMWMPMSPKFSPQYEIGYAYQFLTVCMSAYMYFGVDSVALSMVIFGCAQIDIVKDKILSIKPVSNARGDERKKILSENNKKLIECVVQHQAIVTFTDLVENAYHSYLLFQLSGSVGLICMSALLILIVDWYSIQFLSIVTYLSVMISQLFVCCWCGHELTASSEDLHAVLFQSMWYEQDVKFKRALCFVMIRTSRPMVLRAGHYISLSRQTFVSILRMSYSYFAVLNQTQST

>EhipOR3

MDDMELKKTHPQKYYLKFICNSLYVLGYGSCWYEETPRTNFHKIFYKIWAGIANFFVVIIVINEIMANFRPNLTAKEQNDLVQFTFGHSLIIAKIVTMYYQRDRIKAVLKKLLEENRTIFISADIDKSSVKKVKIYCIVLVTTVYLTMLSAYIDGFRVHFNEGIPIRGEISYYPTPLDSGILVNILRFIMEFHWLYIVTVMNLIDCMSYCTLIFLSSQFKLTQTYYNLLRKKYTKNSNKNTCSVLGEEYKKDFLIGIRLHENALWCAHHVQASLGYMYSSQICQSIILIVMCLVKFVTSARNMTVLLANMTYLSAMTVMTGAYMTAGGDITYEASLVSTSMFLSGWDLVVFDKELRTLAVVAIQRSQAPVYMTAFGVIILSYDNLIMVLRSSYSFFAVMY

>EhipOR2

MSPIKQSESFKFNFVLLKFFGIWPGCTTNKYYKYYSIVYFVTMLLIYNILLTVNLFYTPQKVELLIREVIFIFTEVVVTTKVLMILLMREKLVVLFDMLDDDIFNGEDAIGRDIVLKYNSYYKSYWKMFTVLSLFAYFSQVFLPIIIYFIFNVSNPELPICKYYFLSNQVREKYFWIFLLYQSFGMFGHLIYNVNIDTLMSGLILMAVAQLKLLNHNLRNVKFQKNKSKLKVQIQENIQMTRLNQCLKHYDVLLKYFYKVQEFISVTMFIQFGVASAIICVVMCGLLLSSTTETMMFMVSYLFAMTLEIFVPTWLGTQLSYESQELVFAAYNSEWIPRSESFKRSLRLFMEHANAPLTLTGLKLFPLCLGTFISIMKTAYSFFTLVRNVQDSQGEAI

>EhipOR1

MFYFTEIVVTTKVFMVLGFRSKLLDIMNHLDCEEFKATDENSRKIIDKHVYYYKTYWKIFSTLSHLSYFFLVLLPIIIAKLMGTNLELPICKYYFFDDKLRNRYFYYLFIYQSIGMYAQMTYNVNADTLMSGLILMAVTQLKVLNYKLSNLKVTAEHSKLSLEIQDNIQYQKLNDWLKHHYLITTFCTKIQNLINVTMLIQFGMSAATICVSLCGFLMMSSTGTLMFVSSYLFVMIVEIFVPAWMGTQLSYESRESVFAIYDSEWIPRSEKFKRNMRLFVECTNVPIILRGVKMFPLSLETFTSIMKTAYSFFTLIRNVQDRENGRIS

>EhipPR1

MFFFKENDMNTIKRPQDLRYMKQLQFSLNIVSAWPHKETGDAGSKFVFWWRLYYAFVEGFFWFLGAAYLKNNYGKISFFEFGHTLITHFMNTIACQRLTLPFMKKYRDFIGLFVKQFHLFHYKDKSDYAMKIYLRVYKLSDFFSMYLHILMYIGIVLFNGTPIYKNILSNAYSSNKPENVTFQHSTYFELPIDYKHSLTGYVPLFCFNWYITFICASFFCMFDLLLTVIVLNVYGHLKILVYHLEHFMTPSTNSTSHKQKNVFDIMQFSEEEMKTVTIKLKEVISHQRLITDFIQKMSDIFGPMVCLNLMYQQVSACILLLECSQMDLLALLSYGPLTFFVFQELIQLSVVFELIGATSDDLIDAVYSVPWECMDTKNRKILYTIMIKSQMTTKFKAMGMVDVGVKTMAAILKTIISYFVMLRTVALQN

>CsasOrco

MMNKVKAQGLVSDLMPNIKLMQAAGHFLFNYHSDNAGMSVLLRKIYASAHAFFITLHFLCLVANMAKYSDEVNELTANTITVLFFAHTVIKQLFFAVNAKSFYRTLAVWNQSNSHPLFTESDARYHQLALSKMRRLLYFVLTVTGMTVVSWVTITFFGESVRMITDKETNETLTEVVPRLPLKAWYPFDAMSGTMYIIAFVFQIYWLLFSMGLANLMDVMFCSWLIFACEQLQHLKAIMKPLMELSAALDTYRPNTAELFKASSTEKSEKVPEPVDLDIRGIYSTQQDFGMTLRGAGGRLQTFGQQDMNPNGLTQKQEMLARSAIKYWVERHKHVVRLVTSIGDTYGTALLFHMLVSTITLTLLAYQATKIDGMNVYAFSTVGYLSYTLGQVFHFCIFGNRLIEESSSVMEAAYSCQWYDGSEEAKTFVQIVCQQCQKAMSISGAKFFTVSLDLFASVLGAVVTYFMVLVQLK

>CsasOR3

MLTNEEILNMKYVKIVRLMLIPIAGWPGEQFGENMLTRKKYLKYYLVVYNTIMTVGVFNYISKFLMTQSFFTAGHSVICASLGILTHIKLCSFHLENYGRTIKTFFTDFHLVNFKDKSKLHMTVYKQIEKISYYTSVFVAISTVMAMISFNMTPMYNNIKNGVFRKERPPNVTIDYIVSYLFFNYNTDDHFYTSTFLICYFNTVMVSLSVAIPDLLFILIVFQIIGHIKVLDKTFQNIPQPKTTRIRCQYKNGNIDVDYAVYGTEENKNIQNFLKECIFHHRTIMSFTNDAASLFGPILGSNYMYQLCNCCILLLELSQGGTDALTQYGTLTFVICYQLIVVSILYEEVHTASSRLKDAVYGMPWESMDMKNQRTVYIILYMIQTPIQVTAFHMVTVGVVTMSKILKSTFSYYAFLRTKKH

>CsasOR5

MNRKFKLSGGKKKVEDLNLQYDISTYDKTYEMPLHVFKYVAIRLTRKDGPIAKILYDAFYWFEFLNLITASTVEFLSMLKTATGGSFQDAVAIFLMLPCIGYVILGKVKSFNIVYHRDVYENIVWELRDMWPTRTLTREEHNIISAALKQHRTVLRIYYWCNNVLLLTFYVPPMIGSLRKYAGIDIQYHLPFLYWLPYDAFQPGFYEVTCIFQMWHGFLTVWLMMTGDLMFCTFITHITMQFDLLSIRIHKFIYVPIDQQLPSSFPLAVYSDEIRKDFETSNKALEKELREIMDRQRALIRLSADLEEMCSFALFVNFFNSSIILCFCGFCAVIVEKWNEVIYKSFLVTALLQTWMICWYGQRLLESSTDVSHALYKSGWYRASSKIKRSILIMIHRAQKEVHVTTYGFSIVSLSSYSAIIRTSWSYFTLLLNVYKK

>CsasOR7

MAVMKLQLPFLVNYIVFDPFKASVWPFVYVHQVWSTCIACANVYGTDSLLYALCAYIHINFCIISHRFEHMMSWSYNTRDEVRRRLIENVRRHQDLITIVGQVQTLYSESTLFNFVISSFLICLSAFNITFLDDIGAVVAFLTFLTMNISQISLMCFFGDMLMRSSSKVLDAVSRCPWYDADEETKKSILMVIIRQSTEAMQADSCQLWRSHVDIVHNGDESFLVILRSPEDHDGK

>CsasOR8

MANSLQSDIILNLRYIRIIRFFLLLIGAWPGERFGENIHSRKNYFRFVLISYKIAMSFGIFNYLHTYLKTETLITAGHIIIILSLSIVILIRVIFVHLNGYGTIVEKFITDFHLLNFKTVSKNHTTECLRVEKACNYLTKVMATVTLSALCVFNMSPMIYNWRNGVFKKNRPDNITIDYVVNYEWFGFNSDDHFYISTFFLGYFNSYLVSTTVVMLDLFMTIIVLHIIGHINILRNSLKIPPPQTVRVLHINDISSVNYAVFDADENKSIQQMLIKCVNHHRIIKRFATESSSGFGPILALNYLYQVLNICVLLFECSHLSSTNIGKYGPLTFMIVAQLVSISVLFEFVKTASSKLMEDVYAIPWESMDVKNQRTVYMLQFIVQTPLQVTALKMVPVGVTTMQQIIKSSLSYYAFLRTKSTF

>CsasOR10

MSKIPLAYLSLLPHLKNLRYGGVFLIGSENPKYLRALHTIFRRVVMALALLYDLQQVLKVYHERSDTNKAMATMFLLLTHMNSFYKQFIFTKREDKIVVLLDMLKEPIFNQDTPRHIEIQKDHIRRAHIVVKGFYRVAFCTTVIWALYPIVMYQRGTYIEFSIWLPYDASLTRNFYITMMYTWIQTSWLAVLNTTMDSFMAHIMAQAKVQFTILRYDIENVVNNSLEEAEATSTPFNDIFERRFRHILKHYDEILRLCKKFQDIFDGNLLVQFLSSGWIICTGAYRTLDLKPGSVDFMSMLMYLWCMLVEIFLLCYYGNEVNLESENLVQSTFSMNWMDLPMKQQRRVVIFMERIKRPFQPLAGSFIPLSNATFISVLRSSYSFYTFLKRT

>CsasOR12

MSSNVIPTDFKSTMAYAQKLFDISGLPLVDANKLISRNVRFVSISCILLPIIIIQFLYFFMRLNSDTNFLHFANNVACFSMYFQDIIKILVLFTQRKKIRLLVSEIKEMWPSDFDDQLKRCIFLRSIRKMMIFNKIYYIYAITGYSAFIMAPLIFILYKNITLDPIHEYLLPLDLSYPFEIDSISIYLMVYLQQLGTSAMLYCIYIACDIFIVSLAGQLSTLLRLLQLDLGSCVDVNKEDGDYGRIRTAVQNHQKLLRLVDSLNDIFGVLFLLQVTIASISICFFGFQSVYLTGGEALQSYTAAVGMMYNIYFTSWPGQILYDTSSGVAEAAYNSRWYERDVKYRKYMIIIICRAQKPCRLSALGYTDMTLDTFSTILKTSWSYLSLLKSIYER

>CsasOR14

MKYIQNEFKVMKICLNYMIAIGSWEPYPIEGFSNVLFFCYGIFSYLLQTGFFTIGQVIDLCLIWGDVNLMTSTMFLLLTNIALSAKYFNYLLKKTAIRKVINESNDSIKDEVSIEGREFIQRNNFQSICIWYLYIALSYGSIAGWGLTAEKNSLPLRAWFPYDTKKSPAYQITYAYQLIALFFSAFSNMSMDMVMINLMTQCRTRLQLLSLSLKTLGDDLQLTYNKKATPAQEAIMESRLRKCVRQHQSVLDTVTQLRDFIQVPTGIQFAVSMIIICVTAYQLAFESKNITRTVVMGVYLVDMMLQVFLYCNEGNELLGESSEVATAAYHCPWYTCSAGFRKKMVILMVRSTRVTKIIAGGFFALSLPTFMAIIKASYTFFTMLQQVVEDKM

>CsasOR15

MSTVEARPRRYFRIQFLLFRYLGLGWWHHPDEGNTSNFPGLYIYYTIITELLWVAGFVGLETIDPFIGEKDVDRFMFSLSFVVTHDLTIIKLYIFFFKNKEIQEIVHTLEIGLYNFYQNNKRNRATFKLSRIMTGSFIFFGWITIMNTNIYGIVQDYRWKKEIATLNKSSPMPARTLPQPIYIPWPYQSEGSYVFTFALETIGILWTGHIVMGIDTFIGSIILHMGNQFVNLQEAIRTGYDRAMLQLYERIDTSDRIDDTKTIERIVRAHYPENDIDACVEDVVRKCILQYQVLLDCIQKFNDTYTYGFMTQLLSSVAAICSVMVQLSQDASSFKSIRIVTSLAFFFAMIIQLAIQCFTGNELTYQAAQVSDVLMQCKWERMPVRVRRLLMLTMLRAQRPVRLTAAGFANMDNACFLSIMKAAYSYYAVLSQRQA

>CsasOR16

MYGHMMYNVNIDTFIAGLIIIAVTQIKVLNYKFNHLKSPEIENQATDLEYNYMTKLNRYLRNYDIILKYYAMIQDVISVTMFVQFGIASLVICVILCGLIQPSSTETIVFLTLYLFTMTFQLFVPSWLGTQLDFESKELMFAAYNSDWIPRSAAFKRSIWLFIERAKTPIVLTGLKMFPLSLVSFTSIIKSAYSFFTLVRNVQEGQNM

>CsasOR19

MAKVTSEDLFLNRARFIMTCLGVWVPSTTDTILRKSYRHFMISLQYLFLIFQVIYIGQVWGDLAAVSQASYVLFTQACLCFKVTVFHVNMDMLIEILKQMNSDIFKPQSIKHERTLAWQAARIKRLLLAFMVGSQVTCALWALKPLFDDAGNRQFPFDMWMPVKPNRSPHYYFGYAFQLVTISLSADMYFGVDSVALSSVIFGCAQIEIVKDKILKVKSLQANNKTTAEQSKIQEQNYKILIECIKQHQALITFTKLIEDAYHTYMLFQLSGSVGLICMSALRILVIDWFSMQFMSIALYLTVMISQLFLCCWCGHELTASSEELHTVLYECIWYEQDVKFKRTLCFVMMRLGKPIVIRAGHYITLSRQTFVAILRMSYSYFAVLNQTTQRD

>CsasOR20

MFTTVVIVTVQPIGKYFSSSTYRENVKNGTETYLQVVSSWVPFDKNTIPGYLAASLIQIYAAVYGGGWITSFDTNSMVIMVFLRVELELLRRDCAKVFGSELNPVSNDVAMKRLKECHRRHVELVKHAKIFDACLSPIMLLYMLVCSIMLCVTAYQITIEKNPMQRFLMAEYLVFGVAQLFMYCWHSNDVMYMSKDLTLGLYESTWWTRNVMIRKDLHILTGQFKKTIVFSAGPFANLTVPTFISILKGAYSYYTLLNQSQIEKES

>CsasOR21

MAKEARTIVSDNEENRTYIDPLSFKYIKIIRTIYNPIGLFPVEKFGLTSRWRIQRFIHPFLAAIFWIFEINFVVQNFHELDFFLLGHCYLANVTTGMVAIRTYIYTREKYDNLMYEFLAKFHIVHARFNSPYYEKMFRLEQKISANLTNGIMISTFLGSFLYSSLPIYNNIRNGVFGREKGSNVTVDLSIYFLAPNFDPRDHVISATIFNIYASLCCSFMTMISDLVVYLMVIQIIAHIYGLINDLENIPKPKRFNDRRTFRHEDMIIPLVDMYDAEENAEVLSILGRCIEHHKMIVTFTHEISSFYGPMLAYNYFYQVTSLCVQLIEISRGDMNTFFRYIFFACLVFSELILMSVIFEAVNHTSEKVIQSVYDIPWEYMNTSNRRIVSVLLHRVQSPIKVTAIGMVPVSLGTMTAILKTTFSYFAFLRTLG

>CsasOR22

MGKQFAFNIKSSKVDHIVSVVDGPIFAPQNQYHVDVMKRNAREMSVLLTVSHVGVLSCGILWTIFPAVNKLLGDDVQFTAYFPFDTSQSMIFSIIQIYMSILIAVQAYGNVTMDCTIVAFYAQVRTQLQMLRFNLEHLADFDETKIDTIRTQSNGSIVYKDVVNRERMQTRLVRCVKHYQLIVWFCNEIEIIFGEAMVVMVLVTAWVICMTMYKIVKLSILSVEFLSMVVYLLCMLSQFFIYCFYGTQVIVESEHINESLYSCDWLALTPRFRKQLLIMMQYCTRPLAPRTAYIIPMSLETYISVLRFSYTLFTFLNRK

>CsasOR23

MKTKLLDYFENPNYPLLGPNIWSLDKVWLFLPKTLINKILCLSLHGSAMLFVLSQYIELYVIKTDLDEVLNNLKISMLSTICVLKAGTFLMFNDRWRKIIDYITEADKYERDNRDKIKGQIIDGYTKYCRRISFSYWFLVFCTFVTVTQTPTLKYGTSAALRYNFRNGTQAFQHIFSSYMPFDKNSFPGLPATILWHFCICVYGAWVVAAFDANVVVMMVFFGGQLEIVRERCMHMLDDYDYTDTTEVQAYASICKLHEMHVELIKHSKLFNSVLSPVMGIYMMMCSLMICASLYQFTGDISAAQKIIVAEYVVFGVAQLFVFCWHSNDVLVRVSS

>CsasOR24

MKKNDLVFEDIFKITTIAMHVTRSHPDIKRNIVWIVQVIPYMSLSLWAFINLTRSVIYTDIPNKDYENACKTGIMGIMSVTITYKYYNLVLCQKSIVGLISIMNQDYEISKNLPIEDQIIVRKYCAYGVNVSKFWLVCAFVASSVFPVKAFTLMAYYYWRGEPRFVSMFEFTYFSCIEYYKYLTWVNWLLFGICFAFGLLAMSMYIAFDPVAPIFILHACGQIELLVKKLNNVFLKKTDIKIVETEFKSIVVKLQQIYGFINEVDQNFTVFYEYNMKATTIMIPLSAFQIIKSYHQNEINFEFISFFFGCILQFFVPCYYSDLLMDKGEKLRQAIYSCGWEEQRSRKLRKLVLLILTRASRPLILRSIFNTICLDTFAEMCRQSYTIFNLMNALE

>CsasOR26

MLFLGAFWRWLTNTAALNQLNGDYERMFFEEVYRVVYLTGFSSYDKGIVYLLYSSTVKLLIALFICSEIWYLYSELSSLDTIVENISVTLINLLSVYRYIDLIRNKDIFTKLATAMESRYFDVSTQTRKELVNFWVLRSLSYLRLILYLGTCTLAAWYVYPLVDDLEYNLCVAVRLPFDYRTPLRYTIVYIVVVVAFNYAAFFVMLNDIIMQSYLMHLLCQFTVLADCFENIVTDCETECGGKINRSHLQSNKEFTDTYLKRLRNLSEQHKIILNNTMRLRAVLSTPMLGQLGASSVLICFAGYQVSTTVSISITKCMMSLFYLCYNMFELFIFCRWCDEIKTQSENICQSIYFSGWELGLVTVPGVKRRLMLIVERAQRPMVLTAGGLYELSLVSYTAIVKVSYSALTVLLRLR

>CsasOR28

MMAAIDCLAFCALVFVEYKFRVLQLYFGELKAKYVINVGKKSSAQLIFEFKKCLIVGMKFHENALWCASSVQYSLGNLYSVQIFQSIALVVLCLIKLVVTDHYLIYIVANMVYLACVLILTAIYMKAGGDITYEASMISDSIFYSGWEVMAADRELRCLCVVAIQRSNVPVIMSAFRVIPLSYSNFISVLRYSYSFFAVMY

>CsasOR29

MKVTEYIREKLSIIAPVLPYGVLESWDDLNPRLYHAVHIYWLKFYGMWYNSFEPTQLLFWVQMTYMLVVFWLVCFLPGIGEAVYLLRRKDNIADVADGLYLFLSEMYTYFKIAVFWLNKDKIIGLLDYLHCREFKPQEQEHRVIIRRSIQTARSVMTYYSTMCVGAVSVGIVMPLTEGLNVLPTNVEYPYVDVLSSPTYEALYIHHIYYKPATCIIDGVMDTILAAFVASAIEQIDILAHTLRNFEEIADRRRARAQCDYSKEYYVKTVLKDSIKHHNSIIQYVAMIERAFSLASALQFMLSVMVLCLIGIQFLSIENPSSHPMQIVWMAIYLTCMLIEVFILCWFGDELIWKSMDLQQAAFEGPWRSVDPKTAKLIIVFLERCKRPMKVTAGKIFTLSLDTYTILINWAYKAFAVMSNMKK

>CsasOR30

MPENELEIAQIQIFETLKWSEKCTRQIGIEVTDVSTNTFCGKTGKKCLILLGQTMLFLLVLVQLCHIIEGMVNGDLVYAVLGIHITGYGMLGLGKWVTLQKKQHVIKELVYQLAEIWPVHLEDPEAVEIKRSTLLKLRIVQACYIMFNISGILAYTVFPFIQFLVMMYILGKPADLGYAFFATYPFDKTKPVYHEISFVFESVGGILSVFSMLGSDVMFISMASHISMLLRLLQIRIGRITSAVESGDGVHELFDCSQEAINVIKVHQRLIKYSEDLEDAFTVSNFINILMSSVNICCVTFNIVLIEPVTEYSNKLFLAATLNQVFLICWYADEMYRSSLGVSDAVYKCDWFKCNNRCRRIFLLMIVRSQKALCFTALKFSSITIATFTSILTTSYSYFTLLYTMYSE

>CsasOR32

MNSARVEKINNLMRGHDTLFAARTKKDEEIIMKNNKAMHRLVKAFMYTICMSNGSWIASHFMSRLRDDAAIVPVYMPFKVETWTQFTWSVMIESVFPLLWIGYGHLTLDMTIATYYANSRTQLKIIKYNLEHLFDRNVPLNMSASHSYVDEVDTTVRERFIHYVQRYDMVVWYTRQVDRVFKGAMIFQILSSCVVYCITVIKISQSDARKDSAALIFLLLFFVVMLSQVFVYCYYGGLVEYESKLLNDSLYLSDWTSASPAFRRMLLTAMCRWLRPLTPRAAHSIPLSLTTFFMIMRSAYSLYTVLVSTAQKG

>CsasOR33

MEPETQIKEVDVEIAEDTKVNPLSFKYIKVVRLFYDPLGVFPVERFGEKSRLRPLRFSYPAIVILFWSAEVVFVIRNYKTMDFFLIGHCYLTNVTTGMMVIRVAMYMMKTYDDIMEEYLKSFHIVHSRALSPYYEKLFNYEQKFSSMFAMAMMIFTCTGMLLYSSLPIYNNFHTGVFGREKGSNLTIDLSIYYYFPFYNPVEHVVSACILNFYLSYLCSFMTMVSDLVVYLMIFQIITHIYCLLDDLANIPKPKNFGKNSTFQHYDMIIPLAEMYDAQENAIIQGILGRCVHHHRIIVTFADKISSFFGPMLAYNYFYQVTSLCMQLIEISRGDFNTFVRYVFFTLLVFSELIFMSVIFEKVGSTSEQLIEAVYDVPWEYMSISNKRIVSVFLHRVQSPISITAIGMVPVGLGTMTGILKTTFSYFAFLRTLNS

>CsasOR43

MKPFTMFHKTYYVTALFMSVGMIYPNPHTDRARITFIIFLIVTSLPLCFTMLLDIYNSWLRYDILNIIRHSTVLGPFLGGFFKMLLMFTKRVKAGELIKEFNRDLSLYNDLSAPYKNAARASIRNSNIYSERCWLIAIASCVSLFPLMAICQMLYSVKDVPTKYMIHDTNKPFSKDLDARFESPYFEFMFVYSLFYSMWYIINFVGYDGFFGLCMNHACLKMKIYCMMLEDAFKLDNISDVRRAIVKVIDEQIRTYKFIGNAQETFNEWLGLIFVATIIQICTCMYQVTEGYSLDVRYMIFMIVSVAHIYVPCRYAAKLKDTSVRTAVDFYCSGWEDLNDQSTRKMIMFMMARAQIPIIMSAFKMIDFDMKLFVSMLQSSYSMFALLKS

>CsasOR44

MAQRGITFLKGLEDPKYPLLGPNIRGLYLFGLWLYGSKLRTYCFYIIHSFSIMFVFTMTVQLYLYIKRNDYAKMLENISITALSIVAISKISYVVTHMGKWRELVENISEEERNVIKSRDPEILQITKDYTMYARLVTYFFWGIVLFTNAVTLVTPFITCLTPDYVEKLNNGTEVLPHILNSWFPFDDSKGYGYIASVIIHAYMTTQGAGIIASYDAHAMVIMTFFKGQMMILGSKCRRLFVIDDLITSEGIYSRIKECHRLHNYLMTQFKLFDSLLSPVMFVYMLVCSITICCSVIQLNLPNTSLSQKLWAIEYAAAQIFQLFLYCWHSNDILMESLAVDRGVFNNDWWAADTRIRRHLVMLAAKLNNVFQLKAGPFTRLSMPTFIDIMKGSYSFYTLFAQIPENK

>CsasOR47

MADSILEDAKREIDSTLSLCTFSMHRIGLSFDPPNTASALFRQKLMFTASVLGICYHVFSEIVFIAITLANSPRVEDVVPLFHTFGYGALSIAKVGVLWYKKDVFGHLLQELVGIWPMPPMEEAAKAIKDQKLLALKIAHQWYFMVNVAGVWFYNLTPIGVYLYHVWAGHDATIGFVWVSWYPFDKHQPIAHVVVYIFEIFAGQTCVWIMICTDLLFSGMASHIGLLLRLLQRRLLTLAETTKTDEENYQEILDNIKLHQRLITYCNNLEEAFSLVNLINIVLSSVNICCVVFTIVLLEPLMAVSNKLFLGSALIQIGMLCWYADDIFHANADVASCAYNSHWYRTSPRCRRALIFLIQRAQKPIAFTAMNFTNISLVTYSAILTRSYSYFALLYTMYSDN

>CsasOR50

MANKQIDCFSTNIKFWKFLGIWPENVHSHYKYYSHLFLATFVYLYWILFSINFIYLPRQLDTWIGEMIFYFTDISCLSKVLTFRLMHDHIAKLLNMLESDIFQPATDEGLKIIADVKKFNVKYWKIMAVVSVTSHFTHIFSPLLAHLISHVKLELPVSRYSFIPEKVKEQVAYPLYFYQTIGMHFLMWYNINIDTFFLAIMMFAISQLEILNLKLRNITSAAKLPRESRADAQVDVQRNDEYYVHKLTECIIHYTEVSKFCELIQNIFSITLFIQFSVASCIICICLFRFTLPAPLDYYIFLATYTFIMVIQIMLPCWFGTRIMDKSYELSMAVYSCDWTARSRRFKTNLRLFVERTNRPLSIIGGLMFTLSLSTFTSIMNSAYSFFTLLRHMQSREG

>CsasOR51

MKWFQVADYQEIYIKIKRNMKDYFIFKNICKCIYYVGPGNFWYKPGEVREDNTKGYKAFSTVLFSVYSIVTILEFMAAIFGDFPADEQSDSVTFAVSHTIVMIKIFSVIRNKQVLKNLCRDMIRVCEPYEDPSKMSEKYRIVKINVIAYMISVYGSVLFYVFEGIRKLYAGRNFVTVVTYYPVSEDYSALANCFRVATTIVLCMMMLTMLLTVDSFTMINLIILKYKLITLRDYFYNLREKFEVINKADPRLAADDLANGLIEGVKMHRELLRLSKEILNVFETVITCQVCQSVGAAVSLLLQIALSNDLTFSASMKIIFFVIALFFLLALYLCNAGEITYQASLLSDAIFHCGWHIVPNPARHRRIGRVVLQAVALAQRPLVMKAYNILDLTYGTFIQVVRGTYSVFALIYARNK

>CsasOR52

MSTIMRYYIGNVFVSLRVALTALWMVGYWAPKQLTEWKKTGFFCYGVTWYMVQIGFVALSQIADLIKIWGNFSLMTATAFLLFTKVAVTVKIFNLILRNDTIREIVDECNQELESEGKEAGSAEGEVIISSEIETRNLSSSFAVLSMLTVCSWAAAAEKNQLPLRAWYPWDTTKSPAYELTFAYQIVIGSIAAAMNNGVDAVAIALIGQCRCRLQLIALALRNLCQGLEPGEYKLMSSEQDKILRSRLITCIKKHQAALQSAKRLQACFSLPFLAQFAASVVVICVSGYQLAFEAWKPFRLMAMVSYLLAMMVQVFLYCYQGHFLIEDSTYLVNAACESPWYTLSVSSRRLLVIMMIRAKTATKLTACGLFDLSFQTYMAILKASYTFFTMLQQVVVRE

>OfurOR2

MMTKVKAQGLVSDLMPNIKLMQAAGHFLFNYHSDNSGMTTLLRKVYSSVHAFLIVINYLCMAANMAQYSEEVNELTANTITVLFFAHSVIKMLFFAVNSKSFYRTLAVWNQSNSHPLFTESDARYHQLALTKMRRLLYFICGVTVLAVMSWITITFFGESVRMIANKETNETLTEPAPRLPLKTWYPFDAMSGTMYVVAFVYQVYWLFFSMAIANLMDVMFCSWLIFACEQLQHLKAIMKPLMELSASLDTYRPNTAELFRASSTEKSEKMPDTVDMDIRGIYSTQQDFGMTLRGAGGRLQNFGQPNPNNPNGLTQKQEMLARSAIKYWVERHKHVVRLVASIGDTYGTALLFHMLVSTITLTLLAYQATKINGINVYAFSTIGYLSYTLGQVFHFCIFGNRLIEESSSVMEAAYSCQWYDGSEEAKTFVQIVCQQCQKAMSISGAKFFTVSLDLFASVLGAVVTYFMVLVQLK

>OfurOR3

MFKIGNENDINARHPMDLRYMKFLRMLLRMIDSWPHQQLRDSKPVRFRDSRYLFIEGAGVGIGGLFYVRSHYKVVPFLEIGQTYLTIFLSVVATQRVTIAWFKSFREVITEFVLKIHLFYFRHKSNYTENVYQRINRLCSVFVAFVAVEVTIGIFLFNLMPFLNNYKKGMFNQELPANKVFEHSINYSLPYVDCYTNLIGYIVMTLINIICSYDCGMFFSSVDVCIAVIVFHIWGHLKILDHRLRTFPTPVQMRGHQPGEPGNDLMYTKEENMKAAAMLRDIIEYHGMIMRFMTKTSEAFGPTLCLYYVFHQVSGCILLLECSSLDPESLGRYAGLTVTLFQLLIQVSVIVELLGTQSETLKDAVYSMPWECMDTSNRRTVLFLLYNVQEPIRLKPMGIVSVGVQTMATIIKTSFSYFMLLRTFT

>OfurOR4

MPAVHQNPSTLSYIITVKNALGPSGIWPSNIFEDKLQPLFFRIHRETLPYHTMLIVFGGLYYLSDNFRIMSFLDMGHIILSTFLAMVTAMRSVVPNLKIYVALLTKLGREIHLMHFAHKGPYYEEINKTVDKASHIYTKFIVVFMYMTMMMFNITPIYNISKNILSSKTENSTQEYALYYSFPGINPMNYYPTTTVYNFYLSYNCGIMMCGLDLVLFLMIFQLIGHVYILRHNLENFPSPKNKVVLNIGDLPRYKNKENCIVEMFDAKENEEVRVRLAECIEHHKIIIRFTDEISVVFGPILAFNYMFHMVGCCLLLLECSAGNQIIRYGPLTTVVFGQLIQISVMFEMLGAETEKLKDSAYFVPWECMNISNRRTAQIMLHKMQDKISIKALGLAAVGVNTMMGILKTTFSYYAFLQTMND

>OfurOR6

MQQESPLQLGYIKTIRFFLRPSGSWPSDVFEGYLPLPIRIHRATLPFHTTIIVMGGLYYITDNFHRLSFLDMGHMIITTFLAMVTALRSILPNLQTYNSLLCKFIQEFHLMHHAYKGDYFEEVNKTVDKISSYCTKFSTIIMYLAILFFNITPTYNNIRHTLISKTENYSMEYSVYFSFPGFNPLDHFASTTVYNIYLSYNCSTLFCGFDLLLFLMIFQIIGHVYILRHNLENFQSPKNKITLNLRGDALITNNTCTYEVFDAQENEEVRLQLAECIEHHKIIIGFTDDVSGLYGPLLAFNYFFHMIACCLLLLECTEGSYDAVLRYGPLTILVFGQLIQMSVMFELLGSETEKLKDSAYCLPWEAMNTSNQRTAFIMLHKMQYKISLKALGLAAVGVNTMVGILKTTFSYYAFLQTMGDR

>OfurOR7

MVIIRSAFRVVGAWPSKFIGDVQTTSDVVVKYIQLVLNVVCQVAGILYLRENMDKLSFFELGHSYITVLMSVVSMSRIITYCTEAYQEIFSLYVRKIHLFNVRNDSEYAMEMHTKINKLCYFLTFFIHAFMTLGILMFNLIPMYSNYISGKFNRETGAFSGVSNATMEHAVYFLWPFNDTTDPIGYAIIVVFNWYISLVCSINYCTFDLFVYHLVFHIWGHLKILIHNLETFPRPIGAINEEQNDYTEEESKQIYERLKKLVQHHNLIIDFIARISDTFGLSLFVYLCYHQVCGCILLLECSTLELSALIRYGPLTAITFQLLIQVSLVFELLGSITESLMNAVYELPWEYMEVRHRRTVHIMLRQSQVSLNTRALNMVDIGSRTMIAIIKTSLSYFVMLRTFATDD

>OfurOR8

MSNILKYFNTRNSYELSFFREGDPLALNYFKIIRIFMVAPGAWPADVFGEKLSLLVRVHRALMPYHTSVIVIGELYYLYIHKEELDFLNMGHMIIFSFLGVLIAIRSILPQLRKYHLLLTKFVKVMHLMHFKNKGPYYKQINETVDKISYYYTIFVALLVTTAMINFNIVPLFNNVTNVLIYKTENFTLEFALYYKYPGFDPLDYFTSTTIYNVYLSYNCSIMVSGIDLILFLIIFQIIGHVYILRYNLENFPSPKIKVVFKLKEILKHKGNEDISSEMFDAEENREVRLKLQECIEHHKLIIGFTDELSELFGPILAINYFFHLVCCSLLLLECSEGGAWIRYGPLTVVIYGQLIQMSVIFEMLGSETEKLPDSAYFLPWECMDTSNRRTACIMLHKMQYKISLKALGLAAVGVSTMTGILKTTFSYYAFLQTMGE

>OfurOR10

MFRLKEKDVIASNTQSQVFKPNIFFWKIFGWWPEVTSTIYYRCYYISFLSLTSVVYLFLFTLSLLYSPIELEIIIAQAMFYFTEISGLSKIFMIVIRREDIQKAFKMLDSEEFQGDDVIPREIINKNKVYYLKYYRACATFYYIGSFFLLFLPIIEYVAGHADLELPLCQYYFLSEHVRDKYFNVIFIYQFFGLFVLISGNVNIDTFICGLLLMAIAQFRMLNWKMSNLKMNPLDLENESDDEETIMMRKLNKCLKHYDLILEYCDHIQDVLSAAIFAQYGTAAATMCLSMCTVLMPMTSEDWLFMGCYIGAMTLEIFLPGLLGAELMNESQKLVAAAYSADWIPRSESFKRSLRLLVERANRPIVITGLKMFTLSLETFTSIIKLAYSFFTLLKNVQETEIA

>OfurOR12

MIIKFLQSFEDPDKPFYGPNFWILTKTGLILPENKIAKALYILMHEIVAFFVFTQYMELYIIRSNLDLVLTNLRISMLSVVCVVKANTFVFWQEKWNKIIDYLTEADSVERYSNDPERKKIIDKYTNYSRRVTYTYWVLVFITLATTIGSPFIHFVSASYRESLRNGTELFPHILSSWMPIDKNHSPGIFITIVWHFTVTSYGALIMSSYDTSIMVIMVFFGGKLDVLRERCKQMLGTGEVELSDDEVAARVRELHNTHVLIMKHLRLFDSVLSPVMFVYVVMCSLMLCASAYQLTSATNAAQKLLMAEYLIFGIAQLFIFCWHSNDVLVKSENVMLGPYESRWWDANVRQRKSILLLAGQLRISKVFTAGPFTNLTLSTFITILKGAYSYFTLLRE

>OfurOR13

MLNVLKRLENPKRPLLGPNVKALKFWGLLLPENIYMKYFYLLMHVLVTIFTATEYVDIWFIKYDLNLILNNLKITMLATMSVLKITTFLYWQQHWKDIIEYVTRADLAQRTTDDVEKNVLITKSTRYCRKITLFYWSLMYTTVVIVIFQPIFKYFLSRNYRENVKNGTDSYLQVVSSWVPWDKSTIPGYLIASAFQSYAAIYGGGWITSFDSNAMVIMVFFKAELELLKIDCSNMFGTETKPVSDEVALKRLKDCHRRHVELLKYSRLFDACLSPIMLLYMFVCSVMLCVTAYQITSETSAMQQFLTTEYLVFGIAQLFIYCWHSNDVYYASLQLSQGPYESLWWYRDVSHRKNLYILTAQFSRVVVFSAGPFTKLTVATFLSIIKGAYSYYTLLSKSQTK

>OfurOR14

MTILNSIWRKLTNTKALEKSSGCLETQFFETVYRVSYLTGISMADEDIPYLIYSSVVKLLIFLLIVGEFWHLATEVTSFDEMADMVNITVIQYIAIFRYRSMLYHKDVYKKLAISMESQYFDISTKERRDVVDYWVKRNANNVKLLLVLGNCTLIAWFLYPLVDDLEYNVFIGIRLPFPYYSPVCYAFVYLLLLIVFSYISHFVMANDLIMQAHLLHMVCQFDVLCNCFENLMEDCAKGFKGIDRESLLANANYREVFKARLGDMITQHRYILDHAMELRHTLSGPMLGQLAASGTLICFIGYQATTSGAYNITKCLMSLFYLCYNLLVFYIICRWCEEISVQSQRVGEAVYCSNWECGASNIPGVKVSLLMVITRANKPLTLTAGGVYDLSLMTFSSILKTSYSALTLLLRLKSTE

>OfurOR15

MALMVYQKVLEEKITENDEEIFFKPFEETFTILIFSMVFGMIYPSDRMKNWQILGFFGFIIIMIPALSAVYYDMYLAYLDRDMDTIFRHLIVIGPFNALYLKWVYMYYYRQQSKDAIEEMNRYFANLNFKPITHKRIAKKWLIRSFFLEKSWAYCLIVGSFSFPVMAICKTTYSTLFDEEPRRYFIHELRSPQGPGMNYEFPFFEVLFVNTCIASCMYFLNFSGYDGFFVQLILHTCMRMAICGEAVKDSFKIDDKAMRRSALHKVIDEHIAICHFMDNINCIFVQWMSLFTVALTIHVCICVFHLSEGTYQDMEFMFAFTAASIYLCMITSCGGLVEEESENLADAFYQSGWERVLDTHCNYLLVFMIARAQKTFRVRTLFHVHVNHELLIAIVKMAYTLLTFLKQT

>OfurOR20

MWNIRFLKEKRFTILNVFNFLEDPRYPLVGPHLRLLGLTGLWHPNLNSKTRFKQYLFFITIAFFFSQYVKCAVKLEPSSLMLILQYAPFHLGIIKSCFFQKDHKKWESLIDYISGVERKEIANGNKDSNDIISEYISRSRKVTYFFWALAFFSNFTIFTEPYQKNQINVNGTSVYLKIFDGYTPFSEVPPGYYASMLTQTVLGHIVSAYVVGWDTLVCTIMIFFAGQLKISRLNCANVIDINNAERSHENIVNCHSFHTILVKNQKLFNSLISPAMFVYLIVISVNLGVCIIGIVQLQDDLTTLISSCVFVMACLIQLLLFYWHSNEVTEESTLVSYGSFECDWVELDQRFKKEVALLGMATRTRLVFKAGPFNEMSLTTFIAILRLSCSFYTLLSKTM

>OfurOR21

PRLARAHALYCRFALAATSVYLAQECVYAYQVRNDMDKLARVMFLLLCHVTSITKQLVFYMSADKIDEMINALDDPLYNQPAAWQRALLAATARSAGRLLRAYSGTAVVTCTLWIIFPILYYSQGLPVEFPFWTNLDHSKPTFFVILLMYSYYVTTLVGIANTTMDAFMGTVLYQCKTQLRILRMNLENLIERATTVVKENSDEIFDKVLDRLFLECLEHYRQISETNRRLQDIFGTSILVQFGIGGWILCMAAYKMIGLNILSIEFASMTLFITCILTELFLYCYYGNEVTVESDRMVEAVYAMEWLHAPLRFKRSLVLVMERAKRPLRPAAGHLIPLSLDTFVTILKSSYSFYAVLRQTK

>OfurOR22

MLRNFLLSLENDNHPLLSPTLWGLQKWGLWQPNKVLNSNISNFIHFAATLFVISQYVELWLIRDNLNYALRNLSVTMLSTVCVVKAFTFVTWQDQWKDVIDNVSLLEKRQLSKKDKITDKIISEYTNYARRVTNFYWTLVAATVFTVILAPLVCFLSSPDTRERIRDGYEPYPEIMSSWVPFDRSRGLGYWVTVLEHILICFYGGGIVATYDSNAVVLITFFAGQMKLLSVNCSRLFDDEKEMTYEDDMEKIRACHYHHLMLIKYSKILNSLLSPVMFLYVIICSLMICASGIQLTTEGTTTMQRIWIAEYLMALIAQLFLYCWHSNEALVMSNKVDDGVYASAWWSRSIQVRRCVLLLAGQLRKSVVFTAGPFTKLNVPTFIAILKGSYSYYTLLNNKDD

>OfurOR23

MAEQPIDKSLRKIRFIFRYAGMNLEERPRTWCQSFIYVVNFLWIATDIIGEINWIFEGVSKGTSFVELTHVAPCLSLGTMSEFKTAFVVAHEKSLFRLIGNVREMERKRLVGPIAHKIVKEESKFLYNLVFAMKMVNWVLVVVFDFGPLVWIVVKYFIYGELELLLPIIDIYPFDCYDLRIWPFAYIHQIWTAWVVVTEILGVDCLFYICCTHVMIQFKILNHEVTNVIAESRSAKRIEVTQLREKFNELVKWHQDIINSAGLLEDIYSKSTLVNFLTSSLIICLTGFNMTALDNVRMTVAFAFFVVAMLQIYFLCFFGNMLMDASTDVSTAVYNSRWYLSDAAFGKSALIMQIRAQKPCIVTAAGFAEVNLRAFMKIISTSWSYFALLRTVYQDI

>OfurOR24

MFSLSFVITHDLTLIKLCIFYFKNDQIQDIVRTLEIDLYNFYQNNRKNRATVRITRIMSASFVFFGWITIGNTNVYGTIMDFRWKAEVAKLNASSIKPPRTLPQPIFIPWEYQTDQSYISTFVLETVGLLWTGHIVMAIDTFIGSVILHMSSQFTILREAIVTAYDRTITKMYINAKHQYDSLEAVSSNDEENSSIDQSTQDGMEALVLARFSKKEVELALQETLKNCFQQHQVLIRCVEKFAETYSYGFMTQLLSSMAAICVVMVQVSQDASSFKSIRLVTSLAFFIAMIIQLAIQCFTGNELTLEAARIADAVMQCKWERMPPSLRRMLIMVMMRAQRPLRLSAAGFAYMNNDCFLAIMKAAYSYYAVLSQKTK

>OfurOR25

MAKEAFENSLRLTKLFLLLSGIRITRRKWRKSVENFFDYYLYYISLSWLYTDVCGELNWLIEGILTGKSFIDLSLTAPCITISMLATSKSIFLYWNRDVVAKIVDKLRDIHPEDKEFDEYKQLGLYQVESNEPDVEKEIVEESRKFLSFVVHLLFYICAVVICAFPLMPVTSMAYDYYTTGSTECKYPYLVKYFFDPYTMKMWIAVYFHHVVSTAIVGANVFGSDSLFYVVCIYIQMHFQTLCHRCECAVVSSREGTRRNVANAVKRHQELIDLVNQVELLYSKSTLFNIVTSSVLICLCSFIITVLDEIIVVVTFATFLVMNLSQISLLCYFGDILMRSSTEVSSAVYNSLWYETDQSVKKSMLVILMRAQKPCKLTAWNFADLNLTAFTTILSRSWSYFALLKTMYK

>OfurOR26

MEEESLFDKSLKKITFAFRLTGLNIENDKRNLKQNCVYLFNFLWLNTDIVGALQWVLYGIASGKNFTELTYVAPCLALSILGDIKGVFMILNEKKVHILMDNLRSLELKAKEFENSEREDMIEPEIKFLNIITSVLNVLNCLMIVVFDASPLILIAVKYFTTGQLELMLPFLDVYPFDSFDLRYWPFAYIHQIWSECIVLLEICATDYFFFTCCTHIKIQFKLLQHQFQEIIPSRSVSAVDSIDQAAIRTKFQELIKWHQEIIRSANMLEGVYSKSTLLNFCTSSLVICLTGFNVTTIDDKAFVMTFIIFLFMSLLQVFFLCFFGDILMSSSMDVSNAVYNSRWYLTDVMMGRNVLLVQTRAQDPCKLTAAGFADVNLRAYMKILSTAWSYFALLQTIYC

>OfurOR27

MSDITLSEAKREIAESLTLNTFCMRRIGLSFEEPKNASSYFAQKFMLVLSVMSICYHVFSEIVYIGLTLSNSPNVEDVVPLFHTFGYGALSIAKVFALWYKKDVFKQLISELAGIWPMSPLDDDATVIKAKSLTALRIAHQWYFVINVLGVWFYNLTPIIVYAYRVWQGQDVEMGYVWVSWYPFDKHQPVAHVAVYIFEIFAGQTCVWIMVGTDLLFSGMASHIGLLLRLLQRRLETLATMEQTEEDNYRDILASIKLHQRLIRYCNDLEVAFSFSNLVNIILSSVNICCVVFTIVLLEPFLDISNKLFLGSALIQIGMLCWYADDILHANADVAAAAYTSGWYRTSARCRRALLFLIQRAQKPIAFTAMGFTDISLVTYSSILTRSYSYFALLYTMYNDK

>OfurOR28

MIITYHQDLSTVVEFNTIPYNSMEDHTIRMEKEFGPFHDTYRLNMYSMSYGMIYPNPATNKWRLLAIPILCFTTIPMTVLVFLDIRRYWIDGEILEVIRHVGLIGPFITGILKMCLLYYKEEPTSQILALINRDYASFNQLPESYKPLVRGYVKNTRFYQNIWIACVLMILSTFVLTASVMNICETLFSSEPKRHMIYDVRLPIDRPGAQFETPYFEILYIYMLYIAAVYTINFTGYDGFMIACVYHACLRIELFCKYVHDAMGYEGDELRRRLGEAVNHHCETFKLIEKCESSFNFYLGLVYVVVTTELCLCLYLVMEGFEFDYKFSSFSIGTILHIYVPCLVAEKLKCVCENASDLIYCCGWENNYDLSMRKFIPYMMARAQKPVAMKALGLITFEMSLFASTMKTAYSMYTIIKTQ

>OfurOR29

MKWKKNKYGSRIVSTKKDNEFLQKNNLVTMITHRIKNIGLTFCVSDGIKIHWLAIFAIISFVLTQGLQIIGLFNAKDDIDKVFEYFSVMSFCGMGILKLLSLCRNHKQWKILLDNIKQLEKTQCQNETSNVEYESDGENDTFTFPSYIESYTKKFKIVSTVLSRMYGFTAIVYILSPFAEFTLLIMTGNEDYEKPHVLPGWAPFDSRSFVGYLTNVAVEIISVTYCVLVHITFDLTSIGVMIFICGQFSLIRDYSSNIGGSGASCTLSKRREDRAHHRIITCHKIHCLLMNTCDELGKQLQNILGVYFSVATLTLCSVAVRLNSELSRMELASLLQFMCATLTQLYLFCHFGHNVLHQSSIGMGDGPFGAAYWCLSPRIRQELVILGMGMMMPRYFKAGPFISVDLPSFVQVLRTAYSYYAVIRK

>OfurOR30

MATFNSEDLFLSRAKFVMKFLGVWMPPVDETLPRKLFKIFMLTLQYLFLIFQTIYITQIWGDLEAVSQPSYLLFTQACLCLKITIFHVNIDNLRELLKQMGSEVFLPQSRVHEEILKTQAARIKRFLLAFMISSQIVCTIWVVHPMLQKTGPRKFPFDMWMPVSPDDSPQYEIGYAFQLLTICMSAYMYFGVDSVALSLVIFGCAQVEIIKDKILSISPVQHRLKESERKIIFEKNHKILVECVIQHQAVVTFTQLVEDTYHWYLLFQLTGDVGVTCMSALNILAQEVRSLQFVTILIYVIVMLSQLFICCWSGHELTATSEGLHTVLYQCIWYEQDLKFKRDLRFVMMRMSRPMVLRAGHYIGMSRQTFVAVLRMSYSYFAVLNQANRVEQQ

>OfurOR31

MKFFVVNMYTDLKISLTILLYTGFWTRQKEVTNILSYCYPFLTFMFMAGISIIAQFVDLLHVWGDVSLMTSSSFLLFTNVSFGLKMFNILWKREEVRAIIDDCDQVLRAVDTSWGYEIVKSGIRKSFLLFSIYTFLANISVFGWAISPEKGELPTRAWYPYNTTSSPGYELTYLHQVSAVLLGASVNASLDTVVISLMAQCTCRLRLLAAALRMLGGDMLVTNMFEAEQERAIREELQRCVQQHRSVLQVAGLLQQYFSTSILAQFSVSLVIICVTAYQLAFVSSNVLTILGMTTYFMCMLMQVFLYCYQAHELSTVSSQVGDAVYESLWYEMSAPLRKDLLVLMVRSQRVIKITAGGFTTLSLNTFMAIIKTSYTLFTVLQRED

>OfurOR34

MTSTQANGNRVYSRNDYDETYKLIITNILAKVGIRMTRKDSKYARLGWNVFFCFGFGNMVVTLFLDLVTFQDVVRSGVGEDGYIVFMMLPCMGYMALAMLKTYKMVYKRDVFENLISELREMWPEGLVTEEEHTIISRALNELNIIVKGYYWCNLGLGVSFMAPSFVVAIRRIFGADIPPSLPYFYWLPYDQSQPVAYEFTLVMNTYHTLLTLWYMLAGDLLFCVFLSHITTQFDLMSVRITRLFQVPVDQQLIPEYPLGQQIKDFPENGHLPRLSNNEINSKQENELQKIIVRHNALIRLSGDVEDLFSFAIFINFFNSSIIICFCGFCCVMIEKWNSLMYKTFLATSLSQTWLLCWHGQKLLESSERVADALYNSGWYTAANGIKKSILIMIHRSQKNVYVTTYGFSIICLASYTAIIKTAWSYFTLLLNTYNP

>OfurOR35

MGIVMENVKKRLTILQPILPYGVIEPWDDLNPRLYHAIHIYWLKFYGMWYNNASPKTIVFWLQLIYTATVLWLVCFLPGIGEVVYLLKRRGNIGDVAEGLYLFLSEMYTYFKVAVFWLNKDKVINLLRYLSCEEFKPVEMEHREIIRKSIKAARFVMTYYSTMCVGAVSVGIIMPLTENFDILPTNVEYPYFDVYKSPVYQTLYIHHVYYKPATCIIDGVMDTILAAFVASAIGQIEILAFNLRNFDVVAERRRKRAVAENKPSAAWTQERHIRAVLKDCILHHNSIIKYVSMIEGTFSLASALQFMLSVMVLCLVGIQFLSIENPSSHPMQIMWMAIYLTCMLIEVFILCWFGDELIWKSTALRQAAFDGPWLETNHKTMVFIVIFLERCKRPLRVTAGKIFTLSLDTYTILINWSYKAFAVVSNMKK

>OfurOR36

MKDYEILKKHCKRIYLIGSGDFWYEDGTIGDDKSWYYKVYSWSLLSVYGFMTILEIMAAMIGDYPEDEKRDSVTFAVSHTIVMLKIFSVHSNKQMIKAMNKNMVYICEAHEEPTLMAEKYKIVKINVLAYFSIVYGSGLFYVFEGIRKIFAGSHFVTIVTYPPSYEDDSLYSVAFRVSTTVILFMLLLTMIVSVDSLTMTYLIMFKYKFITLRNYFERLTEDFYKMNDVNPREAADKLTNGLVEGIIMHKELLRMAKDIDQAFGTVIALQLCQSSGSAVSLLLQIALSDQLTFVASMKIIFFVAALFFLLGLFLCNAGEITYQASLLPDAVFYCGWHACARQPPRRSARRIVLLACAQAQRPIVMKAFKMIQLSYSTFLQVLRGTYSVFALFYAQNK

>OfurOR37

MIVKNVTTSVSMSLTALRLVGFWMPEHFGGNKRILYDCYGFFSFMFLLGTYLIIQAVDMCMIWGDLPLMTGVAFILFTNLAQATKIFFMVWRRKQVLTIIRGADEVLRAVESDEAKAIVKSCSRETTFLHIVYNCLTLVTMVGWGTSAEKNQLPLRAWYPYNTSKSPAYELTYMHQIGALCVAAFLNVCKDSLVTSLIAQCRCRLRLLGLSLRSLCKDLHATGKQYTAEQEAIVRARLCACVREHQAALVAAQQIQDVFSEQTFAQFNVSLVIICVTAFQLVSQTGNLVRLMSMGTYLVNMMYQVFLYCYQGNQLSEESAMIAGSAYECPWYLMSISLRRSLLIVMIRTRRVSKITAGGFTTLSLASFMAIIKASYSLFTLLQQVEGKK

>OfurOR39

MDELRLKVMVWSGIYKLHTKNRFLGICHDVYRVLMILYMSIYTVQHFVFIYMNVTRGDAINWQVAVFSIGMLNMVVKGITIYMHPESIDEIHDLIKDPMFAATCKEDEDIIKKNEYHIGLFIKITYVTLTVCLFFWVASIIVTRLVDDTAMPPSYFPFATNPWPQYIIATFVETVGSVLWFGYGHFSIDCSVACYYGRATAQLRIIRYHLEHFFDNGGAQGRFQYKDVVDRSLDEKFVYYVQCYQYVNRMIDNVSDAFNWGIAFHLCIVTTGMGMCVFIISTRDIFSLDMLFTVTIFVLLLLQNFMYCYCGDLVKSESDQVCTSMYFSDWTAVSPRFRRKMLIAMTRWARPIEPRVTIVPISLTTFASILRFSYTLYTMMKTRTM

>OfurOR40

MHCLLQMLNENIVRVIFIKVNSTYYPLSVKIPKTMNNYDILKNHCKKIFFSGSGDVWYEEGTIGDDKSWYYRLYSWSLFSMYMFMTILEIMAAMFGDFPEEEMRDSVSLAVSHAIVMLKIYFLYSNKNLLKTMNQNMVRICEAHEEPSLMAHKHRIVKITLRVYFGIVYGSTFCYVIEGIRKLFDGSHFVTVVTYYPSYEDDSFWANGFRIFNTIVLLMLMMTMIVSVDSLTITYLIMFKYKFITLRHYFKTCSQDFFKLNDVDPRLAADKLTDGIVEGIVMHNELLRMVKDFDQAFGTVMALQLFLSSGTAVSLLLQIALADQLTLVASLKMIFFVTALVFILGLLLCNAGEITYQASLLPNALFYCGWHACVWQPPRRSVRRLVLLACAQAQQPLVIKAFKMFELSYGTYLQVLKGTYSLFTLFYGQNQ

>OfurOR41

MNLLNFFKKYTEDDLINIQEHHFESFNKTYQWIAFTLTLGIMFPNPATDRFRIISINVLLVCVFPLAMMVLIDMYKCWMVKDIFNIIRHSTIVGPFLGAFFKMFLMYYKRAQAKEILDEINRDHASFNFLPRKQQDIAFLNVKKGVFNVERLWAPIVSIAIMTFPGMAVVMTLYSYAFSDNPKRYMIHEVKPPNSRDPEDMLKSPYFEILFVYETGSAIICVLNYTAYDGLFGIATNHACLKMSLCCMKLKEAFRCDSTEDMYKGILTFIEEQKKMFRFVDLIQDTFNIWLGTILTSTMIQIGSLLFHISAGYGFDLRYTLFSFTSVVHIFLPCKNAATLKDMSTEMSTMIYSSGWERSRERRILRMIPFMVARAQVPNYITAFNLFIFDMELFVFILRTSYSMYTLIRS

>OfurOR44

MNIVLVTTTNVYRTSCTKFVQLNTVAPSHSKIDSILNESSLFHVTMTHTFLHRPKTALTMLGLWLLPENYKVPYLIYRSFQLSIQFTFLLFNFIYMGVVWGDLEESSEGFYLLFTQATLCLKSTTFVMNRTRLIRLLRFMESDIFATNTPKHKRILAVQAVKMWQVYMFFMTCATCNVLEWAVVPLLESRGPRVFPFKIWMPADPAMCPDYVYTICYVYQAVTVYLSATTFLTIDFMTVSMITFASAQLEIIAEKIKQIPPVATSSENLKAEEVKSRVQHNNKILNECIQQHQAVIRFVGLVENMFNVNIFFQMSGTVAIICIIGFRITIEPPNSFHFYSTLNYLVTMVAQLYLYCWCGNELTERSQVLRDTLYTSQWYEQDRRFGSTLGIAMECMKRPIIFRAGYYIPLSRPTFVSILRCSYSYFAVLNQANNK

>OfurOR45

MDIPTFEELFKQIKLNLWFFGIPFNGRKIELRFYFMVVVVIIMLIGEISFFVSRYAPENFMELTQLASCICVGALSLLKILPIAHKKQKIFELTESLDGLYNTILENPKKKAIIRRQMILVKILMKYLFIVNIALFVIYNISPLIFMTYNYIATNEVEFILPFALGVPFSIESMATWFPVYAYSVFSSFVSVSYFVTVDALYCILTTHICSNLSMVSEELQNVDTSNEDELKELVKNHQYILKLSENLEEIFSLPNLFNVMMSSLEICAVGFNLTMGPVSEIPRSVVFLSSVLLQILMLSVFGEKLIEESTKVGDAAYNSKWYEVDQKTKKTILIIMTRSSKPQQLTAYKFSVISYGSFTKIISTSWSYFTILKTVYKPPE

>OfurOR53

MFPKTLAPTKINRNNFKFDNMFVVTAMAMFINRSHPSIPRNLFWVFQFLIILTLSTTTFLFLGNSVLLYDIPAGRYAEASKNGTMAIVAFTITIKYSFLLYFQKYMKNLISVVDRDYKLAMDFEEEEKEIVIMYAKKGAKVSWYWLLAALSTSIAFPLKAIFKMGYSYWQGDFKYIPMFDMRYPDRLDILKDIPAMFILLFVLCLMFGCYATTMYIGFDPLVPIFLLHICGQLDILSKRILKIFSENYNEEEINEKLKNVNIKLQDLYGLIENIKNKFTVLFEYNMKTTTFLLPLSLFQVVEDLKRSQLNMEFLSFFVATILHFYMPCYYSDNLLERSYYLREAIYSCGWETHPNTRARKTVLLMMTRTTAPLVLSTIFYTICLDTFAEMCRQSYAIFNIMNAACA

>PintOR1

MMNKVKTQGLVSDLMPNIKLMQAAGHFLFNYYSDESGMSMLLRKIYSSVHAVLIVVNYVCMAVNMAKYSDEVNELTANTITVLFFAHTVIKMLFFALNSKSFYRTLAVWNQSNSHPLFTESDARYHQLALTKIRRLLYFICAVTIFAVISWITITFFGESVRLIANKETNETISEPAPRLPLKAWYPFNAMSGSMYIVAFAYQVYWLFFSMMIANLMDVMFCSWLIFACEQLQHLKAIMKPLMELSASLDTYRPNTAELFRASSTEKSEKVPEATDLDIRGIYSTQQDFGMTLRGAGGRLQTFGQQNTNPNGLTQKQEMLARSAIKYWVERHKHVVRLVTSIGDTYGTALLFHMLISTITLTLLAYQATKIDGINVYAFSTIGYLSYTLGQVFHFCIFGNRLIEESSSVMEAAYSCQWYDGSEEAKTFVQIVCQQCQKAMSISGAKFFTVSLDLFASVLGAVVTYFMVLVQLK

>PintOR3

FVIMEAEAVARPTRYFAFHFYLLRFLGLGWWHQPDENDTRNFPSWYLYYSIVTQLVWVAGFVGLETIDPFVGKKDIDRFMFSLSFVITHDLTLIKLYIFFFKNDKIQEIVRILEIDLYGYYQNNAKNRTTIRTTRIMTSSFIFFGWLTIGNTNVYGTIMDLRWKAEIAKINDTALYPSRTLPQPIFIPWDYQRDFSYISTFVLETVGLLWTGHIVMTIDTFIGSLILHMSSQFAILREALVTAYDRTILQMYENVRFNADGSSLITIDRRMDIDDVDKIVRTRFQDTEVEKQIEKTLLSCIHQHQLLISCVENFARTYSYGFMTQLLSSMAAICVVMVQVSQDASSFKSIRLVTSLAFFIAMIIQLALQCFTANELTLQAARVGDAVMQSRWERMSPRLRRLLMMVMMRAQRPLRLSAAGFAYMSNDCFLAIMKAAYSYYAVLSQKQA

>PintOR4

MKILTENMKKMLSPLQPAIPYGVLESWEDLNPRLYHAVHIYWLKFYGMWYNNFSPKNILFWVQLTYTIVVLWLVCFLPGIGEVVYLLKRSENIANIAEGLYLFLSEMYTYFKISVFWLNKDKVMSLLEYLHCDQFKPTEAEHREIIRKSIKTARFVMTYYSTICVGAVLVGIIMPLTEKFTILPTNVEYPFFDVYKSPAYETLYVHHIYYKPATCIIDGVMDTILAAFVACAIGQIEILSFNLRHFDLVAQRRLQRAIAAKDPSGAYHRDYHIHSVLKDCVRHHNSIIRYVSMIEGAFSLASALQFMLSVMVLCLVGVQFLSIHEPAKHPMQIMWMAIYLTCMLIEVFILCWFGDVLIWKSIDLSHAAFEGPWINTDQKSMKYIVIFMERCKRPMKLTAGKVFTLSLDTYTILINWSYKAFAVMSNMKK

>PintOR5

MLLFSDGSDLRGITRTQDIKYMKYLRGTLSIIAAWPGYVIGESKGIGVGYKVYMDVIALVSLVCEALYVRQNVGKISFFELGQTYITTSMAVMCAYRTLAIWRENYSEIFKKFITVIHLFNHRNKSAYAMKIHLYVHKMCHFFTIYMIAVMVWGIILYNIIPLSNTIAAGGFQWPPPDNVTYDVSIFLTLPFDYQHSLIGYIVVFIFDWYECYLASSFFCIVDLYLALMMFHLWGHLKILVHNLEHFPRPAPRPDNMTQSIELCERFTDEEMQIVRQKLKEIVDHHNLILNYTDEVSETFGLSIALSYAFHQVSGCVLLLECSQMEPKALMRYGPLTLILLQLLAQMSVIFELISTMTDRLVNATYDLPWEFMDSSNRMTVLIILRQIQNPLGLKAVGMVEVGCRTMATILKTSISYFVMLRTMTMME

>PintOR7

MKIAAKDAKTPMDLRYIKILRLALRTIASWPGQELGEHVWMISNYYIYYLLLSLLIALIPVIRYVIQNAKTMHITVLGHICISVLLTFTSTAKIPLLCLRKEYRKFGKMFFTEIHLVYTKNKSEYAMKTHLRVHKLSEYCSIYLISMLSATIIFFNLGPMYVNYSQGLYRDGIPENSTNVYAHAAYYAGFPFQCDYLRDFDCYVVVSLFTWYISYFVGTFLSILDLYIYITVFHIWGHYKILINDLETIAEPNSNGKYSAEESHNITERLKNCIKYHHIISEYTKKMSNLFGATLFANICFMQLVSCLLLLECSYMTAETTMRYGPLTVMVFQELIQFGLVFELTGTAGDDLRKQVYNIPWEYMDTKNRAMVLFFLMNVQKPVRVKALGLTEMGLATVASVIKTSFSYFAFLRTID

>PintOR9

MEYLNKYPEEYARPLVSLFAYLGRLNVKFFNHDMPWFYRNWRFFYIVPILVFHFITMSVYIVEVFVEGVDVFTDVFMIPIYLVHIHSFSKMTILNINKERFEKVIDELGQTWRTDNLNDVQKDIKRSSEKELWMVQLVFMKLPICVASQYVLLPILDTLFCNVILREDDEVRLPLRCSYPFDPSSSWVLYGITYCFQTYCIFNLTFSYVATDFIFLCLSAQLSVEFMLLREDLLHIKPTKVVDNDVTHIVYDNGKIRDFVIYHQKLLRLAEELNCASNKIMFVNLFFTAVNVSLFGLAVLVANNASDKITNLIVILTILVLILTVCYSSEKLKSASEEIFNSACENLWYEGDKEYKQIILFIMKRSQKPCYLKSLDVPITHATFTTVMRTTWSYFSLMNKMYENEETN

>PintOR10

NTFAGELHAMAERKSLKEVYPQKYSLKLICRLLYYSGFGDYWYEPMDRSALEKRLYPIWVVISNGFLAFGIFNEFMAYTRADLTEKEMNDLMQHTFAHSSVYSKFVIIYLQRKRIRAVLKRFVEETRSIYSSSEIDKAATNKSFRFCFAMGFILFVTLTSAEIDGVRAHFQEDIPIRGEVIYYPSKNQTGILVNLMRFSAEFHWYYLVAIMTVADSLAICSMIYIEAKFKLLQLYFQNLGNTLKDETKGITEREEEYKKRFIDGIRLHEDALWSANSIQYALGTIYSVQIFESLSLLVVCLIKLVASELTLLRVIAILAYTTCMITLTGTYMIAAGDITHEASMLSTSIFHCGWELYLKKSEILPLAVVAIQRSQVPVYMTAFGVIELSYVNFISVLRSSYSFFAVMY

>PintOR12

MALTFQSTLKRTKIALLLSGIKLEDVKFGRILEFLFKNLFYFNFFWLGTDLVAETFGFIEGVQMGKNVIELALTAPCFSISLLGTAKSIFLFKHQNIVYKIILKLKNIHPESATENDKLIAIDNSDVKAIGTTEIDEIDNNNIEDKIVENSMKILNQINAVFNLFCSSVVIAFCLLPAATMAYTYYTTGDLVYEYPYLTKYFFNHYTLERWPLVYLHQVYSTAIVAANLLGADSLFHALCLYVEMHFQILCKRFEKSTVGDENTVHNTFMACVERHQELIELVNNMEILYTKSTLFNIVMSSILICLNGFILTVTSDVTIMIMFLTFLSMNLLQIFLLCYFGDLIMKSSVAVSSAIYNSPWYNTYSSSKKNVLFVIKRLKYFLSSNILLVSEVVNRELQYLFVYF

>PintOR13

MIRDKLTHFVQFLENPKHPLLGPNLKGLSFGGMWLKGPNLWICFQKFVNFFTFFFVVSQFMEAYNIRNDFNRLLQNLSITILSFTCYSKCMAFVFRLKRWSELFHSISEEEISCIQSGDKKVIVLMKEYIKYSRFITYSYWILVSVTNAAMMIAPVFKLFSSANYRKEVQQGILPYPEMLSSWFPFDNTKMPGYFFACLVHVSMCSKGAAITAVFDTNAVAIMVFLKGQMIILEEKCKNIFHNCKSRKEALRRIKECHRQHNFILRTHQTFDALLSPIMFLYVLICSMTICFSVVQYVSAGVTISYKIWIIEYTIAQVSQLFLFCWHSNDVLLESKAISESVYLSDWWKTDKILQKQTLILAGKMNRVVIFNAGPYTTLTIPTFIEILKGAYSFFTLFSQIQE

>PintOR14

MDTTRKVGIKIANENKFKTFSETYKLCAFALAIAYIYPNKATARKRLKMLLITIALNIIELYWYMCYLIVCIIRMDIYNFTRQITIGIIISLYLFKAFYGIFVTEKFEHILQEITDDLLRGNELSMDLQEIYFIYIKRAKIAQACWTVIPLLLGTQFTIYSAVCMIYEYMTTDLWNRYMVHEMELQHIQHLQYRTPYFETIFAYNCIQSLILAPNYSGFDGSFCIATTHMCLKLKIVGYSVQKAFKDINDRKDLRKKMKSLIEEHQKALLFHRKMQNVYGEWLFMVFLLTSILISFNVYQIYCIGRIDPKYTIFTLSAVLHMFLPCYYASDLIQANDEFQRDIYNARWETTGDPVVSRYLTFMIARSQQRLILTGKGIVTFDMQLFVTILHTSYSFFTLISTK

>PintOR15

MDYNIILYSYCKNIYLVGSGNFWSNENEVGDDNSISYRLYSLSLFSIYIFMTILEIIAVLYGNFPEDESSDALTFAVSHTIVLIKMFSVISNKKLVRSMNESMIKVCKKHEQNELVLEMYRTVKINVIGYFVTVYGASVFYVFEGLRKMYSGSHFVTIVTYYPSFEDNSMVATLFRVFTTAVLFMMMMTMIVSVDSFTMAYLIMLKYKFKTLRHYFERLRLDFDKIKKSGDQRLAAEKLTDGLVEGIVMHQKLLKLGKNIDQAFGMVIAMQLILSSGSAVSLLLQIALSDQLTFIASMKIIFFVAALFFLLGLFLCNAGEITYQASLLSEAIFHCGWHACPPLPPPHRNHQHLVRHACAQALRPPIMKAFKMIELTYSTFLTVLRSTYSVFALFYAQKK

>PintOR16

MISKFVSNLEDKRRPLLGPTLWGLKFWGLWQPEKGIISNIYNLIHIAAILFVVSQYIELWNIRSDLTLALQNLSVSMLSTVCVVKASTFVCWQKSWREIITYVSSLEVSQLSTKDKNTERIISNYTKYSRRVTNFYWNLVAGTVLTLILAPLATILISSRNGSTSYPEIMSSWAPFDRKNWFGYWVVYVEQAMSCFYGGGIVAAYDTNAVVLMNFFAGQMELLSINCSRLFENSQELSDSEAIRKIQEYHMQHLLLIKHIKILNSVLSPVMFLYVVICSLMICASGIQITMESTTKMDMIRIIEYLVALIAQLFLYCWHSNEVLVMSSKVDDGVYKSDWWKRDFKIQGNILLLGGQLRKTVCFQAGPFVNLNLSTFIAVIRGSYSFYTLLSNKDS

>PintOR17

MKWFVKNQTQSLAIALTALRLVGFWAPESLVGRKKTIYDAYAGFSFMVLLGIFLIAQSIDLFVIWGNIPLMTATAFILFTNLAQAAKFINLAVREKKIRAIVDSADTVLRDADTHEAKSIVKNCDRQTRIQLVAFFTLTLVTITGFATSAEGANLPLRAWYPYDTTKSPAYELTYAHQVYALFVAAFLNVAKDTVVTSLLAQCHCRLQLLSLSLRTLCDDLPVTGLNQLTPHQEKSLKSRIQRCVVHHQTALEAATLMQKYFSEPTFAQFNVSLVIICVTAFQLVSQTDNMVRLVSMCTYLVNMMFQVFLYCYQGNQLSEESSEIANAAYLSPWYTMSPPRRREILFIMTRSRRIARITAGGFTTLSLASFMAIIKASYSLFTLLKQVDDTN

>PintOR19

MGAYKQIDCFNTNIKFFRFLGLWPTDSGKYYRYYALSFIIFFIVIYDFVYTINFLFLPRELVVFIDDTMLYCTVISIMTKTFTFYLLHGKIIEILSVLESDMFQHDSDEAKTIIAGAKKLTIKLWKIITTFSLVANGSHIASPILAHLMTGEELVLPVFSCDFLPISVKNLLIYPLYFYQSIGMHFHVLYNMTTDGMFFGLIILLIAQLDILDLKLRNVTTSDKFNYEKEQEAIRNLNDCIIHYDEVAKFCQLIQDVFSVMLFVQFGTASMIICLCLFRLTLPASTAYYVYIMSYVFVMIFHRAVPCWYGTLIIEKSTSLGCSIYDCDWTPRSGPFKSSMRLFVERVNKPLIITSGKMVMLSLKTFTSIINAAYSFYTLLRHMQSREN

>PintOR20

MVFRKFLSYFEDPKYPSVGPHIRLLSLTGLWHQSNSKIEKVKLYLFYLTVAFFCSQYIRCLMKFDAESLTLILQYAPFHMGIVKSCCFQKDYKKWENVIFYVSSLERDQLLKKNEQSVEITKYIKQSRRVTYFFWALAFFSNFSIFSEPYRKNHENEDGNKIYTQIFDGFIPYSSEAPGYYVSMAIQTVLGHIISAYVVAWDTLVVTIMIFFTGQLKVARINCRNIIKNGEGHENIVSCHRHHTKLVKYQKLFNELISQVMFVYLVVISVNLGVCIIQVAELQDDITALISSCQFVIACLIQLLLFYWHANEVTTESVLVSYSTFESNWTEAPASVQKEVALLGITTNKRLVFRAGPFNEMSLRTFIAILRASYSFYTLLSNTNK

>PintOR22

MNLRKGATLTFGGEIHSLASKNNIFQLNRCILCFFQTRFITLLASRKLDDVIVDFVHDVHLFNHRNNSEYSSKTNLFIHKLSHFYTIYTLGLVSLVAAFFNVTPLINNISNGGFSGHLAANVSYEHAVYYALPFDYQHDFTGFMIIFTFNWYISFSCGMIFLNYDLIVALVVFHLWGHLKILTHSLQYFPRPGFIFGKAAENDDENAVANTETHYSDEEMVEVGKRLKNIINHHKNILKHSANISEAVGINVAMYYMFHQISGCLLLLECSQLDPKALIRFGPLTICLIQLLTTVSVIFELISSMTDHVVRAAYYTPWECMDTKNRKLVITLLRQTQIPLGLKAMGMVEVGVQTMASILKTTLSYFMMLRTVADNE

>PintOR24

MVVGQYAYIAYLLMNNVSLEQLIGSYLHIAGYDTMSFGKLLTIWYKQSSFRQVVNELADIWPVSEEDQKAVAVKDNSLTSLRRRQALYLFWNVLGVWLYNLTPVVIHLYRLGRGMSSDLGYIWQIYYPFDKTKPFVHELVYIFETFAGVASVCCMLGSDVFFISMANHISLLLRLLQERIRRLGMSDVDTNGVLITGTQDCYEQIVGVIKIHQRLISYANDLEDAFSVVNLINVLLSSVNICCVVFSIVFLESWMEMSNKFFLGAAMTQMGIVCWYADDIYRASMGVSDAVYESGWYRCNTRSRRGLLAMLQRSQKPLYFTALKFKTITMTTYTSILTTSYSYFTLLYTSYRQE

>PintOR26

MESQLSYLSLHRNVTFLKMLGIYPIDPKSSKIKKFFHGIYRYIMFFFILLYTVQEIMKIYEGRHNADKVIETIFLLLTYTDYIFKIIVIRMKSDQIEDMLLITKGPIYNQGEVEHRAPLLNTIRAGLLFVRLFNLMTLFTCFLWGLQPTVQHLQGKSIELAIWLPFDVNVNPYFYFAVFYMWAQTSLLALGNSTVDSFVVYLLEQCKTQMTILRCDLENCVEKCKFQSKETPISFSENLEIRLIKTIIHHREIVKMMNQVQAIFGKAIFYQFVVGGWILCTSAYRMVSINPVSVEFISMIFYMSCILIELFIYCYFGNEITIEVSFCLPNYNLN

>PintOR27

MDKLARVMFLLLCHVTSIVKQIVFHTKAHRIDLMVISFDEPAHGGAAPAEWRRALLEHTARSMARLQRAYAGTAVLTCTLWIVFPIMYYMRGQNVEFPFWTGVDYSHLGKFSLVLVYSYYVTTLVGIANTTMDAFVATVLFQCKTQLSILRANFETLSERAVEKSYNREQYDFILNQLFLECLDHYKKITVILSLLQDIFGSAILIQFGIGGWILCMAAYKLVSLNVLSVEFASMTLFITCILTELFLYCYYGNEVQVESDKLMESMYRSGWLGAGLRFQRALLLAMERAKRPLRPAAGLIIPLSLDTFVKILKSSYTFYAVLRQTK

>PintOR29

MEIMCLFITNVNFFYKIMVLKVKYDKVEEVLQITKGPIFNKGEKEHRVPLMRTIRDSLRIISSFNYISLFTAVLWSIRPVLERYQGKEINIQIWLPFDTDVTCYFYICVLYWWIQVSILAAGNSIVDGFIAFLFEQCKTQIKILRLDLKNAVEKSKVAEIETSDSFSNVLEEKLKNIIIHHKEIVKMTNIVQDIFGSSVFNQFLISGWILCTSTYSLVSANTASLQFVQMLFYICCMLIELFMLCYYGNKLTFESNRLVEYAYESDWLEIPVKHRRLLIAFMERIKRPIQPTAGSFIPLSNNTFISIIRSSYTFYAFLKNSN

>PintOR30

MKILTAQRDVYRVCVTNFMKEFHLYHFQHENKYNKKKAIKTERFSLFIQCFLILSIFLDALMWFITSLAKNTIHLEEIHNKTMILQGGTYIWMPFDHTYNFKNWILLEIFFMTYLQFGIGLVIFCVDIMTFTFMLHLIGHLDILKNDIESQDWIGLSDLAVRQKLVDIHNRYTFIKRNFKTFEAAYGINIGAIFLFNLIADSLLMYLLMFNEKENQMLYFIMAFYFFNILIALSYATEKIRKLSEDVFIAIYSCPWESMALSNQKMLLLMLVQSQELLDFKACGYSIGYQPMISIIKATFSYYVMLKETNE

>PintOR31

MDVPSYDDLLKQIKYNFWLLGIPYDDPKIYIRYFIMYISLVLMATEEVLFILSKISTEDLLVITQLTPCSCIGILSLLKMVCLTVKRKKIFSLTQSLKVLYVNIINDKEKKDVISGNIKVVSTLSKYFFVLNVVLVSVYNFSTPIFILYDYITTQKVNYKLPYQVIVPFRTDTWATWLVIYIHSVLCGFICFLYFTTVDVLYCILSSQICNNFTLLSKELLKINKNNVTNVRNIIMQHQHIIRLSEELEDIFNLPNLFNVLVGSVEICALGFNLTITLFLSTKINEVVDSATSNTKHPDTYHYFIIIVG

>PintOR35

MEIKVKDVASMQFKPFHETFPYCTFGLALSSIYPNKYNIKKRILTFVTIITFCVFQIFWFLTYTINCLLKLDILNFARNMTLWIIVILFFFKTIYAIKYTEDYAAILNMISKDFVKANEMDADYQEIFKTYLKKSKFGELVWLVIPIILSSQFPIYPAILMIMENLETDSPNRYMVHEMDMIYVEHIQYTTPFFEIYFAYNMTQCLFLSPIFTGFDGSFCIATNHLCLKLKLMTHKVRRAFKDAKKLGAIEK

>PintOR36

ALILPFLDIYPFDSFDLRIYPFLYFHQIWSEAIVLFQVCSADFLFYTCCTYLYKQFTHLQYQFADIISQNEVKSNPDELRQNFTILIKWHQDLISNALKLEVITSKSVLSNFLMSSLVICLTGFNITTTDDFSAMITFMTFLFMSLIQVFFLCLFGDLLMKSSSDVSDAAYNYKWYLSHVSTGKNILLIQTRSQTPCKITAAGFADVNLRAYTKVNNVKTVIAVFSAAGSILLDTLPQG

>PintOR37

MATVAFVVCFFLQTIELVTAENSARLFECFSILSFCSMGMLKLYFLRIYRKIWLHMLAQMRKLEKHVGHNEMPEEEYESDDDTLDLTVFVKNYTQQFESTSTLLSKIYRSTVTGFITSVFVEHALLKGTDRWHILPCWSGFDHVHVAIYMATILMEFIASIYCVSVHLAFDRSAAGVMIFVKGQFLLLRRYCENIAGKGRKCNISNNRDRRALYRIKYCHQRTLILNE

>PintOR38

MYTTVIIVIMTPIFKYFFSPNYRDNVKNGTETYLEVVSSWVPFDKNNFAGYLIASVYQSYAAIYGGGWITSFDTNAMVIMVFFKTELELLRIDCGDIFGSEKVRVPDKMISERLKNCHKRHVELIKYSTLFNSCLSPIMLLYMCVCSVMLCVTAYQITIEKSAAQRFLTTEYLVFGVAQLFIYCWHSNDVYFASQDLRMGPYESTWWLHRVHRKDLFIL

>PintOR39

MNLSNLSTLFIITVIVVFVYAGAADYLFYTCCLYISIQFKMLQLQFEILIPDDRSQIRLKFEDVENKFVELVEWHEKLISLTNMLEVVYGKITLYNFVTSSVLICLTLFDVTVIKDVAFALTFLFFLSMCLVQISLLCYFGDKVTQASTEVSDAVYNSLWYKCDISVGKHLLIVLMRSQKPCKLTALGFADVNQRAFMRIMSNAWSYFALLKTMYH

>PintOR40

AGTIADVKMKLLVSNVTWSLRLSLTALRFGGFWWPKADGTWKILYNVLTAFSVVYMIGIYLIVQTAEFICVLGDVSQLSRVGFILFSNLVLGIKMYNLVIRADEIRGLVDEMDRRLRDAKGDDEIAIIKSMDKLTMWQLYVYCFASFNTVYGLAISVRKPYLPLPARYPFESTISPAYEIVYTHQVSAITLGATINMSLDTLVTTLMAQC

>CsupOR2

MASSTRPRHYFYLHFLLFRVLGLGWWHQPDERDTRNFFGWYLYYSIVTQFVWVVGFVGLETIDPFIGEKDIDRFMFSLSFVITHDLTLIKLYIFYFKNDQIQDIVRTLEIDLYNFYQNNAKNRATIKISRLMTASFVFFGWITIGNTNVYGTIYDIRWKAEVAKLNNTDLAPPRTLPQPIYIPWNYQTEEAYISTFVLETVGLLWTGHIVMGIDTFIGSLILHMSNQFSILREALITAYDRTMIRLYEGVRQDFIAITNSDIDKKEQYTQDNIEEVVKSRYSKEEIEVALTETFKNCFRQHQALIGCVENFSTTYAYGFMTQLLSSMAAICVVMVQVSQDASSLKSTNLVTSLAFFVAMIIQLALQCFTGNELTLQASRIADAVMHSKWEKMSPKLRRLLMITMMRAQRPLRLTAAGFAYINTDCFISIMKAAYSYYAVLSQKQST

>CsupOR3

MCFNIASVRHTTDNSTQISFEKKSLGALFTIVNLKVKDIRKMARSQRLSIIASIRHILSTAGIKFTDTMHVHWMAKVAMICLIFTYVLQASALIQIRHNWEFFFECSGDLFYRGMSLVKFYIFRRNYETWCSLIEQADKIEEDELSNERDRENGNFLFSEHIQAYSVRYEKIKKIITTIFRTCTVMYVSSAFIEYGIKKQTVDGSVDLPHILEIWSPLDVSIVGYIITVSFELISAVYDTSTQIAFDLTSIGAMIFISGQFSLIRHYSEAIGCKEQIYPSKEQDDLAHKQIIICHQIHIQIKHLTEMLKGLLTNILWLYFIMSTVMLCSIVVRLNLETSLVQLMTMFLYMCGITTQLFLFCYFCDDIQNKSAIGMGEGPYGAAYWSLSPRIRKELMILARGMSIPCQLYAGPFIPVTLPSFVQILRTAYSYYAILGNRG

>CsupOR5

MKILKRNIKEKLAILKPILPYGVLESWDDLDPKLYHAVHIYWLKFYGLWYYDFAPGSFMFWIRFLYTMLVMWLVCFLPGIGEIVYLLKRRDDIGDIAEGLYLFLSEMYTYFKMSVFWLNKDKVLNLLQYLTCEHFKPIEAEHREIIKKSIGTARFLMTFYSTICVGAVSVGIIMPLTENFDILPTNVEYPFFDVYQSPAYGILYFHHAYYKPATCIIDGVMDTILAAFIVSALGQIEILTFNLRNFDVIAKRRHKRALDGNKPEASWSNERHIRAVLKDCIIHHKSIIRYVSMIESAFSLASALQFMLSVMVLCLVGLQFLSIENPASHAMQIIWMAIYLTCMLIEVFIICWFGDELIWKSRGLVQAAFDGPWLKIEQKDKIFIVIFLERCKRPLRVTAGKIFTLSLDTYTILINWAYKAFAVFSKVKK

>CsupOR8

MERMIKNLCDILISVSYREVTTMLRPFFKRLENNNHPLLGPTLWGLARWGMWQPRLGINTKIYCILHIVATLFVISQYVELWIIRYDFNLALRNLSVTMLSTVCVVKAGTFVIWHDQWQEIIEYVSKCENRQLSKRDKITSEIINNYTVYSRRVTYFYWALVAATVFTVTLAPLAAFWSSKEYRARIRAEQIPYPEIMSSWLPIDRTRGIGYWLSIVEHTLICFYGGGIVATYDSNAVALMSFLAGQLKLLNTNCSRLFEENYESRNNTVAKIREYHHDHLRLIKYSKILNGVLSPVMFLYVIICSLMICASAIQIATNGTTSMQRIWIAEYLMALIVQLFLYCWHSNEVLIMSHKVDDGVYASSWWSQSQSVRRSVLLLGGQLRRPIVFTAGPFTKLNLPTFLAILKGSYSFYTLLINKED

>CsupOR9

KRRRQWLSALIQFQLNQQKSTMLLDRLISFAKRWEDPESPLLGPNLKALHLFGLWKTDIRVRSTLLIIVVVFVITQIIDLYLSREDINKALCNFSLTTLSVICIAKSYSLIVHPVLWKKLVENISQEEATQIKKQNPETLSVIGNYTRYSRFISYTYWIMVAMTNFALIVAPLIRYLTVSKYREDIANGIERYPHIINSWFPFDDNAMPGYVYASAIQIIMSIVGSGSLAAYDTTAFAIMIFMKGQLIILKNNCKELFRWETKENNIEFFAKIKECHRHHDFLKRQFNQFNDLMSPTMCLYVLLCSITLCCSVVQLISKEATASQRLWIVQYSSGQILQLFLFCWHANELFLESQNIDGGVYASDWWKADVRMRKQILLLAGKVNYPMLLRAGPLSVLSLSTFFNIIKRSYSFFTLISRMQE

>CsupOR10

HPSAKSPIQRKSFDIKGVLLERVLVSSISMDLPTYDEIFKGIKNIFWLTGIPLDAPHKKLRFYVACLSLIITLYGEIAFFTSKISSENILELMDLAPCFCIGALSFFKGIFLAWKLNKIIVLKNSLEILYDTIFKSDSKRKLLHREIMKVHKLVKYYFGVNTALITVYNFSAPIFMTYHYLSQGKVKFMVPYAVIYPFAIDNWPAWIVAYTEQVFSGFVCILFITMSDALFCVLTSQICNNFYVISDEIKRLKNGNHIGLGEIVKQHQYILKLSEDLEDIFRLTNLFSYLVGSLEICALGFSITIGDWSHFLGYILFLVSVLLQILMMSVFGENIIRESGRVGEAAFLCEWHEINEKAKRTILIIMIRSHKHQKLTAYKFSVISYGSFTKIISTSWSYFTILKTVYKPSEVNNI

>CsupOR11

MNVISINPAEYRNTLILSLNYLKICGITLDRNDSFWEKYCHLCVVSIIMMLHFVSASLYIVQELVQNILQEANFISLWLITVQVFLRGIIILTNKTSIRGIIEQIGCNWRSSDLNEEQIRIKKDFLNRLLYTQKVIKIIGWCAGSLFLLPPLLETVFRSFVLHQDSAFVLPFPCYYQFTVTGWFTYFIAYFIQIYCSSKLIFMYLGADLLLIVLCAHLSNEFELLQVDLGATIKPTKNENEILEDEITAFGREERSIGDFVRRHQKVILLVQLLNISFNKMIFINLLFAAIAIVFFALGGRASRDPTNVANNYMAILVILINMFVLCYSSEMLCTSSSGIADYAYNNIWYEADMRYRTDIYFIIMRSQKACSLSSLNYLPISLSTFGKVLSTTWSYLSLANTFFEN

>CsupOR12

MKILTTQNENSEKEIRIKPFHETYKKITYGLTLGFMFPNPRTAKIRIVTIVIMLVLFQPIVVTVLIDMYSCWQKSDMFNIIRHSTILGPFLGAFYKMFLMHYKRAEVKRIIDEINDDYLTYNNYNHELKQIALESIKSSVFFVEQLWTYTVTACIMAFPVMGIVLTFISHLTQSEPKKYMVHDLKIPFRPPEDRFETPFFEIMFVYMFLAAIICVLNYVSYDGLFGLACYHACLKMRMFSKKLEYVFQCKDGDAYSRLVQVIEEQKATYEYNALVQNSFDIWLGTIVISTMIQLGSLLFHISAGYGFDFRYMLFSCTSVVHIFLPCTYASKLRNTSVETSTLMYCSGWERSRERRIVRVMPFLLARAQIASRITAFYLFDVDMQLFVTMMRTSYTIFTLLRT

>CsupOR13

MKKNMKEFEYDFEKAFRITTKALHLNRAHPFIERNLFWCFQFLLILTLSVMTFVFTFNSLLFYDIPAGEIAEASKNGTMAIVSLTITFKYTFLLYNQNYIKRYIAIINKDYELSKGFVAEERAIVIDYSRKGAKVSLYWLVATTATSILFPVKALVQMVYYHWEDEFRFVPMFDMRYPTTIEIMKNVPAMFCLLFLLCLMFDVYATTMYVGFDPLVPIFLTHICGQLDILSQRIMDIFSDESNLNSQEVNYKLKCINVTLQDQYNMIKEIKSKFTFLYEFTMKTTTILLPLSMFQIVEDLQRRKLNLEFISFFFATILHFYMPCYYSDMLMDRSQKLRDAVYACGWEKRHNARARKTILLMLTRTTVPLALSTVFYPICLDTFAEMCRQSYAIFNIMNAAEV

>CsupOR15

MFYLLKKLEDKNRPLIGPNVKALKFWGILLPKNLYTRYLCILMYLLVVIFVGTEYVDIWFVKADLNLLLNNMKITMLATMSVVKVSTFYRWQQHWLDILNYVTRADLTQRKTNDVNKIEMINKFTTYSRKITYAYWSLVYTTVIFVVGYPIFKYVFFSSYRQNVLNGSEPFFEIVSSWVPFDKSTIWGYILASIYQAYSSIVGGGWITSFDSNAMVIMVFFRAELELLRIDCANIFGTEKAQVSDEVAMVRLKDCQRRHAEVMKYIHLFDECLSPVMLCYTIICSVMLCVTAYQITTEPSFVQRLVFTEYLVFCVTQLFIYCWHSNDVLYASRDLSLAPYESIWWSRGVEHRKNLFILTAQFSKVVEFSVGPFTKLTVATFIQILKGAYSYYTLLSKSDE

>CsupOR16

MKDIFILKTYCQYIYRVGSGNFWYEERIVGNDRSLSYKIYRGLHFFLYGCLTILEIMAAIFGVFPSDEKRDAVTFAVSHTIVMIKLFSVISNKALIKQLNKNMTELCEEHEEQQLMAEKYKIVKINVAVYFIIVYVTAVFFAFEGLRKLFNGVHFVTVVTYYPAYEDNSALANSFRIFTTIILQVMLMSMIVTVDTFTMTYLIIFKYKFITLRHYFDSLRENYLKMSKNGNQEIAAEQLTNGFVKGIIMHQKLLKTAKNIDTAFGLVIALQLCQSSGSAVSLMLQLALTDQLTFLASIKAILFVLALFFLLGMFLCNAGEITYQASLLADAIFYIGWHEFAPQPPPKRSLRRLVLLAIAQAQQPLIMKSFKMIELTYGTFLQVVRGTYSVFALFYAQ

>CsupOR17

MSEEAKREIAESLSLNTFCMERIGISFESPKSNIANVRQKLMFVLSVWGICYHVFSEIAYICLTLTKSPRVEDVVPLFHTFGYGALSITKLFVLWYKKNVFKQLIFELAGIWPLPPLDDDGQSTKNKSLAALRMTHRWYFAVNVLGVWFYNLTPIGIYFYRKWQGLDVEMGYVWVSWYPFDKHMPYAHFAVYIFEMFAGQVSVFIMVSTDLLFSSMASHISLLLRLLHRRLEALATTNKTEHEQFDEISANIKLHQRLIRYCNDLESAFSLSNLVNVVLSSINICCVVFVIVLLEPFLNVSNKLFLGSALIQIGMLCWYADDIFQANLKVSAAAYNSGWYHTSPRCRRAILFVIQRAQKPIAFTAMGFTNITLVTYSAILTRSYSYFALLYTMYNKG

>CsupOR18

MLKRFFNSLEDPDRPLLGPNYWILKKLGLLLHFGKLGNIFTILIHNMGLLFVSTQYVDLYLIRSDLDLVLQNLKISMLSVVCVLKVNTFLLWCSKWKEVINYVTEADKYERNTDNPDNVQIVKKYTKYCRRITYNYWILVFTTAFITVVTPLLQYAYSSTFRESVKNGTEAFPHIFSSWVPINKNDFPFNWVTVAWHTYICVQGALVVMAYDTNVMVTMVFIGGKLDLLRERCKLMVGVNGVTTSNVDLAEKLRELHKTHVLILRYSRLFNSLLSPVMFFYMVMCTLMLCASAYQLTSATDTTQKLVMAEYLTFGIAQLFIFCWLSDDVLTKSEKVMLGPYESQWWLANVKQRKIILLMAGQLRIVPVFTAGPFTKLTLSTFLNILKGSYSYYTLLR

>CsupOR19

MPPAERHFLGSIILQWMSNRGFLMNNNCYTYWLNGVTMICCHVTFVLQCVAVIDARDDPERLMQCFCDASFGGMCVLKEFSLRKHRHCWISLLSKISHLEEEELEFETKSINQDEDAHNIVFSGHIKEINKKVKKLNNILSRLFSVTAIGYMLTPFIEYGIRKLIGAETAGLPHITQYWSPLKTNLLGYILAITLEILFVINNYAVHTTFDFSVFGIMIFISGQFRLLHDYSEGDCGSTLCISETREDLAHAKIKKCHEIHVKLIRITNKLSKLIKNILGVYFTLSTISLCAIAIRLSTETNIIKVMVLALYTLSAFLQLFLFCYFGDNLQNMSSIGMGKGPYAAAYWALSPRVRKELILLAKGMSRPCHLYAGPFTRLNLPSLLQVKKSYISSR

>CsupOR20

MSLASSSVSPHLTLLRRVGYCRLSPGVTGSSSTRYLHEIYRKFALAAILTYTSEQAIYAYQNRRDIEKLAPVLFLFLCHITCIVKKLVFHLNAPRIDQLIAELEDAAYNSQTPPHRAMLRSTASSALRLLRAYVGCAIFTCILWVMFPLVDRLQQQDIEFHFWIPVDYNRPLTFPLVLIYSYYVTTLVAVGNTTMDAFIATMLSQCKTQLSILRMNFEDLPLRAMEMAYNSTSYEAALSKLFLDCIHHYQKISETMNELLRVFGLAVLVQFAVGGWILCMAAYKLVSVNILSFDFVSTTLFLVCILTELFLYCYYGNEVYVESDLMVQSLYSMEWVHTPLAFKRSLLLTMERAKRPLRPAAGHLIPLSLDTFVTILKSSYSFYAVLRQTK

>CsupOR21

MFIKNPNKSVGCSLSAMMMFGFWLPKNLTEIERILYQCYGCFWFILILGCYAATQIVQLYFDLGILHLMISASFLLLTISTEIIKLLNIVYRRRMIKSMIDDFDQVLRSTDTEEARAIVKRCDRETTILLAIYAIVTIITMVGFAAAADKGMLPIRAWFPYDVTKHPNYEITYTYQILALSVDAFLNVSTDTLVSSLMAQVRCRFQLLGLSLRNLCQGIRINEPLLASDQVVIVKDRLRLCVEQHCATLEAAQKLQDYFSFPTFMQLSVSLLIICVTAYQMNAVIGKPMAFIGVAAYLLDMMLQVFLYCYQGSMLSEESIAIADAAYECPWYVMPVPLRRSLLIVMTRTRRVAKFTAEGLTTLSLSCFMGVIKTSYSMFSILQGME

>CsupOR23

MLDENGKKCFENSLRRTKLFLSLSGIRISATKWPKALEKLFDTYFYYFQVFWLYADVLGEISWLIEGVLNGSSFLELSLAVPCITVSCLATSKSIFLYLNRDVVVKVIDKLREIYPESDETLKYHSNADLSHDKELDIFEDDSNKSDINTDIERDIKNESVDFLNLVVKVQYYICSAVVVAFPLMPVSTMVFIYYSTGVLEYKYVYMVKYFFDPFKMALWPFVYFHQVMSTVIVAMNVFGSDTLFYAACIYIQMHFRILCHHYENAVSASSIQTRLNLKVAIRRHHELIDLVNRVEILYTKSTLFNIVTSSFLICLSGFIITMVEDIIVMVTFATFLFMNLSQISLLCYFGDMLMSSSTQIVNAIYNSLWYDADERVKK

>CsupOR24

MVHIYFQHPRLALLLTGLWTPPKEKKFRLLYIAYRICVISMQYAFVTCNLVNMVMLWGDLEQISDACYLFFSVATCSLKNTNYLLSQKKFLSLLDFMENEVFVSQSLVQDKIISAYAKKMGRIYLIYIVCGFCNCVEWALVPVFEKEGHKIFPFKIWMPMDAASAEIPEYLLGYVLQLFGIIFSVSTYLTMDIVAISLLMFVPVQLDIITCKIKEVQHVSILFDPKQRRDLIEHNSALLKDCIRRHQALLRYIEGVADIFDIHIFFQLSATVIVVCIIALQMTIEPPNTFHYYSTVNYLMAMLVQLCFYSWSGNEITERNNVLRDGLYECLWYEQDLSFKRTLWIAMEFMSRPLIFKAGNYIPLSRPTFVSVGLQTWL

>CsupOR26

YLRVCGFYRLDQSSSKSVKILHRIYRRLVLSFFILYTIQQLLKIYDARSNVDKVMGTMFLFLTNTDCIYKAVILWKKADHIEGILEVMKGPIFNKGEPGHRLFLQDTIRKTLLVFRIYNYMSLFTCFLWVLHPTVLHMQGKLIDLPIRLPFDPNTKYYTAALYVWIQTSWLAYCNTAADVFISILLEQCRTQVTILRYDLENVVQKSKEEATETHGNYGDILERKFREMLLHHKEIVKTAGEILDIFSGAVFYQFLVSGWILCTSAYKMVNMNPASIEYASMISYIICVSIQLYVYCYYGNEINYESRRLTDSAYAVDWLEIPVRQRKTLIIFMERIKQPIEPMAGTIIPLSNSTYVSILRSSYSFYAFLKNSSN

>CsupOR27

RDEESNKIYQCYRVVLLTLFKFVFFISFTLDLFFTPINVGLIVSQSLLYFSELAGLFKIFMVLFKRNSILEVFEILDSKEFLAEDEASQQIIQKSHIFFRKYRTACTLFYCTGVIIFFVPVIKYWTTTGAQLKLPSFQYYFLNDNVREKYSMYLFLYQHCCLVLVVLSNSASDTLICGLIMMATTQFEVLNWKMSHLALRPSEKHYDPKEEEIIIKDRLNKCLRHYDVILRYCKEVQETTGLSLFAQYTTGAITICISLSSFLIPMTHEDFVILVCYIAGMTVEIFYPAYLGAELTEKSENLIFSVYCGDWISRPESYKRSLRLMLERANKPVVITCLKMIELSLITFTSVMKSAYSFFTLLKCLLERQQ

>CsupOR28

VWIPPTNKSLLHKLYRSLMITLQYLFLIFQIIFIIQVWGDLETVSQAFYLLFTQACLCLKVSVFHVNVDKLRELLKQMNGEIFQPQSDRQKQILSKQASRIKALLLAFMVSSQFTCSLWAMKPLFDDVGSRKFPFDMWMPVSPEASPHYEIGYAIQVLTIGMSAYMYFGVDSVALSMLIFACAQCEIIMDKIMSVTSINYAMKNKERQKIFAKNRKKLIDCVKHHEALYAFTKLSEDAYHSYLFFQISGNVGIFCMTALRLTVVEWKSVQFFSMATYLYVMMGELFVCSWSGHELTSTSEMLHTAMYDCPWYEQDVRFKRDLCFAMMRMSRPLVFRTGHYVSLSRQTFIAILR

>CsupOR30

FIKGIQDRHSTDELVNTLFLFLTTVTAIVKQVAFTVRMKRIKQLFDTTDGELFSPKNSAHLELVEQNEKYMRRLHFLYICTVLSTCAYMSLYPLGNKAFGQDHQDINYRIYFPFDPRKSPIIEVFACSYFGIALTLQGYVNSIVDCTIVAFYGQCELQLKLLRYNLEHLTDLDDVDLQEGTINENTLSYIDDNLIKKRLVHCVKHHQKIIWFLSETQSISNEFVTLQLSVACWTICMSVYKLVTVDMFSGEFFLTIGYLNCMLMQFFMYCYHGSQVLVESEFIAESAYCSNWVDISPRSRRLLLIFMMCCTRPLVICAAKIVPINLESYLAVLKASYTLFTILHKK

>CsupOR33

VSCPHTTVELGTREQQQKDLTNTTFFMPMKQEMSLAGRSVAPHLLLLRWCGFCRLRPRHASAGHPSLLQRAGRRLHAAYCTFALAATSIYLMQECVYAYQEHNDMDKLARVMFLLLCHITSIIKQVVFYTDADRIDDMIAALDDRLFNPKESSAQALLQGTARSAKRLVRWYSSTAVATCVMWIIFPIMYYVSGHQVEFAFWITVDHSGPLMFTVLIIYSFYVTTLVGIANTTMDAFMATILYQCKTQLRILRLNFENLVETANKIVAANPQESYENVLMKLFLEYLEHYQQISETNNCLQDIFGTSILVQFGIGGWILCMAA

>CsupOR34

IYRKIINYISHVEQKQIADGDLEINQIVSEYISRSRRFCYLFWALPVFVDPIMFLQVFITSIRFGKSTGTYPKILDIYIPYSDYPPGYYFSLLIQTTIGFTMSAYIVSWDSLVCVTMIFFAAQLKITRLMCSRMIDPQNPQKSHDNIVECLKFYTTLIEKQRIFNKLISPVMFVNLFVTSINFGISIIEIARVEDDFATLASGCTYLGACLIQLLIFYWYSNEVTVESAKVSYGVFASDWPLISNKYQREVALLGVATAKTLVFEAGPFNEMTLSTFLGIIRASYSFYTLLNKTN

>CsupOR37

GVLYKSSGKLVMQIPFVAWYPFDETDIRYWPIAYFHQLWAGFFDASSVHGSDSFYSLSCVFLQIQFKTLQYDIEQIIPEETNINTPELYKSFRKRFMLIVMRHQELIRCVNVLEVIYSKSNLCIIAVSSIVICISAFNFTTSDDIIWRTIFLGFFIMWLLQVFSLCYYSNLISLSSTEVSNAIYNSYWYKANAEVMKDLLFVLRRGQKPCKLTAWGYSDLNLAVFSKIVSTSWSYFALLQTMYSE

>CsupOR38

HSRHVTYFFWALAFFSNFSIFSEPYQKNINSDNGDPVFKKIFDGYIPYSDYPPGYYISMFIQTVLGNIVSAYVVGWDTLICTIMIFFAGQLKVARLLCSRVINVQNPELCRKYIADCHRFHTTLVKNQKLFENLISPAMFVYLIVISVNLGVCIIEIAKIKNDTPTLISSCLFLLDCFIQLLLFYWHSNEVTEDSVLVSYGVFESDWYQAENKYQREVA

>CsupOrco

MMAKVKAQGLVSDLMPNIKLMQAAGHFLFNYHSDNSGMSTLLRKIYSSVHAILIVINFLCMAVNMAQYSDEVNELTANTITVLFFTHTVIKLLFFAVNSKSFYRTLAVWNQSNSHPLFTESDARYHQLALTKMRRLLYFICTVTVLAVVSWVTITFFGESVRLIANKETNETLTEPAPRLPLKAWYPFDAMSGTMYIIAFAYQVYWLLFSMAIANLMDVMFCSWLIFACEQLQHLKAIMK

PLMELSASLDTYRPNTAELFRASSTEKSEKVPDPVDLDIRGIYSTQQDFGMTLRGGGGRLQTFGQQNTNNPNGLSQKQEMLARSAIKYWVERHKHVVRLVASIGDTYGTALLFHMLVSTITLTLLAYQATKIGGINVYAFSTVGYLSYTLGQVFHFCIFGNRLIEESSSVMEAAYSCQWYDGSEEAKTFVQIVCQQCQKAMSISGAKFFTVSLDLFASVLGAVVTYFMVLVQLK

>CsupPR1

MDFELKENRFRTIEFLHNRIVRNFLMPLGGWPCEVFQEKTPLFSRFFRRFIPIQGSCMIYGELNYIIQNYSRLNFFLLGHIYVTMFLTGVMIIRAILPNKKEYNDLVNFFYGEFDLEHFKHKGSYYQKASEIVYKFSYYYSLVMAGMMIYGMLLYNALPLYHNYNAGVLHRSNRVANVTIEFSVYYSFPGFMPEDHFWFVTFTNLYLTYSCTVEICIFDLFMALFVFQMIGHIMILINNIKNIEMPKTCHNIEGFKTQTNVTVELYDYEENEILRNKIVEIVNHHRFILRFVSDVSFLLGPALASTYLCHLISCCLLLIECSQLDPDALAQYGPITVIMFYQLFQISVLFELLGAKSEKLIDAVYELPWECMDVRNQRLLCFLLQRVQTPVQVTALGLTKVGVTPMVAILKTTYSLFAFLRSTV

>CsupPR2

MIYGELNYIIQNYSRLNFFLLGHIYITMFLTFVMLVRAVLPNQQLYKDMVEFFYGKFDLEHFKHKGPYYQKASEMVYKISYYYGLVMTGMMICGMFLYNALPLYHNYNAGVLHKRNRVENATLEFSVYFIFPGFMPENHFWSVTFVNLYFTYSCSVEICIIDLFMALFVFHMVGHIMILLNNIENVEMPKTHYNIEGLKAQTSVTVALYNDEENEIMRSKIIEFINHHRFIVSFADDVSSLFGPVLASTYMFHLISCCLLLLECSQLDPDALAQYGPLTVIIFNQLFQISILFEFLGAKSEKLIDAVYGLPWQCMDVRNQRSVCFLLHRVQSPVQVTALGMTNVGVTPMVGILKTTFSFFTFLRSIV

>CsupPR3

MLESTPLKNLETRKFYLLAVFLLSTFQMYETVDLFSRLYTILMINMMVGGMILFNLTPLYSNYKNGVFSKNPPENVTYAYSVTYSVPGFNFYEHFTLTTILNWIMSYDVSVNVCVKDLYLSFLVFQIIGHIQILKYNLEHFPKPKNQATNRFDAEENKQIRKTIAECVDHHRLIVSFADDVSDFYGPMLALNYMYHLISCCLLLLECSQKEPDALARYGPLTVIIFGQLISVSVVFEIVETKVKSTKYGANCLTLTLSMVPFSK

>HarmOrco

MMTKVKAQGLVSDLMPNIKLMQMAGHFLFNYHSENAGMSNLLRKIYASTHAILIFIHYACMGINMAKYSDEVNELTANTITVLFFAHTIIKLAFFALNSKSFYRTLAVWNQSNSHPLFTESDARYHQIALTKMRRLLYFICGMTVLSVISWVTLTFFGESVRMVTNKETNETLTEVVPRLPLKAWYPFNAMSGTMYIVAFAFQVYWLLFSMAIANLMDVMFCSWLIFACEQLQHLKAIMKPLMELSASLDTYRPNTAELFRASSTEKSEKIPDTVDMDIRGIYSTQQDFGMTLRGAGGRLQNFGQQNPNPNGLTPKQEMLARSAIKYWVERHKHVVRLVASIGDTYGTALLFHMLVSTITLTLLAYQATKINGINVYAFSTIGYLSYTLGQVFHFCIFGNRLIEESSSVMEAAYSCQWYDGSEEAKTFVQIVCQQCQKAMSISGAKFFTVSLDLFASVLGAVVTYFMVLVQLK

>BmorOR1

MLLSFKDDSRSPDIQKPQNFQYMKILRFNLKIICAWPEKQLNEIRSLGHSIHRVILPIQSVVCLACGILYIHFHFNEIPFFILASTFITVMMNLVTCSRTALVMLFERYLVLTGRFITVMHLFNFQKNSDYAYKLCTFVNRMSHFYTLYVLFSMFMGLGLFNLLPLYNNYVSGAFSDPYGPNVTFFHSVYFAFPFDYSHNFRGYIIMALFNSYVSVTCSIGLVMFDLLMCLMVMHVWGHLKILSHNLINFPRPKASHVITTPNGPTNVETYTEEESKEVFARLRECIKHYGTVDDFANDMSETFGVILLVYYGFHQVSLCMLLLECSDLSTKAMLRYGPLTLIMIQQLIQISIIFELLGSVADRIPDAVYQLPWECMDVKNRRVVYGFLRRTQNPVRFKAMGMLDVGVQTMASILKTSISYFVMLRTVAT

>BmorOrco

MMTKVKTQGLVTDLMPCIRLLQAAGHFLFNYHADTSGMNMLLRKIYSSAHAVLIVVHYICMGINMAQYKDEVNELTANTITVLFFAHSIIKLAFFAFNSKSFYRTLAVWNQSNSHPLFTESDARYHQISLSKMRRLLYFICGMTVFSVISWVTLTFFGESVRMIASKETNETLTEPAPRLPLKAWYPFKTMSGGGYVFAFIYQIYFLLFSMALANLLDVIFCSWLIFACEQLQHLKAIMKPLMELSAALDTYRPNTAELFRVSSTDKTEKVPDAVDMDIRGIYSTQQDFGMTLRGAGGKLQNFNAENNPNGLTAKQEMLARSAIKYWVERHKHVVRLVASIGDTYGTALLFHMLVSTITLTLLAYQATKINGINVYAFSTIGYLVYTLGQVFHFCIFGNRLIEESSSVMEAAYSCQWYDGSEEAKTFVQIVCQQCQKAMTISGAKFFNVSLDLFASVLGAVVTYFMVLIQLK

>BmorOR3

MIFVDDAVIGIKDPREYRHLRVLRTSLRLLGAWPGHYLGEETGSKYECAPMFLLMFIKIACLYLTIVYLRNNADVLGFFELGHVYLTIFMTFVTLSRGFSLTWNPNYHKVVKKFITEMHLLYFKDNSEYAMKTHRRVHKISHFYTVFLKVQMIAGLTLFNVIPMYNNYRQGNYASDRPANITYDLSIYYETFDILNTPNGYIFICVFNWFASYICCSFFCSFDLILSLMISTVSGHFRILIHNLLTFPLPEAITASKKFVDKHRCNGNRSEFVLEEAKLYSPAEMWQVTDRLRQCIDYHRKLVEFTGDISEAFGPMLFVYYLFHQVSGCLLLLECSQLNTAALVRYGVLTVVLYQQLIQLSVIVESVGTVTGRLKDAVYEVPWEYMDTSNRKTVAIFLMNVQEPLHVNALGLAKVGVQSMAAILKTSFSYFTFLRTVSE

>BmorOR4

MFKIIKNIIVENDALKQVEKPQEFQYMKWVQYHLKYIDGWPNMDMNKKNVSKIRFHKRHLLVVEQTITFLSQMFYIVKNYGKLSFFEIGHSYITALMTIVIFSRSVVTALGRYRKIARYFVSSLHLYHYKDISEYALQTHLLVHRLSHYYTVYLISLVVTGMLLFNITPLYNNISSGVFNSPRPENMTFQHAVYLGLPFDYTTDIKGYFVVFILNWHLSHIAASYFCTFDLFLSLLILHLWGHLRIILNNLKTFPKPYTNNSMYTEEENQVVLLKLQECIRYHNFIISFTVMMSNVYDVVIIVYYLFHQVTGCLLLLQCSTLDWESLSRYGPLTLIIFQQLIQVSMIFEILGFLSDKLPNAVYSIPWEAMNVTNRKLVQVLLQKSQKPIQFKAMNMMSVGVQTMASIIKTSISYFIMLRTIARD

>BmorOR6

MKEEYYLQHPRTQLFYKVLAHVSTIESTIDLTWWGYTFPKYVGWFYHLQCNVVRLFGKCVVVSQILFIILNYQTIDKSVFIIAITITPLGALVGIKAESAKAECYVNLMKNFMDKVHIHSIYRKNENNEFVKKKVIQIERVSRFTAYFLVILIAINCLSWMLKPTLHNIKHFEEIMNKSMEFQYYIYFWTPLDYKYNLRDYIIIHTLCIYLGATAVTVIVTFDIFNFIAVFHVVAHIQILKNNVKSNWSDDFNESEKKGYLVSILEYHAYIIRIFGEVQSAFGLNVASNYLQNLIEDGLFLYQIMNGEKENVLMYGLMIILYLGGLIFLSIVLEEIRRQNYDLCEYVYALPWEGMSLENQKIFVVFLQRTQPDLEFETVCGMKAGVKPAFSIVKSMFSYYVMINSRF

>BmorOR7

MLLYHPNTQVEEKVNNVEEFTYMKFLKSFCKIMDFWPEREEKNSKTRIFRLRYILVLQFCFTLVAGVLYLKNNFGKKTFYDLGHTIITVVMNVVSVSRLILRCFKKYDVVGQQFINKIHLYHFRNDSEYSMKTYKAVHKISNNMTYIFSFSIFVCVVTFNLNPVFNNIGSGAYKNPRPDNVTLQQCVYYALPFDYTGDFKWYMLVAIFNVQKTFFCTSLFILFDLLLSMMIIHLWGHIRIFIHNLNHIPAPRNSLEYTREERQEVDNTLKKCIQHHTLIIGFVRIMSETYGLAVLIYYAFQQVVGCLLLLQCSRLDLKTITRFGFLTTMVNQQLIQISVIFELLGYMNDKLQEAVYCVPWEYMDTSHRKMVYMMFRQSQIPLQLKAMNMLSIGVKTMASILKTSVTYYLMLKTITANEA

>BmorOR8

MSLSTRCLLKDFCKYVYYAGAGNFWYEDIYKETVPYKMYVVISFFTYTVMIFLENLAALFGKLPEVEKNSAVMFAAIHNIVLTKMFLLLYHKRSISKLNCEMAAVGENLEEASIMRRQFRKMRLGTALYFISVYLSLVAYGVESARRTIVEGAPFYTVVTYLPDYDNTTVLASFLRIFFYITWLYMMLPMMSADCMPIAHLITMTYKFVTLCRHFDQIREKFQINVKIMAKTEATEILKLGFIEGIKMHQKLMYLADEIHRVFGIIMALQVCESSAVAVLLLLRLALSPHLDLTNAFMTYTFVCSLFLLLALNLWNAGELTYQASLLSNAMFYSGWYFCDFEKDWCRDIRRLVLIGCAQAQKPLILKAFGVLDLSYETFVSVARMTYSVFAVFYKRGD

>BmorOR9

MVARRPLQFHQGRNVDNVEDFKYVKWLRNHLKTVDAWPVHSKSKRKIQKRYVLPIFSAACFISQTVYLKNGIGTLSFVVLVHSYICFLINGSCLCRGILIATERYKRLATCYLKTVHLFHHKNRSEHAMKIHVIVHRLSHYYTIYLISLVFVGMVLFNFMPIYNNINSGAFKSPRPENVTFQHAMYLALPFDYTTNIKGYFVVFILNWYISLVTTSHFCTFDLFISLMIIHLWGHIKILMCSLEDIEGFVPGSSFKFTIEQNRKIYLILQECIRHHQFTIDFTNEMSSTFGLVILFYYFFYQVSGCLLLLACSQMDIESLSRFGPMTFILFQQLIQLSIVFELISSLSENLPNAVYNVPWEFMDKNNRKMIQVLLLQSQKLIQFKATSMMNVGVQAMATILKTSVSYFIMLRTMYQEH

>BmorOR10

MRTNAKSFLFVPSKVLTLCGVWPVEKTSIFSLIYRSIMLSSQFCFLVFNGIYIGLMWGDLKAVSDALYMFFTQTTCCSKAIGFYFNFMKIKRIVASMDDVLFTAMSIEDQATIFSHSRTVNKLYKGVLGFTGFTLVQWTVLSLIGSGRTLPFNEMWVPTDISKSPNYEITFVVELWMMVISAALFMSVDTITVATMMFSCAQLDIIMKKTQQIQEIPLSPDLSSRNRSELHEKNNGILIDCIKQHQAIVRFSELCEGTFQVHSFFHLGGIVFMICVIGFRMAGESPVSAQFWAALSYLVIILGQLYLYCWCANELTTKSEQLRDKLYLTPWYDQDVKFKRNLCIAMECMAKALTFRAGSYIPLSRAMFVSILRSSYSYFAFLNQANEQ

>BmorOR11

MDEHSHFETSLNKIKVLFKYSGMNLENTVTNTYEFLNHRWVYILNHAWTLAAVTFICIGISNGQNFIEMTCIAPCVAMTVLAVSKSFFHYINENAVKSLLENLIELERTDFERTKSVQRTEIVATEKQLLNMVINVLYVLNCSMILVFDMTPLIIIAIKYWTTNKFVRLLPYLDIFVFVPYKFEYWVMAYILQIWAECIVLLFIGAADCLFFTCCTYIRIHFRLLQYDFERLTSSRRESDGLRDDEDFRETYTNLVKRHQGLIESSSILEMIYSKSTLSNFVLSSLVICLSAFNVTVVNDVTIVMTYLIFLAMSLMQVYFLCFFDMLMSASEEVGNAVYNCSWYTEKASTGKDLLFTITRAQKPCELTAAHFAYVNLKAFMRVSFTSASITTLPTI

>BmorOR12

MTRITDVFSLNFIFWKFLGLWGKSAPSKYNMAYTVFYLFASLFVYDIFLTLNLIHTPRKLETLVRETMFYFNHLVAVTKILMMFIMRKKILVIFDLLDCEEFKPNDENSQEIMKRKTDFYYIYWRIVAVTSNLSCFMLVIGPLIKMLIWKIELGLPVCKFYFMSDELRNKYFVIWYIYQSFGIYNQMVNNLNLDTFNCGMLWMAVGQLQILKTKFVNLKLNDFENGLDLKSRDDMQIERLRKYLTHYEIILKYCAIVQDILNITIFVQLGMSSIVICVGLCGFVAMPSNTETAIFMFSYLTTMTMQIFVPSWMGTQISFECGELMSAAYSCEWIPRSKLFKRSLILFVERAKTPVRITGLKIFTLSLDTFTSIMKTTYSFFTLIRQLQVDEVN

>BmorOR13

MAPKQIDCFEINWKFWKFLGIWSENKPHRYYKYYSKIFITFFVILYDVLYTINFYFVPRQLDLIIGEMLFYLTELSVLSKVFTFIIMRHKLKIIFEILESDAFQTDTEEELKILHRAKVFIKRYWKIVALVSITANLTHISSPLLKNLIFKVELVLPVCSYSFLSESFLKTFEYPLYFYQIVGIHFHMLYNLNIDTYFLGLMILIIAQLDILNVKFRNLKSGKDHTQLNESIMGLNKNLDHYNEIERFCSLVQNIFSFTLFVQFSMASCIICVCLFSFTLSVPVEYYIFLATYMFIMIIQIMVPCWFGSRIMDKSILLSSAIYNCDWTSNSKDFKINMRLFVERANKPLSITGGKMFSLSLATFTSIMNSAYSFFTLLRYIQTRE

>BmorOR14

MSNYIFKPFHETYRIITFTMIAAMIYPNPATEKRRLIYIGLMLLSVIPLAFMIVTEMYEFFMASDLNNTIRHSTVIGPFIGGFVKVALMYYKRRQANELVSEINRDHLAYNGLKGEDREIAASSIRNCQIYCELGWTLIVMSCGLSFPVIAILLKIHSFTFKLDSTKHMIHDINNPFTDDPEDRFESPFFEIMFVYTFFSSFIYIINYVGYDGFFGLCINHACLKMKLYCRALEDAMRSDSRRHEKIVAVIEEQRRTYEYIALIQDTFNIWLGLIYVATMIQMCTCMYHIVQSFNIDVRYIIFVISIIHIYLPCRYAANLKCMAAETPTLIYCCGWESVSDLRIKRMMPFMVARSQVIVEITAFNMFAFDMELFVWIMKTSYSMFTLMRS

>BmorOR15

MMTLVYQTDIFKPNVFFWKMFGIWADRKSSKTYKYYSFVFLFITLIMYNSLLAINLLYTPLKIELLIREVIFCFTEITVTTKVLMILFKRNKILDAFDLLNKNEFRGNSEESSAIIQKNNSAYKTYWKLYAILSNFAYSSQVLGPLIVKLIWKTKLELPICNYYFLNEELRHDFFSGWYIYQSFGMYGHMMYNVNIDTFISGLLMMAVTQLKIIQTKLLSLKLNPRERKMDRGLMNITEVLKLNEILKHYELVLKYCSTVQSILDVAMFVQFGVASAIICVAMCGLIMVRSSTETLLFMVTYLFAMTLQIFVPAWMGTQLHFQSQELVFAAYNSEWIPRCQSFKRSIIIFVERAKIPITITGLKMFPLSLATFTSIMKTAYSFFTLIRNMQTLQEE

>BmorOR16

MSFNSEDLYLNRAKFVMKYLGVWVPPENENFARKFYKIFMMSLQHLFLFFQIIYIVEVWGDLEAVSQASYLLFTQACLCFKITVFQINMNKLKELLKQMNGYVFQPKNINQQNIIKVQATRIKRLLFAFMISSQLTCGMWALKPLFDDVGSRKFPFDMWMPVSPERSPHYHLGYSFQLVTICMSAYMYFGVDSVAFSSVIFGCAQIGVIKDKIMSIKPLGIYRNHKTYTKISRYNRKTLIECVKHHQAVISFTELVEDTYNSYLLFQLVGSVGIICMSALRILVVDWRSVQFFSILCYLSVMISQLFVCCWCGHELSATSEELHTILYNCAWYDQDVKFKRDLNFMMARARRPILLRAGYYISLSRQSFVSILRMSYSYFAVLDQTNK

>BmorOR17

MREDKMEINNSQKFYTKMIFRYLYSVGLGDWWYQHEDRSDSHRKLYCLWAVISNAYIFLNICNELLANFRKDLTDVEKNDAIQFSFAHPLIFAKIASFFFNRKKIREVFGRLLEENRSVYSCGELEKESMKQIKRYSLAFIGVSYMTLVMSTIDGLRAHFKEGIPIRTEVTYYPSPSNSGVIVNILRFLVEFHWWYIVSVMVAIDSLAVASFVFVTFKFKLLQRYFKDMGLTVRRDQSNMTDEALADKFRRDFIVGVKLHENALWCAENVQKAFGWVYSVQVFETVALLVMCLVKLVTTNHNMIFLLANFAFMLCVIILNGSYMMPAGDVTYEASEVPTSIFLCGWELVRQTDLRFLVVVAIQRSQVPVIMKAFGIMTLSYSNFIAVSLFKFYVQFQINLF

>BmorOR21

MNKNHYILKTYCDKIFLVGSGNFWYQKTESRNDKTLLYKIYSCVLFFTYGFMTVLEIMAAMMGDFPEDEKRDSVTFATSHTVVMIKFISIIKNKELLKTLNRKMMMICEAHEEQTLMDEMYRTVKINVVAYCVAVYGSATFYVFEGLRKFYNGSHFVTIVTYYPSNDDDTLAATIVRIATTLVLLMMLLTMIISVDTYTMAYLIMYKYKFITLRHYFKRLRENVDELVAAGKARLAAEKLAQGLVEGIKMHNELLSLSKDIDKAFGTVMALQLCQSSGSAVSLLLQIALSDQLTFTMGMKIFFFLAAMYLLLALFLCNAGEITYQVCTSIV

>BmorOR22

MNKNHYILKTYCDKIFLVGSGNFWHQKTESRNDKTLLYKIYSCVLFFTYGFMTVLEIMAATMGDFPEDEKRDSVTFATSHTVVMIKFISIIKNKELLKTLNRKMMMICEAHEEQTLMDEMYRTVKINVVAYCVAVYGSATFYVFEGLRKFYNGSHFVTIVTYYPSNDDDTMLASIVRIATTLVLLMMLLSMIISVDTYTMAYLIMYKYKFITLRHYFKRLRENVDELVAAGKARLAAEKLAQGLVEGIKMHNELLSLSKDIHKAFGTVMALQLCQSSGSAVSLLLQIALSDQLTFTMGMKIFFFLAAMYLLLALFLCNAGEITYQASLLSDEIFYCGWHKCNSPVLSTQRNIRDIVLIAILRAQSPLVMKAFKMVELTYATFILVVRSTYSVFALFYAQNK

>BmorOR23

MRAKTEFEKTIKLTKTALFLSGINIFLGEWNHWTRTFVDSIAYYLNIVGLYFVLIGEMYWLIDGTITGKSFVELSLIVPCLTISVLATAKVHYLYHNKESLLDVVDKLREIYPDEIEETANDNDQCLNDKKETVYDNDVTEVGIVNEANELLKFVNFLLSTVSFVVTMTFCTMPLFGMAGEFMETGKFVVLYPFAVKYPFDVYNTSFWVIVYVNQFWATIIVCTNIFGVDTLFYALCSYIGMNFRLLSYKFEHLEIKRNDRIINEIIVLIKRHQELIELVNKTQSLYSLSTLFNIVTSSLLICLSGFNITILSRSWSYFALLKTIYS

>BmorOR24

MPEELFLDRSIKKIESYFRWMGINIRSGDNNNKKDVFKIRCIYFINFVLLNTDVLGAIFWFRSGLEQGKTFTEVTYNAPCLTFSFLANFKMLSLIFYEKTVHELIAALQKLEIKHFLRQNCAEELKMLKDEKNFLHAVFKGSKIVNYASILTFGCSPLVLIASNYYKTGRMDYLLPLIVLYPFDVDNITVWPIIYVRQIWSVITAVIGVCATDYLFYTFCVYISTQFRLLGHSIERVVPNNGLSVRTRLNGNLRMKFVENLKWHQELIRAASLLEQIYTKSTLYNFVTSSVIICLTGFNVAVVEDFAVILSFLFFLFMSLLQIILLCFFGDKLMKSSTNISDAVYNSKWYLTEKNVGKVLLMVQIRSQRACRLTAYGFAEVNLRAFMKILSTAWSYFALLQSLYSSHE

>BmorOR25

MFEKALRSANFYMRVIGIPTDIRDGNRTLMERLRNRWFYCINFLWLNTDVAGEITWFVKGLLSGSSTLIENTYLIPCLTLCILGNVKTFFTIKYANHIIDLVAILKDLEIKNNAARKNETEIVKERLKFLTTSNKFLLFVIGTGIIAFGIGPLMLTASIYFSSGDMKLKLPFLIWYPFDSSDIRYWPFVYVHQVWSACIACCAVYGPDCFYFTSCTFIHIHFIHLQNDITNVIVESSRARRNGLYRGCHQAFLELTNRHKDLIRCVNLLEIIYSKSTLVNVVSSSLLICVTGFNVMAIDFLPLIAPFTSFLALGLVQTYLLCYYGDTIMCSSTEVSDAVYNSTWYGTNISQMRDYLFVMKRAQKPCKLTAYGFSDVNLRTFSRILSTAWSYFALLITIYRGNGQQ

>BmorOR26

SLSGSSVFTHLFLLRCCGFCRLSRSSTARRGLSVAHEVYRALTLTLTVVYLLQECVYAYQERTDMDKLSRVMFLLLCHITSVAKQLVFYLDADRIDYLIATLDDPSYNEMSHQRLLVDASRWASRFVWAYSGCAVVTCTLWIVFPIIYHVQGQTVEFPFWIQIDYTKSSMFVVVLLYSYYVTTLVGIANTTMDAFMATILGQCKTQFTILRIKFETLPTRAKQALRCDSEQNYDEVLMRLFHDCLKHYQKIVSAILIQFGIGAWILCMAAYKIVNLSVLSIEFASMILFISCILTELFLYCYYGNEVSTESERLVTSIYSMEWVGARLGFQRGLLVLLERARRPVRPAAGLVIPLSLQTFLKIIKSSYTFYAVLRQTK

>BmorOR27

MPSSFFLPNLENPDYPSLGPTLKGLKYWGMWQSGGIKRILYNSIHAFATFFVITQYVELWIIRNNVELALRNLSVTMLSTVCVVKAGTFVCWQKYWSGIIGFVSNLEKEQLSKNDAATQAAIVKYIKYSRRVTYFYWSLVTATVFTVILAPLVGFLSSPERELIANGTLPYPEIMSSWVPFDRSRGFGYWVTALVHTLICFYGGGVVANYDSNAVVLMSFFAGQMKLLSINCSRLFDDGNEVISNNEAMKRIKECHYHHVFSTIFNSLMSPVLFLYVIICSLMLCASAVQLTTDGTSNMQRIWISEYLMALIAQLFLYCWHSNQVLYMALEDRLGGLFEACLESGRFPSKWKTGRLVLLRKDGRPADSPAGYRPIVLLDEAGKMLERIVAARIVRHLTETAPDLSAE

>BmorOR28

MHTLALVFALLYPSNCNIIKRAIGITLIIALSGGQLFWCMTYTFNVCVLILNYSGFDGSFCIASIRLCMKLKLVVYKVQKAFAESKSVSELKHQLNDAIKDNLDALKFHEQIQNVFFIALVGRRAYGPPDGEWLPSPMDFSNTRGRTKPLSTVYEPWLFLIFLLTFLII

>BmorOR29

MFDFLQNLEDSERPLLGPNFWLINKTGLLLPKTNFGKLAYILVHEIVTFFVVTQYVELYVIRSDLDLVLTNLKISMLSIVCIVKVNTFVFWQTSWREVLEYVNEADKFERNQTDETRGKMIETYTKYCRRLTYFYWSLVFTTFLTTTNTPLMRYWSSPIFRENLRNGTEDFPHIFSSWMPFDKNHSPGSYCTIVWHVLLCAYGAAIMAAYDTCIVVIMVFFGEKLNLLRERCKKMLANDLYNHAFVIGQLHDIHVQLIKQSRLFNSLLSPVMFLYILMCSLMLCASAYQLTSATSTAQKLLMAEYLIFGIAQLFVFCWHGNDVLFKNANVSLGPYESNWWSSSPRVRADVLLLCGQLRVRHVFTAGPFADLTLSTFIKILKGAYSYYTLLRK

>BmorOR30

MSVSNLKFEVLFKPTTMSLHMNRSHPSIKRNKIWLLQFISLMTLTAFCATGLITSLLFHDLKFGKYMEASKNGTIAMLSFTTTFKYSLLLYLQKSLNRLIAKIDMDYEIAKGLTPQEKVTVLNYAKKGVIVSKFWLFTAFAITFCFPLKAFIIMGYRFIIKDEFRLEPMFDMTYPEPIESYKTSFPVYFILFVVCFLFGCYASSLYVAFDPLVPIFVLHACGQLDLLSVRITKLFSDTKNPRIIAKELKVIIIKLQELYSFVNFIKVNFSILYEYNMKITTISMPLSAFQVVESLRRGEFNIEFTYFFFGCILHFFMPCYYSNLLMERSENFRFAIYSCGWENHNDKNIRQMLLFMLTRAAEPLGIATVFTNISLDTFAEVNTFDTVLLA

>BmorOR32

MTTRHAEPCAEAPRLSPAAGGMVGLAPVPQPSSNEMLVQERGRPPGLEGEYVANRPFRKSLRGSQEEPRANGKSENVRFLINSHILHCGLRFNETNCHTHYIAKVAIFCFIVTYMLQVMELYWSKGDQEKLFECFSILSFCGMGVMKLVILRVYHQRWRFLLNQVSILENRHLDPGPLSYDSDNDNDDNEIVTFITKYTDKFKRTSSILIKMYASTLVIYVLSPFVEYIFRQFRGDLNIAYPHILPAWTPLDEFSVTGYLIMVSFETVACIYCVFVHVAFDLTCVGLMIFACGQFYLLRYRSERIGGKGRICRLLKSTEVRAHYRIVFCHGIHVLLVQLIEELDRLIKHILGVYFFLATLTLCSVAVRLKTEDMSITQLVNLLQYMCGTLTQLFLYCKYGDSVYNEADEPYGLPDACLESGRFPKQWKTGRLVLLRKERRPADSPAGYRPIVLLDEAGKLLERVVAARIVQHLTGVGPDLSAEQFGFREGRSTIDAVMRVRALSDEAVGRGGVALAVSLDIANAFNTLSWSVIAGALQYHGVPAYLRRLIGSYLEDRSVVCTGHGGTVLRFPVQRGVPQGSVL

>BmorOR36

MVFNSKKNIISLFSLLEDSRHPSVGPHLRLLSLTGIWYPNSKTNITLLKRACFYVIVLFFVSQYLKCIIKFKIDSLQLILEYAPFHMGIVKTCFFQKDYNVWQDLVSFISKTERDQIAKKDPKSIKTIQSYISRNRKITYSFWALAFIANIGVFSKPYQNNQSDVNGTVTYNHLFDGYTPFSEEPPGYYFSMGIETILGHVVSFYVLGWDTLVVSIMIFFAGQMQMSRLQCSRMINGSPERTHKNIIKCHKFHTDLIKYQKQFNSLISPVMFVYLFVSSINLSVCIVQIAEIEDDFATVLSSFIFLLACLIQLLLFYWHSNEVTVQSELVSYSTFESNWTSTQNKLQKEVALLGLTTSKTLVFTAGSFNHMTLATFISIIRASYSFYALLNSTKY

>BmorOR41

MMGNSTDLFLDRTKSILNFFAMWRSFEKPIPLKVYMAFIMTTQYLFLIFEIIYIVNVWGDMAEVSEASILLFTQASVCYKITSFISKTNNFVILLGLIESEIFSAQTELHEKILILKARKIKRLCMFFLVNAVTTCSLWAVIPLLDISSKMLPFKIWMPASTGESPHYELGYLYQMITIYISAFLFIGVDSVPLSMIMFGCAQLEIIMDKIGKVKSRPLDQQPMQRQAVLNSNYELLVECVRRYQSVVRFIELTEKTYHANIFFQLSGSVLIICNIGFRIAIVDSNSLQFYSMLTYLVTMLSQLFQYCWCGHELTIRGEELRETLYQSPWHEQDIRFRKVLIITMERMKRPIIFKAGHYIPLSRPTFVAILRCSYSYFAVLNRVRNE

>BmorOR42

MDIPKFEELLKQIKMNFWLMGIPFDNPKIQIRYYVLLLPLSLMLIEEIAFFGSRMSSENFLELTQLAPCICIGVLSVLKILALTAKRQKIYELTQNLECLHKIILNDTRKTELVRKNLVLIKFITKYFFVLNAVLIFVYNFSSPVIIAYNYIVSNEVQFVLPYAVLLPFKTDSWIPWLIVYVYSIFCGFTCVLYYATVDVLYCVMTSLVCNNFSLISFKLQKVNRNTAHLLKEVVKEQQYVLKLAEDLENIFTAPNLFNVLIGSVEICALGFNLMIGDLTQIPGCILFLSSVLLQILIMSVFGENLISESSRIAEAAFLCKWYEMDQKSKKTILTIMIRSHKPKKLTAYKFSVISYGSFSKIISTSWSYFTILRTMYTPPGTKFQDDL

>BmorOR44

MYTYFKVLVFWLNKDKVISLQKILHCKEFKPKEPEHKEIIRKSIRKARFVMTSYATMCVGAVSVGIILPLTENFDILPTNVEYPFFDVYKNPTYAYLYLHHIYYKPATCIIDGVMDTILAAFVASAIGQIEILAFNLRNFDVLAERRRKRAISGNKYIGKYTNLYFTKRILKECILLHNSIIRYVSVIESAFSLASALQFMLSVMVLCLIGIQFLSIENPTSHPMQMVWMAIYLTCMLIEVFILCWFGNELIWKSNDLRQAAFDGPWRNLNRKTCMFIIIFMERCKRPMRLSAGKIFTLSLDTYTVLINWAYKAFAVMRNMKK

>BmorOR47

MKLVFDNFISALKVTLNWSRYIGIFIPDELTGRRQKLLVQAYSVFMYYLFIGFFITTQIISFILVWGDLNLMTDVGLVLGTNLALSAKIAVFFFKREDLANILKKNDDTLRSETRAEGKKIISETSCESARVGTTTLPISAVVTILETLELISQASLLITVDIIMLSMIAVCRCRVKLVGLSLQTICDDLPCNVKNKLTSDEEVIVAKRIREYVIEHQAILDCISELQNHFSPALLVQLLTSVVIICVTAYQLAVEKSSDLLRKFTMASFLFAMSTEMFTFGYQGGHLSHDSMEVATAAYSCPWYTFPTSLKRSLLVIMIRAQQPALLTAGGFTTLSLETFVTIMKASYSFFTVLQEATD

>BmorOR51

MDCTIVAFYSQAKTQIKMLRYDLEQLGKIDNIETKFTENIFERSSHIWKALKDEKIKIHSKLVFCVEHYRQIVWFVKEVESIFGEAMTVQFFVMAWVICMTVYKIVGLSIYSAEFVSMGVYLGCMLAQLFIYCYYGTQLKVESESVNTSLYCSNWLSSLPKVRRQMLIMMQYCSKPLTPRTAYVIPMSLETYISVLKSSYSLFTLLNQKH

>BmorOR53

MALKKMLALTKGLEDPTHPLLGPTLKALSVFGLWQTGSQKSTVIYNTFHFLTFLFVITEYIDLYTVRKELSKMLNNLSVTVLSTICMIKTLSYVCRQSHLKVLVREISELELELMKTTDKNIVKRLRQYTVYTRAVTYVYWFLVVGINVVLLTSPLLKYASSEIYRSEIKNGTEPPPLILCSWFPFDSARMPGYFWATMVHIIMSIQGCGVVATYDMNAVAVMSYLKGQTSILKDKCKAIFDETASSRDVLNRIRDCHRHHNILLRHYYMFNSLLSPIMFVYMLICSFTICCSIIQLDSSETTISQRIWIIQYSIGQISQLFLYCWHSNEFAAKVKKKHFPLFPINLF

>BmorOR56

MKLLEKLEDPDRPLLGPNVKALKFWGLLLPESRSKKYFYLFMHFAVTVFTATEYIDVWFVKSDLALLLNNLKITMLATVSVLKVTTFLLWQNAWRDLIGYVSRADLEQRATSDSRKLALINGFTGYCRKITYYYWFLMYTTVAIVTVQPIFKFFSSAAYRLDVQSGNGTYLQVVSSWIPWDKNTLPGYLLASIYQTYAAIYGGGWITSFDTNAIVIMVFFRAELELLRIDCAALFDDEKSFGDMAFMRRLKECHRRHTELVKHSRLFDSCLSPIMLLYMFVCSVMLCVTAYQITIETNPMERFLMTEYLVFGVAQLFMYCWHSNDVLYASQDLSRGPYESAWWSRDVKYRKNLYILVAQFNKVIVFSAGPFTKLTVATFIRILKGAYSYYTLLSQSQMNKT

>BmorOR57

MPSLIKNRIFGLTLTLNTLSWAGLILRDDYTKTQRIIMKVYGGLVFLYLFVFTAYVQIADLVVIWGNIDFMTETSLILFMQLAVSAKVLTLMLKSKKIMEVTNEADAILISEKKVEGQRIIASIDKNTTLFLKYYGFFVAFTIICWFMGENTSTFFIRSKYPFNELKSPGREFAFVHQCIVVIFTGSFDFNVDIIIISLVAVCRCRLKLVALSLRNLCLDIPMNKRNLITSDEEKVITERLRNIISQHKRALDAAEAIKHYLSGALLVQLMVSIVVICTTAYQLAVKKSTTMQSLTMAGYLFGTSLEVFLFCYQGEFLRESSEEIADAAYECPWYTLTRPLKKTLLIIMTRAQRPATLTAGGFVTLDITEYMAVSLISNT

>BmorOR58

KLVFDNFIFALKVTLNWCRYFGIFIPDELTGRRQKLLVQAYSVFMFMLFIGFFIITQIILFILVWGDLSLMTDVGLVLGTNLALSAKIAVFFFKREELASILKKNDDTLRFETREEGKKIISEYPCDTKRSPAYEIIMIHQTIAVAVIASLAITADLLMLSMIAVCRCRVKLVGLYLQTICDDLPCNVKNKLTSDEEVIVAKRIREYVIEHQAVLDCISELQNHFSPALLVQLLTSVVIICVTAYQLAVEKSSDMLRKFTMASFLFGMSTEMFMFGYQGGHLSHDSMEVATAAYSCPWYTFPTSLKRSLLVIMIRAQQPALLTAGGFTTLSLETFVTVS

>BmorOR59

MDTNPSAAGDSVAPHLRRLRQVGFCQLDPTSQSRRPILALMHRVYHRLVLAATVLYIFEQLTYAYQARNDMERLSRVLFLMLCHLTCIAKQFVFHSDADKINQLVVGLDDALCNQPVETHRLLLLETSRRAARLLMLYSGCAVSTCILWAVFPLLDQLRGRTVEFAFWIPIDYRHNAFQFAVVLAYAFYSTSLVAVANTTMDAFIATVLYQCTTQLRILRMNFESLPERAYALSRKTRQDYHTVTHELLVDCLLHYKKITETCNLLEQIFGKAILVQFGVGGWILCMAAYQIVDMEILSIEFASTALFMGCILTELFLYCYYGNEVTVQSGLVSESVYAMSWLSLCPRERRALVVVLERARRPLRPAAGRVVPLTLNTYLKILKSSYSFYAVLRQTK

>BmorOR60

MVRPCRYFAIHFILLRFLGLGWWHHPHENETRNYPGLYLYYSILTQLVWVVGLVGLETIDPFVGEKDMDRFMFSLSFVITHDLTLIKLYIFYFRNVEIQDIVRTIEIDLYRYYQNDDKIRATIRISRIFTAAFLFFGWVTIGNANIYGIVQDLRWKDIVKNLNETTSKPLRTLPQPIFIPWPYQEDKHYILTFILETMGLLWTGHIVMTIDTFIASVILHMSTQFAILREAIVTAYDRTMIALSEGALQSGVLCENSNGNEENNQIFLESFYSKEHIESVLESTLLSCIRQHQLLIGCVEKFSKTYSYGFMTQLLSSMAGICVVMVQVSQGASSFKSVRLVTSLAFFFAMVIQLAIQCFTGNELTIQAERIADAVMESKWEKMPVRLRRLLLVTMMRAQRPLHLTAAGFAYIDNTCFLSILKAAYSYYAVLSQKQG

>BmorOR61

MARITDVFRLNFIFWKFLGIWGKSAPSKYNMAYTALYLSASLFVYDIFLTLNLIHTPRKLETLLRETMFYFNHLVAMTKILKMFIRRKKILVIFDLLDCEEFKPSDEDSQEIMKRKNEFYYIYWRIVAVTSNLSCFMQVVGPLIKMLIWKSELGLPVCKYYFMSDEFRNKYFVIWYIYQSFGIYNQMVNNLNLDTFNCGMLWMAVGQLQILKTKFVNFKLNDIENSLDLKTRDDMQTERLRKYLTHYEIILKYCATVQDILNITIFVQLGMSSIVICVGLCGFVAMPSNTETAIFMSSYLITMTMQIFVPSWMGTQISFECGELMSAAYCCEWIPRSKLFKRSLILFVERAKTPVRITGLKIFTLSLDTFTSIMKTTYSFFTLIRQLQVDEVN

>BmorOR63

MKLWIRNANFTISLSLTLLRCLGFWSPDGLAGNKRLLYNCYSFVFFMFLLGIYILIQVVDMIKIWGDLPLMTGTAFLLFTNFAHATKVINIVIRKNRIQRVIQQANAVLMGVQSEEARRIVKSCDFETSIQLCLYFLLTFVTTVGWATSAEKHQLPLRAWYPYDTSKSPAYELTYIHQVAALLIAAYINVAKDSLVSSLIAQCRCRLRLVGLALASLGQDLKIDYQSQLSPAQENILNLRLKTCVLEHQTVLAAVTELQACFSKPTFAQFTVSLIIICVTAFQLVSQTGNLVRLLSMGTYLMNMIFQVFIYCYQGNKLSVESSEIAGSVYFSPWYLGSVKLRRALLIVMVRSRRVAKLTAGGFTTLSLASFMAIIKASYSLFTLLQQVKQKK

>BmorOR64

MGVSNGRGTVKPFLYPLVDELDYNLIVGVHLPFEYKTPSRYPLAYITVVIAFIYVSYFVMVTDLIMQAHLLHLLCQFNVLADCFENMLNDCVKGFEGPLVSLHEYIHPLIDEFEYNLMVGLRLPFSFDTPLRYLFTYVIVLIAFNYTAHYVMVTDLIMQSYLIPLICQYAVLADCFENILIDCSNDYGDHARRNDIVYSRSMELRAILSRPMLGQLASSGLLICFVGYQATTSISVNIVKCLMSLFYLGYNMFTLFVVCRWCEEITNKSLNIGNAVYCSGWESGMTVVPTVRSTILLVILRANKPIVFTAGGMYNLSLTSYTSLVKGSYSALTFLLRIQHE

**IR:**

>SinsIR93a1

MCYRKMQLWTLIFACVHVYVRAEDFPSLITANASLAVVLDRQHLGEKFQPVLDELRDYIKELARVELKHGGVVVHYYSWTTISLKKGFLAVFSLASCEDTWSLFSRTQDEELLLFALTEVDCPRLPSNSAITVTYMDPGEELPQILLDLRIARAFKWKSAVILHDDTLNRDMVSRAVRSLTSQTDNNDVPSVTVTVFKMKHEVNEYLRRKEIHRVLSKLPVKRIGENFIAIVTTDVMSTLSETARDLGMTHTQAQWLYVISNTNMHNGNLSHLINALYEGENIAYFYNITEDRPECQYDMTCYAHEMMNAFISALDAAVQEELDVAAQVSDEEWEAIRPTKLQRRNMLLKHMQQHIAVNSRCGNCSAWRALAADTWGATYRDHADNILATAAPTSNDTTGVIEHFELLDVGYWRPVDGFKQTDVLFPHVEHGFRGKELPIVTYHNPPWTILQANESGSIISHSGLIFDIVNQLAKNKNFTIKLLLPSNVKQDFSNETSTDMMHSQSAMLTLVAVAKGQAALAAAAFTIVSDPNPGINYTIPVTTQPYSFMIARPRDLSRALLFMLPFTTDTWLCLGFAVILMGPTLFVIHRLSPYYEAMGVTRQGGLSTIHNCLWYIYGALLQQGGMYLPRADSGRLVVGTWWLVVLVVVTTYSGNLVAFLTFPKQEVPVTTVSELIENSAIYTWSINKGSYLETELKNSDEPKYIALLKGAELTTGISGIEGHFGSGSSLLQRVRTQRHVLVDWRLRLSYLMRADYLATDTCDFALSSEEFLDEQVAMIVPTGSPYLNFTVVG

>SinsIR68a

MLKLKLAIFMFLSIEVSTDIASILKQLQERRDLEYVVTDLVNVVTRYDDVTCIAIICDQVYLNVFEGTLFRRTLAVPYVMIVVEDYEDLLSPNFDTLESLRETRKVGCSIYIILLANGIQASRLLRFGDRYRILDTRAKYIMLHDFRLFQSDLNYLWKRIVNVIFLRYHKKIVGVLKSKAWFDLSTVPFPNPIKGVFVSRRVDIWKNGKFHYNRTLFADKTRNLNKEVLNVVYLDYVPSVVVIKDNDTSKIGGVEMEILNMIAEKMNFKPKLYQPMNVELHKWGQKQPNGSFSGLLGEMVNGNADVALGNLQYTPYHLDLTDLSVPYTSQCWTFLTPESLTDNSWKTLILPFKLYMWIAVLLVLLVTGMIFYGLARYYINLMAYKHDPSDTGVSVPSSKGKHEEVDQFDEKPVGLYLFGEIINSILYTYGMLLVVSLPKLPTGWSIRLLTGWYWLYCILLVVSYKASMTAILANPAPRVTIDTLTELVESKVTCGGWGSETKNFFENSLDDAGQKIGQRFQTVDDPNEAAKRVAQGHYAYYDNKYFLKYLSVKRSNVFMNMEIEMDNSTMNGTEVTVRTETERNLHIMTDCVVNIPVSIGFHKNSPLKPLADIYIRRIVEVGLVEKWLNDAMDSIKSLEAEDEEIKALMNLKKLYGAFVALAIGYFLSVVCLIGELIHWYLIVKK

>SinsIR75q2

MKNICLTVFLLIVITCCHAETDLEVMMIANVIHAMERPSAVIATLCWPLHKKVQLYSILAGENVVQINMMQILKPGHIPQRHSQDQHIIFLVDLGCPDIYEYFVRSKIENHFRSPFRWIIIDGFNNGTHKSIIPQSLSNIEVLLDAEVLIARPIDNSTYDLHLVYKISHTNNWKIEFYGNWSIEYGFQKAYQLIDSAALRRLNLNGYEINICYVLTDNDSINHLTDGVNDHIDTITKVNFPTTNHLLDFLNAKRKYIFVETWGYRVNGTWNGMTGYLVREEVEIGGSPMFFTSERIAIVDYISSPTPTRSKFIFQQPKLSYENNLFLLSFRSTVWYSTLGLIFLLFLALFIVAMWERKRDQLKEKDADILRPSLADVALLIFGAACQQGSPVELKSSLGRVVMLILFLTLMFLYTSYSANIVALLQSSSSQIRTLEDLLHSRIKFGVHDTVFNRYYFSTATEPIRKAIYQTKIAPPGTKPRFMTMQEGVKKMQQGLFAFHMETGVGYKFVGKYFQEAEKCGLKEIEYLQVIDPWLAVRKNTPFKEMFKIGLKRIQEHGLQSRENHLFYEKRPKCTGTGSNFVSVSIVDCYPAFLVLFYGALVSISLLIIEHLLHKREMLMIRNCYMIPEPPYCS

>SinsIR75p2

MNVILKYAFLIFVIISNVISAKDDSMTDFIKRFVINEGKPTSLIYNNLCWSKHVQIKLAKEMSNSFIRFSTSIKLNKYQDHHSLFLIDLDCPNAEEILFNATAKNLFQFPYRWLVLSHSSSIEEYKVESLLKCPLLSDSDLVFAEKSQNGYKLVELHKSSPNYSIQYTPRGYYDRTLVDIRVHRELFRRRRDIMGHALTMSNVIQDSNSTQYHLSKEDRLELQYDAFTKICWINIKLAFQMLNATPRYIFSHRWGYRRNGQWSGMVDDLLSGRADVGTNLLMTVDRLDVITYTEGLSPYRVRFIFRQPPLSYVANIFSLPFSSGVWIAIVVCAIVSTVALYLASKWEIAIGKSPTHLDGFQDSLFLTMSAVSQQGCIMEPKKISGRIIMLFVFVSLMALYTAYSANIVVLLQAPSNSIRTLSQLARSKVTIAANDVDYNHIVFKLFKDPVRVSIQKKLEPENGKAQFYDMNEGVERIRQGLFAFHSIVEPVYRRIEKTFLETEKCDLTEVDFLNSLDPFTPIKKHSPYTELLRVVIKQIRESGILSAVYKRLQVPKPRCTEKVSAFSSVGLLDLRAVMFLMLIGAAVSIGVMFIEIIFHKLNKRQIATGNMKLNIIKCIYIRE

>SinsIR75q1

MPLYYFMNATTVLTFPDAWGYYINGTWNGMIGDVVSGKADLAGSVMFITRQRIDFLDYLIHPSPGLTVKFLFREPPLSYQYNLFLLPFKLNVWLFTLRETLNYKDHHLHIRTINLYLYRDL

>SinsIR41a

MLTLQPNVFHIELLLHTILNQYLHNSYCVTFVSEIPLDINFPITFTCIVPDPENLVNQLLEVSEKGCSDYIVRMKEPQEFMNAYETVNYLGNARRSDKKLIFLPFLEDDNTTNENILPLLNLLSTKETSFVANILLLIPSRESTAECKLYDIITHKYVGADKETNQPLYLDRWNSCTEKFVMNVNLFPHDMSNLYGKTVKVACFTYKPYVLLDLDTPTGRDGMELRIVEEFCRWVNCTMEVVRDDAHEWGEIYDNQTGVGVLGNVLEDRADLGITALYSWYEEYVVLDFSAPCIRTAITCVAPAPRLLASWELPLMPFTWHMWIALIFTFLFSSVALIVAKGFSSKNVFITTFGMMVTQCQPEVRADWRVRSITGWLLITGLVFDNAYSGGLASTFTVPKYETSIDTVQDIVDRKMEWGATHDAWIFSITLSTEPLIKQLVSQFKIYSAEELKRKSFTRNMAFSIEKLPAGYFAVGEYITKEAMLDLEIMLEDFYYEQCVVMLRKSSPYTSKISDLVGRLHESGLMLAWETQVALKHLNYKVQLEVKLSRSRRDVDNIEPLSFRQVVGIFIIYSIGVSLSIVIFVAEMCINDKTKKKLNQ

>SinsIR21a

MELLLNILILKFLFYAYGQEIEYYPSQNVLDNSVVKIGSNSKQQLINTEYNELFSKNAHDKIQWRYFNENESDKIKNISKRATDPVFYGHPKTTEELWNEHFLNQSSAFDQMPSLIKLIHNITLTYLSDCIPVILYDKQVKSQESYLFEDLFKHFPITYVHGYINDDDTLKEPKLLFSDQNCLHFIVFLTDVKTCTKVLGKQSQSKVVVVARSSQWAVQEYLASPLSRVFVNLLVIGQSFKDDDDDSLEAAYILYTHKLYTDGLGASRPVVLNSWSHGKYSREVNLFPKKMRKGYAGHRFLVAAANQPPFVFRRIKRDEESGNPKVVWDGIEVRLLQLLADRNNFSIEIVEPRELNLGPGDAVAKEIVTERADIGIAGIYLTEDRIREMDVTFAHSQDCAAFITLMSIALPRYRAILGPFHWHVWLALSFTYIFAIFPLAFSDKLTLRHLIHNGGEVENMFWYVFGTFTNCFTFVGKNSWSKTTKITTRLLIGWYWVFTIIITSCYTGSIIAFVTLPVYPETVDSVRQLLSGFYRIGTLDRGGWERWFLNSSDKYTNKLFKKIELVPSVEAGIRNTTKAFFWPYAFLGSRAELEYIVQSNFSMTKSKRGLLHISNECFVPFGVSFAFPNNSLYTAKFSNDVRRMLQSGIIQKIVDEVRWEMQRSSTGKLLSAGIGSLNTLPIEEKGLTLEDTQGMFLLLGAGFLIAASALISEWIGGCSRLCRLSKNKNPPTSINSGDHLIPTPKTDTQSTINIISDGTDSRLHFDTRPQSADSRDTLDGQIINVTEENITVHDNFKVDGWDSRRSSSIDLDREVKEIFEKDQKRRRIFSDGMIELSGNKRHPTASKGAFGDTVGN

>SinsIR75a2

MFITKQRIDVLDYLIHPTSGLTVKFVFREPPLSYQNNLYLLPFKLNVWLCIAAFVMILAFILYVNAFWETQKSKSNDKDKLDNTTLKPNVSDIAIFVISAMSQQGSAMELKGTLGRVVTFILFLTFLFLYTSYSASIVALLQSSSKQIRTLSDLLHSKLELGVEDTPYNRYYFSTAKDPVRKAISQKIAPPGSKPNFLNLEDGIKKLQKRPFAFNMNLGTGYKIIERYFHEHEKCGLQEIDFIPDNKPWLGCRKYSPYKEILKIGLYRLQEHGLTMRENRLMYSTKPVCTARGGSFGSVDILDCYPVLLMLLYGMILSFFLLFIEILFHRRQKLLRRIRDIR

>SinsIR76b

MELIISSICNATFCEAVYDNPLIESQLTKTQIELLALAEELNGKHLKIGTYNSYPLSWTERADNGTLIGRGVAFTIIDILREKFNFTFEVVIPNNNFEFGGSRPEDSLIGLVNSSKVDMVAAFMPTLYKLKDLVSSSVDIDEGVWVMMLKRPAESAAGSGLLAPFESHVWYLILTAVLSYGPCITLLTRLRSKLIKDHERYIPLSPSCWFVYGAFIKQGTTLSPEANTTRVLFATWWLFIILLSAFYTANLTAFLTLSKFTLDIETPRDLYKKNYRWVATQGGTVEYSVKDPDEDIHYLNQMISKGRAEFRSVSNSEDYLTMVKGGAVLVKDRTGIDHMMYADYLNKARQGIEETSRCTYVIAPNSFMKKNRAFIFTKNSKLKKLFDPILTNLLQAGIVNFLKNRDLPSTKICPLDLQSKDRRLRNSDLMMTYLIMVIGLASAIAVFIMEIIIKKCFHIRLKTEGPRPKRTRANRVRFQNHDETQPPPYESLFGRNSRYKMTDQFQTKIINGREYWVVDTVSGDTRLIPMRTPSAFLYQRQRRM

>CsasIR75q.2

MKMIYYLKILLLTISNTICYAETDFEPNIVHDVIQAMGRPSSVIATLCWSDQKKMQLVSTFSGSISKQAAMIVFVQQGQVEVQTYDDHHIVFVVDLTCPNITDHLVQNNRANHFRVPFRWLLIGYENSESSVPEELTVLDLLPDSEVIIVQHVGNSVYDINYVYKISVNGTWYTEAFGYWNKGGGFKKIIRPESTALRRLDLKGYTITICYVLTDDDSINHLTDEVNDHIDTITKVNFPTTNHLIDFLNASRKYIFVNTWGYKVNGTWNGMTGYLVREKVEIGGSPMFFTSERVSIVEYISSPTPTRSKFVFQQPKLSYENNLFLLSFQNSVWYSSLALVTIMFLAIFIATLWEWKKRGYNEKTDTDAHVGLLRPKMSDVIILIFGATCQQGSPVELKGSLGRIVMLILFIALMFLYTSYSANIVALLQSSSSGIKTLDDLLHSRLKFGVHDTVFNRYYFTTATEPTRKAIYETKVAPPGVKPRFMTMEEGVKKMQQGLFAFHMETGVGYKFVGKFFHESEKCGLKEIQYLQVIDPWLAVRKNTPYLEMFKIGTKRIQEHGLQSRENHLLYERRPKCSGGDGNFVSVSMVDCYPALLVLSYGSIMAVFFCVLEILYKRKHEILTRLSCHSDIE

>CsasIR76b

MDTGVALIISSICNATFCENVYDNPLVEQQLSHNQNVLRELAREVNGKRLKIASYSNFPLSWIDEGDNGTLVGKGVAFVLVNILSKKFNFTYDVVRPEKNFEVGGTRPEESLIGLVNNSMVDMAAAFLPTLMRYRQKVTFSTVLDEGTWVMMLKRPKESATGSGLLAPFKNHVWYMTLAAVLCYGPCITLLTRIRANIIKDDNYCINLSPSFWFVYGAFIKQGTTLAPDANTTRVLFATWWIFIILLSAFYTANLTAFLTLSRFTLDIEYAHDLYKKNYRWVAQEGGAVQFIIQDPNEELHYLQQMVQKGRAEFRSLSQSVDYLPLVVGGAVLVKERIAVDHLMYTDYLVKAKAGVVESDRCTYVVAPNAFISRIRAFAYPIGSKLNVLFDPVLAHLVQAGIVKFLANRDLPSTKICPLDLQSKDRQLRNSDLMMTYMIMITGLSAAAAVFIGEIVFKRYIHIKIRKNKGDKPKKKKTNKRIRFNEYDDAHPPPYDALFGKSSKYKTENSTKKIVNGREYWVVDTVNGDSRLIPVRTPSAFLYR

>CsasIR1

MKHFYVVLYFAFVNEITGDDYNHFLSELNHKIECPLRLTHTDQTWSLLLDLTTKLEQELLNCLIKGEKEDTRWLIISEFNERLINHLTSNEYNSKGYPITVIIKNLNYSVIDHPLIKHFLAKSQWSIIYFIIDTKEISYSCQNGAFENDTMLIIEDYFNEIWQKYQAVNVVMTFPDCPRYYIIFSEVKELIDTDNLYRRKITIVQADDQKNLQRNVMKKSRRVSEGYPLRANIFDRFPTSINDCSGMVYYVNPKRDFIKNFCGMDAMIMSDVVKHFKFHLSFPDLGEDGSMYGFLKNNKTLSGTLKHIVQKNIDVSFNSRFMANYMEKREYDFLLFVSVDALCLVTPRPDFVPLWHYPYNVYSMIIWALLVVVLSITGLFTWLIVKYVQKPTKKSFSSFVGYTIDCIITGLFGISMTRRFIITRAFCLGVSIILSALYQSHINYVFTTLVHYDSMKTIEEVYRSCTIYLSPSIADLLKPPSNDLQETILAAMVTEPPTNGSRLAFLLEHPNTASVERKADIILEIFKYHTDDRGFPKLYVIEECFRQYYLSYISRIGFLFNEQLATFIARLNEAGLPSQYYTWTRYALRLPKTSFNMGPEPRPFSKITLGEQIIPFYILFIGYVSSTIVFVVEIWQAQRKAHIKPIIE

>CsasIR41a

MNLSQIIEPIGTLLQIILDQYFITSFCVTIVSETPLDLKIPINFMYIKPRAENLADILLDASDKGCSDYVVLMNEAEIFMAAFEKVNHLGLVRRSDRKIVIAPIANGNNILNILSLKESEFVPNILVVAPEELIDGCQVYDLVSHNYVGLDVESNLPLYLDRWDSCTEKFEKKTHLFPHDMSNMMGKTLKVACFTYKPYVLLDLDITKSATGRDGIEMRMIEEFCSWVNCSIEIVRDDVNEWGDIYENNTGVGILGNIVEDRADVGISALYSWYEIYRVLDFSAAGVRTAISCIAPAPGLLDSWETPLLPFTWEMWLCIIFTYIYASLALSIANGWSIDRVFLTTFGMMITQSQFDAGTSWRIRSVTGWLLITGLILDNAYGGGLASVFTVPKYESSIDTVQDLIDHKMEWGATHDAWIFSLTLSSEEKVKQLISKFRVYSAEELQRKSFERSMAFSIEKLPAGYFAIGEYITKDAVPSYQLMLEDFYYEQCVVMLRKSSPYTAKISDFVGRLHDSGLMLAWETQVAINNLDFKVQLEVKLSRSIRDVENVQSLSFRNVLGIFFIYIAGVTVAILAFLAEKLTNNKKQKRANMVIKFT

>CsasIR8a

MWDMFQINMEIYFVLFIFLINLACVVSEISLRFVFIVEAQEMDLPQKIGRALKLSEEMRPEIRVSEDVVMLDRENDEESYRKLCAALSNGASMLIDLSWAPWQAAEQVSMDSGLPLIRTQLGSQQLLTALDSYLETRNATDAALLLESESDVDRTLYELLGQSNVRVWVHAGLTKDSARSLKIMRPEPSFYAVIGEGGFVADTYRRAVKEKLVRRTYRWNLVLTDYLPFDVAQLVLPTMILQIDPAECCRLINQRENCNCPGDVPRKQQILTALLQYIIETYYKLEQDLPLMINRVECDVVVVDFNGTRDRLYRQFAEDTAMSNDTIFYWNSERSGLFLRSGFVLSTYTPDEGQQTVATWSASEEFKLLPGITLEPLRLFFRVGTAPAVPWTMLKIDPDTGEPMFDEEERPIYEGYCVDLIDKLAETMGFDYEIVPPTTGGFGKKLPNGTWDGVVGDLTRGETDIAVSALTMTAEREEVIDFVAPYFEQTGILIVIRKPIRKTSLFKFMTVLRTEVWLSIVAALVLTGFMIWLLDKYSPYSARNNPEAYPYPCREFTLKESFWFALTSFTPQGGGEAPKALSGRTLVAAYWLFVVLMLATFTANLAAFLTVERMQTPVSSLEQLARQSRINYTVVEGSTVHQYFINMKFAEDTLYRVWKEITLNATSDQAQYRVWDYPIREQYGHILLAINASIPVPDAKTGFDQVNEHTEADFAFIHDSAEIKYEITRNCNLTEVGEVFAEQPYAIAVQQGSRLQEDLSRALLELQKERFLEQLASKYW

NETARQACPDADESEGITLESLGGVFIATLFGLGLAMITLAWEVFYYKRKERNKVQTIDNKNVEAFAEPKKNIEKKIADGVARIRKRKKLGKVDKVGKSVTIGDSFKPAAEKGVSYINVYPKGGFQP

>CsasIR21a

MILHRKNICLLIFYLELVINFCIIEVVRSEDVEYYPSQSVSLSSKKSKVFHSHRRKRYSRNGSKLEFRRFNNPAKETDVVKIKRSADPVFHGHPKTREELWNEHFLNLSNAFDQTPSLIKLLHNITMTYLDDCTPVILYDKQVKNQESYLFQNLFKDFPVSYVHGYINERNELMEPALLQSVKECIHFMVFLKDVSVIAKVLGKQSQSKVVIVARSSQWAVQEFLAGPLSRMFINLLVVAPSFKDDDDTSIEAPYILYTHKLYTDGLGASQPRVLNSWTHGKYSRDMNLFPVKMTAGYAGHRFLVATANQPPYVFRRIISDSDGGNPRVIWDGIEIRLLQLLASRNNFSIEFIEPREPNLGPGDAVAKEVAQGRADIGVAGMYMTDDRARDMDMTVGHSQDCAAFITLMSTALPRYRAILGPFHWHVWVALTFTYLIGIFPLAFSDKHSLRHLIHNSGEIENMFWYVFGTFTNCFTFVGKNSWSKTTKITTRLLIGWYWLFTIIITSCYTGSIIAFVTLPVFPETVDSIQQLLMGFYRVGTLDRGGWERWFFNSSDPKTNKLLRKIELVPNVAAGIRNTTKAFFLALRILGIACGIRIHCTS

>CsasIR68

MLKAIVFVFFTLGQLNGANANISNMLKEVEHTKDLQYLLIDLVNVMTRHYDVTCIAVLCDTTYLNVFEGTLFRWTIAVPILMIVVEEYEDLLSPNFDTLEALKEAKKNGCNAYVVLLANGLQASRLLRFGDRHRILDTRAKYAMLHDVRLFHSDLHYLWKRIVNVIFIKHHQKISGVLKSKAWFDLSTVPFPNPIKGIFVPRRVDIWDNGRFHYNRHLFADKTKNLNYEVLNVVYLDHVPSVIVQKMNESNKVGGVEIEILNTLSQKMNFDPKLYQPSNVELHKWGQKQANGSFSGLLGEMVNGKADMALGNLQYTPYHLELTDLSIPYTTQCWTFLTPEALTDNSWKTLILPFKLYMWITVLLVLLITGAIFYGLAKYYMYLEKFIKDQRKFNKSVSNEKQDEEDAKPVGLFLFGEIINSILYTYGMLLVVSLPKLPSGWSIRLLTGWYWLYCILLVVSYRASMTAILANPAPRVTIDTLKELVDSKLTCGGWGTQTKAFFEGSLDEIGQKIGERFEAINDPFDAANRVAQGVYAYYDDSDFLKFLSVKRKNTFLMEDMSNSTANATEIMALKTANERNLHIMSNCVVNIPISIGFHKNSPLKPLADIYLRRIVEVGLVEKWLNDAMQSIRSLGSNIDEVKALMNLQKLYGAFIALAIGYTLSLFCLIGELLYWNCVVKKDPNYDKYALDLYYKNKRH

>OfurIR21a

MTRLKCLIANIFIFVSFVLCEDVEYYPSQAALNSYSNIAKRSVNEPELEKGKVAIKWRHFNENKNETQEVKTKRAVDPIFHGHPKTREELWNERFLNKSSAFDQTPSLIKLIHNITLRYLNDCIPVILYDSQIKTRESYLFQNLLKDFPVSYVHGYIDDNNKLKEPELLIPVKQCLHFIVFLTEVKSSAKVLGKQSESKVVVVARSSQWAVQEFLASSYSRVFINLLVIGQSFKDDDDNSLEAPYILYTHKLYTDGLGASQPKVLSSWTHGKYSRDVNLFPPKMTEGYAGHRFIVAASNQPPFVFRKIKTDLDGGNPRVIWDGIEMRLLHLLAERNNFSIEILEPQEPHLGSGDAVTKEIAMGRADIGVAGMYLTVDRTKSMDMSFSHSQDCAVFITLMSTALPRYRAILGPFHWHVWVALTFTYLIGILPLAFSDKHTLRHLLHNSGEIENMFWYVFGTFTNCFTFVGKNSWSKTTKITTRLLIGWYWIFTIIITSCYTGSIIAFVTLPVFPETVDTIEQLIAGFYRVGTLDRGGWERWFFNSSDAKTNKLFKKLELVPNVESGIRNTTKAFFWPYAFLGSQAELEYIVQSNFTATKSKRAMLHISNECFVPFGVSMGFPTNSLYSAKLSGDLRRMFQSGIVDKIVDEVRWEMQRSATGKLLSAGSGSLKITSAEEKGLTLDDTQGMFLLLAAGFLMGASALVSEWMGGITRRCRIGRKKPSSANSKEELIATPELESEIKVISDCTESRLNFDTRCSSACSRDTLEGQVINVTEENIVVHETLDAATWDSRRSSSVDLDREVQEIFEKDLRRRRIVTGDIEEAAEVKRELTASNGAFGDHLN

>OfurIR25a

MKPKESRFSLKLLLLFSFVRVAIFQTTQNINVLLINEENNALAEKAFEVAKEYVRRNPSLGLAVDPVIVVGNRSDAKVFLENVCRKYNDMLSAKKTPHVVLDFTMTGVGSETIKSFTAALALPTMSSSFGQAGDLRQWRSLDANQTRFLLQVMPPADILPESIRAIVTKQDITNAAIIFDELFVMDHKYKSLLQNIPTRHVITPVKSFNKDEIKTQLRSLRELDIVNFFVVGSLRTIKNVLDAADENQYFGRKTAWFALSLDKGDITCGCKDATIVYMRPTPDAKSRDRLGKIKTTYSMNGEPEITSAFYFDLSLRTFLAVKSLLDSGKWPNDMKYITCDDYDGKNTPNRTLDLKAAFQEIKETPTYAPFYIPEDDPMNGRSYMEFNTDITAVTVKDGASIGSRVLGSWKAGLSNPLSLTDPDNMSDYSAQLVYRVVTVEQEPFIIRDDEAPKGFKGYCIDLIEEIRQIVKFDYEIVLSPDGNFGTMDENGNWNGIIKELIDKRADIGLTSLSVMAERENVVDFTVPYYDLVGITIMMKLPRTATSLFKFLTVLENDVWLSILAAYFFTSFLMWVFDKWSPYSYQNNREKYKDDEEKREFTLKECLWFCMTSLTPQGGGEAPKNLSGRLLAATWWLFGFIIIASYTANLAAFLTVSRLDTPIESLDDLSKQYKIQYAPLNGSAAMTYFERMAHIEVKFYEIWKEMSLNDSLSDVERAKLAVWDYPVSDKYSKMWQAMKEAGLPNSIEEALQRVRDSKSSSEGFAWLGDATDVRYHVLTSCDLQMVGDEFSRKPYAIAVQQGSPLKDQFNNAILQLLNKRKLEKLKENWWTNNPNAMKCEKQDDQSDGISIQNIGGVFIVIFMGIGLACITLGVEYWWYKIRKRSTIGDITQVEPAKSSRINTDFKGEGFTFRSRNLGLSNLKPKF

>OfurIR40a

MKFVPFFLFLNTAHCFFDIQDIISQTMTKLPKDFAVAIKDIAEGLPAKTITVVRGESTKIRSQDIFQLLCLLSEHNIQVINLDITTKQNKDKYYSFVKQALDISEDRTSLILCEPFECEKILTELTDNNLIHRTILYIFYWPYGTVSDKFLNTMKEAMRVAVLTNPRESVFRVYYNQATPDRLHHLSLVNWWSGRLYKSPVLPPAGKIYQDFKGRMFDVPVLHAPPWHFVRYNNDSSVNVTGGRDDKLLSLISKKLNFRYQYYDPPDRSQGSSISGNGTFKGTLGLLWKRKADFFIGDVTMTWERLQAVEFSFLTLADSGAFLTHAPAKLSETLAIIRPFRWEVWPLVCATVLVTGPALWVVIAAPSLWQRRQRDQLRLLNNCCWFTTTLFLRQSSSKEPSKTHKARLVSVLVSLGATYVIGDMYSANLTSLLARPARERPIGTLQALEEAMRDRGYELVVERHSSSLTILENGTGVYGRLARLMRRQRVQRVRSVEVGVRLVLTRRHVAILGGRETLYYDTERFGSHNFHLSEKLYTRYSAIALQIGCPYLETFNNVVMTLFEAGILAKMTTDEYKNLPEQSRRSEPVTESDKPNNDITGDSPAASQGGTTPGESTKALEPVSLRMLRGAFCLLGIGHLLAAIALGVEIQIHRRSKKFIKIVEPNGGKNVPGKRALRKANKFIRQGIGRMVRAFCRSVDRALGPGNQ

>OfurIR8a

MMEIPLLLLFLINLGCVLSEISLRFVFITEVHDSDLAHQIGRALRNAEEQRSGVKISDYMVQLDRENEDESYRRLCSGVSKGASLVIDLSWAPWDMAEQLCAESGLPLVRTLLGSQQLVAALDEYLESRNATDAAILLESESDVDKTLYELLGRSNVRLWVHAGLTRDSAKALKSMRPEPSFFVIVGESGFLMDTYRRAVKEKLVRRDYRWNLVLTDYSGDSIDVTQLPLPTMILHVDQVECCRLLGLREECSCPSDLKRKQLIISALVLYLSETYSKLERELPVLSTKVDCDNVLASEMNVTRDRLVRQFGEDVEINNDTLFYWDDDRSGLFLRSSFVLSVYRPDSGLETVASWSANDEYKLLPGVTLDPLKLFFRIGTSPAVPWTLPKLDPETGEPEVNEDGQPVYEGYCIDLISKLAETMEFDYEIITPKSGSFGKKLPNGSWDGVVGDLMRGETDLAVAALTMTAEREEVIDFVAPYFEQTGILIAIRKPIRKTSLFKFMTVLRTEVWLSIVAALVLTGLMIWLLDKYSPYSARNNPQAHPYPCREFTLKESFWFA

LTSFTPQGGGEAPKALSGRTLVAAYWLFVVLMLATFTANLAAFLTVERMQTPVSSLEQLARQSRINYTVVEGSTIHQYFINMKFAEDTLYRVWKEITLNATSDQAQYRVWDYPIREQYGHILLAINASGPVPDAKTGFEQVNEHTDADFAFIHDSAEIKYEVTRNCNLTEVGEVFAEQPYAIAVQQGSRLQEHLSRALLDLQKERFLEQLASKYWNESARQACPDADESEGITLESLGGVFIATLFGLGLAMITLAWEVFYYKRKERNKIQGIDAKVEKAAFVDPKKKDKLGVRLRKGKSKVAKLDVVGKGKGVTIGDTFKPAAEKMGVSYISVYPKGEYRP

>OfurIR41a

MLQDTVLFFPVEILLTSIVNLYLNSSYCLTIVSEKSLDLSISNSFTSMVPEDGDLLVNQLLQVSEMGCSDYIVKMRDPARFMAAFERVNHLGNVRRSDRKVVFLPYEDNNITRTDLLQLLTLKETSFLANILLILPSLESGLCSIYDLATHKYTGPDDQVDQPYYMDRWNSCSLKFEKDANLFPHDMTNLHGKTVKVACFTYKPYALLDLDPLEEPLGRDGTEVRIVDEFCRWINCTIEVVRDDEHEWGELYDNQTGVGVLGNVVKDRADLGITALYSWYEEYLELDFSGSGIRTAITCVAPSPRLLASWEMPLLPFSWYMWMALGFTFVYASFALAIAKGCSTDKVFLATFGMMVTQSQADVGATWRVRSITGWMLLTGLVLDNAYGGGLASVFTVPKYEKSIDTVQDIVDRGMEWGATHDAWVFSLTLSPEPLVKQLVSLFRVSSAEDMKIKSMQRSMAFSVERLPAGYFAVGDYITKDAMLGLTLMQEDFYYEQCVVMMRKSSPYTQKVSKLIGRLHESGLMLAWETQVALKYLNYEVQLEVRLSRFHKDVDNVEPLKLRHVVGVFIIYIIGVIISTMLFILEIIHKHKKRVY

>OfurIR64a

MDINYFLNFISIAEISLVIDLLKLKEIQNVVNINCDGQKSIFHHKILNDNNIHASYWSLNSTDQNMMQMSYHKTGVILDASCSNWEQALNNFDNSMFRNEFIWLIITEDLLSTARSLTNCPIEIDSDVTVALKTNGIFMLYEVFHTNYSSGVLSIRNVGYWDTTLHIATSSRRDLQGLKMRCPVVVTDKVVHQTFEEYLSKHQVFQVDSLHKLKFVALLNYIRDMYNMSYELQRTNSWGYMRNGSFDGVVGSLQRQHADFGGSPLFFRADRAELIDYIAETWQSRQCFILRHPKHPGGYYTIYTRPLTAKVWYCILAMLIFSGVILCLMLKTKVTQSHEKSTDSSFSLALLFAWSAICQQGMTVNRSSTSVKIVVIVTFVYAVTLYQYYNATVVSTLLREPPKNIRTLEDLLQSNLKAGAENVLYTKDYFKRTTDPVALRMYHKKITPKHQYNFYSPEYGMSLVKQGGFAFHVDSVVAYRIMRKTFTEREICEAHEVLLYPPQKMGMVVRKASPYKEHFTYGIRKIYEAGLMDRLQSVWDEPKPSCVHTPDSSVFSVSIVEFSTALLALVAGNVAAILVLFAEIVLHRCEMKKRIAFTH

>OfurIR68a

MWIAVLVVLLISGSIFYGLARHYMNLQEYIKTHENRNTNEKQLDTAEKPVGLYLFGEIINSILYTYAMLLVVSLPKLPTGWSIRLLTGWYWLYCVLLVVSYRASMTAILANPAPRVTIDTLKELVESKIACGGWGMETKKFFQESSDDIQTIGQRFETINDPFVAANKVAKGVYAYYDNENFLKYIRVKRKNIDMNIQSDMVNATSNTTDVFAGEMERNLHIMSDCVVNTPISIGFHKNSPLKPLADIYLRRIVEVGLVEKWLNDAMHPIKSLETNEDEVKALMNLKKLYGAFIALAIGYSISTIGLIGELIHWHLIVKRDPKFDKYAIDLYYLSKNKKQ

>OfurIR75

MNLRDLLLVFILVYFVCTFSKSSDEISIITDLIQSSDKLTSVVAHACWKPSKQIQLASRLGNKNRPMTVRFVNKNWAGIVEPQHRERLLIVADLNCPSTKVFFKLANTTNKFSFPYRWIVIGKAVNESVAVTSNFQNIPLLPDSDVIIAQKNDKNSYILTTIYKIQIKGKWIVEQFGIWTSANGLKKFEAVKHPISTRRKNFQRAPIKMAMVILDNRTISNPYDLSDILTDTVSKSSFRQTDPIFGYLNASRTLIYSPTWGYYRNGSYGGMIADMTIGDAELAGTVLIATQDRMEVVEYLSCPTPISIKFVFRQPPLSYQNNLFLLPFKSTVWYCIGAFVLVLAFILYINALWENKKLESSEQNFEDPTVLRPNVGDIAILVISAISQQGSSTELKGTLGRIVMFILFLAFLLLYSSYSASIVALLQSSSNQIRTLSDLLNSKLELGVEDTPYNRYFFPIATEPVRRAIYQTKIAPKGTKPKFMSLEDGVKKLQKEPFAFNMNKGIGYRLVERYFHEHEKCGLQEIPYLYATKTYITCRKNSPYKEIFKIGLFRIQEHGLSDRENRLIYARKPPCQARGGSFGSVNMVDFHPILLMYLYGILLAFFFFFVEILAHKKLHPRQSQRR

>OfurIR87a

MCTIIFLPLFFALHVSATINENSLLTTTGNSEQTAKTAECVLKLSAKYFVEKKALSGSIVIININSYASTTQVLLLQTIHGGIKYSVMVKDSFYPHANASHFPEKAKNYMLILEEKSELTRNILQLNKLPTWNPLAKAIVFYQLNKTEDAEQTAIEFINELRHYKLFKSIIFIYSPEEKEVISYTWTPYSDTNCGGKCDSVYILDTCKDNVIHELATQKEMFPLDMKGCPLVTYAIVSEPYVLPPAMKLSNTSYNDAYVFQKGGEINLVKIITQFTNMSLVMRTSDVPENWGNVYWNGTATGAYGVLRNDEVDMVIGNIEVTRTIRRWFHPTVSYTQDEMTWCVPKAGQASTWNNLVIIFQWSTWVATFGSLFVMGLLFHYMYYRENNQKVTKWPTNSLLMTFSMLLGWGASFEPKSATFRILIFGWLCFSVNMGISYESFLRSFLMHPRFEKQISTETDLIQSRIPLGGREIYRSYFETNNASSFYLYRKYNSTTFAEGIKRAAKDRNFAVVSSRRQAAYADQRLGKGKPLIYCFPESDNLYKYGVVLLARKWFPMLER

FNTIIRSVSENGLIDKWNQELLIHTANAEGASEIEPLSIQHLLGAFMFIGFMYAASIAVFIAEICIGVFQKWKARKNWQSLYKHIRFRNAK

>OfurIR93a

MRICLFVSLYLLRVSGEEFPSLITANASIAVVLDRQFLGEQYQATLDELKDYIKELARVELKHGGVVVHYFSWTTISLKKGFLAVFSVASCEDTWSLFSRTEEEELLLFALTEVDCPRLPTDSAITVTNVMPGEELPQILLDMRTEMAFKWKSAVILHDDTLSRDMVSRVVQSLTVQIDEGASTSPVSVSVYKMKHEINEYLRRKEITRVLSKLPVKYIGENFMAIVTTEVMTTMAEIARDLVMSHTLAQWLYVISDTDAQNGNLSSLINALYEGENVAFMYNITESNPECKNGLMCYCQEMMNAFISALDAAVQDEFDVAAQVSDEEWEAIRPNKIQRRGMLLKHMQQHISTKSSCGNCSTWRALAADTWGATYRSYGDSDQLFKEPDNATTKGVIEHVDLLQVGYWRPIDALRFEDVLFPHVEHGFRGKALPIITYHNPPWTILQVNESGSVVSCSGLIFDIVNQLAKNKNFTVKVILPSHVKNLLSNDTTADMMHSQDAALTLIAVAKGQAAIAAVAFTVLSDPPSGINYTLAVSTQPYSFMIARPRELSRALLFLLPFTTDTWLCLGLGVILMGPTLYIIHRLSPYYEAKEITRQGGLSTIHNCLWYVYGALLQQGGMYLPRADSGRLVVGTWWLVVLVVVTTYSGNLVAFLTFPKQEIPVTTIGELLENQLTYTWSIQKGSYLEMELKNSDEPKYAALLKGAELSGAGASGNLSSWKKQLIRIREQRHVIFDWKLRLSYLMRAEHMLTDTCDFALSAEEFMDEQLAMVLPAGSPYLPVINKEINRMQKAGLISKWLFAYLPKRDRCWKTSSIAQEVNNHTVNLRDMQGSFFVLFLGFFSASVVLLLEWFCNRRRRRSEDVIIKPYVE

>OfurIR1

AAATSLYHKHQDITPILRWENVIDKVDLVHPPVTSIETRYFYRIPTYGAGKFENQFLRPLSYGAWISVVIVITLCACVLLISAKLERRRSAGQYAIFSVLASMCQQFFEDNTSFTPRVSAARQLTIFVTGISCVLIYNYYTSSVVSWLLNGPPPSINSLQELLESPLELIFEDIGYTRSWLQKKSYYYNIRNIEIEDELRKKKVFNKKPNAPLLVPVEEGIKLVKAGGYAYHTDTNNANRLISQTFTQSELCELGSLQSMAKAELYPALQRNSPYKEFFVWGSIRLYERGVVKFVQRRISSPAVECEGSSPRALALGGAAPAFLLLAAGYLLATVIMLVERAIWRRKYKSKVVLKLKK

>OfurIR2

RVLVPAKLWYSTQDVLRSESADLNAGVLRIMETRMEYLDYIMPIWLFSVGFTYLAERESSSNMYVEPFTAACWWTCLGIGVTLALAQRVAARGKQEKEGAFMAVLATWLQQDAAAVPDGVAGRWTFTVMSICAMLVHAYYTSAIVSALMSTGRGGPNTLRELADSRYAIASEDHDFIRNGMFNVETDWEELEYLKKKKMTSKLFQDMEYGVQLIQQGYTAYHAEYHQLYHFLEKFSDDEICKMQHVDTIPEIMTWVSASARGQWTEIFRSTGAWLYETGLARQLLSSIQTHPPPCRAAMLAERVTFWDVAPLLGLTTIGAIASIGLLGLEIVLHRWTEKNRREKKWGSSEASLVALE

>CpunIR8a

MEIPLFLLIFFINLGCVVSELSLRFVFIIENHEPELGQLVGRALKVAEEQQDVRVDDSIVLLDRENEEESYGRFCSAISKGVSLIVDLSWSPWELAESVASGGGVPLVRTALSMQRLLSAVASHCASRNATDAALITESEADVDRALYELLGRSNIRLWVHAGLTRDSARALKNMRPDPSFYIIVGESGFVMDTYRRAVKEKLVRRDFRWYLLLTDYSGDSFDTSQLVLPTMMLHVDANECCKLLGSKDDCSCPSDLKRKQYILSGLMTYLAETYSKLENDLSVVTAKMDCDNIQASEMNVTRERVVRQFADDEQISNDTLFYWDGERSALYLRSTFVLSTFKPDSGLETVASWSANEEYKLLPGVTLEPLRPFFRVGTSPAVPWTLPKLDPDTGEQVYNEDGQPEYEGYCIDLIARIAETMEFDYEIITPKSGTFGKKLPNGSWDGVVGDLMRGETDLAVAALTMTAEREEVIDFVAPYFEQTGILIAIRKPIRKTSLFKFMTVLRTEVWLSIVAALVLTGLMIWLLDKYSPYSARNNPTAYPYPCREFTLKESFWFALTSFTPQGGGEAPKALSGRTLVAAYWLFVVLMLATFTANLAAFLTVERMQTPVSSLEQLARQSRINYTVVEGSTIHQYFINMKFAEDTLYRVWKEITLNATSDQAQYRVWDYPIREQYGHILLAINASGPVPDAKTGFEQVNEHTDADFAFIHDSAEIKYEVTRNCNLTEVGEVFAEQPYALAVQQGSRLQEQLSRALLDLQKERFLEQLTSKYWNESARQACPDADESEGITLESLGGVFIATLFGLGLAMITLAWEVFYYKRKEKNKVQTIDAKMEKAAFTEPKNAEKTGVRFRKKEKKSKLGKISKLGKVEEGKLGKRVTIGDSFKPASEGAGVSYISVFPKGEYRP

>CpunIR41a

MINILRIIFTIQIDWVNKFILFFKYKNFISYLFTMMLDSVLSLMPIEILLQTIFNEYLSNSYCLTVVSEKPLDLHVNISYAYISVENGELSPDQMLKLSENGCSDYIVQVKNPQKFMGAFETVNLLGNVRRGDRKIVFLPYREDNATTTLLLEILTLKETSFIANILLILPSPEQSTCSYYDLVTHKYVGQDNEINQPYYIDRWNACTLNFEKNVSLFPHDMTNLYGKTLKVACFTYMPYVLLDLSEAQEPFGRTGTEIKIVDEFCRWVNCTVELVREDEHMWGEIYDNLTGVGVIGNLVEDRADIGITALYSWYEEYVVLDFSAPGVRTAVTCIAPSPRLLASWEEPLLPFSWYMWLALIFTFVYASLALTIAQGFTTDNAFLTTFGIMIAQSQHDVGASWRVRSVTGWMLLTGLVIGNAYGGGLASVFTVPKYEKSIDTVQDIVDRKMEWGATHDAWVFSLTSSNEPLIKKLVNQFKVYPADVLKKKSLDRSMAFSIERLPSGYYAIGDYITKEAMLDLTVMLEDFYFEQCVAMLRKSSPYTKKISQLIGRLHESGLLLVWETQMALKYLNYEVQLEVRLSRSQKDINTEALSLRHVVGVFILYLIGMIFSVIIFTLEVMNVNKKRNTSSF

>CpunIR2

MLNKMQDMKTYEVLFILLMSCFSLIIANPINEFRMIADVIKDSNKSTSVVAHLCWNPSKQIQMASYLHNSELTQLVLLVNESWADIKEPQHRERLLLIADIDCPSTTAFFKMANETKKFSLPYRWLIIGKAVNKSTDVTADFDGLHLLPDSDVIIAQKNDSNSFYMNMIYKIKIKSKWIIEDFGTWTTNTGLIKSDLAQYSTSTRRKNFHGESFTTAMVIFDNKTISNLFDLSDILTDVVTKSSFRQIVPLYGYMNASQQHIYSKTWGYYRNGTFDGMIAELTVGDADLGGTVLIVTWDRMQVVDYLSKPGSITVKFVFREPPLSYQNNLYLLPFKVTVWYCMGAFVLVMGFILYITALWENKKMGENQEISNDPTVLKPNVSDIAILIISAVSQQGTTLELKGTLGRIVMIIQFIAFLLLYASYSASIVALLQSSSNQIRTFSDLLNSKLELGIEDTPYNRYFFPIAVEQVKKEIYKSKTPPRWTEPKFMSLEDGVKKLQKKPFAFNMLQGIGYKLVERYFHEHEKCGLQEIELQYGTKTYIASRKNSPYKEIFKIGLFRIQEHGISDREFRLLYARKPTCQVRGGNFDSVNMVDFHPVLLMYLYGILLAIALLVIEILVFKKQQLMCSAASRRQRSGSC

>CpunIR3

MKATVLLLFCLKYLNVKSHANTNSVMHMVGDIIRAMEKPSSVVATLCWLTDEKVQFYYAVTASDRFSRVNTAQFVDMRHVSEDHGQEQHIVFVADLSCPNISAYFDEKRAQNYFRGPFRWILIGNVVEEDIVPNSIAHIDALPDSQVIVARQIDEESYDLYTIYKINANDDWRTKLYGKWNQQTRFTITNPHMESIALERLDLLGSEISVCYVLTDKDSINHLTDEVNDHIDTITKVNFPTTNHLLDIVNASRKYIFADTWGYRVNGTWNGMTGYLIREEVEIGGSPMFFTSERISVVDYIASPTPTRSKFVFQQPKLSYENNLFLLSFRTSVWYSSTGLIFLLLLALFVVAAWEWKKHTNDNQVSFQRENDAGTLRPNFVDVIVLIFGAICQQGSPVELKGSLGRVVMLILFLALMFLYTSYSANIVALLQSSSTKIRNLDDLLHSRLKFGVHDTVFNRYYFSTATEPVRKAIYEKKVAPPGTTPRFISMEEGVKKMRKGLFAFHMETGVGYKFVGKYFDEGEKCGLQEIQYLQVIDPWLAVRKHTPYKEMFKIGMKRIQEHGLQSRENLLLYEKRPKCSGRESNFVSVSMVDCYPALLILSYGILVALFFLAFELLIHKRQTIVHRLSHCRRNSIDSRFI

>CpunIR21a

MSHSMIHLRCIVAYTLLLYYTVWCEDVEYYPSQYTIDNHNIAKRSSNYLQSTDNKWNIKEKVTQMKLRYFNDNDIKAKDNKTKRAVDPVFHGHPKTREQLWHEHFLNKSTAFDQNPSLIKLIHKITLKYLNDCIPVILYDSQVKSKESYLFQNLLKDFPVSYVHGYIDDSNNLKEPELLVPVKQCLHYIIFSTEVKSSAKVLGKQSESKVVVVARSSQWAVQEFLASPESRMFINLLVIGQSFKDDDDETMEAPYILYTHKLYTDGLGASKPVVLTSWTHGKYSREVNLFPAKMTEGYAGHRFIVSASNQPPFVFRRIKSDLDGGNPRVVWDGVELRLLSMLAERNNFSIEIKEPQEPSLGPGDAVSKEVAMGRADIGVAGMYFTSERTYGLDMSFSHSQDCAVFITLMSTALPRYRAILGPFHWHVWVALTFTYLIGILPLAFSDKHTLRHLLHNSGEIENMFWYVFGTFTNCFTFLGKNSWSKTTKITTRLLIGWYWIFTIIITSCYTGSIIAFVTLPVFPETVDTIQQLLAGFYRVGTLDRGGWERWFFNSSDPNTNKLFKKLELVPNVEAGIRNTTKAFFWPYAFLGSQAELEYIVQANFSMAKSKRAMLHISDECFVPFGVSMAFPSNSLYSSKLSGDLRRMFQSGLIYKIVDEVRWEMQRSSSGKLLSAGAGSLKIVSAEEKGLTLEDTQGMFLLLAAGFLLAASALISEWMGGIGRRCRQLRNKLPSSANSKEQLVISSPDLESEVNDGTESRLQFGTRSTSAGSRDTLDGQVINVTEENIIVHELMVEGLDSRRSSSVDLDREVQEIFERDLRRRKIVTGDSIEVSEEKREPTASKGAFGDPLS

>CpunIR4

MATGLELILSSICNATFCEPIFDNPLLGRQDSPKDVKYNDMVNEINGKHLKIATYDNRPMSWVEKGENGTIIGKGVAFVIVNILQKKYNFTYEVVVPEKNFEMGGDNPQDSLVGLANSSLVDMVAAFLPKVNKYREKVSFSYDLDEGVWMMMLKRPKESAAGSGLLAPFDNAVWYLILIAVLSFGPCITLLTRLRNKMVPDGEKFIPLSPSFWFVYGAFIKQGTNLAPEANTTRVLFTTWWIFIILLSAFYTANLTAFLTLSKFTLDIETPQDLYKKNYRWVSPEGSAVQYVVNSPNEDLYYLSRMIGTGRAEFRTVPNSQDYLPLVDGGAVLVREQIGIDELMYGDYLKKAREGVAEADRCTYVVAPNNFMTKLRGFAYPRDSKLQYFFDSILTYILQAGIIDFLEKKDLPSTKICPLDLQSKDRQLRNSDLMMTYMIMVTGLAAAVAVFIGELFIKRYICKTKDEVTKPKRKKTKFEKRLRIHTYDDSQPPPYDAIFGRNPKIKVTERAQRKIINGREYLVIDVSNGETRLIPVRTPSALLYQLDK

>PintIR1

MEIVYIFFFILLFNMACVASELSLRFVFIIENHEQDLVRQVGRGLKQAEETHPDIRITDEIVLLNREDDTESYSKLCAAVSRGVSLVIDLSWSPWPAADGLCSSAGLPLVRCQLGTQNLITALDDYLETRNATDAAFLLETESEVDKTLYELLGRSNIRVWVHAGLSRDSARSLKTMRPEPSFYVIVGSSSFVMDTYRRAVKEKLVRRDYRWNLVFTDYTTNVDVTQLVLPTVLLAVDQSECCELLARREECGCSEIQRSQHILAALLQYITETYNKLEHDLQQLPAKLDCGSAAGSELNGTRERLYRQLADDSDITNDTLFYWNADRSGVFLRSRFVLSTYSAESGRRSIATWRAGDQYRLLPGVELEPLKLFFRIGTSPAVPWTLPKLDPVTGEQMEDEDGRPLYEGYCVDLIAKLAETMNFDYQIVTPKTGGFGKKLPNGSWDGVVGDLMRGETDIAVAALTMTAEREEVIDFVAPYFEQTGILIAIRKPIRKTSLFKFMTVLRTEVWLSIVAALLLTGFMIWFLDKYSPYSARNNPDAYPYPCREFTLKESFWFALTSFTPQGGGEAPKALSGRTLVAAYWLFVVLMLATFTANLAAFLTVERMQTPVSSLEQLARQSRINYTVVEGSTVHQYFINMKFAEDTLYRVWKEITLNATSDQAQYRVWDYPIREQYGHILLAINASGPVPDAKTGFHQVNEHTDADFAFIHDSAEIKYEVSRNCNLTEVGEVFAEQPYAIGVQQGSRLQEALSRALLELQKERFLEQLTAKYWNESARQACPDADESEGITLESLGGVFIATLFGLGLAMITLAWEVFYYKRKEKNKVKSLENVEKPAFASEKIPEKKIDNFTKARKRGKTDKGKKMKKESKGVTIGDSFKPAADKISYISVFPKGDFRP

>PintIR2

MRFKLHFVFISIHLTTTTCEMTEYYPSQSLLNTKLKKLFKDNWNITEYDKSVNFFNNKAYGKIEWRHFGEQENVVKNITKRAIDPVFHGHPKTREELWNEHFLNKTKTFDQNPSLITLIHNITMTYLNDCIPVILYDDQIKSGDNYLFEDLLMDFPISYVHGYINQDNRLKEPRLLFGTEECLHFIVFLTDIMRSAKVLGKQSVCKVVIVARSSQWAVQEFLFSPLSRKFVNLLVIGQSFKDDDDETIEAPYILYTHKLYTDGLGASKPVVLTSWSHGKFSRHVNLFPMKMTEGYAGHRFVVAAAHQPPFVFRRIITDLDGGNPRIKWDGIEIRLLKLLAEKNNFSIEVIEPREPNLGSSDAVLKDIAKGRADIGIAGIYLTSERAIQVDISFSHSQDCAVFVTLMSTALPRYRAILGPFHWHVWVALTFTYLIGIFPLAFSDKHTLKHLLHNSGEVENMFWYVFGTFTNCFTFVGKNSWSKTTKVTTRLLIGRLFFIMFVYLCTKSAMIFKLIFILGWYWIFTIIITSCYTGSIIAFVTLPIFPETVDTIDQLLSGFYRVGTLGRGGWERWFLNSSDTKAKKLFKKLELVPNVESGIRNITKAFFWPYAFLGSQAELQYIVQSNFSKTSSKRALLHIADECFVPFGVSMVFPNNSLYSAKLSDDMRRVFQSGLMDKIVDEVRWDIQRSSTGKFLAVIPGFHITSAEEKGLTLEDTQGMFLLLAAGFLLAAAALISEWMGGISQRCRRKKPPSAKSQEHLIPSEIREVSNVDPRNSSAESRNTLDGEIINITEDDIMVHENFNTDVLESRRSSSVDLDKEVQEIFEKDMMRRNIIRGDTIEFDDDREPTASKENFGDRIKL

>PintIR3

MVFSTVIKLFLFYHLLHGCDAKLSPIIKDLHESKDLQLVLIDMLNGQARRHDVTCVVVICDTVYLNVFDGAMFKRILSVPMVMIVVEEYEDLLSPNFDTLESLREARMDGCNIYIILLANGLQVSRLLRFGDRYRILDTRAKYVMLHDYRLFHSDLHYIWKRIVNVIFLRYHRKITGVLKSKAWFDLSTVPFPNPIKTVFVSRRVDIWKNGRFHYNRTLFADKTSNLNGEALNVVYFDHAPSVVIMKMNDSSKVSGVEIEILNTLSQQMNFKPKLYQPNNIEVHKWGQKLANGTFSGLLGEMVNGKADVALGNLQYNPYHLELTDLSIPYTSQCWTFLTPEALTDNSWKTLILPFKLYMWIAVLLVLLITGIIFYGLAKYYMNLQELKKEKPFLESNKNVEKQEYVFEPSEKPVGLYLFGEIINSILYTYGMLLVVSLPKLPTGWSIRLLTGWYWLYCILLVVSYRASMTAILANPAPRVTIDTIKELVDSKVTCGGWGMETKKFFEKSTDETQKIGDRFELVDEPFEAANKIAKGVYAYYDNKDFLKYLSVKRKNSLINVDKAVNATINDTDIVSTDTERNLHIMTDCVVNIPISIGFHKNSPLKPLADIYLARIVEVGLVEKWLNDAMNPIRVQETNENEIKALMNLKKLYGAFIALAIGYILSLIGLIGELIHWHCVVKRDPQFDKYALDVYYAKNKKQ

>PintIR4

MFIKSRNTERKLYDVTSIPIEPKMNILYTFFSIIHLRTILGIDMITIDFIKQFVENEQAPTFLILCNICWRPDLQVTLMKNLTRSGCSSSSLDTKSKYHDHYTMFLLDAHCPGFDDVIASAISRKLFATPYRWLILDDRQHELLPDWPMYSDSDVVVAQRTEDGYKMTEIHKPSNSSSMVYYPRGYYNATTATLADTRPSRTLFRRRRDVMGAPITMSNVIQDSNSTQYHLPREDRLELQYDVVAKVCWVNVKIAFEMLNATPRYIFSHRWGYKQRGEWDGMINDLHTGNAELGTNCLVSDPQRLSVITYTDTLSSFRVRFIFRQPPLSYVSNIFSLPFSSSVWVAVAVCAAIATAAFYVTSKWEAKGGTSPSQLDGSMGDALLLTMSAVSQQGCVLEPRRVSGRIMLWIFFAALMALYAAYSANIVVLLQAPSKSIRTLAQLANSKLTIAANDVDYNHFVFKLYPDPVRVAISKRLEPEKGRGQFYDIKDGVERIRQGLFAFHSIVEPVYRQIEKTFLEYEKCDLVEVDYLNGFEPLVPVKKDSPYLELLRIVFKQIRESGIQSAVNRRMQIPKPHCSGQVAAFSSVGILDLKPVMLLMVYGTLLSVGILFVEILFGRLMKSDKLKHLKSKYLIKNTKVPLLKI

>PintIR5

MQLWVIYVSCFLLSVSGEEFPSLITANASIAVVLDRQYLGEQYQPILDDLKDYIKELARVELKHGGVVVHYFSWTAINLKKGFLAVFSIASCEDTWSLFSRTEEEELLLFALTEVDCPRLPLQSAITVTYMDQGQELPQILLDLRTTKAFKWKSAVILHDDTLNRDMVSRVVQSLTLQVEDNAVSSISVTVFKMRHEVNEYLRRKEMYRVLSKLPVKYIGENFVAIVTTEVMTTMIEVARDLGMTHTMAQWFYVISDTNSHSGNFSNLINALYEGENLAFMYNVTDNSPDCQNGILCYSQEMLNAFISALDMAVQDEFDVAAQVSDEEWEAIRPSKLQRREMLLKHMQQHISSKSRCGNCNTWRGLAADTWGATYRQFTDDQGSQPSAQEVKENHQSYINMTTSVIEQIELLQVGNWRPIDAMRYTDVLFPHVEQGFRGKELPIITFHNPPWSILQVNESGMVSSYAGLMFDIVEQLAKNKNFTIKILLPGNVKHDFSNDSSSDSMHSRSAMLAVSAIAKGQAALAASSFTVLPNPIPGINYTMAVSTQPYAFIIARPRELSRALLFLLPFTTDTWLCLGL

>PintIR6

MAAGVDLIISAVCNATFCEPIYDNPALETTHSKSRAVLNTLAKEVNGAHLKIATFNNFPMSWVERQENGTHVGRGVAFTIVDILREKFNFTYEIVVPEKNFEMGGKMPEESLVGMVNVSNVDMAAAFVPKVWQYQRKVDFSTDLDEGVWIMMLKRPKESAAGSGLLAPFTSHVWYLILVAVLAYGPCITLLTRLRSKIMPEDEQPIKLSPSFWFVYGAFIKQGTNLAPEANTTRVLFTTWWLFIILLSAFYTANLTAFLTLSKFTLDIEYPKDLYKKNYRWVAAEGSAVQYIVAAPNENLYYLNAMVKNGRAEFRSFQNHSEYLPMVKNGAVLVKERDAIEHTMYADYLQKAREGVAEADRCTYVVAPNKFMAKRRGFIYPRNSKLKPLFDSILWFINQAGLVNFLKSRDLPSTKICPLDLQSKDRQLRNSDLIMTYMIMIVGLCAASAIFIGEVVVKRYVRIKFRKPADKTKEKRKKTKFNKSSTVHYYYDSKPPPYDAIFGKSKPKLLEGRQLKVVNGREYLVVEFNGGSRLVPVRTPSAFLYNLEK

>PintIR7

NIFTISVWFKRQRKRNKGHHIEIMMKMVPTFLFLIFLTSSNADTDFQATMIADLVRSMERPSLVIATLCWPAYKKLKLYSMLDGWYQEWESFDLHLEKYAQMQSIVFLTDLNCPNVSNHLQKSLEKKYFRSPYRWLILKTTESNKENKIVPEAIYDFDIFPDSEVMVIFPVGDTSDIYFIYRVGAGEAWKTEFYGTWDVQNKVRKSSAMSLSTALRRQDLSGHEISICYVLTDSDSINHLTDEVNDYIDTITKVNFPTTNHLLDFLNANRKYLFANTWGYHVNGTWDGMTGYLVREEVEIGGSPMFFTSERMSVVEYIASPTPTRSKFVFQQPKLSYENNLFLLSFKSAVWYSCIALVFIMLLAIFVVAVWEWKKHADTFDSRNKDAGVLRPSIADIVILIFGATCQQGSPVELKGLLGRIVMLILFLALMFLYTSYSANIVALLQSSSSKIKSLEDLLNSRIKFGVHDTVFNRYYFATATEPIRKAIYEKKVAPPGSKPRFMSMEEGVKLMQKVGN

>PintIR8

MIHIAFVSPIEVLLQTIINQYLGISYCLTVVAETPINCMYPVSFTYIVPNDNLTGQMLDVSEKGCSDYIVRVREPNLFMVSFDEVNQLGNTRRSNRKIVFLPAENDEYDTNKLLDILSLKQTSFVPHLLMIVPSANISGDECQSYDLVTHNYVGSDDESSQLVNLDQWNSCTDKFHGNVNLFPHDMSNLYGKTFKIACFTYKPYTLLDIDSIEHEITGRDGTEMRVMDEFCKWVNCTVKIIRDDDHEWGEIYENRTGVGILGHVVEDRADAGISALYSWYEEYVQLDFSIPTVRTAITCVAPSPRLLASWEMPLMPFNLNMWIALIFTFIFACICLTIAKRCSSDRVGLTTFGMLITQPLIKQLVSLFRVMTAEDLKEKSFTRKLAYSIEKLPAGNFAIGDYITQEALIDLTVMQEDFYYEQSVVMLRKSSPYTEKISQFLGRLHASGLILAWETQVVLKHMNFEVQLEVRYSRARKEVEAFEPLNFRHVFQIKMKI

>PintIR11

MLNLIYKIKMANEDWHVEYYGNWEASGGLIKSMQMEVTTAMRRRNLERNVITTSLIAVDNRTKENLYDLRNVEIDVTTKANIRHIDVLYDFINATKAIKFTNTWGYFINGSWNGIVGHFVRGETDIGGTVMFINLERIRLLKFICHPTNIVVEFVFREPPLSYHSNLFLLPFTRTVWMCIAAFVLIFLFITYLNVYWESIKIDKEIMKEAPNHPALRANVSDIVVLLISAMCQQGSSIELKGGVGRLIMFLIFLSFLFLYTSYSASIVALLQSSSSHIRTLADLYNSGMELGIENLPYNIYYFTSAKDPLRKAIYQNRVAAKGRKPNILSIEEGVKRVQTVSTYLSGILM

>BmorIR87a

MTTGNSDQIAKTAECVLKLSAKYFVERKALSGSIVIINVNSYSSTTQGLLLKTIHSSIKYSVMAKDSFYPHANASHFPEKAKNYMLILEERTELKRNIFQLNKLPSWNPLAKAVVFYQIKGNESAQRIAIEFINELREHKFFRSIIFINNGTESGVTSYTWRPYSENNCGGKCDSVYVLDRCKNNIVEQIEPQPEWFPSNMNGCPLTTYAIVSEPYVMPPIRKIPNAKFDDVYEFQKGGETNLVKTIAEFSNMTLIVRLSAIEENWGIIYANGTATGAYGVLRNDSVDIVFGNIEVTKQIRKWFHPTISYTQDEITWCLPKAGQASAWDNLVIIFQWTIWVATFTSLILMGLLFHYMYYREKNKKITKWPTNSLLMTFSMLLGWGSHFEPKTATFRILIFGWLCFSINMGISYESFLRSFLMHPRFEKQIATESDLIQSGIRFGGREIYRTYFESNDASSSYLHTEYSSTTFSEGIRRAALNRDFAVVSSRRQAEYQDQKLGKGASLIYCFPESDNLYKYSVVLLARKWFPMLERFNGIIRSVSENGLINKWNDEMFIHRVSLEGASTIVPLSIQHLLGAFMFIGFMYGTSAFIFLVEVFVGFVQRRAFLSAFFCGKKKRFSAVFKVKV

>BmorIR7d.1

FNLPLKLTILSLFLGIILLNMLRKTIFFNNIRRVCNITPPKRNSLFYAWLLFLGLPLEKFSSRKHFKIIILAWIWFSFVIRCAYQVTLVTSLKSITYNYNLRYDSDILKYPFGGMSSIRDYFIEDKDFYENWTSVDMQKAYKLLDEIMEEKTDFVLALNKDTILHHAAEHIGSKRIQVIDNCIVNSPIVLYFRKHSPMTDPIAKIMNAALECGFIQYSYQTNWKRQKHLLNSHYAYNLQPLTLDNFSGCFFLLIIGYGISILYFVLEVVCHKIDKTNQRIDLRVDQE

>BmorIR41a

WINCTVQGVIGSVVEGRSDFGIAALYSWYEEWKAMDFSVSVVRSAVICLVPAPRVWELPFLPFKSIWIAVVITFVYASIGLTIAQGCKLLIVFGTIISQSQYIVSDSWRIRSVIGWLLVSSLILVSAYGAGLASTFTVPPSIDTVQDLLNSRMEWGFEELQRRSAFSLEQDMQMFYFDCVAMLHKNSPYTEKLSELIGRLHQSGLLWESQVSLNFNHVEGIFLIFITGTILSTLFFALE

>BmorIR68a

KMNFRPKGLLGEMVNGRADLALGNLQYTPYHLELIDLSIPYTSQCWTFLTPEALTWKTLLLPFLYMWIAVLLVLITGTIFYGLARYFGNILYTYGMLLVVSLPKLPTGWSIRFLTGWYWLYCILLVVSYRASMTAILANPVTIDTLVELAASKLTCGPNIAADKVAYYDNRNLHMVVNIISIGFHKNSPLKPLTDIYITRIVEVGLVWLNDAMMNLKKLYGAFIALAIGYFLSVMCLIGE

>BmorIR21a

KNNFSIEAVAKEIAKGRADIGVAGMYLTIDRTREMDVTFAHSQDCAVFITLMSTAYQAILGPFWHVWVALTLTYLFGMFPLAFSDKHSGNFWYVFGTFTGRNSWSKTDKITTRLLIEMVLDFTIIITSCYTGSIIAFVTLPETVDTIHQLLAGFYRVGVEAGIMNTAFLGSKVLHSFVPFVTIGFPNNSLYTAKLNNDLRRMVQSGIVIVDEVRLTLEDTQGMFLLLAAGFLIAATALISE

>BmorIR64a

MYNITYDGMVGSLQRHEADVGGSPIFFKTDRAYVVDYVAETWPSKQSFIFRHPKHHTVYSRPLNSVWYCVIAFLFVTASTVFFMLKFETLFLFAWSAICQQGMSLRRNSLALKVVVFVTFVCSITLYQYYNATVVSTLLKEITIRTLKDLLQSDLKVGPEYGMSLVAFHVDCEIHVYPPQMGAVLKKNSPYRNYFAIGIRRLWETGLMMKHIWDVSILEFSTPLFIVVFGVIASVVVLLCE

>BmorIR75p

MLNATPRGMINDLHTSKADLGTNCVVSDVERLSVVTYTDMLAPFRVRFVFRQPPLANIFYLPFGRVWAAVAVCAMVYTAAIYWASKWDGDMLLTMSALSQQGCFIEPKRAPGRIMLFVLFTALMALYAAYSANIVVLLQAPNSITSLAQLAASKVTLALEDGVDMIAFHSICDLTVLSSFPFVPVKKDSPYLELLRVSFKQIRESGIQLNRRYQVGIVDLRPVLIMMIYGIISSCLILIME

>BmorIR75q1

FMNASHKGMMGDLAKGTVDFGGTIAFLTSQRLQVVDYLSSPVPINAKFVFREPPLNNLFLLPYANVWYCTAAFVVLLVIILYINAKWQPDTILVISAISQQGSSNELKGTLGRAVLFLLFLTFLFLYISYSANIVALLQSNKQIRTLQDLLNSNLNIGIEEGVKKLAFNMNCGLQIIESSPWMSCRKNSPFREIYKLGLFKLQEHGITENRLLFVNMVDVYPVILMFLYGLFLAFLILLVE

>BmorIR75q2

FLNAERKGLTGFLVNGDVEIGGSPMFFTAERTAVVDFISSPTPTRSKFVFQQPKLNNLFLLSFTAVWYSTLALISLIFTMLLSVTAWRPDTMLVFGATCQQGSTVELKGSLGRVVMLILFLTLMFLYTSYSANIVALLQSSSQIKTLEDLLHSRLKFGMEEGVKKMAFHMECGLKILQVIPWLAVRKNTPYKEMFKIGMKRIQEHGLQENRLLYVSMVDCYPALLVLSYGIIIAIALVIME

>BmorIR40a

KLNFRYRGTLGLIWKRQADFFLGDVTMTWERLQAVEFSFLTLADSGAFLTHAPAKTLAIIRPFWEVWPLVCATLFITGPALWIVIAAMGNCWFTVTLFLRQSSTKPSSTHKARLVTVLISLATYVIGDMYSANLTSLLARPPPIGTLPALEEAMREHGVEAGVRLVAVLGGHNFHLLYTRSAIAFQIGSPYLETINNVVMTLFEAGILMTTDEYVSLTMLRGAFCLLGIGHLLAGVTLLIE

>BmorIR76b

KFNFTYESLIGLTNTSKVDMIAAFIPRLVRFRKLVTFSRDLDEGVWMMMLRRPKEGSGLLAPFNFVWYVTLASVLCYGPCICFLTHVPLPFWFVYSAFIKQSTNLAPEANTTRVLFATWWLFIILLSAFYTANLTAFLTLSLDIETPEDLYKKNYRWVPDQEYLPIAVLVKCTYVAFMKKRAFVYPVGSKLKSLFDPTLAYILQSGIILEHKDLLTNSHLMMTYYIMCVGLASGLAVFVVE

>BmorIR93a

NKNFTIRYNNIPLYFRAVFIHQAGVNLKNNYYRCINYTIPVSTQPHTFIVARPREALLFLLPFTDTWLCLGFAVILMGPMLYIVHRLLANLWYIYGALLQQGGMYLPRADSGRLVIGTWWLVVLVIVTTYSGNLVAFLTFPAPVTTISELLKNSYTWSARGTLDRVLIFDWCDFAAFMEEVAMIVPAGSPYLPVINKEINRMHKAGLIWLSAYLVNLSDMQGSFFVLFLGNDKIVYMYIAE

>BmorIR8a

AMNFDYEGVVGDLTTGETDIAVAALTMTAEREEVIDFVAPYFEQGILIAIRKPIRLFKFMTVLTEVWLSIVAALVLTGFMIWLLEKYDFEFWFALTSFTPQGGGEAPKALSGRTLVAAYWLFVVLMLATFTANLAAFLTVETPVSSLEQLARQSINYTAETGFKQVAFIHDCNLTVFAEQYAIAVQQGSRLQEDISRALLELQKERFLLTSKYWITLESLGGVFIATLFGLGLAMITLAWE

>BmorIR25a

IVKFDYEGIIKELIEKRADIALTSLSVMAERENVVDFTVPYYDVGITIMMKLPRTLFKFLTVLNDVWLSILAAYFFTSFLMWVFDKWEFELWFCMTSLTPQGGGEAPKNLSGRLLAATWWLFGFIIIASYTANLAAFLTVSTPIESLDDLSKQYIQYAIEEAVQRVAWLGDCDLQVFSRKYAIAVQQGSPLKDQFNNAILQLLNRRRLLKENWWISIQNIGGVFIVIFMGIGLACITLGVE

>EhipIR68a

MHYLDMLKLKLAIFMFLSIEVSTDIAPILKQLQERRDLEYVVTDLVNVVTRYDDVTCIAIICDQVYLNVFEGTLFRRTLAVPYVMIVVEDYEDLLSPNFDTLQSLRETRKVGCNIYIILLANGIQASRLLRFGDRYRILDTRAKYIMLHDFRLFQSDLNYLWKRIVNVIFLRYHKKIVGVLKSKAWFDLSTVPFPNPIKGVFVSRRVDIWKNGKFHYNRALFADKTRNLNKEVLNVVYLDYVPSVVVIKDNDTSKIGGVEIEILNMIAEKMNFKPKLYQPMNVELHKWGQKQPNGSFSGLLGEMVNGNADVALGNLQYTPYHLDLTDLSVPYTSQCWTFLTPESLTDNSWKTLILPFKLYMWIAVLLVLLVTGMIFYGLARYYINLMAYKHDPSDIGVSVPLSSKGKHEEVDQFDEKPVGLYLFGEIINSILYTYGMLLVVSLPKLPTGWSIRLLTGWYWLYCILLVVSYKASMTAILTNPAPRVTIDTLTELVESKVTCGGWGSETKKFFENSLDDAGQKIGQRFQTVDDPNEAAKRVAQGHYAYYDNKYFLKYLSVKRRNVFMNMEIEMDNSTMNGTEVTVRTETERNLHIMTDCVVNIPVSIGFHKNSPLKPLADIYIRRIVEVGLVEKWLNDAMHPIKSLEAEDEEIKALMNLKKLYGAFVALAIGYFLSAVCLIGELIHWYLIVKKDPHFDKYAL

> EhipIR75p2

MSMLSFLLLPIPCIISRELQYDAFTKICWINIKLAFQMLNATPRYIFSHRWGYRRNGQWSGMVDDLLSGRADVGTNLLMTVDRLDVITYTEGLSPYRVRFIFRQPPLSYVANIFSLPFSSGVWIATVVCAIVSTIALYLASKWEVAIGKSPTHLDGFGDSLFLTMSAVSQQGCIMEPKKISGRIIMLFVFVSLMALYTAYSANIVVLLQAPSNSIRTLSQLARSKVTIAANDVDYNHIVFKLFKDPVRVSIQKKLEPENGKAQFYDMNEGVERIRQGLFAFHSIVEPVYRRIEKTFLETEKCDLTEVDFLNSLDPFTPIKKHSPYTELLRVVIKQIRESGILSAVYKRLQVPKPRCTEKVSAFSSVGLLDLRAVMFLMLIGAAVSIGVMFIEIIFHKLNKRQRGI

>EhipIR21a

MELLLNILILKFLFYAHGQEIEYYPSQNVLDNSVVKIGSNSKQQLINTEYNELFSKNAHDKIQWRYFNENESDKIKNISKRAADPVFHGHPKTTEELWNEHFLNQSSAFDQMPSLIKLIHNITLTYLSDCIPVILYDKQVKSQESYLFQDLFKDFPVTYVHGYINDDDTLKEPKLLFSDQNCLHFIVFLTDVKTCTKVLGKQSQSKVVVVARSSQWAVQEFLASPLSRVFVNLLVIGQSFKDDDDDSLEVAYILYTHKLYTDGLGASRPVVLNSWSHGKYSRKVNLFPKKMRKGYAGHRFLVAAANQPPFVFRRIKRDEESGNLKVVWDGIEVRLLQLLADRNNFSIEIVEPRELNLGPGDAVAKEIATGRADIGIAGIYLTQDRIREMDVTFAHSQDCAAFITVMSIALPRYRAILGPFHWHVWLALSFTYIFAIFPLAFSDKLTLRHLMHNGGEVENMFWYVFGTFTNCFTFVGKNSWSKTTKITTRLLIGWYWVFTIIITSCYTGSIIAFVTLPVYPETVDSVRQLLSGFYRIGTLDRGGWERWFLNSSDKYTNKLFKKIEFVPSVEAGIRNTTKAFFWPYAFLGSRAELEYIVQSNFSMTKSKRGLLHISNECFVPFGVSFAFPNNSLYTAKFSNDVRRMLQSGIIQKIVDEVRWEMQRSSTGKLLSAGIGSLNTLSIEEKGLTLEDTQGMFLLLGAGFLIAASALISEWIGGCSRLCRLNRNKNPPTSVNSGDHLIPTPKTDTQSTINIISDGADSRLHFDTRPPSADSRDTLDGQIINVTEENITVHDNSKLDGWDSRRSSSIDLDREVKEIFEKDQKRRRILSDGMIELSGNKRHPTASKGAFGDTVGN

> EhipIR76b

MELIISSICNATFCEAVYDNPLIESQLTKTQIELLALAEELNGKHLKIGTYNNYPLSWTERADNGTLIGGGVAFTIIDILREKFNFTFEVVIPNNNFEFGGSRPEDSLIGLVNSSKVDMVAAFMPTLYKLKDLVSSSVDIDEGVWVMMLKRPAESAAGSGLLAPFESHVWYLILTAVLSYGPCITLLTRLRSKLIKDHERYIPLSPSCWFVYGAFIKQGTTLSPEANTTRVLFATWWLFIILLSAFYTANLTAFLTLSKFTLAIETPRDLYKKNYRWVATQGGTIEYAVRDPDEDIHYLNQMISNGRAEFRSVSNSEDYLTMVKGGAVLVKDRTGIDHMMYADYLNKARQGIEETSRCTYVIAPNSFMKKNRAFIFPKNSKLKKLFDPILTNLLQAGIVNFLKNRDLPSTKICPLDLQSKDRRLRNSDLMMTYLIMVIGLAAAIAVFIMEIIIKKCFHIRLKTEGPRPKRTRANRVRFQNHDETQPPPYESLFGRNSRYKMTDQFQTKIINGREYWVVDTVSGDTRLIPMRTPSAFLYQRQRRM

>EhipIR31a

MSIANILLLILAGVPTYWTTMLVQAIADFFKYKIINTVIVLSCWSSNDRVKFMRQLSDHGLIATISCDPTILDYVQNHHFQGILYVKQVNDSLLEKVNPVYFSNWYKWLVISDEAPSSLHATRYDADVVLIESSKRIMARATGNNEIMSTAAIKQTIYFNDVYVHPRNGASLNPWAVWTGTLEVTHERERILRRLDLKKYPLRIATPVGHYSEDTYNGTFEEYLADNTMPERDSATRCGHAASSLILESLKATEVLTPTLLWATELNNSSMMLRVASGTAEISGSILRVLPERIKRLDYVMPIWPFSVGFTYLAERASSSNMFVEPFSPGVWWTCLAIAVLLSFAQRLTAREPMEKEGAYIAVLATWLQQDASAVPEGASGRWTFIVLSVCSMLVHAYYTSAIVSALMSTGRSGPDSLKALGDSKYAIASEDYDYMRYTMFGMETNWDDLEYLKKKKMHSNFYQNIERGVELIREGNTAFHTEYNHIYPHLRTFNDEHLCKLAYVDTIPEIMTWITTTKRCQWTDVLRTAGGWLNEVGLVKRLVSRWRIRPPPCRASLLAERVKFGDVAPVLCLTAIGAIASLILLGLEIIFAKWTGSKYRNSPVSDVADVASGDENINK

>EhipIR75q1

MKVIVVMFTILFGTKLVTSSLNKNNELAMIVDVIKSYDKPTFVIANVCWPTLQKVKLATELSDTETPKMVQFINNNMITEDFNHKENVFFVIDTNCSFIEDFFTQANNSKKFNAPYRWLILEKPSNESSDVLLRLNHLNILTDSEVIISRNRSNGSFVFHMIYKIKSKSEWRLEFFGTWTTTNGLRKLNNMVTVPISMRRKNLLGASIVTSLVITNNKTKGNLYDTRDIEVDGVSKTSYRQIMPLYYFMNATRVLTFPDAWGYYINGTWNGMIGDVVSGNAHLAGSVMFITRQRIDFLDYLIHPSPGLTVKFLFREPPLSYQNNLFLLPFKLNVWLCIAAFVIILTLILYVNALWETQKTEIIYTNNLDHTTLRPNVSDIAFLVISAISQQGSAMELKGTLGRIVTFILFLTFLFLYTSYSASIVALLQSSSKQIRTLSDLLHSKLELGAEDTPYNRYHFSTAKEPVRKAIYQKIAPPESKPNFLNLEDGIKKLQKVCFILRNFIVNIENIIRSENFTI

>EhipIR25a

MGPHKEILSSLMLLILLFIRNSFSQTTQNINVLLINEENNALAERAFEVAKEYVRRNPSLGLAVDPVIVVGNRTDAKAFLENVCRKYNDMLSVKKTPHVVLDFTMTGVGSETIKSFTAALALPTVSGSFGQAGDLRQWRNLSPNQTRFLLQVMPPADILPEAIRAIVTKQDITSAAIIFDEFFVMDHKYKSLLQNIPTRHVITPVKSFNKDEIKSQLRSLRELDIVNFFIVGSLRTIKNVLDAADDNQYFGRKTAWFALSLDKGDINCGCKDATIVYMRPTPDAKSRDRLGKIKTTYSMNGEPEITSAFYFDLSLRTFLAIKSLLDSGKWPNDMKYITCDDYDGKNTPNRTLDLKTAFQEVKEPPTYAPFYIPEDDPMNGRSFMEFSTDLSAVTVKDGASIGSRSLGSWKAGLASPLSLTDPENMSDYSAQLVYRVVTIEQQPFIIRDDSAPKGYKGYCIDLIEEIRQIVKFDYEISLTPDGNFGTMDENGNWNGLIKELIEKRADIALASLSVMAERENVVDFTVPYYDLVGITILMKLPRTPTSLFKFLTVLENDVWLSILAAYFFTSFLMWVFDKWSPYSYQNNREKYKDDEEKREFTLKECLWFCMTSLTPQGGGEAPKNLSGRLLAATWWLFGFIIIASYTANLAAFLTVSRLDTPIESLDDLSKQYKIQYAPLNGSAAMTYFERMAHIEVKFYEIWKDMSLNDSLSDVERAKLAVWDYPVSDKYSKMWQAMKEAGLPNTVEEAVQRVRDSTSSSEGFAWLADATDVRYQVLTSCDLQMVGDEFSRKPYAIAVQQGSPLKDQFNNAILQLLNKRKLEKLKETWWNNNPAAMKCEKQDDQSDGISIQNIGGVFIVIFMGIGLACITLGVEYWWYKWRKRPIIGDVTQVDPAKSTRNNVDNFGNTKTGEGFTFRARNLGLSNFRSKF

>EhipIR41a

MLTLQPNVFHIELLLHTILNQYLYNSYCVTFVSEIPLDINFSITFTCIVPDPENLTNQLLEVSEKGCSDYVVRMKEPQEFMNAYEKVNHLGNARRSDKKLIFLPFLEDDNTTNETISPLLNLLSMKETSFVANILLLIPSRETTAECKFYDIVTHKYVGPDKETNHPLYLDRWNSCTEKFVMNVNLFPHDMSNLYGKTVKVACFTYKPYVLLDLDTPTGRDGMELRIVEEFCRWVNCTMEVVRNDAHEWGEIYDNQTGVGVLGNVLEDRADLGITALYSWYEEYVVLDFSAPCIRTAVTCVAPAPRLLASWELPLMPFTWHMWIALIFTFLFSSVALIVVKGFSSKNVFITTFGMMVTQCQPEVRADWRVRSITGWLLITGLVFDNAYSGGLASTFTVPKYETSIDTVQDIVDRKMEWGATHDAWTFSITLSTEPLIKQLVSQFKIYSAEELKRKSFTRNMAFSIEKLPAGYFAVGEYITKEAMLDLEIMLEDFYYEQCVVMLRKSSPYTSKISDLVGRLHESGLMLAWETQVALKYLNYKVQLEVKLSRSRRDVDNIEPLSFRQVVGIFIIYLIGVSLSIVIFVAEMCINDKNKKKLNQ

>EhipIR8a

MEMFSLFLLILLLNLGCVASELSLRFVFIIEVHETDVAQQIGRALKAAEEKIPELRVDDSIVQLDRENEDQSYRILCSALSKGVSMVIDLSWSPWEMAEQLTSDTGLPLVRTLLGSQQLLVALDEYLETRNATDAALLLESEGDVDRTLYQLLGESNIRVWVHAGLTRDSARALKTMRPDPSFYAIIGSAAFVTDTYRRAVKEKLVRRDYRWNLVLTDYSGANLDVSELVLPTMILHVDPSECCKLMGRRDGCMCPVELERKQYILNSLIQYISETYTKLEKDLSLISIRVDCDNVQGDMNGTRDRLFRHFAEDADVNNETLFYWDSDRSGLFLRSRFVLSTYKPDEGLQTAATWSADEEYRLLPGVILEPLRLFFRIGTTAAVPWTMAKLDPESGEPMYSEDGQPLYVGYCVDLIEKLAEQMGFDFEIVTPKYGTFGRKLPNGTWNGVVGDLMRGETDMAVAALTMTAEREEVIDFVAPYFEQTGILIVIRKPTRKTSLFKFMTVLRTEVWLSIVAALVLTGVMIWLLDKYSPYSARNNPTAYPYPCRKFTLKESFWFALTSFTPQGGGEAPKALSGRTLVAAYWLFVVLMLATFTANLAAFLTVERMQTPVSSLEQLARQSRINYTVVEASTIHQYFINMKFAEDTLYRVWKEITLNATSDQAQYRVWDYPIREQYGHILLAINASGPVPDAKTGFQQVNEHTDADFAFIHDSAEIKYEVTKNCNLTEVGEVFAEQPYAIGVQQGSRLQEDLSRALLELQKERFLEQLASKYWNESARQACPDADESEGITLESLGGVFIATLFGLGLAMITLAWEVFYYKRKEKNKTQTMDANIERRQAFVEPKKKEKKHGVARLRKRDKKVGNIGRSVTIGDTFKPAVNKSNVSYISVYPKGGFQP

>EhipIR75q2b

MFFTSERIAIVDYISSPTPTRSKFVFQQPKLSYENNLFLLSFRSTVWYSTLGLVILLFLALFVVAMWERKRDQLKEKDADILRPSLADVALLIFGAACQQGSPVELKSSLGRVVMLILFLTLMFLYTSYSANIVALLQSSSSQIRTLEDLLHSRIKFGVHDTVFNRYYFSTATEPIRKAIYQTKIAPPGTKPQFMTMQEGVKKMQQVNYLYISLCPTYIYV

**SNMP:**

>SinsSNMP1

MKLPKHMKIAMGAGGAAVFGVLFGWVIFPVVLKSQLKKEMALSQKTDVRQMWQKIPFALDFKIYLFNYTNPEEVQKGGIPIVKEVGPYHFDEWKEKVEVEDHEEDDTITYKKLDVFYFRPDLSGPGLTGEEIIVMPHAFLLSVVTIVSRDKPSMLNMIGKAINGIFDNPQDVFMRVKAMDILFRGVVINCARTDFAPKALCTALKKEAVSGLVIEPDNMHKFSIFGTRNGTVDPHVVTVKRGVKNVMEVGQVVAIDGKTQQDKWKDSCNEYEGTDGTVFPPFLTESDRLQSFASDLCRSFKPWYQKKTSYRGIKTNRYVANIGDFANDPELQCYCESPSQCPKKGLMDLTKCIGAPMYVSMPHYLESDPELLQNVKGLTPDINAHGIQIDFEPITGTPLVARQRIQFNLQLLKNDKLDLFKDLPDTIAPLFWIEEGLALNKTFVNMMKHQLFIPKRVVGVVRWLLLSFGILGVLGSVVFHFKGRGVQTTA

>SinsSNMP2

MIGKHSRLFFGISLVVLVVAIILAAWGFPKIIQNQIQKNIQLDNSSAMFEKWRKMPIPFDFKIYVFNVTNAEDVNNGAKPKLAEIGPYVYKEYREKTILGYGPNDTIQYTVKKRFVFDNNASAELSEDDEVTVINFSYMAAILSVYELMPSMIGMINKALEQFFPNLTDPFLRVKVKDLFFDGIYLNCVGDNSALGLVCGKIKADMPPTMRPSEDGNGFYFSMFSHLNTTEIGPYEIVRGRVNMYDLGHIVSYKGKTSMSQWGDPYCGQINGSDSTIFPPIDENNVPRRLYTFEPDICRSMYVGLIGKRSLFNLTTYYYEMTDALAAKSANPDNKCFCKRNWSGNHDGCLLMGVLNLMPCQGAPAIASMPHFYLASEELLEYFDEGIQPDKEKHNSYVYIDPVTGVVLKGLKRLQFNIELRNVKDAPQLENVPTGLFPLLWIEEGAEIPESIQNELRQSHSMLGYVETARWMLLVIGIALTIISAVILARSSSILGWPRNSNSVSFILRSGVSASVRNKSY

>EhipSNMP1

MDKYSNSCLLIRTAAVRYYSLKMKLPKHMKIAMGAGGAAVFGVLFGWVIFPVVLKSQLKKEMALSQKTDVRQMWQKIPFALDFKIYLFNYTNPEEVQKGGIPIVKEVGPYHFDEWKEKVEVEDHEEDDTITYKKLDVFYFRPDLSGPGLTGEEIIVMPHAFLLSVVTVVSRDKPSMLNMIGKAINGIFDNPQDVFMRVKAMDILFRGVIINCARTEFAPKALCTALKKEAVSGLVIEPDNMYKFSIFGTRNGTVDPHVVTVKRGVKNVMEVGQVVAIDGKTQQDKWKGSCNEYEGTDGTIFPPFLTESDRLQSFSSDLCRSFKPWYQKKTSYRGIKTNRYVANIGDFANDPELQCYCESPSQCPKKGLMDLTKCISAPMYVSMPHYLESDPELLQNVKGLTPDINAHGIQIDFEPITGTPLVAKQRIQFNLQLLKNDKLDLFKDLPDTIAPLFWIEEGLALNKTFVNMMKHQLFIPKRVVGVVRWLLLSFGILGVLGSVVFHFKGRGVQTTA

>EhipSNMP2

MIGKHSRLFFGISLVVLVVAIILAAWGFPKIIQNQIHKNIQLDNSSAMFEKWRKMPIPFDFKIYVFNVTNAEDVNNGAKPKLAEIGPYVYKEYREKTILGYGPNDTIQYTVKKRFVFDKNASAELSEDDEVTVINFSYMAAILSVYELMPSMIGMINKALEQFFPNLTDPFLRVKVKDLFFDGIYLNCVGDNSALGLVCGKIKADMPPTMRPSEDGNGFYFSMFSHLNTTEIGPYEMVRGRVNMYDLGHVVSYKSKTSMSQWGDPYCGQINGSDSTIFPPIDENNVPRRLYTFEPDICRSMYVGLVGKRSLFNLTTYYYEMTDALAAKSANPDNKCFCKRNWSGNHDGCLLMGVLNLMPCQGAPAIASMPHFYLASEELLEYFDEGIQPDKEKHNSYVYIDPVTGVVLKGLKRLQFNIELRNVKDAPQLENVPTGLFPLLWIEEGAEIPESIQNELRQSHSMLGYVETARWMLLVIGIALTIISAVILARSSSILGWPRNSNSVSFILRSGVSASVRNKS

>SlitSNMP1

MLLPKELKYAAIAGGVAIFGLIFGWVLFPTILKSQLKKEMALSKKTDVRQMWEKIPFPLDFKVYIFNYTNAEEVAKGAVPILKEIGPYHFDEWKEKVDVEDHEEDDTITYKRRDVFYLNPELTAPGLTGEEIVVIPHVFMLGMALTVQREKPAMLNMVGKAMNGIFDDPPDIFLRVKAMDILFRGMIINCARTEFASKATCTALKKEAVSGLVLEPNNQFRFSIFGTRNNTIDPHVITVKRGIKNVMDVGQVVAVDGQTEQTIWKDTCNEYQGTDGTVFPPFLTENDRLQSFSTDLCRSFKPWYQKKSSYRGIKTNRYVANIGNLAEDPELQCFCPQPDKCPPKGLMDLAPCIKAPMYASMPHFLDCDPALLSKVKGLNPDVNAHGIEIDFEPISGTPLVARQRIQFNIQLLKTDKLDLCKDLSGDIVPLFWIEEGLALNKTFVNMLKHQLFIPKRVVGVLRWWMVSFGSLGAVIGIVFHFRDHIMRLAVSGDSKVSKVTPEEVEEQKDISVIGPAQEPAKINI

>SlitSNMP2

MLGKHSKLIFAVSMGFLVVAVIMAAWGFQKIVDKQIQKNVQLENNSMMFDKWLKLPMPLEFKVYIFNVTNVEDVNQGEKPILNEIGPYVYKQYRERTILGYGPNDTIKYMLRKRFEFDPEASGVLTEDDEVTVINFSYLAAVLTVHDMMPSFVGMVNKALEQFFPSLEDAFLRVKVRDLFFDGIYLNCDGDNAALGLVCGKIKSDTPPTMRPAEGANGFYFSMFSHMNRTETGPYHMIRGRENVYELGNIVSYKEQKVMPMWGDKYCGQINGSDSSIFPPIKEGNVPKKLYTFEPDICRSVYVDLVGKKEIFNISAYYYEISESAFAAKSANPNNKCFCRKNWSANHDGCLLMGLLNLMPCQGAPAIASLPHFFLGSEELLEYFGSGIMPDKEKHNTYVYIDPTSGVVLSGLKRLQFNIELRQIDTVPQLKRVPTGLFPMLWLEEGATIPASIQQELRDSHKLIGYVEVARWFLLTAAIIAVVTSAVAVARANALLSWPRNSNSVSFILGPSVTQVNKGN

>CsupSNMP1

MQLPKHLKIGAGTAAAGVFGIIFGWVLFPAILKSQLKKEMALSKKTDVRGMWEKIPFALSFKVFLFNYTNVEEIQKGGVPIVKEIGPYHFDEWKEKLEVEDHEEDDTITYKKRDVFYFRPELSGPGLTGEEIITMPHILMVSIATVVNKEKPAMLNMIGKAFNGIFDGPQHVFMNVKALDIMFRGTIINCARTEFAPKAVCTAIKKEASGLIIEPNNQFRFSLFGMRNDTIDPHVITVKRGIKNVMDVGQVVAVDGNPEQSIWRDSCNMYEGTDGTVFPPFLTENDRLESFSTDMCRSFKALYQKKTSYKGIKTNRYVVTIGDLANDPDLQCFCEAPEKCPPKGTMDLMKCMNAPMYASLPHYLDCDPEVQKKVKGLNPDVNVHGIDIDFEPISGTPMVANQRMMFSLVLQQIDKLDLFKDLPGTMTPLFWIEEGIALNKTFVKMLKNQLFVPKRIVGALRWLLVAVGVCGVIVTGIIHYKGSILGFTLPRGSATVAKVNPETNQPKDISVIGNAQSPPKVDM

>CsupSNMP2

MLAKHMKVFFLASLAALVLAVILAAWGFPRIVSKQIQKNVQLENSSVMFEKWRKLPMPLTFKIYVFNVTNAEDINSGAKPMLTEIGPYVYKEYRERTILGYGENDTVRYTLKKTFIFDAEESGPLTENDEVVVINFSYMAAILAVQEMMPSLTTVVNQALEEFFTDLKDPFMRIKVRDLFFDGIHVNCVGNHSALGLVCGQLKSDTPPTMRPTEDGTGYYFSMFSHMNRTESGPYDMVRGTEDIRELGHVVAYKGERSMSQWGDPYCGQLNGSDSSIFPPIDGGNVPQRLYIFEPEICRSMFATLVGKTTVFNMSAFHYSISSDVLAARSANPNNKCYCRKNWSANHDGCLLMGVMNLAPCQGAPAIASLPHFYLASEELLQYFASGINPDKEKHDTYLYLEPVTGVVLKGLRRFQFNIELRNIPEVPQLAKVPTGLFPLLWIEEGATLPDSVVKELQSSHKLLSYVEAARWILLVVAVIATVVSAVTLARSGVLPVCPRNSNSVSFILNPHPTVIDVNKVH

>OnubSNMP1

MQLQKPLKIGLGMMGAGLFGIIFGWVLFPVILKSQLKKEMALSKKTDVRAMWEKIPFALDFKVYMFNYTNVEEIMKGAAPIVKEIGPFHFDEWKEKVDIEDHDEDDTITYKKRDYFYFRPDKSGPGLTGEEVVVMPHLLMLSMATIVNNDKPAMLNMLGKAFNGIFDEPKDIFMRVKVLDLLFRGIIINCARTEFAPKAVCTALKKEGATGMTFEPNNQFRFSLFGMRNGTIDPHVVTVRRGIKNVMDVGKVIAIDGKTEQDVWRDKCNEFEGTDGTVFPPFLTEKDNLESFSGDLCRSFKPWYQKKTSYRGIKTNRYVANIGDFANDPELQCYCDSPDKCPPKGLMDLMKCMKAPMYASLPHYLDSDPQLLKDVKGLSPDANEHGIEIDFEPISGTPMVAKQRVQFNIILLKADKMDLIKDLPGTMTPLFWIEEGLALNKTFVKMLKNQLFIPKRIVSVVKWLLAGVGFVGLVGSVVYQFKGKMINFALSPSSAPVTKVNPEINQQNQPKDISIIGESQNPPKVDM

>OnubSNMP2

MLGKHTKLFFGVSLVALIVSVILAAWGFPKIVSKQIQKNIQIDNSSVMFEKWRKIPMPLTFNVYVFNVTNVEDVNNGAKPRLQQIGPYAYKEYRERTVLGYGDNDTVSYTLKKTFIFDQEASGLLSEDDEVTVIHFSYMAAILTVNDMMPSITGVVNGALEQFFTNLTDPFLRVKVKDLFFDGVYVNCAGNHSALGLVCGKLKADAPQTMRPAGDGNGFYFSMFSHMNRTESGPYEMIRGRENIKELGHIISYKGKSFMKNWGNDMYCGQLNGSDASIFPPIDENNVPEKLYTFEPEVCRSLYASLVGKSSIFNMSAYYYEISSDALASKSANPGNKCYCKKNWSANHDGCLIMGILNLMPCQDAPAIASLPHFYLASEELLEYFDGGISPDKEKHNTYIYLEPVTGVVLKGLRRLQFNIELRNIPMVPQLAKVPTGLFPLLWIEEGAELPDSIIQELRQSHTLLGYVEAVRWALLAIAIVATAISAIAVARSGLIPVWPRNANSVSFILSPHPNSDVNKVH

>SinfSNMP1

RLQSFSTDLCRSFKPWYQKKTSYRGIKTNRYIANIGNFAEDPELQCFCPEPDQCPPKGLMDLAPCIKAPMYASMPHFLDCDPSLQNNVKGLNPDVNQHGIEIDFEPISGTP

>SinfSNMP2

MLGKHSKLMFAVSMGFLVVAIILASWGFEKIVDKQIQKNVQLENNSMMFDRWLKVPMPLDFKVYVFNVTNAEEVNQGEKPMLKEIGPYVYKQYREKTILGYGPNDTIKYMLRKRFEFDAEASGGLTEDDEVTVIHFSYLAALLTVHDMMPSLVGVINKALEQFFPSLEDAFLRIKVRDLFFDGIYLSCDGDNAALGLVCSKIKGDMPPTMRPAEGTNGFYFSMFSHMNRSETGPFEMKRGRENVYELGNIVTYKDQHTMKVWEDKYCGQINGSDSSIFPPIKEGNVPKKLYSFEPDICRSLYVDLVGKREKFNISSYYYEISESALAAKSANHDNKCFCKKNWSANHDGCLLMGLLNLMPCQGAPAIASLPH
