# Supplementary material for: Antennal transcriptome analyses and olfactory protein identification in an important wood-boring moth pest, Streltzoviella insularis (Lepidoptera: Cossidae)
Source: Sci Rep. 2019 Nov 29;9:17951. doi: 10.1038/s41598-019-54455-w (PMC6884542; doi:10.1038/s41598-019-54455-w)
Supplement: Supplementary file 11 — Supplementary Table S11 [file 41598_2019_54455_MOESM11_ESM.docx]

**Supplementary Information for**

**Antennal transcriptome analyses and olfactory protein identification in an important wood-boring moth pest, *Streltzoviella insularis* (Lepidoptera: Cossidae)**

**Yuchao Yang^1^, Wenbo Li^1^, Jing Tao^1^*, Shixiang Zong^1^***

^1^Beijing Key Laboratory for Forest Pest Control, Beijing Forestry University, Beijing 100083, China

* Corresponding authors

**Email addresses:**

Yuchao Yang: yangyc68@126.com

Wenbo Li: leonardolee24@hotmail.com

Jing Tao: taojing1029@hotmail.com

Shixiang Zong: zongsx@126.com

**Table S11.** Primers used for the RT-qPCR analysis of OBPs, CSPs and SNMPs of *S. insularis*.

| **Gene name** | **Forward primer** | **Reverse primer** |
| --- | --- | --- |
| SinsOBP1 | TTTGCCTTCTCCAATTCACC | GCAAGGAGCATAGCATCACA |
| SinsOBP2 | AGCCGCTTTCAAAATCAGAA | TCGCGTACGTTCTGTAGCTC |
| SinsOBP3 | CTCCTCCATTATGCACGACA | GTGGCTGAGGTTGCAAAAAT |
| SinsOBP4 | AGCAGACCTGCGTCAAGAAT | CGACCCAAAGCTCAAATGTT |
| SinsOBP5 | CCGAAAAATGACGTCACAGA | TGGCGGAAACATCATATCAA |
| SinsOBP6 | CTGCCACTTGCGTTTAATGA | TACGCAAGGACGATGTCAAG |
| SinsOBP7 | GCCTGTGCGTACAAGATTGA | TTTCACCGTCGCTTACTTCC |
| SinsOBP8 | CGTGCCCTGTGGTATTCTTT | CGTCTCTTTCGAGCCTCTGT |
| SinsOBP9 | TTCGTAGTCGCTCAGGCTTT | CCGTCCTTGGTCATCAGTTC |
| SinsOBP10 | GTAGCGGGTTTGACGTTGAT | GTAGCATTTCTCGCGCTCTT |
| SinsOBP11 | ATTGACGATCCAGGGTTGAG | TCTTCAGGTGAGTCGCCTTT |
| SinsOBP12 | ACTGAAAATGATGGGGGACA | GTCCCTTCTCCGACAGACAC |
| SinsOBP13 | ATGCCGAAAAACGACGTTAC | CGGGTGGAAACAACATATCC |
| SinsOBP14 | ACCGGAATGATGGACGATAA | ATTTGGCTCTCTTGCAGCTC |
| SinsOBP15 | TCGTTATGGTAGCTTTTGCTG | TCAGGTCACAGAAGATTTAATAGGC |
| SinsOBP16 | GCCTTTTGAAGGGGAGCTAT | GCATGAAGATCAGCAACGAA |
| SinsOBP17 | AAAGCCAATTGGTTGGACAG | GATCGATCGCAACCTTCATT |
| SinsOBP18 | ACGCTTCACAGATGTCCTTG | GGCGTGTATCATGAAAATGG |
| SinsOBP19 | TGCCTGTTCAAGAAAATTGG | CTTGCTCGATCACACCCTTT |
| SinsOBP20 | CAAGTTAACTCAAGCGGTGAAA | AGATTGCCTCGCACATATCA |
| SinsOBP21 | GAAGGTCTGGTGGGCTTGTA | CGGTCAGATATGCACTCGAA |
| SinsOBP22 | ATCAAAGATGCCGACGAACT | AACCAGAATGCCGAACTGAG |
| SinsOBP23 | ACCGGAATTCAGTGAGGAAA | GAGACTCGCCTCCTCTAGCA |
| SinsPBP1 | CCATCAGGATCCACCAAATC | GAAAAGCGTTGGCAGATTGT |
| SinsPBP2 | CTTGGAGCGGAAGCATTTAG | AAAATGCATCATGGGAAAGC |
| SinsPBP3 | CGAATGCAAACAAGAGCTGA | TTTGCGTTTCCATGGTGTAA |
| SinsGOBP1 | CGAAAGAATGGAACGGAAAA | CGCCAGAAGTGGAAGAACTC |
| SinsGOBP2 | CTTCTCGCAATTGTGGATCA | AAGCACTTCTGGAGCGAAGA |
| SinsCSP1 | ATGTTGGACCAGGGAAAATG | CAAAGTGACGGTCTGGATCA |
| SinsCSP2 | GGCTCGTCCTGATGACAAAT | TGTGCCGTTACGTTGAGTTT |
| SinsCSP3 | GACCCTTGTGTGTCTGTCGAT | ATCTGGCACCTTGCTTCTGT |
| SinsCSP4 | TCTTCGCGCTGGTAGCTATT | GCCTTTCCTCTGTGCTTCAG |
| SinsCSP5 | ACCTGAAGGTTCCGATTTCA | TCGATTGGATGAATTTGTCG |
| SinsCSP6 | TCATAGCTTTTTGCGCATTG | ATCAATTCGCGCTGTTTAGG |
| SinsCSP7 | AAACGAACGCATCCTTCTTG | CCCTGATGCCTAGCAACATT |
| SinsCSP8 | TTGTCTGTTTGTTCGCTGTTG | ATTTGAGCTCCTTGCCTTCA |
| SinsCSP9 | CCTTGGAAAGGGCAAATGTA | TCCGGGTCGTACGTCTTTAC |
| SinsCSP10 | TTGCCTGACGCGATAACTAA | CCCTTTTCAGCGTTATGTGT |
| SinsCSP11 | ATCAACCTGGACGAAGTGCT | AGGGTCGTATTTCTCCACCA |
| SinsCSP12 | TTGTTGTTGGCCAGGACATA | GGTCCTCTTTTGTCGCTCTG |
| SinsSNMP1 | TTCGCCAGTGATCTATGCAG | ATGTGGCATGGAGACGTACA |
| SinsSNMP2 | AACAACGTGCCGAGAAGACT | ACACCCATAAGCAAGCAACC |
| Actin | CGACAGGATGCAGAAGGAAA | TAGAAGCACTTGCGGTGGAC |
